# Supplementary material for: Catalytic 1,1-diazidation of alkenes
Source: Nat Commun. 2024 Apr 29;15:3632. doi: 10.1038/s41467-024-47854-9 (PMC11058774; doi:10.1038/s41467-024-47854-9)
Supplement: Supplementary file 1 — Supplementary Information [file 41467_2024_47854_MOESM1_ESM.pdf]

Supplementary Information for

# Catalytic 1,1-diazidation of alkenes

Wangzhen Qiu[1,2], Lihao Liao[1,2*], Xinghua Xu[1], Hongtai Huang[1], Yang Xu[1], and Xiaodan Zhao[1*]

[1]Institute of Organic Chemistry and MOE Key Laboratory of Bioinorganic and Synthetic Chemistry, School of Chemistry, IGCME, Sun Yat-Sen University, Guangzhou 510006, P. R. China.

[2]These authors contributed equally.

[*]Corresponding Author(s): liaolh5@mail.sysu.edu.cn; zhaoxd3@mail.sysu.edu.cn

# Table of Contents


| | | |
|---|---|---|
| **1.** | **Supplementary Notes** | **3** |
| **2.** | **Preparation of Alkene Substrates** | **5** |
| | *2.1 Wittig Reactions* | **6** |
| | *2.2 Horner-Wadsworth-Emmons Reactions* | **9** |
| | *2.3 Suzuki-Miyaura Reactions* | **12** |
| | *2.4 Other Reactions* | **12** |
| | *2.5 Characterization Data of New Alkene Substrates* | **15** |
| **3.** | **Condition Evaluation** | **30** |
| | *3.1 Catalyst Evaluation* | **30** |
| | *3.2 Oxidant Evaluation* | **31** |
| | *3.3 Amount Evaluation* | **32** |
| | *3.4 Solvent and Z/E Configuration Evaluation* | **33** |
| **4.** | **Catalytic Oxidative 1,1-Diazidation of Alkenes** | **33** |
| | *4.1 General Procedure* | **33** |
| | *4.2 Characterization Data of Geminal Diazides* | **34** |
| | *4.3 Safety Evaluation of Geminal Diazides* | **61** |
| | *4.4 Limitation of the Developed Method* | **67** |
| **5.** | **Scale-up Synthesis and Further Transformations of the Products** | **68** |
| | *5.1 Scale-up Synthesis* | **68** |
| | *5.2 Further Transformations of the Products* | **69** |
| | *5.3 Characterization Data* | **77** |
| **6.** | **Mechanistic Studies** | **86** |
| | *6.1 Competition between 1,1- vs 1,2-Diazidation of Stilbenes* | **86** |
| | *6.2 Isotope Labeling Experiments* | **89** |
| | *6.3 Diazidation vs Carbon Deletion Reaction of Trisubstituted Alkenes* | **91** |
| | *6.4 Determination of By-products in Carbon Deletion Reaction* | **95** |
| | *6.5 Influence of Tetrafluoroborates in the Reactions* | **98** |
| | *6.6 Isolation of Possible Intermediate and Its Oxidative Azidation* | **99** |
| | *6.7 Studies of Exclusion of Aldehyde as Possible Intermediate* | **102** |
| | *6.8 In Comparison with Hypervalent Iodine System* | **104** |
| | *6.9 Comparison of Characterization Data between Geminal and Vicinal Diazides* | **108** |
| | *6.10 Supplementary Discussion* | **112** |
| **7.** | **X-Ray Crystallographic Data** | **113** |
| **8.** | **Supplementary NMR Spectra for New Compounds** | **116** |
| **9.** | **Supplementary References** | **227** |

## 1. Supplementary Notes

Unless otherwise noted, commercial reagents were purchased from Adamas, Energy Chemical, Alfa Aesar, TCI, *J&K*, or Macklin and used without further purification. Reaction solvents THF, $CH_2Cl_2$, and toluene were purified by Pure Solv MD-5 (Innovative Technology); other solvents were purchased from Guangzhou Chemical Reagent Factory or Guangdong Guanghua Sci-Tech Co., Ltd. and used without further purification. All the reactions were carried out with oven-dried glassware. All catalytic reactions were conducted without special care. Analytical thin layer chromatography was performed on 0.20 mm silica gel HSGF-254 plates (Huanghai, China), and visualized under 254 nm UV light or by staining with potassium permanganate. Preparative thin layer chromatography was performed on 0.9 mm-1.0 mm silica gel HSGF-254 plates (Huanghai, China). Column chromatography was performed on 200-300 mesh silica gel (Huanghai, China).

$^{1}H$, $^{19}F$, and $^{13}C\{^{1}H\}$ NMR spectra were recorded on a Bruker AVANCE III 400MHz or Ascend$^{TM}$ 600 MHz spectrometer at ambient temperature. $^{1}H$ NMR spectra are referred to the TMS signal (δ = 0 ppm) or residual $CHCl_3$ signal (7.26 ppm) and $^{13}C$ NMR spectra are referred to the residual solvent signal (δ = 77.16 ppm). Data for $^{1}H$ NMR are reported as follows: chemical shift (δ ppm), multiplicity (s = singlet, d = doublet, t = triplet, q = quartet, m = multiplet), coupling constant (Hz), integration. Data for $^{13}C$ NMR, and $^{19}F$ NMR are reported as follows: chemical shift (δ ppm), multiplicity (d = doublet, q = quartet), coupling constant (Hz). High resolution mass spectra of new compounds were recorded on LTQ Orbitrap Elite LC/MS (ESI) at Guangzhou Micromon Technology Services CO. LTD.

IR spectrograms were recorded using FT-IR, Bruker Vertex 70 (Diamond ATR, resolution ratio: 4 $cm^{-1}$, scanning 64 times) or FT-IR, Bruker TENSOR27 (Diamond ATR, resolution ratio: 4 $cm^{-1}$, scanning 32 times). Peaks are reported in $cm^{-1}$ with indicated relative intensities: s (strong, 0–50% T), m (medium, 51–80% T), w (weak, 81–100% T), and br (broad).

TGA-DSC plots was recorded using dry nitrogen gas at a heating rate of 10 $K\bullet min^{-1}$ on TA-SDT650. Impact sensitivity measurements were carried out using a standard BAM Fallhammer and a BAM friction tester by China National Quality Inspection and Testing

Center of Explosion-proof Equipment (Guangdong), Guangzhou Academy of Special Equipment Inspection and Testing.

# 2. Preparation of Alkene Substrates

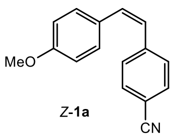

*Z*-**1a**

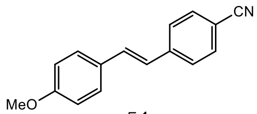

*E*-**1a**

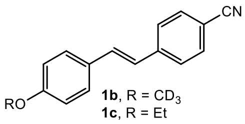

**1b**, R = $CD_3$
**1c**, R = Et

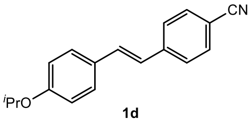

**1d**

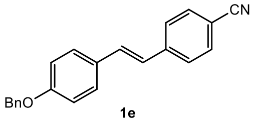

**1e**

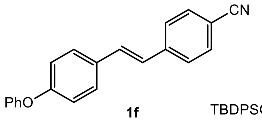

**1f**

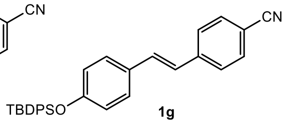

**1g**

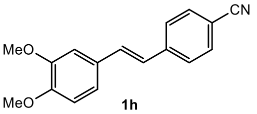

**1h**

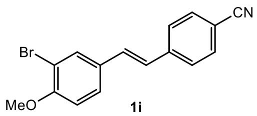

**1i**

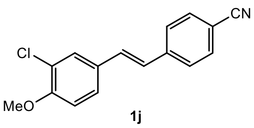

**1j**

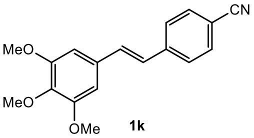

**1k**

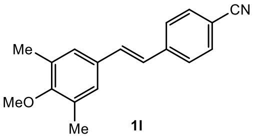

**1l**

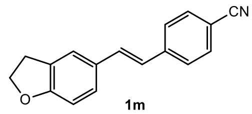

**1m**

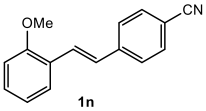

**1n**

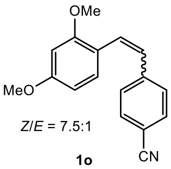

*Z*/*E* = 7.5:1
**1o**

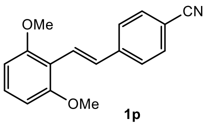

**1p**

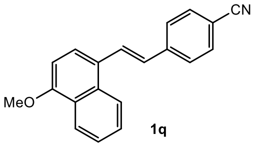

**1q**

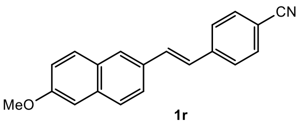

**1r**

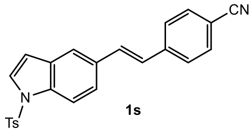

**1s**

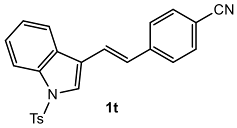

**1t**

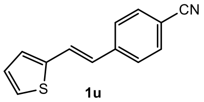

**1u**

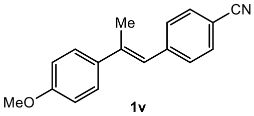

**1v**

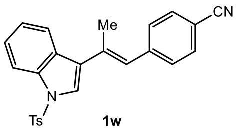

**1w**

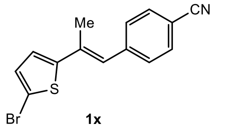

**1x**

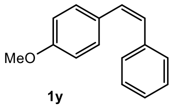

**1y**

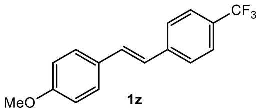

**1z**

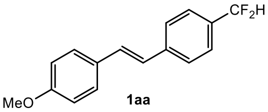

**1aa**

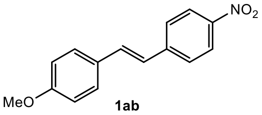

**1ab**

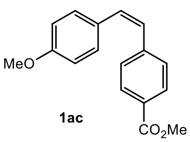

**1ac**

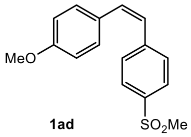

**1ad**

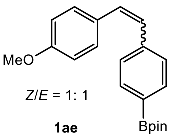

*Z*/*E* = 1: 1
**1ae**

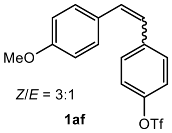

*Z*/*E* = 3:1
**1af**

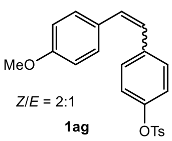

*Z*/*E* = 2:1
**1ag**

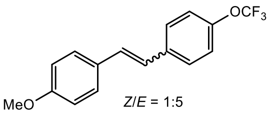

*Z*/*E* = 1:5
**1ah**

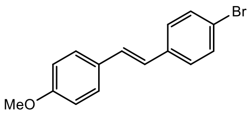

**1ai**

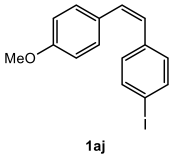

**1aj**

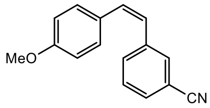

**1ak**

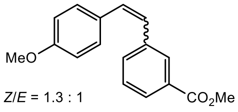

*Z*/*E* = 1.3 : 1
**1al**

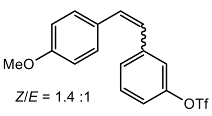

*Z*/*E* = 1.4 :1
**1am**

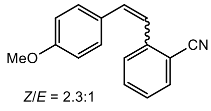

*Z*/*E* = 2.3:1
**1an**

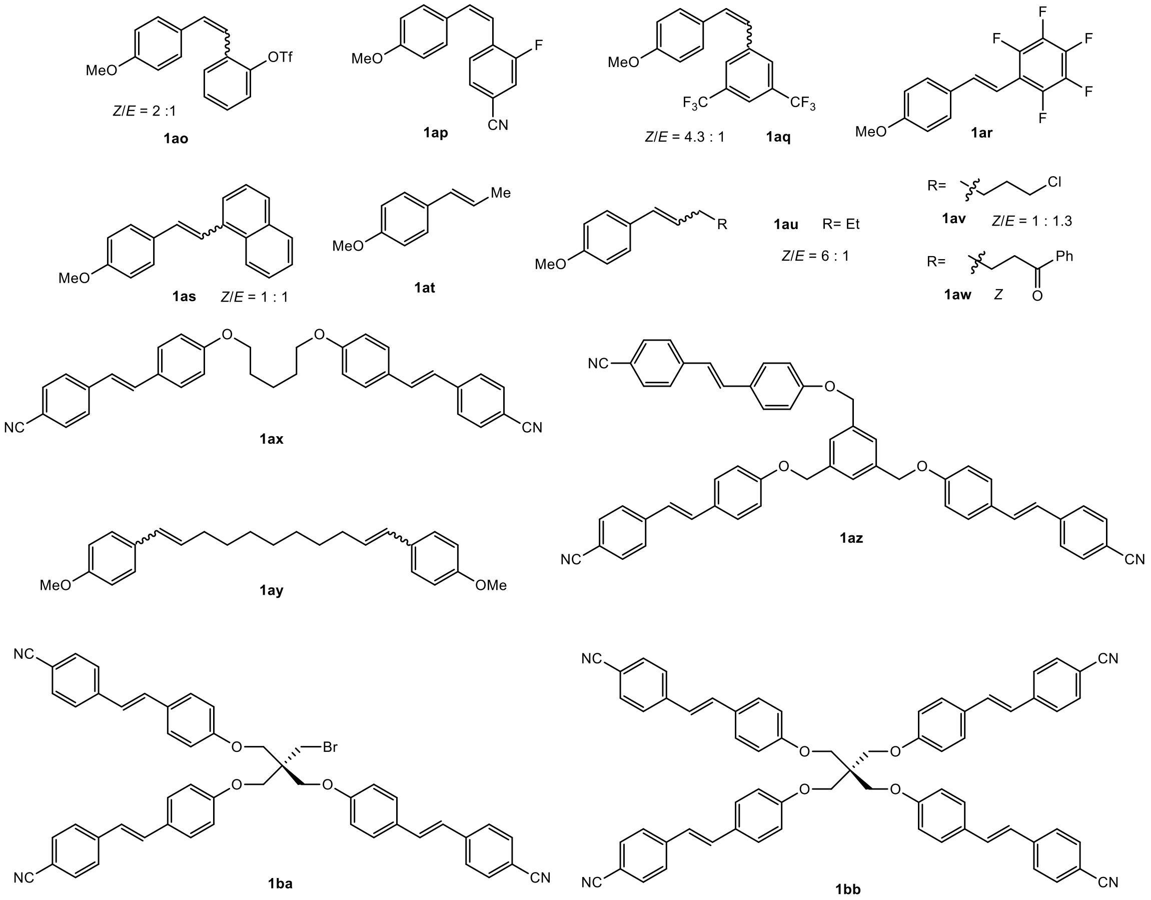


**Supplementary Figure 1.** Substrates used in reactions

Alkene **1at** (*trans*-anethole) is commercially available. Other alkenes were prepared mainly according to the Wittig reactions, Horner-Wadsworth-Emmons reactions, and Suzuki-Miyaura reactions. Generally, Wittig reactions generate *Z*-type alkenes as major products; Horner-Wadsworth-Emmons reactions generate *E*-type alkenes as major products; Suzuki-Miyaura reactions generate alkenes with the same configuration as the corresponding alkenyl substrates.

## 2.1 Wittig Reactions

### 2.1.1 Reaction of Aldehydes with (4-Methoxybenzyl)triphenylphosphonium Chloride

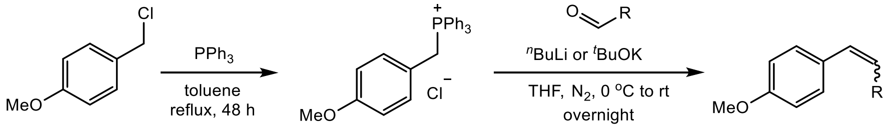


(4-Methoxybenzyl)triphenylphosphonium chloride was prepared according to the

literature procedure.[1] Some alkenes were prepared via Wittig reactions of aldehydes with (4-methoxybenzyl)triphenylphosphonium chloride.

(1) $^{n}$BuLi as base: to a suspension of (4-methoxybenzyl)triphenylphosphonium chloride (501 mg, 1.2 mmol, 1.2 equiv) in dry THF (4 mL) at -78 $^{o}$C under $N_2$ was added $^{n}$BuLi (1.6 mol/L in hexane, 0.75 mL, 1.2 equiv). The reaction was stirred at -78 $^{o}$C for 30 min, and then a solution of aldehyde (1 mmol) in dry THF (1 mL) was added dropwise. The mixture was warmed to room temperature and stirred overnight. Water (5 mL) was added carefully to quench the reaction and the mixture was extracted with EtOAc (20 mL x 3). The combined organic layers were washed with brine, dried over $Na_2SO_4$, and concentrated under reduced pressure. The residue was purified by flash column chromatography to give alkenes *Z*-**1a**, *E*-**1a**, **1y**, **1z**, **1ab**, **1ac**, **1ad, 1af**, **1ah**, **1ai**, **1aj**, **1ak**, **1an**, and **1as**. Alkenes *Z*-**1a**[2], *E*-**1a**[2], **1y**[3], **1z**[4], **1ab**[4], **1ac**[4], **1ad**[5]**, 1ah**[6], **1ai**[7], **1aj**[8], **1ak**[9], **1an**[10], and **1as**[11] are known compounds.

(2) $^{t}$BuOK as base: to a suspension of (4-methoxybenzyl)triphenylphosphonium chloride (543 mg, 1.3 mmol, 1.3 equiv) in dry THF (4 mL) at 0 $^{o}$C under $N_2$ was added $^{t}$BuOK (146 mg, 1.3 mmol, 1.3 equiv). The reaction was stirred at 0 $^{o}$C for 30 min, and then a solution of aldehyde (1 mmol) in dry THF (1 mL) was added dropwise. The mixture was warmed to room temperature and stirred overnight. Water (5 mL) was added carefully to quench the reaction and the mixture was extracted with EtOAc (20 mL x 3). The combined organic layers were washed with brine, dried over $Na_2SO_4$, and concentrated under reduced pressure. The residue was purified by flash column chromatography to give alkenes **1ag**, **1al**, **1am**, **1ao**, **1ap**, **1aq**, **1av**, and **1ax**. Alkenes **1al**[12], **1aq**[13] ,**1av**[14] are known compounds.

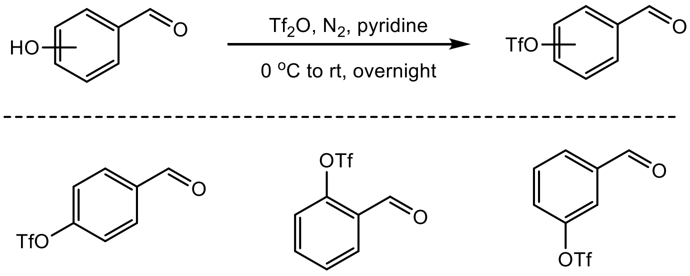

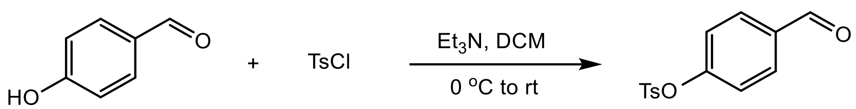


TfO and TsO-substituted aromatic aldehydes were prepared via sulfonylation of the corresponding phenols according to the literature procedure[15,16].

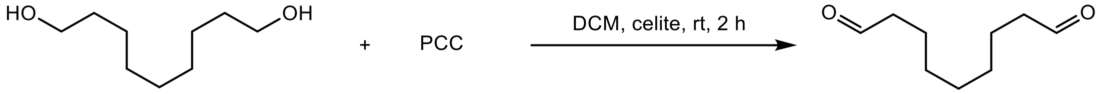


Nonanedial were prepared via oxidation of nonane-1,9-diol by PCC according to the literature procedure[17].

Other aldehydes are commercially available.

## 2.1.2 Preparation of Alkene 1o

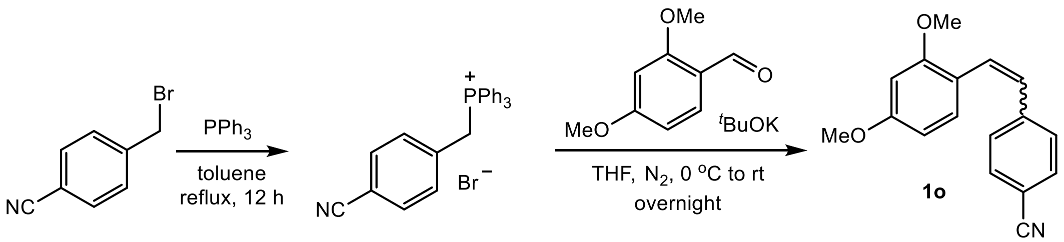


(4-Cyanobenzyl)triphenylphosphonium bromide was prepared similar to the synthesis of (4-methoxybenzyl)triphenylphosphonium chloride. Reaction of 2,4-dimethoxybenzaldehyde with (4-cyanobenzyl)triphenylphosphonium bromide using ${}^{t}$BuOK as base gave alkene **1o.**

## 2.1.3 Preparation of Alkene 1au

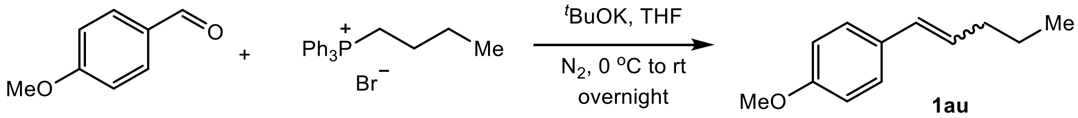


Reaction of *p*-anisaldehyde with butyltriphenylphosphonium bromide using ${}^{t}$BuOK as

base gives alkene **1au**. Alkene **1au**[18] is a known compound.

## 2.2 Horner-Wadsworth-Emmons Reactions

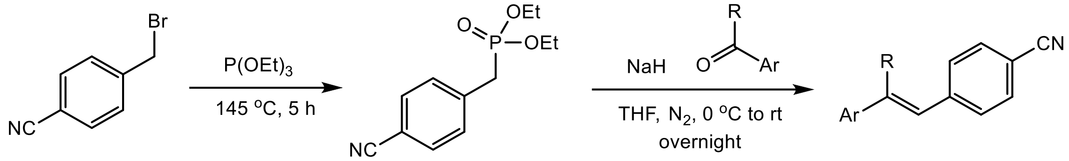


Diethyl (4-cyanobenzyl)phosphonate was prepared via Michaelis-Arbuzov reaction according to the literature procedure.[19] Some alkenes were prepared via Horner-Wadsworth-Emmons reaction of aldehydes or ketones with diethyl (4-cyanobenzyl)phosphonate.

To a suspension of NaH (60% dispersion in mineral oil, 120 mg, 3.0 mmol, 3.0 equiv) in dry THF (4 mL) at 0 °C under $N_2$ was added diethyl (4-cyanobenzyl)phosphonate (256 $\mu$L, 1.2 mmol, 1.2 equiv) dropwise. The reaction was stirred at 0 °C for 30 min, and then a solution of aldehyde or ketone (1 mmol) in dry THF (1 mL) was added dropwise. The mixture was warmed to room temperature and stirred overnight. Water (5 mL) was added carefully to quench the reaction and the mixture was extracted with EtOAc (20 mL x 3). The combined organic layers were washed with brine, dried over $Na_2SO_4$, and concentrated under reduced pressure. The residue was purified by flash column chromatography to give alkenes *E*-**1a, 1b-1e**, **1g-1n**, **1p-1r**, **1t**, **1v-1x**, **1ax**, and **1az-1bb**. Alkenes **1h**[20], **1k**[10], and **1n**[21] are known compounds.

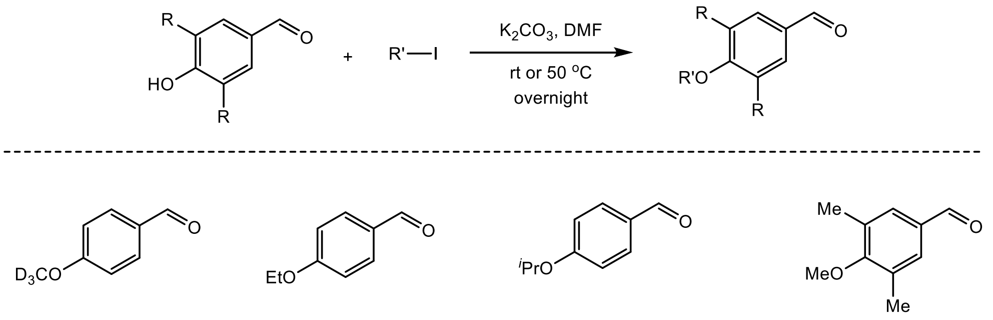


Some alkoxy substituted aromatic aldehydes were prepared via alkylation of the corresponding phenols with alkyl iodides according to the literature procedure[22].

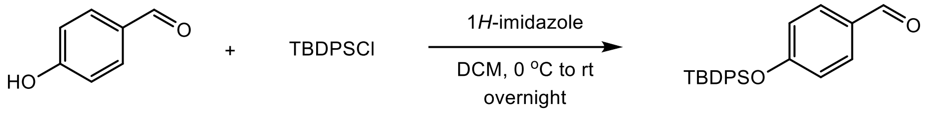


TBDPSO-substituted aromatic aldehyde was prepared via silylation of the corresponding phenol according to the literature procedure[23].

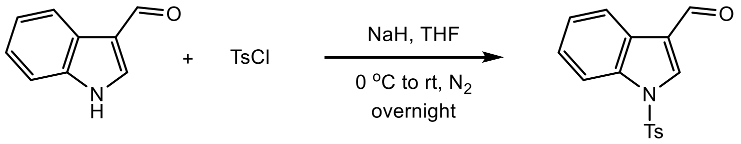


1-Tosyl-1*H*-indole-3-carbaldehyde was prepared via sulfonylation of 1*H*-indole-3-carbaldehyde according to the literature procedure[24].

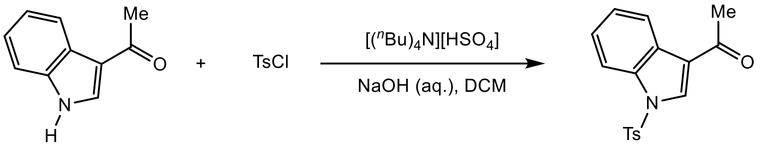


1-(1-Tosyl-1*H*-indol-3-yl)ethan-1-one was prepared via sulfonylation of 1-(1*H*-indol-3-yl)ethan-1-one according to the literature procedure[25].

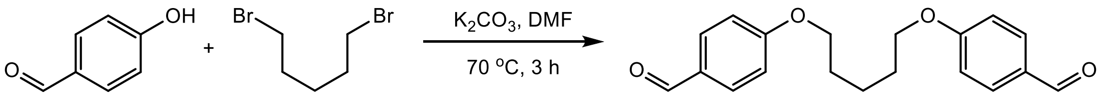


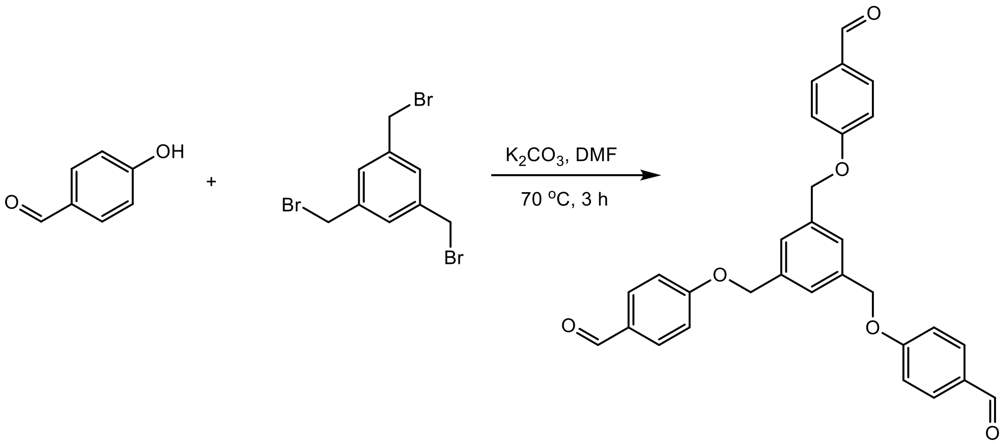

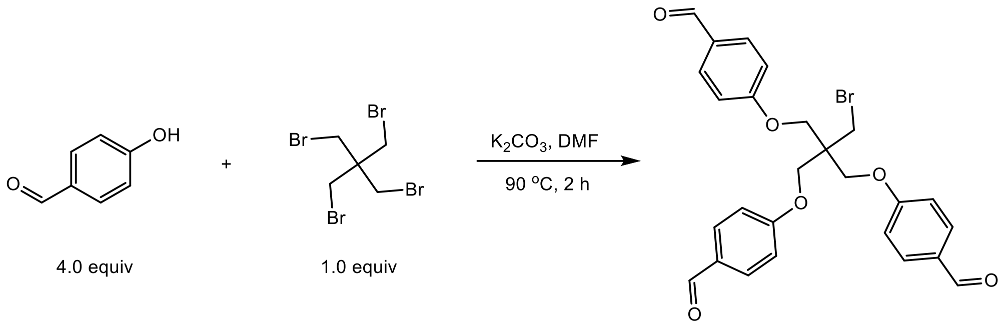


Dimeric and trimeric aromatic aldehydes were prepared via alkylation of the corresponding phenols with alkyl bromides using $K_2CO_3$ as base according to the literature procedure[26].

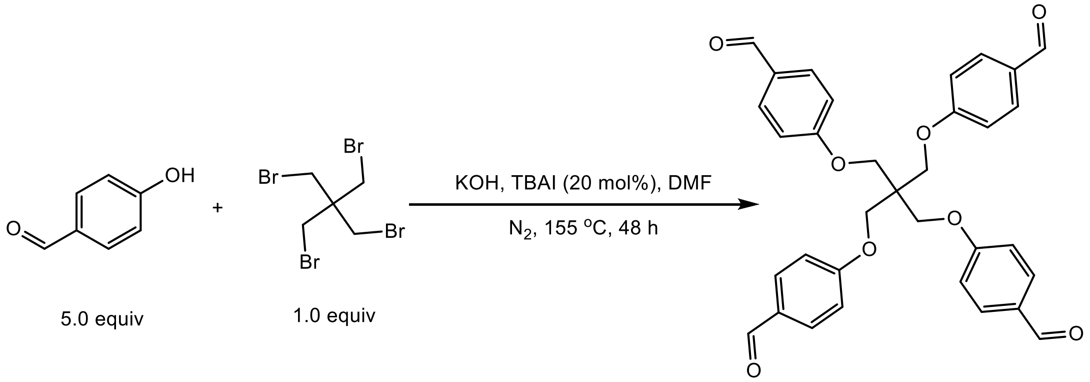


Tetrameric aromatic aldehyde was prepared via alkylation of corresponding phenols with alkyl bromide using KOH as base according to the literature procedure[27].

Other aldehydes and ketones are commercially available.

## 2.3 Suzuki-Miyaura Reactions

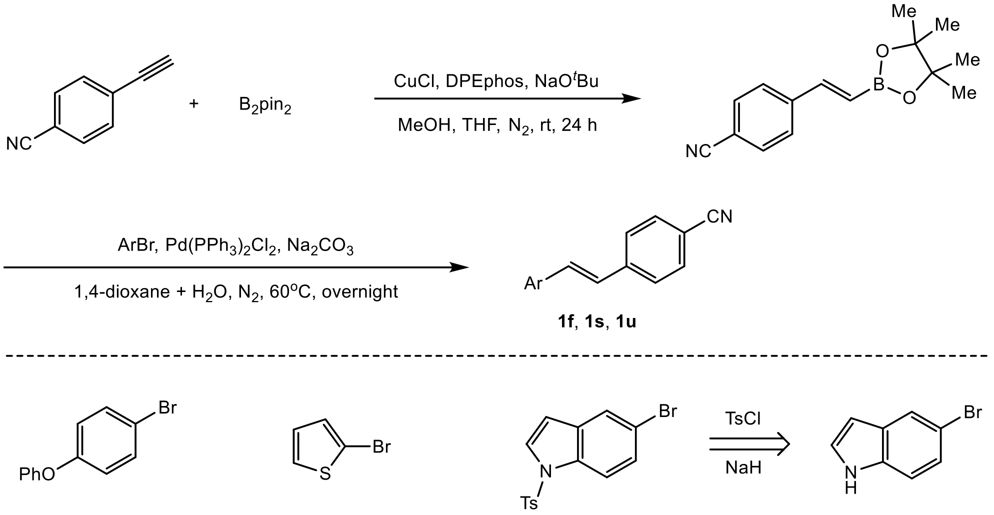


($E$)-4-(2-(4,4,5,5-Tetramethyl-1,3,2-dioxaborolan-2-yl)vinyl)benzonitrile was prepared via hydroboration of alkyne according to the literature procedure[28]. Some alkenes are prepared via Suzuki-Miyaura reaction of aryl bromides with ($E$)-4-(2-(4,4,5,5-tetramethyl-1,3,2-dioxaborolan-2-yl) vinyl)benzonitrile.

To a solution of $Pd(PPh_3)_2Cl_2$ (17.5 mg, 0.025 mmol, 5 mol%), $Na_2CO_3$ (212 mg, 2 mmol, 4.0 equiv), ($E$)-4-(2-(4,4,5,5-Tetramethyl-1,3,2-dioxaborolan-2-yl)vinyl)benzonitrile (140 mg, 0.55 mmol, 1.1 equiv) in dioxane-water(4 mL+1.6 mL) under $N_2$ was added aryl bromide (0.5 mmol, 1.0 equiv). The reaction was stirred at 60 $^oC$ overnight. After cooled to room temperature, the reaction was quenched by saturated $NH_4Cl$ aqueous solution (5 mL) and the mixture was extracted with EtOAc (20 mL x 3). The combined organic layers were washed with brine, dried over $Na_2SO_4$, and concentrated under reduced pressure. The residue was purified by flash column chromatography to give alkenes **1f**, **1s**, and **1u**. 5-Bromo-1-tosyl-1$H$-indole was prepared via sulfonylation of 5-bromo-1$H$-indole according to the literature procedure[24]. Alkene **1u**[29] is a known compound.

## 2.4 Other Reactions

### 2.4.1 Synthesis of 1aa

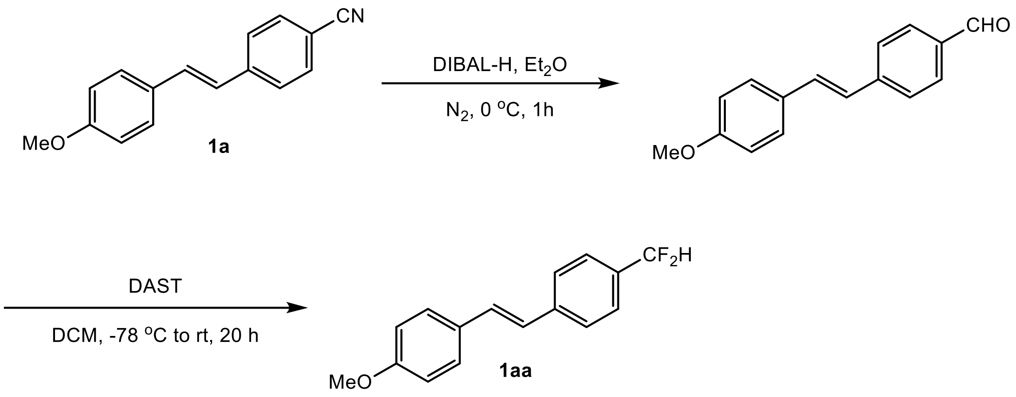


Alkene **1aa** was prepared via reduction of nitrile **1a** by DIBAL-H to aldehyde, followed by deoxyfluorination by DAST, according to the literature procedure[30,31]. **1aa** is a known compound[31].

## 2.4.2 Synthesis of 1ae

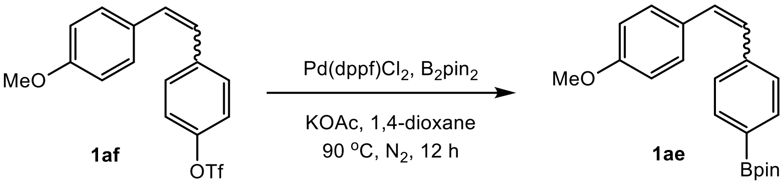


Alkene **1ae** was prepared via Miyaura boronation of trifluoromethanesulfonate **1af** according to the literature procedure[32]. **1af** is a known compound[33].

## 2.4.3 Synthesis of 1ar

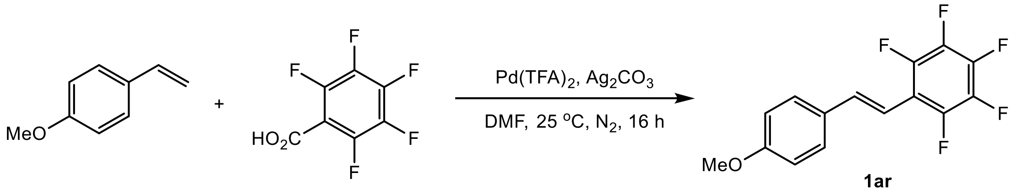


Alkene **1ar** was prepared via decarboxylative Heck reaction according to the literature procedure[34]. **1ar** is a known compound[34].

## 2.4.4 Synthesis of 1aw

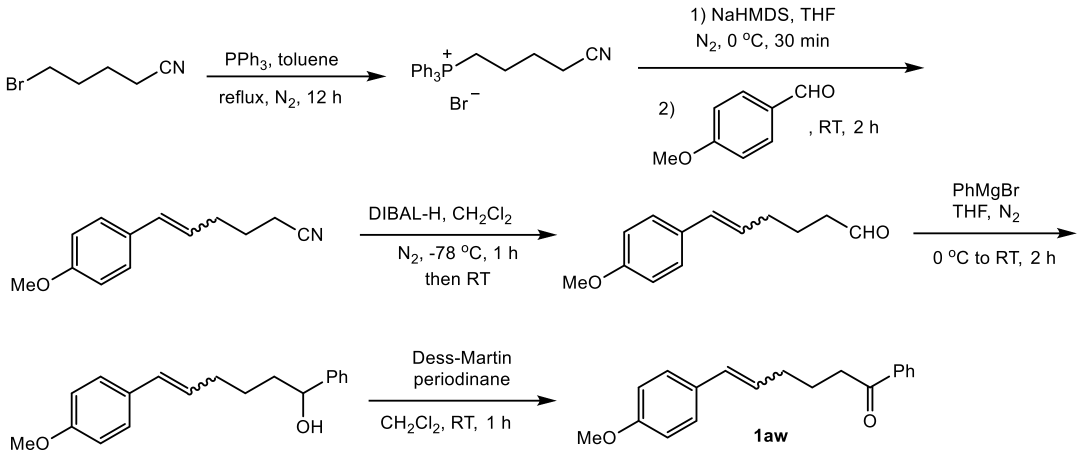


To a solution of $PPh_3$ (2623 mg, 10 mmol) in toluene (10 mL) under $N_2$ was added 5-bromovaleronitrile (1168 $\mu$L, 10 mmol) at room temperature. The reaction was stirred at reflux for 12 h. After cooled to room temperature, the solvent was removed under reduced pressure. To a solution of the obtained phosphonium salt in dry THF (20 mL) under $N_2$ at 0 $^oC$ was added NaHMDS (12 mL, 1.0 M in THF, 12 mmol, 1.3 equiv) dropwise, and the reaction was stirred at the same temperature for 30 min. *p*-Anisaldehyde (1093 µL, 9 mmol, 1.0 equiv) was added and the reaction was warmed to room temperature and stirred for 2 h. The reaction was quenched by water and the mixture was extracted with EtOAc. The combined organic layers were washed with brine, dried over $Na_2SO_4$, and concentrated under reduced pressure. The residue was purified by flash column chromatography (eluent: PE/EA = 30:1 to 10:1, v/v) to give nitrile-containing alkene (1093.8 mg, 5.43 mmol, 60%) as a yellow oil.

To a solution of nitrile-containing alkene (1093.8 mg, 5.43 mmol, 1.0 equiv) in dry DCM (40 mL) under $N_2$ at -78 $^oC$ was added DIBAL-H (13.6 mL, 1.0 M in hexane, 2.5 equiv) dropwise. The reaction was stirred at -78 $^oC$ for 1 h. The reaction was warmed to room temperature and was carefully quenched by ethanol and 1M HCl. The mixture was extracted with DCM and the combined organic layers were washed with brine, dried over $Na_2SO_4$, and concentrated under reduced pressure. The residue was purified by flash column chromatography (eluent: PE/EA = 50:1 to 30:1, v/v) to give aldehyde-containing alkene (495.4 mg, 2.43 mmol, 45%) as a yellow oil.

To a solution of aldehyde-containing alkene (204.3 mg, 1 mmol, 1.0 equiv) in dry THF (4 mL) under $N_2$ at 0 $^oC$ was added PhMgBr (1.2 mL, 1.0 M in THF, 1.2 mmol, 1.2 equiv)

dropwise. The reaction was warmed to room temperature and stirred for 2 h. The reaction was quenched by saturated aqueous $NH_4Cl$. The mixture was extracted with EtOAc and the combined organic layers were washed with brine, dried over $Na_2SO_4$, and concentrated under reduced pressure. To a solution of the obtained crude alcohol in DCM (5 mL) was added Dess-Martin periodinane (466.6 mg, 1.1 mmol, 1.1 equiv). The reaction was stirred at room temperature for 1 h and the solvent was removed under reduced pressure. The residue was purified by flash column chromatography (eluent: PE/EA = 50:1 to 20:1, v/v) to give ketone-containing alkene **1aw** (177.8 mg, 0.63 mmol, 63%) as a light-yellow oil.

## 2.5 Characterization Data of New Alkene Substrates

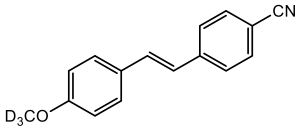


**(*E*)-4-(4-(Methoxy-*d*3)styryl-*d*3)benzonitrile (1b)**

Prepared according to procedure **2.2**. Flash column chromatography on silica gel (eluent: PE/EA = 5:1 to 3:1, v/v) to afford **1b** as a white solid (202.6 mg, 85%, 1 mmol scale). $R_f$ = 0.3 (PE/EA = 5:1, v/v). $^1$H NMR (600 MHz, $CDCl_3$) δ 7.61 (d, *J* = 8.0 Hz, 2H), 7.55 (d, *J* = 8.1 Hz, 2H), 7.48 (d, *J* = 8.7 Hz, 2H), 7.17 (d, *J* = 16.3 Hz, 1H), 6.95 (d, *J* = 16.3 Hz, 1H), 6.92 (d, *J* = 8.6 Hz, 2H). $^{13}$C NMR (151 MHz, $CDCl_3$) δ 160.22, 142.37, 132.60, 132.10, 129.19, 128.42, 126.70, 124.68, 119.31, 114.43, 110.19. HR-ESI-MS m/z calcd for $C_{16}H_{10}D_3NONa$ [(M+Na)$^+$]: 261.1078, found: 261.1087.

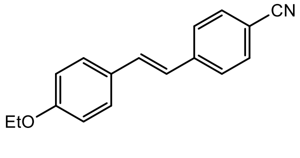


**(*E*)-4-(4-Ethoxystyryl)benzonitrile (1c)**

Prepared according to procedure **2.2**. Flash column chromatography on silica gel (eluent: PE/EA = 15:1 to 8:1, v/v) to afford **1c** as a white solid (243.1 mg, 98%, 1 mmol scale). $R_f$

= 0.6 (PE/EA = 10:1, v/v). $^{1}$H NMR (400 MHz, $CDCl_3$) δ 7.61 (d, *J* = 8.4 Hz, 2H), 7.54 (d, *J* = 8.4 Hz, 2H), 7.46 (d, *J* = 8.7 Hz, 2H), 7.16 (d, *J* = 16.3 Hz, 1H), 6.94 (d, *J* = 16.8 Hz, 1H), 6.91 (d, *J* = 8.9 Hz, 2H), 4.07 (q, *J* = 7.0 Hz, 2H), 1.43 (t, *J* = 7.0 Hz, 3H). $^{13}$C NMR (101 MHz, $CDCl_3$) δ 159.67, 142.44, 132.59, 132.20, 129.08, 128.42, 126.69, 124.59, 119.28, 115.00, 110.20, 63.73, 14.93. HR-ESI-MS m/z calcd for $C_{17}H_{15}NONa$ [$(M+Na)^+$]: 272.1051, found: 272.1059.

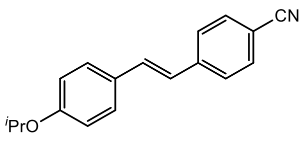


**(*E*)-4-(4-Isopropoxystyryl)benzonitrile (1d)**

Prepared according to procedure **2.2**. Flash column chromatography on silica gel (eluent: PE/EA = 15:1 to 8:1, v/v) to afford **1d** as a white solid (222.8 mg, 85%, 1 mmol scale). $R_f$ = 0.6 (PE/EA = 10:1, v/v). $^{1}$H NMR (600 MHz, $CDCl_3$) δ 7.61 (d, *J* = 8.3 Hz, 2H), 7.54 (d, *J* = 8.5 Hz, 2H), 7.46 (d, *J* = 8.7 Hz, 2H), 7.16 (d, *J* = 16.2 Hz, 1H), 6.94 (d, *J* = 16.3 Hz, 1H), 6.90 (d, *J* = 8.7 Hz, 2H), 4.59 (hept, *J* = 6.1 Hz, 1H), 1.36 (d, *J* = 6.1 Hz, 6H). $^{13}$C NMR (151 MHz, $CDCl_3$) δ 158.61, 142.43, 132.59, 132.17, 128.90, 128.43, 126.67, 124.50, 119.32, 116.17, 110.11, 70.10, 22.15. HR-ESI-MS m/z calcd for $C_{18}H_{17}NONa$ [$(M+Na)^+$]: 286.1208, found: 286.1209.

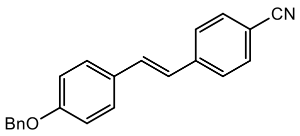


**(*E*)-4-(4-(Benzyloxy)styryl)benzonitrile (1e)**

Prepared according to procedure **2.2**. Recrystallization from EA/PE solution to afford **1e** as a white solid (238.4 mg, 76%, 1 mmol scale). $R_f$ = 0.5 (PE/EA = 10:1, v/v). $^{1}$H NMR (400 MHz, $CDCl_3$) δ 7.61 (d, *J* = 8.3 Hz, 2H), 7.54 (d, *J* = 8.4 Hz, 2H), 7.49 – 7.32 (m, 7H), 7.16 (d, *J* = 16.2 Hz, 1H), 6.99 (d, *J* = 8.8 Hz, 2H), 6.95 (d, *J* = 16.3 Hz, 1H), 5.11 (s, 2H). $^{13}$C NMR (101 MHz, $CDCl_3$) δ 159.45, 142.37, 136.86, 132.61, 132.09, 129.51,

128.80, 128.44, 128.24, 127.60, 126.73, 124.87, 119.27, 115.42, 110.29, 70.27. HR-ESI-MS m/z calcd for $C_{22}H_{17}NONa$ [(M+Na)$^+$]: 334.1208, found: 334.1201.

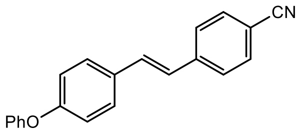


**(*E*)-4-(4-Phenoxystyryl)benzonitrile (1f)**

Prepared according to procedure **2.3**. Flash column chromatography on silica gel (eluent: PE/EA = 30:1 to 10:1, v/v) to afford **1f** as a light-yellow solid (88.8 mg, 60%, 0.5 mmol scale). $R_f$ = 0.6 (PE/EA = 10:1, v/v). $^1$H NMR (400 MHz, $CDCl_3$) δ 7.63 (d, *J* = 8.2 Hz, 2H), 7.56 (d, *J* = 8.3 Hz, 2H), 7.50 (d, *J* = 8.6 Hz, 2H), 7.37 (t, *J* = 7.9 Hz, 2H), 7.22 – 7.12 (m, 2H), 7.09 – 6.95 (m, 5H). $^{13}$C NMR (101 MHz, $CDCl_3$) δ 158.09, 156.82, 142.12, 132.63, 131.79, 131.49, 130.01, 128.51, 126.86, 125.88, 123.89, 119.44, 119.18, 118.98, 110.57. HR-ESI-MS m/z calcd for $C_{21}H_{15}NONa$ [(M+Na)$^+$]: 320.1051, found: 320.1047.

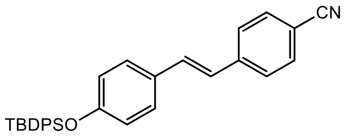


**(*E*)-4-(4-((*tert*-Butyldiphenylsilyl)oxy)styryl)benzonitrile (1g)**

Prepared according to procedure **2.2**. Flash column chromatography on silica gel (eluent: PE/EA = 50:1 to 20:1, v/v) to afford **1g** as a white solid (319.9 mg, 99%, 0.7 mmol scale). $R_f$ = 0.3 (PE/EA = 20:1, v/v). $^1$H NMR (400 MHz, $CDCl_3$) δ 7.76 – 7.71 (m, 4H), 7.59 (d, *J* = 8.4 Hz, 2H), 7.50 (d, *J* = 8.4 Hz, 2H), 7.47 – 7.42 (m, 2H), 7.41 – 7.36 (m, 4H), 7.28 (d, 2H), 7.09 (d, *J* = 16.3 Hz, 1H), 6.87 (d, *J* = 16.3 Hz, 1H), 6.80 – 6.75 (m, 2H), 1.12 (s, 9H). $^{13}$C NMR (101 MHz, $CDCl_3$) δ 156.42, 142.38, 135.62, 134.94, 132.81, 132.57, 132.19, 130.15, 129.53, 128.18, 127.98, 126.68, 124.78, 120.30, 119.26, 26.64, 19.62. HR-ESI-MS m/z calcd for $C_{31}H_{29}NOSiNa$ [(M+Na)$^+$]: 482.1916, found: 482.1915.

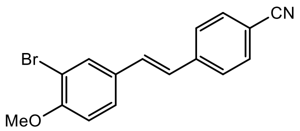


**(*E*)-4-(3-Bromo-4-methoxystyryl)benzonitrile (1i)**

Prepared according to procedure **2.2**. Flash column chromatography on silica gel (eluent: PE/EA = 8:1 to 4:1, v/v) to afford **1i** as a white solid (346.6 mg, 99%, 1 mmol scale). $R_f$ = 0.35 (PE/EA = 5:1, v/v). $^1$H NMR (600 MHz, $CDCl_3$) δ 7.76 (d, *J* = 2.2 Hz, 1H), 7.62 (d, *J* = 8.2 Hz, 2H), 7.54 (d, *J* = 8.1 Hz, 2H), 7.42 (dd, *J* = 8.5, 2.2 Hz, 1H), 7.09 (d, *J* = 16.3 Hz, 1H), 6.95 (d, *J* = 16.4 Hz, 1H), 6.91 (d, *J* = 8.5 Hz, 1H), 3.93 (s, 3H). $^{13}$C NMR (151 MHz, $CDCl_3$) δ 156.25, 141.83, 132.65, 131.51, 130.61, 130.59, 127.63, 126.85, 126.01, 119.16, 112.41, 112.06, 110.63, 56.49. HR-ESI-MS m/z calcd for $C_{16}H_{12}NOBrNa$ [$(M+Na)^+$]: 336.0000, found: 335.9994.

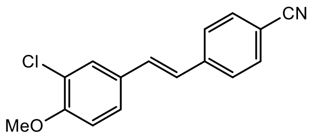


**(*E*)-4-(3-Chloro-4-methoxystyryl)benzonitrile (1j)**

Prepared according to procedure **2.2**. Flash column chromatography on silica gel (eluent: PE/EA = 8:1 to 4:1, v/v) to afford **1j** as a white solid (252.5 mg, 94%, 1 mmol scale). $R_f$ = 0.4 (PE/EA = 5:1, v/v). $^1$H NMR (600 MHz, $CDCl_3$) δ 7.62 (d, *J* = 8.5 Hz, 2H), 7.58 (s, 1H), 7.54 (d, *J* = 8.3 Hz, 2H), 7.37 (d, *J* = 8.5 Hz, 1H), 7.09 (d, *J* = 16.3 Hz, 1H), 6.99 – 6.91 (m, 2H), 3.93 (s, 3H). $^{13}$C NMR (151 MHz, $CDCl_3$) δ 155.37, 141.83, 132.65, 130.74, 130.12, 128.37, 126.92, 126.85, 126.00, 123.19, 119.16, 112.24, 110.63, 56.39. HR-ESI-MS m/z calcd for $C_{16}H_{12}NOClNa$ [$(M+Na)^+$]: 292.0505, found: 292.0505.

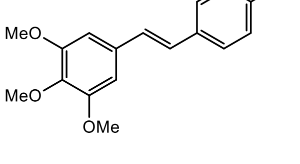


**(*E*)-4-(4-Methoxy-3,5-dimethylstyryl)benzonitrile (1l)**

Prepared according to procedure **2.2**. Flash column chromatography on silica gel (eluent: PE/EA = 30:1 to 10:1, v/v) to afford **1l** as a white solid (208.5 mg, 79%, 1 mmol scale). $R_f$ = 0.45 (PE/EA = 10:1, v/v). $^1$H NMR (600 MHz, $CDCl_3$) δ 7.62 (d, *J* = 8.2 Hz, 2H), 7.55 (d, *J* = 8.2 Hz, 2H), 7.20 (s, 2H), 7.11 (d, *J* = 16.3 Hz, 1H), 6.97 (d, *J* = 16.3 Hz, 1H), 3.74 (s, 3H), 2.32 (s, 6H). $^{13}$C NMR (151 MHz, $CDCl_3$) δ 157.76, 142.26, 132.60, 132.19, 131.97, 131.48, 127.62, 126.79, 125.70, 119.26, 110.34, 59.92, 16.33. HR-ESI-MS m/z calcd for $C_{18}H_{17}NONa$ [$(M+Na)^+$]: 286.1208, found: 286.1204.

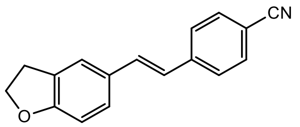


**(*E*)-4-(2-(2,3-Dihydrobenzofuran-5-yl)vinyl)benzonitrile (1m)**

Prepared according to procedure **2.2**. Flash column chromatography on silica gel (eluent: PE/EA = 8:1 to 3:1, v/v) to afford **1m** as a white solid (250.8 mg, 99%, 1 mmol scale). $R_f$ = 0.4 (PE/EA = 5:1, v/v). $^1$H NMR (600 MHz, $CDCl_3$) δ 7.61 (d, *J* = 8.0 Hz, 2H), 7.53 (d, *J* = 8.1 Hz, 2H), 7.42 (s, 1H), 7.28 (dd, *J* = 8.3, 1.9 Hz, 1H), 7.16 (d, *J* = 16.2 Hz, 1H), 6.92 (d, *J* = 16.3 Hz, 1H), 6.79 (d, *J* = 8.2 Hz, 1H), 4.62 (t, *J* = 8.7 Hz, 2H), 3.25 (t, *J* = 8.7 Hz, 3H). $^{13}$C NMR (151 MHz, $CDCl_3$) δ 161.01, 142.49, 132.59, 132.53, 129.32, 128.07, 128.03, 126.61, 124.06, 123.26, 119.34, 110.03, 109.74, 71.79, 29.62. HR-ESI-MS m/z calcd for $C_{17}H_{13}NONa$ [$(M+Na)^+$]: 270.0895, found: 270.0891.

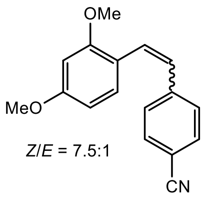


**4-(2,4-Dimethoxystyryl)benzonitrile (1o)**

Prepared according to procedure **2.1.2**. Flash column chromatography on silica gel (eluent: PE/EA = 8:1 to 5:1, v/v) to afford **1o** as a light-yellow oil (254.1 mg, 96%, 1 mmol scale). $R_f$ = 0.7 (PE/EA = 3:1, v/v). $^1$H NMR (400 MHz, $CDCl_3$) δ: *Z*-isomer: 7.51

– 7.44 (m, 2H), 7.33 (d, *J* = 8.3 Hz, 2H), 6.99 (dd, *J* = 8.5, 0.8 Hz, 1H), 6.77 (d, *J* = 12.1 Hz, 1H), 6.50 (d, *J* = 12.3 Hz, 1H), 6.46 (d, *J* = 2.4 Hz, 1H), 6.31 (dd, *J* = 8.5, 2.4 Hz, 1H), 3.80 (s, 3H), 3.79 (s, 3H); *E*-isomer: 3.88 (s, 3H), 3.84 (s, 3H). $^{13}$C NMR (101 MHz, $CDCl_3$) δ: *Z*-isomer: 161.07, 158.45, 142.81, 131.96, 130.61, 129.41, 128.95, 127.14, 119.27, 117.87, 109.93, 104.47, 98.52, 55.52, 55.48; *E*-isomer: $^{13}$C NMR (101 MHz, $CDCl_3$) δ 161.45, 158.58, 143.06, 132.49, 132.17, 129.94, 127.90, 127.24, 126.67, 124.93, 118.53, 109.72, 105.28, 55.63, 55.57. HR-ESI-MS m/z calcd for $C_{17}H_{15}NO_2Na$ [$(M+Na)^+$]: 288.1000, found: 288.0999.

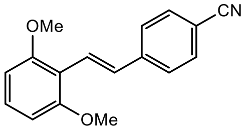


**(*E*)-4-(2,6-Dimethoxystyryl)benzonitrile (1p)**

Prepared according to procedure **2.2**. Flash column chromatography on silica gel (eluent: PE/EA = 10:1 to 3:1, v/v) to afford **1p** as a white solid (271.6 mg, 99%, 1 mmol scale). $R_f$ = 0.6 (PE/EA = 3:1, v/v). $^{1}$H NMR (400 MHz, $CDCl_3$) δ 7.61 – 7.56 (m, 6H), 7.22 (t, *J* = 8.4 Hz, 1H), 6.60 (d, *J* = 8.4 Hz, 2H), 3.91 (s, 6H). $^{13}$C NMR (101 MHz, $CDCl_3$) δ 159.13, 144.22, 132.42, 130.32, 129.40, 126.87, 123.89, 119.52, 114.09, 109.86, 104.13, 55.98. HR-ESI-MS m/z calcd for $C_{17}H_{15}NO_2Na$ [$(M+Na)^+$]: 288.1000, found: 288.0988.

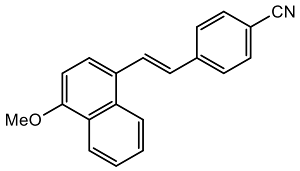


**(*E*)-4-(2-(4-Methoxynaphthalen-1-yl)vinyl)benzonitrile (1q)**

Prepared according to procedure **2.2**. Flash column chromatography on silica gel (eluent: PE/EA = 20:1 to 10:1, v/v) to afford **1q** as a light-yellow solid (258.2 mg, 90%, 1 mmol scale). $R_f$ = 0.45 (PE/EA = 10:1, v/v). $^{1}$H NMR (400 MHz, $CDCl_3$) δ 8.36 – 8.32 (m, 1H), 8.14 (d, *J* = 8.2 Hz, 1H), 7.92 (d, *J* = 16.0 Hz, 1H), 7.70 (d, *J* = 8.1 Hz, 1H), 7.67 – 7.51 (m, 6H), 7.03 (d, *J* = 16.0 Hz, 1H), 6.86 (d, *J* = 8.1 Hz, 1H), 4.04 (s, 3H). $^{13}$C NMR (101

MHz, $CDCl_3$) δ 156.32, 142.54, 132.60, 132.34, 129.54, 127.73, 127.13, 126.89, 126.49, 125.75, 125.52, 124.61, 123.27, 122.82, 119.25, 110.37, 103.96, 55.75. HR-ESI-MS m/z calcd for $C_{20}H_{15}NONa$ [$(M+Na)^+$]: 308.1051, found: 308.1047.

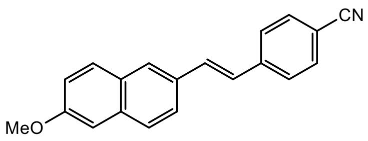


**(*E*)-4-(2-(6-Methoxynaphthalen-2-yl)vinyl)benzonitrile (1r)**

Prepared according to procedure **2.2**. Flash column chromatography on silica gel (eluent: PE/DCM = 5:1 to 1:1, v/v) to afford **1r** as a light-yellow solid (253.5 mg, 89%, 1 mmol scale). $R_f$ = 0.5 (PE/DCM = 1:1, v/v). $^1$H NMR (400 MHz, $CDCl_3$) δ 7.81 (s, 1H), 7.74 (d, *J* = 10.2 Hz, 2H), 7.69 (dd, *J* = 8.6, 1.7 Hz, 1H), 7.65 – 7.56 (m, 4H), 7.33 (d, *J* = 16.3 Hz, 1H), 7.19 – 7.10 (m, 3H), 3.93 (s, 3H). $^{13}$C NMR (101 MHz, $CDCl_3$) δ 158.37, 142.17, 134.85, 132.69, 132.60, 131.78, 129.83, 129.06, 127.65, 127.52, 126.84, 126.02, 124.02, 119.45, 119.25, 110.40, 106.08, 55.48. HR-ESI-MS m/z calcd for $C_{20}H_{15}NONa$ [$(M+Na)^+$]: 308.1051, found: 308.1049.

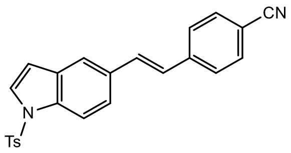


**(*E*)-4-(2-(1-Tosyl-1*H*-indol-5-yl)vinyl)benzonitrile (1s)**

Prepared according to procedure **2.3**. Flash column chromatography on silica gel (eluent: PE/EA = 5:1 to 3:1, v/v) to afford **1s** as a yellow solid (95.8 mg, 48%, 0.5 mmol scale). $R_f$ = 0.25 (PE/EA = 5:1, v/v). $^1$H NMR (600 MHz, $CDCl_3$) δ 7.99 (d, *J* = 8.6 Hz, 1H), 7.77 (d, *J* = 8.5 Hz, 2H), 7.66 (d, *J* = 1.7 Hz, 1H), 7.63 (d, *J* = 8.4 Hz, 2H), 7.59 – 7.54 (m, 3H), 7.52 (dd, *J* = 8.7, 1.8 Hz, 1H), 7.29 – 7.21 (m, 3H), 7.06 (d, *J* = 16.3 Hz, 1H), 6.66 (dd, *J* = 3.7, 0.8 Hz, 1H), 2.34 (s, 3H). $^{13}$C NMR (151 MHz, $CDCl_3$) δ 145.28, 142.05, 135.32, 134.98, 132.64, 132.54, 131.95, 131.42, 130.09, 127.37, 126.95, 126.87, 126.26, 123.47, 120.30, 119.20, 114.03, 110.54, 109.26, 21.72. HR-ESI-MS m/z calcd for

$C_{24}H_{18}N_2O_2SNa$ [(M+Na)$^+$]: 421.0987, found: 421.0988.

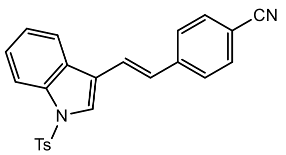


**(*E*)-4-(2-(1-Tosyl-1*H*-indol-3-yl)vinyl)benzonitrile (1t)**

Prepared according to procedure **2.2**. Flash column chromatography on silica gel (eluent: PE/EA = 5:1 to 2:1, v/v) to afford **1t** as a yellow solid (204.5 mg, 51%, 1 mmol scale). $R_f$ = 0.6 (PE/EA = 3:1, v/v). $^1$H NMR (400 MHz, $CDCl_3$) δ 8.03 (d, *J* = 8.0 Hz, 1H), 7.83 (d, *J* = 7.5 Hz, 1H), 7.81 – 7.78 (m, 3H), 7.64 (d, *J* = 8.4 Hz, 2H), 7.57 (d, *J* = 8.4 Hz, 2H), 7.43 – 7.28 (m, 3H), 7.24 (d, *J* = 8.1 Hz, 2H), 7.15 (d, *J* = 16.5 Hz, 1H), 2.34 (s, 3H). $^{13}$C NMR (101 MHz, $CDCl_3$) δ 145.43, 142.05, 135.76, 135.15, 132.67, 130.15, 128.74, 127.69, 127.05, 126.68, 125.46, 125.35, 123.93, 123.24, 120.46, 120.04, 119.12, 114.06, 110.71, 21.71. HR-ESI-MS m/z calcd for $C_{24}H_{18}N_2O_2SNa$ [(M+Na)$^+$]: 421.0987, found: 421.0986.

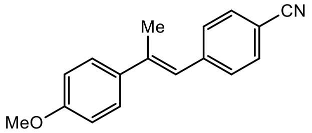


**(*E*)-4-(2-(4-Methoxyphenyl)prop-1-en-1-yl)benzonitrile (1v)**

Prepared according to procedure **2.2**. Flash column chromatography on silica gel (eluent: PE/EA = 30:1 to 15:1, v/v) to afford **1v** as a white solid (117.2 mg, 47%, 1 mmol scale). $R_f$ = 0.4 (PE/EA = 10:1, v/v). $^1$H NMR (600 MHz, $CDCl_3$) δ 7.37 (d, *J* = 8.5 Hz, 2H), 7.08 – 7.05 (m, 2H), 7.03 (d, *J* = 8.3 Hz, 2H), 6.84 – 6.81 (m, 2H), 6.41 (d, *J* = 2.0 Hz, 1H), 3.81 (s, 3H), 2.21 (d, *J* = 1.5 Hz, 3H). $^{13}$C NMR (101 MHz, $CDCl_3$) δ 159.25, 142.94, 142.62, 133.31, 131.82, 129.56, 129.32, 124.78, 119.32, 114.27, 109.31, 55.38, 27.30. HR-ESI-MS m/z calcd for $C_{17}H_{15}NONa$ [(M+Na)$^+$]: 272.1051, found: 272.1046.

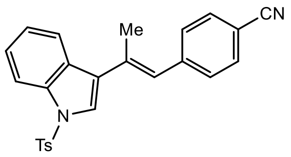


**(*E*)-4-(2-(1-Tosyl-1*H*-indol-3-yl)prop-1-en-1-yl)benzonitrile (1w)**

Prepared according to procedure **2.2**. Flash column chromatography on silica gel (eluent: PE/EA = 20:1 to 10:1, v/v) to afford **1w** as a white solid (147.2 mg, 36%, 1 mmol scale). $R_f$ = 0.6 (PE/EA = 10:1, v/v). $^1$H NMR (400 MHz, $CDCl_3$) δ 8.01 (d, *J* = 8.4 Hz, 1H), 7.70 (d, *J* = 8.4 Hz, 2H), 7.36 (s, 1H), 7.30 (dt, *J* = 8.4, 4.4 Hz, 1H), 7.26 (s, 1H), 7.24 (s, 1H), 7.14 (d, *J* = 8.5 Hz, 2H), 7.12 – 7.06 (m, 2H), 6.64 (d, *J* = 1.7 Hz, 1H), 2.41 (s, 3H), 2.26 (d, *J* = 1.6 Hz, 3H). $^{13}$C NMR (101 MHz, $CDCl_3$) δ 145.37, 142.24, 135.43, 135.41, 133.56, 131.76, 130.06, 129.04, 128.81, 127.79, 126.96, 125.20, 123.99, 123.52, 123.29, 120.91, 119.14, 114.05, 109.59, 27.08, 21.77. HR-ESI-MS m/z calcd for $C_{25}H_{20}N_2O_2SNa$ [$(M+Na)^+$]: 435.1143, found: 435.1141.

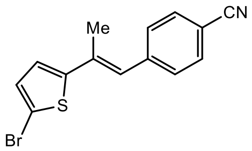


**(*E*)-4-(2-(5-Bromothiophen-2-yl)prop-1-en-1-yl)benzonitrile (1x)**

Prepared according to procedure **2.2**. Flash column chromatography on silica gel (eluent: PE/EA = 50:1, v/v) to afford **1x** as a yellow solid (55.8 mg, 37%, 1 mmol scale). $R_f$ = 0.2 (PE/EA = 50:1, v/v). $^1$H NMR (400 MHz, $CDCl_3$) δ 7.64 (d, *J* = 8.4 Hz, 2H), 7.41 (d, *J* = 8.2 Hz, 2H), 6.99 (d, *J* = 3.9 Hz, 1H), 6.92 (d, *J* = 3.9 Hz, 1H), 6.83 (s, 1H), 2.25 (s, 3H). $^{13}$C NMR (101 MHz, $CDCl_3$) δ 148.37, 142.04, 133.50, 132.20, 130.74, 129.86, 124.74, 124.68, 119.07, 112.17, 110.34, 17.11. HR-ESI-MS m/z calcd for $C_{14}H_{10}NSNaBr$ [$(M+Na)^+$]: 325.9615, found: 325.9610.

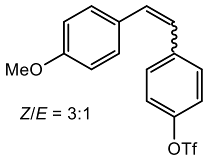



**4-(4-Methoxystyryl)phenyl trifluoromethanesulfonate (1af)**

Prepared according to procedure **2.1.1**. Flash column chromatography on silica gel (eluent: PE/EA = 50:1 to 20:1, v/v) to afford **1af** as a colorless oil (255.0 mg, 71%, 1 mmol scale). $R_f$ = 0.6 (PE/EA = 20:1, v/v). $^1$H NMR (400 MHz, $CDCl_3$) δ 7.54 (d, *J* = 8.5 Hz, 0.7H), 7.46 (d, *J* = 8.5 Hz, 0.7H), 7.33 (d, *J* = 8.4 Hz, 2H), 7.25 (d, *J* = 8.2 Hz, 0.4H), 7.17 – 7.11 (m, 4H), 7.07 (d, *J* = 16.3 Hz, 0.4H), 6.99 – 6.89 (m, 2H), 6.78 (d, *J* = 8.4 Hz, 1H), 6.63 (d, *J* = 12.1 Hz, 1H), 6.46 (d, *J* = 12.1 Hz, 1H), 3.84 (s, 1H), 3.80 (s, 3H). $^{13}$C NMR (101 MHz, $CDCl_3$) δ 159.90, 159.17, 148.45, 148.24, 138.31, 138.22, 131.72, 130.73, 130.48, 130.25, 129.54, 128.94, 128.12, 127.79, 126.77, 124.51, 121.68, 121.28, 118.88 (q, *J* = 321.0 Hz), 114.37, 113.94, 55.46, 55.33.$^{19}$F NMR (376 MHz, $CDCl_3$) δ -72.79, -72.87. HR-ESI-MS m/z calcd for $C_{16}H_{14}O_4SF_3$ [$(M+H)^+$]: 359.0565, found: 359.0561.

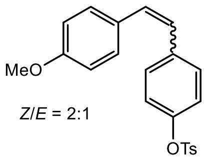



**4-(4-Methoxystyryl)phenyl 4-methylbenzenesulfonate (1ag)**

Prepared according to procedure **2.1.1**. Flash column chromatography on silica gel (eluent: PE/EA = 10:1 to 4:1, v/v) to afford **1ag** as a white solid (236.6 mg, 62%, 1 mmol scale). $R_f$ = 0.4 (PE/EA = 5:1, v/v). $^1$H NMR (400 MHz, $CDCl_3$) δ 7.75 – 7.68 (m, 2 H), 7.42 (d, *J* = 8.8 Hz, 0.7 H), 7.38 (d, *J* = 8.7 Hz, 0.7 H), 7.34 – 7.28 (m, 2 H), 7.16 (d, *J* = 8.3 Hz, 1.4 H), 7.10 (d, *J* = 8.5 Hz, 1.4 H), 7.02 – 6.93 (m, 1H), 6.92 – 6.86 (m, 1H), 6.84 (d, *J* = 8.3 Hz, 1.4 H), 6.74 (d, *J* = 8.7 Hz, 1.4 H), 6.54 (d, *J* = 12.1 Hz, 0.7 H), 6.42 (d, *J* = 12.2 Hz, 0.7 H), 3.82 (s, 1 H), 3.79 (s, 2 H), 2.44 (s, 3 H). $^{13}$C NMR (101 MHz, $CDCl_3$) δ 159.72, 159.02, 148.69, 148.42, 145.45, 136.86, 136.75, 132.62, 130.85, 130.23, 130.14, 129.89, 129.83, 129.46, 129.28, 128.70, 128.68, 127.95, 127.44, 127.32, 125.19, 122.72,

122.32, 114.35, 113.82, 55.47, 55.34, 21.83. HR-ESI-MS m/z calcd for $C_{22}H_{20}O_4SNa$ $[(M+Na)^+]$: 403.0980, found: 403.0984.

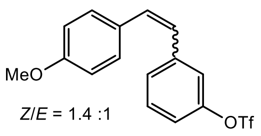


**3-(4-Methoxystyryl)phenyl trifluoromethanesulfonate (1am)**

Prepared according to procedure **2.1.1**. Flash column chromatography on silica gel (eluent: PE/EA = 30:1 to 20:1, v/v) to afford **1am** as a light-yellow oil (207.7 mg, 58%, 1 mmol scale). $R_f$ = 0.5 (PE/EA = 10:1, v/v). $^1$H NMR (400 MHz, $CDCl_3$) δ 7.50 – 7.45 (m, 1.4 H), 7.44 – 7.36 (m, 0.9 H), 7.34 – 7.27 (m, 1 H), 7.19 – 7.13 (m, 2 H), 7.13 – 7.07 (m, 1.4 H), 6.97 – 6.90 (m, 1.2 H), 6.83 – 6.77 (m, 1.4 H), 6.65 (d, *J* = 12.2 Hz, 0.6 H), 6.46 (d, *J* = 12.1 Hz, 0.6 H), 3.85 (s, 1.3 H), 3.80 (s, 1.8 H). $^{13}$C NMR (101 MHz, $CDCl_3$) δ 160.06, 159.34, 150.23, 149.71, 140.76, 140.53, 132.26, 130.97, 130.44, 130.22, 130.11, 129.37, 129.03, 128.71, 128.24, 126.53, 126.19, 124.48, 121.55, 119.66, 119.52, 118.93 (q, *J* = 320.7 Hz, *E*), 118.83 (q, *J* = 320.8 Hz, *Z*), 118.73, 114.42, 114.07, 55.46, 55.35. $^{19}$F NMR (377 MHz, $CDCl_3$) δ -72.89 (*E*), -73.00 (*Z*). HR-ESI-MS m/z calcd for $C_{16}H_{13}F_3O_4SNa$ $[(M+Na)^+]$: 381.0384, found: 381.0380.

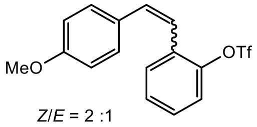


**2-(4-Methoxystyryl)phenyl trifluoromethanesulfonate (1ao)**

Prepared according to procedure **2.1.1**. Flash column chromatography on silica gel (eluent: PE/EA = 30:1 to 20:1, v/v) to afford **1ao** as a light-yellow oil (219.4 mg, 61%, 1 mmol scale). $R_f$ = 0.5 (PE/EA = 10:1, v/v). $^1$H NMR (400 MHz, $CDCl_3$) δ 7.76 (d, *J* = 7.2 Hz, 0.3 H), 7.51 – 7.46 (m, 0.7 H), 7.39 – 7.27 (m, 3 H), 7.22 – 7.09 (m, 3H), 6.96 – 6.91 (m, 0.7 H), 6.79 – 6.72 (m, 2 H), 6.50 (d, *J* = 12.1 Hz, 0.7 H), 3.85 (s, 1H), 3.78 (s, 2H). $^{13}$C NMR (101 MHz, $CDCl_3$) δ 160.19, 159.35, 147.76, 147.09, 133.85, 132.64, 132.03,

131.69, 131.41, 130.41, 129.47, 128.93, 128.65, 128.55, 128.50, 128.37, 128.13, 126.94, 122.01, 121.80, 120.91, 118.81 (q, $J$ = 320.7 Hz), 117.88, 114.47, 113.86, 55.48, 55.31. $^{19}$F NMR (377 MHz, $CDCl_3$) δ -73.63 (*E*), -73.69 (*Z*). HR-ESI-MS m/z calcd for $C_{16}H_{14}O_4F_3S$ [$(M+H)^+$]: 359.0565, found: 359.0569.

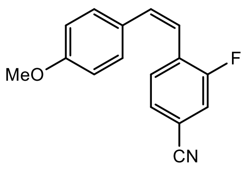


**(*Z*)-3-Fluoro-4-(4-methoxystyryl)benzonitrile (1ap)**

Prepared according to procedure **2.1.1**. Flash column chromatography on silica gel (eluent: PE/EA = 20:1 to 10:1, v/v) to afford **1ap** as a light-yellow oil (154.2 mg, 61%, 1 mmol scale). $R_f$ = 0.45 (PE/EA = 10:1, v/v). $^1$H NMR (400 MHz, $CDCl_3$) δ 7.40 – 7.30 (m, 2H), 7.23 (dd, $J$ = 8.0, 1.6 Hz, 1H), 7.12 (d, $J$ = 8.6 Hz, 2H), 6.84 – 6.74 (m, 3H), 6.46 (d, $J$ = 12.2 Hz, 1H), 3.79 (s, 3H). $^{13}$C NMR (101 MHz, $CDCl_3$) δ 159.84 (d, $J$ = 251.2 Hz), 159.62, 134.78, 131.60 (d, $J$ = 4.2 Hz), 131.21 (d, $J$ = 14.5 Hz), 130.19, 128.45, 127.65 (d, $J$ = 3.8 Hz), 119.54 (d, $J$ = 25.7 Hz), 119.16 (d, $J$ = 2.9 Hz), 117.86 (d, $J$ = 2.8 Hz), 114.06, 111.85 (d, $J$ = 9.4 Hz), 55.35. $^{19}$F NMR (377 MHz, $CDCl_3$) δ -111.79 – -111.83 (m). HR-ESI-MS m/z calcd for $C_{16}H_{13}NOF$ [$(M+H)^+$]: 254.0981, found: 254.0975.

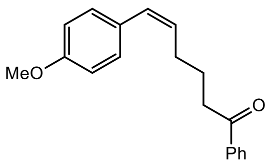


**(*Z*)-6-(4-Methoxyphenyl)-1-phenylhex-5-en-1-one** (**1aw**)

Flash column chromatography (eluent: PE/EA = 50:1 to 20:1, v/v) to afford **1aw** as a light-yellow oil (177.8 mg, 63%, 1 mol scale). $R_f$ = 0.2 (PE/EA = 30:1, v/v). $^1$H NMR (400 MHz, $CDCl_3$) δ 7.93 (dd, $J$ = 8.4, 1.4 Hz, 2H), 7.55 (t, $J$ = 7.4 Hz, 1H), 7.49 – 7.41 (m, 2H), 7.21 (d, $J$ = 8.5 Hz, 2H), 6.86 (d, $J$ = 8.8 Hz, 2H), 6.40 (d, $J$ = 11.6 Hz, 1H), 5.59 (dt, $J$ = 11.6, 7.2 Hz, 1H), 3.81 (s, 3H), 2.99 (t, $J$ = 7.4 Hz, 2H), 2.44 (qd, $J$ = 7.3,

1.8 Hz, 2H), 1.91 (p, *J* = 7.5 Hz, 2H). $^{13}$C NMR (101 MHz, $CDCl_3$) δ 200.32, 158.42, 137.19, 133.06, 130.55, 130.08, 129.30, 128.69, 128.18, 113.75, 55.39, 38.17, 28.22, 24.60. HR-ESI-MS m/z calcd for $C_{19}H_{21}O_2$ [$(M+H)^+$]: 281.1536, found: 281.1535

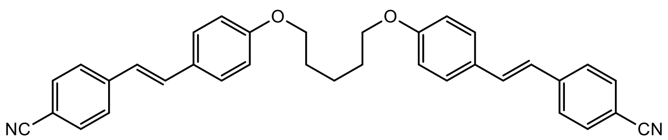


**4,4'-((1*E*,1'*E*)-((Pentane-1,5-diylbis(oxy))bis(4,1-phenylene))bis(ethene-2,1-diyl))dibenzonitrile (1ax)**

Prepared according to procedure **2.2**. Flash column chromatography (eluent: DCM/PE = 1:1 to 2:1 to 1:0, v/v) to afford **1ax** as a white solid (433.2 mg, 85%, 1.0 mmol scale). $R_f$ = 0.4 (DCM/PE = 2:1, v/v). Note: it was extracted with DCM instead of EtOAc after the reaction, since this compound has poor solubility in EtOAc. $^1$H NMR (400 MHz, $CDCl_3$) δ 7.61 (d, *J* = 8.4 Hz, 4H), 7.54 (d, *J* = 8.4 Hz, 4H), 7.46 (d, *J* = 8.7 Hz, 4H), 7.16 (d, *J* = 16.3 Hz, 2H), 6.95 (d, *J* = 16.6 Hz, 2H), 6.91 (d, *J* = 8.7 Hz, 4H), 4.03 (t, *J* = 6.3 Hz, 4H), 1.94 – 1.83 (m, 4H), 1.74 – 1.64 (m, 2H). $^{13}$C NMR (101 MHz, $CDCl_3$) δ 159.74, 142.41, 132.61, 132.15, 129.18, 128.42, 126.70, 124.67, 119.27, 115.02, 110.24, 68.02, 29.14, 22.90. HR-ESI-MS m/z calcd for $C_{35}H_{30}N_2O_2Na$ [$(M+Na)^+$]: 533.2205, found: 533.2206.

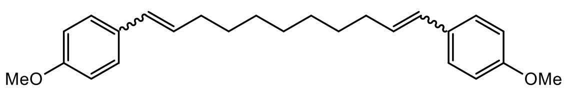


**1,11-Bis(4-methoxyphenyl)undeca-1,10-diene (1ay)**

Prepared according to procedure **2.1.1**. Flash column chromatography (eluent: PE/EA = 50:1 to 40:1, v/v) to afford **1ay** as a white solid (115.1 mg, 16%, 2 mmol scale). $R_f$ = 0.3 (PE/EA = 50:1, v/v). $^1$H NMR (400 MHz, $CDCl_3$) δ 7.49 – 7.40 (m, 0.4 H), 7.31 – 7.27 (m, 2 H), 7.25 – 7.20 (m, 1 H), 7.14 – 7.06 (m, 0.6 H), 6.98 – 6.76 (m, 4.6 H), 6.41 – 6.28 (m, 1.7 H), 6.16 – 6.03 (m, 1 H), 5.64 – 5.52 (m, 0.7 H), 3.84 – 3.79 (m, 6 H), 2.38 – 2.28 (m, 2 H), 2.26 – 2.13 (m, 2 H), 1.54 – 1.23 (m, 10 H). $^{13}$C NMR (101 MHz, $CDCl_3$) δ 158.70, 158.25, 131.80, 130.91, 130.62, 130.06, 129.17, 129.13, 128.21, 127.55, 127.10, 127.08, 126.28, 114.01, 113.65, 113.63, 55.44, 55.40, 55.36, 33.16, 30.16, 29.63, 29.53,

29.47, 29.31, 28.78. HR-ESI-MS m/z calcd for $C_{25}H_{33}O_2$ [(M+H)$^+$]: 365.2481, found: 365.2478.

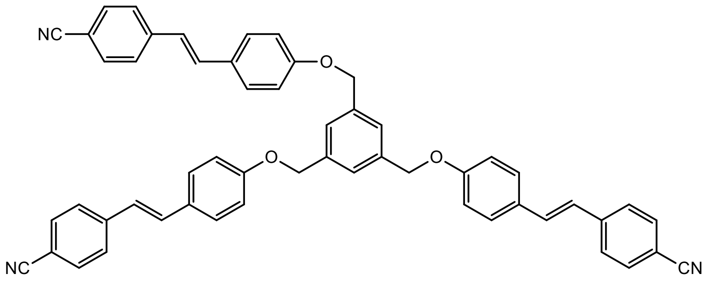


**4,4',4''-((1*E*,1'*E*,1''*E*)-(((Benzene-1,3,5-triyltris(methylene))tris(oxy))tris(benzene-4,1-diyl))tris(ethene-2,1-diyl))tribenzonitrile (1az)**

Prepared according to procedure **2.2**. Flash column chromatography (eluent: DCM/PE = 2:1 to 3:1 to 1:0, v/v) to afford **1az** as a white solid (191.4 mg, 75%, 0.33 mmol). $R_f$ = 0.5 (DCM). $^1$H NMR (400 MHz, $CDCl_3$) δ 7.63 – 7.58 (m, 6H), 7.54 (d, *J* = 8.4 Hz, 6H), 7.50 (s, 3H), 7.47 (d, *J* = 8.6 Hz, 6H), 7.16 (d, *J* = 16.3 Hz, 3H), 6.99 (d, *J* = 8.8 Hz, 6H), 6.95 (d, *J* = 16.6 Hz, 3H), 5.14 (s, 6H). $^{13}$C NMR (101 MHz, $CDCl_3$) δ 159.24, 142.27, 137.94, 132.62, 131.97, 129.72, 128.48, 126.74, 126.16, 125.04, 119.21, 115.40, 110.38, 69.90. HR-ESI-MS m/z calcd for $C_{54}H_{39}N_3O_3Na$ [(M+Na)$^+$]: 800.2899, found: 800.2899.

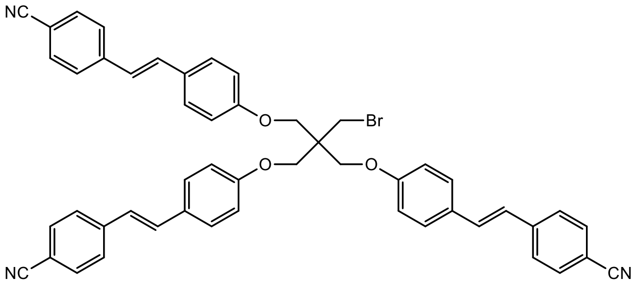


**Alkene 1ba**

Prepared according to procedure **2.2**. Flash column chromatography (eluent: DCM/PE = 5:1 to 2:1 to 1:0, v/v) to afford **1ba** as a white solid (116.9 mg, 48%, 0.3 mmol scale). $R_f$ = 0.4 (DCM). $^1$H NMR (400 MHz, $CDCl_3$) δ 7.61 (d, *J* = 8.1 Hz, 6H), 7.54 (d, *J* = 8.1 Hz, 6H), 7.47 (d, *J* = 8.3 Hz, 6H), 7.16 (d, *J* = 16.3 Hz, 3H), 6.99 – 6.91 (m, 9H), 4.31 (s, 6H), 3.92 (s, 2H). $^{13}$C NMR (101 MHz, $CDCl_3$) δ 159.01, 142.15, 132.61, 131.79, 129.89, 128.41, 126.72, 125.09, 119.25, 115.15, 110.31, 67.08, 44.48, 33.90. HR-ESI-MS m/z

calcd for $C_{50}H_{38}N_3O_3BrNa$ [(M+Na)$^+$]: 830.1994, found: 830.1997.

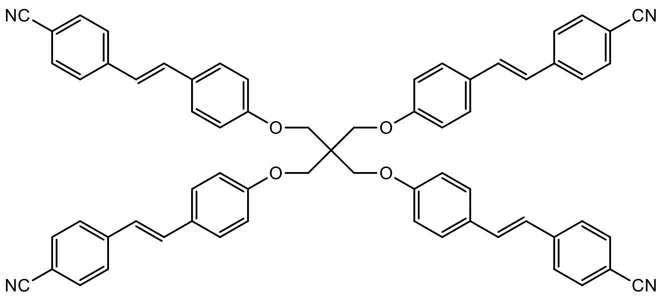


**Alkene 1bb**

Prepared according to procedure **2.2**. Flash column chromatography (eluent: PE/EA = 3:1 to 2:1 to 4:3, v/v) to afford **1bb** as a white solid (145.8 mg, 31%, 0.5 mmol). $R_f$ = 0.4 (DCM). $^1$H NMR (400 MHz, $CDCl_3$) δ 7.61 (d, *J* = 8.4 Hz, 8H), 7.53 (d, *J* = 8.4 Hz, 8H), 7.46 (d, *J* = 8.7 Hz, 8H), 7.15 (d, *J* = 16.3 Hz, 4H), 6.96 (d, *J* = 8.7 Hz, 8H), 6.94 (d, *J* = 16.2 Hz, 4H), 4.42 (s, 8H). $^{13}$C NMR (101 MHz, $CDCl_3$) δ 159.30, 142.19, 132.59, 131.87, 129.82, 128.39, 126.71, 125.06, 119.18, 115.20, 110.38, 66.80, 45.01. HR-ESI-MS m/z calcd for $C_{65}H_{48}N_4O_4Na$ [(M+Na)$^+$]: 971.3573, found: 971.3578.

# 3. Condition Evaluation

## 3.1 Catalyst Evaluation

### Supplementary Table 1. Catalyst evaluation

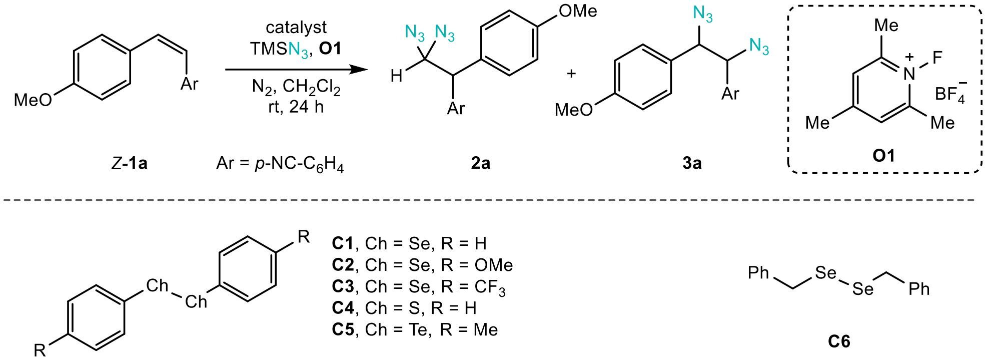


| entry | catalyst | yield [%][a] | |
|---|---|---|---|
| | | **2a** | **3a** |
| 1 | **C1** | 88 | 4 |
| 2 | **C2** | 81 | 11 |
| 3 | **C3** | 39 | 7 |
| 4 | **C4** | 0 | 0 |
| 5 | **C5** | 0 | 9 |
| 6 | **C6** | 8 | 0 |

Reaction conditions: ***Z*-1a** (0.05 mmol), $TMSN_3$ (3.0 equiv), **O1** (2.0 equiv), catalyst (10 mol%), $CH_2Cl_2$ (350 $\mu$L), $N_2$, RT, 24 h. [a]Refers to NMR yield with 1,1,2,2-tetrachloroethane as the internal standard.

## 3.2 Oxidant Evaluation

### Supplementary Table 2. Oxidant evaluation

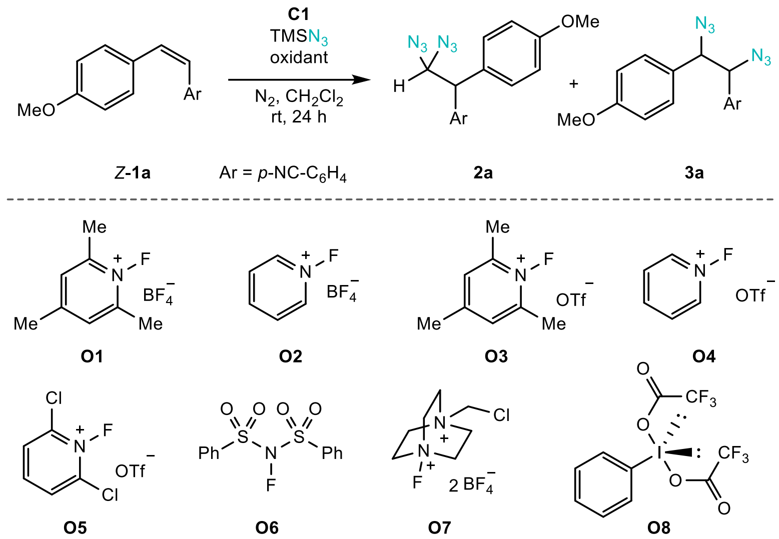


| entry | oxidant | yield [%][a] | |
|---|---|---|---|
| | | **2a** | **3a** |
| 1 | **O1** | 88 | 4 |
| 2 | **O2** | 40 | 9 |
| 3 | **O3** | 0 | 10 |
| 4 | **O4** | 0 | 22 |
| 5 | **O5** | 0 | 0 |
| 6 | **O6** | 0 | 5 |
| 7 | **O7** | 0 | 17 |
| 8 | **O8** | 0 | 55 |

Reaction conditions: *Z*-**1a** (0.05 mmol), $TMSN_3$ (3.0 equiv), oxidant (2.0 equiv), **C1** (10 mol%), $CH_2Cl_2$ (350 $\mu$L), $N_2$, RT, 24 h. [a]Refers to NMR yield with 1,1,2,2-tetrachloroethane as the internal standard.

## 3.3 Amount Evaluation

## Supplementary Table 3. Amount evaluation

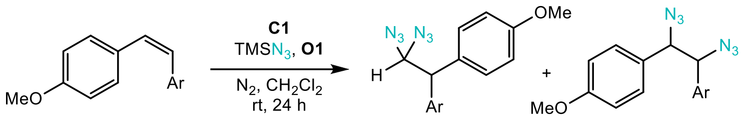


*Z*-**1a** Ar = *p*-NC-$C_6H_4$ **2a** **3a**

| entry | **C1** / mol% | **O1** / equiv | $TMSN_3$ / equiv | yield [%][a] | |
|---|---|---|---|---|---|
| | | | | **2a** | **3a** |
| 1 | 10 | 2.0 | 3.0 | 88 | 4 |
| 2 | 10 | 1.5 | 3.0 | 86 | 8 |
| 3 | 10 | 1.2 | 3.0 | 71 | 8 |
| 4 | 10 | 2.0 | 4.0 | 81 | 9 |
| 5 | 10 | 2.0 | 2.0 | 76 | 10 |
| 6 | 20 | 2.0 | 3.0 | 72 | 15 |
| 7 | 5 | 2.0 | 3.0 | 90 | 5 |
| 8 | 2 | 2.0 | 3.0 | 77 | 18 |
| 9 | 1 | 2.0 | 3.0 | 67 | 28 |
| 10 | --- | 2.0 | 3.0 | 0 | 0 |

Reaction conditions: *Z*-**1a** (0.05 mmol), $TMSN_3$ (*x* equiv), **O1** (*y* equiv), **C1** (*z* mol%), $CH_2Cl_2$ (350 $\mu$L), $N_2$, RT, 24 h. [a]Refers to NMR yield with 1,1,2,2-tetrachloroethane as the internal standard.

### 3.4 Solvent and *Z*/*E* Configuration Evaluation

**Supplementary Table 4. Solvent and *Z*/*E* configuration evaluation**

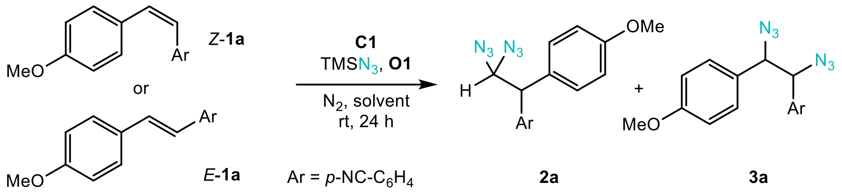


| entry | substrate | solvent | yield [%][a] | |
|---|---|---|---|---|
| | | | **2a** | **3a** |
| 1 | ***Z*-1a** | $CH_2Cl_2$, 350 $\mu$L | 90 | 5 |
| 2 | ***Z*-1a** | $CH_2ClCH_2Cl$, 350 $\mu$L | 80 | 20 |
| 3 | ***Z*-1a** | MeCN, 350 $\mu$L | 89 | 5 |
| 4 | ***Z*-1a** | $CHCl_3$, 350 $\mu$L | no reaction | |
| 5 | ***Z*-1a** | $MeNO_2$, 350 $\mu$L | no reaction | |
| 6 | ***Z*-1a** | Acetone, 350 $\mu$L | no desired products | |
| 7 | ***Z*-1a** | Toluene, 350 $\mu$L | no desired products | |
| 8 | ***Z*-1a** | THF, 350 $\mu$L | no desired products | |
| 9 | ***Z*-1a** | $CH_2Cl_2$, 175 $\mu$L | 96 | 4 |
| 10 | ***Z*-1a** | $CH_2Cl_2$, 700 $\mu$L | 85 | 14 |
| 11 | ***E*-1a** | $CH_2Cl_2$, 350 $\mu$L | 92 | 8 |

Reaction conditions: ***Z*-1a or *E*-1a** (0.05 mmol), $TMSN_3$ (3.0 equiv), **O1** (2.0 equiv), **C1** (5 mol%), solvent (*x* $\mu$L), $N_2$, RT, 24 h. [a]Refers to NMR yield with 1,1,2,2-tetrachloroethane as the internal standard.

## 4. Catalytic Oxidative 1,1-Diazidation of Alkenes

### 4.1 General Procedure

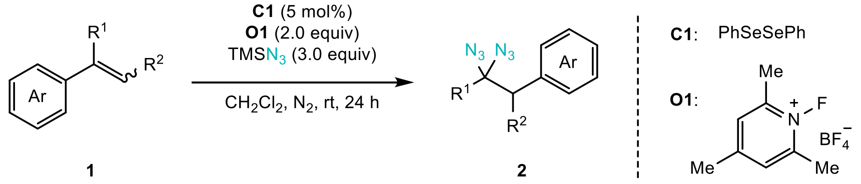


To a dry 4 mL vial equipped with a stir bar were added alkene **1** (0.1 mmol, 1.0 equiv), **O1** (45.4mg 0.2 mmol, 2.0 equiv), and **C1** (1.6 mg, 0.005 mmol, 5 mol%) successively. Then the vial was transferred into glovebox. After dry $CH_2Cl_2$ (350 $\mu$L) and $TMSN_3$

(39.5 μL, 0.3 mmol, 3.0 equiv) were added successively under inert gas, the vial was capped and removed from glovebox. The reaction was performed at room temperature for 24 h. Then the resulting mixture was quenched with diluted hydrochloric acid (1 mL, 1 M) and extracted with $CH_2Cl_2$ (3 mL × 3). The combined organic layers were washed with brine, dried over $Na_2SO_4$, and concentrated under reduced pressure. The residue was purified by preparative thin layer chromatography to give the desired geminal diazides **2**.

### 4.2 Characterization Data of Geminal Diazides

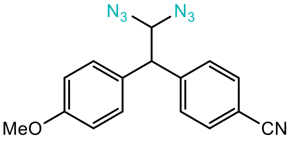


**4-(2,2-Diazido-1-(4-methoxyphenyl)ethyl)benzonitrile (2a)**

Preparative thin layer chromatography twice (eluent: PE/EA = 10:1 to 5:1, v/v) to afford **2a** as a light-yellow oil (30.3 mg, 95%). $R_f$ = 0.3 (PE/EA = 10:1, v/v). $^1$H NMR (400 MHz, $CDCl_3$) δ 7.62 (d, $J$ = 8.4 Hz, 2H), 7.42 (d, $J$ = 8.3 Hz, 2H), 7.18 (d, $J$ = 8.7 Hz, 2H), 6.88 (d, $J$ = 8.7 Hz, 2H), 5.31 (d, $J$ = 8.3 Hz, 1H), 4.16 (d, $J$ = 8.3 Hz, 1H), 3.79 (s, 3H).$^{13}$C NMR (101 MHz, $CDCl_3$) δ 159.44, 144.77, 132.63, 129.68, 129.44, 118.63, 114.63, 111.52, 79.71, 55.42, 55.14. HR-ESI-MS m/z calcd for $C_{16}H_{13}N_7ONa$ [$(M+Na)^+$]: 342.1079, found: 342.1081. IR: 2934(w), 2838(w), 2228(m), 2099(s), 1609(m), 1511(s), 1245(s), 1179(s), 1031(s), 950(m), 817(m), 742(w), 667(m), 577(s), 537(s) cm$^{-1}$.

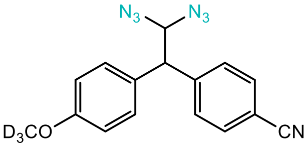


**4-(2,2-Diazido-1-(4-(methoxy-*d*$_3$)phenyl)ethyl)benzonitrile (2b)**

Preparative thin layer chromatography twice (eluent: PE/EA = 10:1, v/v) to afford **2b** as a light-yellow oil (30.6 mg, 95%). $R_f$ = 0.25 (PE/EA = 10:1, v/v). $^1$H NMR (400 MHz, $CDCl_3$) δ 7.62 (d, $J$ = 8.4 Hz, 2H), 7.42 (d, $J$ = 8.3 Hz, 2H), 7.19 (d, $J$ = 8.8 Hz, 2H), 6.88 (d, $J$ = 8.7 Hz, 2H), 5.32 (d, $J$ = 8.4 Hz, 1H), 4.16 (d, $J$ = 8.4 Hz, 1H). $^{13}$C NMR (101 MHz, $CDCl_3$) δ 159.38, 144.76, 132.61, 129.65, 129.40, 118.64, 114.58, 111.45, 79.66, 55.09. HR-ESI-MS m/z calcd for $C_{16}H_{10}D_3N_7ONa$ [$(M+Na)^+$]: 345.1268, found:

345.1268. IR: 3324(w), 3039(w), 2228(m), 2099(s), 1609(m), 1509(s), 1256(s), 1182(s), 1107(s), 993(m), 955(m), 814(s), 759(m), 570(s), 537(m) $cm^{-1}$.

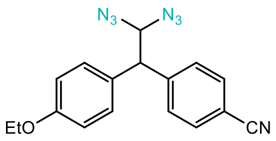


**4-(2,2-Diazido-1-(4-ethoxyphenyl)ethyl)benzonitrile (2c)**

Preparative thin layer chromatography twice (eluent: PE/EA = 10:1, v/v) to afford **2c** as a light-yellow oil (32.3 mg, 96%). $R_f$ = 0.33 (PE/EA = 10:1, v/v). $^1$H NMR (400 MHz, $CDCl_3$) δ 7.62 (d, *J* = 8.4 Hz, 2H), 7.42 (d, *J* = 8.3 Hz, 2H), 7.17 (d, *J* = 8.7 Hz, 2H), 6.87 (d, *J* = 8.8 Hz, 2H), 5.32 (d, *J* = 8.4 Hz, 1H), 4.15 (d, *J* = 8.4 Hz, 1H), 4.01 (q, *J* = 7.0 Hz, 2H), 1.40 (t, *J* = 7.0 Hz, 3H).$^{13}$C NMR (101 MHz, $CDCl_3$) δ 158.80, 144.80, 132.61, 129.64, 129.45, 129.43, 118.65, 115.11, 111.46, 79.70, 63.62, 55.12, 14.89. HR-ESI-MS m/z calcd for $C_{17}H_{15}N_7ONa$ $[(M+Na)^+]$: 356.1236, found: 356.1237. IR: 3322(w), 2981(w), 2929(w), 2228(m), 2099(s), 1610(m), 1511(s), 1392(s), 1304(s), 1237(s), 1179(s), 1117(m), 1045(s), 923(m), 818(s), 743(m), 668(m), 601(m), 556(m), 539(m) $cm^{-1}$.

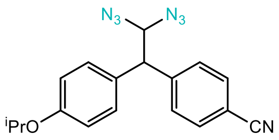


**4-(2,2-Diazido-1-(4-isopropoxyphenyl)ethyl)benzonitrile (2d)**

Preparative thin layer chromatography (eluent: PE/EA = 10:1, v/v) to afford **2d** as a light-yellow oil (34.9 mg, 99%). $R_f$ = 0.33 (PE/EA = 10:1, v/v). $^1$H NMR (400 MHz, $CDCl_3$) δ 7.62 (d, *J* = 8.4 Hz, 2H), 7.42 (d, *J* = 8.3 Hz, 2H), 7.16 (d, *J* = 8.7 Hz, 2H), 6.86 (d, *J* = 8.7 Hz, 2H), 5.32 (d, *J* = 8.4 Hz, 1H), 4.52 (p, *J* = 6.1 Hz, 1H), 4.15 (d, *J* = 8.4 Hz, 1H), 1.32 (d, *J* = 6.0 Hz, 6H).$^{13}$C NMR (101 MHz, $CDCl_3$) δ 157.76, 144.81, 132.59, 129.64, 129.43, 129.29, 118.65, 116.28, 111.42, 79.69, 70.03, 55.12, 22.09. HR-ESI-MS m/z calcd for $C_{18}H_{17}N_7ONa$ $[(M+Na)^+]$: 370.1392, found: 370.1389. IR: 2978(w), 2228(m), 2100(s), 1609(m), 1508(s), 1373(w), 1239(s), 1183(s), 1108(s), 1021(w), 952(s), 814(m), 743(w), 605(m), 556(m), 540(m) $cm^{-1}$.

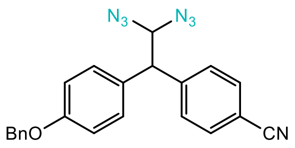


**4-(2,2-Diazido-1-(4-(benzyloxy)phenyl)ethyl)benzonitrile (2e)**

Preparative thin layer chromatography twice (eluent: PE/EA = 10:1, v/v) to afford **2e** as a white solid (27.0 mg, 68%). $R_f$ = 0.25 (PE/EA = 10:1, v/v). $^1$H NMR (400 MHz, $CDCl_3$) δ 7.62 (d, $J$ = 8.4 Hz, 2H), 7.45 – 7.34 (m, 7H), 7.19 (d, $J$ = 8.7 Hz, 2H), 6.96 (d, $J$ = 8.7 Hz, 2H), 5.32 (d, $J$ = 8.3 Hz, 1H), 5.05 (s, 2H), 4.17 (d, $J$ = 8.3 Hz, 1H). $^{13}$C NMR (101 MHz, $CDCl_3$) δ 158.64, 144.70, 136.76, 132.62, 129.93, 129.70, 129.43, 128.76, 128.22, 127.59, 118.63, 115.49, 111.49, 79.66, 70.19, 55.12. HR-ESI-MS m/z calcd for $C_{22}H_{18}N_7O$ $[(M+H)^+]$: 396.1573, found: 396.1572. IR: 3034(w), 2910(w), 2864(w), 2229(m), 2094(s), 1607(m), 1510(s), 1463(w), 1384(m), 1252(s), 1223(s), 1181(s), 1120(m), 1021(s), 951(m), 905(m), 857(m), 818(m), 743(s), 697(m), 622(m), 568(s), 541(s) $cm^{-1}$.

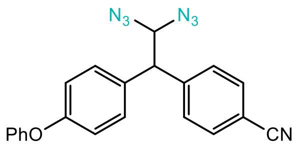


**4-(2,2-Diazido-1-(4-phenoxyphenyl)ethyl)benzonitrile (2f)**

Preparative thin layer chromatography twice (eluent: PE/EA = 10:1, v/v) to afford **2f** as a light-yellow oil (35.7 mg, 94%). $R_f$ = 0.25 (PE/EA = 10:1, v/v). $^1$H NMR (400 MHz, $CDCl_3$) δ 7.64 (d, $J$ = 8.4 Hz, 2H), 7.43 (d, $J$ = 8.4 Hz, 2H), 7.34 (dd, $J$ = 8.6, 7.4 Hz, 2H), 7.22 (d, $J$ = 8.7 Hz, 2H), 7.13 (t, $J$ = 7.4 Hz, 1H), 7.03 – 6.93 (m, 4H), 5.33 (d, $J$ = 8.2 Hz, 1H), 4.20 (d, $J$ = 8.2 Hz, 1H). $^{13}$C NMR (101 MHz, $CDCl_3$) δ 157.48, 156.59, 144.44, 132.68, 132.05, 129.99, 129.96, 129.47, 123.94, 119.48, 119.01, 118.59, 111.65, 79.57, 55.23. HR-ESI-MS m/z calcd for $C_{21}H_{15}N_7ONa$ $[(M+Na)^+]$: 404.1236, found: 404.1231. IR: 3063(w), 2923(w), 2442(w), 2229(m), 2100(s), 1588(m), 1505(s), 1487(s), 1415(w), 1232(s), 1170(m), 1114(m), 1071(m), 1019(m), 952(m), 871(m), 830(m), 750(s), 692(s), 582(s), 552(s), 529(s) $cm^{-1}$.

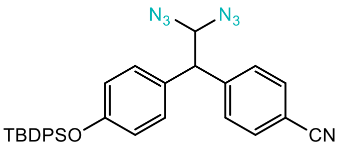


**4-(2,2-Diazido-1-(4-((*tert*-butyldiphenylsilyl)oxy)phenyl)ethyl)benzonitrile (2g)**

Preparative thin layer chromatography (eluent: PE/EA = 10:1, v/v) to afford **2g** as a light-yellow oil (46.6 mg, 85%). $R_f$ = 0.45 (PE/EA = 10:1, v/v). $^1$H NMR (400 MHz, $CDCl_3$) δ 7.71 – 7.67 (m, 4H), 7.59 (d, *J* = 8.3 Hz, 2H), 7.42 (d, *J* = 7.5 Hz, 2H), 7.39 – 7.31 (m, 6H), 6.98 (d, *J* = 8.6 Hz, 2H), 6.74 (d, *J* = 8.6 Hz, 2H), 5.22 (d, *J* = 8.1 Hz, 1H), 4.09 (d, *J* = 8.0 Hz, 1H), 1.09 (s, 9H). $^{13}$C NMR (101 MHz, $CDCl_3$) δ 155.64, 144.69, 135.64, 132.78, 132.53, 130.12, 129.98, 129.54, 129.51, 127.94, 120.44, 118.64, 111.49, 79.70, 55.25, 26.60, 19.57. HR-ESI-MS m/z calcd for $C_{31}H_{29}N_7ONaSi$ [$(M+Na)^+$]: 566.2101, found: 566.2108. IR: 3071(w), 2932(w), 2858(w),2229(m), 2102(s), 1607(m), 1508(s), 1427(m), 1255(s), 1177(m), 1109(s), 1013(w), 913(s), 822(s), 736(m), 700(s), 611(m), 565(m), 501(s) cm$^{-1}$.

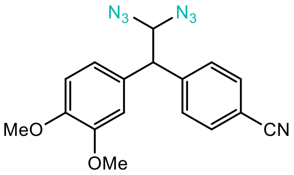


**4-(2,2-Diazido-1-(3,4-dimethoxyphenyl)ethyl)benzonitrile (2h)**

Preparative thin layer chromatography (eluent: PE/EA = 3:1, v/v) to afford **2h** as a light-yellow oil (33.4 mg, 97%). $R_f$ = 0.50 (PE/EA = 3:1, v/v). $^1$H NMR (400 MHz, $CDCl_3$) δ 7.63 (d, *J* = 8.4 Hz, 2H), 7.43 (d, *J* = 8.3 Hz, 2H), 6.89 – 6.79 (m, 2H), 6.74 (s, 1H), 5.32 (d, *J* = 8.3 Hz, 1H), 4.15 (d, *J* = 8.3 Hz, 1H), 3.86 (s, 3H), 3.85 (s, 3H).$^{13}$C NMR (101 MHz, $CDCl_3$) δ 149.43, 149.02, 144.61, 132.63, 130.05, 129.43, 120.69, 118.62, 112.01, 111.59, 79.66, 56.15, 56.03, 55.44. HR-ESI-MS m/z calcd for $C_{17}H_{15}N_7O_2Na$ [$(M+Na)^+$]: 372.1185, found: 372.1181. IR: 2936(w), 2837(w), 2228(m), 2100(s), 1606(m), 1592(m), 1514(s), 1463(m), 1418(m), 1338(w), 1234(s), 1144(s) 1023(s), 966(m), 837(m), 808(m), 751(m), 699(w), 596(m), 555(m) cm$^{-1}$.

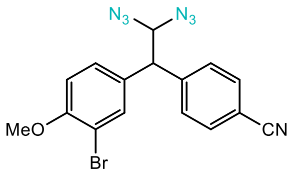


**4-(2,2-Diazido-1-(3-bromo-4-methoxyphenyl)ethyl)benzonitrile (2i)**

Preparative thin layer chromatography (eluent: PE/EA = 5:1, v/v) to afford **2i** as a light-yellow oil (38.7 mg, 97%). $R_f$ = 0.40 (PE/EA = 5:1, v/v). $^1$H NMR (400 MHz, $CDCl_3$) δ 7.64 (d, *J* = 8.4 Hz, 2H), 7.44 (d, *J* = 2.3 Hz, 1H), 7.40 (d, *J* = 8.3 Hz, 2H), 7.19 (dd, *J* = 8.5, 2.3 Hz, 1H), 6.87 (d, *J* = 8.5 Hz, 1H), 5.31 (d, *J* = 8.2 Hz, 1H), 4.13 (d, *J* = 8.2 Hz, 1H), 3.88 (s, 3H). $^{13}$C NMR (101 MHz, $CDCl_3$) δ 155.77, 144.02, 133.39, 132.74, 131.08, 129.43, 128.72, 118.52, 112.35, 112.25, 111.78, 79.37, 56.41, 54.67. HR-ESI-MS m/z calcd for $C_{16}H_{13}N_7O_{Br}$ [(M+H)$^+$]: 398.0365, found: 398.0351. IR: 3325(w), 2943(w), 2840(w), 2466(w), 2228(m), 2098(s), 1924(w), 1798(w), 1694(w), 1604(m), 1496(s), 1461(m), 1440(m), 1410(m), 1255(s), 1053(s), 1018(s), 952(m), 829(m), 812(m), 737(m), 671(m), 598(m), 555(s) cm$^{-1}$.

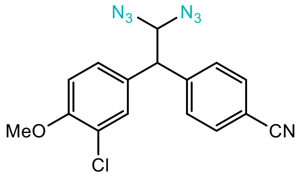


**4-(2,2-Diazido-1-(3-chloro-4-methoxyphenyl)ethyl)benzonitrile (2j)**

Preparative thin layer chromatography (eluent: PE/EA = 5:1, v/v) to afford **2j** as a light-yellow oil (33.1 mg, 94%). $R_f$ = 0.40 (PE/EA = 5:1, v/v). $^1$H NMR (400 MHz, $CDCl_3$) δ 7.64 (d, *J* = 8.4 Hz, 2H), 7.41 (d, *J* = 8.3 Hz, 2H), 7.27 (d, *J* = 2.3 Hz, 1H), 7.14 (dd, *J* = 8.5, 2.3 Hz, 1H), 6.91 (d, *J* = 8.5 Hz, 1H), 5.31 (d, *J* = 8.1 Hz, 1H), 4.13 (d, *J* = 8.1 Hz, 1H), 3.89 (s, 3H). $^{13}$C NMR (101 MHz, $CDCl_3$) δ 154.89, 144.02, 132.75, 130.62, 130.36, 129.43, 123.17, 118.52, 112.44, 111.80, 79.38, 77.48, 77.16, 76.84, 56.32, 54.76.HR-ESI-MS m/z calcd for $C_{16}H_{12}N_7ONaCl$ [(M+Na)$^+$]: 376.0690, found: 376.0679. IR: 2931(w), 2840(w), 2229(m), 2097(s), 1604(m), 1502(s), 1459(m), 1442(m), 1286(s), 1256(s), 1964(s), 1018(s), 953(m), 833(m), 810(m), 737(m), 687(s), 555(s), 432(m) cm$^{-1}$.

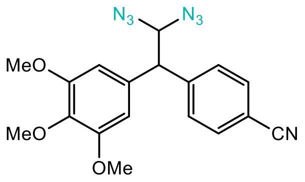


**4-(2,2-Diazido-1-(3,4,5-trimethoxyphenyl)ethyl)benzonitrile (2k)**

Preparative thin layer chromatography (eluent: PE/EA = 3:1, v/v) to afford **2k** as a light-yellow oil (31.4 mg, 83%). $R_f$ = 0.50 (PE/EA = 3:1, v/v). $^1$H NMR (400 MHz, $CDCl_3$) δ 7.64 (d, *J* = 8.4 Hz, 2H), 7.44 (d, *J* = 8.4 Hz, 2H), 6.46 (s, 2H), 5.34 (d, *J* = 8.2 Hz, 1H), 4.13 (d, *J* = 8.2 Hz, 1H), 3.83 (s, 6H), 3.82 (s, 3H). $^{13}$C NMR (101 MHz, $CDCl_3$) δ 153.70, 144.21, 138.02, 133.12, 132.62, 132.59, 129.41, 118.57, 111.65, 105.84, 79.47, 60.97, 56.38, 55.94. HR-ESI-MS m/z calcd for $C_{18}H_{17}N_7O_3Na$ [(M+Na)$^+$]: 402.1291, found: 402.1295. IR: 2939(w), 2840(w), 2228(m), 2100(s), 1590(s), 1505(s), 1459(s), 1423(s), 1327(s), 1231(s), 1123(s), 1001(m), 938(w), 833(m), 754(m), 664(m), 556(m) cm$^{-1}$.

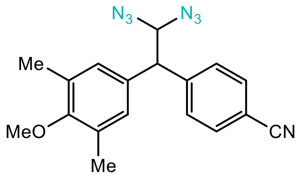


**4-(2,2-Diazido-1-(4-methoxy-3,5-dimethylphenyl)ethyl)benzonitrile (2l)**

Preparative thin layer chromatography twice (eluent: PE/EA = 10:1, v/v) to afford **2l** as a light-yellow oil (34.3 mg, 99%). $R_f$ = 0.35 (PE/EA = 10:1, v/v). $^1$H NMR (400 MHz, $CDCl_3$) δ 7.63 (d, *J* = 8.1 Hz, 2H), 7.42 (d, *J* = 8.3 Hz, 2H), 6.89 (s, 2H), 5.33 (d, *J* = 8.7 Hz, 1H), 4.08 (d, *J* = 8.7 Hz, 1H), 3.70 (s, 3H), 2.26 (s, 6H). $^{13}$C NMR (101 MHz, $CDCl_3$) δ 156.83, 144.72, 132.89, 132.63, 131.80, 129.39, 128.71, 118.67, 111.46, 79.61, 59.79, 55.37, 16.38. HR-ESI-MS m/z calcd for $C_{18}H_{17}N_7ONa$ [(M+Na)$^+$]: 370.1392, found: 370.1395. IR: 3321(w), 2927(w), 2455(w), 2228(m), 2099(s), 1923(w), 1607(m), 1484(m), 1417(m), 1302(m), 1217(s), 1144(s), 1077(w), 1006(s), 935(m), 836(m), 763(m), 641(m), 555(s) cm$^{-1}$.

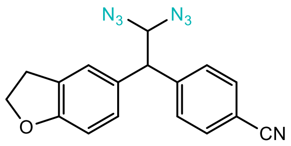


**4-(2,2-Diazido-1-(2,3-dihydrobenzofuran-5-yl)ethyl)benzonitrile (2m)**

Preparative thin layer chromatography twice (eluent: PE/EA = 10:1, v/v) to afford **2m** as a light-yellow oil (30.4 mg, 92%). $R_f$ = 0.25 (PE/EA = 10:1, v/v). $^1$H NMR (400 MHz, $CDCl_3$) δ 7.62 (d, *J* = 8.4 Hz, 2H), 7.42 (d, *J* = 8.3 Hz, 2H), 7.08 (d, *J* = 1.9 Hz, 1H), 7.01 (dd, *J* = 8.2, 2.1 Hz, 1H), 6.75 (d, *J* = 8.2 Hz, 1H), 5.31 (d, *J* = 8.4 Hz, 1H), 4.56 (t, *J* = 8.7 Hz, 2H), 4.14 (d, *J* = 8.4 Hz, 1H), 3.19 (t, *J* = 8.7 Hz, 2H). $^{13}$C NMR (101 MHz, $CDCl_3$) δ 160.09, 144.97, 132.61, 129.60, 129.37, 128.40, 128.23, 125.10, 118.62, 111.45, 109.82, 79.77, 71.56, 55.39, 29.76. HR-ESI-MS m/z calcd for $C_{17}H_{13}N_7ONa$ [(M+Na)$^+$]: 354.1079, found: 354.1073. IR: 2962(w), 2897(w), 2856(w), 2228(m), 2098(s), 1923(w), 1609(m), 1491(s), 1441(m), 1414(m), 1315(m), 1229(s), 1104(m), 1067(m), 981(m), 944(m), 816(m), 740(m), 665(m), 616(m), 559(s), 492(m) cm$^{-1}$.

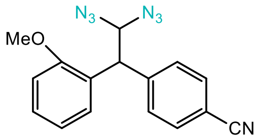


**4-(2,2-Diazido-1-(2-methoxyphenyl)ethyl)benzonitrile (2n)**

Preparative thin layer chromatography (eluent: PE/EA = 10:1, v/v) to afford **2n** as a light-yellow oil (25.8 mg, 81%). $R_f$ = 0.45 (PE/EA = 10:1, v/v). $^1$H NMR (400 MHz, $CDCl_3$) δ 7.59 (d, *J* = 8.3 Hz, 2H), 7.46 (d, *J* = 8.1 Hz, 2H), 7.32 – 7.27 (m, 1H), 7.23 (dd, *J* = 7.6, 1.6 Hz, 1H), 6.97 (t, *J* = 7.5 Hz, 1H), 6.90 (d, *J* = 8.1 Hz, 1H), 5.56 (d, *J* = 9.2 Hz, 1H), 4.56 (d, *J* = 9.2 Hz, 1H), 3.84 (s, 3H). $^{13}$C NMR (101 MHz, $CDCl_3$) δ 156.87, 144.61, 132.40, 129.71, 129.41, 129.20, 126.37, 121.19, 118.81, 111.45, 111.25, 79.19, 55.63, 50.45. HR-ESI-MS m/z calcd for $C_{16}H_{13}N_7ONa$ [(M+Na)$^+$]: 342.1079, found: 342.1074. IR: 2939(w), 2840(w), 2228(m), 2099(s), 1604(m), 1493(s), 1462(m), 1244(s), 1122(m), 1024(s), 950(m), 819(m), 754(s), 674(w), 617(m), 555(m), 500(w) cm$^{-1}$.

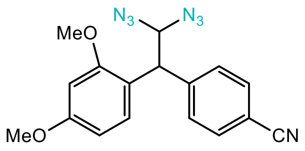


**4-(2,2-Diazido-1-(2,4-dimethoxyphenyl)ethyl)benzonitrile (2o)**

Preparative thin layer chromatography (eluent: PE/EA = 3:1, v/v) to afford **2o** as a light-yellow oil (23.4 mg, 67%). $R_f$ = 0.50 (PE/EA = 3:1, v/v). $^1$H NMR (400 MHz, $CDCl_3$) δ 7.59 (d, *J* = 8.3 Hz, 2H), 7.45 (d, *J* = 8.4 Hz, 2H), 7.12 (d, *J* = 8.3 Hz, 1H), 6.52 – 6.44 (m, 2H), 5.52 (d, *J* = 9.1 Hz, 1H), 4.48 (d, *J* = 9.1 Hz, 1H), 3.81 (s, 3H), 3.79 (s, 3H). $^{13}$C NMR (101 MHz, $CDCl_3$) δ 160.73, 157.94, 145.01, 132.35, 129.82, 129.60, 118.84, 118.82, 111.08, 104.87, 99.29, 79.23, 55.63, 55.49, 49.98. HR-ESI-MS m/z calcd for $C_{17}H_{15}N_7O_2Na$ [(M+Na)$^+$]: 372.1185, found: 372.1177. IR: 3004(w), 2938(w), 2838(w), 2457(w), 2228(m), 2099(s), 1609(s), 1585(s), 1505(s), 1461(s), 1417(m), 1338(w), 1294(m), 1238(m), 1207(s), 1159(m), 1130(m), 1030(m), 922(m), 825(m), 798(m), 745(w), 666(w), 575(m), 557(m), 502(m) cm$^{-1}$.

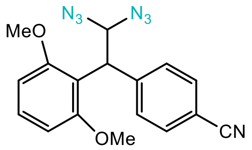


**4-(2,2-Diazido-1-(2,6-dimethoxyphenyl)ethyl)benzonitrile (2p)**

Preparative thin layer chromatography (eluent: PE/EA = 3:1, v/v) to afford **2p** as a light-yellow oil (27.8 mg, 80%). $R_f$ = 0.40 (PE/EA = 3:1, v/v). $^1$H NMR (400 MHz, $CDCl_3$) δ 7.59 – 7.49 (m, 4H), 7.23 (t, *J* = 8.4 Hz, 1H), 6.58 (d, *J* = 8.4 Hz, 2H), 5.96 (d, *J* = 10.8 Hz, 1H), 4.84 (d, *J* = 10.8 Hz, 1H), 3.86 (s, 3H). $^{13}$C NMR (101 MHz, $CDCl_3$) δ 158.07, 145.34, 132.15, 129.56, 119.00, 115.00, 110.77, 104.61, 79.20, 55.95, 46.23. HR-ESI-MS m/z calcd for $C_{17}H_{15}N_7O_2Na$ [(M+Na)$^+$]: 372.1185, found: 372.1184. IR: 2939(w), 2840(w), 2227(m), 2095(s), 1922(w), 1592(s), 1504(w), 1473(s), 1436(m), 1340(w), 1239(s), 1104(s), 1034(m), 947(m), 853(m), 812(m), 782(m), 584(m), 554(m) cm$^{-1}$.

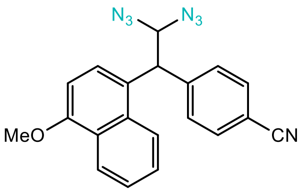


**4-(2,2-Diazido-1-(4-methoxynaphthalen-1-yl)ethyl)benzonitrile (2q)**

Preparative thin layer chromatography twice (eluent: PE/EA = 10:1, v/v) to afford **2q** as a light-yellow oil (30.2 mg, 82%). $R_f$ = 0.33 (PE/EA = 10:1, v/v). $^1$H NMR (400 MHz, $CDCl_3$) δ 8.33 (dd, *J* = 7.6, 2.1 Hz, 1H), 7.85 (d, *J* = 7.7 Hz, 1H), 7.59 (d, *J* = 8.2 Hz, 2H), 7.55 – 7.38 (m, 5H), 6.85 (d, *J* = 8.1 Hz, 1H), 5.51 (d, *J* = 7.9 Hz, 1H), 4.94 (d, *J* = 7.9 Hz, 1H), 4.02 (s, 3H). $^{13}$C NMR (101 MHz, $CDCl_3$) δ 155.63, 144.66, 132.51, 132.33, 129.87, 127.51, 126.38, 125.67, 125.52, 125.38, 123.21, 122.42, 118.67, 111.56, 103.11, 80.01, 55.71, 50.34. HR-ESI-MS m/z calcd for $C_{20}H_{15}N_7ONa$ [$(M+Na)^+$]: 392.1236, found: 392.1233. IR: 3074(w), 2937(w), 2845(w), 2228(m), 2101(s), 1928(w), 1585(m), 1514(m), 1462(m), 1426(w), 1387(m), 1336(w), 1258(s), 1217(s), 1159(m), 1093(s), 1034(m), 948(m), 896(w), 803(m), 761(s), 711(w), 602(m), 557(m) cm$^{-1}$.

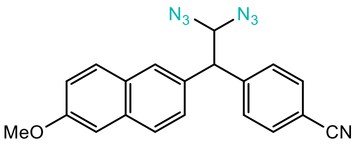


**4-(2,2-Diazido-1-(6-methoxynaphthalen-2-yl)ethyl)benzonitrile (2r)**

Preparative thin layer chromatography twice (eluent: PE/EA = 8:1, v/v) to afford **2r** as a light-yellow oil (31.5 mg, 85%). $R_f$ = 0.33 (PE/EA = 8:1, v/v). $^1$H NMR (400 MHz, $CDCl_3$) δ 7.75 – 7.67 (m, 3H), 7.63 (d, *J* = 8.0 Hz, 2H), 7.48 (d, *J* = 8.1 Hz, 2H), 7.30 (dd, *J* = 8.6, 1.9 Hz, 1H), 7.18 (dd, *J* = 8.9, 2.5 Hz, 1H), 7.11 (d, *J* = 2.5 Hz, 1H), 5.46 (d, *J* = 8.4 Hz, 1H), 4.34 (d, *J* = 8.5 Hz, 1H), 3.91 (s, 3H). $^{13}$C NMR (101 MHz, $CDCl_3$) δ 158.31, 144.53, 134.11, 132.72, 132.64, 129.60, 129.51, 128.90, 127.90, 127.44, 126.53, 119.75, 118.63, 111.58, 105.72, 79.57, 55.78, 55.49. HR-ESI-MS m/z calcd for $C_{20}H_{15}N_7ONa$ [$(M+Na)^+$]: 392.1236, found: 392.1233. IR: 3059(w), 2936(w), 2843(w), 2228(m), 2099(s), 1919(w), 1718(w), 1632(m), 1604(s), 1504(m), 1483(m), 1392(m), 1346(w), 1263(m), 1212(s), 1162(m), 1121(m), 1028(m), 948(m), 902(w), 848(m), 818(m), 764(m), 665(m), 554(s), 474(s) cm$^{-1}$.

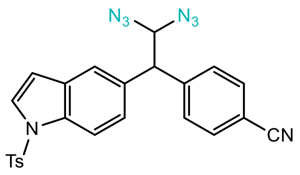


**4-(2,2-Diazido-1-(1-tosyl-1*H*-indol-5-yl)ethyl)benzonitrile (2s)**

Preparative thin layer chromatography (eluent: PE/DCM = 1:2, v/v) to afford **2s** as a light-yellow oil (31.9 mg, 66%). $R_f$ = 0.45 (PE/DCM = 1:2, v/v). $^1$H NMR (400 MHz, $CDCl_3$) δ 7.96 (d, *J* = 8.6 Hz, 1H), 7.76 (d, *J* = 8.4 Hz, 2H), 7.61 (d, *J* = 8.3 Hz, 2H), 7.57 (d, *J* = 3.7 Hz, 1H), 7.53 – 7.38 (m, 3H), 7.22 (d, *J* = 8.0 Hz, 2H), 7.19 (dd, *J* = 8.6, 1.8 Hz, 1H), 6.62 (d, *J* = 3.7 Hz, 1H), 5.38 (d, *J* = 8.4 Hz, 1H), 4.27 (d, *J* = 8.4 Hz, 1H), 2.34 (s, 3H). $^{13}$C NMR (101 MHz, $CDCl_3$) δ 145.33, 144.53, 135.32, 134.32, 132.75, 132.63, 131.28, 130.10, 129.50, 127.37, 126.97, 124.95, 121.35, 118.57, 114.19, 111.57, 108.84, 79.64, 55.70, 21.70. HR-ESI-MS m/z calcd for $C_{24}H_{18}N_8O_2NaS$ [(M+Na)$^+$]: 505.1171, found: 505.1172. IR: 2966(w), 2925(w), 2228(m), 2101(s), 1597(m), 1493(w), 1459(m), 1414(w), 1368(s), 1240(m), 1170(s), 1127(s), 1091(m), 994(m), 811(m), 726(m), 702(m), 674(s), 579(s), 553(m), 538(s) cm$^{-1}$.

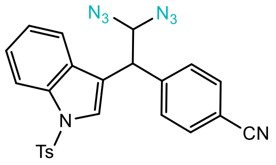


**4-(2,2-Diazido-1-(1-tosyl-1*H*-indol-3-yl)ethyl)benzonitrile (2t)**

Preparative thin layer chromatography (eluent: PE/EA = 3:1, v/v) to afford **2t** as a light-yellow oil (40.7 mg, 84%). $R_f$ = 0.6 (PE/EA = 3:1, v/v). $^1$H NMR (400 MHz, $CDCl_3$) δ 7.98 (d, *J* = 8.4 Hz, 1H), 7.79 (d, *J* = 8.4 Hz, 2H), 7.75 (s, 1H), 7.59 (d, *J* = 8.3 Hz, 2H), 7.41 (d, *J* = 8.3 Hz, 2H), 7.31 (ddd, *J* = 8.4, 5.9, 2.5 Hz, 1H), 7.25 (d, *J* = 9.3 Hz, 2H), 7.18 – 7.12 (m, 2H), 5.36 (d, *J* = 6.4 Hz, 1H), 4.44 (d, *J* = 6.4 Hz, 1H), 2.36 (s, 3H). $^{13}$C NMR (101 MHz, $CDCl_3$) δ 145.45, 142.46, 135.13, 135.08, 132.59, 130.10, 129.96, 129.69, 126.98, 125.52, 124.54, 123.67, 119.37, 118.66, 118.46, 114.06, 112.19, 79.12, 47.62, 21.71. HR-ESI-MS m/z calcd for $C_{24}H_{18}N_8O_2SNa$ [(M+Na)$^+$]: 505.1171, found: 505.1177. IR: 3099(w), 2920(w), 2851(w), 2228(m), 2104(s), 1920(w), 1718(w), 1598(m), 1502(w), 1447(m), 1366(m), 1240(m), 1212(m), 1171(s), 1121(s), 1089(m),

1020(m), 960(m), 811(m), 745(s), 703(m), 673(s), 572(s), 535(s), 493(w) $cm^{-1}$.

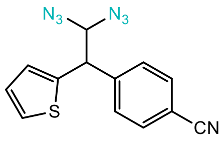


**4-(2,2-Diazido-1-(thiophen-2-yl)ethyl)benzonitrile (2u)**

Preparative thin layer chromatography twice (eluent: PE/EA = 10:1, v/v) to afford **2u** as a light-yellow oil (24.6 mg, 83%). $R_f$ = 0.30 (PE/EA = 10:1, v/v). $^1$H NMR (400 MHz, $CDCl_3$) δ 7.66 (d, *J* = 8.4 Hz, 2H), 7.50 (d, *J* = 8.4 Hz, 2H), 7.30 (dd, *J* = 4.6, 1.8 Hz, 1H), 7.04 – 6.97 (m, 2H), 5.28 (d, *J* = 7.1 Hz, 1H), 4.49 (d, *J* = 7.1 Hz, 1H). $^{13}$C NMR (101 MHz, $CDCl_3$) δ 143.53, 139.69, 132.66, 129.69, 127.24, 127.20, 126.16, 118.53, 112.08, 79.94, 51.70. HR-ESI-MS m/z calcd for $C_{13}H_9N_7NaS$ [$(M+Na)^+$]: 318.0538, found: 318.0545. IR: 3324(w), 3073(w), 2924(w), 2461(w), 2229(m), 2100(s), 1923(w), 1801(w), 1719(w), 1608(m), 1504(m), 1415(m), 1321(m), 1233(s), 1117(m), 1021(m), 947(m), 902(m), 835(m), 790(w), 701(s), 619(m), 553(s), 516(m) $cm^{-1}$.

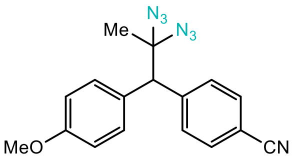


**4-(2,2-Diazido-1-(4-methoxyphenyl)propyl)benzonitrile (2v)**

Preparative thin layer chromatography (eluent: PE/EA = 8:1, v/v) to afford **2v** as a light-yellow oil (20.0 mg, 60%). $R_f$ = 0.3 (PE/EA = 10:1, v/v).$^1$H NMR (400 MHz, $CDCl_3$) δ 7.61 (q, *J* = 8.4 Hz, 4H), 7.36 (d, *J* = 8.6 Hz, 2H), 6.87 (d, *J* = 8.6 Hz, 2H), 4.10 (s, 1H), 3.79 (s, 3H), 1.61 (s, 3H). $^{13}$C NMR (101 MHz, $CDCl_3$) δ 159.42, 144.76, 132.27, 130.85, 130.54, 129.64, 118.78, 114.27, 111.36, 83.03, 60.72, 55.39, 24.28. HR-ESI-MS m/z calcd for $C_{17}H_{15}N_7ONa$ [$(M+Na)^+$]: 356.1236, found: 356.1231. IR: 3330(w), 2933(w), 2838(w), 2468(w), 2228(m), 2102(s), 1609(m), 1511(s), 1461(m), 1381(m), 1247(s), 1180(s), 1114(m), 1088(m), 1032(s), 818(s), 745(m), 690(m), 579(s), 540(m) $cm^{-1}$.

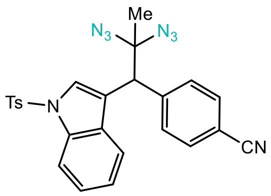


**4-(2,2-Diazido-1-(1-tosyl-1*H*-indol-3-yl)propyl)benzonitrile (2w)**

Preparative thin layer chromatography (eluent: PE/EA = 5:1, v/v) to afford **2w** as a light-yellow oil (33.2 mg, 67%). $R_f$ = 0.5 (PE/EA = 5:1, v/v). $^1$H NMR (400 MHz, $CDCl_3$) δ 7.97 (d, *J* = 9.2 Hz, 2H), 7.78 (d, *J* = 8.1 Hz, 2H), 7.58 (d, *J* = 8.0 Hz, 2H), 7.54 (d, *J* = 8.2 Hz, 2H), 7.33 – 7.14 (m, 5H), 4.40 (s, 1H), 2.35 (s, 3H), 1.63 (s, 3H). $^{13}$C NMR (101 MHz, $CDCl_3$) δ 145.40, 142.75, 135.09, 134.68, 132.78, 132.25, 130.80, 130.07, 128.06, 126.96, 125.41, 124.82, 123.64, 118.92, 118.77, 114.01, 112.05, 82.81, 52.30, 23.94, 21.71. HR-ESI-MS m/z calcd for $C_{25}H_{20}N_8O_2NaS$ [(M+Na)$^+$]: 519.1328, found: 519.1329. IR: 3053(w), 2921(w), 2228(m), 2122(s), 2103(s), 1917(w), 1598(m), 1502(w), 1447(m), 1368(s), 1238(m), 1173(s), 1134(m), 1120(m), 1090(s), 1020(m), 976(s), 883(w), 812(m), 746(s), 674(s), 573(s), 537(s) cm$^{-1}$.

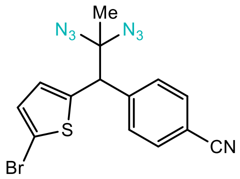


**4-(2,2-Diazido-1-(5-bromothiophen-2-yl)propyl)benzonitrile (2x)**

Preparative thin layer chromatography (eluent: PE/EA = 10:1, v/v) to afford **2w** as a light-yellow oil (27.9 mg, 72%). $R_f$ = 0.3 (PE/EA = 10:1, v/v). $^1$H NMR (400 MHz, $CDCl_3$) δ 7.66 – 7.58 (m, 4H), 6.92 (d, *J* = 3.8 Hz, 1H), 6.78 (d, *J* = 3.8 Hz, 1H), 4.35 (s, 1H), 1.61 (s, 3H). $^{13}$C NMR (101 MHz, $CDCl_3$) δ 142.83, 140.86, 132.42, 130.58, 129.41, 128.63, 118.48, 113.42, 112.23, 82.61, 77.48, 77.16, 76.84, 57.86, 23.83. HR-ESI-MS m/z calcd for $C_{14}H_{10}N_7SBrNa$ [(M+Na)$^+$]: 409.9799, found: 409.9806. IR: 3329(w), 3096(w), 2933(w), 2461(w), 2229(m), 2102(s), 1607(m), 1503(m), 1436(m), 1382(m), 1234(s), 1179(m), 1084(s), 1021(m), 967(s), 839(m), 797(s), 738(m), 682(m), 626(m), 556(s), 513(m) cm$^{-1}$.

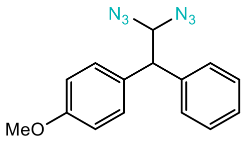


**1-(2,2-Diazido-1-phenylethyl)-4-methoxybenzene (2y)**

Preparative thin layer chromatography (eluent: PE/EA = 50:1, v/v) to afford **2y** as a light-yellow oil (26.5 mg, 90%). $R_f$ = 0.50 (PE/EA = 50:1, v/v). $^1$H NMR (400 MHz, $CDCl_3$) δ 7.36 – 7.28 (m, 5H), 7.24 (d, *J* = 8.8 Hz, 2H), 6.87 (d, *J* = 8.7 Hz, 2H), 5.31 (d, *J* = 8.8 Hz, 1H), 4.11 (d, *J* = 8.8 Hz, 1H), 3.79 (s, 3H). $^{13}$C NMR (101 MHz, $CDCl_3$) δ 159.03, 139.49, 131.18, 129.63, 128.95, 128.44, 127.56, 114.34, 80.38, 55.38, 55.26. HR-ESI-MS m/z calcd for $C_{15}H_{15}N_6O$ [$(M+H)^+$]: 295.1307, found: 295.1317. IR: 3323(w), 3031(w), 2934(w), 2837(w), 2477(w), 2096(s), 1884(w), 1610(m), 1511(s), 1455(m), 1303(m), 1245(s), 1179(s), 1117(m), 1032(s), 949(m), 815(m), 727(m), 698(s), 616(m), 565(m), 535(m) $cm^{-1}$.

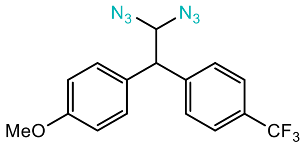


**1-(2,2-Diazido-1-(4-(trifluoromethyl)phenyl)ethyl)-4-methoxybenzene (2z)**

Preparative thin layer chromatography (eluent: PE/EA = 20:1, v/v) to afford **2z** as a colorless oil (29.3 mg, 81%). $R_f$ = 0.50 (PE/EA = 20:1, v/v). $^1$H NMR (400 MHz, $CDCl_3$) δ 7.59 (d, *J* = 8.6 Hz, 2H), 7.43 (d, *J* = 8.2 Hz, 2H), 7.21 (d, *J* = 8.6 Hz, 2H), 6.88 (d, *J* = 8.8 Hz, 2H), 5.32 (d, *J* = 8.6 Hz, 1H), 4.17 (d, *J* = 8.6 Hz, 1H), 3.79 (s, 3H). $^{13}$C NMR (101 MHz, $CDCl_3$) δ 159.37, 143.51, 130.22, 129.87 (q, *J* = 26.5 Hz), 129.69, 128.96, 125.86 (q, *J* = 3.8 Hz), 124.14 (q, *J* = 272.2 Hz), 114.58, 80.01, 55.42, 55.04. $^{19}$F NMR (376 MHz, $CDCl_3$) δ -62.58. HR-ESI-MS m/z calcd for $C_{16}H_{14}N_6OF_3$ [$(M+H)^+$]: 363.1181, found: 363.1179. IR: 2936(w), 2840(w), 2100(s), 1614(m), 1512(s), 1464(m), 1419(m), 1323(s), 1247(s), 1164(s), 1116(s), 1068(s), 1032(m), 1018(s), 950(m), 817(s), 602(m), 562(m), 535(m) $cm^{-1}$.

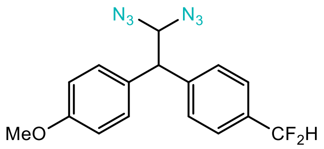


**1-(2,2-Diazido-1-(4-(difluoromethyl)phenyl)ethyl)-4-methoxybenzene (2aa)**

Preparative thin layer chromatography (eluent: PE/EA = 10:1, v/v) to afford **2aa** as a light-yellow oil (23.7 mg, 69%). $R_f$ = 0.33 (PE/EA = 10:1, v/v). $^1$H NMR (400 MHz, $CDCl_3$) δ 7.48 (d, *J* = 8.0 Hz, 2H), 7.40 (d, *J* = 8.1 Hz, 2H), 7.22 (d, *J* = 8.5 Hz, 2H), 6.88 (d, *J* = 8.5 Hz, 2H), 6.62 (t, *J* = 56.5 Hz, 1H), 5.32 (d, *J* = 8.6 Hz, 1H), 4.16 (d, *J* = 8.6 Hz, 1H), 3.79 (s, 3H).$^{13}$C NMR (101 MHz, $CDCl_3$) δ 159.23, 142.27, 133.61 (t, *J* = 22.6 Hz), 130.49, 129.65, 128.86, 126.18 (t, *J* = 6.1 Hz), 114.56 (t, *J* = 238.9 Hz), 114.48, 80.09, 55.40, 55.02. $^{19}$F NMR (376 MHz, $CDCl_3$) δ -110.84. HR-ESI-MS m/z calcd for $C_{16}H_{14}N_6OF_2Na$ $[(M+Na)^+]$: 367.1095, found: 367.1092. IR: 2959(w), 2935(w), 2839(w), 2099(s), 1720(w), 1612(m), 1512(s), 1463(m), 1376(m), 1303(m), 1246(s), 1222(s), 1179(s), 1115(m), 1072(s), 1021(s), 950(s), 902(w), 803(s), 668(w), 580(m), 554(m), 529(m) $cm^{-1}$.

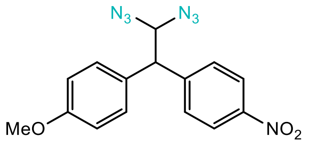


**1-(2,2-Diazido-1-(4-methoxyphenyl)ethyl)-4-nitrobenzene (2ab)**

Preparative thin layer chromatography (eluent: PE/EA = 8:1, v/v) to afford **2ab** as a light-yellow oil (30.8 mg, 91%). $R_f$ = 0.45 (PE/EA = 8:1, v/v). $^1$H NMR (400 MHz, $CDCl_3$) δ 8.19 (d, *J* = 8.8 Hz, 2H), 7.48 (d, *J* = 8.8 Hz, 2H), 7.20 (d, *J* = 8.7 Hz, 2H), 6.89 (d, *J* = 8.7 Hz, 2H), 5.34 (d, *J* = 8.3 Hz, 1H), 4.22 (d, *J* = 8.3 Hz, 1H), 3.79 (s, 3H). $^{13}$C NMR (101 MHz, $CDCl_3$) δ 159.53, 147.29, 146.78, 129.71, 129.58, 124.06, 114.71, 79.74, 55.44, 54.98. HR-ESI-MS m/z calcd for $C_{15}H_{14}N_7O_3$ $[(M+H)^+]$: 340.1158, found: 340.1152. IR: 3079(w), 2935(w), 2839(w), 2455(w), 2100(s), 1607(m), 1512(s), 1463(w), 1345(s), 1247(s), 1180(s), 1112(m), 1031(s), 952(m), 807(m), 729(m), 695(m), 570(m), 537(m) $cm^{-1}$.

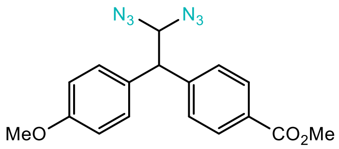


**Methyl 4-(2,2-diazido-1-(4-methoxyphenyl)ethyl)benzoate (2ac)**

Preparative thin layer chromatography (eluent: PE/EA = 10:1, v/v) to afford **2ac** as a light-yellow oil (33.1 mg, 94%). $R_f$ = 0.30 (PE/EA = 10:1, v/v). $^1$H NMR (400 MHz, $CDCl_3$) δ 8.03 (d, *J* = 8.4 Hz, 2H), 7.40 (d, *J* = 8.4 Hz, 2H), 7.23 (d, *J* = 8.7 Hz, 2H), 6.90 (d, *J* = 8.7 Hz, 2H), 5.35 (d, *J* = 8.7 Hz, 1H), 4.19 (d, *J* = 8.7 Hz, 1H), 3.92 (s, 3H), 3.80 (s, 3H).$^{13}$C NMR (101 MHz, $CDCl_3$) δ 166.77, 159.23, 144.56, 130.32, 130.18, 129.66, 129.37, 128.53, 114.46, 114.46, 79.99, 55.37, 55.16, 52.27. HR-ESI-MS m/z calcd for $C_{17}H_{16}N_6O_3Na$ $[(M+Na)^+]$: 375.1182, found: 375.1180. IR: 2953(w), 2838(w), 2099(s), 1717(s), 1611(s), 1512(s), 1461(m), 1436(m), 1279(s), 1246(s), 1180(s), 1110(s), 1032(s), 1020(s), 953(m), 830(m), 804(m), 731(m), 702(m), 658(w), 570(m), 537(m) cm$^{-1}$.

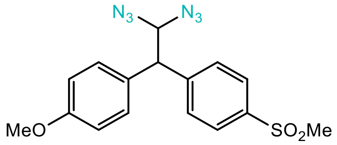


**1-(2,2-Diazido-1-(4-(methylsulfonyl)phenyl)ethyl)-4-methoxybenzene (2ad)**

Preparative thin layer chromatography (eluent: PE/EA = 3:1, v/v) to afford **2ad** as a light-yellow oil (27.0 mg, 72%). $R_f$ = 0.40 (PE/EA = 3:1, v/v). $^1$H NMR (400 MHz, $CDCl_3$) δ 7.90 (d, *J* = 8.4 Hz, 2H), 7.51 (d, *J* = 8.4 Hz, 2H), 7.20 (d, *J* = 8.7 Hz, 2H), 6.88 (d, *J* = 8.7 Hz, 2H), 5.36 (d, *J* = 8.5 Hz, 1H), 4.20 (d, *J* = 8.5 Hz, 1H), 3.79 (s, 3H), 3.04 (s, 3H).$^{13}$C NMR (101 MHz, $CDCl_3$) δ 159.39, 145.76, 139.59, 129.71, 129.67, 129.59, 127.94, 114.60, 79.72, 55.40, 55.01, 44.60. HR-ESI-MS m/z calcd for $C_{16}H_{16}N_6O_3NaS$ $[(M+Na)^+]$: 395.0902, found: 395.0903. IR: 3306(w), 2927(w), 2838(w), 2100(s), 1610(s), 1512(s), 1463(m), 1409(m), 1301(s), 1246(s), 1180(m), 1146(s), 1091(s), 1030(s), 901(w), 816(m), 763(s), 677(m), 575(s), 524(s) cm$^{-1}$.

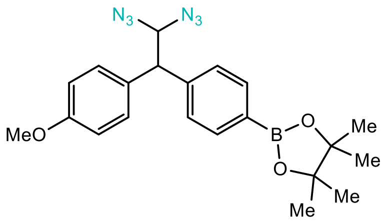


**2-(4-(2,2-Diazido-1-(4-methoxyphenyl)ethyl)phenyl)-4,4,5,5-tetramethyl-1,3,2-dioxaborolane (2ae)**

Preparative thin layer chromatography (eluent: PE/EA = 10:1, v/v) to afford **2ae** as a colorless oil (21.4 mg, 51%). $R_f$ = 0.30 (PE/EA = 10:1, v/v). $^1$H NMR (400 MHz, $CDCl_3$) δ 7.78 (d, *J* = 8.1 Hz, 2H), 7.31 (d, *J* = 8.0 Hz, 2H), 7.21 (d, *J* = 8.7 Hz, 2H), 6.85 (d, *J* = 8.8 Hz, 2H), 5.32 (d, *J* = 9.0 Hz, 1H), 4.11 (d, *J* = 9.0 Hz, 1H), 3.78 (s, 3H), 1.33 (s, 12H). $^{13}$C NMR (101 MHz, $CDCl_3$) δ 159.09, 142.60, 135.41, 131.00, 129.64, 127.78, 114.38, 83.97, 80.28, 55.41, 55.39, 24.99. The signal of the carbon directly attached to the boron atom was not detected due to the quadrupolar broadening. HR-ESI-MS m/z calcd for $C_{21}H_{25}N_6O_3BNa$ [(M+Na)$^+$]: 443.1979, found: 443.1970. IR: 2978(w), 2929(w), 2838(w), 2100(s), 1611(s), 1512(s), 1463(m), 1399(m), 1358(s), 1324(m), 1247(s), 1179(m), 1142(s), 1090(s), 1033(m), 1022(m), 962(m), 858(s), 822(m), 801(m), 726(m), 657(s), 571(m), 538(m) cm$^{-1}$.

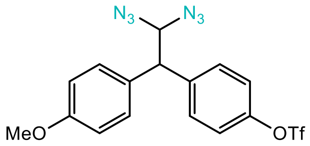


**4-(2,2-Diazido-1-(4-methoxyphenyl)ethyl)phenyl trifluoromethanesulfonate (2af)**

Preparative thin layer chromatography (eluent: PE/EA = 20:1, v/v) to afford **2af** as a yellow oil (39.3 mg, 89%). $R_f$ = 0.40 (PE/EA = 20:1, v/v). $^1$H NMR (400 MHz, $CDCl_3$) δ 7.39 (d, *J* = 8.7 Hz, 2H), 7.24 (d, *J* = 8.8 Hz, 2H), 7.20 (d, *J* = 8.7 Hz, 2H), 6.89 (d, *J* = 8.7 Hz, 2H), 5.28 (d, *J* = 8.2 Hz, 1H), 4.16 (d, *J* = 8.2 Hz, 1H), 3.80 (s, 3H).$^{13}$C NMR (101 MHz, $CDCl_3$) δ 159.33, 148.77, 139.97, 130.51, 130.07, 129.69, 121.71, 118.84 (q, *J* = 320.8 Hz), 114.55, 79.95, 55.40, 54.51. $^{19}$F NMR (376 MHz, $CDCl_3$) δ -72.82. HR-ESI-MS m/z calcd for $C_{16}H_{13}N_6O_4NaSF_3$ [(M+Na)$^+$]: 465.0569, found: 465.0576. IR: 3321(w), 2969(w), 2840(w), 2445(w), 2103(s), 1901(w), 1611(m), 1512(s), 1464(w), 1419(s), 1304(w), 1247(s), 1207(s), 1182(s), 1136(s), 1032(m), 1017(m), 944(w), 885(s),

828(m), 777(m), 743(m), 701(w), 605(s), 565(m), 518(m) cm$^{-1}$.

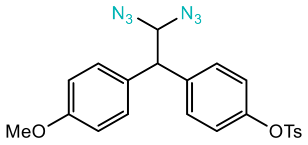


**4-(2,2-Diazido-1-(4-methoxyphenyl)ethyl)phenyl 4-methylbenzenesulfonate (2ag)**

Preparative thin layer chromatography twice (eluent: PE/EA = 5:1, v/v) to afford **2ag** as a yellow oil (39.5 mg, 85%). $R_f$ = 0.50 (PE/EA = 5:1, v/v). $^1$H NMR (400 MHz, $CDCl_3$) δ 7.68 (d, $J$ = 8.5 Hz, 2H), 7.29 (d, $J$ = 7.8 Hz, 2H), 7.23 (d, $J$ = 8.9 Hz, 2H), 7.17 (d, $J$ = 8.2 Hz, 2H), 6.95 (d, $J$ = 8.7 Hz, 2H), 6.87 (d, $J$ = 8.6 Hz, 2H), 5.23 (d, $J$ = 8.2 Hz, 1H), 4.08 (d, $J$ = 8.3 Hz, 1H), 3.79 (s, 3H), 2.44 (s, 3H). $^{13}$C NMR (101 MHz, $CDCl_3$) δ 159.16, 148.89, 145.54, 138.31, 132.41, 130.43, 129.89, 129.85, 129.65, 128.62, 122.76, 114.39, 80.08, 55.38, 54.60, 21.84. HR-ESI-MS m/z calcd for $C_{22}H_{20}N_6O_4NaS$ [$(M+Na)^+$]: 487.1164, found: 487.1166. IR: 2933(w), 2838(w), 2099(s), 1918(w), 1610(m), 1502(s), 1461(m), 1370(s), 1303(m), 1247(s), 1176(s), 1152(s), 1118(w), 1092(s), 1032(m), 1018(m), 950(m), 863(s), 813(s), 741(s), 706(m), 660(s), 550(s) cm$^{-1}$.

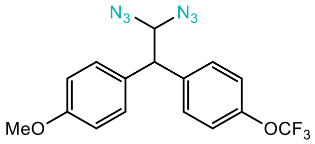


**1-(2,2-Diazido-1-(4-(trifluoromethoxy)phenyl)ethyl)-4-methoxybenzene (2ah)**

Preparative thin layer chromatography twice (eluent: PE/EA = 50:1, v/v) to afford **2ah** as a light-yellow oil (26.8 mg, 71%). $R_f$ = 0.3 (PE/EA = 50:1, v/v). $^1$H NMR (400 MHz, $CDCl_3$)δ 7.34 (d, $J$ = 8.7 Hz, 2H), 7.21 (d, $J$ = 8.7 Hz, 2H), 7.18 (d, $J$ = 9.0 Hz, 2H), 6.89 (d, $J$ = 8.7 Hz, 2H), 5.28 (d, $J$ = 8.4 Hz, 1H), 4.14 (d, $J$ = 8.4 Hz, 1H), 3.80 (s, 3H).$^{13}$C NMR (101 MHz, $CDCl_3$) δ 159.27, 148.58, 138.12, 130.57, 129.99, 129.66, 121.28, 120.59 (d, $J$ = 257.7 Hz), 114.51, 80.18, 55.40, 54.58. $^{19}$F NMR (377 MHz, $CDCl_3$) δ -57.83. HR-ESI-MS m/z calcd for $C_{16}H_{13}N_3O_2F_3$ [$(M-N_3)^+$]: 336.0960, found: 336.0960. IR: 2959(w), 2937(w), 2840(w), 2469(w), 2101(s), 1611(m), 1510(s), 1464(w), 1247(s), 1209(s), 1159(s), 1118(m), 1033(s), 954(m), 923(m), 915(s), 676(m), 627(w), 572(m), 541(m) cm$^{-1}$.

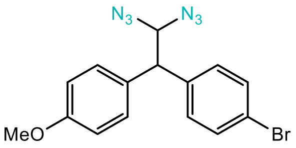


**1-Bromo-4-(2,2-diazido-1-(4-methoxyphenyl)ethyl)benzene (2ai)**

Preparative thin layer chromatography twice (eluent: PE/EA = 20:1, v/v) to afford **2ai** as a yellow oil (28.6 mg, 77%). $R_f$ = 0.5 (PE/EA = 20:1, v/v). $^1$H NMR (400 MHz, $CDCl_3$) δ 7.46 (d, *J* = 8.4 Hz, 2H), 7.19 (dd, *J* = 8.6, 6.3 Hz, 4H), 6.87 (d, *J* = 8.7 Hz, 2H), 5.26 (d, *J* = 8.5 Hz, 1H), 4.07 (d, *J* = 8.5 Hz, 1H), 3.79 (s, 3H).$^{13}$C NMR (101 MHz, $CDCl_3$) δ 159.24, 138.51, 132.03, 130.57, 130.26, 129.62, 121.59, 114.48, 80.10, 55.41, 54.67. HR-ESI-MS m/z calcd for $C_{15}H_{14}N_6OBr$ [$(M+H)^+$]: 373.0412, found: 3473.0417. IR: 3323(w), 3033(w), 2933(w), 2837(w), 2464(w), 2097(s), 1898(w), 1610(m), 1511(s), 1488(s), 1405(w), 1303(m), 1245(s), 1178(s), 1119(m), 1074(s), 1031(s), 1010(s), 952(m), 900(m), 854(m), 806(s), 771(m), 738(m), 655(m), 568(s), 536(m) $cm^{-1}$.

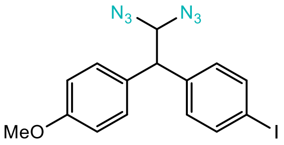


**1-(2,2-Diazido-1-(4-iodophenyl)ethyl)-4-methoxybenzene (2aj)**

Preparative thin layer chromatography twice (eluent: PE/EA = 50:1, v/v) to afford **2aj** as a yellow oil (33.6 mg, 76%). $R_f$ = 0.3 (PE/EA = 50:1, v/v). $^1$H NMR (400 MHz, $CDCl_3$) δ 7.66 (d, *J* = 8.4 Hz, 2H), 7.19 (d, *J* = 8.7 Hz, 2H), 7.05 (d, *J* = 8.4 Hz, 2H), 6.87 (d, *J* = 8.7 Hz, 2H), 5.26 (d, *J* = 8.5 Hz, 1H), 4.06 (d, *J* = 8.5 Hz, 1H), 3.79 (s, 3H).$^{13}$C NMR (101 MHz, $CDCl_3$) δ 159.22, 139.19, 137.99, 130.47, 129.60, 114.47, 93.10, 80.03, 55.40, 54.76. HR-ESI-MS m/z calcd for $C_{15}H_{14}N_6OI$ [$(M+H)^+$]: 421.0274, found: 421.0265. IR: 3324(w), 3001(w), 2932(w), 2836(w), 2480(w), 2096(s), 1899(w), 1610(m), 1584(m), 1511(s), 1484(s), 1303(m), 1245(s), 1178(s), 1119(m), 1032(s), 1006(s), 954(m), 806(s), 737(s), 673(w), 567(m), 536(m) $cm^{-1}$.

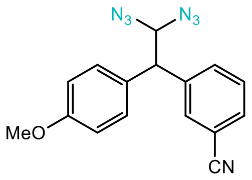


**3-(2,2-Diazido-1-(4-methoxyphenyl)ethyl)benzonitrile (2ak)**

Preparative thin layer chromatography (eluent: PE/EA = 10:1, v/v) to afford **2ak** as a yellow oil (18.7 mg, 59%). $R_f$ = 0.3 (PE/EA = 10:1, v/v). $^1$H NMR (400 MHz, $CDCl_3$) δ 7.60 (s, 1H), 7.58 – 7.53 (m, 2H), 7.44 (t, *J* = 7.8 Hz, 1H), 7.20 (d, *J* = 8.7 Hz, 2H), 6.90 (d, *J* = 8.7 Hz, 2H), 5.31 (d, *J* = 8.3 Hz, 1H), 4.15 (d, *J* = 8.2 Hz, 1H), 3.80 (s, 3H). $^{13}$C NMR (101 MHz, $CDCl_3$) δ 159.47, 141.01, 133.20, 132.31, 131.18, 129.71, 129.69, 129.63, 118.66, 114.67, 113.08, 79.77, 55.43, 54.72. HR-ESI-MS m/z calcd for $C_{16}H_{13}N_7ONa$ [(M+Na)$^+$]: 342.1076, found: 342.1075. IR: 3322(w), 3004(w), 2935(w), 2838(w), 2467(w), 2230(m), 2099(s), 1894(w), 1611(m), 1583(m), 1512(s), 1462(m), 1440(m), 1303(m), 1246(s), 1180(s), 1117(m), 1031(s), 959(m), 919(m), 831(m), 801(m), 747(m), 690(s), 553(m), 531(m) cm$^{-1}$.

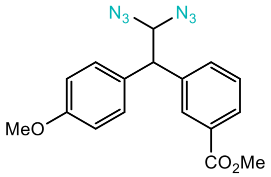


**Methyl 3-(2,2-diazido-1-(4-methoxyphenyl)ethyl)benzoate (2al)**

Preparative thin layer chromatography (eluent: PE/EA = 8:1, v/v) to afford **2al** as a colorless oil (29.2 mg, 83%). $R_f$ = 0.3 (PE/EA = 10:1, v/v). $^1$H NMR (400 MHz, $CDCl_3$) δ 8.00 (s, 1H), 7.95 (d, *J* = 7.7 Hz, 1H), 7.50 (d, *J* = 7.6 Hz, 1H), 7.41 (t, *J* = 7.7 Hz, 1H), 7.23 (d, *J* = 8.3 Hz, 2H), 6.88 (d, *J* = 8.3 Hz, 2H), 5.36 (d, *J* = 8.8 Hz, 1H), 4.16 (d, *J* = 8.8 Hz, 1H), 3.92 (s, 3H), 3.78 (s, 3H). $^{13}$C NMR (101 MHz, $CDCl_3$) δ 166.85, 159.14, 139.93, 133.07, 130.76, 130.55, 129.61, 129.44, 129.03, 128.75, 114.44, 80.07, 55.37, 55.01, 52.38. HR-ESI-MS m/z calcd for $C_{17}H_{16}N_6O_3Na$ [(M+Na)$^+$]: 375.1182, found: 375.1178. IR: 3321(w), 3002(w), 2953(w), 2838(w), 2467(w), 2099(s), 1716(s), 1610(s), 1585(m), 1512(s), 1442(m), 1286(s), 1245(s), 1198(s), 1179(s), 1110(s), 1088(m), 1032(s), 987(m), 952(m), 832(m), 745(s), 732(s), 693(s), 621(s), 573(s) cm$^{-1}$.

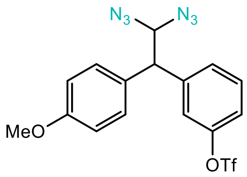


**3-(2,2-Diazido-1-(4-methoxyphenyl)ethyl)phenyl trifluoromethanesulfonate (2am)**

Preparative thin layer chromatography (eluent: PE/EA = 10:1, v/v) to afford **2am** as a colorless oil (37.6 mg, 86%). $R_f$ = 0.45 (PE/EA = 10:1, v/v). $^1$H NMR (400 MHz, $CDCl_3$) δ 7.42 (t, $J$ = 8.0 Hz, 1H), 7.34 (d, $J$ = 7.7 Hz, 1H), 7.28 – 7.12 (m, 4H), 6.90 (d, $J$ = 8.7 Hz, 2H), 5.29 (d, $J$ = 8.1 Hz, 1H), 4.16 (d, $J$ = 8.1 Hz, 1H), 3.80 (s, 3H).$^{13}$C NMR (101 MHz, $CDCl_3$) δ 159.39, 149.78, 142.37, 130.61, 129.76, 129.70, 128.72, 121.70, 120.36, 118.86 (q, $J$ = 320.8 Hz), 114.57, 79.84, 55.41, 54.71. $^{19}$F NMR (376 MHz, $CDCl_3$) δ -72.73. HR-ESI-MS m/z calcd for $C_{16}H_{13}N_6O_4NaSF_3$ [(M+Na)$^+$]: 465.0569, found: 465.0572. IR: 3006(w), 2937(w), 2840(w), 2453(w), 2102(s), 1612(s), 1582(m), 1513(s), 1486(m), 1420(s), 1303(w), 1246(s), 1206(s), 1181(s), 1137(s), 1118(s), 1032(m), 963(m), 935(m), 856(s), 824(s), 794(m), 746(m), 689(m), 604(s), 567(m), 511(m) cm$^{-1}$.

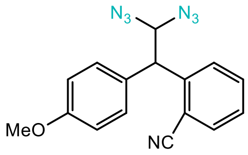


**2-(2,2-Diazido-1-(4-methoxyphenyl)ethyl)benzonitrile (2an)**

Preparative thin layer chromatography (eluent: PE/EA = 10:1, v/v) to afford **2ak** as a yellow oil (18.1 mg, 57%). $R_f$ = 0.3 (PE/EA = 10:1, v/v). $^1$H NMR (400 MHz, $CDCl_3$) δ 7.68 – 7.50 (m, 3H), 7.37 (td, $J$ = 7.5, 1.4 Hz, 1H), 7.27 (d, $J$ = 8.7 Hz, 2H), 6.88 (d, $J$ = 8.7 Hz, 2H), 5.42 (d, $J$ = 8.7 Hz, 1H), 4.59 (d, $J$ = 8.6 Hz, 1H), 3.79 (s, 3H).$^{13}$C NMR (101 MHz, $CDCl_3$) δ 159.49, 143.15, 133.67, 133.11, 129.89, 129.18, 128.15, 127.90, 117.73, 114.60, 113.52, 79.72, 55.40, 53.05. HR-ESI-MS m/z calcd for $C_{16}H_{13}N_7ONa$ [(M+Na)$^+$]: 342.1079, found: 342.1071. IR: 2959(w), 2934(w), 2838(w), 2224(m), 2105(s), 1721(m), 1610(m), 15152(s), 1486(m), 1462(m), 1304(m), 1249(s), 1181(s), 1118(m), 1032(s), 957(m), 821(m), 760(s), 619(m), 555(m) cm$^{-1}$.

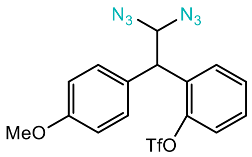


**2-(2,2-Diazido-1-(4-methoxyphenyl)ethyl)phenyl trifluoromethanesulfonate (2ao)**

Preparative thin layer chromatography (eluent: PE/EA = 10:1, v/v) to afford **2ao** as a yellow oil (33.6 mg, 67%). $R_f$ = 0.45 (PE/EA = 10:1, v/v). $^1$H NMR (400 MHz, $CDCl_3$) δ 7.55 (d, *J* = 7.5 Hz, 1H), 7.39 (dt, *J* = 14.9, 7.2 Hz, 2H), 7.31 (d, *J* = 7.4 Hz, 1H), 7.24 (d, *J* = 8.5 Hz, 2H), 6.88 (d, *J* = 8.4 Hz, 2H), 5.31 (d, *J* = 8.2 Hz, 1H), 4.54 (d, *J* = 8.3 Hz, 1H), 3.79 (s, 3H). $^{13}$C NMR (101 MHz, $CDCl_3$) δ 159.38, 147.68, 132.57, 129.96, 129.81, 129.34, 128.84, 128.62, 121.95, 118.61 (q, *J* = 319.9 Hz), 114.44, 79.79, 77.48, 77.16, 76.84, 55.38, 47.94. $^{19}$F NMR (376 MHz, $CDCl_3$) δ -73.80. HR-ESI-MS m/z calcd for $C_{16}H_{14}N_6O_4SF_3$ [(M+H)$^+$]: 443.0749, found: 443.0743. IR: 3326(w), 3034(w), 2936(w), 2839(w), 2102(s), 1611(m), 1513(m), 1489(m), 1418(s), 1371(m), 1247(s), 1212(s), 1174(s), 1136(s), 1090(s), 1033(m), 977(m), 894(s), 854(m), 812(m), 763(m), 746(m), 703(m), 676(m), 571(s), 537(m), 521(m) cm$^{-1}$.

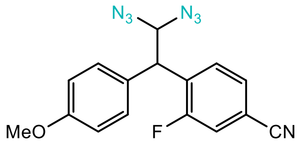


**4-(2,2-Diazido-1-(4-methoxyphenyl)ethyl)-3-fluorobenzonitrile (2ap)**

Preparative thin layer chromatography (eluent: PE/EA = 10:1, v/v) to afford **2ap** as a yellow oil (27.7 mg, 82%). $R_f$ = 0.30 (PE/EA = 10:1, v/v). $^1$H NMR (400 MHz, $CDCl_3$) δ 7.53 – 7.42 (m, 2H), 7.37 (d, *J* = 9.7 Hz, 1H), 7.22 (d, *J* = 9.0 Hz, 2H), 6.88 (d, *J* = 7.8 Hz, 2H), 5.43 (d, *J* = 8.9 Hz, 1H), 4.40 (d, *J* = 8.9 Hz, 1H), 3.79 (s, 3H). $^{13}$C NMR (101 MHz, $CDCl_3$) δ 160.03 (d, *J* = 250.0 Hz), 159.56, 132.88 (d, *J* = 14.0 Hz), 130.83 (d, *J* = 4.6 Hz), 129.70, 128.57, 128.52, 119.87 (d, *J* = 26.7 Hz), 117.38 (d, *J* = 2.8 Hz), 114.67, 114.64, 114.62, 112.83 (d, *J* = 10.1 Hz), 79.19, 79.17, 55.41, 49.25. $^{19}$F NMR (376 MHz, $CDCl_3$) δ -112.73. HR-ESI-MS m/z calcd for $C_{16}H_{12}N_7OFNa$ [(M+Na)$^+$]: 360.0985, found: 360.0990. IR: 3322(w), 3004(w), 2958(w), 2839(w), 2457(w), 2235(w), 2103(s), 1610(m), 1569(m), 1512(s), 1462(m), 1415(m), 1305(m), 1247(s), 1180(s), 1115(m), 1031(s), 946(m), 877(m), 747(w), 622(m), 543(m), 472(m) cm$^{-1}$.

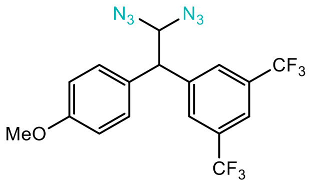


**1-(2,2-Diazido-1-(4-methoxyphenyl)ethyl)-3,5-bis(trifluoromethyl)benzene (2aq)**

Preparative thin layer chromatography (eluent: PE/EA = 10:1, v/v) to afford **2aq** as a yellow oil (42.8 mg, 99%). $R_f$ = 0.70 (PE/EA = 10:1, v/v). $^1$H NMR (400 MHz, $CDCl_3$) δ 7.80 (s, 1H), 7.76 (s, 2H), 7.21 (d, *J* = 8.2 Hz, 2H), 6.92 (d, *J* = 8.2 Hz, 2H), 5.36 (d, *J* = 7.9 Hz, 1H), 4.25 (d, *J* = 8.0 Hz, 1H), 3.81 (s, 3H). $^{13}$C NMR (101 MHz, $CDCl_3$) δ 159.60, 141.96, 132.10 (q, *J* = 33.4 Hz), 129.73, 129.18 (q, *J* = 15.6 Hz), 128.92, 123.28 (q, *J* = 272.7 Hz), 121.64 (t, *J* = 3.8 Hz), 114.79, 79.59, 55.44, 54.79. $^{19}$F NMR (376 MHz, $CDCl_3$) δ -62.79. HR-ESI-MS m/z calcd for $C_{17}H_{13}N_6OF_6$ [$(M+H)^+$]: 431.1055, found: 431.1054. IR: 3006(w), 2938(w), 2841(w), 2103(s), 1612(m), 1513(s), 1465(m), 1375(s), 1276(s), 1251(s), 1168(s), 1126(s), 1033(s), 964(m), 900(m), 828(m), 704(s), 681(s), 647(m), 557(m) $cm^{-1}$.

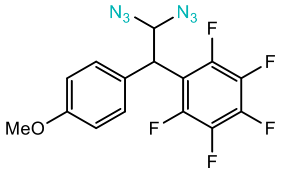


**1-(2,2-Diazido-1-(4-methoxyphenyl)ethyl)-2,3,4,5,6-pentafluorobenzene (2ar)**

Preparative thin layer chromatography twice (eluent: PE/EA = 100:1, v/v) to afford **2ar** as a yellow oil (23.8 mg, 62%). $R_f$ = 0.33 (PE/EA = 100:1, v/v). $^1$H NMR (400 MHz, $CDCl_3$) δ 7.27 (d, *J* = 8.7 Hz, 2H), 6.89 (d, *J* = 8.7 Hz, 2H), 5.63 (d, *J* = 10.8 Hz, 1H), 4.37 (d, *J* = 10.7 Hz, 1H), 3.80 (s, 3H).$^{13}$C NMR (101 MHz, $CDCl_3$) δ 159.82, 146.59-145.98 (m), 143.93-143.54 (m), 141.80-141.68 (m), 139.37-139.21 (m), 136.93-136.54 (m), 129.44, 128.15, 114.88, 114.10-113.80 (m), 78.66 (t, *J* = 4.4 Hz), 55.45, 46.38. $^{19}$F NMR (376 MHz, $CDCl_3$) δ -141.46 – -141.60 (m), -154.63 (t, *J* = 21.0 Hz), -160.73 (td, *J* = 22.4, 7.6 Hz). HR-ESI-MS m/z calcd for $C_{15}H_9N_3OF_5$ [$(M- N_3)^+$]: 342.0666, found: 342.0664. IR: 3005(w), 2937(w), 2841(w), 2104(s), 1655(w), 1612(m), 1498(s), 1307(m), 1248(s), 1181(s), 1118(m), 1076(m), 1034(m), 1001(s), 957(s), 952(m),

830(m), 788(m), 740(m), 677(m), 556(m), 532(m), 443(m) $cm^{-1}$.

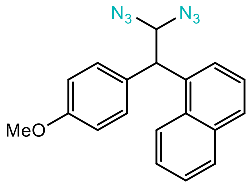


**1-(2,2-Diazido-1-(4-methoxyphenyl)ethyl)naphthalene (2as)**

Preparative thin layer chromatography twice (eluent: PE/EA = 50:1, v/v) to afford **2as** as a yellow oil (18.9 mg, 55%, dr = 5:1). $R_f$ = 0.33 (PE/EA = 50:1, v/v). $^1$H NMR (400 MHz, $CDCl_3$) δ 8.04 (d, *J* = 8.0 Hz, 1.2H), 7.89 – 7.78 (m, 2.4H), 7.60 (d, *J* = 7.1 Hz, 1H), 7.57 – 7.44 (m, 3.4H), 7.40 (d, *J* = 7.4 Hz, 0.4H), 7.31 (d, *J* = 8.3 Hz, 2H), 7.04 (d, *J* = 8.2 Hz, 0.4H), 6.84 (d, *J* = 9.0 Hz, 2H), 6.70 (d, *J* = 8.3 Hz, 0.4H), 5.50 (d, *J* = 8.3 Hz, 1H), 5.35 (d, *J* = 7.7 Hz, 0.2H), 5.02 (d, *J* = 7.7 Hz, 0.2H), 4.95 (d, *J* = 8.4 Hz, 1H), 3.75 (s, 3H), 3.71 (s, 0.6H). $^{13}$C NMR (101 MHz, $CDCl_3$) δ 159.73, 159.07, 135.13, 134.27, 133.97, 131.73, 131.62, 130.71, 130.05, 129.58, 129.28, 129.19, 128.63, 128.27, 126.73, 126.67, 125.93, 125.87, 125.23, 124.77, 123.18, 122.94, 114.24, 114.04, 80.78, 69.14, 55.32, 50.03. Some peaks superimposed together. HR-ESI-MS m/z calcd for $C_{19}H_{16}N_6ONa$ $[(M+Na)^+]$: 367.1283, found: 367.1278. IR: 3324(w), 3047(w), 2933(w), 2836(w), 2091(s), 1609(m), 1510(s), 1461(m), 1303(m), 1246(s), 1177(s), 1113(m), 1030(s), 947(m), 828(m), 799(m), 776(s), 732(m), 617(m), 543(m), 530(m), 442(m) $cm^{-1}$.

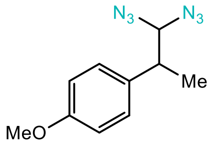


**1-(1,1-Diazidopropan-2-yl)-4-methoxybenzene (2at)**

Preparative thin layer chromatography (eluent: PE/EA = 50:1, v/v) to afford **2as** as a light-yellow oil (22.6 mg, 95%). $R_f$ = 0.45 (PE/EA = 50:1, v/v). $^1$H NMR (400 MHz, $CDCl_3$) δ 7.17 (d, *J* = 8.2 Hz, 2H), 6.89 (d, *J* = 8.5 Hz, 2H), 4.73 (d, *J* = 7.1 Hz, 1H), 3.81 (s, 3H), 2.95 (p, *J* = 7.0 Hz, 1H), 1.36 (d, *J* = 7.0 Hz, 3H). $^{13}$C NMR (101 MHz, $CDCl_3$) δ 159.09, 132.49, 129.06, 129.03, 114.19, 82.62, 55.39, 44.04, 17.09. HR-ESI-MS m/z calcd for $C_{10}H_{13}N_6O$ $[(M+H)^+]$: 233.1151, found: 233.1144. IR: 2970(w), 2936(w),

2837(w), 2096(s), 1612(m), 1513(s), 1461(m), 1303(w), 1242(s), 1179(s), 1122(m), 1037(s), 925(m), 830(s), 729(w), 651(w), 548(s) $cm^{-1}$.

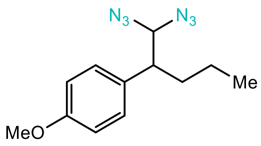


**1-(1,1-Diazidopentan-2-yl)-4-methoxybenzene (2au)**

Preparative thin layer chromatography (eluent: PE/EA = 50:1, v/v) to afford **2au** as a light-yellow oil (26.0 mg, 71%). $R_f$ = 0.50 (PE/EA = 50:1, v/v). $^1$H NMR (400 MHz, $CDCl_3$) δ 7.14 (d, *J* = 8.7 Hz, 2H), 6.88 (d, *J* = 8.7 Hz, 2H), 4.76 (d, *J* = 7.2 Hz, 1H), 3.81 (s, 3H), 2.77 (td, *J* = 7.1, 3.6 Hz, 1H), 1.78 (ddd, *J* = 9.4, 7.0, 3.9 Hz, 1H), 1.65 (dddd, *J* = 13.7, 10.9, 9.2, 5.2 Hz, 1H), 1.16 (dt, *J* = 16.2, 8.4 Hz, 2H), 0.86 (t, *J* = 7.3 Hz, 3H). $^{13}$C NMR (101 MHz, $CDCl_3$) δ 159.15, 130.72, 129.70, 114.21, 82.00, 55.36, 49.71, 33.39, 20.29, 13.99, 13.96. HR-ESI-MS m/z calcd for $C_{12}H_{17}N_6O$ $[(M+H)^+]$: 261.1464, found: 261.1455. IR: 2959(m), 2873(w), 2837(w), 2100(s), 1612(m), 1513(s), 1463(m), 1303(m), 1249(s), 1180(m), 1125(w), 1036(m), 945(m), 892(m), 830(m), 745(w), 663(w), 550(m) $cm^{-1}$.

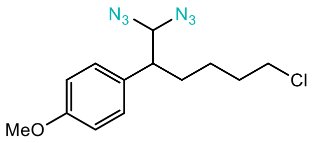


**1-(1,1-Diazido-6-chlorohexan-2-yl)-4-methoxybenzene (2av)**

Preparative thin layer chromatography (eluent: PE/EA = 50:1, v/v) to afford **2av** as a light-yellow oil (18.8 mg, 61%). $R_f$ = 0.40 (PE/EA = 50:1, v/v). $^1$H NMR (400 MHz, $CDCl_3$) δ 7.13 (d, *J* = 8.7 Hz, 2H), 6.89 (d, *J* = 8.7 Hz, 2H), 4.78 (d, *J* = 7.1 Hz, 1H), 3.81 (s, 3H), 3.46 (td, *J* = 6.7, 3.8 Hz, 2H), 2.75 (ddd, *J* = 11.1, 7.1, 3.9 Hz, 1H), 1.84 (dtt, *J* = 13.1, 6.7, 3.3 Hz, 1H), 1.81 – 1.61 (m, 3H), 1.32 – 1.21 (m, 2H). $^{13}$C NMR (101 MHz, $CDCl_3$) δ 159.21, 130.19, 129.64, 114.30, 81.82, 55.36, 49.84, 44.76, 32.44, 30.39, 24.49. HR-ESI-MS m/z calcd for $C_{13}H_{17}N_3OCl$ $[(M-N_3)^+]$: 266.1060, found: 266.1050. IR: 2938(m), 2866(w), 2837(w), 2453(w), 2098(s), 1611(m), 1583(w), 1512(s), 1461(m), 1303(w), 1243(s), 1179(s), 1114(m), 1033(s), 944(m), 830(s), 738(m), 651(m), 548(m)

cm$^{-1}$.

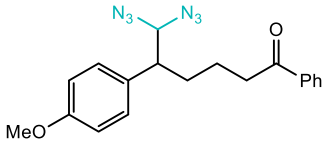


**6,6-Diazido-5-(4-methoxyphenyl)-1-phenylhexan-1-one** (**2aw**)

Preparative thin layer chromatography (eluent: PE/EA = 10:1, v/v) to afford **2aw** as a colorless oil (22.5 mg, 62%). $R_f$ = 0.33 (PE/EA = 10:1, v/v). $^1$H NMR (400 MHz, $CDCl_3$) δ 7.89 (d, *J* = 7.3 Hz, 2H), 7.57 – 7.51 (m, 1H), 7.44 (t, *J* = 7.8 Hz, 2H), 7.16 (d, *J* = 8.7 Hz, 2H), 6.88 (d, *J* = 8.8 Hz, 2H), 4.79 (d, *J* = 7.0 Hz, 1H), 3.80 (s, 3H), 2.92 (dt, *J* = 11.0, 7.2 Hz, 2H), 2.81 (ddd, *J* = 10.8, 7.0, 3.9 Hz, 1H), 1.99 – 1.86 (m, 1H), 1.84 – 1.70 (m, 1H), 1.64 – 1.54 (m, 3H). $^{13}$C NMR (151 MHz, $CDCl_3$) δ 199.80, 159.29, 137.02, 133.13, 130.15, 129.76, 128.71, 128.11, 114.35, 81.84, 55.37, 49.99, 38.28, 30.66, 21.87. HR-ESI-MS m/z calcd for $C_{19}H_{20}O_2N_6Na$ [(M+Na)$^+$]: 387.1540, found: 387.1544. IR: 2933(w), 2838(w), 2097(s), 1681(s), 1611(m), 1511(s), 1449(m), 1303(w), 1242(s), 1179(s), 1032(m), 830(m), 740(m), 687(s), 548(m) cm$^{-1}$.

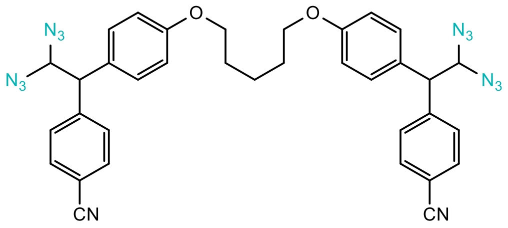


**4,4'-(((Pentane-1,5-diylbis(oxy))bis(4,1-phenylene))bis(2,2-diazidoethane-1,1-diyl))dibenzonitrile (2ax)**

**1ax** (25.5 mg, 0.05 mmol) was utilized as substrate. Preparative thin layer chromatography (eluent: PE/DCM = 1:3, v/v) to afford **2ax** as a yellow oil (31.4 mg, 93%). $R_f$ = 0.40 (PE/DCM = 1:3, v/v). $^1$H NMR (400 MHz, $CDCl_3$) δ 7.62 (d, *J* = 8.4 Hz, 4H), 7.42 (d, *J* = 8.3 Hz, 4H), 7.17 (d, *J* = 8.7 Hz, 4H), 6.87 (d, *J* = 8.7 Hz, 4H), 5.32 (d, *J* = 8.4 Hz, 2H), 4.16 (d, *J* = 8.4 Hz, 2H), 3.96 (t, *J* = 6.3 Hz, 4H), 1.84 (p, *J* = 6.6 Hz, 4H), 1.62 (p, *J* = 8.3 Hz, 2H). $^{13}$C NMR (101 MHz, $CDCl_3$) δ 158.87, 144.77, 132.61, 129.64, 129.54, 129.40, 118.64, 115.09, 111.46, 79.67, 67.86, 55.10, 29.05, 22.84. HR-ESI-MS m/z calcd for $C_{35}H_{30}N_{14}O_2Na$ [(M+Na)$^+$]: 701.2574, found: 701.2579. IR:

2941(w), 2870(w), 2228(m), 2099(s), 1609(s), 1581(w), 1510(s), 1472(m), 1303(m), 1237(s), 1178(s), 1118(m), 1020(m), 947(m), 822(s), 742(m), 602(m), 556(m), 538(m) $cm^{-1}$.

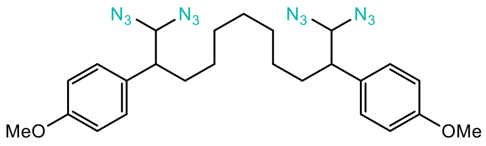


**4,4'-(1,1,11,11-Tetraazidoundecane-2,10-diyl)bis(methoxybenzene) (2ay)**

**1ay** (18.3 mg, 0.05 mmol) was utilized as substrate. Preparative thin layer chromatography (eluent: PE/EA = 10:1, v/v) to afford **2ay** as a yellow oil (15.8 mg, 59%). $R_f$ = 0.50 (PE/EA = 10:1, v/v). $^1$H NMR (400 MHz, $CDCl_3$) δ 7.11 (d, *J* = 8.4 Hz, 4H), 6.87 (d, *J* = 8.5 Hz, 4H), 4.74 (d, *J* = 7.1 Hz, 2H), 3.81 (s, 6H), 2.71 (td, *J* = 7.1, 3.6 Hz, 2H), 1.78 (ddd, *J* = 13.5, 7.0, 4.1 Hz, 2H), 1.67 – 1.55 (m, 2H), 1.22 – 1.02 (m, 10H). $^{13}$C NMR (101 MHz, $CDCl_3$) δ 159.09, 130.64, 129.66, 114.17, 81.95, 55.35, 49.88, 31.12, 29.38, 29.18, 26.99. HR-ESI-MS m/z calcd for $C_{25}H_{32}N_{12}O_2K$ $[(M+K)^+]$: 571.2408, found: 571.2408. IR: 2931(m), 2856(w), 2096(s), 1611(m), 1462(m), 1303(w), 1243(s), 1178(s), 1120(w), 1034(s), 919(s), 829(s), 736(w), 662(w), 547(m) $cm^{-1}$.

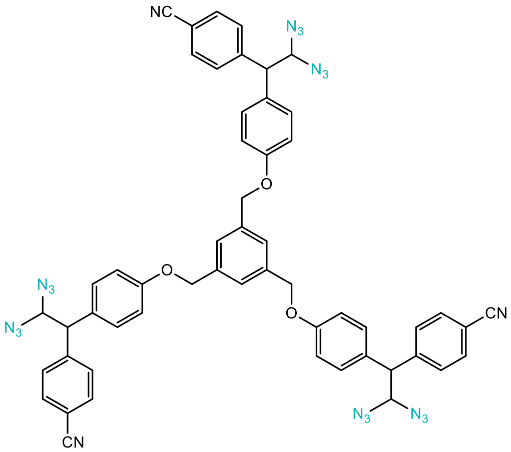


**4,4',4''-((((Benzene-1,3,5-triyltris(methylene))tris(oxy))tris(benzene-4,1-diyl))tris(2,2-diazidoethane-1,1-diyl))tribenzonitrile (2az)**

**1az** (25.9 mg, 0.0333 mmol) was utilized as substrate. Preparative thin layer chromatography (eluent: DCM) to afford **2az** as a yellow oil (29.0 mg, 85%). $R_f$ = 0.50 (DCM). $^1$H NMR (400 MHz, $CDCl_3$) δ 7.63 (d, *J* = 8.4 Hz, 6H), 7.43 (d, *J* = 1.9 Hz, 9H),

7.20 (d, *J* = 8.8 Hz, 6H), 6.95 (d, *J* = 8.7 Hz, 6H), 5.32 (d, *J* = 8.3 Hz, 3H), 5.05 (s, 6H), 4.17 (d, *J* = 8.3 Hz, 3H). $^{13}$C NMR (101 MHz, $CDCl_3$) δ 158.43, 144.64, 137.78, 132.64, 130.17, 129.80, 129.41, 126.20, 118.62, 115.43, 111.53, 79.61, 69.77, 55.11. HR-ESI-MS m/z calcd for $C_{54}H_{39}N_{21}O_3Na$ [(M+Na)$^+$]: 1052.3442, found: 1052.3448. IR: 2918(m), 2850(w), 2228(m), 2099(s), 1608(s), 1508(s), 1456(w), 1415(w), 1303(m), 1223(s), 1179(s), 1118(m), 1052(m), 1014(m), 949(m), 904(m), 824(s), 731(s), 628(m), 566(m), 537(m) cm$^{-1}$.

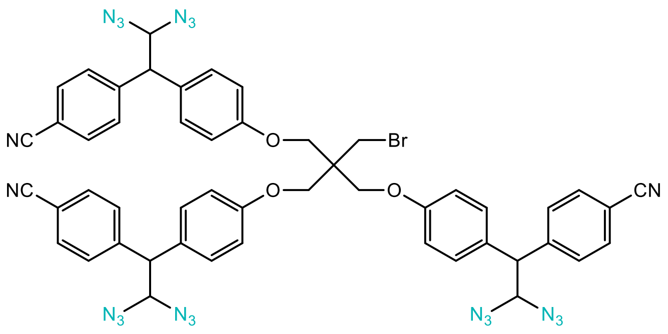


**Product 2ba**

**1ba** (27.0 mg, 0.0333 mmol) was utilized as substrate. Preparative thin layer chromatography (eluent: DCM) to afford **2ba** as a colorless oil (32.6 mg, 92%). $R_f$ = 0.50 (DCM). $^1$H NMR (400 MHz, $CDCl_3$) δ 7.62 (d, *J* = 6.9 Hz, 6H), 7.40 (d, *J* = 7.8 Hz, 6H), 7.17 (d, *J* = 7.9 Hz, 6H), 6.88 (d, *J* = 8.0 Hz, 6H), 5.31 (d, *J* = 8.6 Hz, 3H), 4.19 (s, 6H), 4.15 (d, *J* = 8.4 Hz, 3H), 3.83 (s, 2H). $^{13}$C NMR (101 MHz, $CDCl_3$) δ 158.20, 144.58, 132.65, 130.48, 129.75, 129.37, 118.56, 115.27, 111.58, 79.58, 77.48, 66.88, 55.06, 44.31, 33.74. HR-ESI-MS m/z calcd for $C_{50}H_{38}N_{21}O_3NaBr$ [(M+Na)$^+$]: 1082.2548, found: 1082.2554. IR: 2918(m), 2850(m), 2228(m), 2099(s), 1608(s), 1509(s), 1466(m), 1416(w), 1304(w), 1234(s), 1178(s), 1116(w), 1034(m), 950(w), 860(w), 824(s), 730(s), 628(m), 601(m), 557(s), 538(m) cm$^{-1}$.

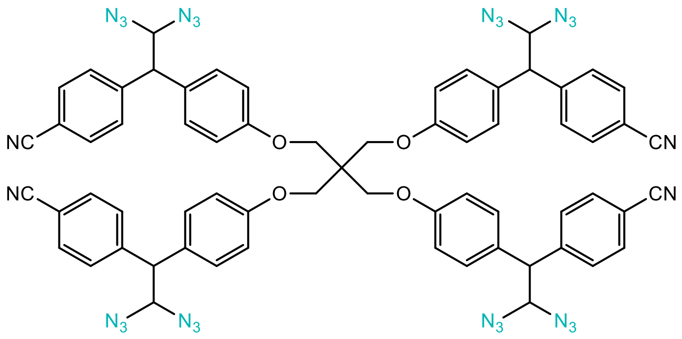


**Product 2bb**

**1bb** (23.8 mg, 0.025 mmol) was utilized as substrate. Preparative thin layer chromatography (eluent: DCM) to afford **2bb** as a yellow oil (22.9 mg, 71%). $R_f$ = 0.40 (DCM). $^1$H NMR (400 MHz, $CDCl_3$) δ 7.61 (d, *J* = 8.4 Hz, 8H), 7.39 (d, *J* = 8.3 Hz, 8H), 7.16 (d, *J* = 8.7 Hz, 8H), 6.86 (d, *J* = 8.7 Hz, 8H), 5.30 (d, *J* = 8.3 Hz, 4H), 4.27 (s, 8H), 4.14 (d, *J* = 8.3 Hz, 4H). $^{13}$C NMR (101 MHz, $CDCl_3$) δ 158.42, 144.62, 132.65, 130.36, 129.75, 129.37, 118.54, 115.24, 111.63, 79.62, 55.10, 44.70. HR-ESI-MS m/z calcd for $C_{65}H_{48}N_{28}O_4Na$ $[(M+Na)^+]$: 1307.4311, found: 1307.4312. IR: 2917(m), 2849(m), 2228(m), 2098(s), 1608(m), 1509(s), 1467(m), 1415(w), 1304(w), 1232(s), 1178(s), 1117(m), 1021(m), 949(m), 823(s), 738(m), 630(m), 559(m), 538(m) $cm^{-1}$.

## 4.3 Safety Evaluation of Geminal Diazides

### 4.3.1 $(N_C+N_O)/N_N$ Ratio

Organic azides are potentially explosive substances that can decompose with the slight input of energy from external sources (heat, light, pressure, etc). Generally, when designing the organic azides used for the project, the following equation can be kept in mind to evaluate the safety if the desired azide is stable enough:

$$(N_C + N_O)/N_N \geqslant 3 \qquad \text{(eq.1)}$$

In the above equation, *N* signifies the number of atoms. It is noted that this equation takes into account all nitrogen atoms on the organic azides, not just those on the azido group. The ratio $[(N_C+N_O)/N_N]$ of each obtained geminal diazides was calculated. It was found that the ratios of some geminal diazides we have obtained are less than 3. But these compounds can be stored at room temperature and can be handled as other compounds

without special care. Despite all this, one can't be too careful with these compounds.

**Supplementary Table 5** The ratios [($N_C$+$N_O$)/$N_N$] of geminal diazides

| Compound | Molecular Formula | $N_C$ | $N_O$ | $N_N$ | ($N_C$+$N_O$)/$N_N$ |
|---|---|---|---|---|---|
| **2a** | $C_{16}H_{13}N_7O$ | 16 | 1 | 7 | 2.429 |
| **2b** | $C_{16}H_{10}D_3N_7O$ | 16 | 1 | 7 | 2.429 |
| **2c** | $C_{17}H_{15}N_7O$ | 17 | 1 | 7 | 2.571 |
| **2d** | $C_{18}H_{17}N_7O$ | 18 | 1 | 7 | 2.714 |
| **2e** | $C_{22}H_{17}N_7O$ | 22 | 1 | 7 | 3.286 |
| **2f** | $C_{21}H_{15}N_7O$ | 21 | 1 | 7 | 3.143 |
| **2g** | $C_{31}H_{29}N_7OSi$ | 31 | 1 | 7 | 4.571 |
| **2h** | $C_{17}H_{15}N_7O_2$ | 17 | 2 | 7 | 2.714 |
| **2i** | $C_{16}H_{12}BrN_7O$ | 16 | 1 | 7 | 2.429 |
| **2j** | $C_{16}H_{12}ClN_7O$ | 16 | 1 | 7 | 2.429 |
| **2k** | $C_{18}H_{17}N_7O_3$ | 18 | 3 | 7 | 3.000 |
| **2l** | $C_{18}H_{17}N_7O$ | 18 | 1 | 7 | 2.714 |
| **2m** | $C_{17}H_{13}N_7O$ | 17 | 1 | 7 | 2.571 |
| **2n** | $C_{16}H_{13}N_7O$ | 16 | 1 | 7 | 2.429 |
| **2o** | $C_{17}H_{15}N_7O_2$ | 17 | 2 | 7 | 2.714 |
| **2p** | $C_{17}H_{15}N_7O_2$ | 17 | 2 | 7 | 2.714 |
| **2q** | $C_{20}H_{15}N_7O$ | 20 | 1 | 7 | 3.000 |
| **2r** | $C_{20}H_{15}N_7O$ | 20 | 1 | 7 | 3.000 |
| **2s** | $C_{24}H_{18}N_8O_2S$ | 24 | 2 | 8 | 3.250 |
| **2t** | $C_{24}H_{18}N_8O_2S$ | 24 | 2 | 8 | 3.250 |
| **2u** | $C_{13}H_9N_7S$ | 13 | 0 | 7 | 1.857 |
| **2v** | $C_{17}H_{15}N_7O$ | 17 | 1 | 7 | 2.571 |
| **2w** | $C_{25}H_{20}N_8O_2S$ | 25 | 2 | 8 | 3.375 |
| **2x** | $C_{13}H_8BrN_7S$ | 13 | 0 | 7 | 1.857 |
| **2y** | $C_{15}H_{14}N_6O$ | 15 | 1 | 6 | 2.667 |
| **2z** | $C_{16}H_{13}F_3N_6O$ | 16 | 1 | 6 | 2.833 |
| **2aa** | $C_{16}H_{14}F_2N_6O$ | 16 | 1 | 6 | 2.833 |
| **2ab** | $C_{15}H_{13}N_7O_3$ | 15 | 3 | 7 | 2.571 |
| **2ac** | $C_{17}H_{16}N_6O_3$ | 17 | 3 | 6 | 3.333 |
| **2ad** | $C_{16}H_{16}N_6O_3S$ | 16 | 3 | 6 | 3.167 |
| **2ae** | $C_{21}H_{25}BN_6O_3$ | 21 | 1 | 6 | 3.667 |

| | | | | | |
|---|---|---|---|---|---|
| **2af** | $C_{16}H_{13}F_3N_6O_4S$ | 16 | 4 | 6 | 3.333 |
| **2ag** | $C_{22}H_{20}N_6O_4S$ | 22 | 4 | 6 | 4.333 |
| **2ah** | $C_{16}H_{13}F_3N_6O_2$ | 16 | 2 | 6 | 3.000 |
| **2ai** | $C_{15}H_{13}BrN_6O$ | 15 | 1 | 6 | 2.667 |
| **2aj** | $C_{15}H_{13}IN_6O$ | 15 | 1 | 6 | 2.667 |
| **2ak** | $C_{16}H_{13}N_7O$ | 16 | 1 | 7 | 2.429 |
| **2al** | $C_{17}H_{16}N_6O_3$ | 17 | 3 | 6 | 3.333 |
| **2am** | $C_{16}H_{13}F_3N_6O_4S$ | 16 | 4 | 6 | 3.333 |
| **2an** | $C_{16}H_{13}N_7O$ | 16 | 1 | 7 | 2.429 |
| **2ao** | $C_{16}H_{13}F_3N_6O_4S$ | 16 | 4 | 6 | 3.333 |
| **2ap** | $C_{16}H_{12}FN_7O$ | 16 | 1 | 7 | 2.429 |
| **2aq** | $C_{17}H_{12}F_6N_6O$ | 17 | 1 | 6 | 3.000 |
| **2ar** | $C_{15}H_9F_5N_6O$ | 15 | 1 | 6 | 2.667 |
| **2as** | $C_{19}H_{16}N_6O$ | 19 | 1 | 6 | 3.333 |
| **2at** | $C_{10}H_{12}N_6O$ | 10 | 1 | 6 | 1.833 |
| **2au** | $C_{12}H_{16}N_6O$ | 12 | 1 | 6 | 2.167 |
| **2av** | $C_{13}H_{17}ClN_6O$ | 13 | 1 | 6 | 2.333 |
| **2aw** | $C_{19}H_{20}N_6O_2$ | 19 | 2 | 6 | 3.500 |
| **2ax** | $C_{35}H_{30}N_{14}O_2$ | 35 | 2 | 14 | 2.643 |
| **2ay** | $C_{25}H_{32}N_{12}O_2$ | 25 | 2 | 12 | 2.250 |
| **2az** | $C_{54}H_{39}N_{21}O_3$ | 54 | 3 | 21 | 2.714 |
| **2ba** | $C_{50}H_{38}BrN_{21}O_3$ | 50 | 3 | 21 | 2.524 |
| **2bb** | $C_{65}H_{48}N_{28}O_4$ | 65 | 4 | 28 | 2.464 |

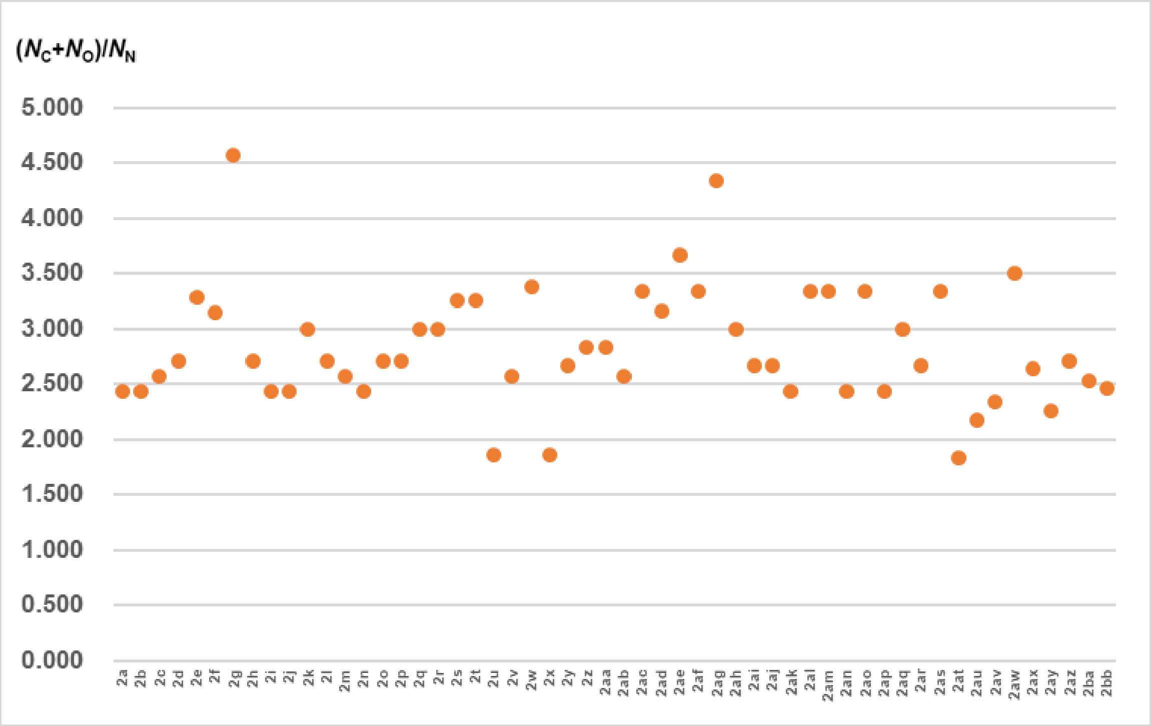


**Supplementary Figure 2.** The ratios [$(N_C+N_O)/N_N$] of geminal diazides

### 4.3.2 TGA-DSC Analysis

We studied the TGA-DSC spectrums of representative geminal diazides with relatively lower carbon to nitrogen ratios ($(N_C+N_O)/N_N$). It was found that most products were stable before 118 $^oC$ (except **2at**, 84 $^oC$), but gradually decomposed when the temperature was higher. These results revealed that the obtained geminal diazides are thermally stable.

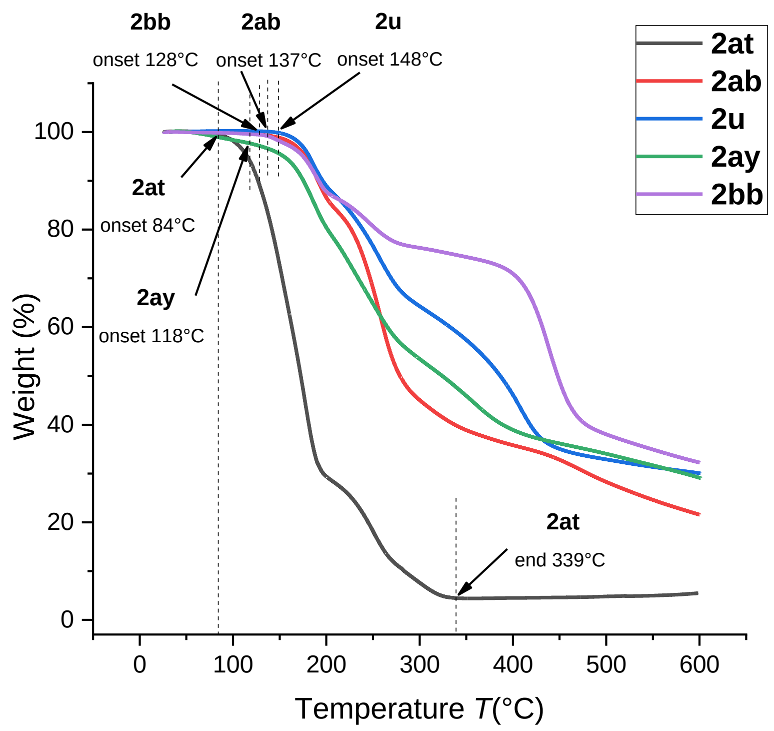


**Supplementary Figure 3.** TGA plots of **2at**, **2ab**, **2u**, **2ay** and **2bb** (heating rates:10 K·min$^{-1}$)

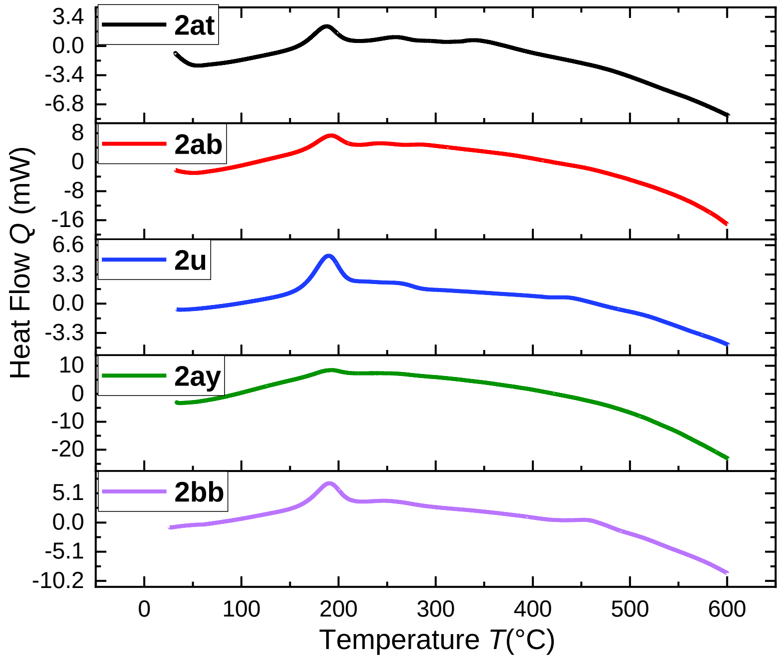


**Supplementary Figure 4.** DSC curves of **2at**, **2ab**, **2u**, **2ay** and **2bb** (heating rates:10 K·min$^{-1}$)

### 4.3.3 Impact Sensitivity Tests

Considering relatively faster decomposition rate of **2at**, the impact sensitivity of **2at** was measured by standard BAM fallhammer tests. It was found that the sample is insensitive to impact (IS > 80 J).

Impact: insensitive＞40 J, less sensitive⩾35 J, sensitive ⩾4 J, very sensitive ⩽3 J; according to the UN Recommendations on the Transport of Dangerous Goods, *Manual of Tests and Criteria*, United Nations Publication, New York, 5th edn, **2009**.

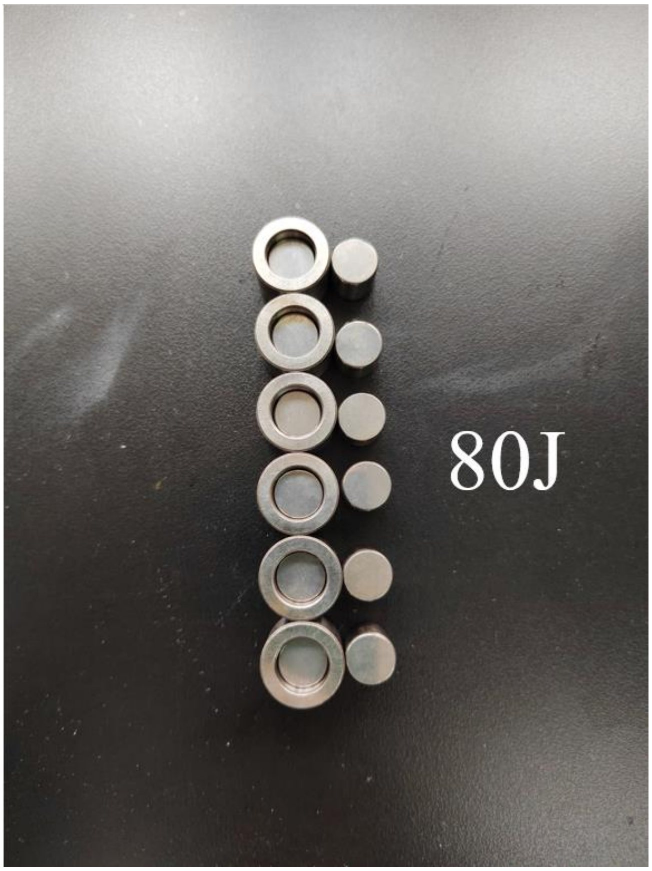


**Supplementary Figure 5.** Impact sensitivities of **2at**

## 4.4 Limitation of the Developed Method

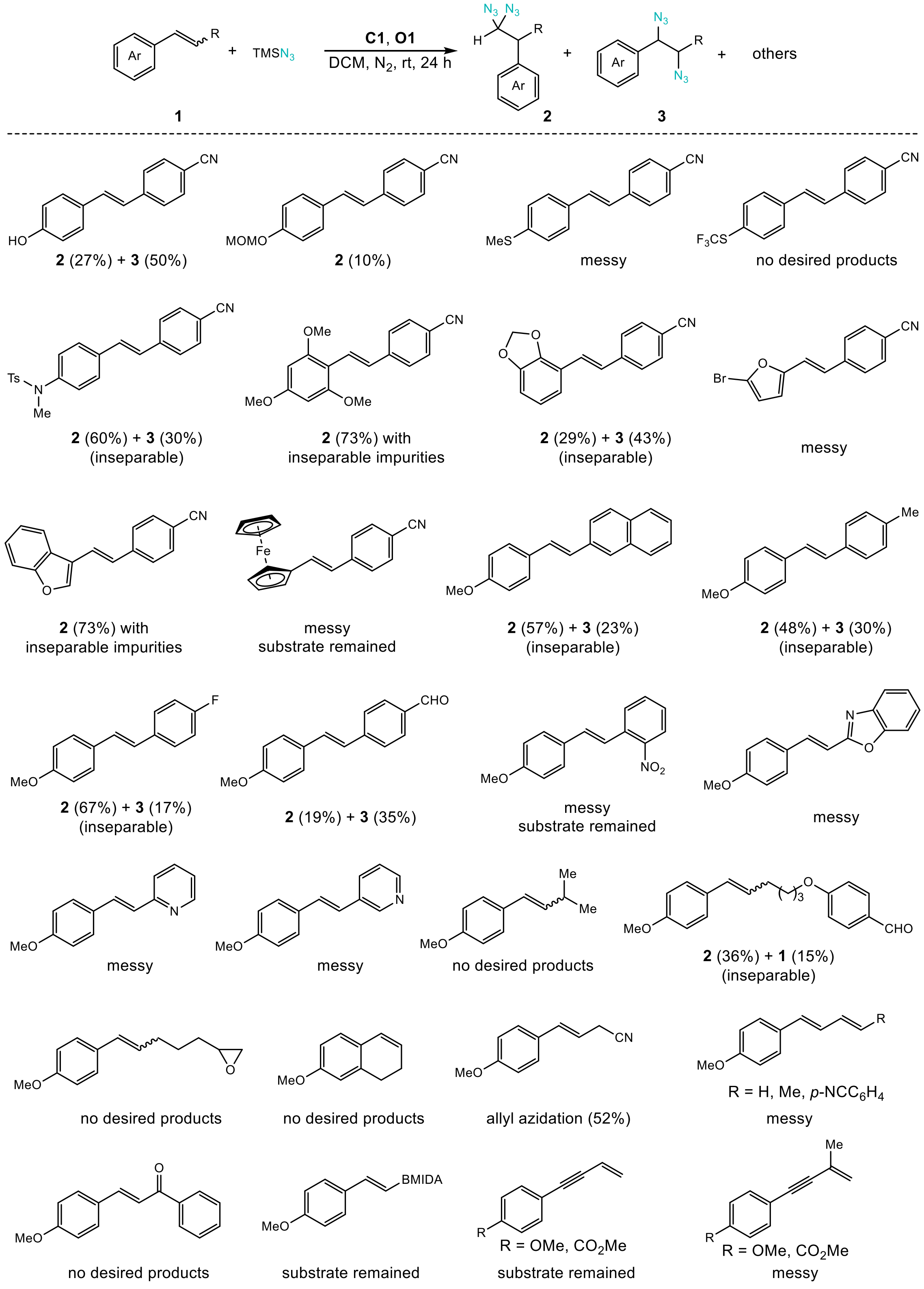


**Supplementary Figure 6.** Limitation of the developed method

## 5. Scale-up Synthesis and Further Transformations of the Products

### 5.1 Scale-up Synthesis

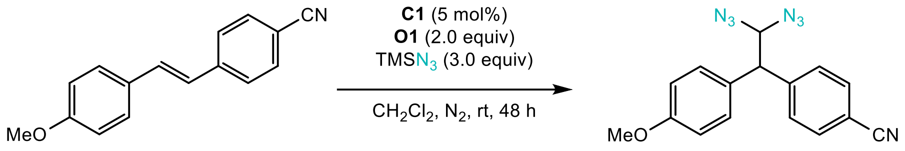


*E*-**1a**
1.06 g, 4.5 mmol

**2a**
95%, 1.33 g, 4.28 mmol

To a dry 50 mL Schlenk bottle equipped with a stir bar were added alkene *E*-**1a** (1.058 g, 4.5 mmol, 1.0 equiv), **O1** (2.043 g, 9.0 mmol, 2.0 equiv), and **C1** (70.2 mg, 0.225 mmol, 5 mol%) successively. Then the Schlenk bottle was transferred into glovebox. After dry $CH_2Cl_2$ (15.8 mL) and $TMSN_3$ (1.78 mL, 13.5 mmol, 3.0 equiv) were added successively under inert gas, the Schlenk bottle was capped and removed from glovebox. The reaction was carried out at room temperature for 48 h. Then the resulting mixture was quenched with diluted hydrochloric acid (10 mL, 1 M) and extracted with $CH_2Cl_2$ (20 mL × 3). The combined organic layers were washed with brine, dried over $Na_2SO_4$, and concentrated under reduced pressure. The residue was purified by flash column chromatography to give the desired geminal diazide **2a** (1.33 g, 95%).

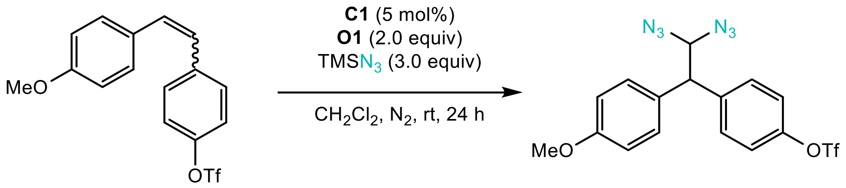


**1af**, *Z*/*E* = 3: 1
171 mg, 0.48 mmol

**2af**
75%, 160 mg, 0.36 mmol

To a dry 20 mL Schlenk bottle equipped with a stir bar were added alkene **1af** (171mg, 0.48 mmol, 1.0 equiv), **O1** (218 mg, 0.96 mmol, 2.0 equiv), and **C1** (7.7 mg, 0.024 mmol, 5 mol%) successively. Then the Schlenk bottle was transferred into glovebox. After dry $CH_2Cl_2$ (1.68 mL) and $TMSN_3$ (192 $\mu$L, 1.44 mmol, 3.0 equiv) were added successively under inert gas, the vial was capped and removed from glovebox. The reaction was carried out at room temperature for 24 h. Then the resulting mixture was quenched with diluted hydrochloric acid (5 mL, 1 M) and extracted with $CH_2Cl_2$ (10 mL × 3). The combined organic layers were washed with brine, dried over $Na_2SO_4$, and concentrated

under reduced pressure. The residue was purified by flash column chromatography to give the desired geminal diazide **2af** (160 mg, 75%).

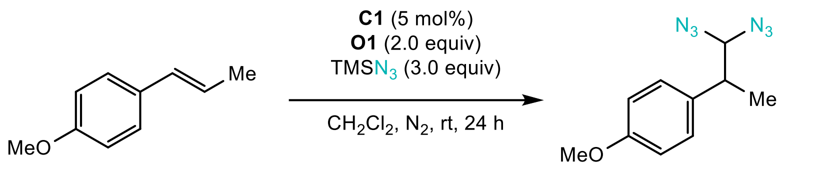


To a dry 20 mL Schlenk bottle equipped with a stir bar were added alkene **1at** (296 mg, 2.0 mmol, 1.0 equiv), **O1** (908 mg, 4.0 mmol, 2.0 equiv), and **C1** (31.2 mg, 0.01 mmol, 5 mol%) successively. Then the vial was transferred into glovebox. After dry $CH_2Cl_2$ (7.0 mL) and $TMSN_3$ (0.8 mL, 9.0 mmol, 3.0 equiv) were added successively under inert gas, the vial was capped and removed from glovebox. The reaction was carried out at room temperature for 24 h. Then the resulting mixture was quenched with diluted hydrochloric acid (10 mL, 1 M) and extracted with $CH_2Cl_2$ (20 mL × 3). The combined organic layers were washed with brine, dried over $Na_2SO_4$, and concentrated under reduced pressure. The residue was purified by flash column chromatography to give the desired geminal diazide **2at** (451 mg, 97%).

## 5.2 Further Transformations of the Products

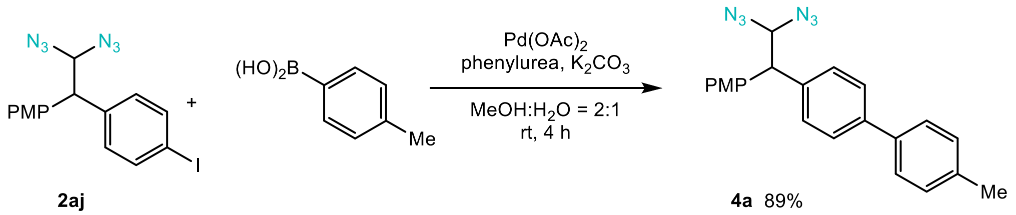


To a dry 10 mL Schlenk bottle equipped with a stir bar were added geminal diazide **2aj** (42 mg, 0.1 mmol, 1.0 equiv), $Pd(OAc)_2$ (2.2 mg, 0.01 mmol, 10 mol%), *p*-tolylboronic acid (20 mg, 0.15 mmol, 1.5 equiv), $K_2CO_3$ (27.6 mg, 0.2 mmol, 2.0 equiv) and phenylurea (2.7 mg, 0.02 mmol, 20 mol%) successively. Then the schlenk bottle was capped and pumped-refilled nitrogen three times. MeOH (1 mL) and $H_2O$ (0.5 mL) were added successively under inert gas. After the reaction was performed at room temperature

for 4 h, water (10 mL) was added. Then the resulting mixture was extracted with $CH_2Cl_2$ (5 mL × 3). The combined organic layers were washed with brine, dried over $Na_2SO_4$, and concentrated under reduced pressure. The residue was purified by preparative thin layer chromatography to give the desired cross coupling product **4a** (38.6 mg, 89%).

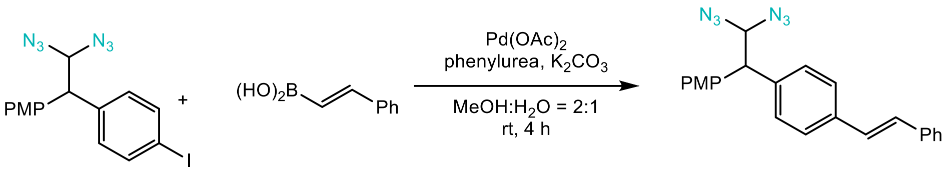


**2aj** **4b**, 97%

To a dry 10 mL Schlenk bottle equipped with a stir bar were added geminal diazide **2aj** (42 mg, 0.1 mmol, 1.0 equiv), $Pd(OAc)_2$ (2.2 mg, 0.01 mmol, 10 mol%), 2-phenylvinylboronic acid (22 mg, 0.15 mmol, 1.5 equiv), $K_2CO_3$ (27.6 mg 0.2 mmol, 2.0 equiv) and phenylurea (2.7 mg, 0.02 mmol, 20 mol%) successively. Then the Schlenk bottle was capped and pumped-refilled nitrogen three times. MeOH (1 mL) and $H_2O$ (0.5 mL) were added successively under inert gas. After the reaction was performed at room temperature for 4 h, water (10 mL) was added. Then the resulting mixture was extracted with $CH_2Cl_2$ (5 mL × 3). The combined organic layers were washed with brine, dried over $Na_2SO_4$, and concentrated under reduced pressure. The residue was purified by preparative thin layer chromatography to give the desired cross coupling product **4b** (39.7 mg, 97%).

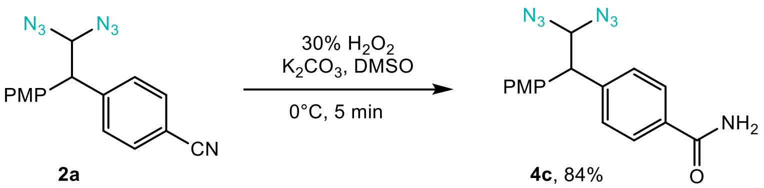


**2a** **4c**, 84%

To a solution of **2a** (31.9 mg, 0.1 mmol, 1.0 equiv) in DMSO (1 mL) at 0°C (ice bath) was added $K_2CO_3$ (27.6 mg 0.2 mmol, 2.0 equiv) and $H_2O_2$ (30%) (20 $\mu$L) successively. After the reaction was stirred at 0 °C (ice bath) for 5 min, water (10 mL) was added. The resulting mixture was extracted with EtOAc (5 mL × 3). The combined organic layers were washed with brine, dried over $Na_2SO_4$, and concentrated under reduced pressure. The residue was purified by preparative thin layer chromatography to give the desired

product **4c** (32.3 mg, 84%).

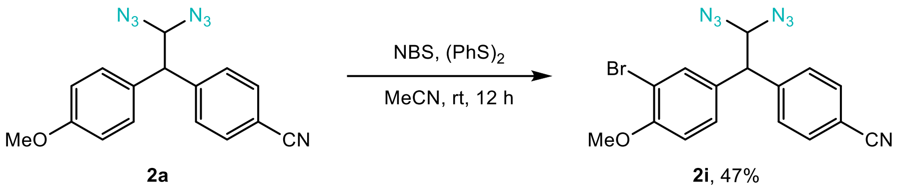


To a solution of **2a** (31.9 mg, 0.1 mmol, 1.0 equiv) in MeCN (0.5 mL) was added NBS (53.4 mg, 0.3 mmol, 3.0 equiv) and $(PhS)_2$ (6.5 mg, 0.03 mmol, 0.3 equiv) successively. After the reaction was stirred at room temperature overnight, water (10 mL) was added. The resulting mixture was extracted with EtOAc (5 mL × 3). The combined organic layers were washed with brine, dried over $Na_2SO_4$, and concentrated under reduced pressure. The residue was purified by preparative thin layer chromatography to give the desired product **2i** (18.8 mg, 47%).

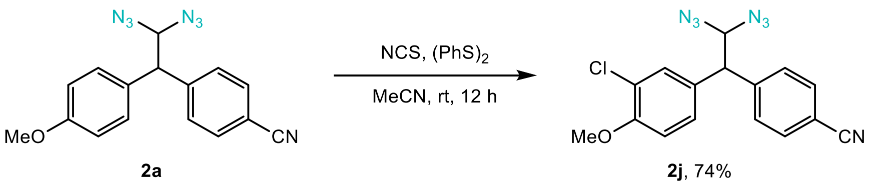


To a solution of **2a** (31.9 mg, 0.1 mmol, 1.0 equiv) in MeCN (0.5 mL) was added NCS (40.1 mg, 0.3 mmol, 3.0 equiv) and $(PhS)_2$ (6.5 mg, 0.03 mmol, 0.3 equiv) successively. After the reaction was stirred at room temperature overnight, water (10 mL) was added. The resulting mixture was extracted with EtOAc (5 mL × 3). The combined organic layers were washed with brine, dried over $Na_2SO_4$, and concentrated under reduced pressure. The residue was purified by preparative thin layer chromatography to give the desired product **2j** (26.3 mg, 74%).

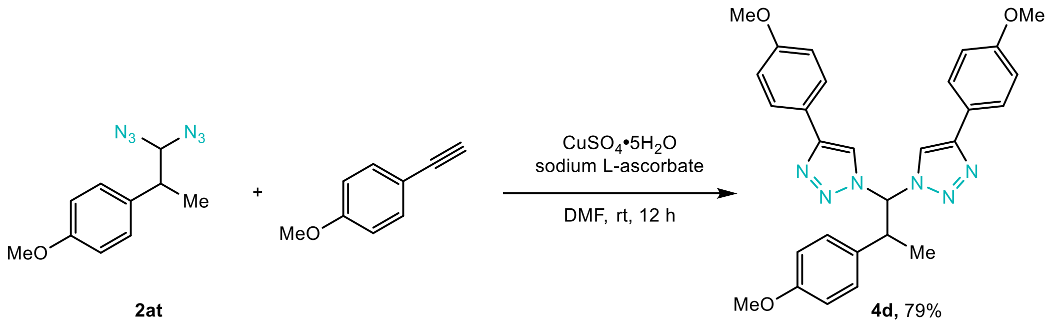


To a solution of **2at** (23.2 mg, 0.1 mmol, 1.0 equiv) in dry DMF (2.0 mL) was added 4-ethynylanisole (51.9 $\mu$L, 0.4 mmol, 4.0 equiv), sodium L-ascorbate (19.8 mg, 0.1 mmol, 1.0 equiv), and $CuSO_4 \cdot 5H_2O$ (2.5 mg, 0.01 mmol, 10 mol%) successively. After the reaction was stirred at room temperature for 12 h, water (10 mL) was added. The resulting mixture was extracted with EtOAc (15 mL × 3). The combined organic layers were washed with brine, dried over $Na_2SO_4$, and concentrated under reduced pressure. The residue was purified by preparative thin layer chromatography to give the desired product **4d** (39.4 mg, 79%).

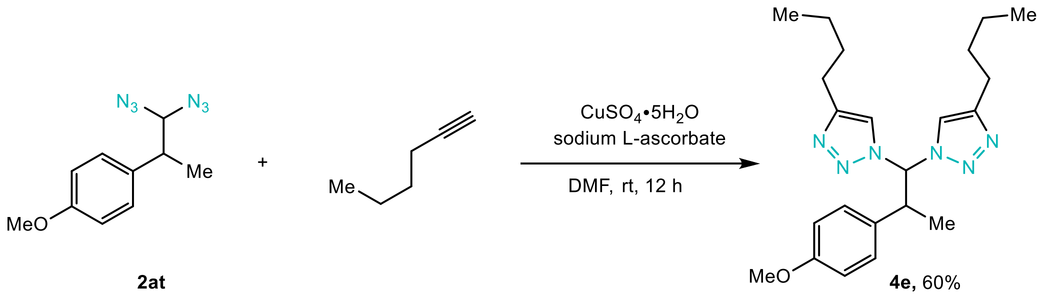


To a solution of **2at** (23.2 mg, 0.1 mmol, 1.0 equiv) in dry DMF (2.0 mL) was added 1-hexyne (44.6 $\mu$L, 0.4 mmol, 4.0 equiv), sodium L-ascorbate (19.8 mg, 0.1 mmol, 1.0 equiv), and $CuSO_4 \cdot 5H_2O$ (2.5 mg, 0.01 mmol, 10 mol%) successively. After the reaction was stirred at room temperature for 12 h, water (10 mL) was added. The resulting mixture was extracted with EtOAc (15 mL × 3). The combined organic layers were washed with brine, dried over $Na_2SO_4$, and concentrated under reduced pressure. The residue was purified by preparative thin layer chromatography to give the desired product **4e** (23.8 mg, 60%).

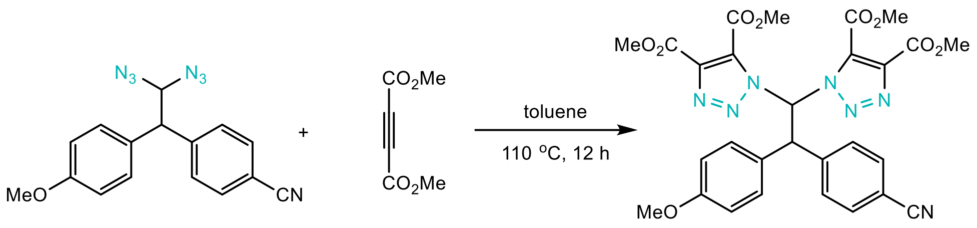


**2a** **4f**, 71%

To a solution of **2a** (31.9 mg, 0.1 mmol, 1.0 equiv) in toluene (3.2 mL) was added dimethyl acetylenedicarboxylate (73.8 $\mu$L, 0.6 mmol, 6.0 equiv). The reaction was stirred at 110 $^{o}$C overnight. After cooled to room temperature, the solvent was removed under reduced pressure. The residue was purified by preparative thin layer chromatography to give the desired product **4f** (42.8 mg, 71%).

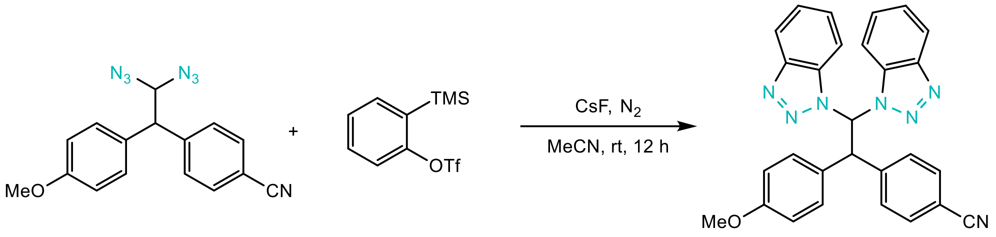


**2a** **4g**, 40%

To a solution of **2a** (31.9 mg, 0.1 mmol, 1.0 equiv) in dry MeCN (2.0 mL) was added 2-(trimethylsilyl)phenyl triflate (146 $\mu$L, 0.6 mmol, 6.0 equiv) and CsF (92 mg, 0.6 mmol, 6.0 equiv) under $N_2$ successively. After the reaction was stirred at room temperature for 12 h, water (10 mL) was added. The resulting mixture was extracted with EtOAc (15 mL × 3). The combined organic layers were washed with brine, dried over $Na_2SO_4$, and concentrated under reduced pressure. The residue was purified by preparative thin layer chromatography to give the desired product **4g** (18.9 mg, 40%).

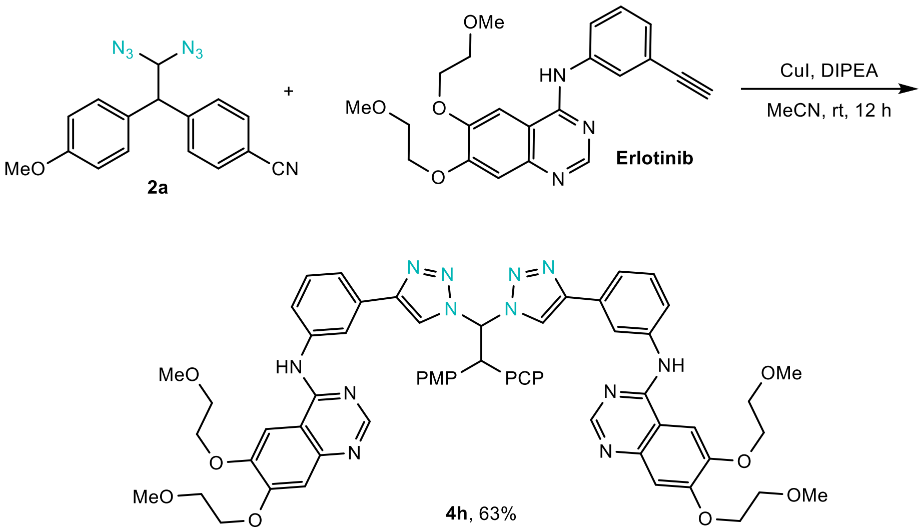


To a solution of **2a** (31.9 mg, 0.1 mmol, 1.0 equiv) and Erlotinib (86.46 mg, 0.22 mmol, 2.2 equiv) in MeCN (2 mL) was added CuI (3.8 mg, 0.02 mmol, 20 mol%) and DIPEA (3.2 $\mu$L, 0.02 mmol, 20 mol%) successively. After the reaction was stirred at room temperature for 12 h, water (10 mL) was added. The resulting mixture was concentrated under reduced pressure. The residue was purified by preparative thin layer chromatography to give the desired product **4h** (69.6 mg, 63%).

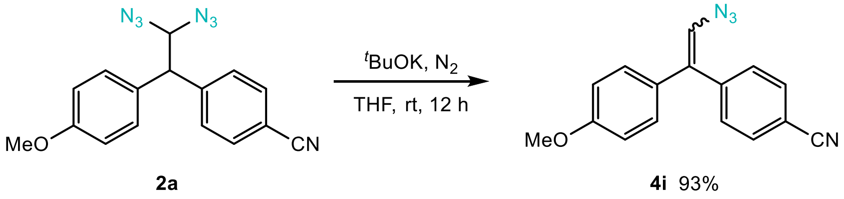


To a solution of **2a** (32 mg, 0.1 mmol, 1.0 equiv) in dry THF (500 $\mu$L) was added $^t$BuOK (22.4 mg 0.2mmol, 2.0 equiv) under $N_2$ atmosphere. After the reaction was stirred at room temperature overnight, water (5 mL) was added. The resulting mixture was extracted with EtOAc (5 mL × 3). The combined organic layers were washed with brine, dried over $Na_2SO_4$, and concentrated under reduced pressure. The residue was purified by preparative thin layer chromatography to give the desired product **4i** (25.7 mg, 93%).

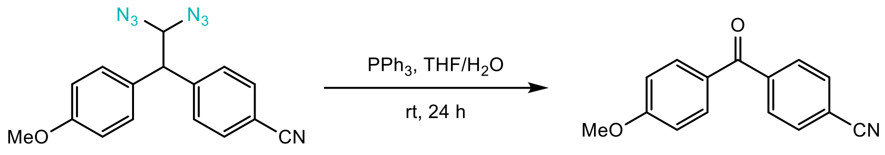


**2a** **4j**, 41%

To a solution of **2a** (32 mg, 0.1 mmol, 1.0 equiv) in THF/$H_2O$ (1.0 mL + 0.1 mL) was added $PPh_3$ (105 mg, 0.4 mmol, 4.0 equiv). After the reaction was stirred at room temperature for 24 h, water (5 mL) was added. The resulting mixture was extracted with EtOAc (15 mL × 3). The combined organic layers were washed with brine, dried over $Na_2SO_4$, and concentrated under reduced pressure. The residue was purified by preparative thin layer chromatography to give the desired product **4j** (9.7 mg, 41%).

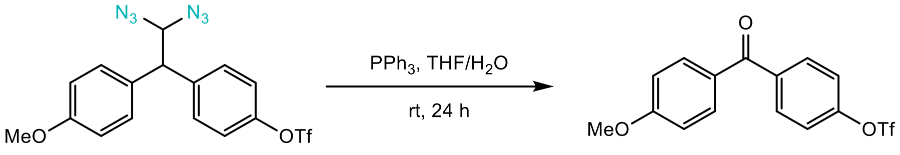


**2af** **4k**, 55%

To a solution of **2af** (44.2 mg, 0.1 mmol, 1.0 equiv) in THF/$H_2O$ (1.0 mL + 0.1 mL) was added $PPh_3$ (105 mg, 0.4 mmol, 4.0 equiv). After the reaction was stirred at room temperature for 24 h, water (5 mL) was added. The resulting mixture was extracted with EtOAc (15 mL × 3). The combined organic layers were washed with brine, dried over $Na_2SO_4$, and concentrated under reduced pressure. The residue was purified by preparative thin layer chromatography to give the desired product **4k** (19.9 mg, 55%).

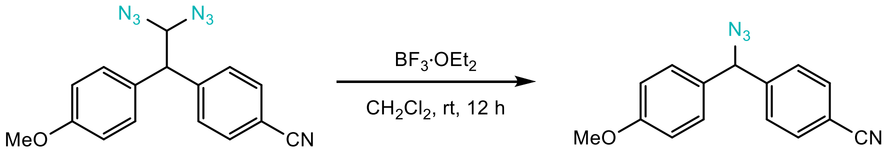


**2a** **4l**, 64%

To a solution of **2a** (32 mg, 0.1 mmol, 1.0 equiv) in $CH_2Cl_2$ (4.0 mL) was added $BF_3$ $OEt_2$ (12 $\mu$L, 0.1 mmol, 1.0 equiv). The reaction was stirred at room temperature for 12 h, and then quenched by $Et_3N$ (40 $\mu$L). The solvent was removed under reduced pressure. The residue was purified by preparative thin layer chromatography to give **4l** (16.9 mg, 64%).

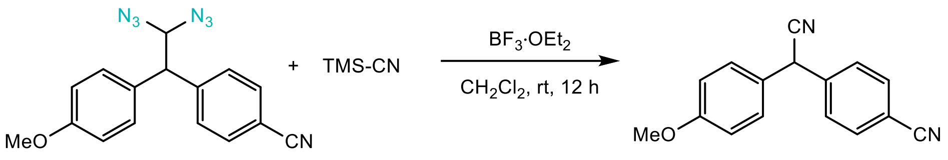


**2a** **4m**, 88%

To a solution of **2a** (32 mg, 0.1 mmol, 1.0 equiv) in $CH_2Cl_2$ (4.0 mL) were added TMSCN (99.2 $\mu$L, 0.8 mmol, 8.0 equiv) and $BF_3$ $OEt_2$ (60 $\mu$L, 0.5 mmol, 5.0 equiv) successively. The reaction was stirred at room temperature for 12 h, and then quenched by $Et_3N$ (70 $\mu$L). The solvent was removed under reduced pressure. The residue was purified by preparative thin layer chromatography to give **4m** (21.9 mg, 88%).

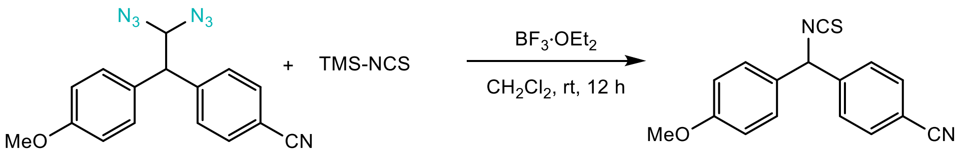


**2a** **4n**, 56%

To a solution of **2a** (32 mg, 0.1 mmol, 1.0 equiv) in $CH_2Cl_2$ (4.0 mL) were added TMSNCS (282 $\mu$L, 2.0 mmol, 20.0 equiv) and $BF_3$ $OEt_2$ (60 $\mu$L, 0.5 mmol, 5.0 equiv) successively. The reaction was stirred at room temperature for 12 h, and then quenched by $Et_3N$ (70 $\mu$L). The solvent was removed under reduced pressure. The residue was purified by preparative thin layer chromatography to give **4n** (15.6 mg, 56%).

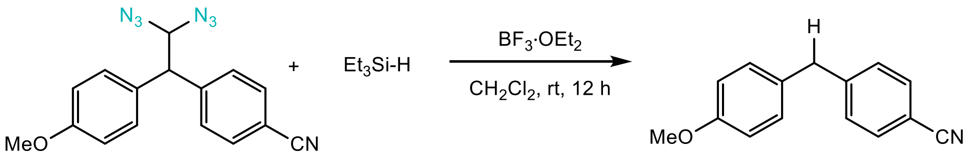


**2a** **4o**, 86%

To a solution of **2a** (32 mg, 0.1 mmol, 1.0 equiv) in $CH_2Cl_2$ (4.0 mL) were added $Et_3SiH$ (240 $\mu$L, 1.5 mmol, 15.0 equiv) and $BF_3$ $OEt_2$ (60 $\mu$L, 0.5 mmol, 5.0 equiv) successively. The reaction was stirred at room temperature for 12 h, and then quenched by $Et_3N$ (70 $\mu$L). The solvent was removed under reduced pressure. The residue was purified by preparative thin layer chromatography to give **4o** (19.3 mg, 86%).

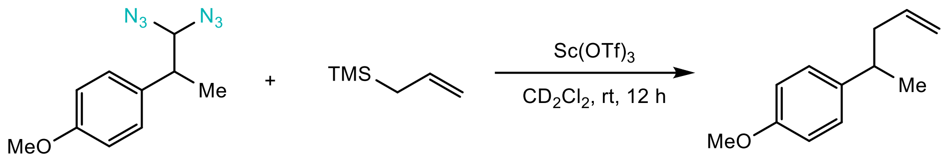


To a solution of **2at** (23.2 mg, 0.1 mmol, 1.0 equiv) in dry $CD_2Cl_2$ (500 $\mu$L) was added $Sc(OTf)_3$ (10 mg 0.01mmol, 0.2 equiv) and allyltrimethylsilane (48 $\mu$L, 0.3 mmol, 3.0 equiv) under $N_2$ atmosphere at 0°C. Then the reaction was stirred at room temperature overnight. The yield (79%) of **4p** was determined by using 1,1,2,2-tetrachloroethane as internal standard owing to the low boiling point of **4p**.

## 5.3 Characterization Data

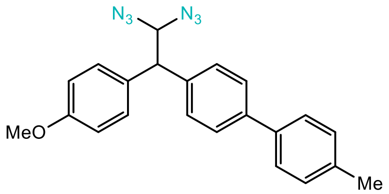


**4-(2,2-Diazido-1-(4-methoxyphenyl)ethyl)-4'-methyl-1,1'-biphenyl (4a)**

Preparative thin layer chromatography (eluent: PE/EA = 50:1, v/v) to afford **4a** as a yellow oil (38.6 mg, 89%). $R_f$ = 0.30 (PE/EA = 50:1, v/v). $^1$H NMR (400 MHz, $CDCl_3$) δ 7.55 (d, *J* = 8.3 Hz, 2H), 7.47 (d, *J* = 8.1 Hz, 2H), 7.36 (d, *J* = 8.3 Hz, 2H), 7.28 – 7.19 (m, 4H), 6.89 (d, *J* = 8.7 Hz, 2H), 5.35 (d, *J* = 8.8 Hz, 1H), 4.15 (d, *J* = 8.8 Hz, 1H), 3.80 (s, 3H), 2.39 (s, 3H). $^{13}$C NMR (101 MHz, $CDCl_3$) δ 159.06, 140.36, 138.18, 137.75, 137.32, 131.17, 129.63, 128.75, 127.44, 127.01, 114.38, 80.39, 55.38, 54.92, 21.24. HR-ESI-MS m/z calcd for $C_{22}H_{20}N_6ONa$ [$(M+Na)^+$]: 407.1596, found: 407.1598. IR: 3323(w), 3000(w), 2921(w), 2836(w), 2476(w), 2097(s), 1907(w), 1610(m), 1511(s), 1461(m), 1303(w), 1246(s), 1179(s), 1114(m), 1032(s), 1006(m), 947(m), 806(s), 773(m), 683(m), 583(m), 511(m) cm$^{-1}$.

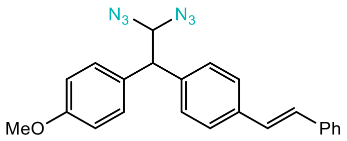


**(*E*)-1-(2,2-Diazido-1-(4-methoxyphenyl)ethyl)-4-styrylbenzene (4b)**

Preparative thin layer chromatography (eluent: PE/EA = 50:1, v/v) to afford **4b** as a yellow oil (39.7 mg, 97%). $R_f$ = 0.20 (PE/EA = 50:1, v/v). $^1$H NMR (400 MHz, $CDCl_3$) δ 7.58 – 7.47 (m, 4H), 7.37 (t, *J* = 7.5 Hz, 2H), 7.31 (d, *J* = 8.2 Hz, 2H), 7.29 – 7.24 (m, 3H), 7.10 (s, 2H), 6.90 (d, *J* = 8.7 Hz, 2H), 5.32 (d, *J* = 8.7 Hz, 1H), 4.14 (d, *J* = 8.7 Hz, 1H), 3.80 (s, 3H). $^{13}$C NMR (101 MHz, $CDCl_3$) δ 159.10, 138.81, 137.36, 136.71, 131.08, 129.63, 129.20, 128.82, 128.80, 128.14, 127.84, 127.00, 126.67, 114.39, 80.33, 55.37, 55.01. HR-ESI-MS m/z calcd for $C_{23}H_{20}N_6ONa$ [$(M+Na)^+$]: 419.1596, found: 419.1591. IR: 3325(w), 3027(w), 2934(w), 2836(w), 2482(w), 2097(s), 1609(m), 1510(s), 1448(m), 1303(m), 1246(s), 1178(s), 1117(m), 1031(s), 961(s), 800(m), 752(m), 691(s), 629(m), 577(s), 542(s) cm$^{-1}$.

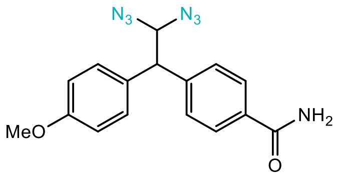


**4-(2,2-Diazido-1-(4-methoxyphenyl)ethyl)benzamide (4c)**

Preparative thin layer chromatography (eluent: PE/EA = 2:1, v/v) to afford **4c** as a colorless oil (32.3 mg, 84%). $R_f$ = 0.50 (PE/EA = 2:1, v/v). $^1$H NMR (400 MHz, $CDCl_3$) δ 7.78 (d, *J* = 8.3 Hz, 2H), 7.38 (d, *J* = 8.3 Hz, 2H), 7.21 (d, *J* = 8.7 Hz, 2H), 6.87 (d, *J* = 8.7 Hz, 2H), 6.24 (s, 2H), 5.33 (d, *J* = 8.7 Hz, 1H), 4.16 (d, *J* = 8.7 Hz, 1H), 3.78 (s, 3H). $^{13}$C NMR (101 MHz, $CDCl_3$) δ 169.22, 159.18, 143.60, 132.51, 130.32, 129.62, 128.74, 127.96, 114.44, 79.96, 55.36, 54.98. HR-ESI-MS m/z calcd for $C_{16}H_{15}N_7O_2Na$ [$(M+Na)^+$]: 360.1185, found: 360.1181. IR: 3346(s), 3192(s), 2837(w), 2098(s), 1655(s), 1610(s), 1566(m), 1511(s), 1462(m), 1385(m), 1303(m), 1243(s), 1179(s), 1123(m), 1030(m), 949(m), 822(m), 734(m), 707(w), 568(m), 534(m) cm$^{-1}$.

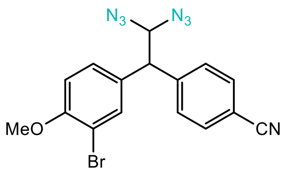


**4-(2,2-Diazido-1-(3-bromo-4-methoxyphenyl)ethyl)benzonitrile (2i)**

Preparative thin layer chromatography (eluent: PE/EA = 5:1, v/v) to afford **2i** as a

light-yellow oil (18.8 mg, 47%). $R_f$ = 0.40 (PE/EA = 5:1, v/v).

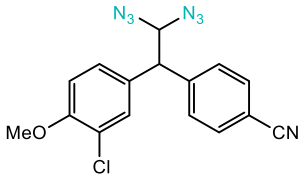


**4-(2,2-Diazido-1-(3-chloro-4-methoxyphenyl)ethyl)benzonitrile (2j)**

Preparative thin layer chromatography (eluent: PE/EA = 5:1, v/v) to afford **2j** as a light-yellow oil (26.3 mg, 74%). $R_f$ = 0.40 (PE/EA = 5:1, v/v).

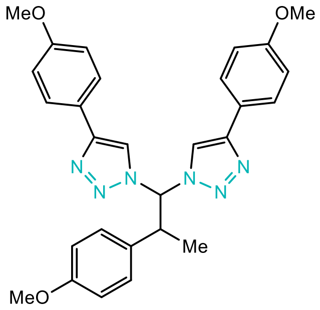


**1,1'-(2-(4-Methoxyphenyl)propane-1,1-diyl)bis(4-(4-methoxyphenyl)-1*H*-1,2,3-triazole) (4d)**

Preparative thin layer chromatography (eluent: PE/EA = 1:1, v/v) to afford **4d** as a white solid (39.4 mg, 79%). $R_f$ = 0.5 (PE/EA = 1:1, v/v). $^1$H NMR (400 MHz, $CDCl_3$) δ 8.35 – 8.28 (m, 1H), 7.84 – 7.74 (m, 3H), 7.62 (d, *J* = 8.7 Hz, 2H), 7.14 (d, *J* = 8.5 Hz, 2H), 7.07 – 6.99 (m, 1H), 6.96 (d, *J* = 8.6 Hz, 2H), 6.88 (d, *J* = 8.6 Hz, 2H), 6.73 (d, *J* = 8.4 Hz, 2H), 4.38 (dq, *J* = 13.9, 6.9 Hz, 1H), 3.83 (s, 3H), 3.79 (s, 3H), 3.68 (s, 3H), 1.28 (d, *J* = 6.9 Hz, 3H). $^{13}$C NMR (101 MHz, $CDCl_3$) δ 160.03, 159.89, 159.02, 148.77, 147.69, 131.12, 128.39, 127.28, 127.16, 122.57, 122.51, 119.17, 118.50, 118.45, 114.43, 114.30, 78.18, 55.43, 55.40, 55.22, 44.22, 18.36. HR-ESI-MS m/z calcd for $C_{28}H_{28}N_6O_3Na$ [$(M+Na)^+$]: 519.2121, found: 519.2120. IR: 3108(w), 2964(w), 2837(w), 1616(m), 1581(m), 1497(s), 1441(m), 1353(m), 1304(m), 1251(s), 1177(s), 1108(w), 1073(w), 1028(s), 975(w), 929(w), 822(s), 792(s), 613(m), 561(m), 534(s) cm$^{-1}$.

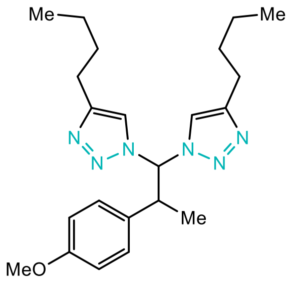


**1,1'-(2-(4-Methoxyphenyl)propane-1,1-diyl)bis(4-butyl-1*H*-1,2,3-triazole) (4e)**

Preparative thin layer chromatography (eluent: PE/EA = 2:1, v/v) to afford **4e** as a white solid (23.8 mg, 60%). $R_f$ = 0.5 (PE/EA = 2:1, v/v). $^1$H NMR (400 MHz, $CDCl_3$) δ 7.84 (s, 1H), 7.23 (s, 1H), 7.04 (d, *J* = 8.5 Hz, 2H), 6.77 (d, *J* = 11.3 Hz, 1H), 6.72 (d, *J* = 8.5 Hz, 2H), 4.20 (dq, *J* = 13.9, 6.9 Hz, 1H), 3.72 (s, 3H), 2.73 (t, *J* = 7.7 Hz, 2H), 2.53 (t, *J* = 7.5 Hz, 2H), 1.71 – 1.61 (m, 2H), 1.51 – 1.42 (m, 2H), 1.37 (dq, *J* = 14.5, 7.4 Hz, 2H), 1.22 – 1.12 (m, 4H), 0.92 (t, *J* = 7.3 Hz, 3H), 0.83 (t, *J* = 7.3 Hz, 3H). $^{13}$C NMR (101 MHz, $CDCl_3$) δ 158.98, 149.66, 148.29, 131.31, 128.31, 121.09, 120.37, 114.28, 77.95, 55.27, 44.46, 31.40, 31.25, 25.47, 25.07, 22.42, 22.05, 18.08, 13.93, 13.83. HR-ESI-MS m/z calcd for $C_{22}H_{32}N_6ONa$ [$(M+Na)^+$]: 419.2535, found: 419.2542. IR: 3126(m), 3083(w), 2956(m), 2926(s), 2858(m), 1615(m), 1553(w), 1517(s), 1456(s), 1436(s), 1341(w), 1287(s), 1251(s), 1178(s), 1153(m), 1110(m), 1046(s), 1036(s), 1003(m), 926(m), 842(s), 830(s), 810(s), 756(w), 731(m), 555(s), 526(w) cm$^{-1}$.

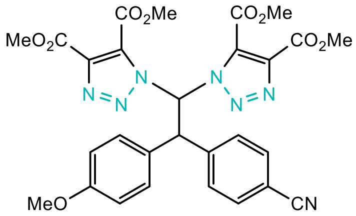


**Tetramethyl1,1'-(2-(4-cyanophenyl)-2-(4-methoxyphenyl)ethane-1,1-diyl)bis(1*H*-1,2,3-triazole-4,5-dicarboxylate) (4f)**

Preparative thin layer chromatography (eluent: PE/EA = 1:2, v/v) to afford **4f** as a yellow oil (42.8 mg, 71%). $R_f$ = 0.7 (PE/EA = 1:2, v/v). $^1$H NMR (400 MHz, $CDCl_3$) δ 8.52 (d, *J* = 11.7 Hz, 1H), 7.55 – 7.45 (m, 4H), 7.18 (d, *J* = 8.8 Hz, 2H), 6.74 (d, *J* = 8.8 Hz, 2H), 6.24 (d, *J* = 11.7 Hz, 1H), 4.02 (s, 3H), 3.98 (s, 3H), 3.90 (s, 3H), 3.90 (s, 3H), 3.71 (s, 3H). $^{13}$C NMR (101 MHz, $CDCl_3$) δ 159.78, 159.65, 159.60, 158.92, 158.78, 142.81,

139.81, 139.77, 132.85, 130.95, 130.59, 129.35, 128.96, 127.59, 118.25, 114.81, 112.01, 72.02, 55.34, 54.33, 54.17, 53.99, 53.02, 52.99. HR-ESI-MS m/z calcd for $C_{28}H_{25}N_7O_9Na$ [(M+Na)$^+$]: 626.1611, found: 626.1619. IR: 2957(w), 2848(w), 2227(m), 1727(s), 1609(m), 1566(m), 1512(s), 1452(s), 1347(m), 1327(m), 1225(s), 1180(s), 1157(s), 1068(m), 1039(m), 963(m), 894(w), 820(s), 770(m), 580(s), 527(m) cm$^{-1}$.

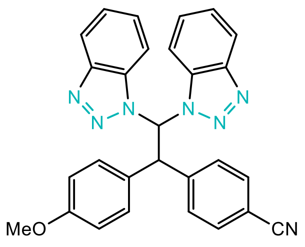


**4-(2,2-Bis(1*H*-benzo[*d*][1,2,3]triazol-1-yl)-1-(4-methoxyphenyl)ethyl)benzonitrile (4g)**

Preparative thin layer chromatography (eluent: PE/EA = 3:2, v/v) to afford **4g** as a yellow oil (18.9 mg, 40%). $R_f$ = 0.3 (PE/EA = 2:1, v/v). $^1$H NMR (400 MHz, $CDCl_3$) δ 8.31 (d, *J* = 12.0 Hz, 1H), 8.00 (d, *J* = 8.4 Hz, 2H), 7.93 (d, *J* = 8.4 Hz, 2H), 7.54 – 7.47 (m, 4H), 7.44 (d, *J* = 8.3 Hz, 2H), 7.33 (td, *J* = 7.8, 3.9 Hz, 2H), 7.23 (d, *J* = 8.6 Hz, 2H), 6.67 (d, *J* = 8.6 Hz, 2H), 6.46 (d, *J* = 12.0 Hz, 1H), 3.65 (s, 3H). $^{13}$C NMR (101 MHz, $CDCl_3$) δ 159.27, 146.34, 144.05, 132.80, 131.95, 131.91, 129.02, 128.86, 128.83, 128.47, 125.10, 124.93, 120.56, 120.49, 118.29, 114.70, 111.65, 110.10, 110.01, 73.27, 55.27, 51.98. HR-ESI-MS m/z calcd for $C_{28}H_{21}N_7ONa$ [(M+Na)$^+$]: 494.1705, found: 494.1706. IR: 2931(w), 2836(w), 2229(m), 1610(m), 1512(m), 1451(m), 1382(w), 1288(w), 1252(s), 1181(m), 1160(m), 1136(w), 1081(m), 1032(s), 933(m), 808(s), 765(s), 743(s), 573(s), 537(m), 429(m) cm$^{-1}$.

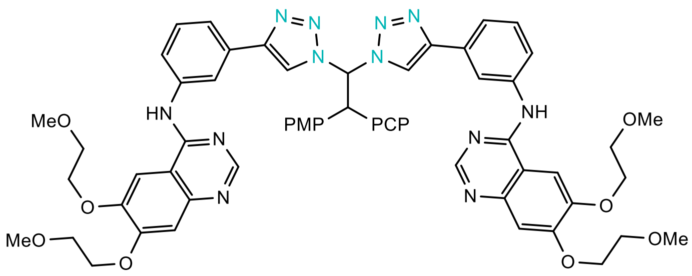


**Triazole (4h)**

Preparative thin layer chromatography (eluent: DCM/MeOH = 10:1, v/v) to afford **4h** as a white solid (69.6 mg, 63%). $R_f$ = 0.6 (DCM/MeOH = 10:1). $^{1}$H NMR (400 MHz, $CDCl_3$) δ 8.70 – 8.39 (m, 3H), 8.15 (s, 2H), 7.85 (s, 5H), 7.63 – 6.97 (m, 12H), 6.64 – 6.37 (m, 2H), 5.64 (s, 1H), 4.56 (s, 1H), 4.26 – 3.91 (m, 8H), 3.70 (s, 4H), 3.61 (s, 4H), 3.50 (s, 3H), 3.27 (m, 12H). $^{13}$C NMR (101 MHz, $CDCl_3$) δ 159.21, 156.69, 154.36, 153.28, 148.76, 148.15, 146.74, 143.32, 139.63, 132.73, 129.94, 129.84, 129.44, 128.76, 128.38, 128.19, 122.60, 121.18, 120.28, 119.16, 118.30, 114.69, 111.31, 109.39, 108.03, 102.87, 74.51, 70.65, 70.36, 68.80, 68.32, 59.11, 59.07, 55.06. HR-ESI-MS m/z calcd for $C_{60}H_{59}N_{13}O_9Na$ [(M+Na)$^+$]: 1128.4456, found: 1128.4456. IR: 3337(m), 3129(m), 2926(m), 2822(w), 2229(w), 1621(m), 1578(m), 1504(s), 1425(s), 1391(s), 1240(s), 1200(m), 1122(m), 1027(m), 925(m), 860(m), 786(m), 689(w), 580(m), 547(m) cm$^{-1}$.

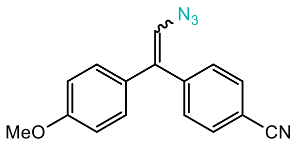


**4-(2-Azido-1-(4-methoxyphenyl)vinyl)benzonitrile (4i)**

Preparative thin layer chromatography (eluent: PE/EA = 10:1, v/v) to afford **4i** as a yellow oil (25.7 mg, 93%, the isomer ratio is 10:1). $R_f$ = 0.50 (PE/EA = 10:1, v/v). $^{1}$H NMR (400 MHz, $CDCl_3$) δ 7.57 (d, *J* = 8.4 Hz, 2H), 7.31 (d, *J* = 8.5 Hz, 2H), 7.20 (d, *J* = 8.7 Hz, 2H), 6.92 (d, *J* = 8.8 Hz, 2H), 6.75 (s, 1H), 3.84 (s, 3H). $^{13}$C NMR (101 MHz, $CDCl_3$) δ 159.53, 145.01, 132.30, 131.37, 130.11, 128.32, 128.08, 125.81, 118.96, 113.96, 110.91, 55.41. HR-ESI-MS m/z calcd for $C_{16}H_{12}N_4ONa$ [(M+Na)$^+$]: 299.0909, found: 299.0911. IR: 3346(m), 2917(w), 2836(w), 2224(m), 2101(s), 1601(s), 1508(s), 1456(m), 1402(m), 1283(m), 1245(s), 1176(s), 1112(m), 1028(s), 963(w), 829(s), 545(s) cm$^{-1}$.

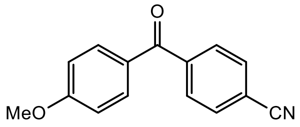


**4-(4-Methoxybenzoyl)benzonitrile (4j)**

Preparative thin layer chromatography (eluent: PE/EA = 3:1, v/v) to afford **4j** as a white solid (9.7 mg, 41%). $R_f$ = 0.3 (PE/EA = 5:1, v/v). Known compound[35]. $^{1}$H NMR (400

MHz, $CDCl_3$) δ 7.85 – 7.75 (m, 6H), 6.98 (d, *J* = 8.7 Hz, 2H), 3.90 (s, 3H). $^{13}$C NMR (101 MHz, $CDCl_3$) δ 193.87, 164.03, 142.23, 132.76, 132.23, 130.06, 129.07, 118.25, 115.28, 114.06, 55.74.

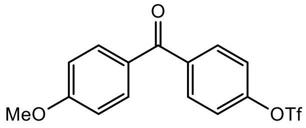


**4-(4-Methoxybenzoyl)phenyl trifluoromethanesulfonate (4k)**

Preparative thin layer chromatography (eluent: PE/EA = 8:1, v/v) to afford **4k** as a yellow oil (19.9 mg, 55%). $R_f$ = 0.3 (PE/EA = 10:1, v/v). $^{1}$H NMR (400 MHz, $CDCl_3$) δ 7.85 (d, *J* = 8.6 Hz, 2H), 7.81 (d, *J* = 8.7 Hz, 2H), 7.39 (d, *J* = 8.6 Hz, 2H), 6.98 (d, *J* = 8.7 Hz, 2H), 3.90 (s, 3H). $^{13}$C NMR (101 MHz, $CDCl_3$) δ 193.67, 163.83, 151.71, 138.49, 132.71, 131.93, 129.42, 121.44, 118.85 (q, *J* = 320.9 Hz), 113.97, 55.71. $^{19}$F NMR (376 MHz, $CDCl_3$) δ -72.72. HR-ESI-MS m/z calcd for $C_{15}H_{12}O_5F_3S$ [$(M+H)^+$]: 361.0358, found: 361.0353. IR: 3020(w), 2921(w), 2848(w), 1645(s), 1599(s), 1509(m), 1493(m), 1465(w), 1418(s), 1305(m), 1261(m), 1205(s), 1177(m), 1133(s), 1032(m), 1014(m), 966(w), 930(w), 881(s), 855(s), 841(s), 800(m), 769(s), 747(m), 681(m), 619(s), 572(m),524(m), 504(m) $cm^{-1}$.

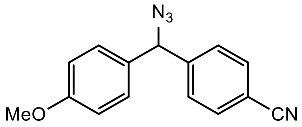


**4-(Azido(4-methoxyphenyl)methyl)benzonitrile (4l)**

Preparative thin layer chromatography (eluent: PE/EA = 5:1, v/v) to afford **4l** as a light-yellow oil (16.9 mg, 64%). $R_f$ = 0.5 (PE/EA = 5:1, v/v). $^{1}$H NMR (400 MHz, $CDCl_3$) δ 7.66 – 7.61 (m, 2H), 7.43 (d, *J* = 8.1 Hz, 2H), 7.20 – 7.14 (m, 2H), 6.93 – 6.88 (m, 2H), 5.71 (s, 1H), 3.81 (s, 3H). $^{13}$C NMR (101 MHz, $CDCl_3$) δ 159.94, 145.31, 132.53, 130.48, 129.08, 127.88, 118.64, 114.53, 111.80, 67.54, 55.44. HR-ESI-MS m/z calcd for $C_{15}H_{12}NO$ [$(M-N_3)^+$]: 222.0919, found: 222.0914. IR: 2934(w), 2838(w), 2229(m), 2097(s), 1609(s), 1510(s), 1462(w), 1303(w), 1246(s), 1174(s), 1112(m), 1031(s), 945(w), 827(s), 813(s), 759(m), 666(m), 626(m), 576(s), 538(s) $cm^{-1}$.

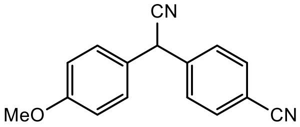


**4-(Cyano(4-methoxyphenyl)methyl)benzonitrile (4m)**

Preparative thin layer chromatography (eluent: PE/EA = 5:1, v/v) to afford **4m** as a light-yellow oil (21.9 mg, 88%). $R_f$ = 0.4 (PE/EA = 5:1, v/v). $^1$H NMR (600 MHz, $CDCl_3$) δ 7.67 (d, *J* = 8.4 Hz, 2H), 7.47 (d, *J* = 8.1 Hz, 2H), 7.22 (d, *J* = 8.7 Hz, 2H), 6.91 (d, *J* = 8.7 Hz, 2H), 5.14 (s, 1H), 3.80 (s, 3H). $^{13}$C NMR (151 MHz, $CDCl_3$) δ 159.99, 141.44, 133.07, 129.09, 128.55, 126.55, 118.90, 118.23, 115.01, 112.49, 55.52, 41.91. HR-ESI-MS m/z calcd for $C_{16}H_{12}N_2ONa$ [$(M+Na)^+$]: 271.0847, found: 271.0840. IR: 2931(w), 2839(w), 2230(m), 1610(m), 1510(s), 1462(m), 1413(m), 1305(w), 1251(s), 1179(s), 1113(m), 1030(s), 827(s), 810(s), 770(m), 591(m), 563(m) $cm^{-1}$.

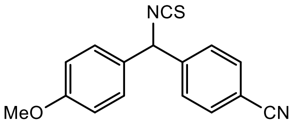


**4-(Isothiocyanato(4-methoxyphenyl)methyl)benzonitrile (4n)**

Preparative thin layer chromatography (eluent: PE/EA = 4:1, v/v) to afford **4n** as a light-yellow oil (15.6 mg, 56%). $R_f$ = 0.5 (PE/EA = 5:1, v/v). $^1$H NMR (400 MHz, $CDCl_3$) δ 7.66 (d, *J* = 7.2 Hz, 2H), 7.43 (d, *J* = 7.6 Hz, 2H), 7.19 (d, *J* = 7.3 Hz, 2H), 6.90 (d, *J* = 7.5 Hz, 2H), 5.99 (s, 1H), 3.81 (s, 3H). $^{13}$C NMR (101 MHz, $CDCl_3$) δ 160.04, 144.60, 136.84, 132.84, 130.57, 128.16, 127.35, 118.42, 114.70, 112.32, 63.88, 55.51. HR-ESI-MS m/z calcd for $C_{16}H_{13}N_2OS$ [$(M+H)^+$]: 281.0749, found: 281.0754. IR: 3493(w), 2933(w), 2837(w), 2229(m), 2050(s), 1725(w), 1608(m), 1510(s), 1460(w), 1408(w), 1303(m), 1250(s), 1175(s), 1112(w), 1030(s), 812(s), 764(s), 749(s), 701(m), 631(w), 577(s), 537(s), 483(w) $cm^{-1}$.

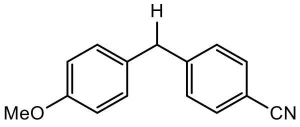


**4-(4-Methoxybenzyl)benzonitrile (4o)**

Preparative thin layer chromatography (eluent: PE/EA = 5:1, v/v) to afford **4o** as a

light-yellow oil (19.3 mg, 86%). $R_f$ = 0.65 (PE/EA = 5:1, v/v). Known compound[36]. $^1$H NMR (600 MHz, $CDCl_3$) δ 7.56 (d, *J* = 8.0 Hz, 2H), 7.27 (d, *J* = 8.1 Hz, 2H), 7.07 (d, *J* = 8.5 Hz, 2H), 6.85 (d, *J* = 8.6 Hz, 2H), 3.97 (s, 2H), 3.79 (s, 3H). $^{13}$C NMR (151 MHz, $CDCl_3$) δ 158.48, 147.37, 132.40, 131.51, 130.07, 129.64, 119.16, 114.27, 110.03, 55.39, 41.21.

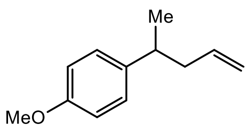


**1-Methoxy-4-(pent-4-en-2-yl)benzene (4p)**

Preparative thin layer chromatography (eluent: PE/EA = 100:1, v/v) to afford **4p** as a yellow oil (79% NMR yield). $R_f$ = 0.50 (PE/EA = 100:1, v/v). Known compound[37]. $^1$H NMR (400 MHz, $CDCl_3$) δ 7.12 (d, *J* = 8.7 Hz, 2H), 6.84 (d, *J* = 8.7 Hz, 2H), 5.78 – 5.64 (m, 1H), 5.04 – 4.91 (m, 2H), 3.79 (s, 3H), 2.75 (h, *J* = 7.0 Hz, 1H), 2.35 (dt, *J* = 13.6, 6.8 Hz, 1H), 2.32 – 2.20 (m, 1H), 1.23 (d, *J* = 7.0 Hz, 3H). $^{13}$C NMR (101 MHz, $CDCl_3$) δ 157.88, 139.32, 137.45, 127.97, 115.93, 113.80, 55.37, 43.00, 39.05, 21.86.

# 6. Mechanistic Studies

## 6.1 Competition between 1,1- *vs* 1,2-Diazidation of Stilbenes

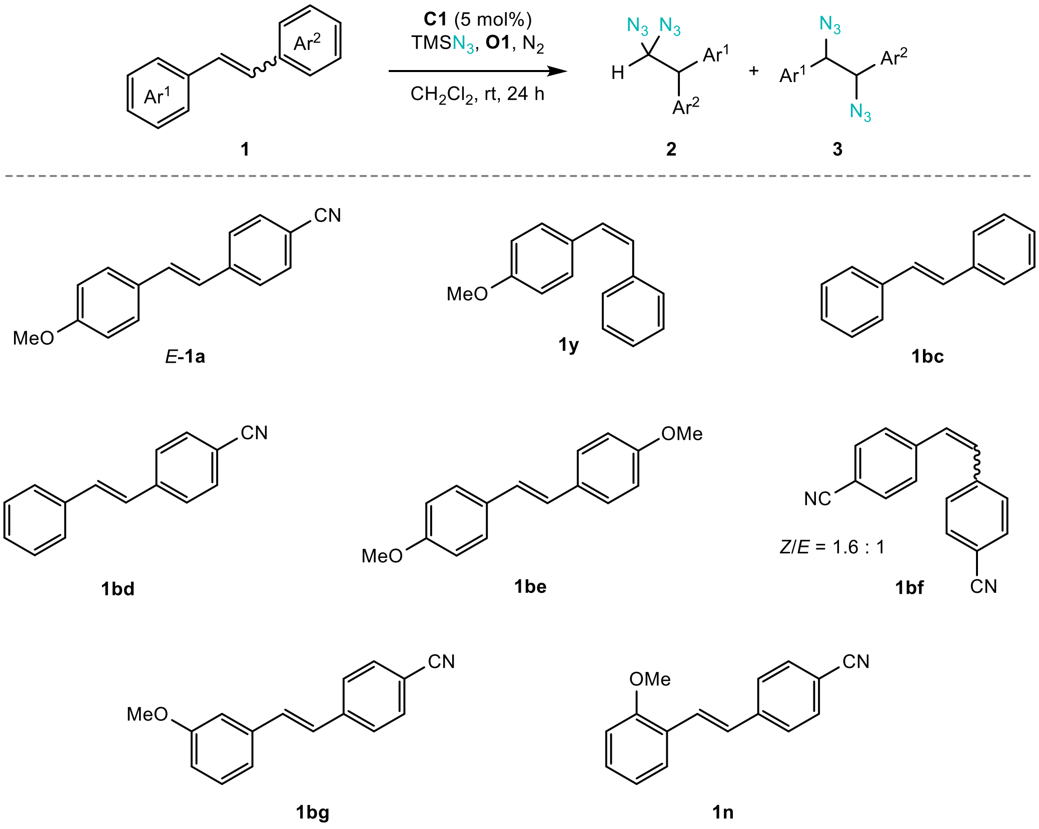


**Supplementary Figure 7.** Competition between 1,1- vs 1,2-diazidation of stilbenes

Alkene **1bc** is commercially available. Alkene **1bd** was prepared via Heck reaction of styrene and 4-bromobenzonitrile according to the literature procedure[38]. Alkene **1be** was prepared according to procedure **2.1.1**. Alkene **1bf** was prepared according to procedure **2.1.2**. Alkene **1bg** was prepared according to procedure **2.2**. Alkenes **1bd**[39], **1be**[40], **1bf**[41], **1bg**[42] are known compounds.

General procedure: To a dry 4 mL-vials separately equipped with a stir bar were added alkene shown as above (0.05 mmol, 1.0 equiv), **O1** (22.7 mg 0.1 mmol, 2.0 equiv), and **C1** (0.8 mg, 0.0025 mmol, 5 mol%) successively. Then the vial was transferred into glovebox. After dry $CH_2Cl_2$ (175 $\mu$L) and $TMSN_3$ (20.0 $\mu$L, 0.15 mmol, 3.0 equiv) were added successively under inert gas, the vial was capped and removed from glovebox. The reaction was performed at room temperature for 24 h. The solvent was removed under reduced pressure. The yield was determined by proton NMR using

1,1,2,2-tetrachloroethane as the internal standard. The reactions with each alkene were carried out according to the general procedure. The superposed NMR spectra were shown below:

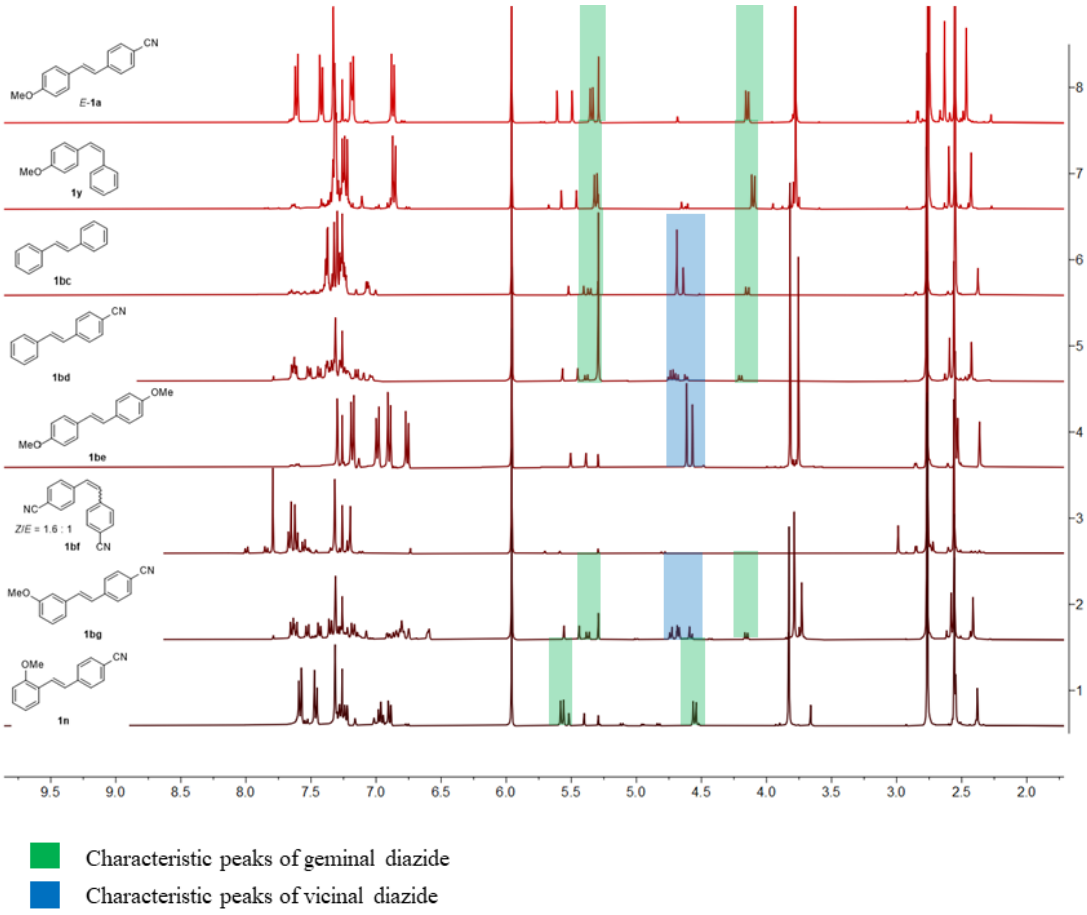


**Supplementary Figure 8.** The superposed NMR spectra of diazidation of stilbenes

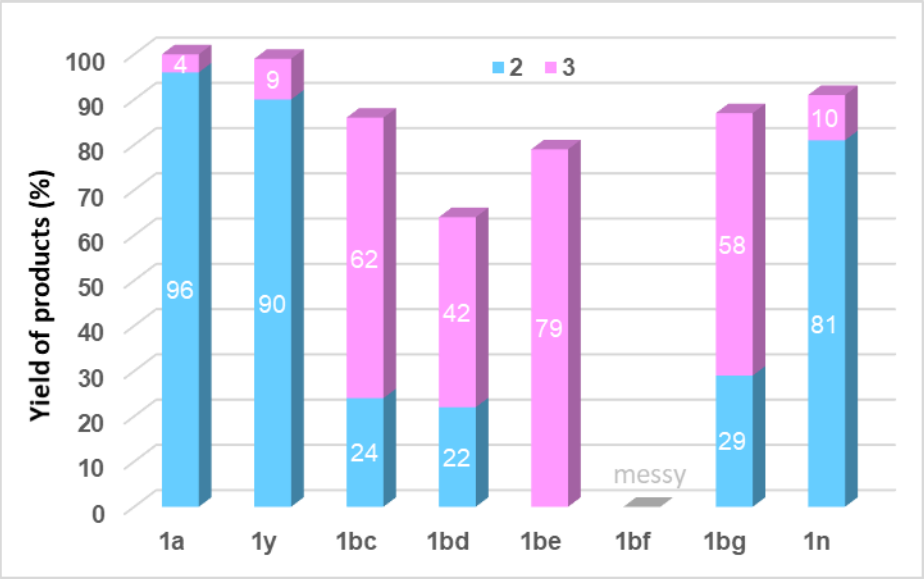


**Supplementary Figure 9.** NMR yield in diazidation of stilbenes

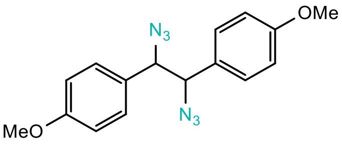


**1,2-Diazido-1,2-bis(4-methoxyphenyl)ethane (3be)**

Preparative thin layer chromatography (eluent: PE/EA = 10:1, v/v) to afford a major diastereoisomer as white solid (15.2 mg, 46%) and a minor diastereoisomer as light-yellow oil (7.5 mg, 24%). $R_f$ = 0.4 (PE/EA = 10:1, v/v). For the major diastereoisomer: $^1$H NMR (400 MHz, $CDCl_3$) δ 7.19 (d, $J$ = 8.7 Hz, 4H), 6.91 (d, $J$ = 8.7 Hz, 4H), 4.61 (s, 2H), 3.83 (s, 6H). $^{13}$C NMR (101 MHz, $CDCl_3$) δ 160.07, 129.35, 128.02, 114.20, 77.48, 77.16, 76.84, 69.37, 55.43. HR-ESI-MS m/z calcd for $C_{16}H_{16}N_6O_2Na$ [$(M+Na)^+$]: 347.1232, found: 347.1227. IR: 3321(w), 2961(w), 2909(w), 2837(w), 2479(w), 2176(w), 2090(s), 1609(s), 1584(m), 1511(s), 1462(m), 1304(m), 1235(s), 1175(s), 1110(s), 1027(s), 930(w), 894(w), 854(w), 824(s), 769(s), 677(m), 538(s) cm$^{-1}$.

## 6.2 Isotope Labeling Experiments

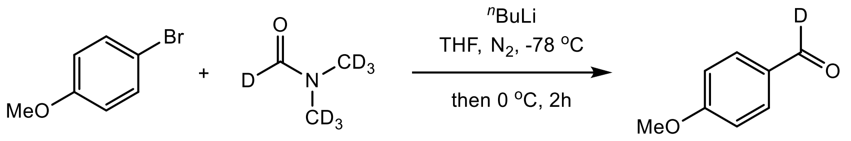


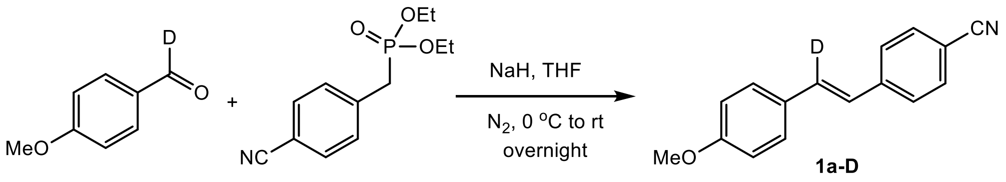


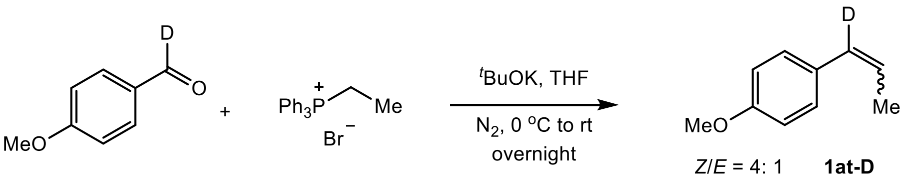


Deuterated stilbene **1a-D** was prepared via Horner-Wadsworth-Emmons reaction of deuterated *p*-anisaldehyde with diethyl (4-cyanobenzyl)phosphonate using NaH as base according to procedure **2.2**. Deuterated styrene **1at-D** was prepared via Wittig reaction of deuterated *p*-anisaldehyde with ethyltriphenylphosphonium bromide using $^t$BuOK as base according to procedure **2.1.3**. Deuterated *p*-anisaldehyde was prepared according to the literature procedure[43]. Deuterated *p*-anisaldehyde[43] and deuterated styrene **1at-D**[44] are known compounds.

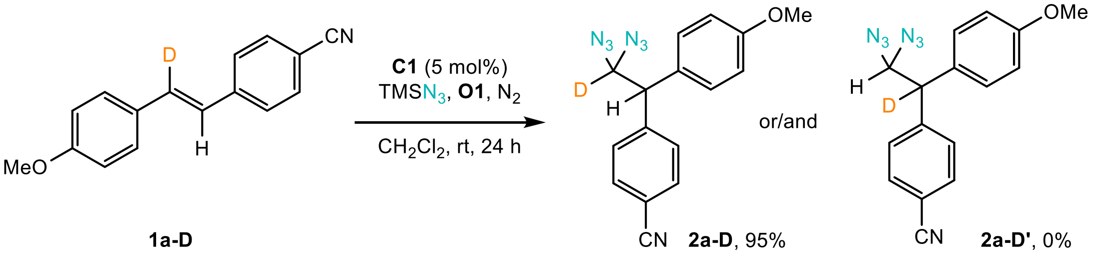


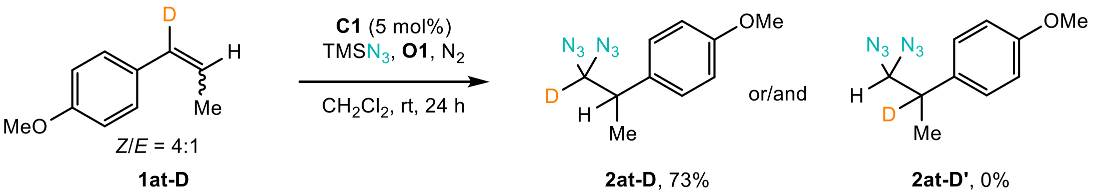

To a dry 4 mL vial equipped with a stir bar were added alkene **1a-D or 1at-D** (0.1 mmol, 1.0 equiv), **O1** (45.4 mg 0.2 mmol, 2.0 equiv), and **C1** (1.6 mg, 0.005 mmol, 5 mol%) successively. Then the vial was transferred into glovebox. After dry $CH_2Cl_2$ (350 $\mu$L) and $TMSN_3$ (39.5 $\mu$L, 0.3 mmol, 3.0 equiv) were added successively under inert gas, the vial was capped and removed from glovebox. The reaction was performed at room temperature for 24 h. Then the resulting mixture was quenched with diluted hydrochloric acid (1 mL, 1 M) and extracted with $CH_2Cl_2$ (3 mL × 3). The combined organic layers were washed with brine, dried over $Na_2SO_4$, and concentrated under reduced pressure. The residue was purified by preparative thin layer chromatography to give the desired geminal diazides.

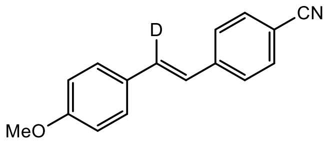


**(*E*)-4-(2-(4-Methoxyphenyl)vinyl-2-*d*)benzonitrile (1a-D)**

Prepared according to procedure **2.2**. Flash column chromatography on silica gel (eluent: PE/EA = 5:1 to 3:1, v/v) to afford **1a-D** as a white solid (173.1 mg, 73%, 1 mmol scale). $R_f$ = 0.3 (PE/EA = 5:1, v/v). $^1$H NMR (600 MHz, $CDCl_3$) δ 7.61 (d, *J* = 8.5 Hz, 2H), 7.55 (d, *J* = 8.1 Hz, 2H), 7.50 – 7.46 (m, 2H), 6.95 (s, 1H), 6.93 – 6.90 (m, 2H), 3.84 (s, 3H). $^{13}$C NMR (151 MHz, $CDCl_3$) δ 160.23, 142.37, 132.61, 129.15, 128.40, 126.70, 124.58, 119.31, 114.45, 110.18, 55.51. HR-ESI-MS m/z calcd for $C_{16}H_{12}DNONa$ [$(M+Na)^+$]: 259.0952, found: 259.0948.

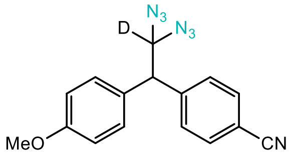


**4-(2,2-Diazido-1-(4-methoxyphenyl)ethyl-2-*d*)benzonitrile (2a-D)**

Preparative thin layer chromatography (eluent: PE/EA = 8:1, v/v) to afford **2a-D** as a light-yellow oil (30.3 mg, 95%). $R_f$ = 0.45 (PE/EA = 8:1, v/v). $^1$H NMR (400 MHz, $CDCl_3$) δ 7.62 (d, *J* = 8.1 Hz, 2H), 7.42 (d, *J* = 8.1 Hz, 2H), 7.19 (d, *J* = 8.7 Hz, 2H), 6.89 (d, *J* = 8.3 Hz, 2H), 4.16 (s, 1H), 3.79 (s, 3H). $^{13}$C NMR (101 MHz, $CDCl_3$) δ 159.36,

144.74, 132.60, 129.63, 129.38, 118.64, 114.57, 114.55, 111.42, 55.39, 54.96. HR-ESI-MS m/z calcd for $C_{16}H_{12}DN_7ONa$ [$(M+Na)^+$]: 343.1142, found: 343.1141. IR: 3324(w), 3004(w), 2935(w), 2838(w), 2454(w), 2228(m), 2099(s), 1610(m), 1511(s), 1462(m), 1415(m), 1245(s), 1179(s), 1114(s), 1030(s), 971(m), 919(w), 817(s), 774(w), 756(w), 701(w), 629(w), 578(s), 537(s) $cm^{-1}$.

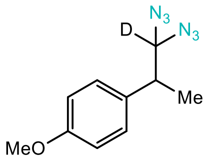


**1-(1,1-Diazidopropan-2-yl-1-*d*)-4-methoxybenzene (2at-D)**

Preparative thin layer chromatography (eluent: PE/EA = 50:1, v/v) to afford **2at-D** as a light-yellow oil (16.9 mg, 73%). $R_f$ = 0.50 (PE/EA = 50:1, v/v). $^1H$ NMR (400 MHz, $CDCl_3$) δ 7.17 (d, *J* = 7.8 Hz, 2H), 6.89 (d, *J* = 8.6 Hz, 2H), 3.81 (s, 3H), 2.95 (q, *J* = 7.0 Hz, 1H), 1.36 (d, *J* = 7.1 Hz, 3H). $^{13}C$ NMR (101 MHz, $CDCl_3$) δ 159.07, 132.47, 129.04, 114.18, 55.38, 43.92, 17.08. HR-ESI-MS m/z calcd for $C_{10}H_{11}DN_6ONa$ [$(M+Na)^+$]: 256.1033, found: 256.1040. IR: 3323(w), 2971(w), 2837(w), 2095(s), 1612(m), 1513(s), 1461(m), 1377(w), 1304(w), 1245(s), 1180(s), 1123(m), 1090(m), 1034(s), 985(m), 829(m), 745(w), 548(m) $cm^{-1}$.

## 6.3 Diazidation *vs* Carbon Deletion Reaction of Trisubstituted Alkenes

### 6.3.1 Preparation of Substrates

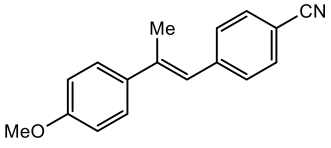


**1v**

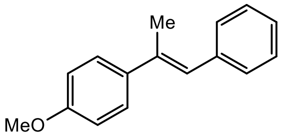


**1bh**

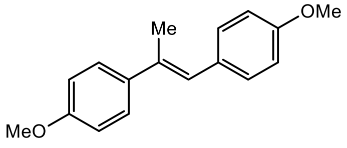


**1bi**

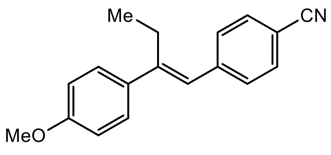


**1bj**

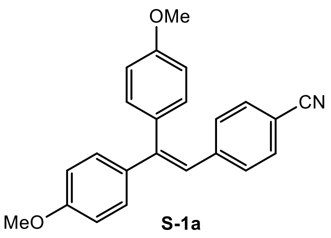


**S-1a**

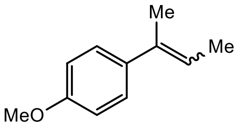


**S-1b**

**Supplementary Figure 11.** Trisubstituted alkene substrates used in reactions

Alkenes **1bh** and **S-1b** were prepared according to procedure **2.1.3**. Alkene **1bj** and **S-1a** were prepared according to procedure **2.2**. Alkenes **1bh**[45], **S-1a**[46], **S-1b**[47] are known compounds.

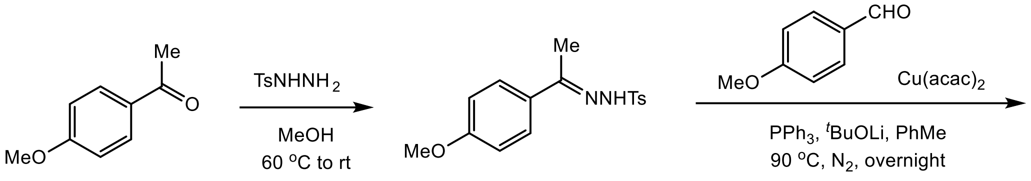


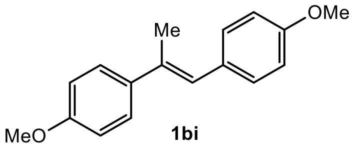


**1bi**

Alkenes **1bi** was prepared via copper-catalyzed cross coupling reaction of sulfonylhydrazone and *p*-anisaldehyde according to the literature procedure[48]. Alkene **1bi**[49] is a known compound.

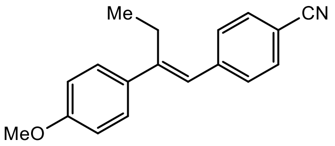


**(*E*)-4-(2-(4-Methoxyphenyl)but-1-en-1-yl)benzonitrile (1bj)**

Flash column chromatography on silica gel (eluent: PE/EA = 30:1 to 20:1, v/v) to afford **1bj** as a light-yellow oil (208 mg, 79%, 1 mmol scale). $R_f$ = 0.45 (PE/EA = 10:1, v/v). $^1$H NMR (400 MHz, $CDCl_3$) δ 7.36 (d, *J* = 8.5 Hz, 2H), 7.05 – 6.98 (m, 4H), 6.87 – 6.82 (m, 2H), 6.38 (s, 1H), 3.82 (s, 3H), 2.51 (qd, *J* = 7.4, 1.4 Hz, 2H), 1.07 (t, *J* = 7.4 Hz, 3H). $^{13}$C NMR (101 MHz, $CDCl_3$) δ 159.16, 148.86, 142.91, 132.63, 131.74, 129.57 (2C), 123.53, 119.32, 114.30, 109.22, 55.35, 33.71, 12.88. HR-ESI-MS m/z calcd for $C_{18}H_{17}NONa$ [$(M+Na)^+$]: 286.1208, found: 286.1205.

**6.3.2 Catalytic Azidation of Trisubstituted Alkenes**

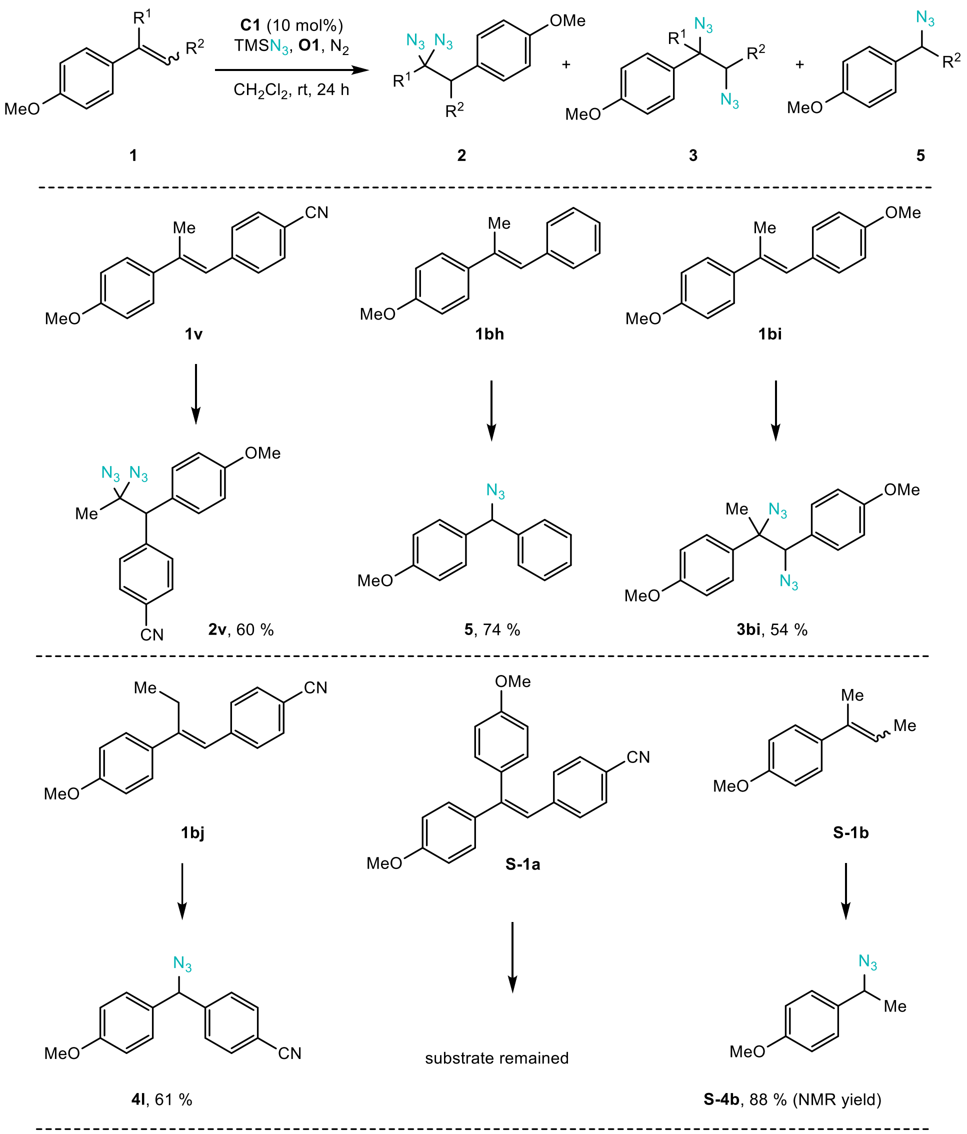


**Supplementary Figure 12.** Result of azidation of trisubstituted alkenes

To a dry 4 mL vial equipped with a stir bar were added trisubstituted alkene **1** (0.1 mmol, 1.0 equiv), **O1** (27.2 mg 0.12 mmol, 1.2 equiv), and **C1** (3.2 mg, 0.01 mmol, 10 mol%) successively. Then the vial was transferred into glovebox. After dry $CH_2Cl_2$ (700 $\mu$L) and $TMSN_3$ (39.5 $\mu$L, 0.3 mmol, 3.0 equiv) were added successively under inert gas, the vial was capped and removed from glovebox. The reaction was performed at room temperature for 24 h. Then the resulting mixture was quenched with diluted hydrochloric acid (1 mL, 1 M) and extracted with $CH_2Cl_2$ (5 mL × 3). The combined organic layers were washed with brine, dried over $Na_2SO_4$, and concentrated under reduced pressure. The residue was purified by preparative thin layer chromatography to give the

corresponding products. Reactions of **S-1a** and **S-1b** were preformed in 0.05 mmol scale, and the yield of **S-4b** (known compound[50]) was determined by proton NMR using 1,1,2,2-tetrachloroethane as the internal standard.

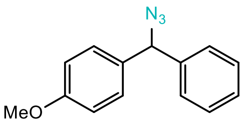


**1-(Azido(phenyl)methyl)-4-methoxybenzene (5)**

Preparative thin layer chromatography (eluent: PE/EA = 50:1, v/v) to afford **5** as a light-yellow oil (17.7 mg, 74%). $R_f$ = 0.50 (PE/EA = 50:1, v/v). Known compound[51]. $^{1}$H NMR (400 MHz, $CDCl_3$) δ 7.40 – 7.28 (m, 5H), 7.23 (d, *J* = 8.7 Hz, 2H), 6.89 (d, *J* = 8.7 Hz, 2H), 5.68 (s, 1H), 3.80 (s, 3H). $^{13}$C NMR (101 MHz, $CDCl_3$) δ 159.50, 140.01, 131.88, 128.90, 128.77, 128.04, 127.38, 114.20, 68.24, 55.43.

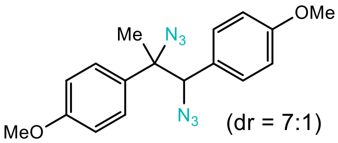


**4,4'-(1,2-Diazidopropane-1,2-diyl)bis(methoxybenzene) (3bi)**

Preparative thin layer chromatography (eluent: PE/EA = 20:1, v/v) to afford **3bi** as a light-yellow oil (18.2 mg, 54%, dr = 7:1). $R_f$ = 0.3 (PE/EA = 30:1, v/v). $^{1}$H NMR (400 MHz, $CDCl_3$) δ 7.23 – 7.18 (m, 2H), 7.01 – 6.95 (m, 2H), 6.87 – 6.82 (m, m, 2H), 6.81 – 6.77 (m, 2H), 4.58 (s, 1H), 3.81 (s, 3H), 3.79 (s, 3H), 1.72 (s, 3H). $^{13}$C NMR (101 MHz, $CDCl_3$) δ 159.80, 159.36, 132.10, 130.20, 128.36, 127.30, 113.64, 113.44, 73.96, 68.90, 55.39, 55.35, 21.33. HR-ESI-MS m/z calcd for $C_{17}H_{18}N_6O_2Na$ [$(M+Na)^+$]: 361.1389, found: 361.1382. IR: 3321(w), 2961(w), 2909(w), 2837(w), 2479(w), 2176(w), 2090(s), 1609(s), 1584(m), 1511(s), 1462(m), 1304(m), 1235(s), 1175(s), 1110(s), 1027(s), 930(w), 894(w), 854(w), 824(s), 769(s), 677(m), 538(s) $cm^{-1}$.

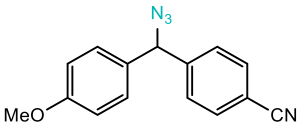


**4-(Azido(4-methoxyphenyl)methyl)benzonitrile (4l)**

Preparative thin layer chromatography (eluent: PE/EA = 8:1, v/v) to afford **4l** as a colorless oil (16.2 mg, 61%). $R_f$ = 0.3 (PE/EA = 10:1, v/v).

## 6.4 Determination of By-products in Carbon Deletion Reaction

### 6.4.1 Preparation of Substrates

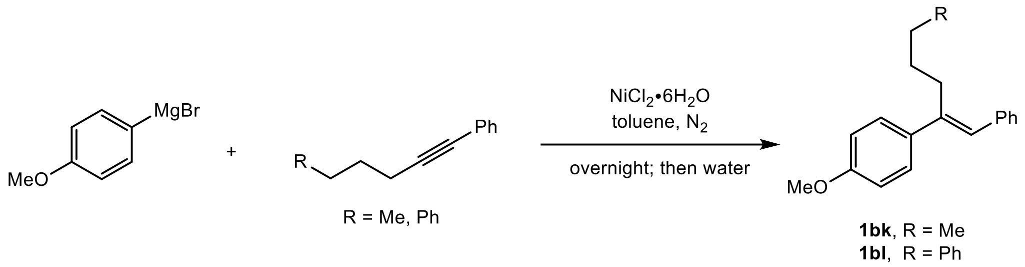


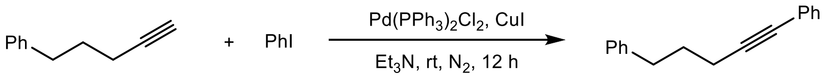


Trisubstituted alkenes **1bk** and **1bl** were prepared via nickel-catalyzed addition reaction of (4-methoxyphenyl)magnesium bromide to the corresponding alkynes according to the literature procedure[52]. Alkene **1bk**[52] is a known compound.

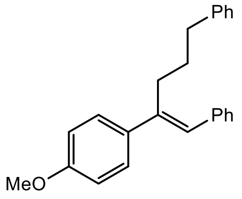


**(*E*)-(2-(4-Methoxyphenyl)pent-1-ene-1,5-diyl)dibenzene (1bl)**

Flash column chromatography on silica gel (eluent: PE/EA = 80:1 to 30:1, v/v) to afford **1bl** as an orange solid (816.8 mg, 83%, 3 mmol scale). $R_f$ = 0.7 (PE/EA = 30:1, v/v). $^{1}$H NMR (400 MHz, $CDCl_3$) δ 7.42 – 7.33 (m, 2H), 7.33 (m, 2H), 7.30 – 7.24 (m, 2H), 7.27 – 7.14 (m, 4H), 7.12 (d, *J* = 7.0 Hz, 2H), 6.95 – 6.87 (m, 2H), 6.67 (s, 1H), 3.85 (s, 3H), 2.77 – 2.71 (m, 2H), 2.63 (t, *J* = 7.5 Hz, 2H), 1.79 (tt, *J* = 10.7, 6.6 Hz, 2H). $^{13}$C NMR (101 MHz, $CDCl_3$) δ 159.11, 142.38, 142.20, 138.49, 135.47, 128.85, 128.62, 128.38, 128.36, 127.77, 127.28, 126.46, 125.88, 113.92, 55.44, 35.88, 30.48, 29.74. HR-ESI-MS m/z calcd for $C_{24}H_{25}O$ [$(M+H)^+$]: 329.1905, found: 329.1901.

### 6.4.2 Reactions of 1bj and 1bk

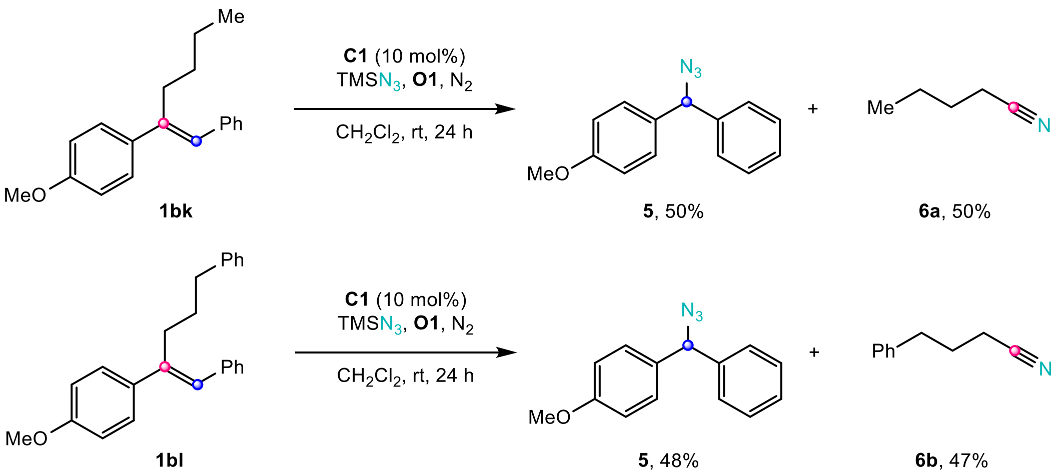


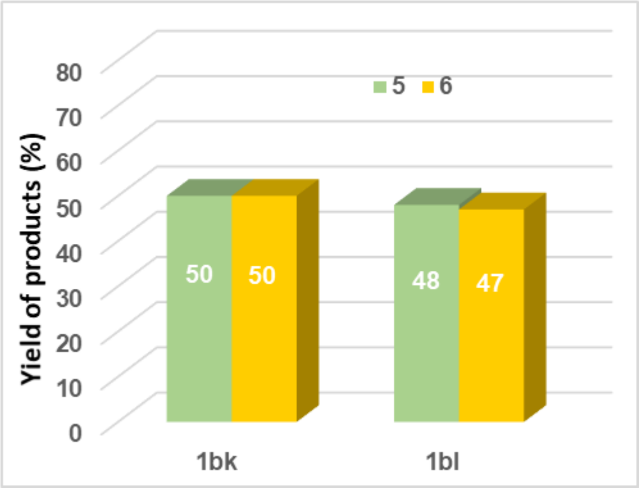


**Supplementary Figure 13.** Results of azidation of **1bk** and **1bl**

To a vial equipped with a stir bar were added trisubstituted alkene **1bk** or **1bl** (0.05 mmol, 1.0 equiv), **O1** (13.6 mg 0.06 mmol, 1.2 equiv), and **C1** (1.6 mg, 0.005 mmol, 10 mol%) successively. Then the vial was transferred into glovebox. After dry $CH_2Cl_2$ (350 $\mu$L) and $TMSN_3$ (20.0 $\mu$L, 0.15 mmol, 3.0 equiv) were added successively under inert gas, the vial was capped and removed from glovebox. The reaction was performed at room temperature for 24 h. The solvent was removed under reduced pressure. Yields of the different species were determined by proton NMR using 1,1,2,2-tetrachloroethane as the internal standard. Nitrile **6a** is commercially available, and nitrile **6b** is a known compound[53].

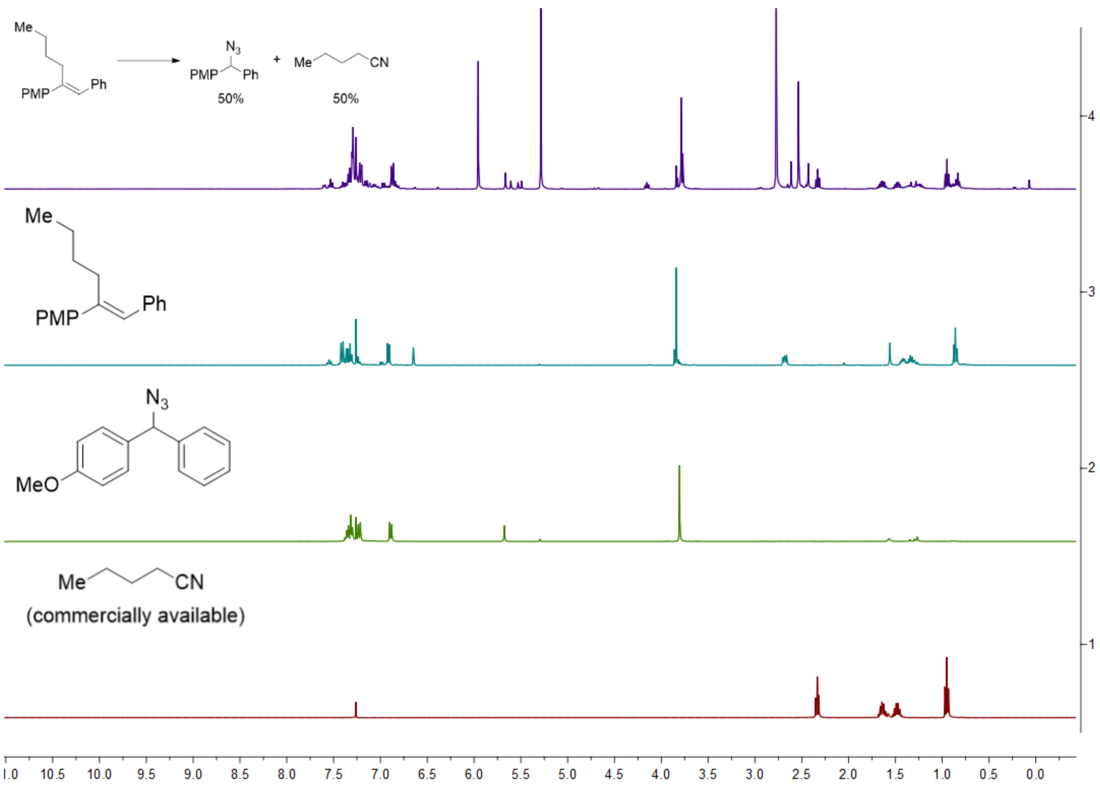


**Supplementary Figure 14.** The superposed NMR spectra of reaction of **1bk**

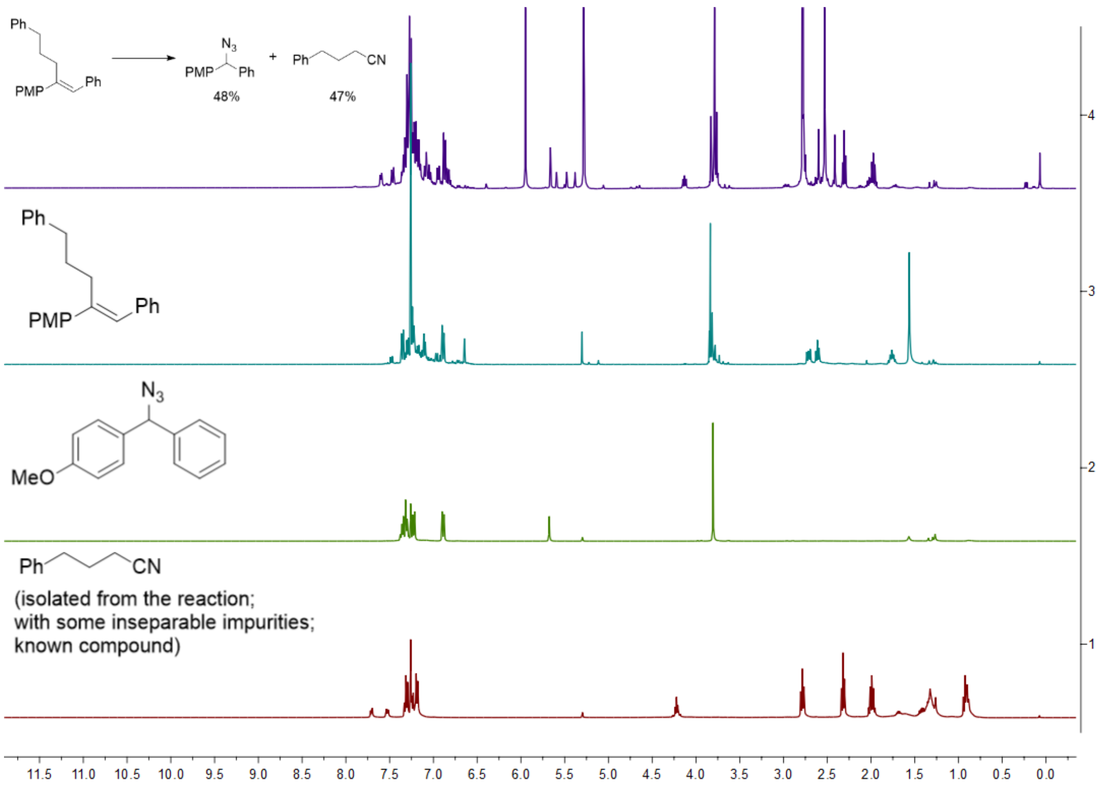


**Supplementary Figure 15.** The superposed NMR spectra of reaction of **1bl**

### 6.5 Influence of Tetrafluoroborates in the Reactions

**Supplementary Table 6. Influence of tetrafluoroborates**

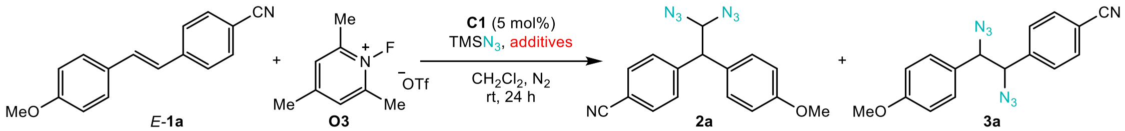


| entry | additives | yield [%][a] | |
|---|---|---|---|
| | | **2a** | **3a** |
| 1 | no | 0 | 9 |
| 2 | $NaBF_4$ (2.0 equiv) | 4 | 7 |
| 3 | $LiBF_4$ (2.0 equiv) | 27 | 55 |
| 4 | $NH_4BF_4$ (2.0 equiv) | 46 | 16 |

Reaction conditions: *E*-**1a** (0.05 mmol), $TMSN_3$ (3.0 equiv), **O3** (2.0 equiv), **C1** (5 mol%), solvent (175 $\mu$L), $N_2$, RT, 24 h. [a]Refers to NMR yield with 1,1,2,2-tetrachloroethane as the internal standard.

To a vial equipped with a stir bar were added *E*-**1a** (11.8 mg, 0.05 mmol, 1.0 equiv), **O3** (28.9 mg, 0.1 mmol, 2.0 equiv), **C1** (0.8 mg, 0.0025 mmol, 5 mol%), and additives (0.1 mmol, 2.0 equiv: $NaBF_4$, 11.0 mg; $LiBF_4$, 9.4 mg; $NH_4BF_4$, 10.5 mg) successively. Then the vial was transferred into glovebox. After dry $CH_2Cl_2$ (175 $\mu$L) and $TMSN_3$ (20.0 $\mu$L, 0.15 mmol, 3.0 equiv) were added successively under inert gas, the vial was capped and removed from glovebox. The reaction was performed at room temperature for 24 h. The solvent was removed under reduced pressure. Yields of the different species were determined by proton NMR using 1,1,2,2-tetrachloroethane as the internal standard.

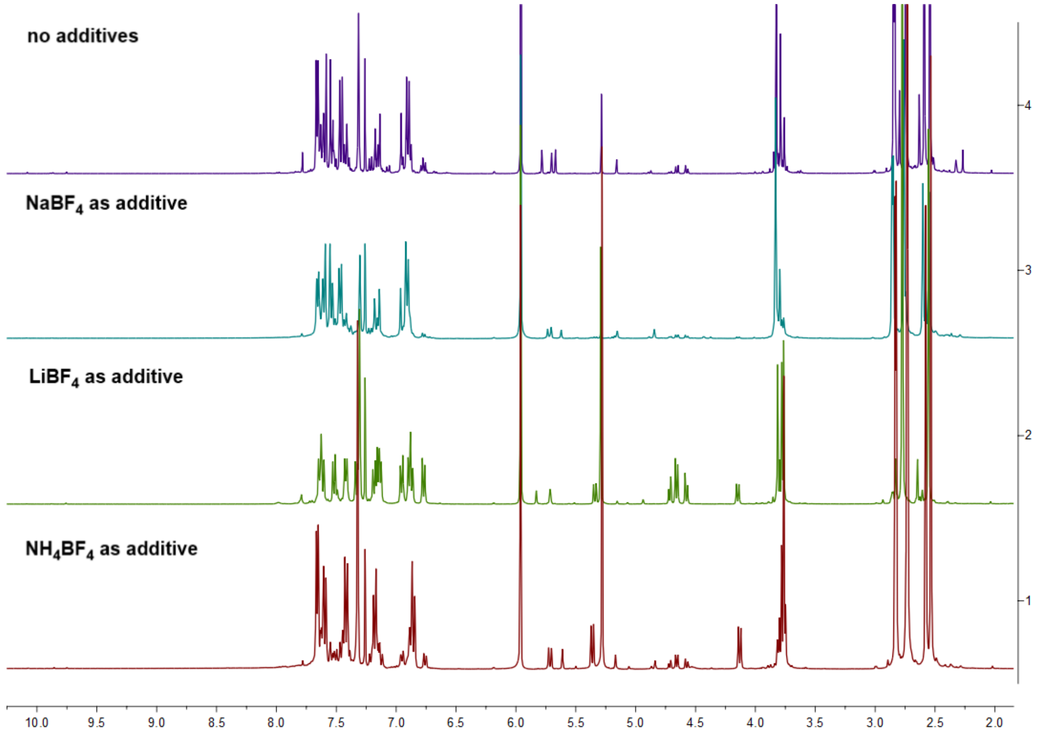


**Supplementary Figure 16.** The superposed NMR spectra of reactions using different tetrafluoroborates

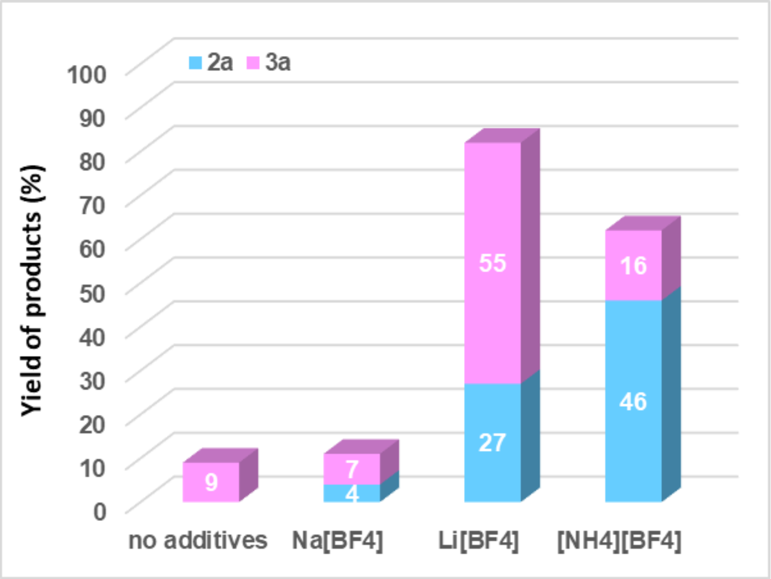


**Supplementary Figure 17.** NMR yields of reactions using different tetrafluoroborates

## 6.6 Isolation of Possible Intermediate and Its Oxidative Azidation

### 6.6.1 Isolation of Possible Intermediate

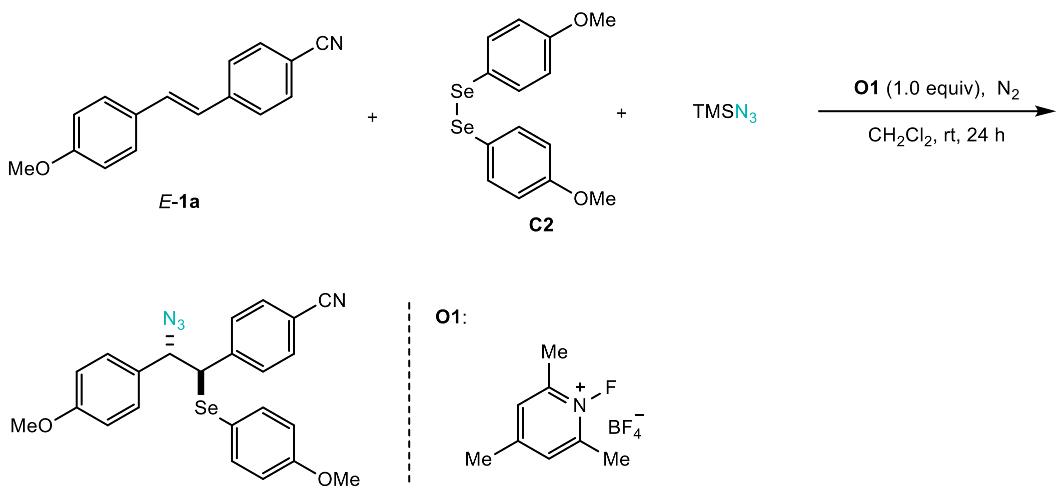


To a dry vial equipped with a stir bar were added *E*-**1a** (47.1 mg, 0.2 mmol, 1.0 equiv), **O1** (45.4 mg 0.2 mmol, 1.0 equiv), and **C2** (74.4 mg, 0.2 mmol, 1.0 equiv) successively. Then the vial was transferred into glovebox. After dry $CH_2Cl_2$ (700 $\mu$L) and $TMSN_3$ (80.0 $\mu$L, 0.6 mmol, 3.0 equiv) were added successively under inert gas, the vial was capped and removed from glovebox. The reaction was performed at room temperature for 24 h. Then the resulting mixture was quenched with diluted hydrochloric acid (1 mL, 1 M) and extracted with $CH_2Cl_2$ (5 mL × 3). The combined organic layers were washed with brine, dried over $Na_2SO_4$, and concentrated under reduced pressure. The residue was purified by flash column chromatography to give the selenylated azide **7**.

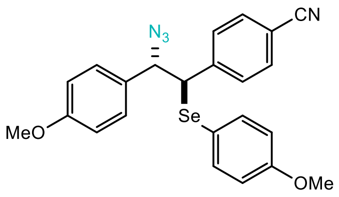


***rel*-4-((1*R*,2*S*)-2-Azido-2-(4-methoxyphenyl)-1-((4-methoxyphenyl)selanyl)ethyl)benzonitrile (7)**

Flash column chromatography (eluent: PE/EA = 10:1 to 5:1, v/v) to afford **7** as a light-yellow oil (75.8 mg, 82%). $R_f$ = 0.4 (PE/EA = 5:1). $^1$H NMR (400 MHz, $CDCl_3$) δ 7.49 (d, *J* = 8.1 Hz, 2H), 7.22 (d, *J* = 8.1 Hz, 2H), 7.16 (d, *J* = 8.6 Hz, 2H), 7.10 (d, *J* = 8.6 Hz, 2H), 6.88 (d, *J* = 8.6 Hz, 2H), 6.66 (d, *J* = 8.7 Hz, 2H), 4.97 (d, *J* = 8.8 Hz, 1H), 4.36 (d, *J* = 8.9 Hz, 1H), 3.82 (s, 3H), 3.76 (s, 3H). $^{13}$C NMR (151 MHz, $CDCl_3$) δ 160.32, 160.12, 144.88, 138.06, 131.98, 129.63, 128.92, 128.84, 118.89, 118.13, 114.80, 114.27, 111.00, 68.54, 55.40, 55.38, 52.97. HR-ESI-MS m/z calcd for $C_{23}H_{20}N_4O_2SeNa$

[(M+Na)$^+$]: 487.0649, found: 487.0645. IR: 3003(w), 2936(w), 2837(w), 2227(m), 2094(s), 1607(m), 1587(m), 1512(m), 1489(s), 1461(m), 1286(m), 1244(s), 1173(s), 1103(w), 1027(s), 823(s), 738(m), 659(m), 601(m), 553(s), 521(s) cm$^{-1}$.

### 6.6.2 Oxidative Azidation of Possible Intermediate

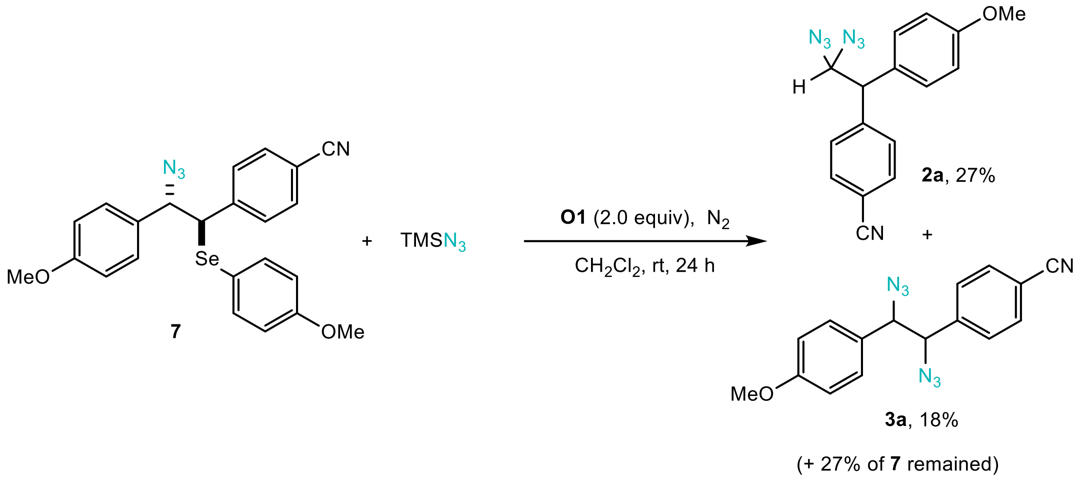


To a dry vial equipped with a stir bar was added **O1** (22.7 mg, 0.1 mmol, 2.0 equiv). Then the vial was transferred into glovebox. To the vial was added a solution of **7** (0.05 mmol, 1.0 equiv) and $TMSN_3$ (0.15 mmol, 3.0 equiv) in dry $CH_2Cl_2$ (200 $\mu$L) under inert gas. After the vial was capped and removed from glovebox, the reaction was performed at room temperature for 24 h. The solvent was removed under reduced pressure. Yields of the different species were determined by proton NMR using 1,1,2,2-tetrachloroethane as the internal standard.

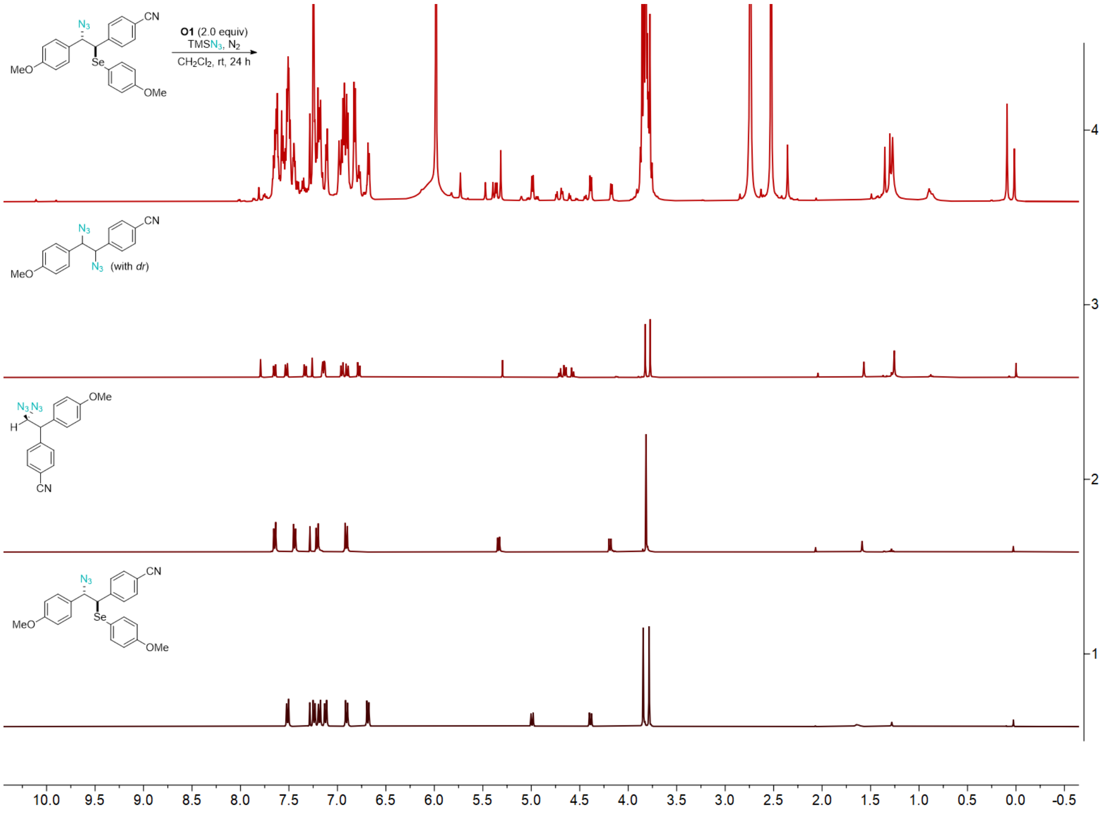

**Supplementary Figure 18.** Oxidative azidation of possible intermediate

## 6.7 Studies of Exclusion of Aldehyde as Possible Intermediate

### 6.7.1 Formation of Aldehyde using Water as Nucleophile

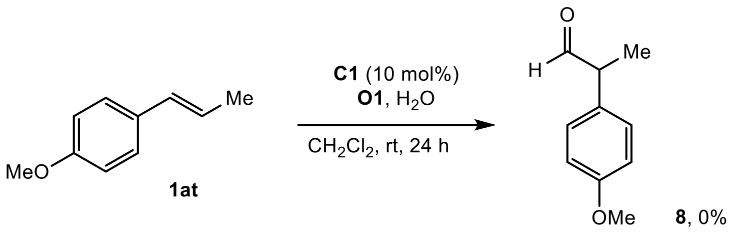


To a vial equipped with a stir bar were added **1at** (7.5 $\mu$L, 0.05 mmol, 1.0 equiv), **O1** (13.6 mg, 0.06 mmol, 1.2 equiv), and **C1** (1.6 mg, 0.005 mmol, 10 mol%) successively. After that $CH_2Cl_2$ (500 $\mu$L) and $H_2O$ (1.0 $\mu$L, 0.05 mmol, 1.0 equiv) were added successively under air, the vial was capped and the reaction was performed at room temperature for 24 h. The solvent was removed under reduced pressure. Yields of the product was determined by proton NMR using 1,1,2,2-tetrachloroethane as the internal standard.

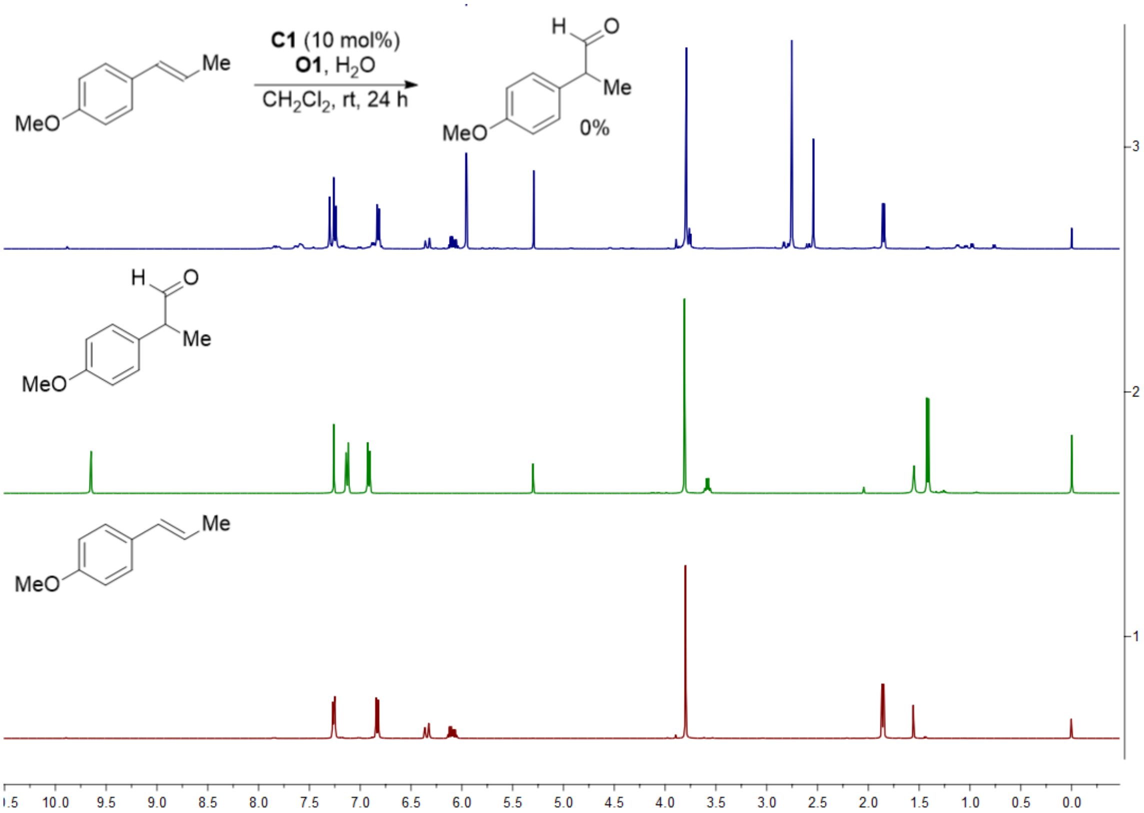


**Supplementary Figure 19.** Reaction using water instead of azidotrimethylsilane as nucleophile

### 6.7.2 Using Aldehyde as Substrate in Possible Azidation

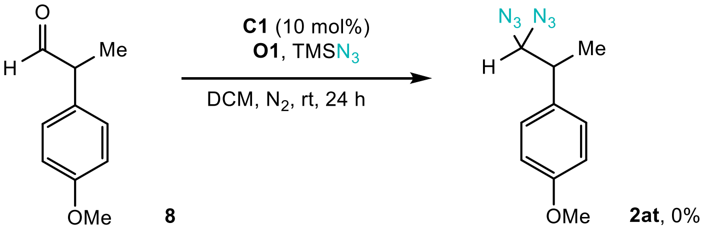


To a vial equipped with a stir bar were added aldehyde **8** (8.2 mg, 0.05 mmol, 1.0 equiv), **O1** (13.6 mg, 0.06 mmol, 1.2 equiv), and **C1** (1.6 mg, 0.005 mmol, 10 mol%) successively. Then the vial was transferred into glovebox. After dry $CH_2Cl_2$ (350 $\mu$L) and $TMSN_3$ (20.0 $\mu$L, 0.15 mmol, 3.0 equiv) were added successively under inert gas, the vial was capped and removed from glovebox. The reaction was performed at room temperature for 24 h. The solvent was removed under reduced pressure. The residue was analyzed by proton NMR using 1,1,2,2-tetrachloroethane as the internal standard.

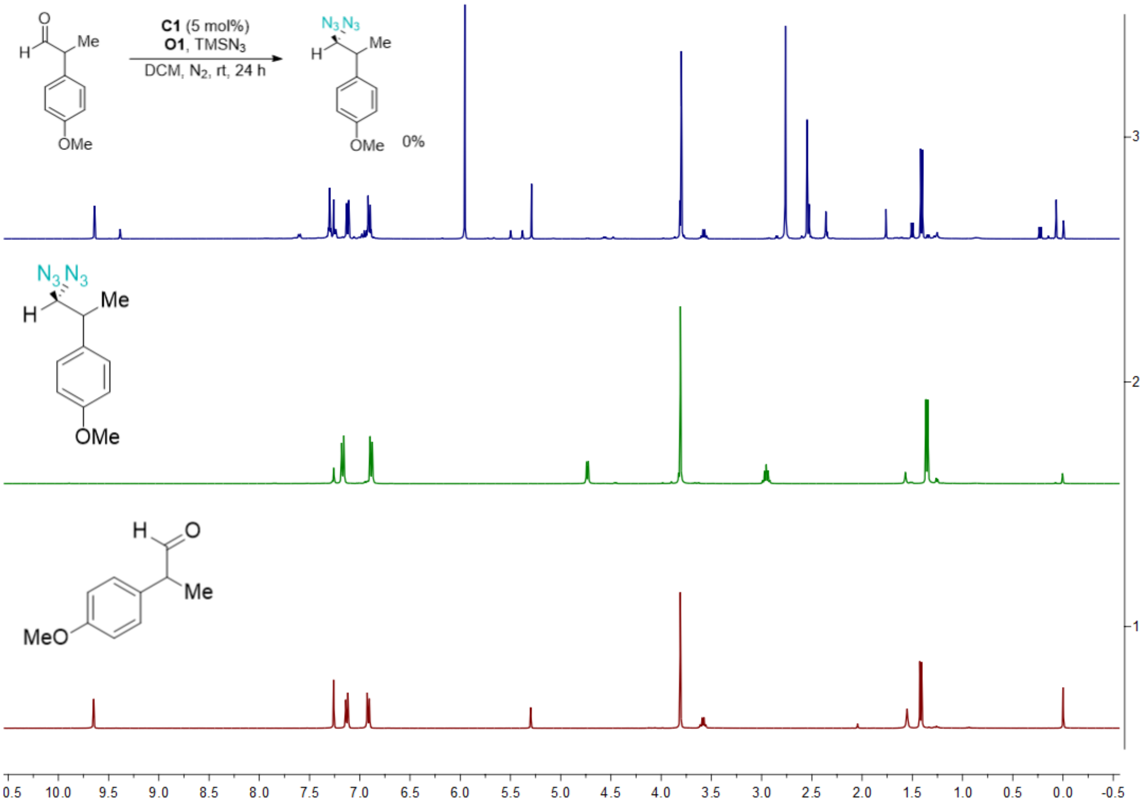


**Supplementary Figure 20.** Reaction using aldehyde as substrate in standard condition

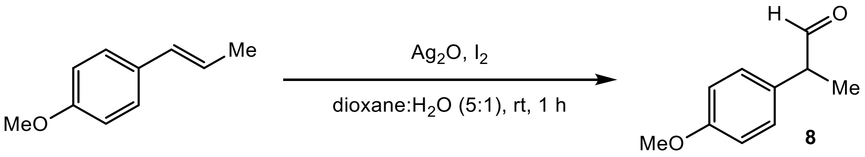

**8**

Aldehyde **8** was prepared according to the literature procedure[54].

## 6.8 In Comparison with Hypervalent Iodine Systems

### 6.8.1 Using Stoichiometric Hypervalent Iodine Reagents as Oxidants

**Supplementary Table 7. Exploration of azidation mediated by hypervalent iodine reagents**

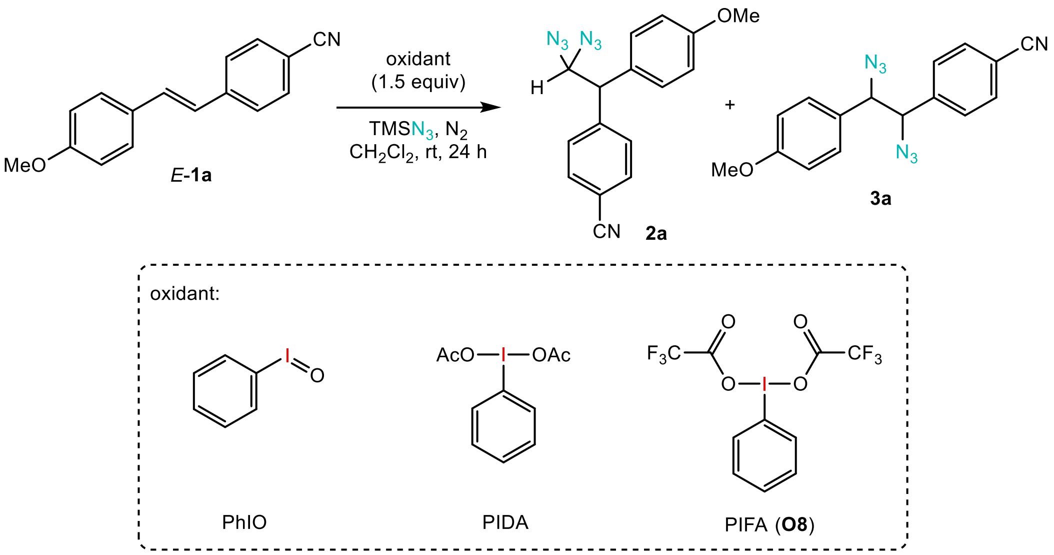


| entry | oxidant | yield [%][a] | |
|---|---|---|---|
| | | **2a** | **3a** |
| 1 | PhIO | 0 | 35 |
| 2 | PIDA | 0 | 47 |
| 3 | PIFA | 0 | 39 |

Reaction conditions: *E*-**1a** (0.05 mmol), $TMSN_3$ (3.0 equiv), **O3** (1.5 equiv), $CH_2Cl_2$ (175 $\mu$L), $N_2$, RT, 24 h. [a]Refers to NMR yield with 1,1,2,2-tetrachloroethane as the internal standard.

To a vial equipped with a stir bar were added *E*-**1a** (11.8 mg, 0.05 mmol, 1.0 equiv), and **O3** (28.9 mg, 0.1 mmol, 2.0 equiv) successively. Then the vial was transferred into glovebox. After dry $CH_2Cl_2$ (175 $\mu$L) and $TMSN_3$ (20.0 $\mu$L, 0.15 mmol, 3.0 equiv) were added successively under inert gas, the vial was capped and removed from glovebox. The reaction was performed at room temperature for 24 h. The solvent was removed under reduced pressure. Yields of the different species were determined by proton NMR using 1,1,2,2-tetrachloroethane as the internal standard.

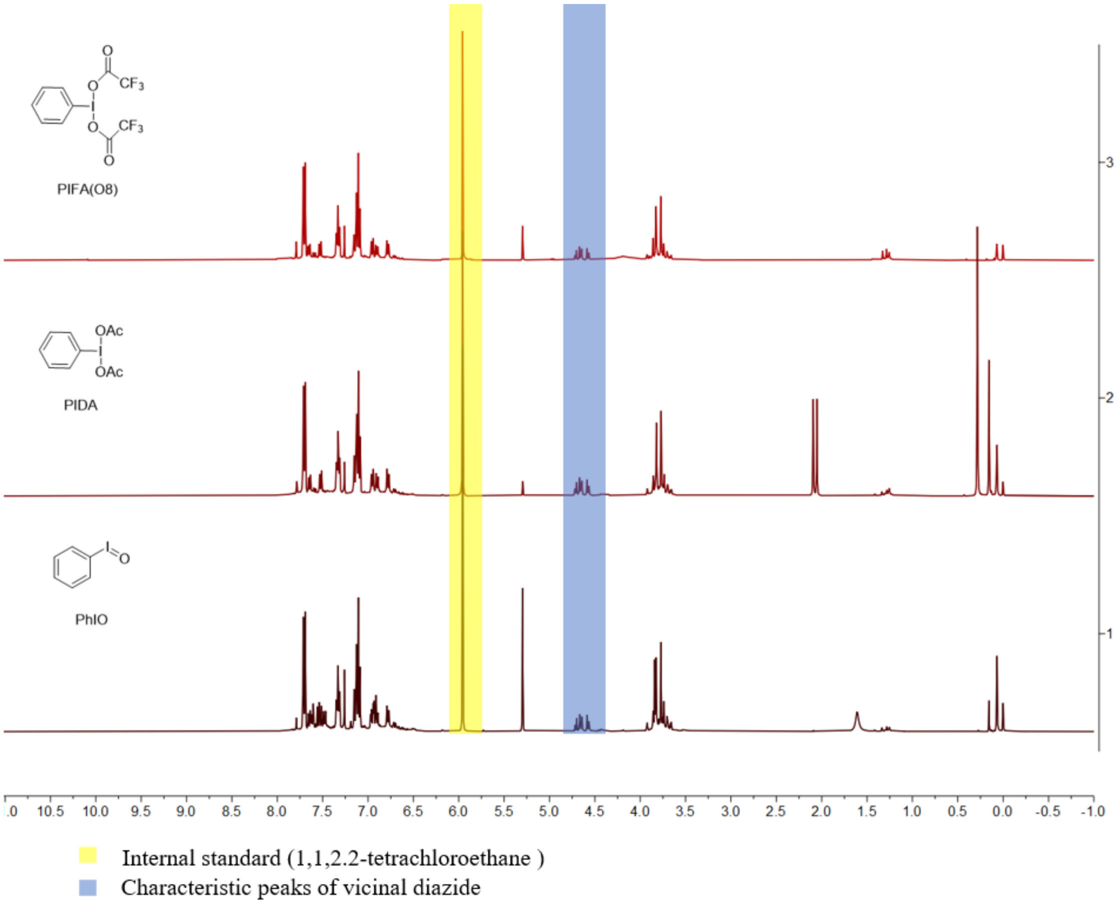


**Supplementary Figure 21.** The superposed NMR spectra of azidation mediated by hypervalent iodine reagents

### 6.8.2 Using Catalytic Aryl Iodide Combining with Stoichiometric Oxidants

## Supplementary Table 8. Exploration of azidation catalyzed by aryl iodine

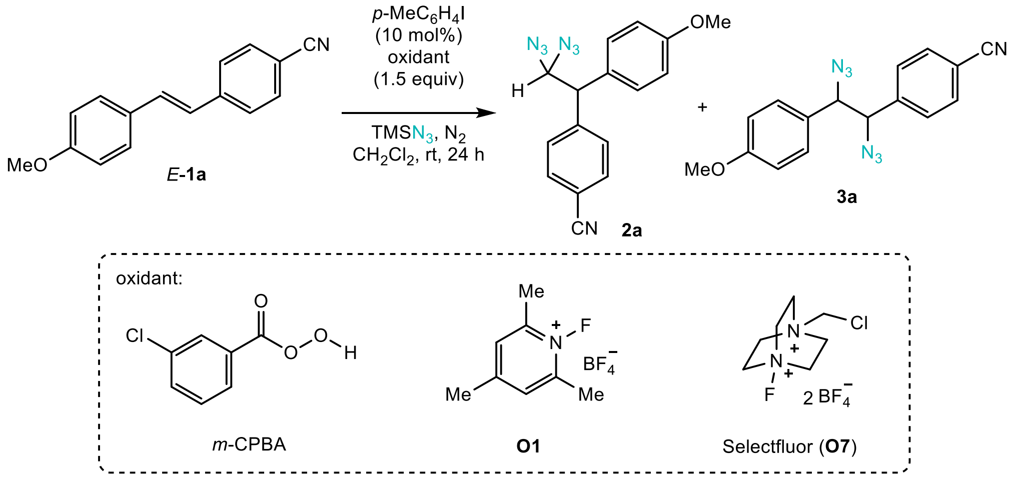

| entry | oxidant | yield [%][a] | | |
|---|---|---|---|---|
| | | **2a** | **3a** | **remaining 1a** |
| 1 | *m*-CPBA | 0 | 0 | 80 |
| 2 | **O1** | 0 | 0 | 92 |
| 3 | Selectfluor | 0 | 0 | 99 |

Reaction conditions: *E*-**1a** (0.05 mmol), $TMSN_3$ (3.0 equiv), **O3** (1.5 equiv), $CH_2Cl_2$ (175 $\mu$L), $N_2$, RT, 24 h. [a]Refers to NMR yield with 1,1,2,2-tetrachloroethane as the internal standard.

To a vial equipped with a stir bar were added *E*-**1a** (11.8 mg, 0.05 mmol, 1.0 equiv), and **O3** (28.9 mg, 0.1 mmol, 2.0 equiv) successively. Then the vial was transferred into glovebox. After dry $CH_2Cl_2$ (175 $\mu$L) and $TMSN_3$ (20.0 $\mu$L, 0.15 mmol, 3.0 equiv) were added successively under inert gas, the vial was capped and removed from glovebox. The reaction was performed at room temperature for 24 h. The solvent was removed under reduced pressure. Yields of the different species were determined by proton NMR using 1,1,2,2-tetrachloroethane as the internal standard.

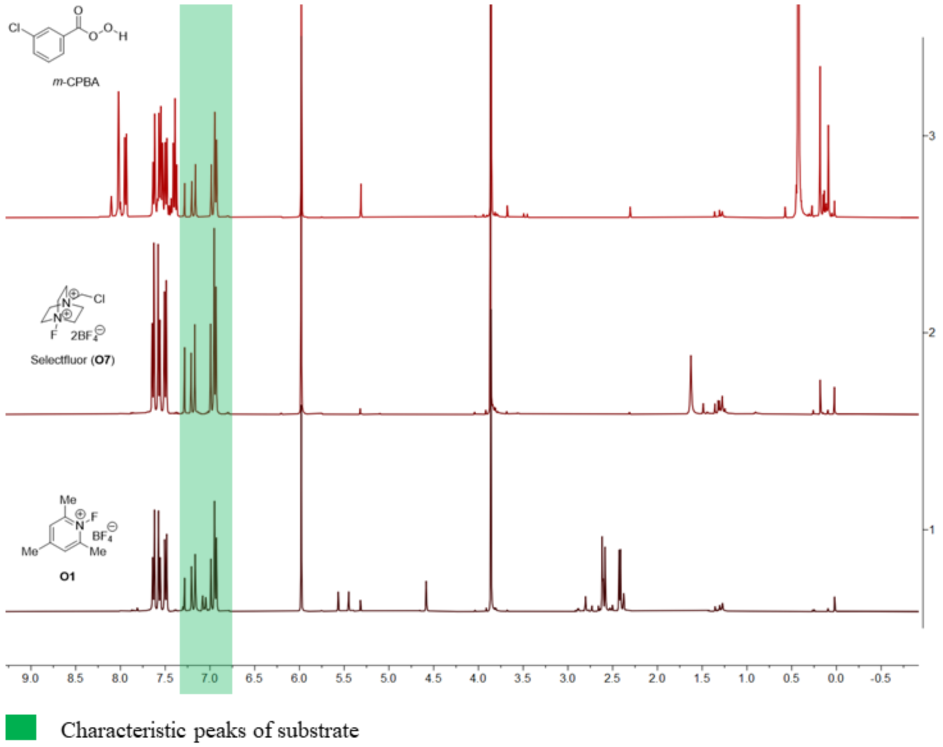


**Supplementary Figure 22.** The superposed NMR spectra of azidation catalyzed by aryl iodine

## 6.9 Comparison of Characterization Data between Geminal and Vicinal Diazides

To compare the difference of characterization data between geminal and vicinal diazides, the following vicinal diazides were prepared according to the literature procedure via *Method a* or *Method b* [55,56].

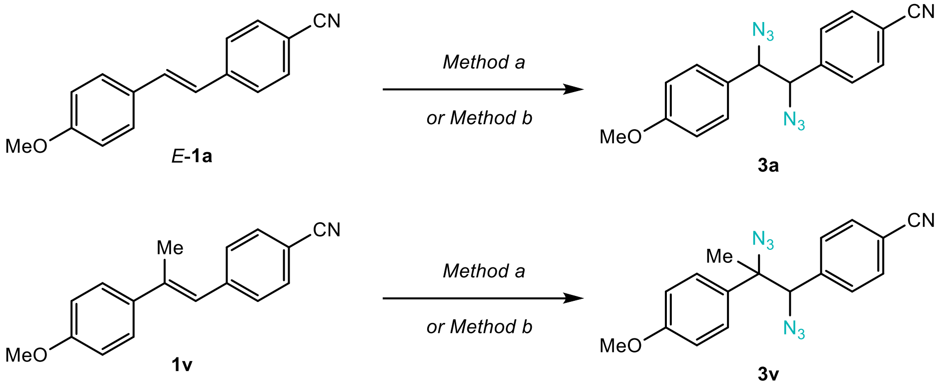


*Method a*: *E*-**1a** (0.1 mmol), $TMSN_3$ (4.4 equiv), $PhI(OAc)_2$ (2.2 equiv), $CH_2Cl_2$ (500 $\mu$L), $N_2$ atmosphere, 0-15 ℃, blue light (7W), 4 h. *Method b*: *E*-**1a** (0.1 mmol), $TMSN_3$ (3.5 equiv), CuI (10 mol%), benzenecarboperoxoic acid (1.5 equiv), MeCN (500 $\mu$L), $N_2$ atmosphere, 50°C, 4 h.

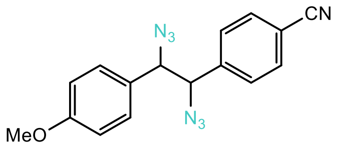


*dr* = 1:1.2

**4-(1,2-Diazido-2-(4-methoxyphenyl)ethyl)benzonitrile (3a)**

According to method a，preparative thin layer chromatography (eluent: PE/EA = 10:1, v/v) afford **3a** as a light-yellow oil (23.8 mg, 74%, *dr* = 1:1.2) with inseparable impurities. $R_f$ = 0.3 (PE/EA = 10:1, v/v). $^1$H NMR (400 MHz, $CDCl_3$) δ 7.65 (d, *J* = 8.0 Hz, 2H), 7.53 (d, *J* = 8.1 Hz, 2.5H), 7.33 (d, *J* = 8.0 Hz, 2H), 7.14 (d, *J* = 9.0 Hz, 4.5H), 6.95 (d, *J* = 8.5 Hz, 2.5H), 6.90 (d, *J* = 8.6 Hz, 2H), 6.78 (d, *J* = 8.9 Hz, 2.5H), 4.71 (d, *J* = 7.0 Hz, 1H), 4.65 (d, *J* = 9.1 Hz, 2.2H), 4.57 (d, *J* = 8.3 Hz, 1.2H), 3.82 (s, 3H), 3.77 (s, 3.7H). $^{13}$C NMR (101 MHz, $CDCl_3$) δ 160.38, 160.18, 141.37, 132.92, 132.41, 132.34, 129.28, 129.00, 128.88, 128.60, 126.94, 118.45, 118.36, 114.40, 114.32, 112.89, 112.67, 70.31, 70.18, 69.27, 69.21, 55.45, 55.40. HR-ESI-MS m/z calcd for $C_{16}H_{13}N_7ONa$ [$(M+Na)^+$]: 342.1079, found: 342.1073. IR: 3334(w), 2935(w), 2839(w), 2496(w), 2229(m), 2096(s),

1610(m), 1512(s), 1461(m), 1415(m), 1247(s), 1176(s), 1112(m), 1030(s), 941(w), 829(s), 776(m), 750(m), 676(m), 560(s), 503(w) cm$^{-1}$.

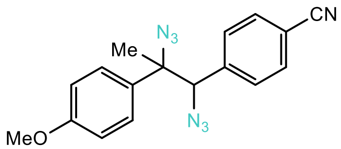


*dr* = 4:1

**4-(1,2-Diazido-2-(4-methoxyphenyl)propyl)benzonitrile (3v)**

According to method b，preparative thin layer chromatography (eluent: PE/EA = 10:1, v/v) afford **3a** as a colorless oil (29.1mg, 87%, *dr* = 4:1) with inseparable impurities. $R_f$ = 0.3 (PE/EA = 10:1, v/v). $^1$H NMR (400 MHz, $CDCl_3$) δ 7.54 (d, *J* = 8.4 Hz, 0.5H), 7.48 (d, *J* = 8.4 Hz, 2H), 7.18 (dd, *J* = 8.5, 6.2 Hz, 1H), 7.08 (d, *J* = 8.9 Hz, 2H), 6.96 (d, *J* = 8.3 Hz, 2H), 6.88 – 6.80 (m, 2.5H), 4.68 (s, 1H), 4.64 (s, 0.25H), 3.81 (s, 3H), 3.81 (s, 0.75H), 1.75 (s, 0.75H), 1.69 (s, 3H). $^{13}$C NMR (101 MHz, $CDCl_3$) δ 159.74, 159.61, 140.54, 132.91, 131.67, 131.58, 131.00, 130.72, 129.71, 129.37, 128.29, 127.99, 118.49, 113.94, 113.89, 112.39, 73.80, 73.65, 69.71, 55.42, 21.27, 19.45. HR-ESI-MS m/z calcd for $C_{17}H_{16}N_7O$ [(M+H)$^+$]: 334.1416, found: 334.1414. IR: 3339(w), 2936(w), 2838(w), 2495(w), 2229(m), 2098(s), 1675(w), 1608(m), 1511(s), 1461(m), 1414(m), 1378(m), 1339(m), 1249(s), 1181(s), 1119(m), 1076(m), 1029(s), 966(m), 837(s), 759(m), 676(m), 559(m) cm$^{-1}$.

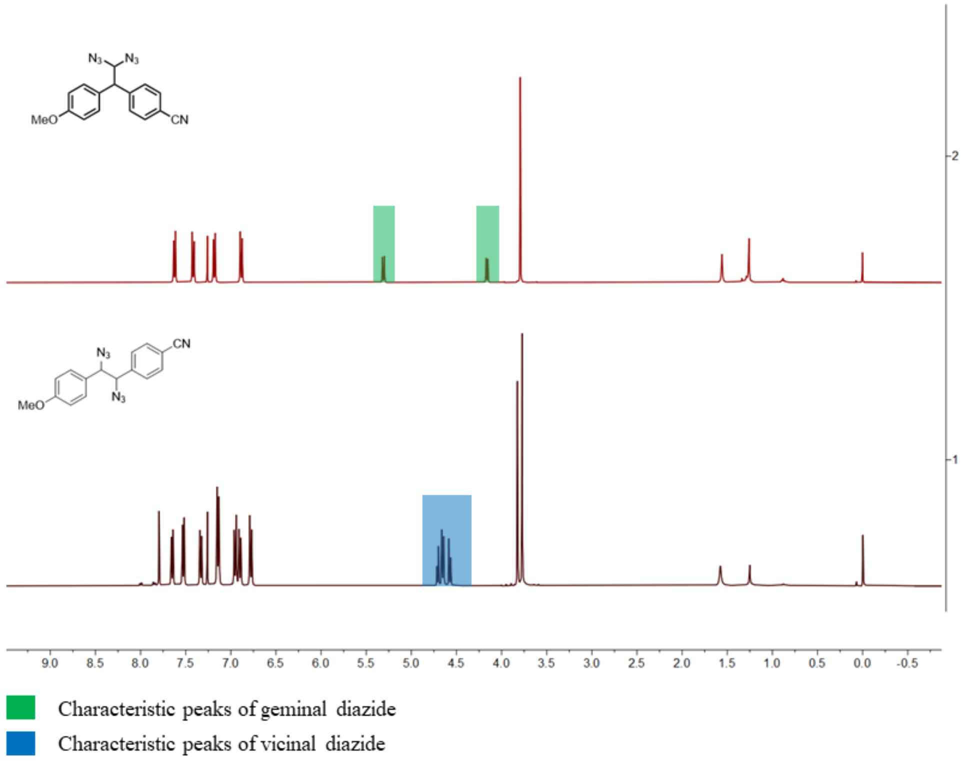


**Supplementary Figure 23.** NMR spectra comparison between vicinal and geminal diazides generated from disubstituted alkenes

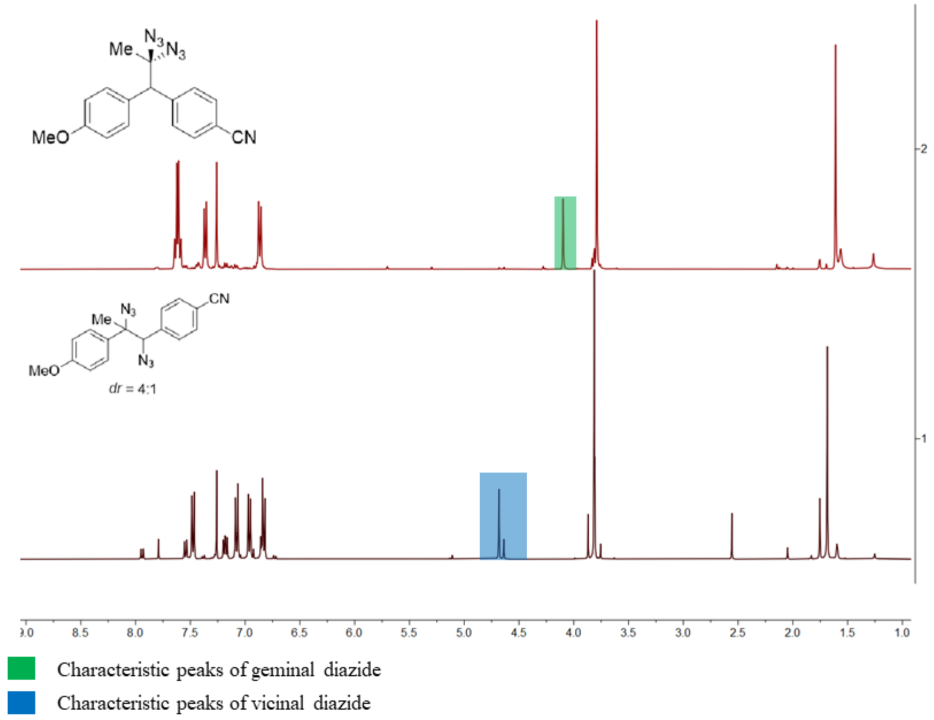


**Supplementary Figure 24.** NMR spectra comparison between vicinal and geminal diazides generated from trisubstituted alkenes.

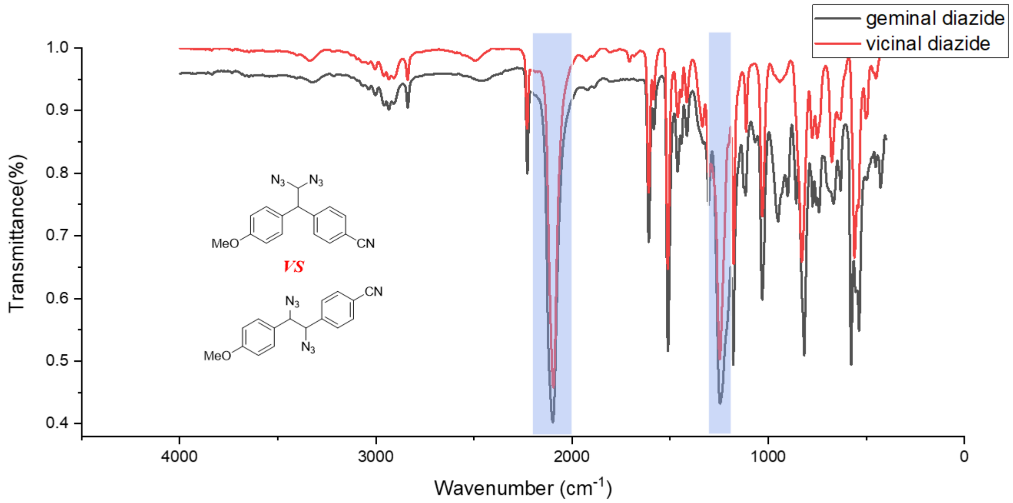


**Supplementary Figure 25.** IR spectrogram of **2a** (geminal diazide) and **3a** (vicinal diazide), characteristic absorption peaks of azide group are framed in light purple

## 6.10 Supplementary Discussion

### 6.10.1 Proposed Mechanisms of Formation of 4j and 4k

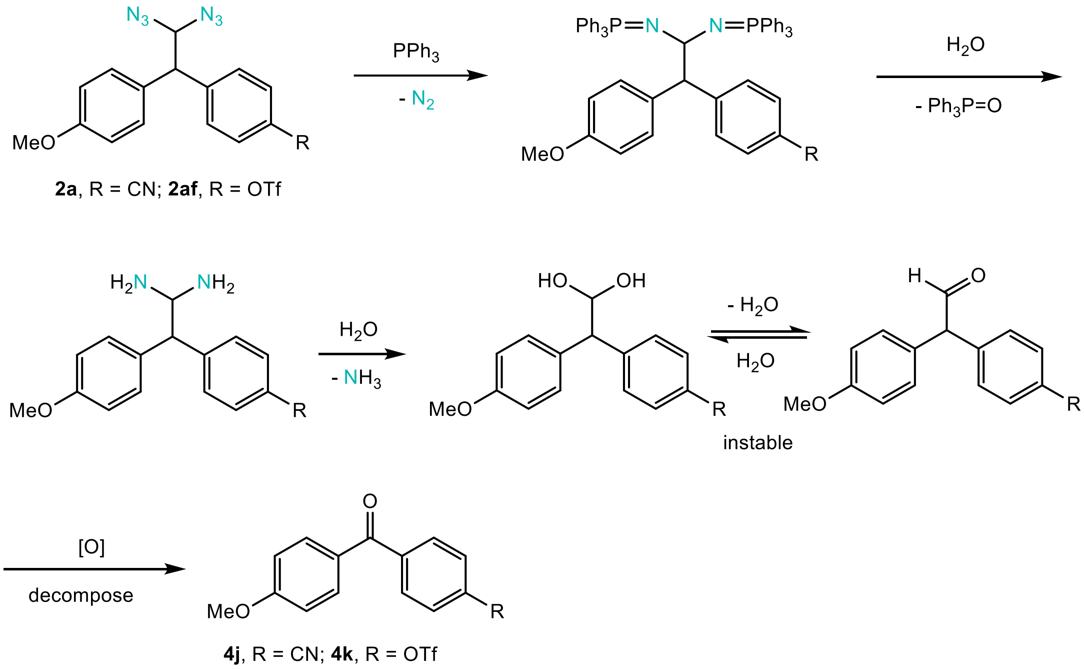


### 6.10.2 Proposed Mechanisms of Formation of 4l-4p

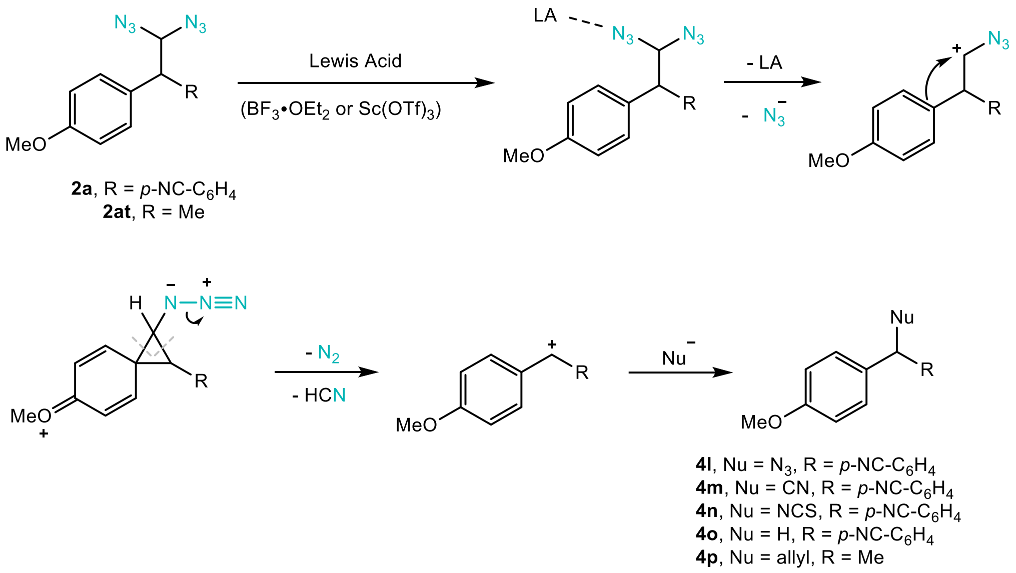

# 7. X-Ray Crystallographic Data

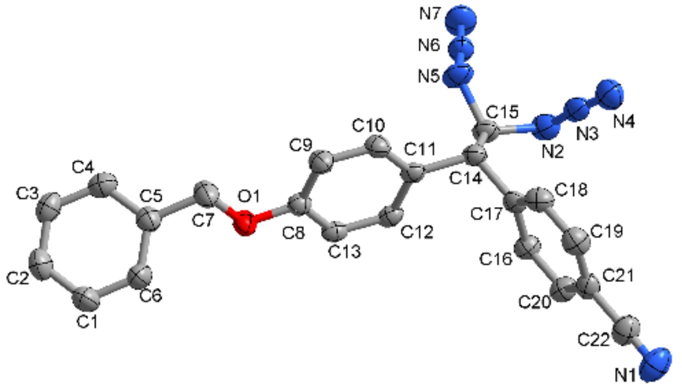


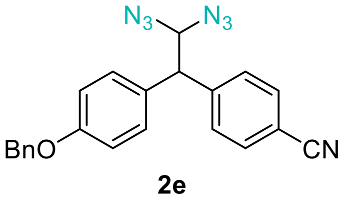


**2e**

CCDC 2253572

Single crystals of **2e** were grown by slow evaporation of its $CH_2Cl_2$/PE solution. Single crystal X-ray diffraction data were collected with a SuperNova, Dual, Cu at home/near, AtlasS2 diffractometer. The crystal was kept at 149.99(10) K during data collection. Using Olex2,[57] the structure was solved with the ShelXT[58] structure solution program using Intrinsic Phasing and refined with the ShelXL[59] refinement package using Least Squares minimization. Supplementary crystallographic data have been deposited at the Cambridge Crystallographic Data Center (CCDC 2253572).

**Supplementary Table 9. Crystal Data and Structure Refinement for 2e**

| | |
|---|---|
| Empirical formula | $C_{22}H_{17}N_7O$ |
| Formula weight | 395.42 |
| Temperature/K | 149.99(10) |
| Crystal system | triclinic |
| Space group | P-1 |
| a/Å | 10.5318(3) |
| b/Å | 12.2652(4) |
| c/Å | 17.0621(6) |
| α/° | 70.186(3) |
| β/° | 75.610(3) |

| | |
|---|---|
| γ/° | 86.008(2) |
| Volume/Å$^3$ | 2008.20(12) |
| Z | 4 |
| $\rho_{calc}$g/cm$^3$ | 1.308 |
| μ/mm$^{-1}$ | 0.698 |
| F(000) | 824.0 |
| Crystal size/mm$^3$ | 0.180 × 0.120 × 0.080 |
| Radiation | Cu Kα (λ = 1.54184) |
| 2Θ range for data collection/° | 5.67 to 134.998 |
| Index ranges | -12 ≤ h ≤ 9, -14 ≤ k ≤ 14, -20 ≤ l ≤ 19 |
| Reflections collected | 13770 |
| Independent reflections | 7241 [$R_{int}$ = 0.0327, $R_{sigma}$ = 0.0410] |
| Data/restraints/parameters | 7241/0/549 |
| Goodness-of-fit on F$^2$ | 1.045 |
| Final R indexes [I>=2σ (I)] | $R_1$ = 0.0543, w$R_2$ = 0.1452 |
| Final R indexes [all data] | $R_1$ = 0.0630, w$R_2$ = 0.1557 |
| Largest diff. peak/hole / e Å$^{-3}$ | 0.45/-0.49 |

**Supplementary Table 10. Bond Lengths for 2e**

| Atom | Atom | Length/Å | Atom | Atom | Length/Å |
|---|---|---|---|---|---|
| O1 | C8 | 1.368(2) | C14 | C15 | 1.539(3) |
| O1 | C7 | 1.431(3) | C10 | C9 | 1.391(3) |
| N3 | N2 | 1.245(3) | C8 | C9 | 1.388(3) |
| N3 | N4 | 1.129(3) | C16 | C20 | 1.381(3) |
| N6 | N5 | 1.238(3) | C21 | C20 | 1.395(3) |
| N6 | N7 | 1.124(3) | C21 | C19 | 1.383(3) |
| N5 | C15 | 1.484(3) | C21 | C22 | 1.445(3) |
| N2 | C15 | 1.463(3) | C5 | C6 | 1.387(3) |
| C13 | C12 | 1.380(3) | C5 | C4 | 1.391(3) |
| C13 | C8 | 1.391(3) | C5 | C7 | 1.501(3) |
| C11 | C12 | 1.396(3) | N1 | C22 | 1.144(3) |
| C11 | C14 | 1.525(3) | C18 | C19 | 1.382(3) |
| C11 | C10 | 1.386(3) | C6 | C1 | 1.383(3) |
| C17 | C14 | 1.523(3) | C4 | C3 | 1.386(3) |

| Atom | Atom | Length/Å | Atom | Atom | Length/Å |
|---|---|---|---|---|---|
| C17 | C16 | 1.390(3) | C3 | C2 | 1.373(4) |
| C17 | C18 | 1.389(3) | C1 | C2 | 1.381(3) |

**Supplementary Table 11. Bond Angles for 2e**

| Atom | Atom | Atom | Angle/˚ | Atom | Atom | Atom | Angle/˚ |
|---|---|---|---|---|---|---|---|
| C8 | O1 | C7 | 117.56(15) | C8 | C9 | C10 | 119.71(18) |
| N4 | N3 | N2 | 172.8(2) | C20 | C21 | C22 | 120.3(2) |
| N7 | N6 | N5 | 172.3(3) | C19 | C21 | C20 | 119.86(19) |
| N6 | N5 | C15 | 114.53(19) | C19 | C21 | C22 | 119.8(2) |
| N3 | N2 | C15 | 113.93(18) | C6 | C5 | C4 | 118.2(2) |
| C12 | C13 | C8 | 120.35(17) | C6 | C5 | C7 | 120.86(19) |
| C12 | C11 | C14 | 121.24(16) | C4 | C5 | C7 | 120.89(19) |
| C10 | C11 | C12 | 117.95(18) | C16 | C20 | C21 | 119.84(19) |
| C10 | C11 | C14 | 120.81(17) | N5 | C15 | C14 | 104.07(16) |
| C16 | C17 | C14 | 123.17(18) | N2 | C15 | N5 | 111.83(17) |
| C18 | C17 | C14 | 118.01(17) | N2 | C15 | C14 | 115.77(18) |
| C18 | C17 | C16 | 118.81(18) | C19 | C18 | C17 | 120.99(19) |
| C13 | C12 | C11 | 121.11(17) | C18 | C19 | C21 | 119.8(2) |
| C11 | C14 | C15 | 110.61(16) | C1 | C6 | C5 | 120.8(2) |
| C17 | C14 | C11 | 112.06(14) | C3 | C4 | C5 | 120.9(2) |
| C17 | C14 | C15 | 113.26(15) | C2 | C3 | C4 | 120.1(2) |
| C11 | C10 | C9 | 121.52(18) | O1 | C7 | C5 | 108.29(17) |
| O1 | C8 | C13 | 115.36(16) | C2 | C1 | C6 | 120.2(2) |
| O1 | C8 | C9 | 125.30(17) | N1 | C22 | C21 | 178.1(3) |
| C9 | C8 | C13 | 119.34(18) | C3 | C2 | C1 | 119.8(2) |
| C20 | C16 | C17 | 120.67(19) | | | | |

# 8. Supplementary NMR Spectra for New Compounds

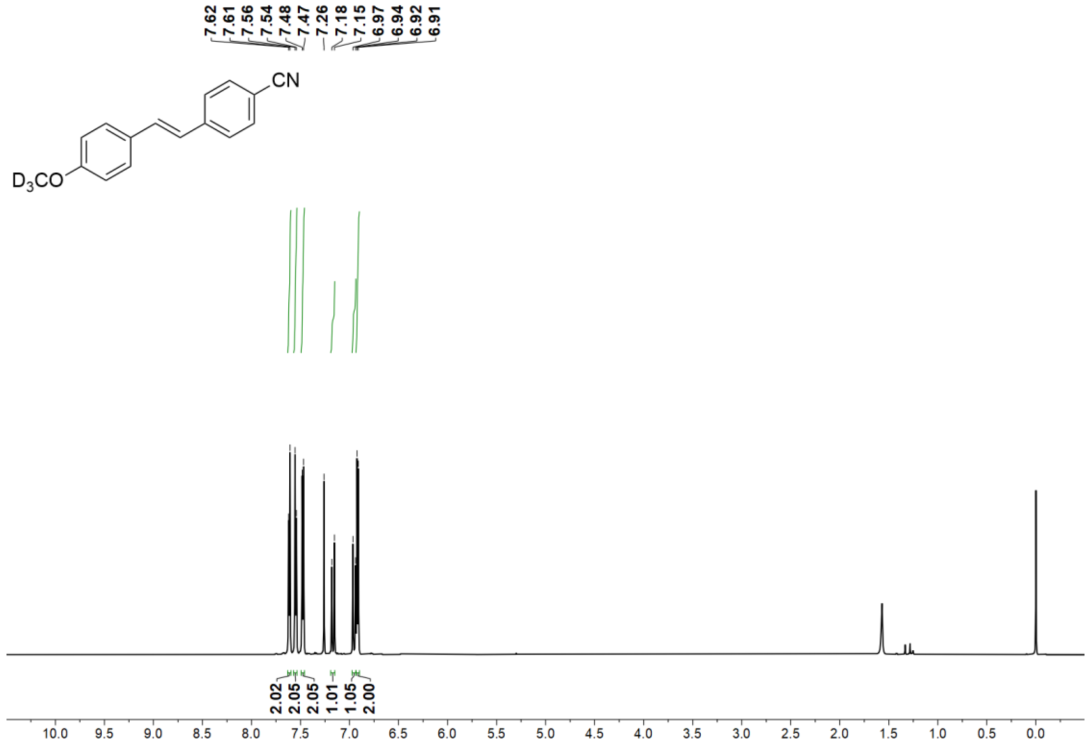


**Supplementary Figure 26.** $^{1}$H NMR (600 MHz, $CDCl_3$) spectrum of compound **1b**

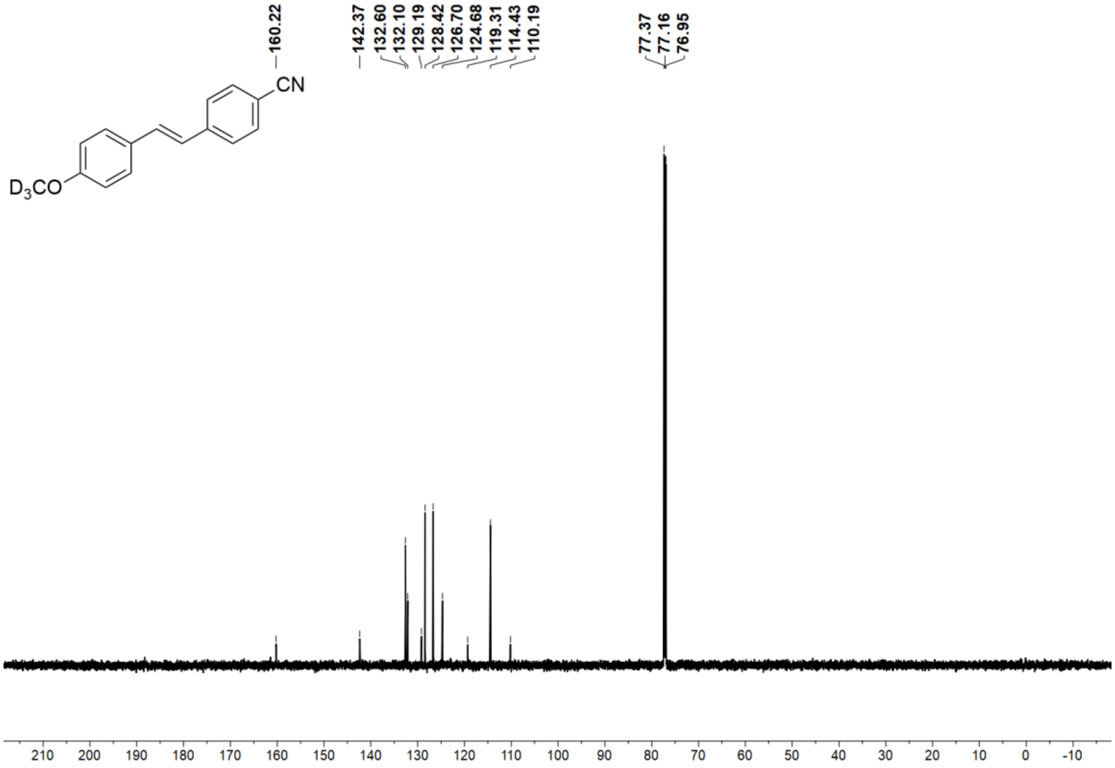


**Supplementary Figure 27.** $^{13}$C NMR (151 MHz, $CDCl_3$) spectrum of compound **1b**

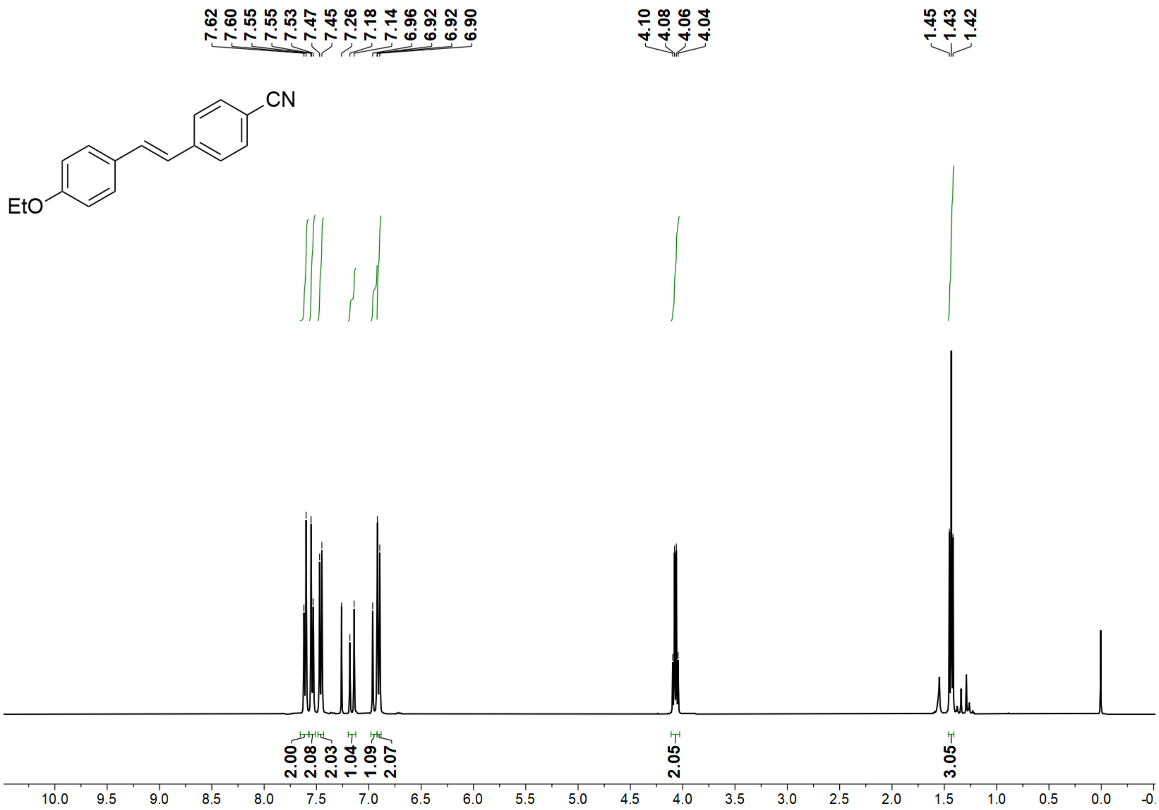


**Supplementary Figure 28.** $^{1}$H NMR (400 MHz, $CDCl_3$) spectrum of compound **1c**

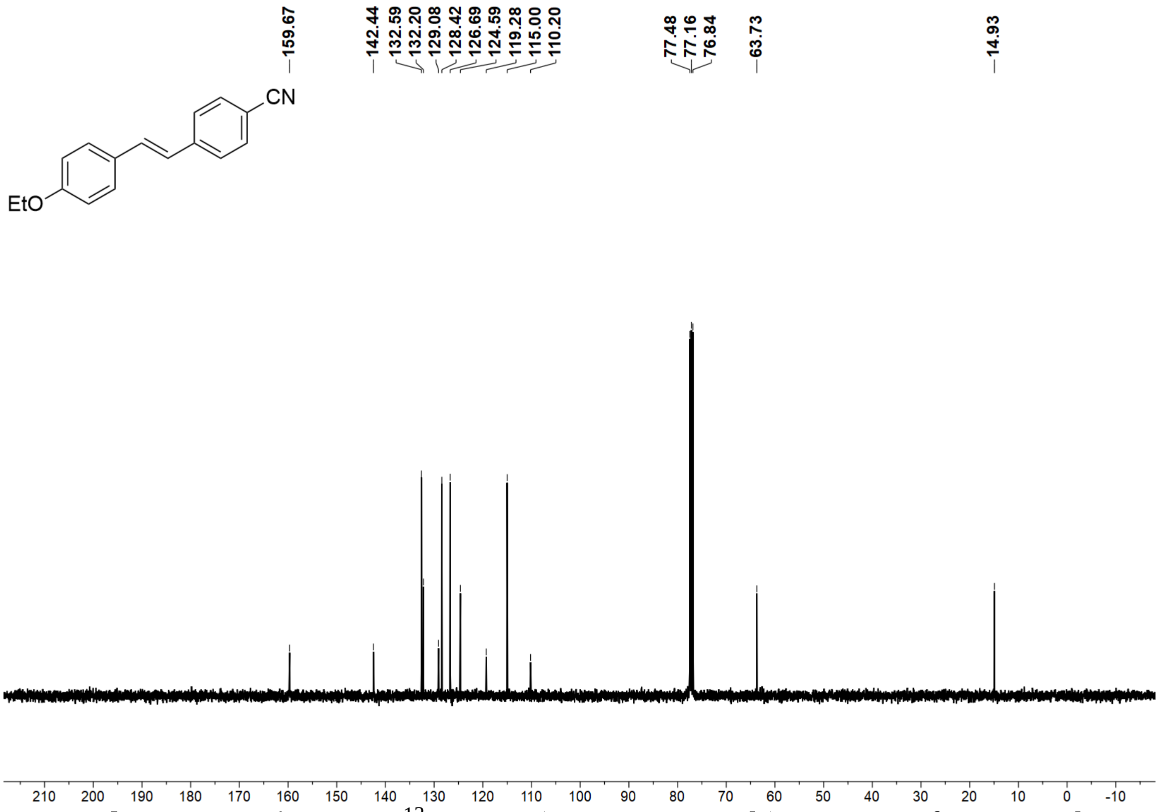


**Supplementary Figure 29.** $^{13}$C NMR (101 MHz, $CDCl_3$) spectrum of compound **1c**

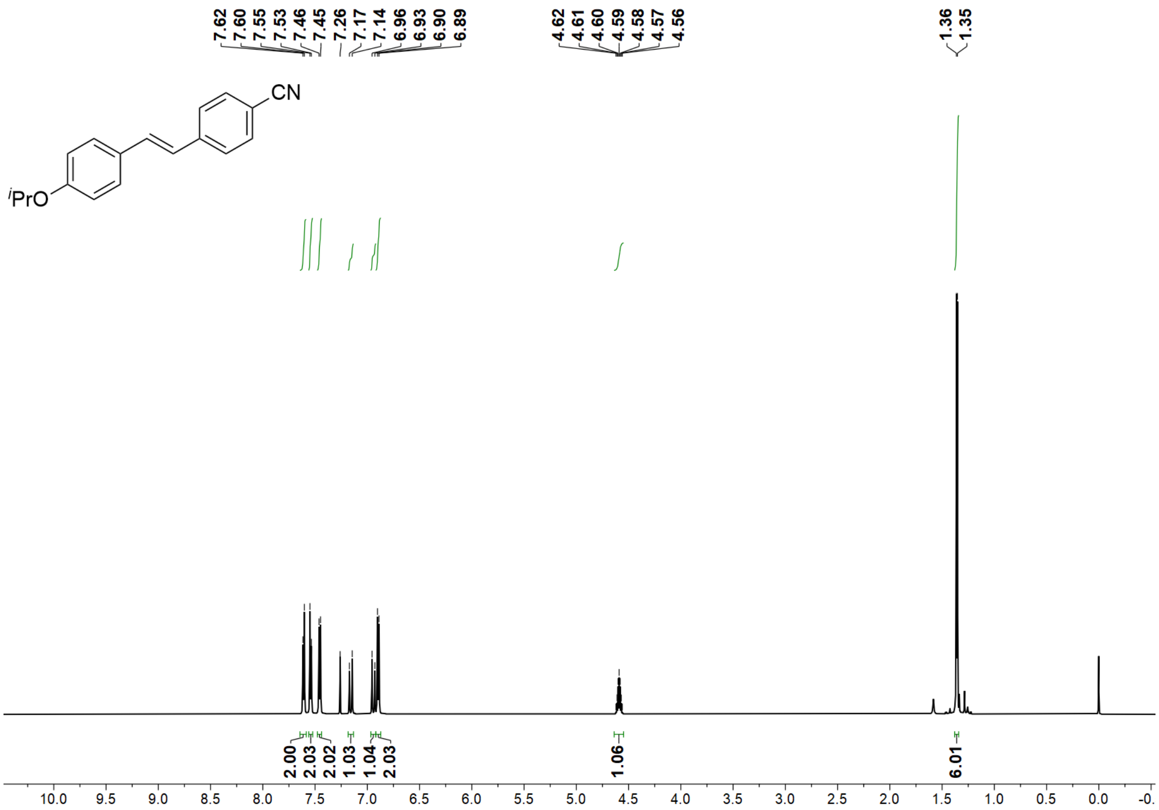


**Supplementary Figure 30.** $^{1}$H NMR (600 MHz, $CDCl_3$) spectrum of compound **1d**

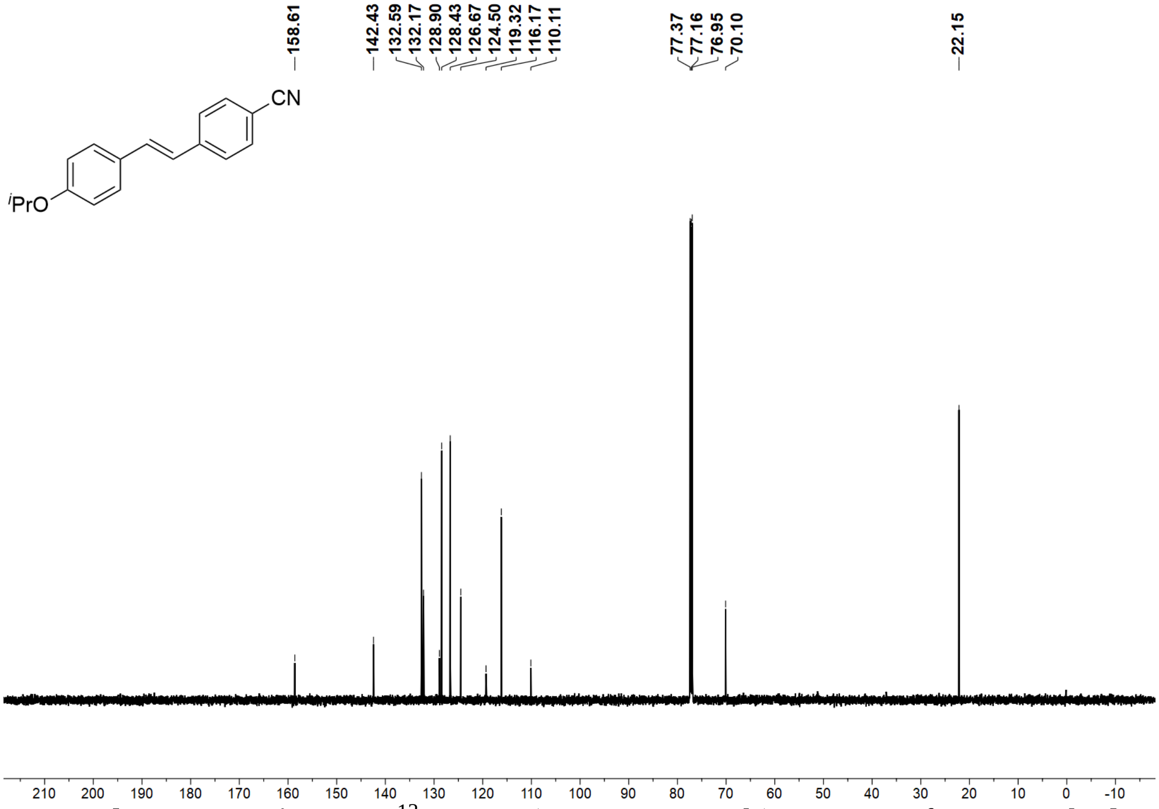


**Supplementary Figure 31.** $^{13}$C NMR (151 MHz, $CDCl_3$) spectrum of compound **1d**

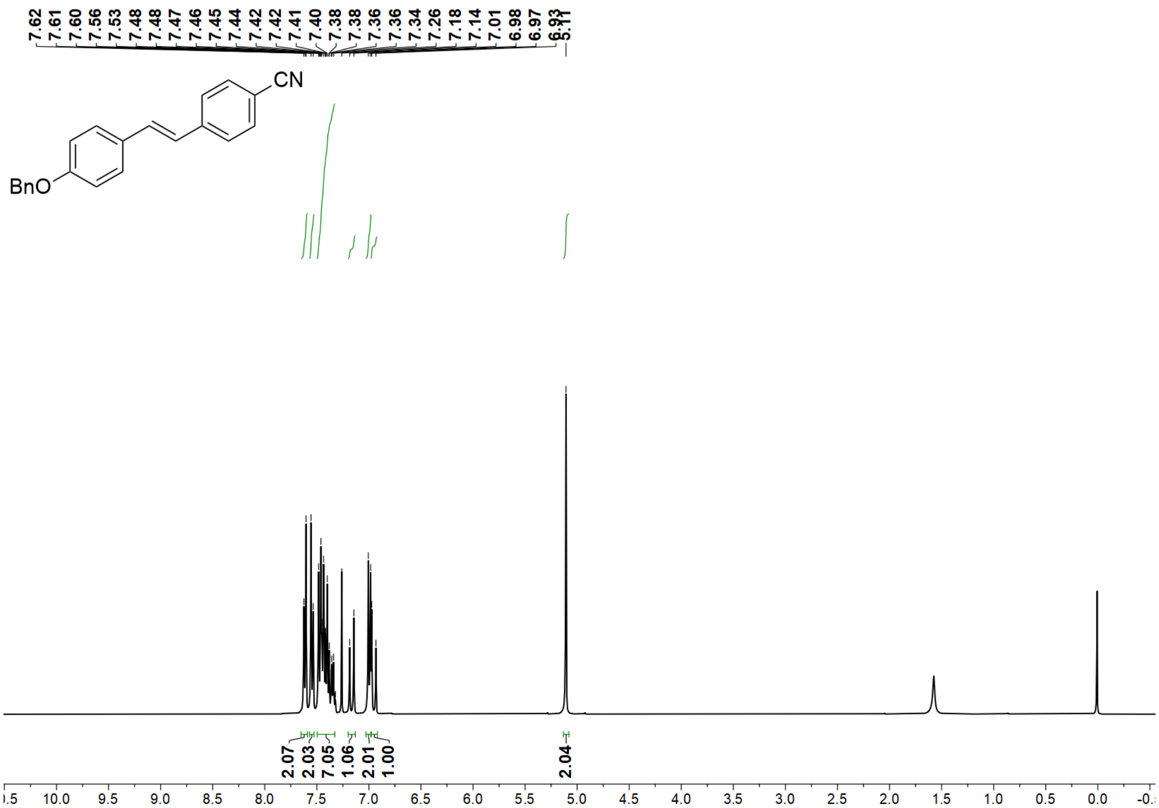


**Supplementary Figure 32.** $^{1}$H NMR (400 MHz, $CDCl_3$) spectrum of compound **1e**

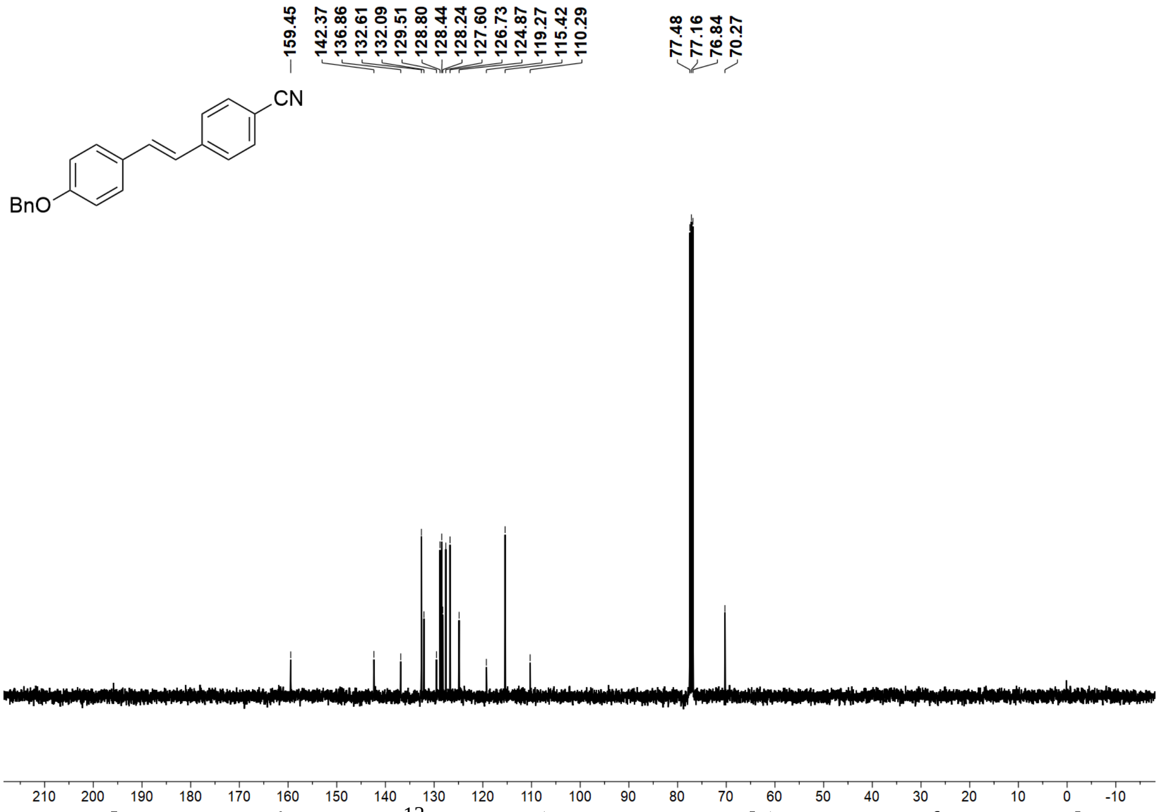


**Supplementary Figure 33.** $^{13}$C NMR (101 MHz, $CDCl_3$) spectrum of compound **1e**

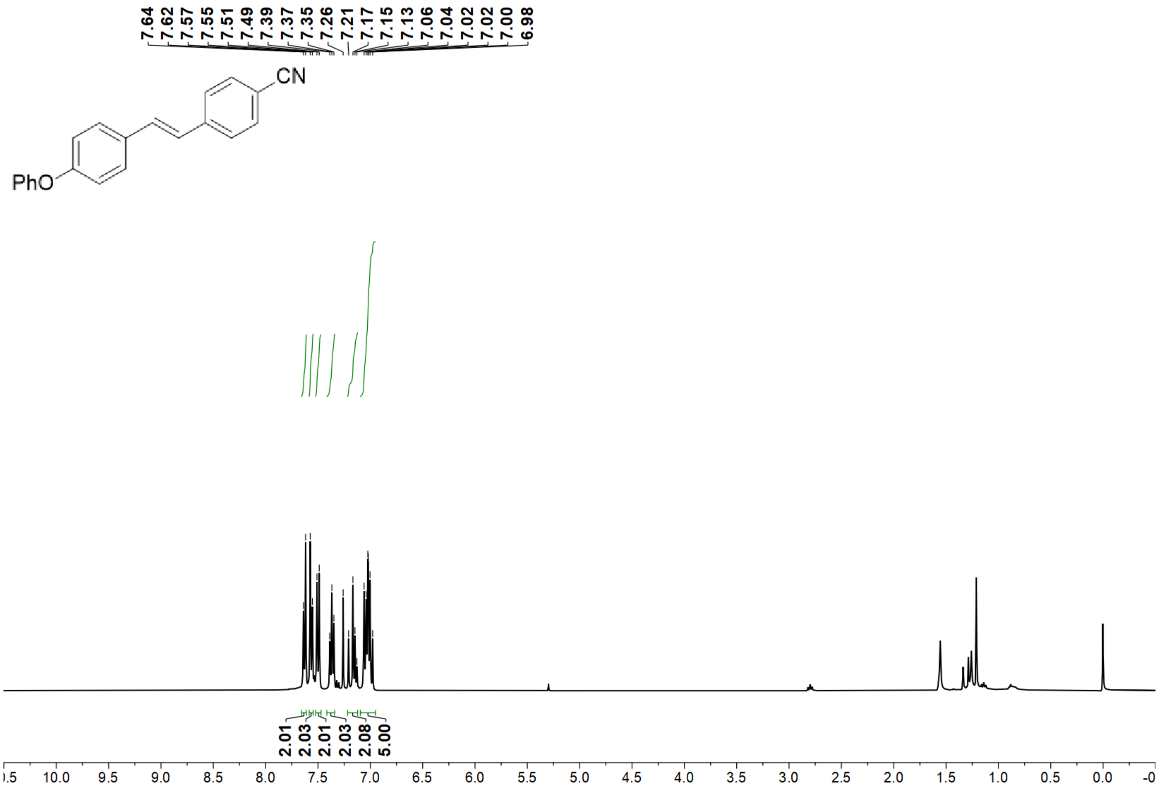


**Supplementary Figure 34.** $^{1}$H NMR (400 MHz, $CDCl_3$) spectrum of compound **1f**

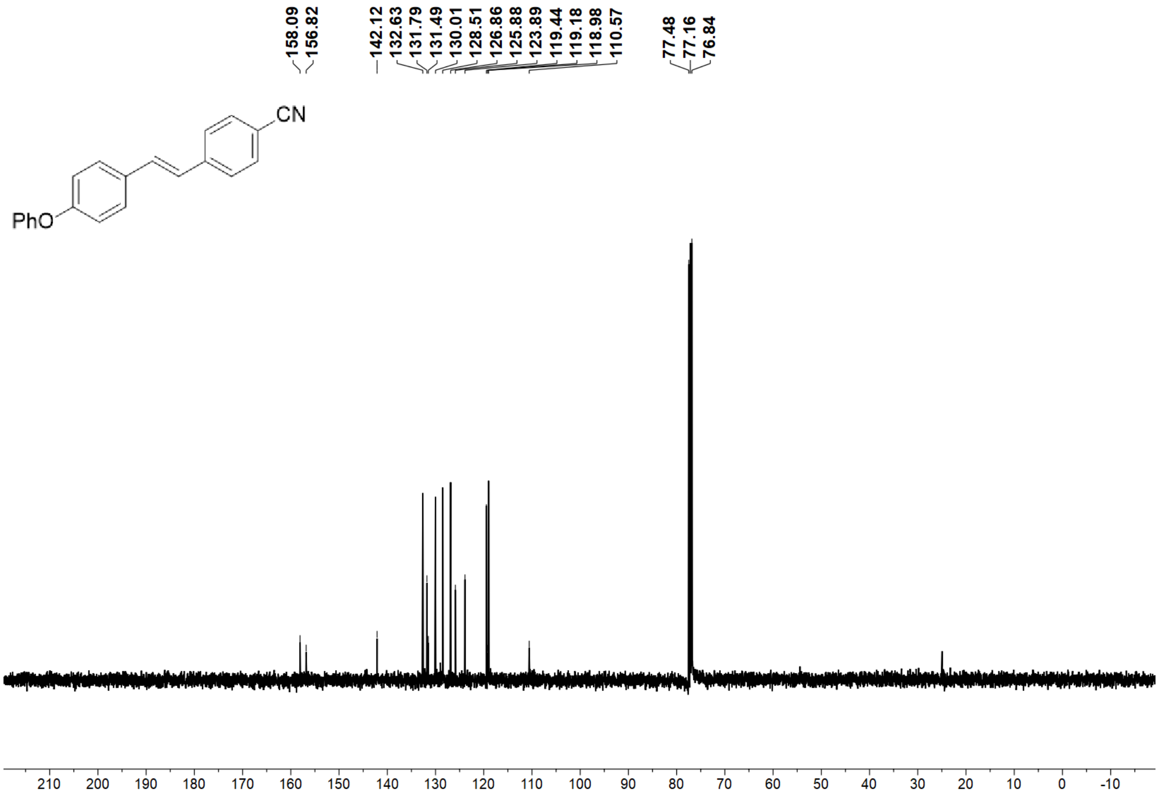


**Supplementary Figure 35.** $^{13}$C NMR (101 MHz, $CDCl_3$) spectrum of compound **1f**

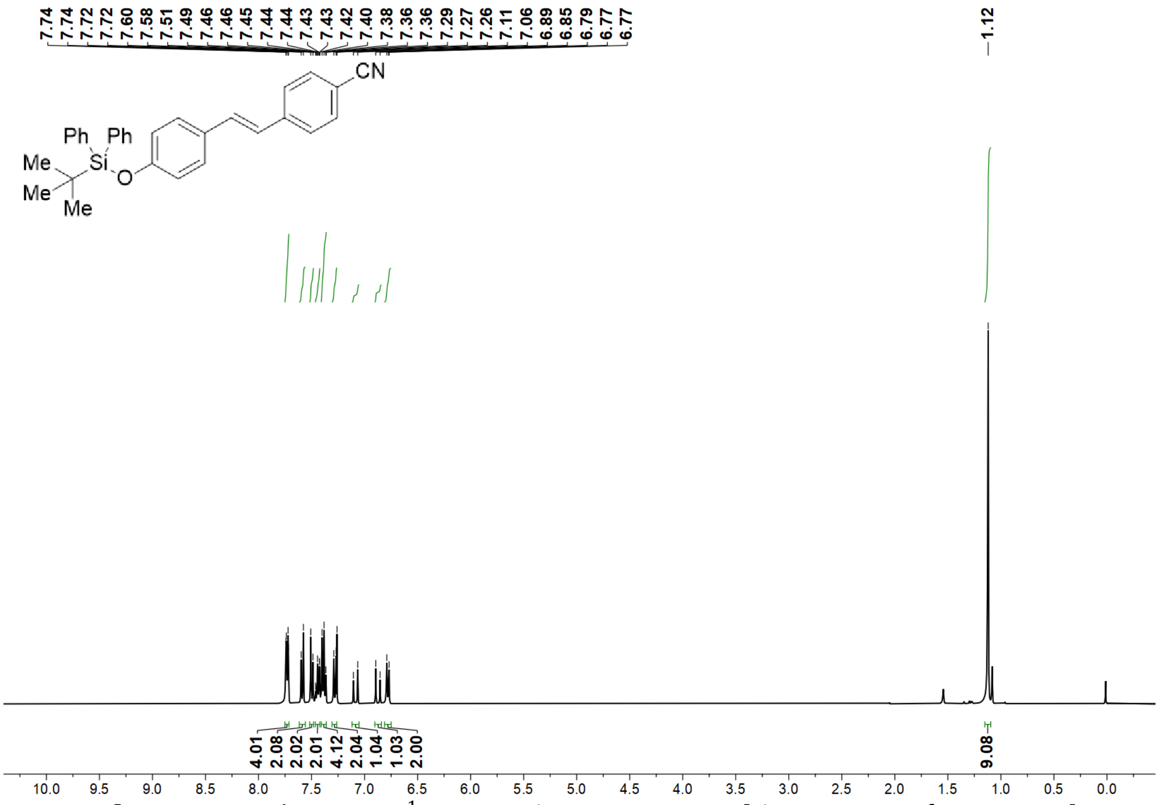

**Supplementary Figure 36.** $^{1}H$ NMR (400 MHz, $CDCl_3$) spectrum of compound **1g**

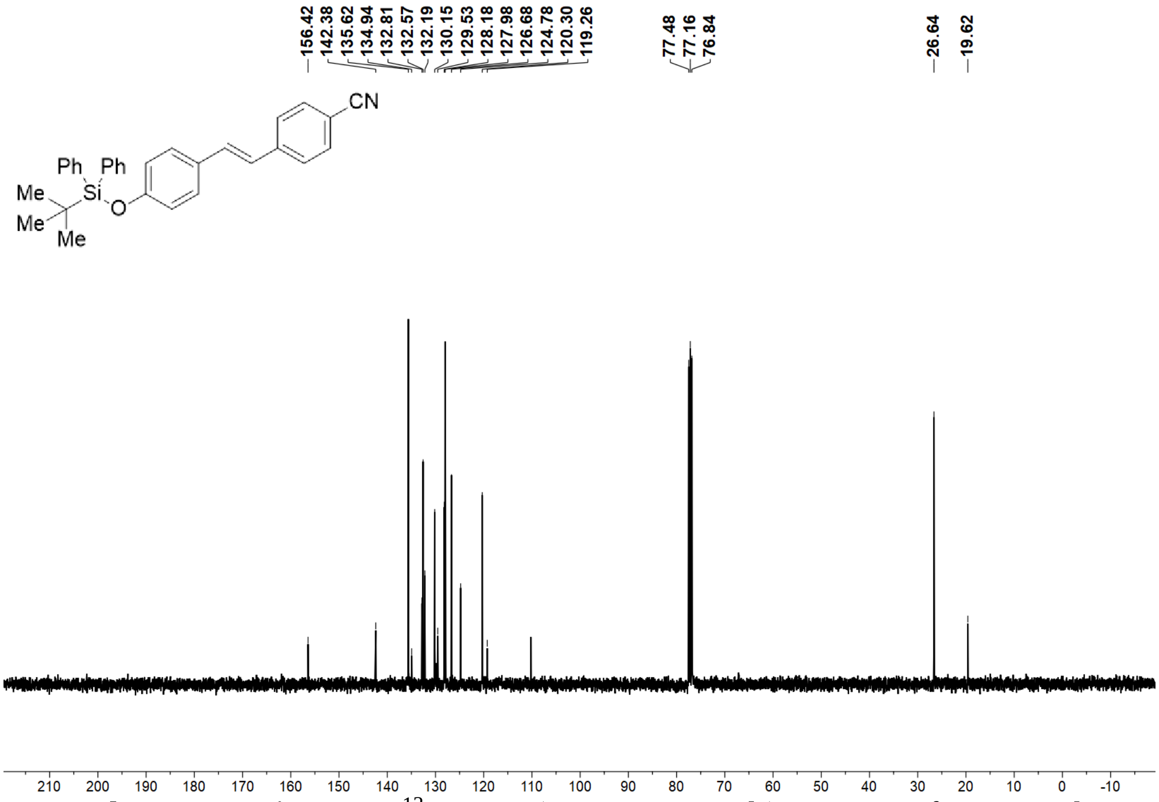

**Supplementary Figure 37.** $^{13}C$ NMR (101 MHz, $CDCl_3$) spectrum of compound **1g**

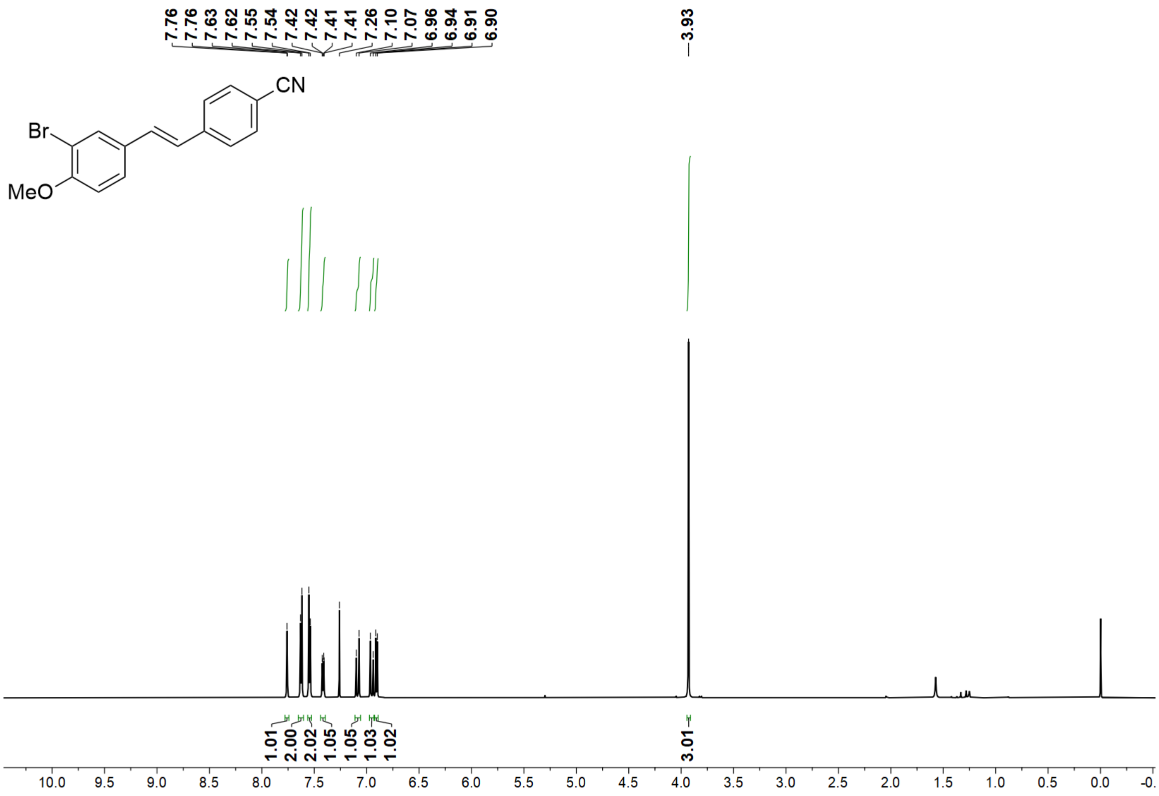


**Supplementary Figure 38.** $^{1}$H NMR (600 MHz, $CDCl_3$) spectrum of compound **1i**

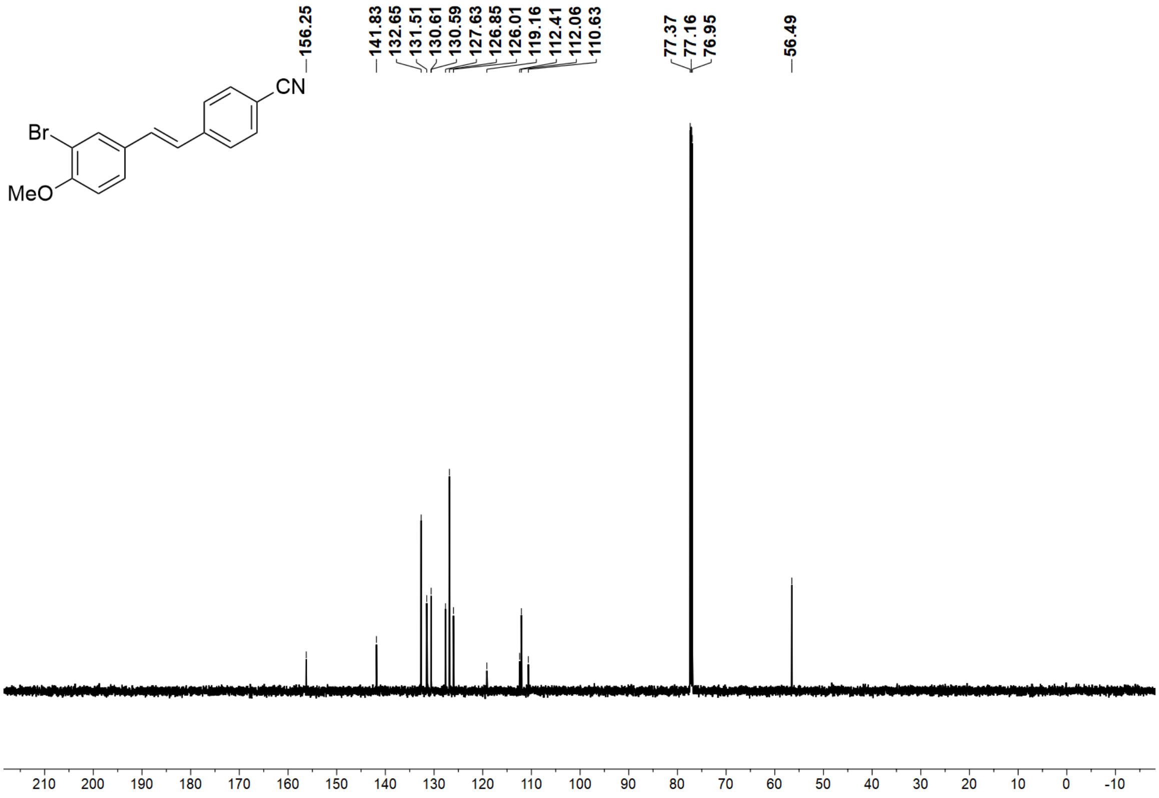


**Supplementary Figure 39.** $^{13}$C NMR (151 MHz, $CDCl_3$) spectrum of compound **1i**

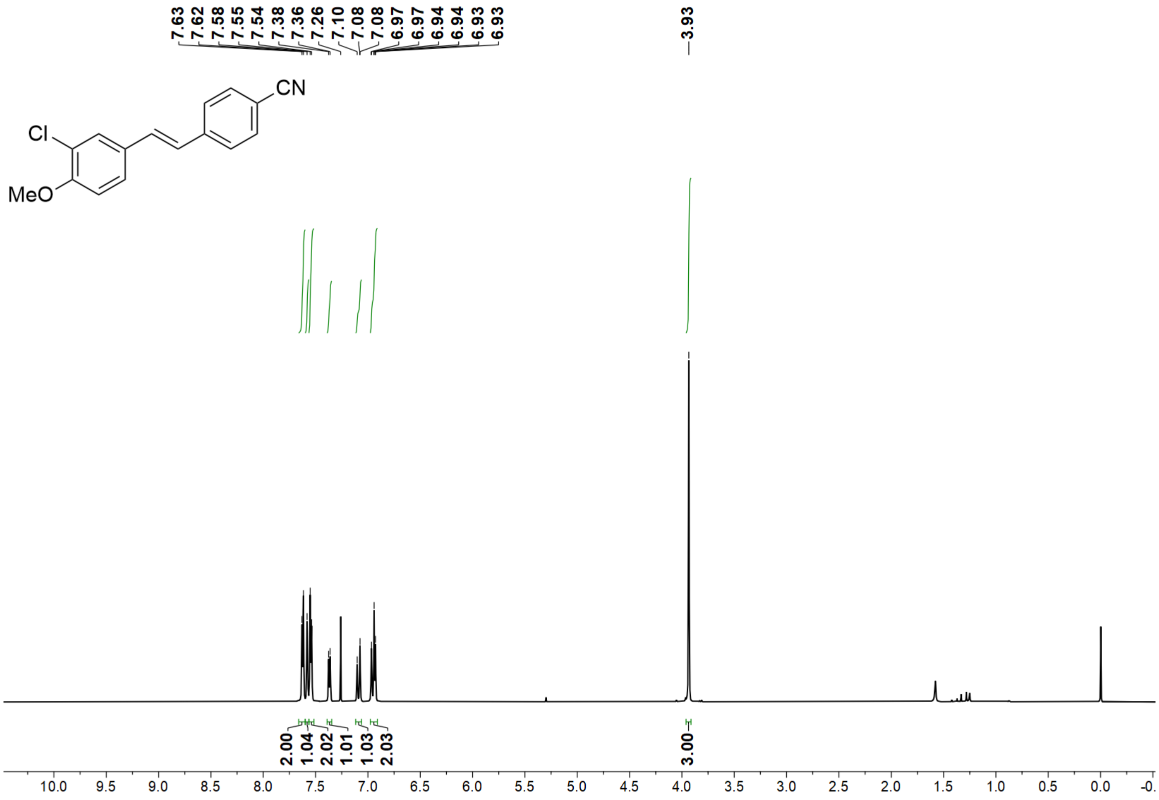


**Supplementary Figure 40.** $^{1}$H NMR (600 MHz, $CDCl_3$) spectrum of compound **1j**

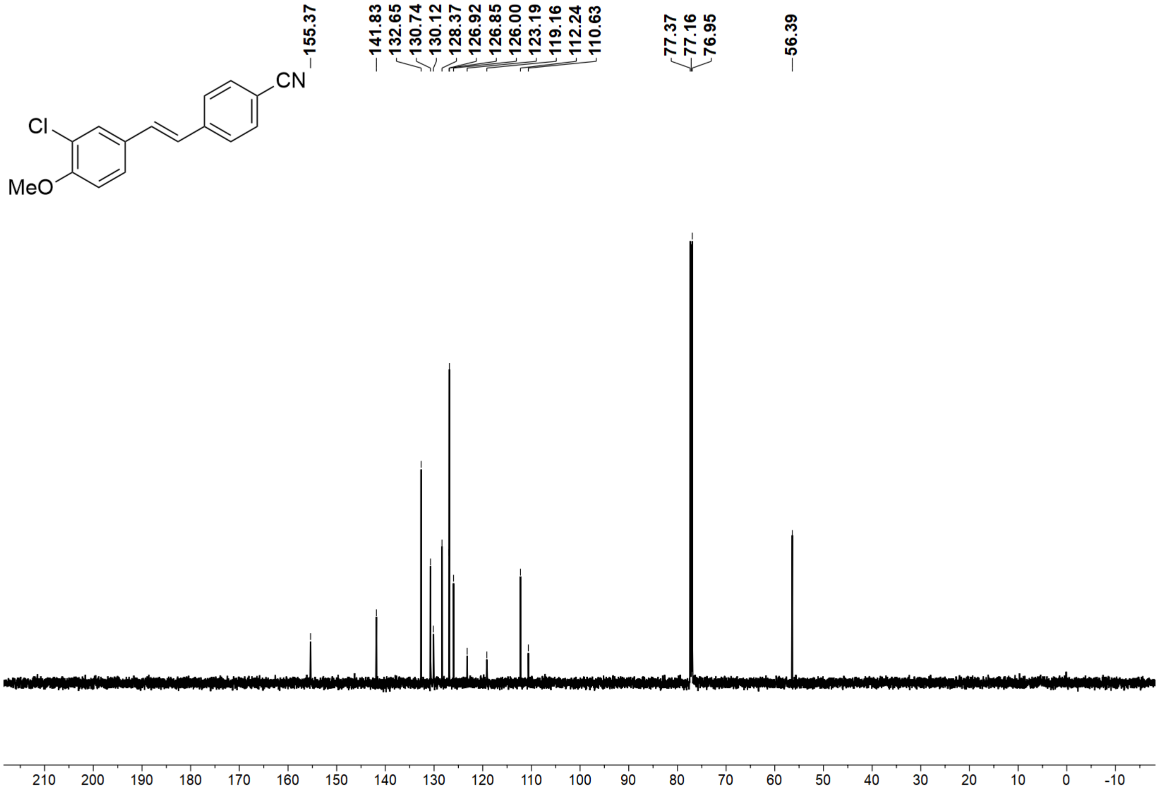


**Supplementary Figure 41.** $^{13}$C NMR (151 MHz, $CDCl_3$) spectrum of compound **1j**

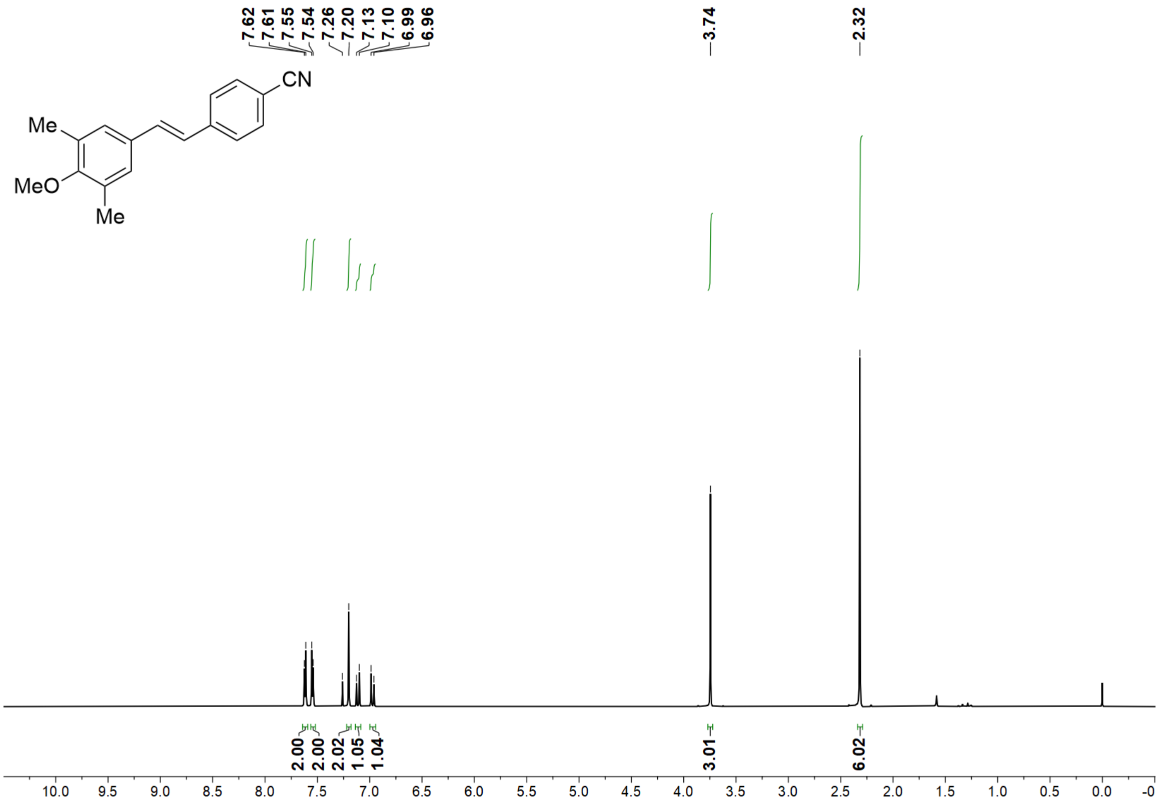

**Supplementary Figure 42.** $^{1}$H NMR (600 MHz, $CDCl_3$) spectrum of compound **1l**

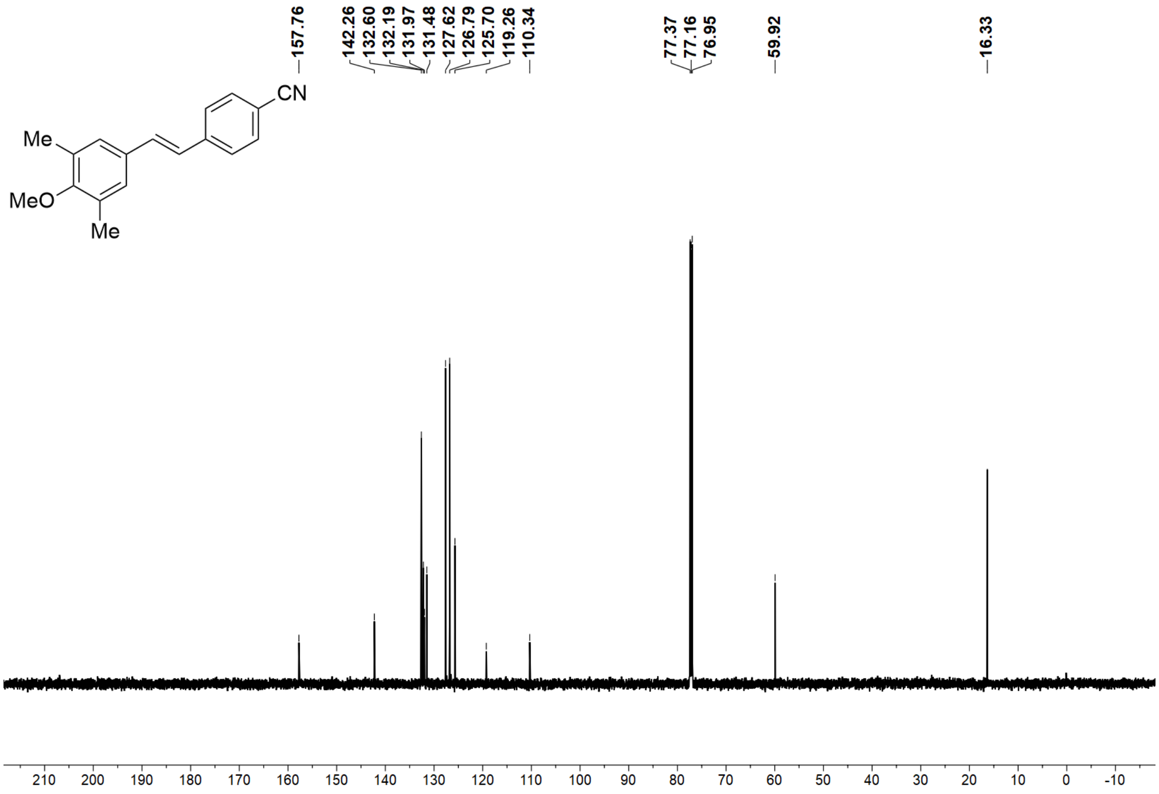

**Supplementary Figure 43.** $^{13}$C NMR (151 MHz, $CDCl_3$) spectrum of compound **1l**

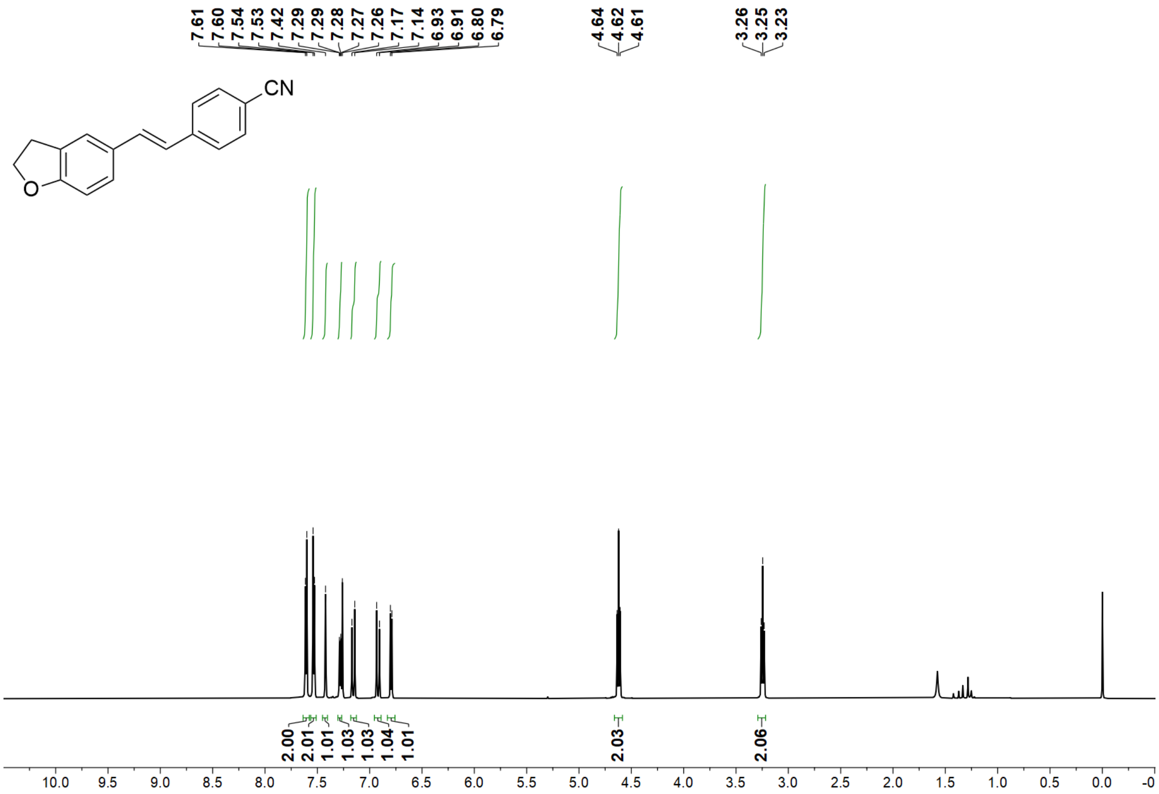


**Supplementary Figure 44.** $^{1}$H NMR (600 MHz, $CDCl_3$) spectrum of compound **1m**

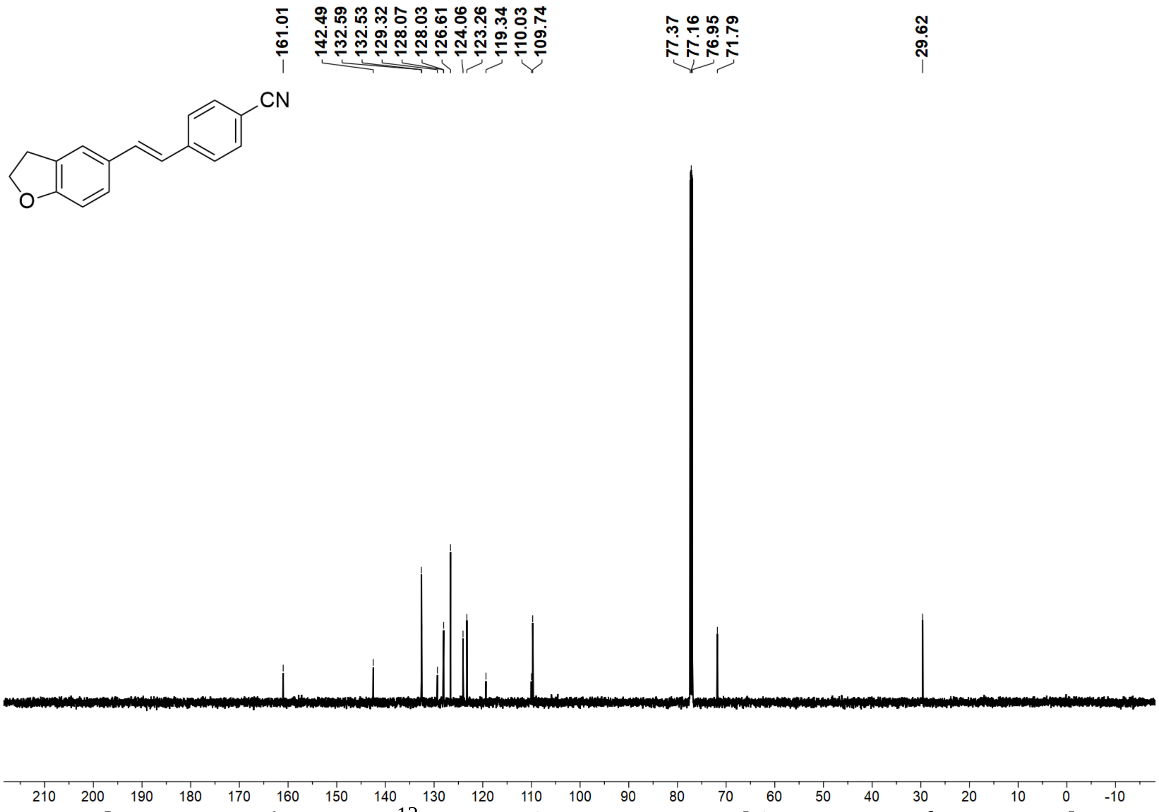


**Supplementary Figure 45.** $^{13}$C NMR (151 MHz, $CDCl_3$) spectrum of compound **1m**

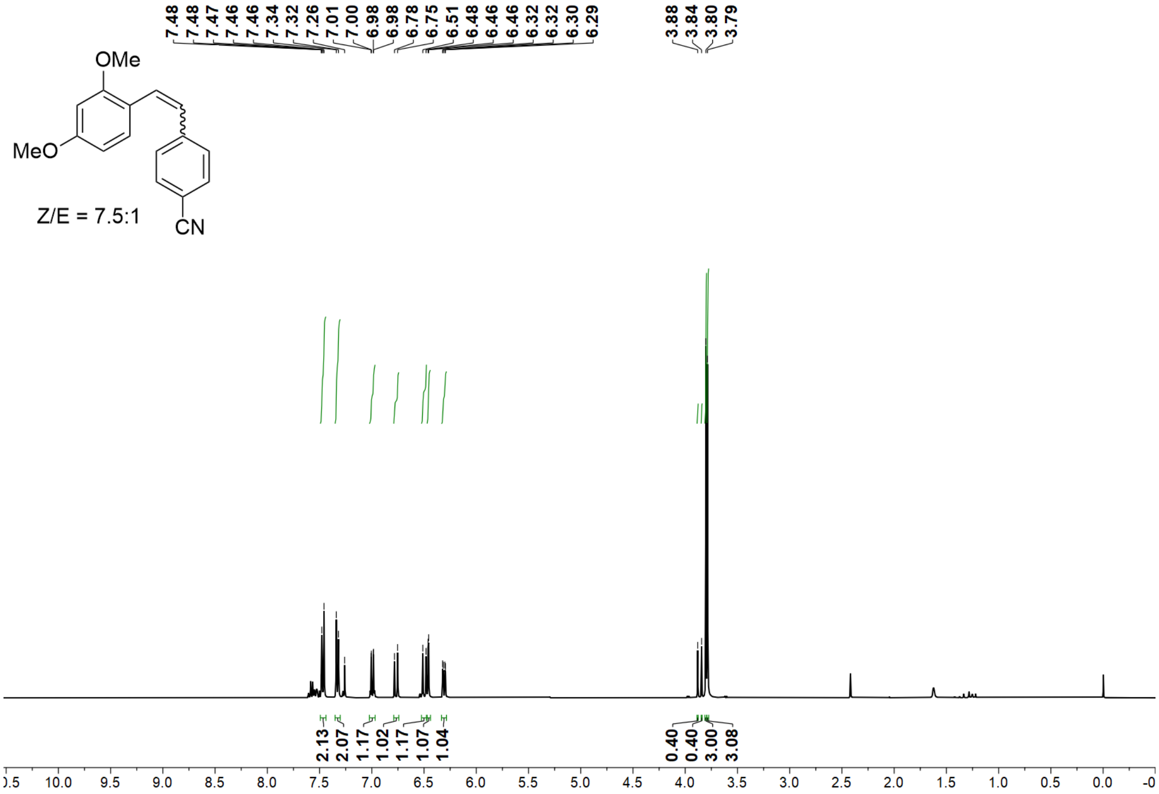


**Supplementary Figure 46.** $^{1}H$ NMR (400 MHz, $CDCl_3$) spectrum of compound **1o**

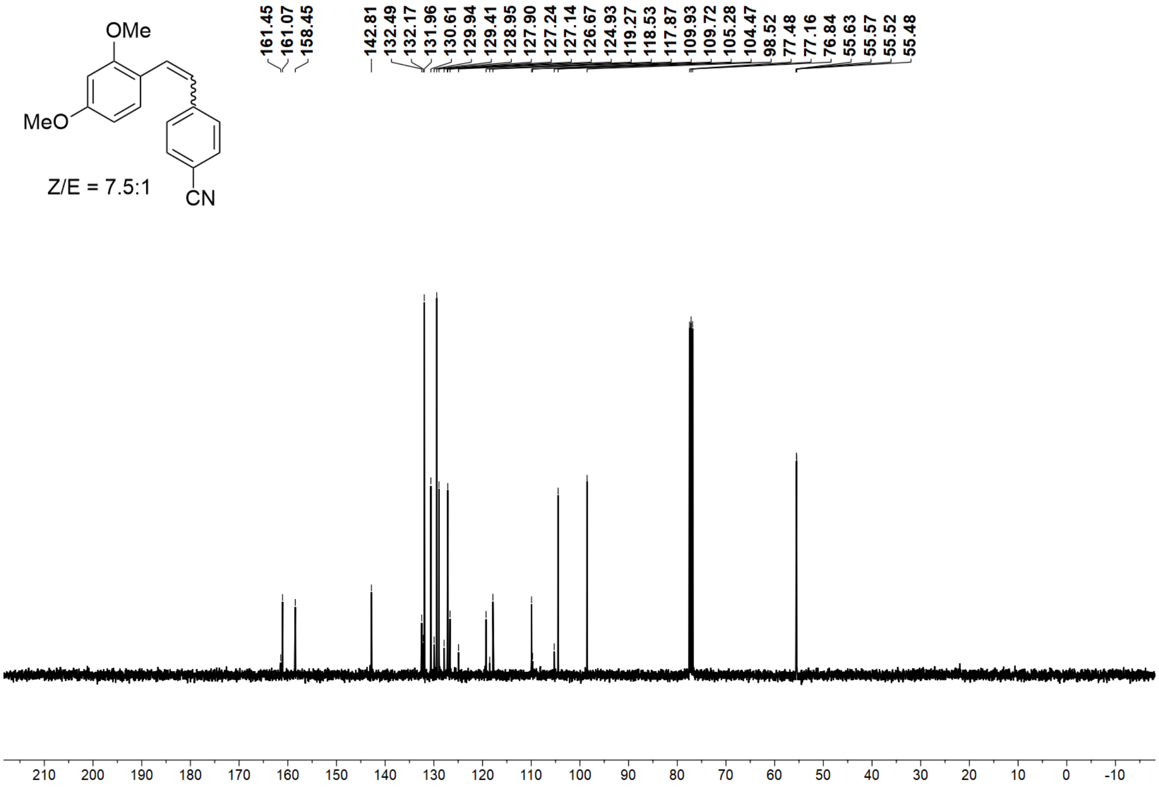


**Supplementary Figure 47.** $^{13}C$ NMR (101 MHz, $CDCl_3$) spectrum of compound **1o**

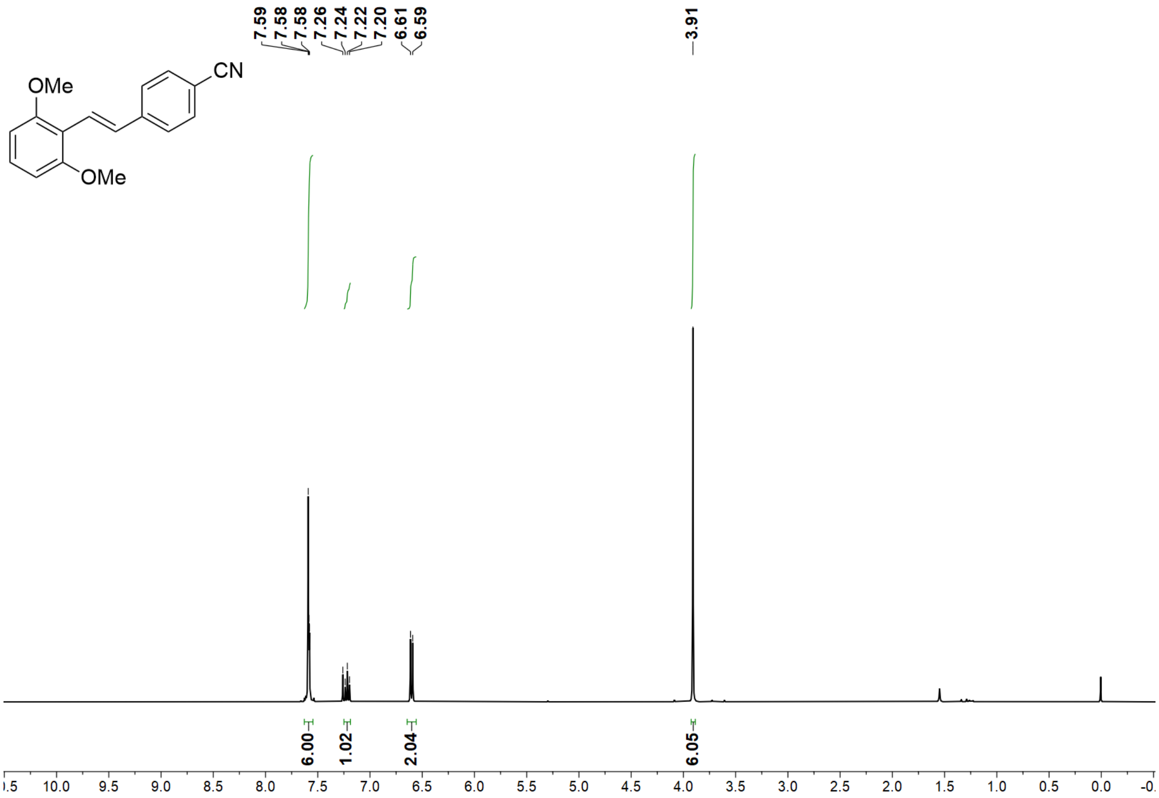


**Supplementary Figure 48.** $^{1}H$ NMR (400 MHz, $CDCl_3$) spectrum of compound **1p**

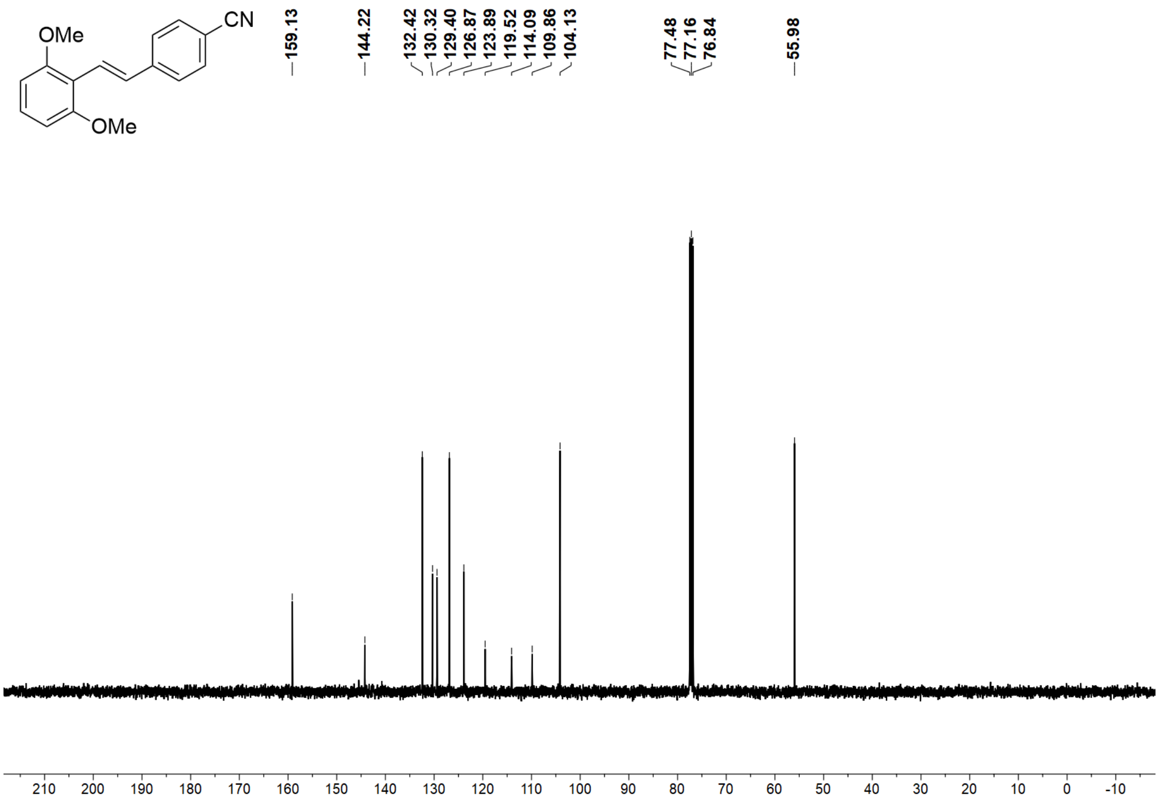


**Supplementary Figure 49.** $^{13}C$ NMR (101 MHz, $CDCl_3$) spectrum of compound **1p**

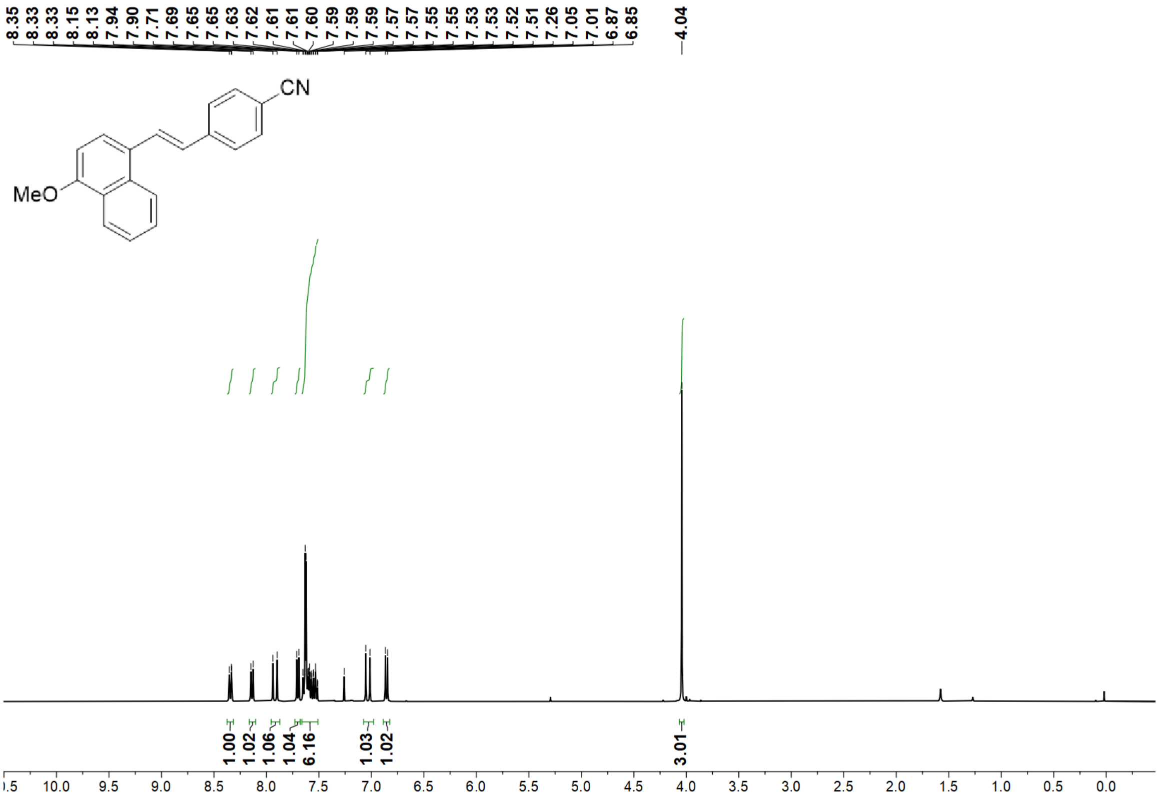


**Supplementary Figure 50.** $^{1}H$ NMR (400 MHz, $CDCl_3$) spectrum of compound **1q**

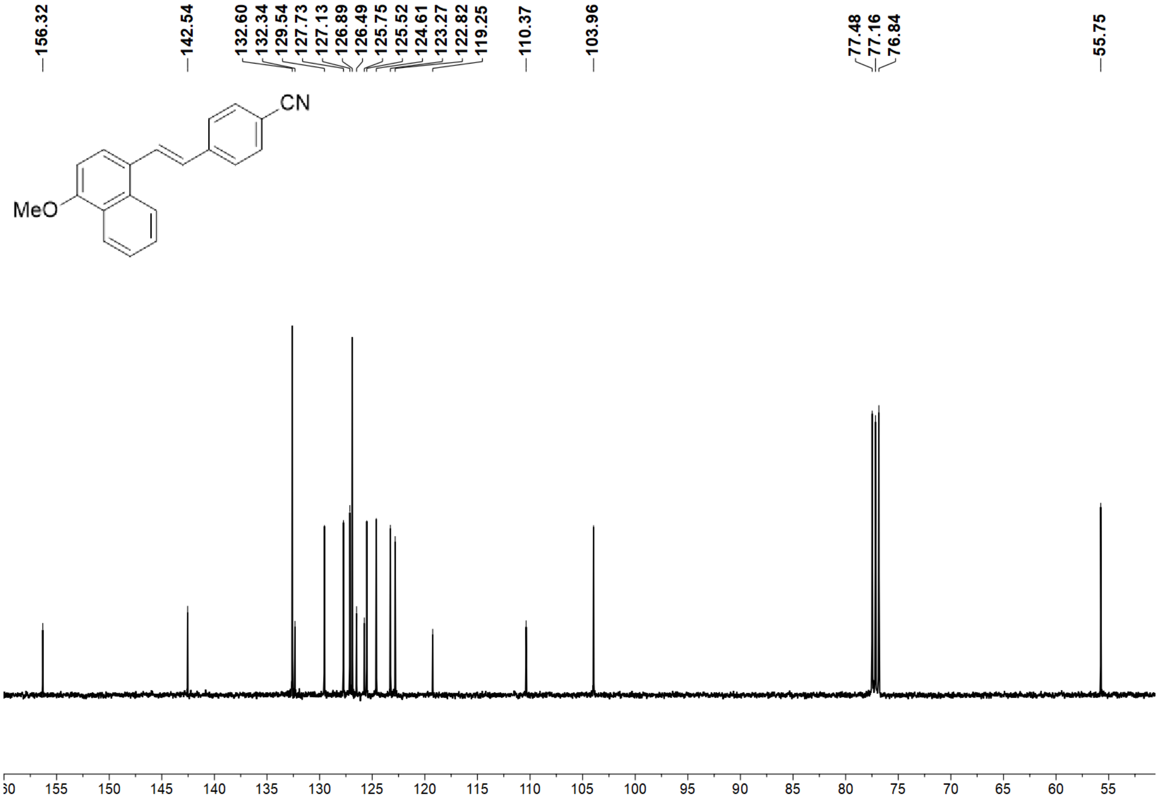


**Supplementary Figure 51.** $^{13}C$ NMR (101 MHz, $CDCl_3$) spectrum of compound **1p**

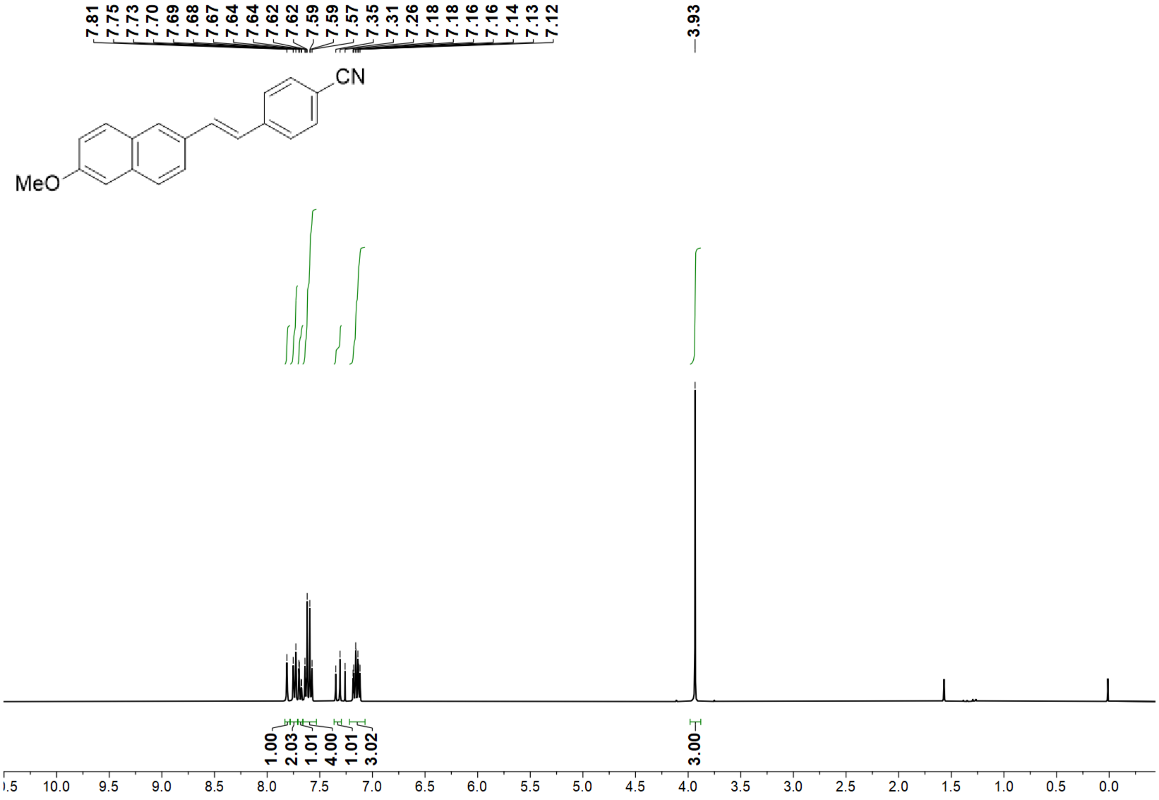


**Supplementary Figure 52.** $^{1}$H NMR (400 MHz, $CDCl_3$) spectrum of compound **1r**

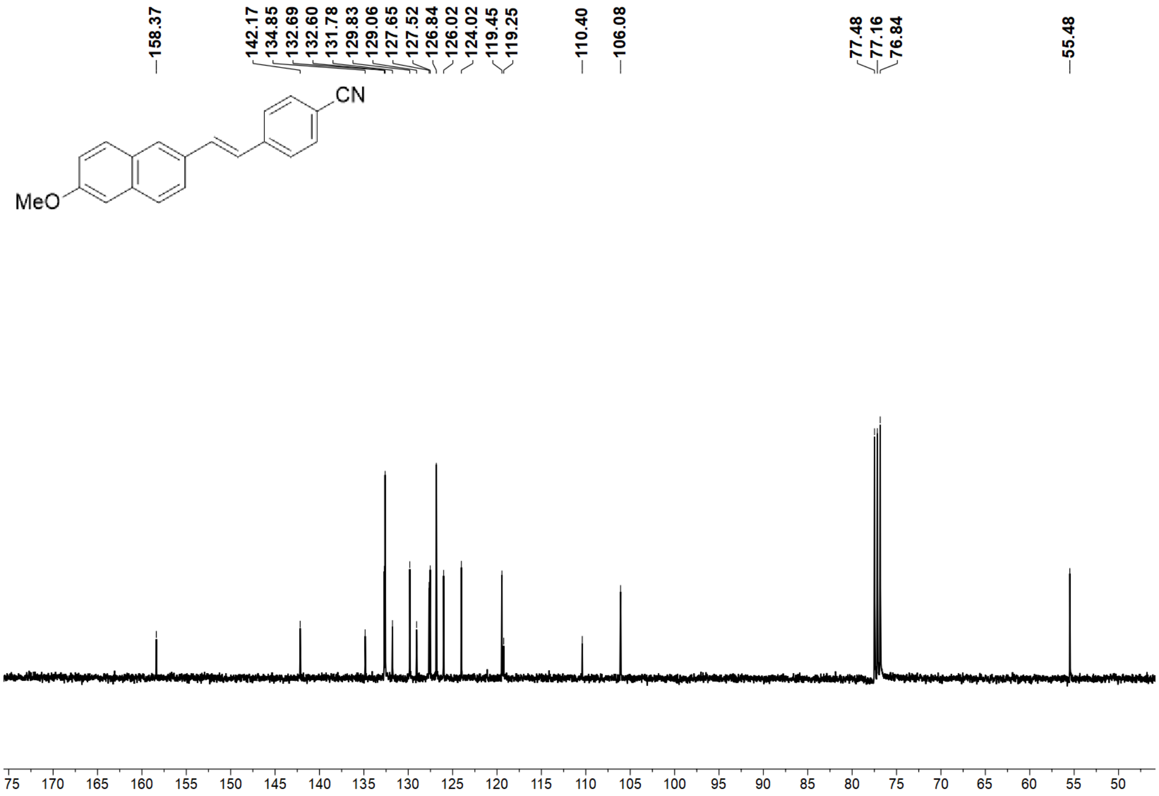


**Supplementary Figure 53.** $^{13}$C NMR (101 MHz, $CDCl_3$) spectrum of compound **1r**

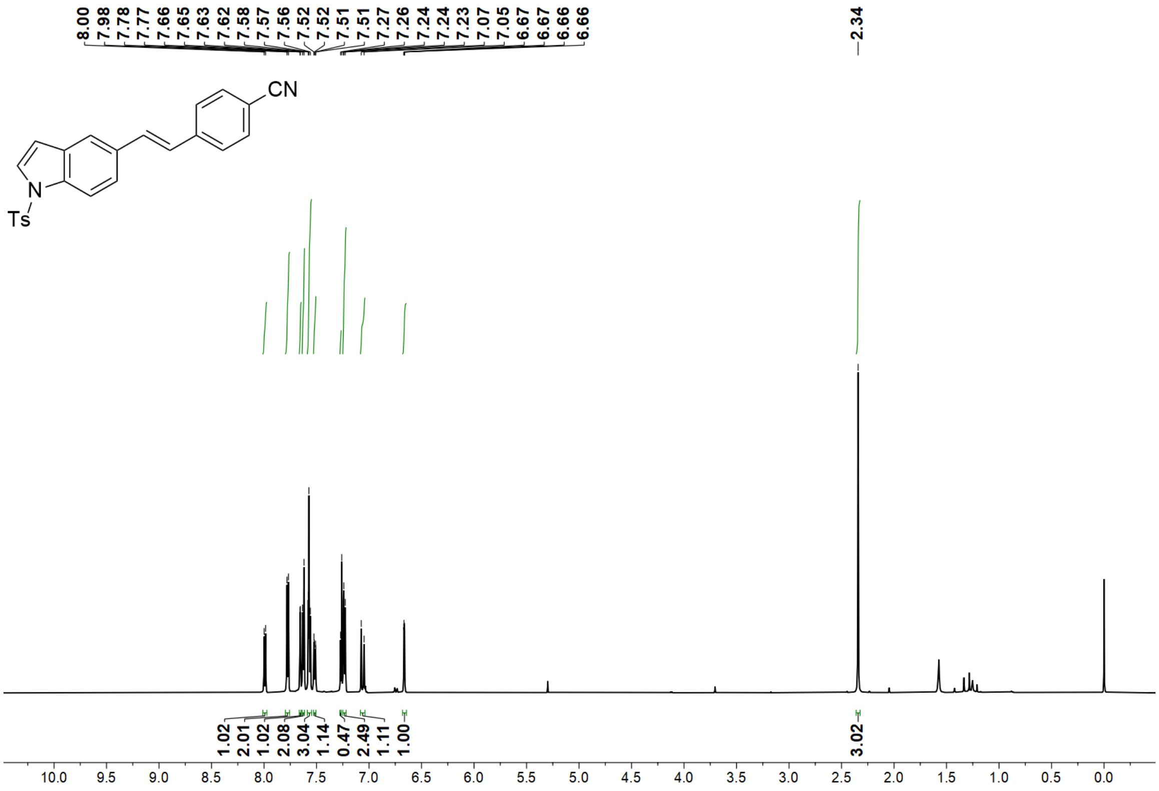


**Supplementary Figure 54.** $^{1}$H NMR (600 MHz, $CDCl_3$) spectrum of compound **1s**

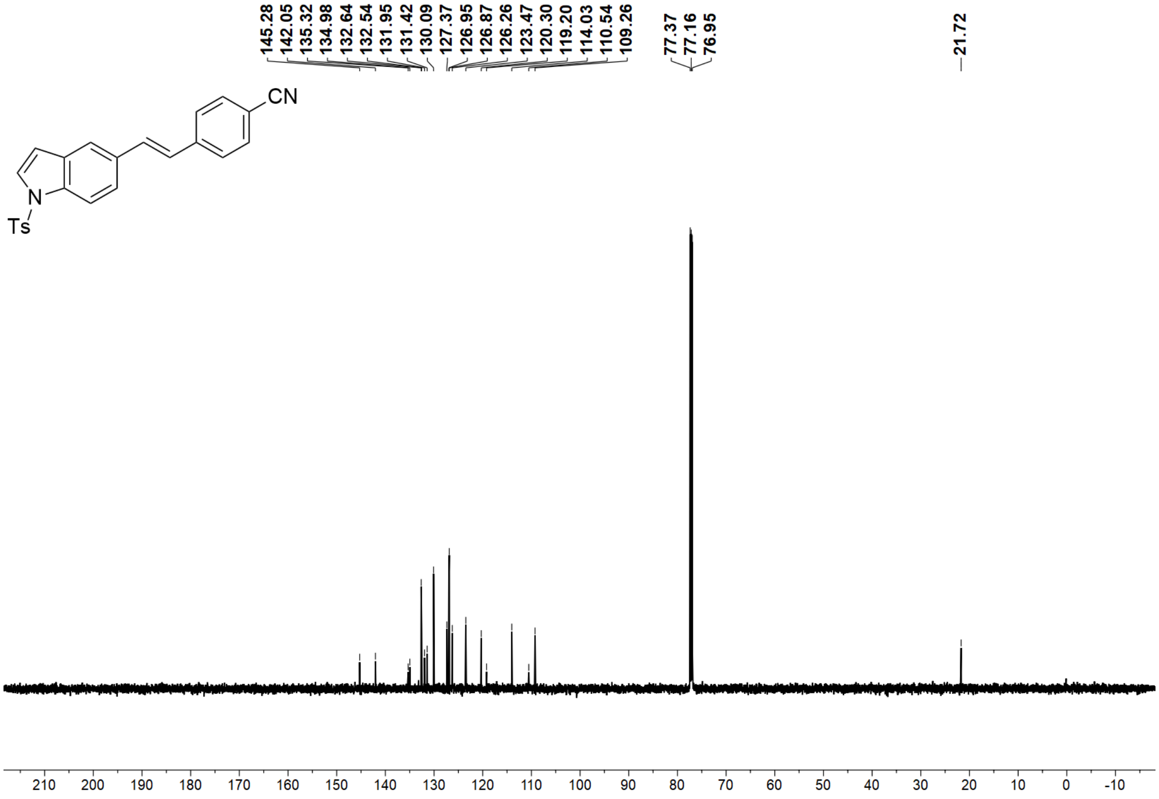


**Supplementary Figure 55.** $^{13}$C NMR (151 MHz, $CDCl_3$) spectrum of compound **1s**

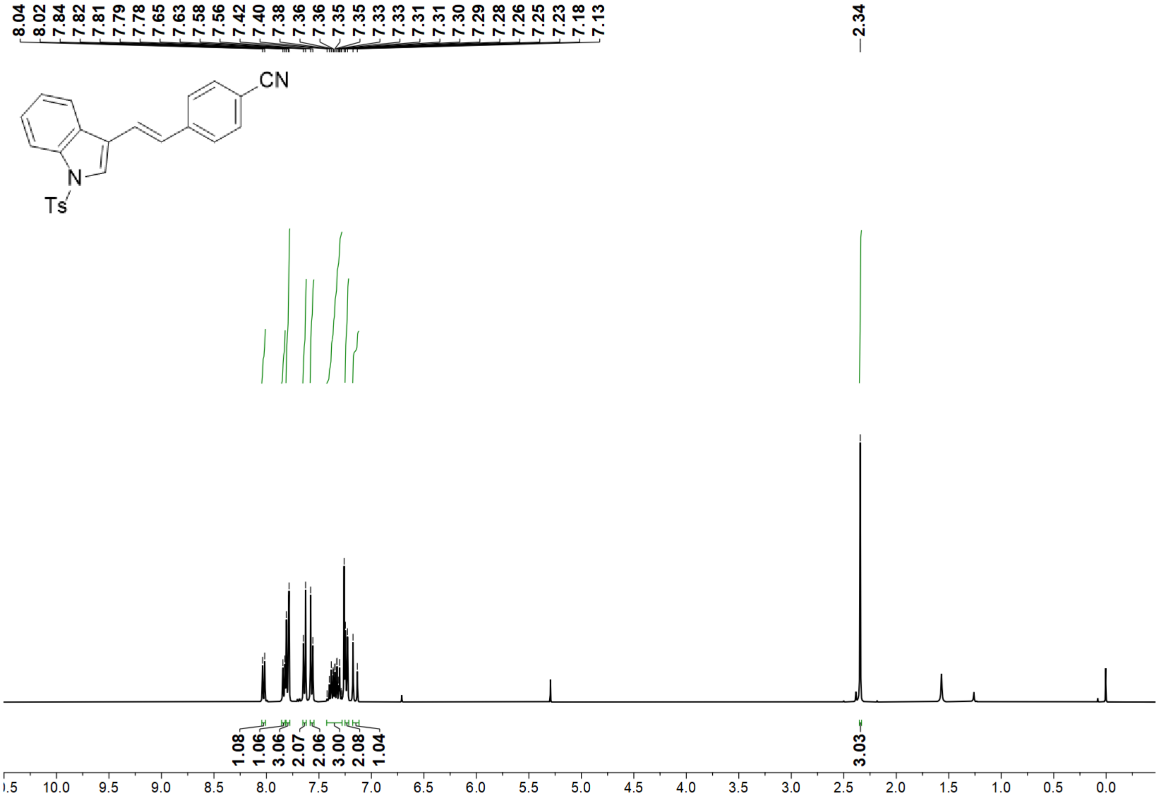


**Supplementary Figure 56.** $^{1}$H NMR (400 MHz, $CDCl_3$) spectrum of compound **1t**

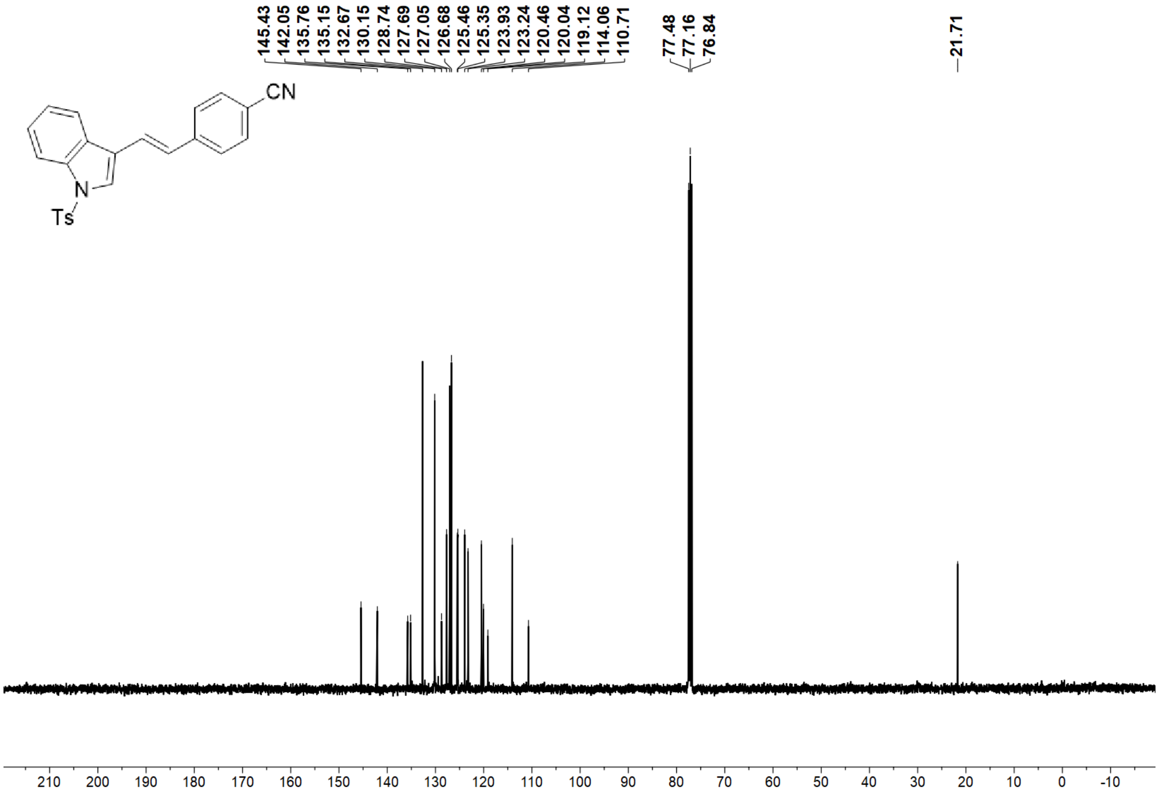


**Supplementary Figure 57.** $^{13}$C NMR (101 MHz, $CDCl_3$) spectrum of compound **1t**

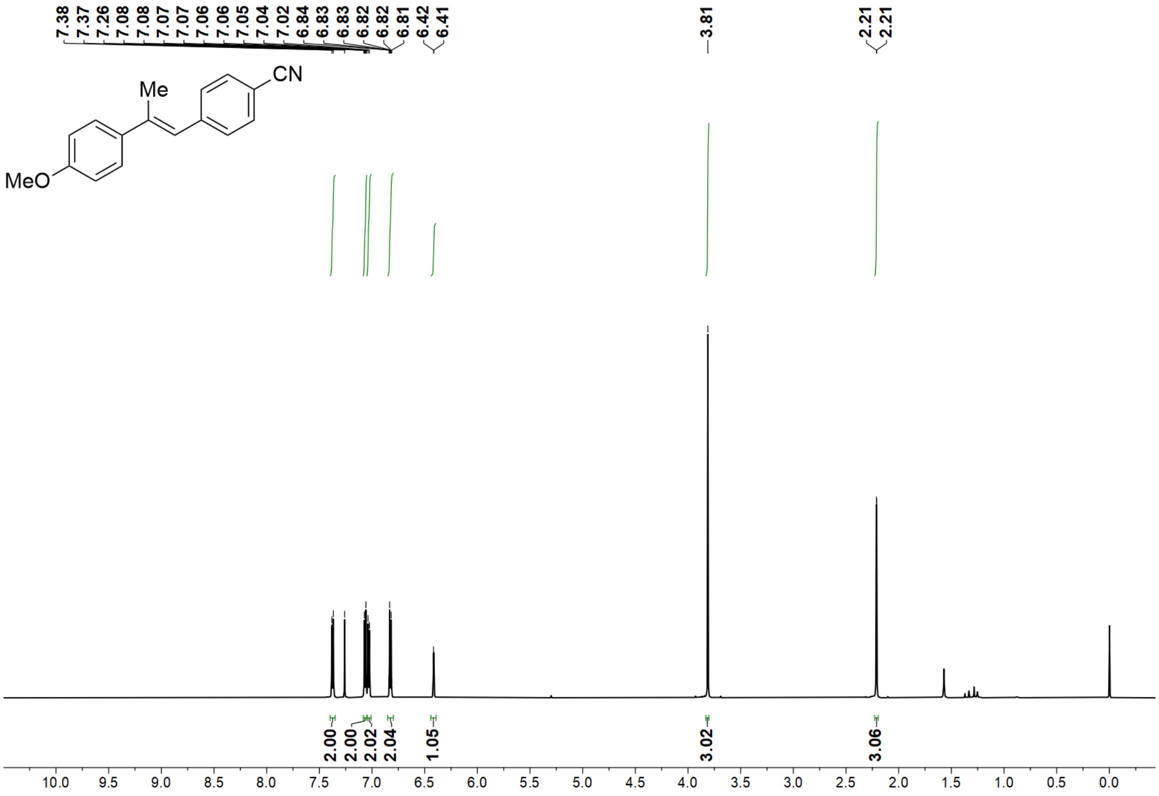

**Supplementary Figure 58.** $^{1}$H NMR (600 MHz, $CDCl_3$) spectrum of compound **1v**

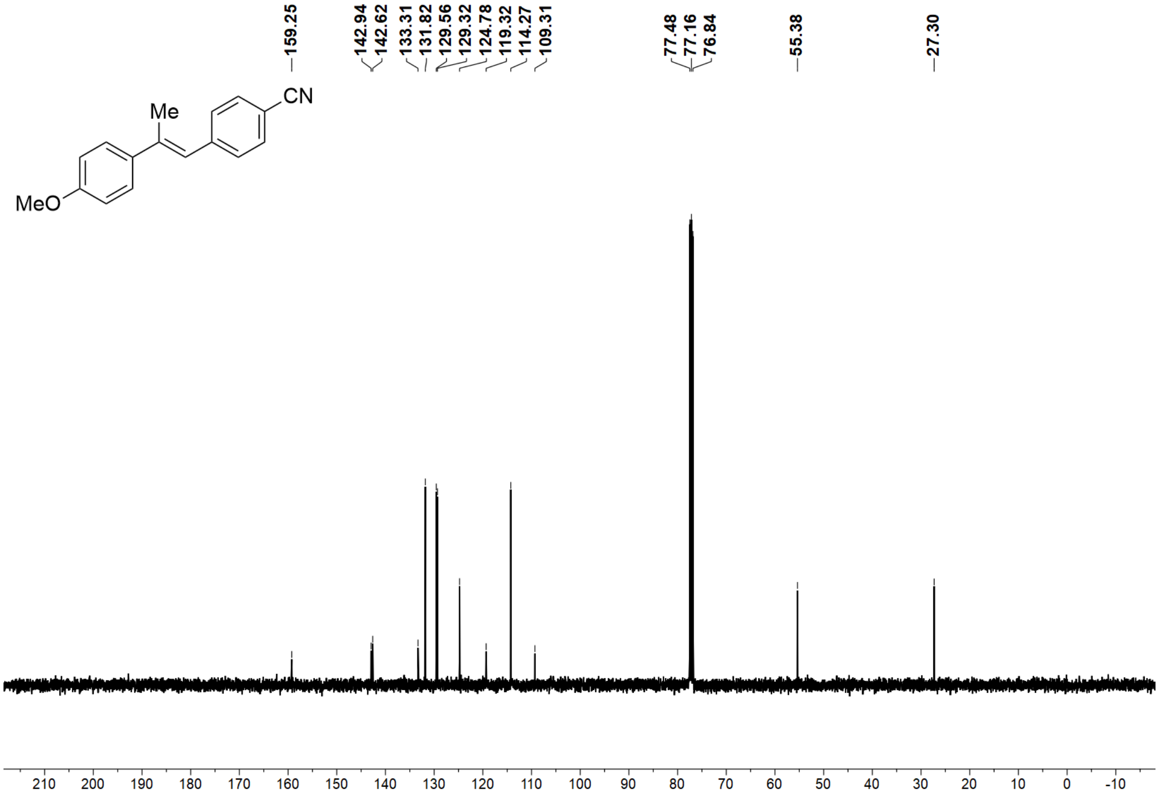

**Supplementary Figure 59.** $^{13}$C NMR (151 MHz, $CDCl_3$) spectrum of compound **1v**

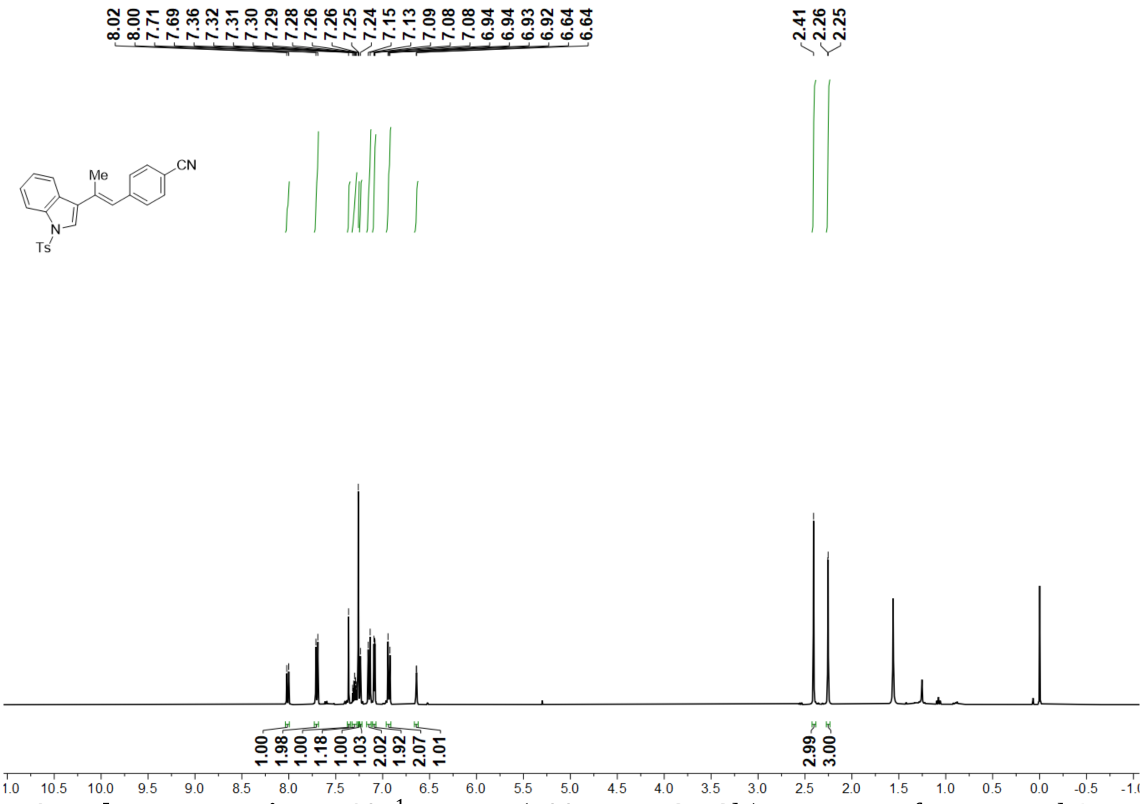

Supplementary Figure 60. $^1$H NMR (400 MHz, $CDCl_3$) spectrum of compound **1w**

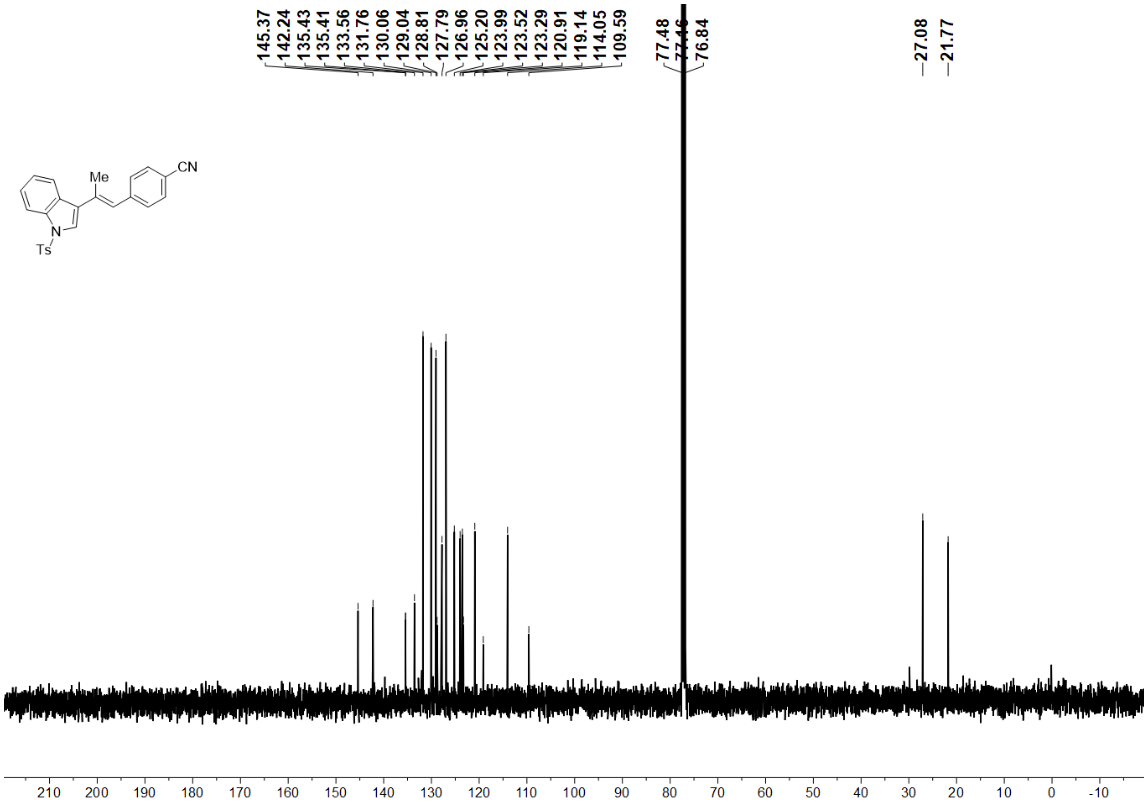

Supplementary Figure 61. $^{13}$C NMR (101 MHz, $CDCl_3$) spectrum of compound **1w**

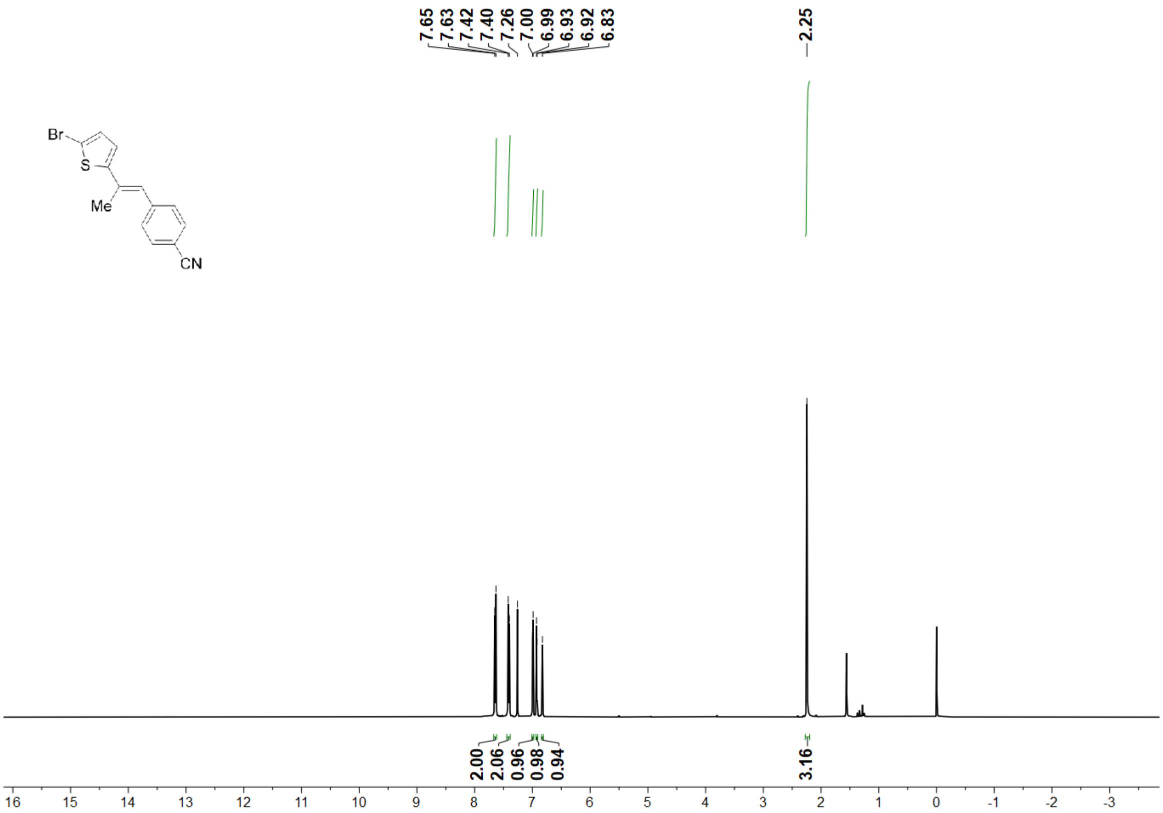


**Supplementary Figure 62.** $^{1}H$ NMR (400 MHz, $CDCl_3$) spectrum of compound **1x**

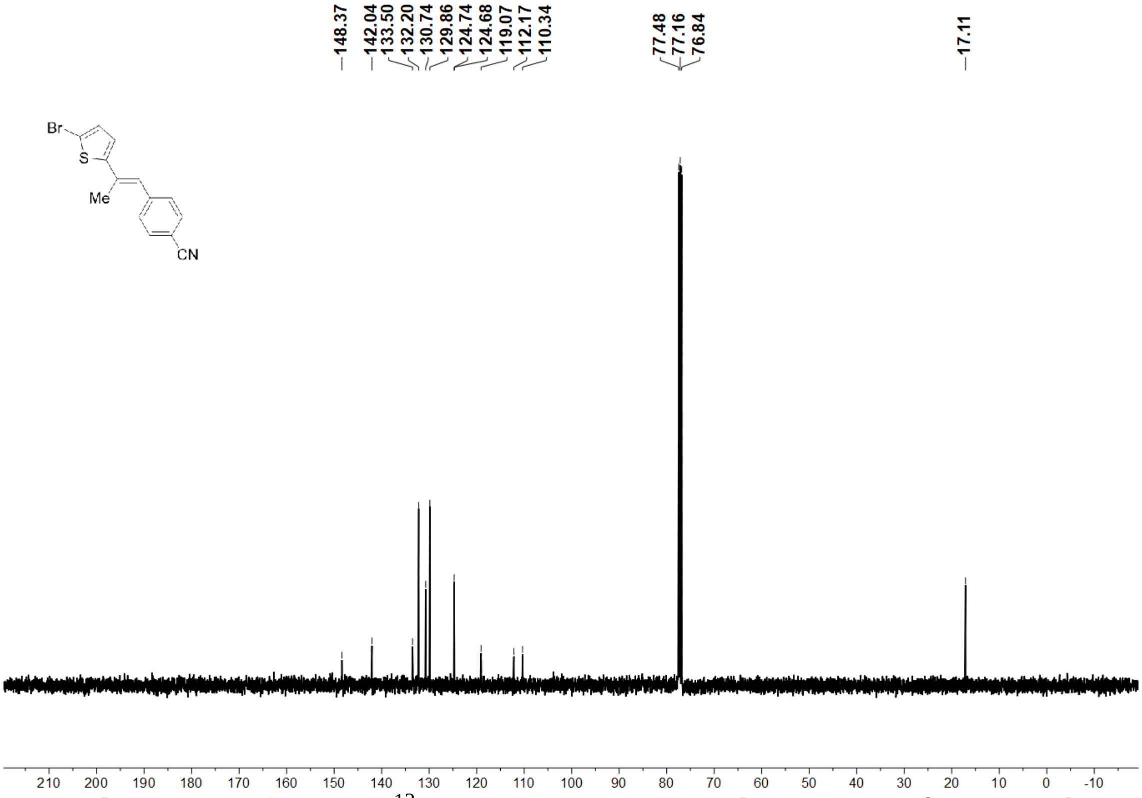


**Supplementary Figure 63.** $^{13}C$ NMR (101 MHz, $CDCl_3$) spectrum of compound **1x**

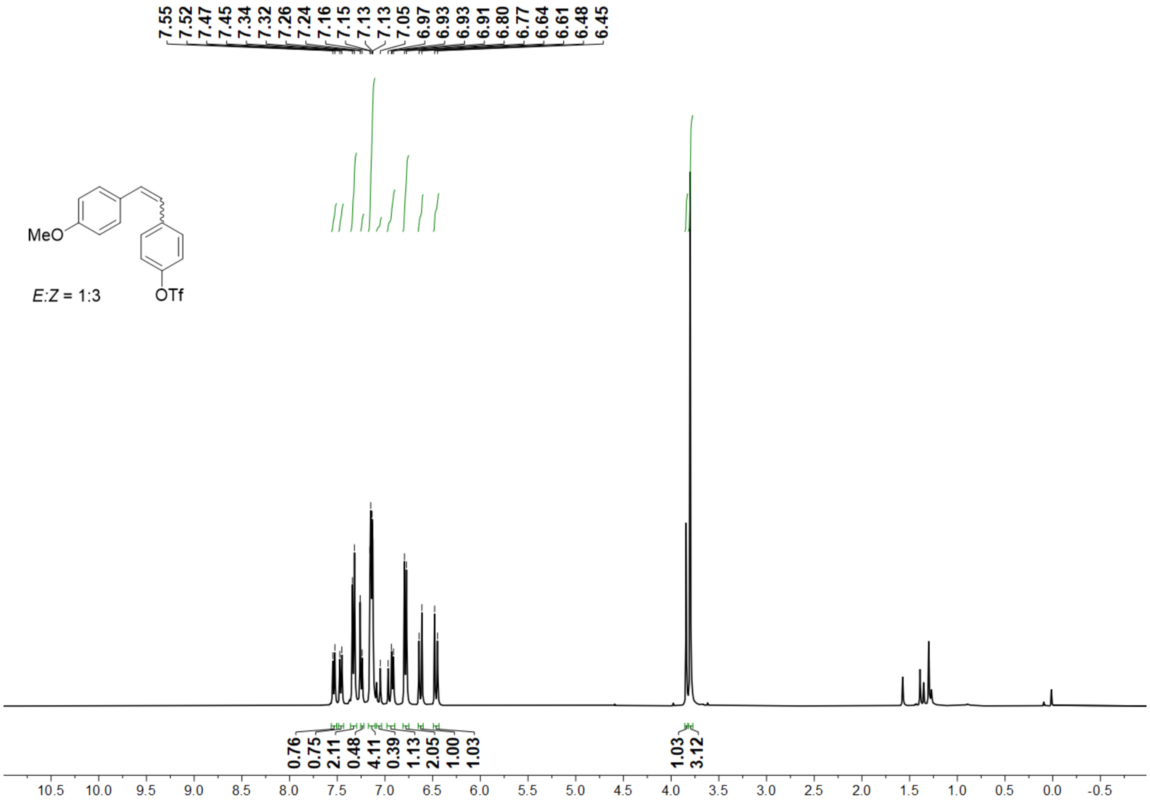

**Supplementary Figure 64.** $^{1}$H NMR (400 MHz, $CDCl_3$) spectrum of compound **1af**

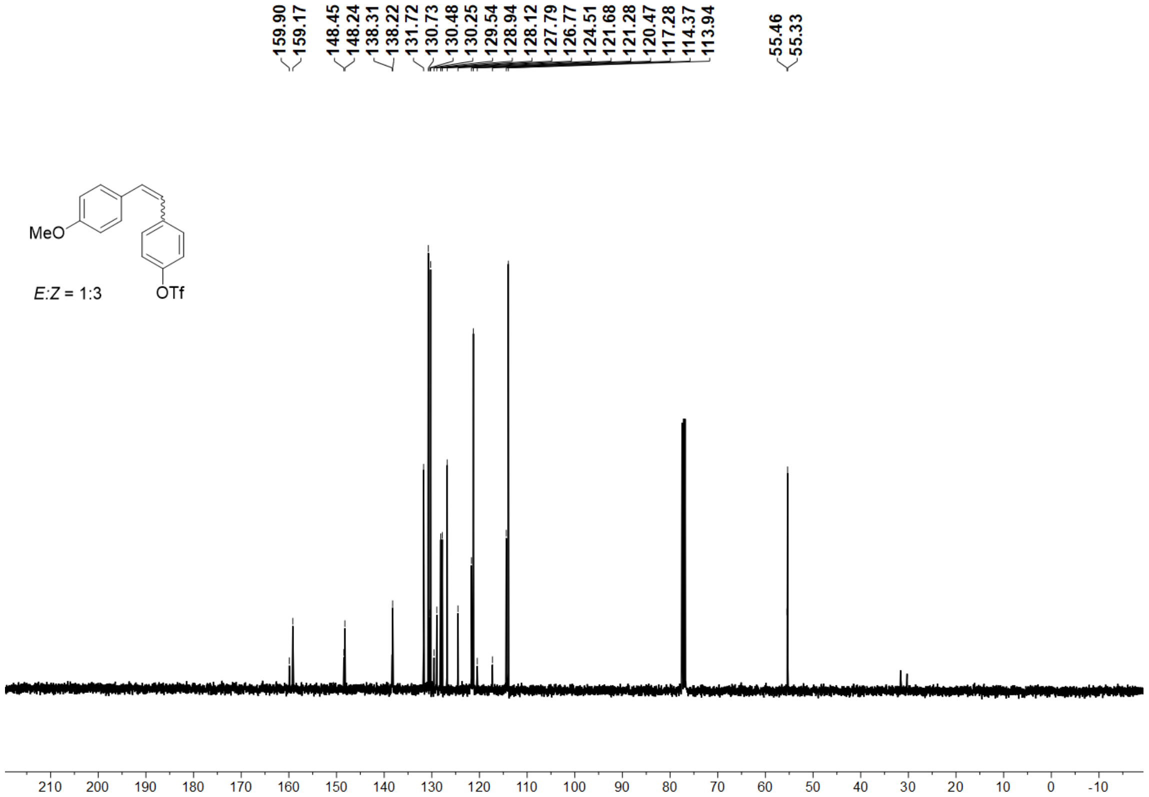

**Supplementary Figure 65.** $^{13}$C NMR (101 MHz, $CDCl_3$) spectrum of compound **1af**

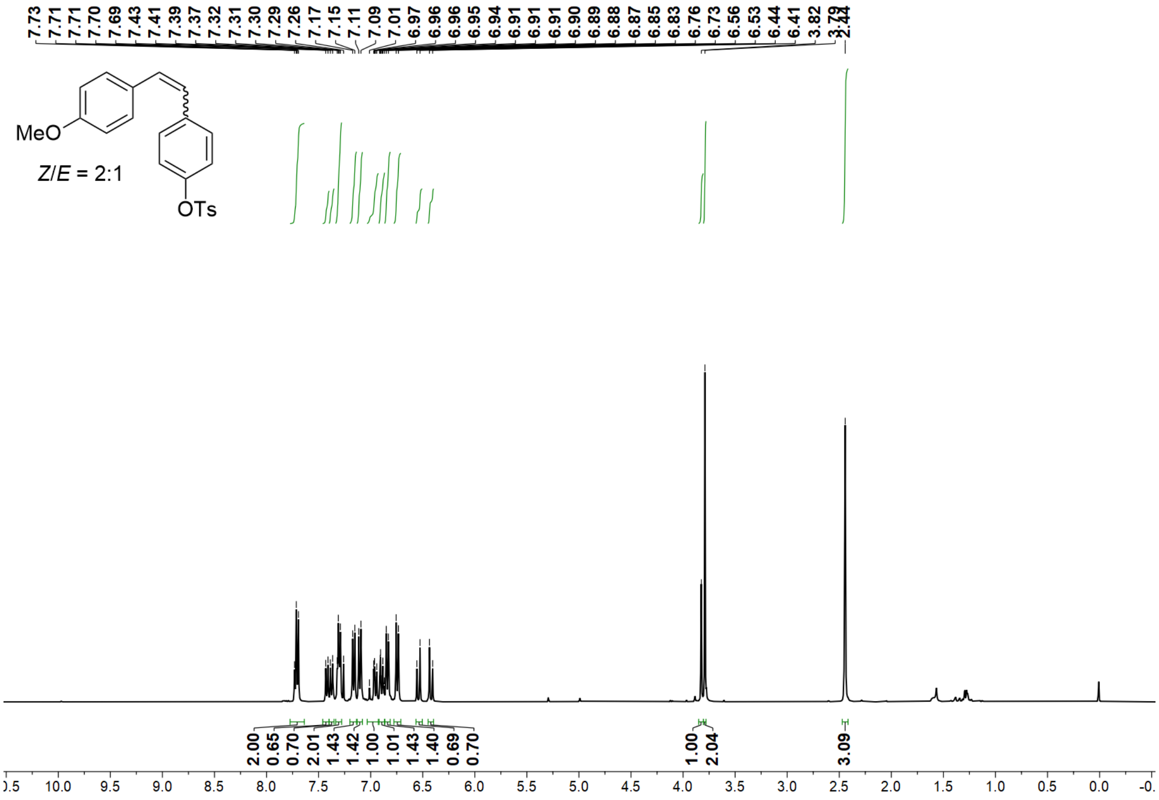

**Supplementary Figure 66.** $^{1}$H NMR (400 MHz, $CDCl_3$) spectrum of compound **1ag**

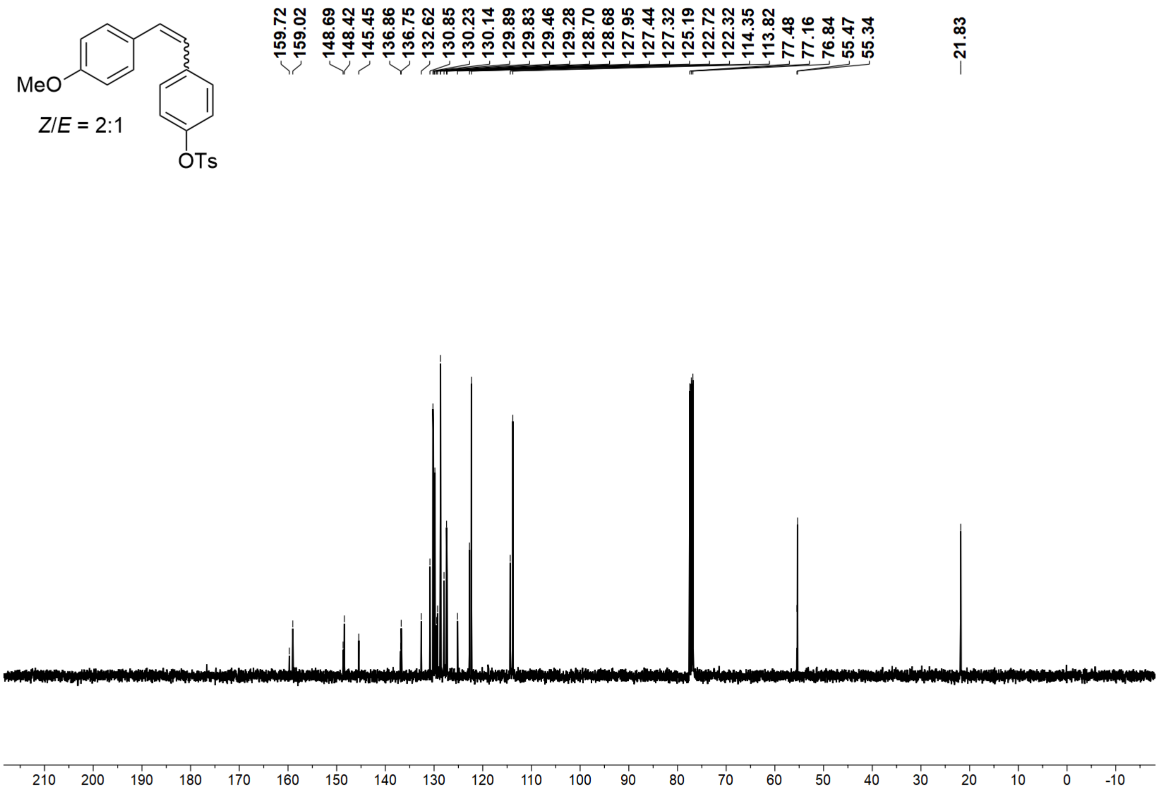

**Supplementary Figure 67.** $^{13}$C NMR (101 MHz, $CDCl_3$) spectrum of compound **1ag**

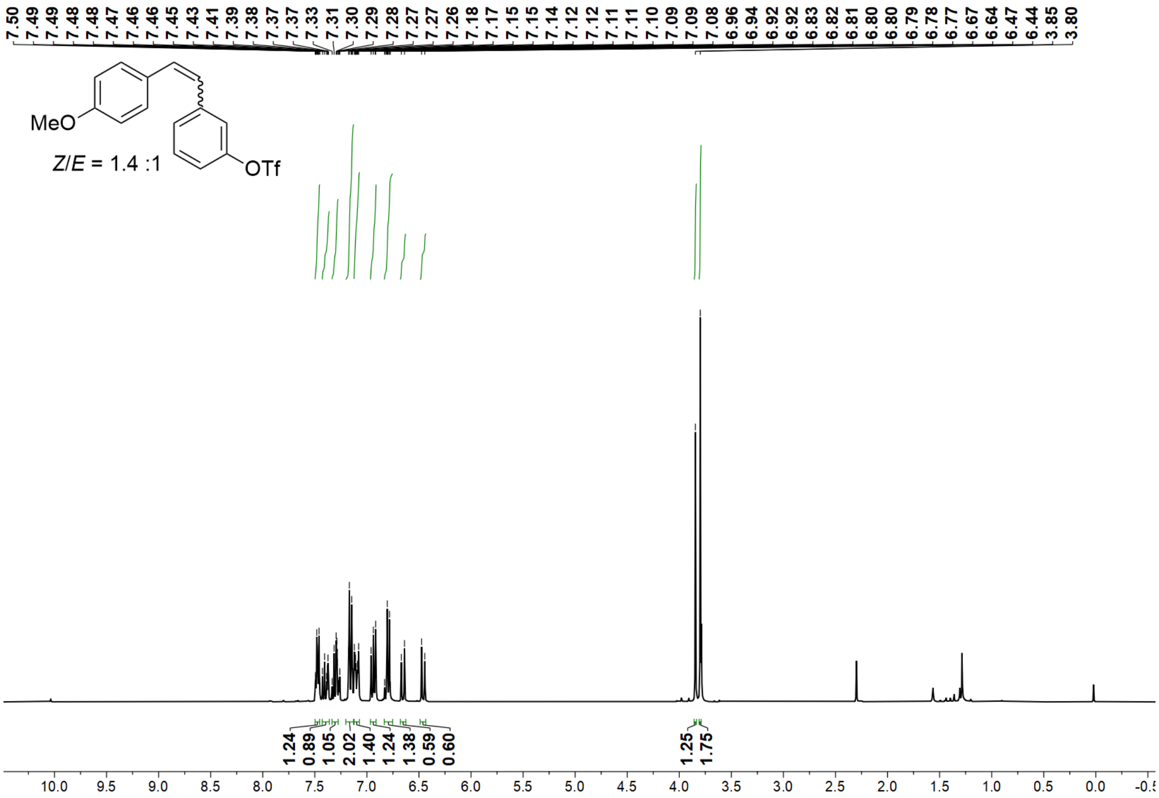


**Supplementary Figure 68.** $^{1}$H NMR (400 MHz, $CDCl_3$) spectrum of compound **1am**

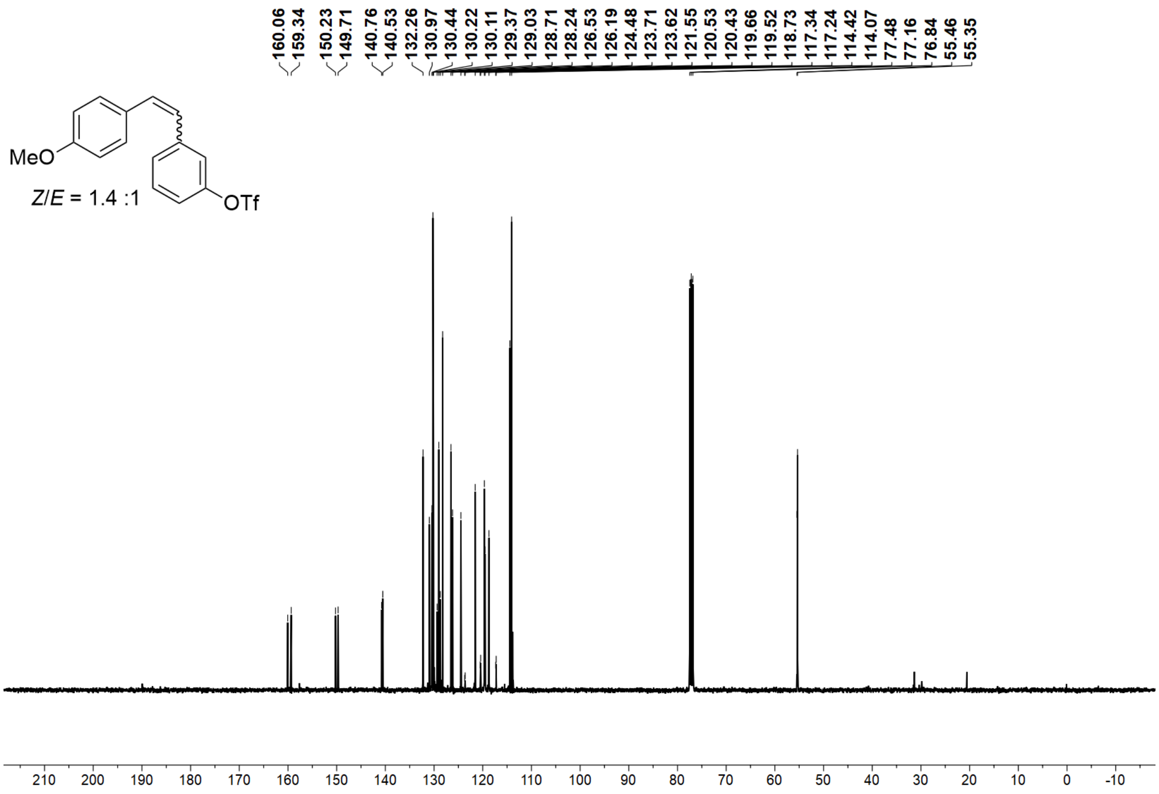


**Supplementary Figure 69.** $^{13}$C NMR (101 MHz, $CDCl_3$) spectrum of compound **1am**

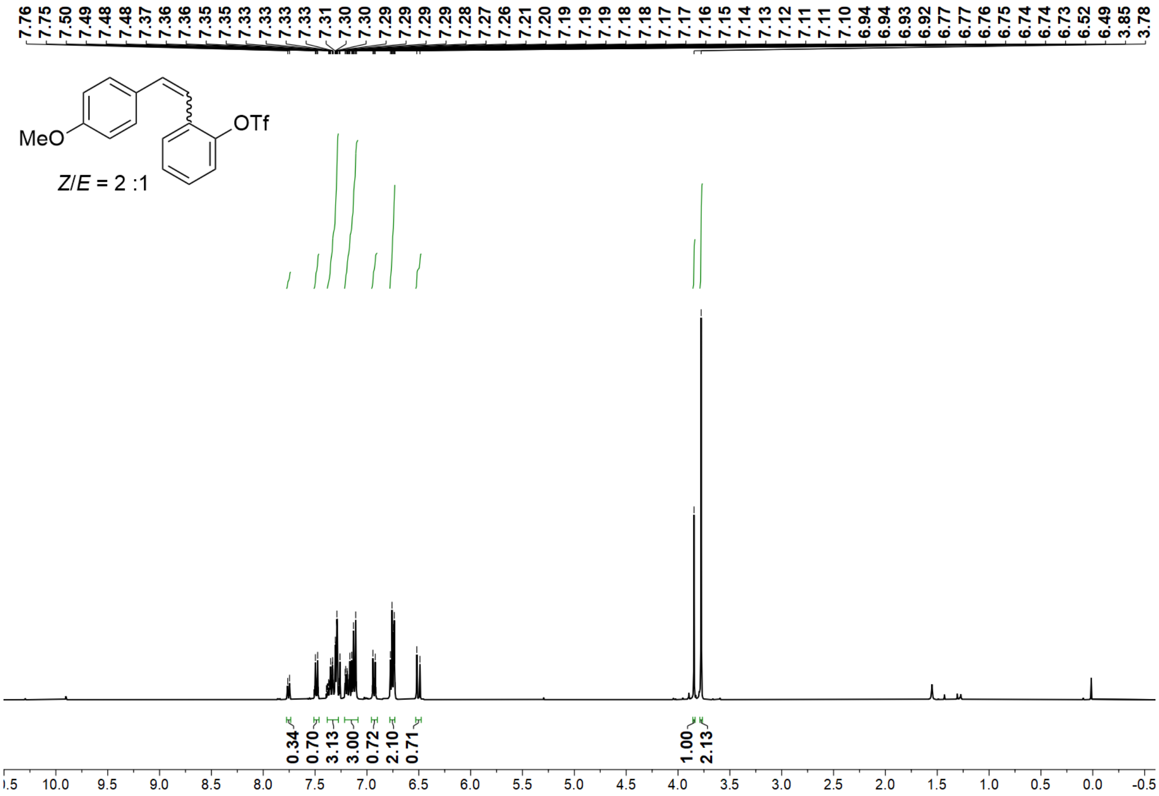


**Supplementary Figure 70.** $^{1}$H NMR (400 MHz, $CDCl_3$) spectrum of compound **1ao**

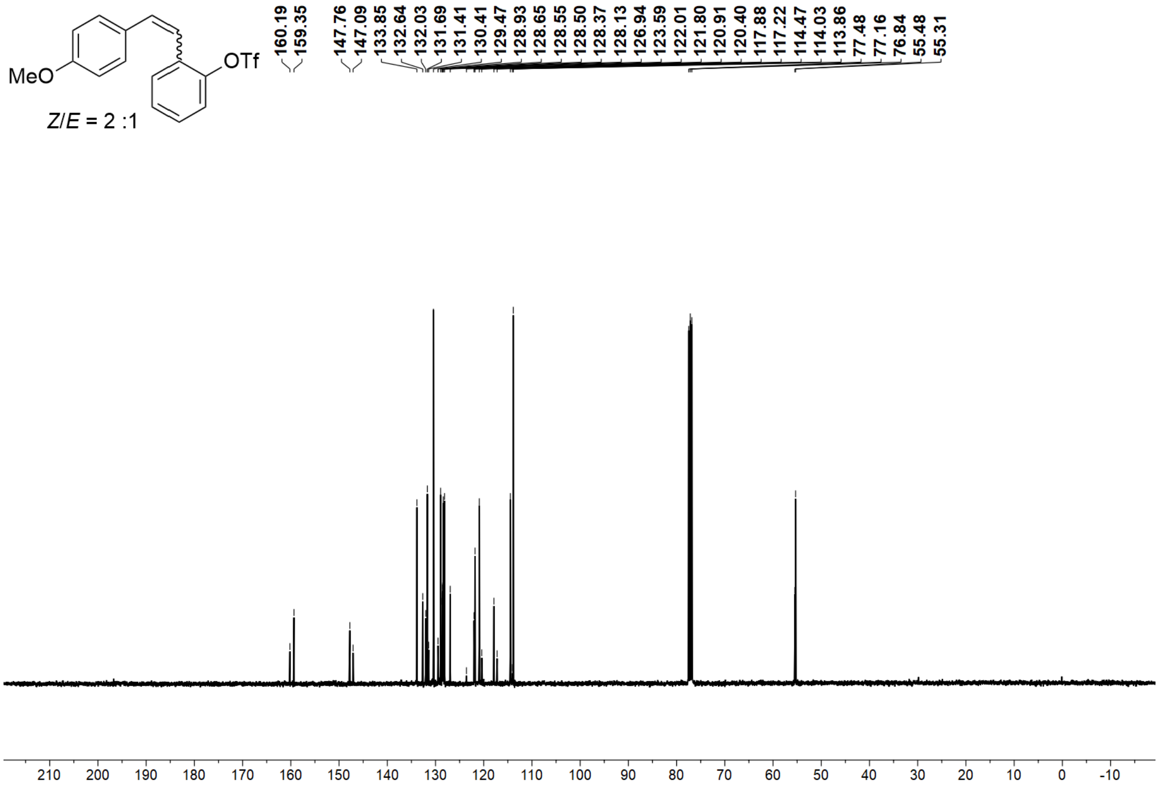


**Supplementary Figure 71.** $^{13}$C NMR (101 MHz, $CDCl_3$) spectrum of compound **1ao**

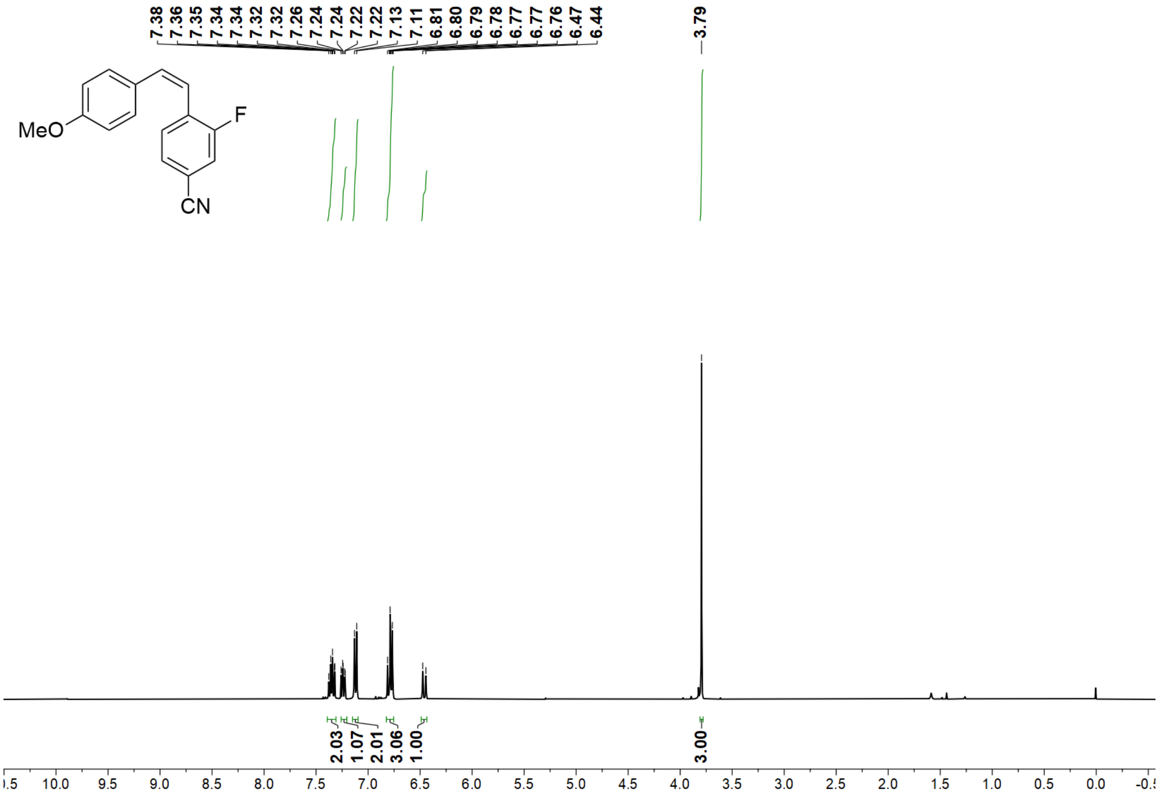

**Supplementary Figure 72.** $^{1}$H NMR (400 MHz, $CDCl_3$) spectrum of compound **1ap**

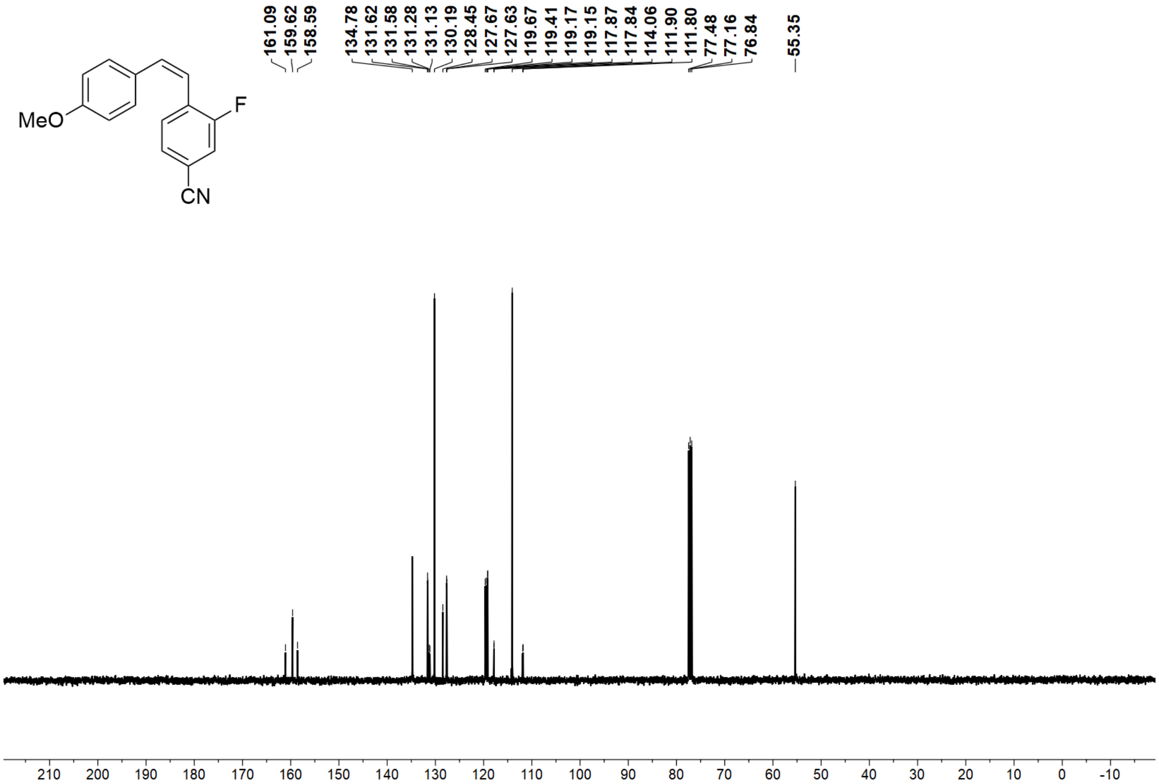

**Supplementary Figure 73.** $^{13}$C NMR (101 MHz, $CDCl_3$) spectrum of compound **1ap**

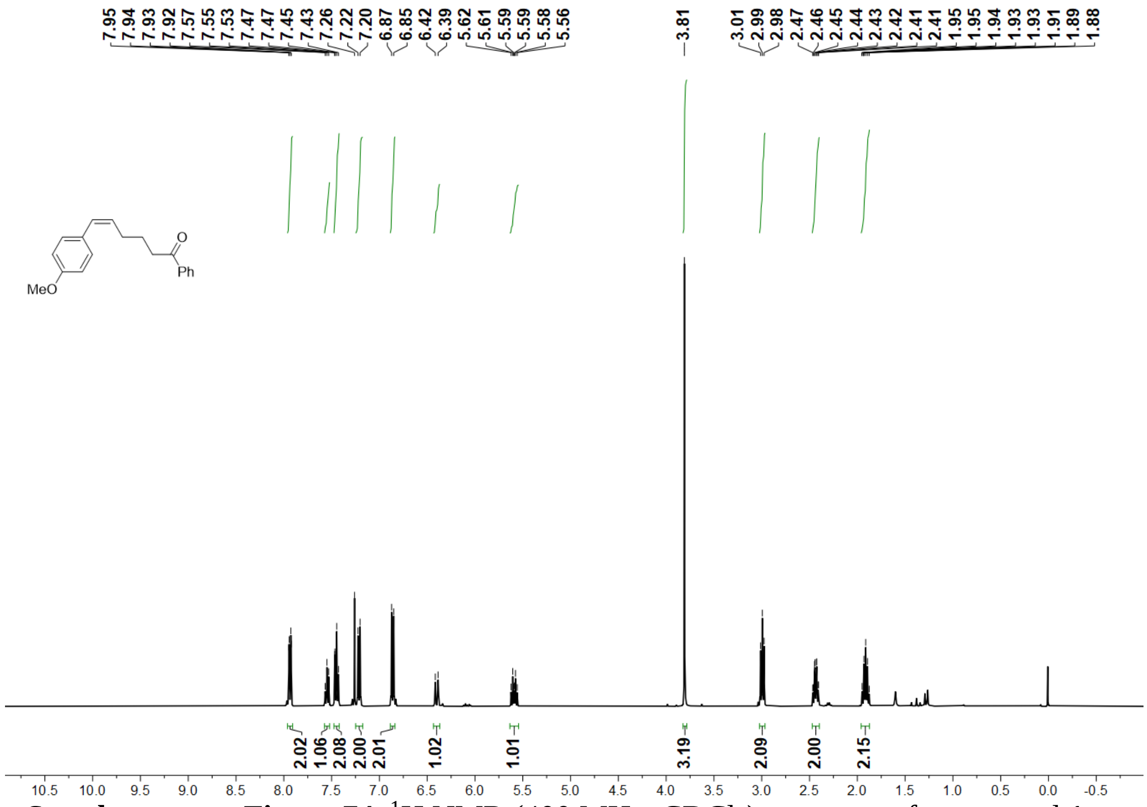

**Supplementary Figure 74.** $^{1}H$ NMR (400 MHz, $CDCl_3$) spectrum of compound **1aw**

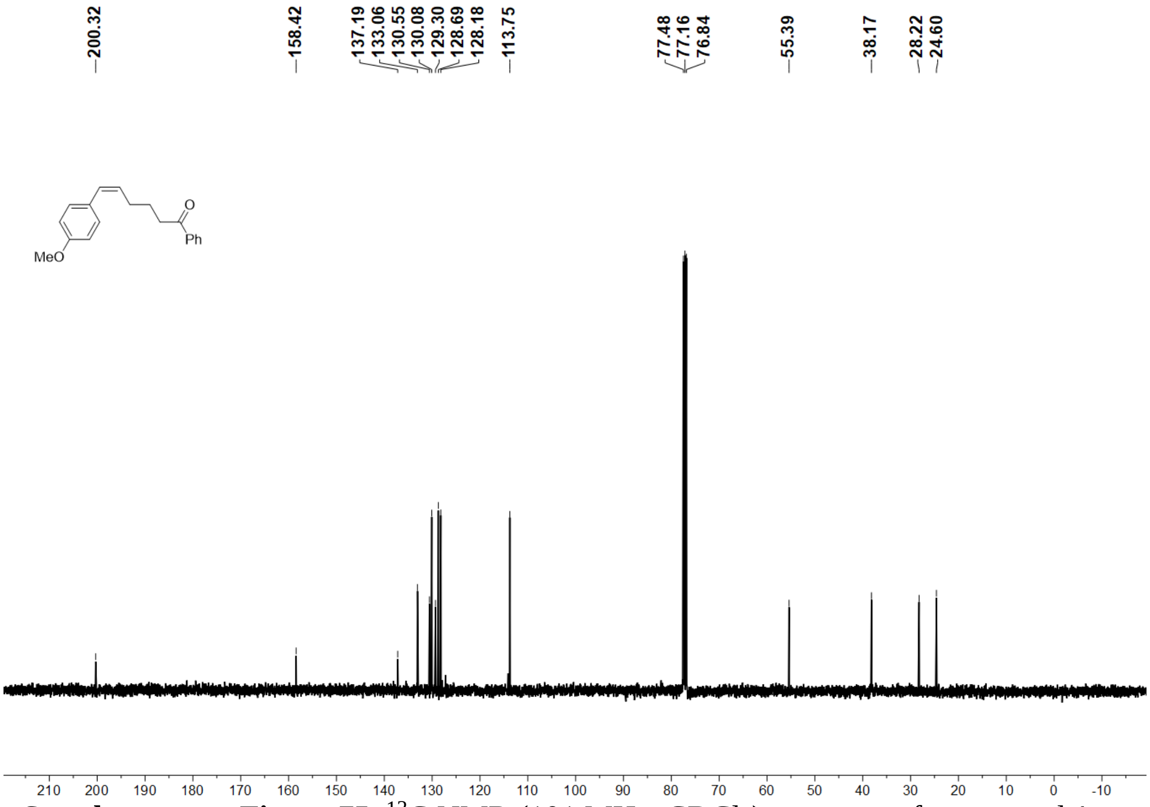

**Supplementary Figure 75.** $^{13}C$ NMR (101 MHz, $CDCl_3$) spectrum of compound **1aw**

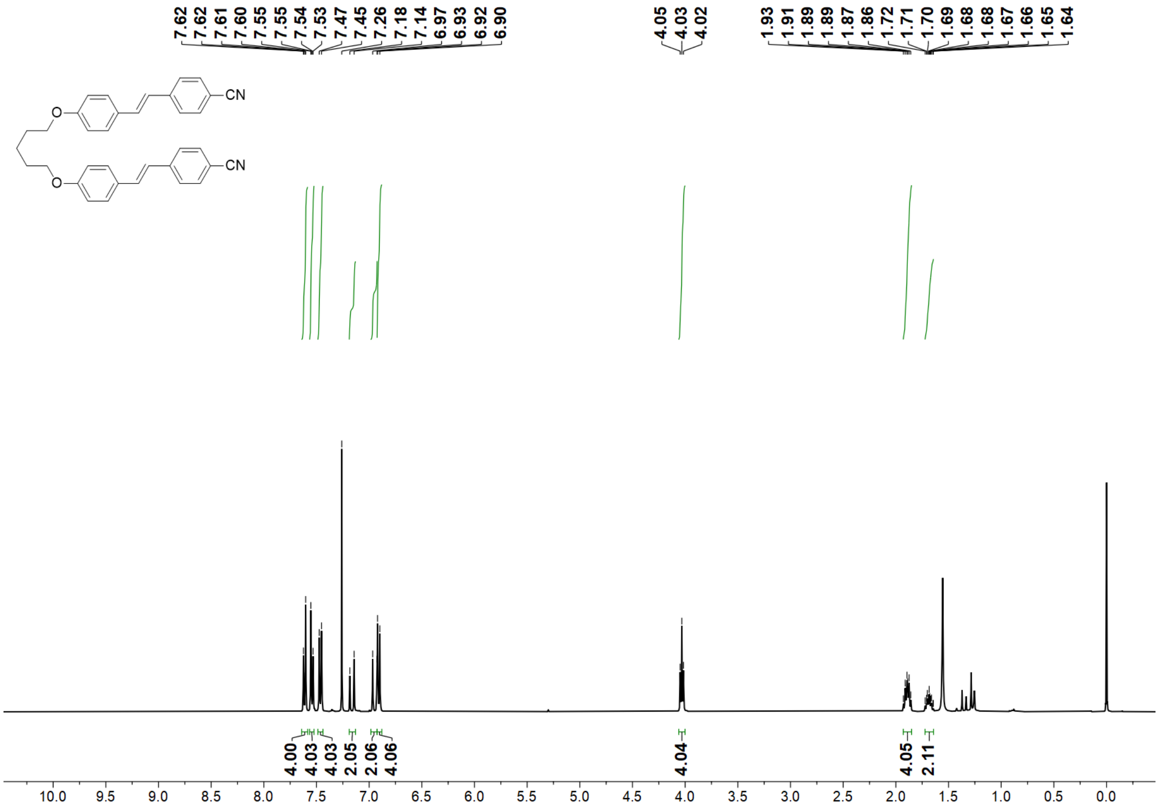


**Supplementary Figure 76.** $^{1}H$ NMR (400 MHz, $CDCl_3$) spectrum of compound **1ax**

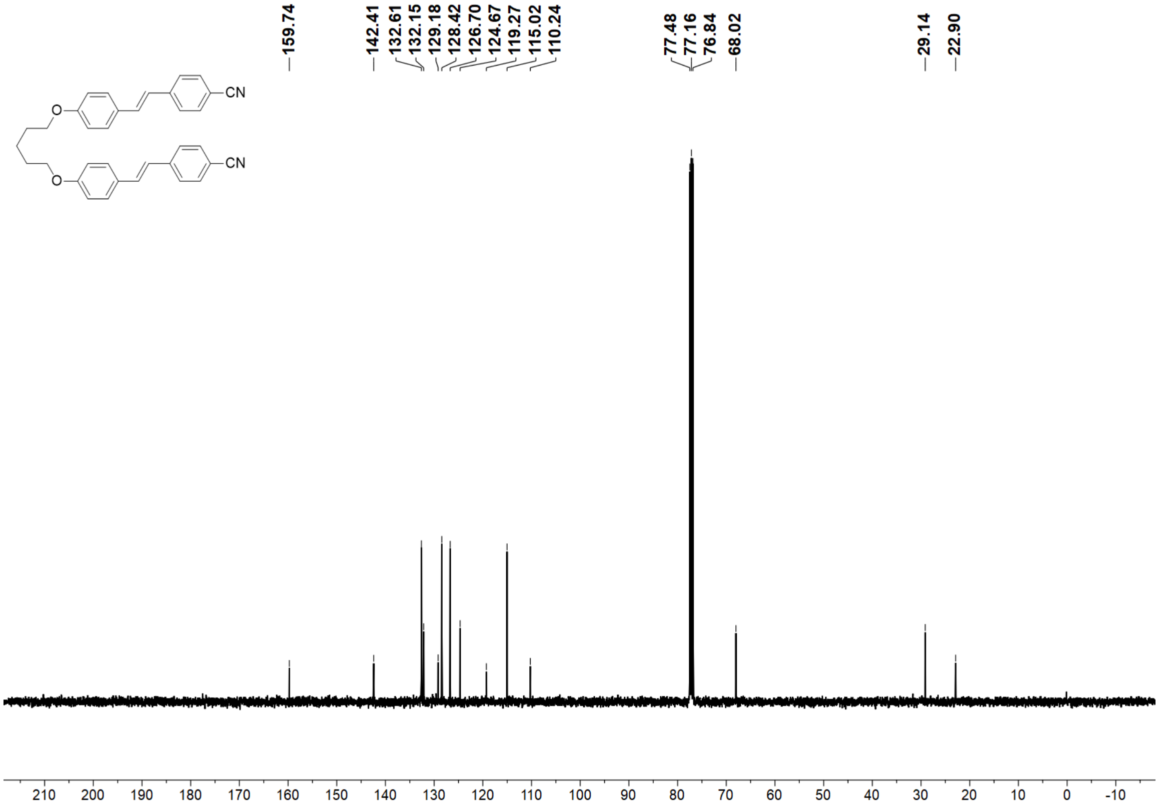


**Supplementary Figure 77.** $^{13}C$ NMR (101 MHz, $CDCl_3$) spectrum of compound **1ax**

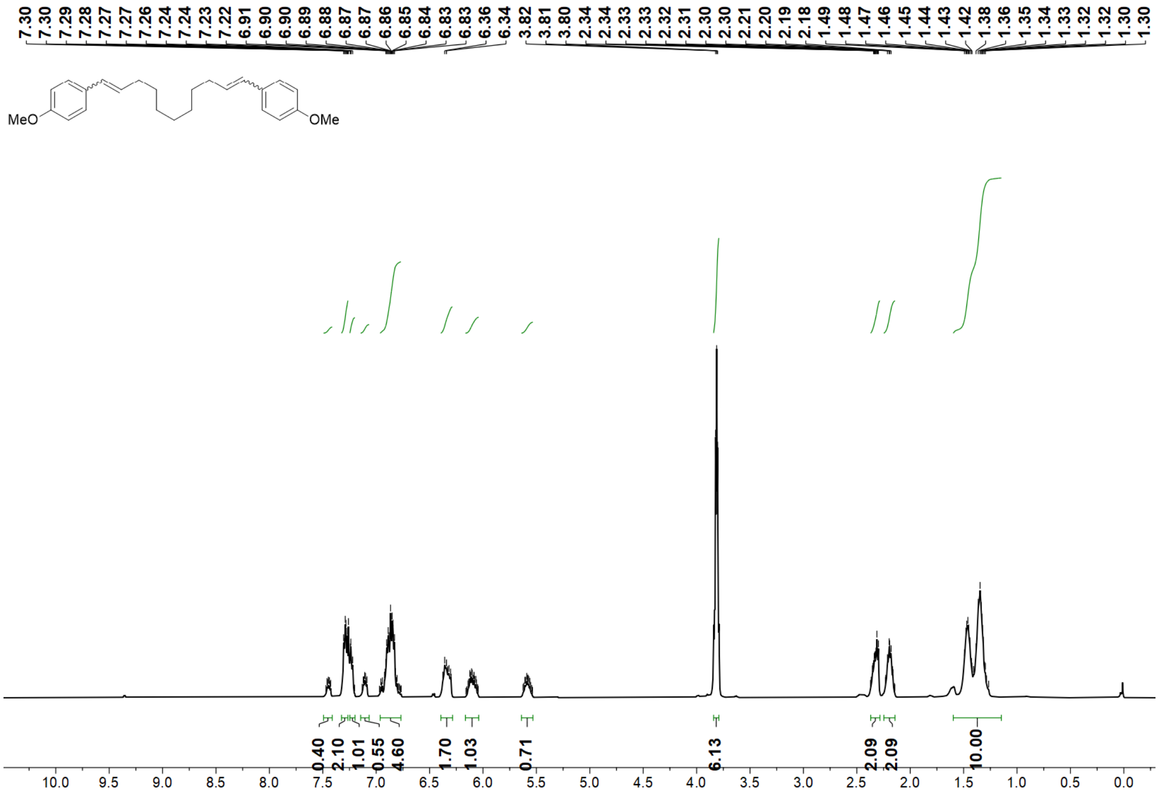


**Supplementary Figure 78.** $^{1}H$ NMR (400 MHz, $CDCl_3$) spectrum of compound **1ay**

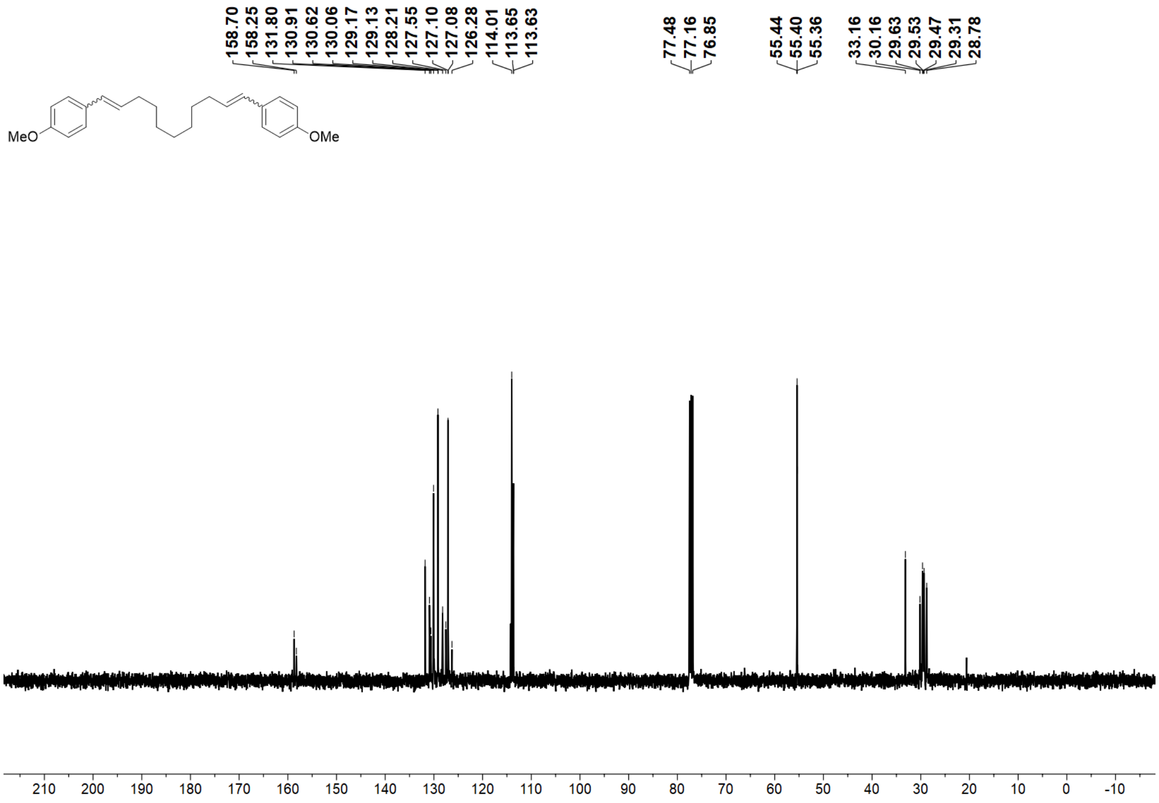


**Supplementary Figure 79.** $^{13}C$ NMR (101 MHz, $CDCl_3$) spectrum of compound **1ay**

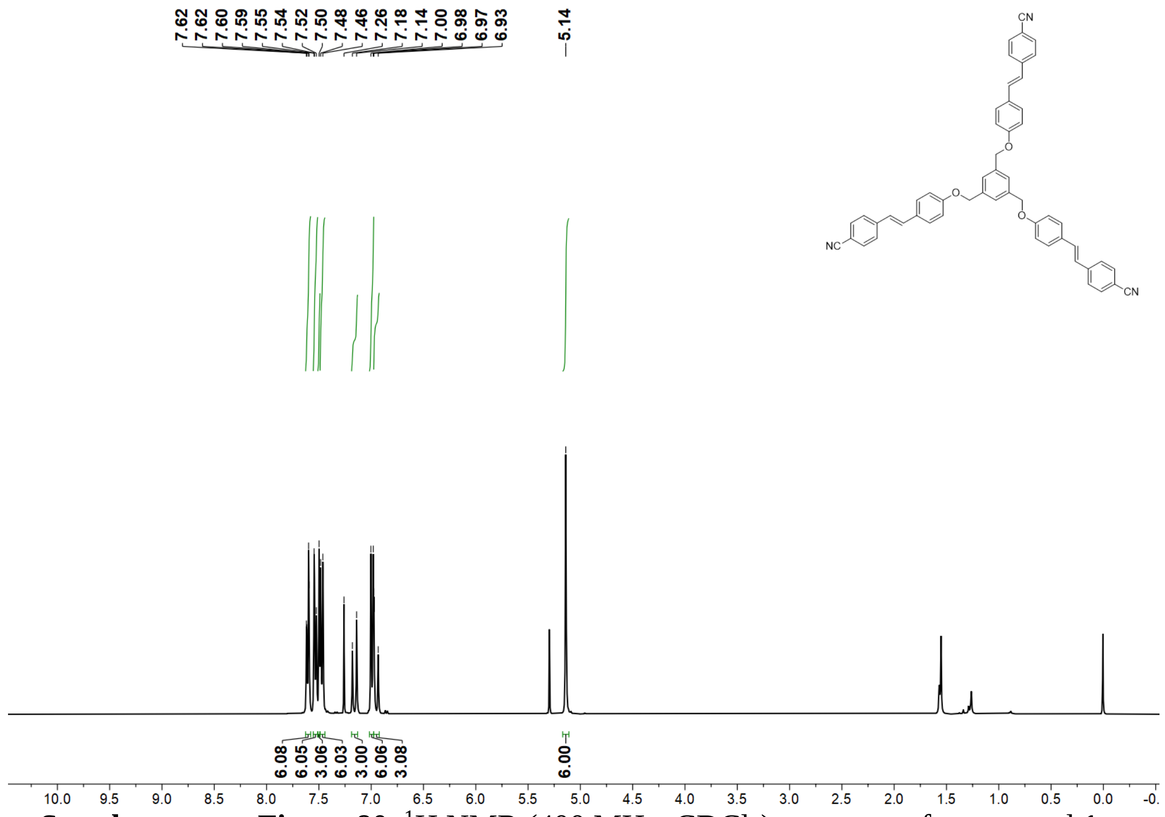


**Supplementary Figure 80.** $^{1}$H NMR (400 MHz, $CDCl_3$) spectrum of compound **1az**

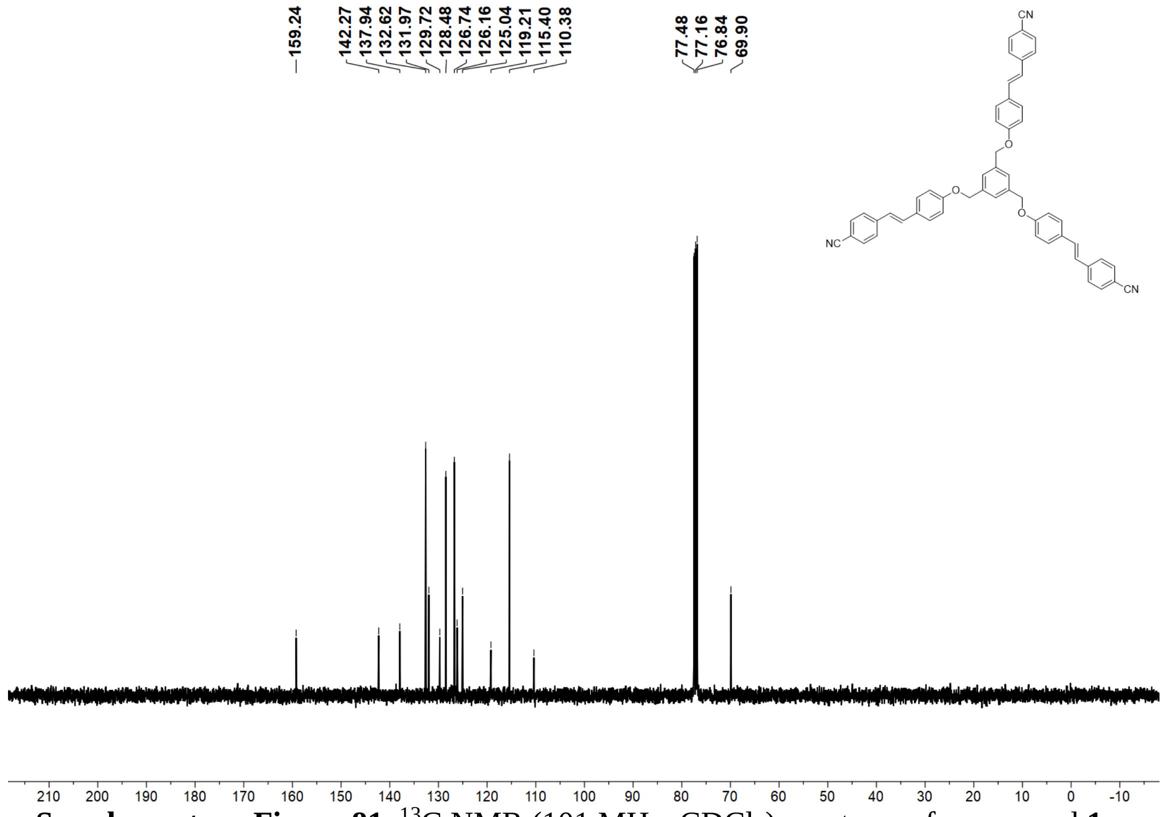


**Supplementary Figure 81.** $^{13}$C NMR (101 MHz, $CDCl_3$) spectrum of compound **1az**

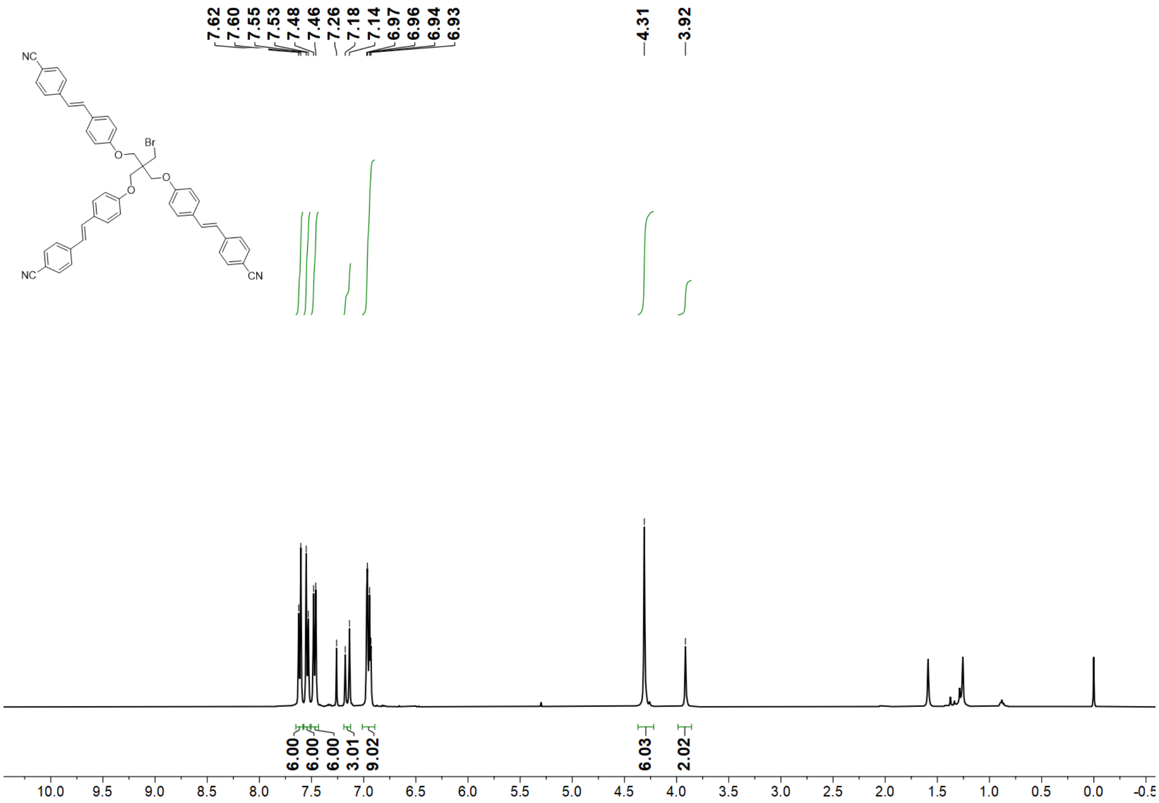


**Supplementary Figure 82.** $^{1}$H NMR (400 MHz, $CDCl_3$) spectrum of compound **1ba**

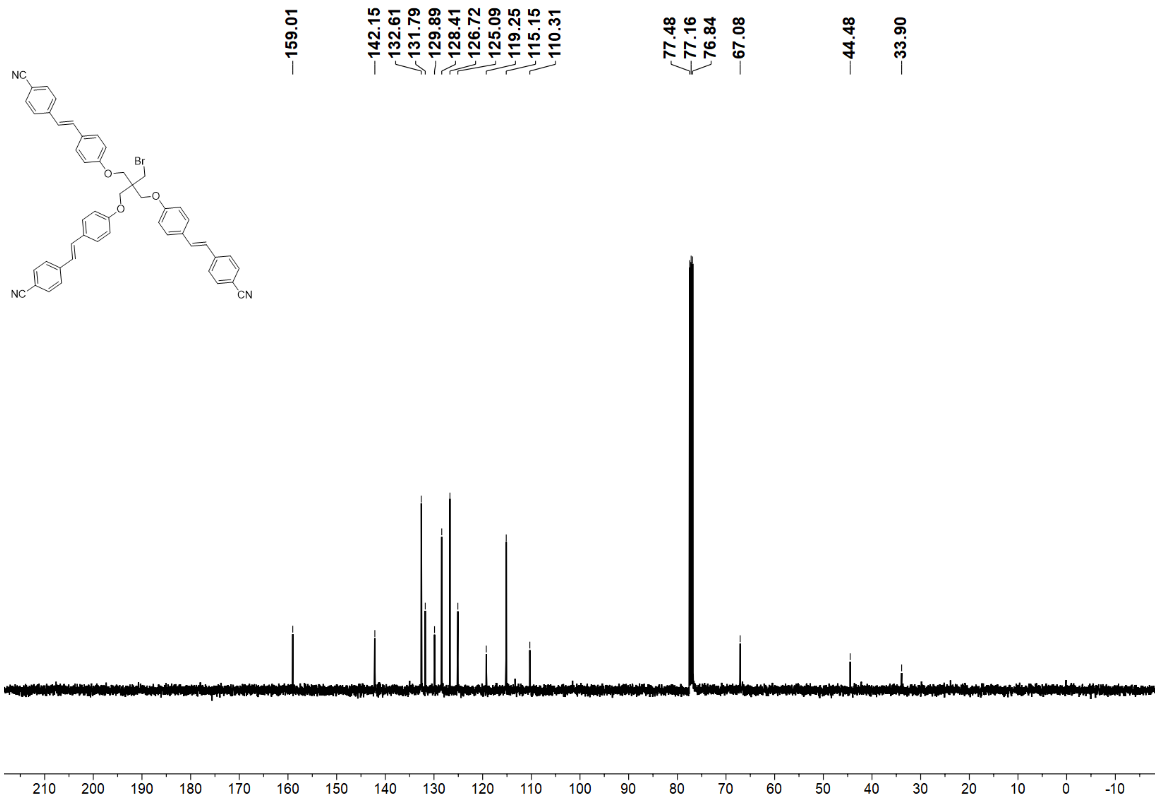


**Supplementary Figure 83.** $^{13}$C NMR (101 MHz, $CDCl_3$) spectrum of compound **1ba**

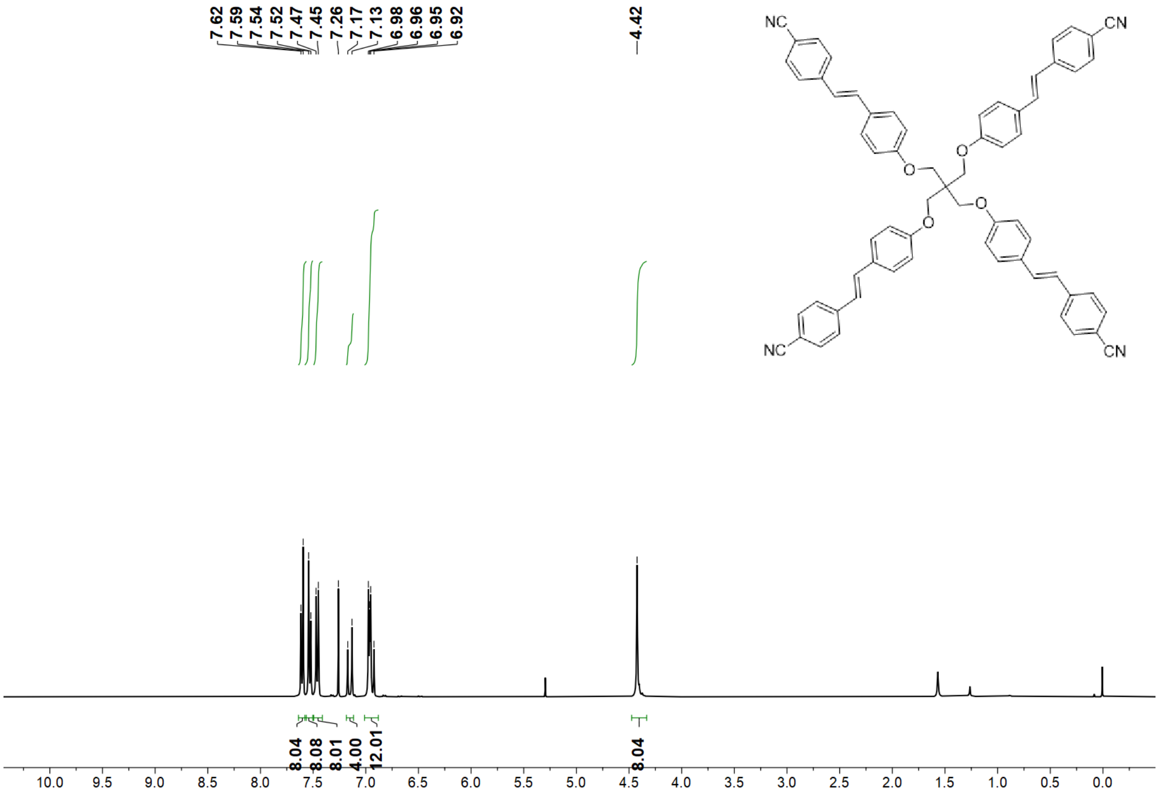


**Supplementary Figure 84.** $^{1}$H NMR (400 MHz, $CDCl_3$) spectrum of compound **1bb**

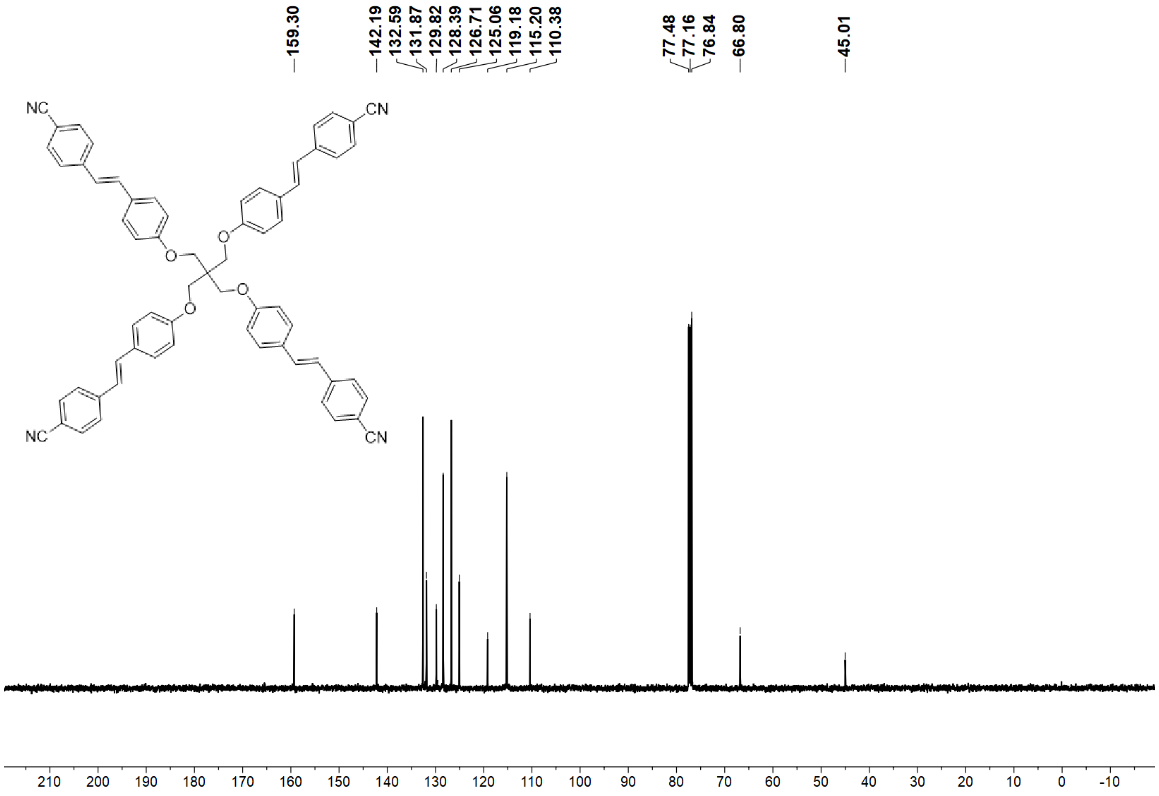


**Supplementary Figure 85.** $^{13}$C NMR (101 MHz, $CDCl_3$) spectrum of compound **1bb**

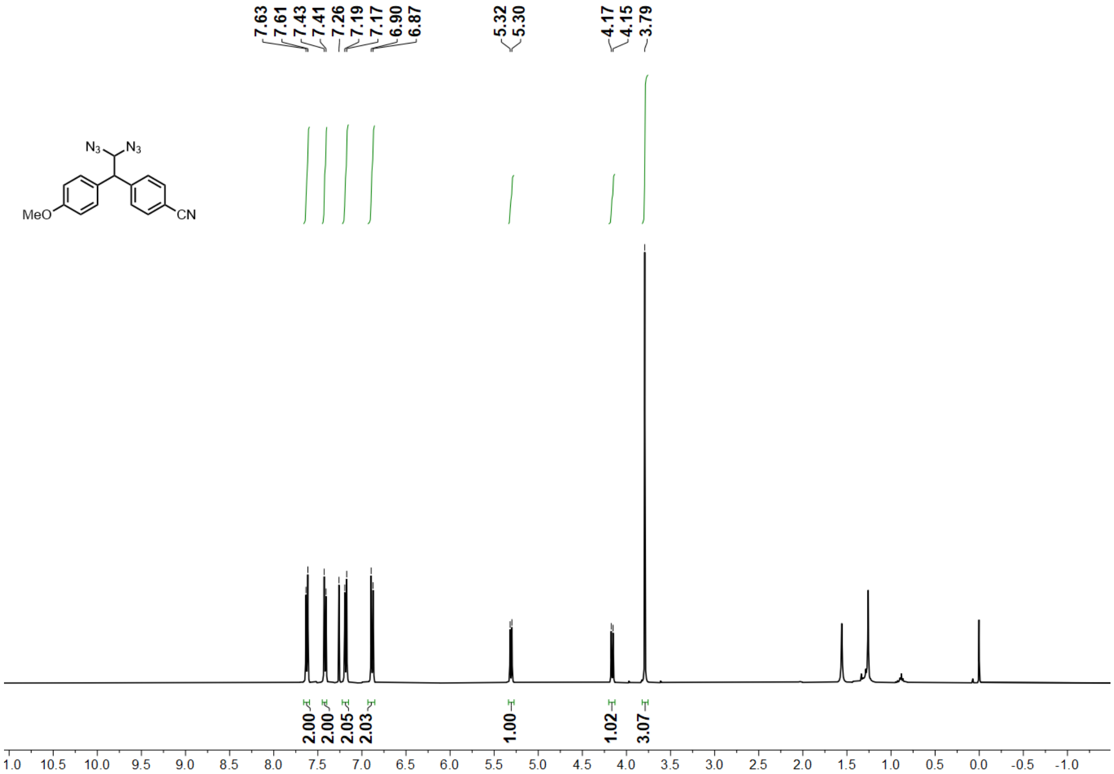


**Supplementary Figure 86.** $^{1}$H NMR (400 MHz, $CDCl_3$) spectrum of compound **2a**

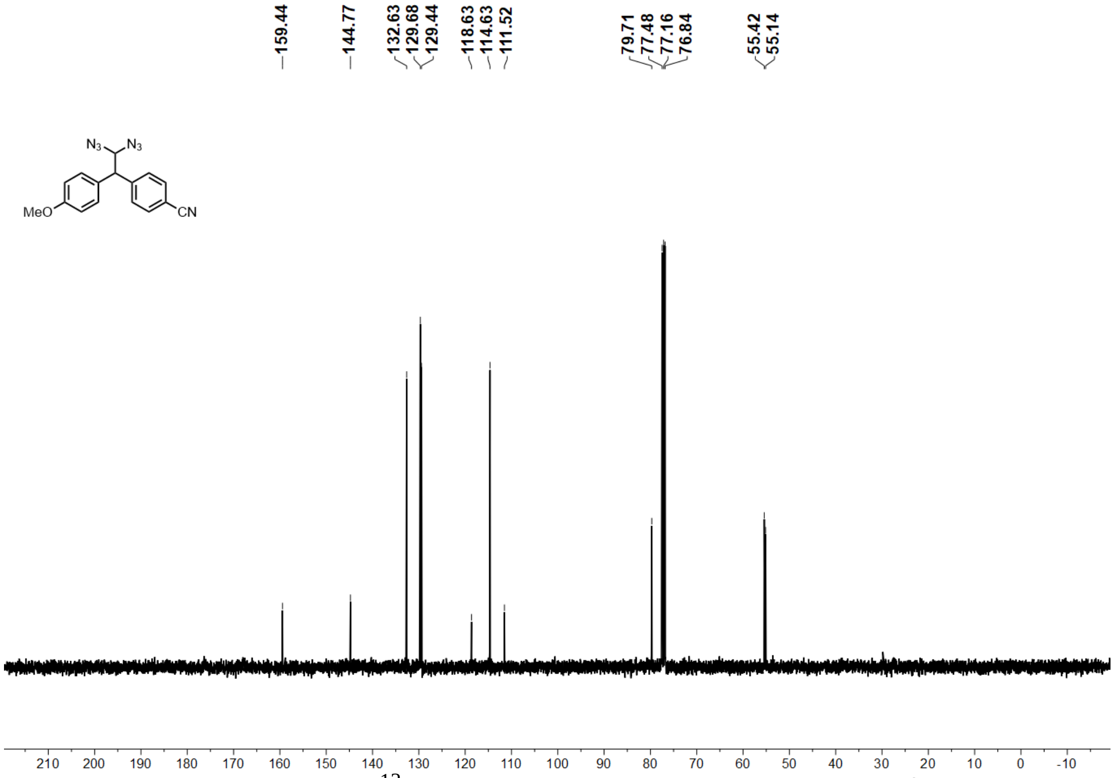


**Supplementary Figure 87.** $^{13}$C NMR (101 MHz, $CDCl_3$) spectrum of compound **2a**

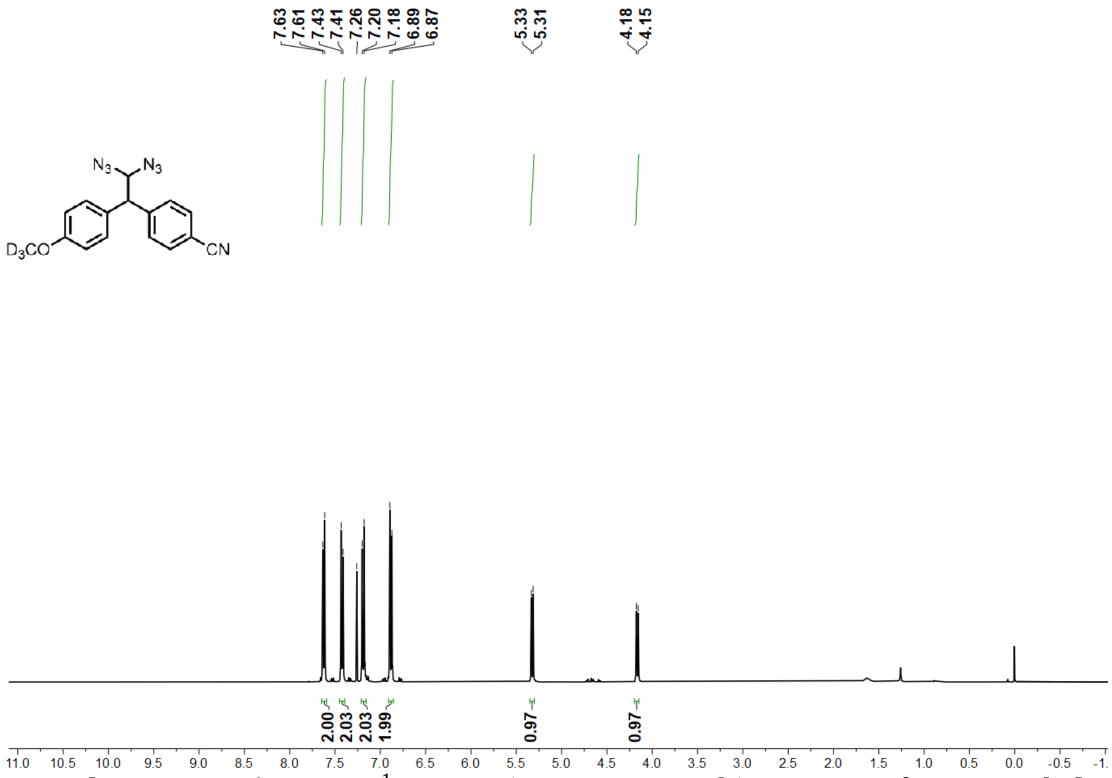


**Supplementary Figure 88.** $^{1}H$ NMR (400 MHz, $CDCl_3$) spectrum of compound **2b**

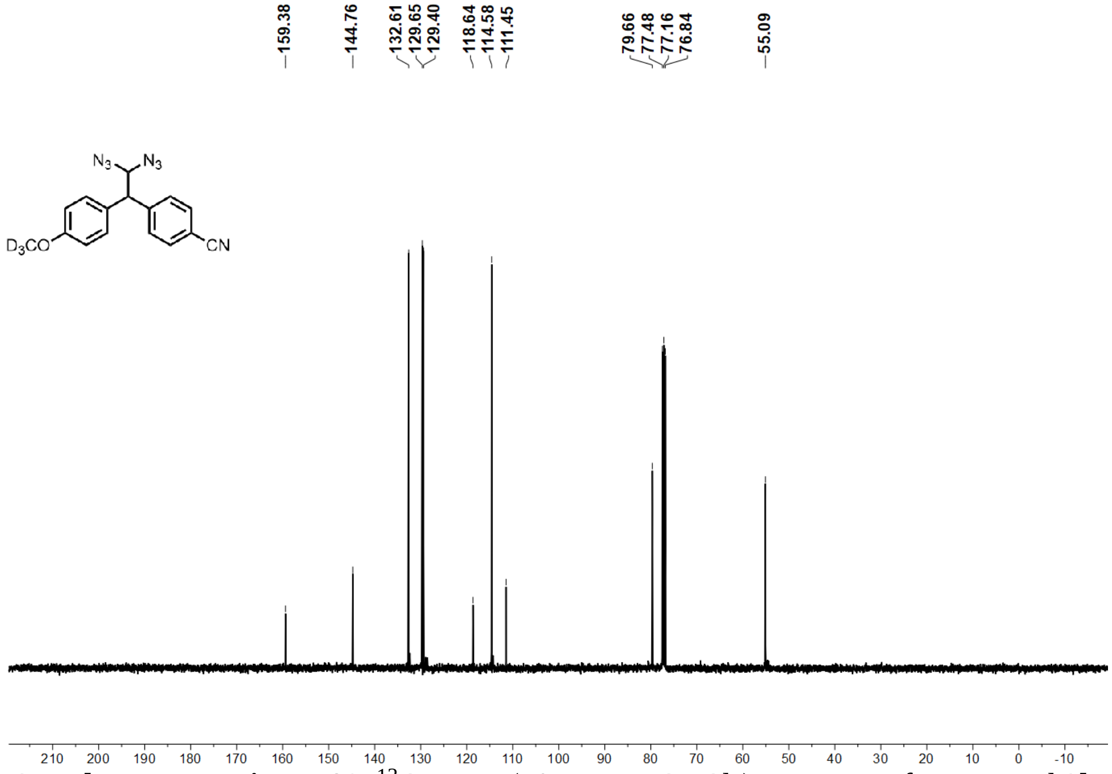


**Supplementary Figure 89.** $^{13}C$ NMR (101 MHz, $CDCl_3$) spectrum of compound **2b**

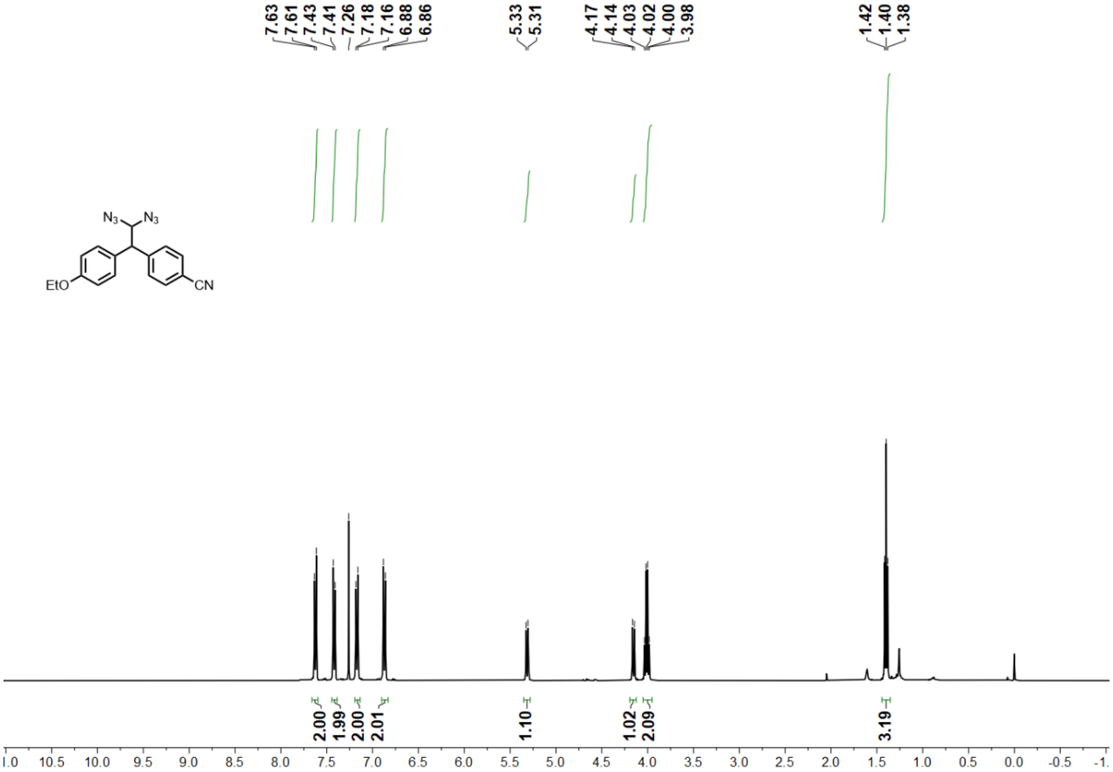


**Supplementary Figure 90.** $^{1}H$ NMR (400 MHz, $CDCl_3$) spectrum of compound **2c**

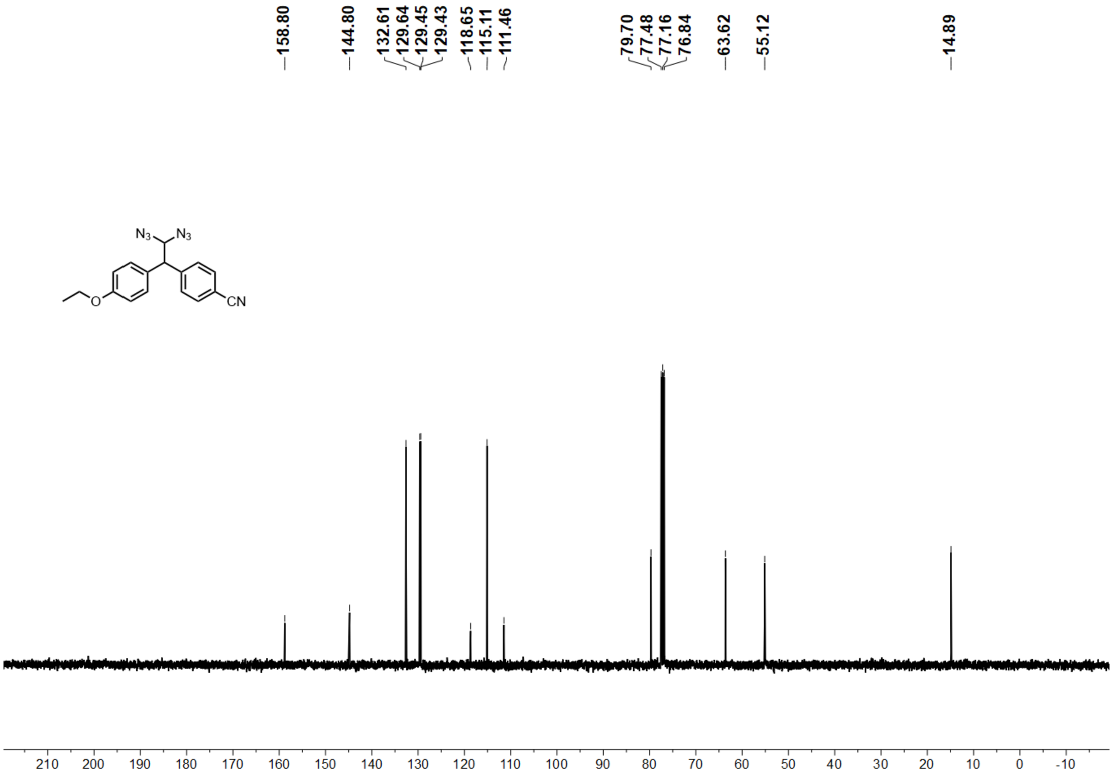


**Supplementary Figure 91.** $^{13}C$ NMR (101 MHz, $CDCl_3$) spectrum of compound **2c**

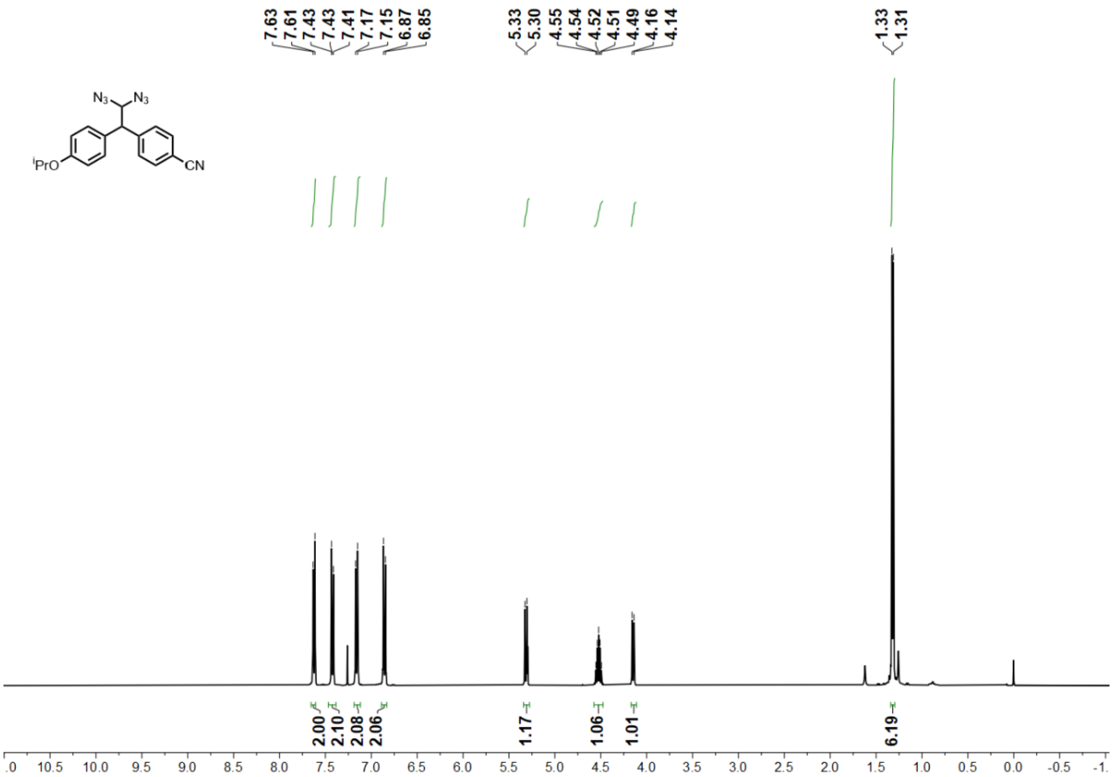


**Supplementary Figure 92.** $^{1}$H NMR (400 MHz, $CDCl_3$) spectrum of compound **2d**

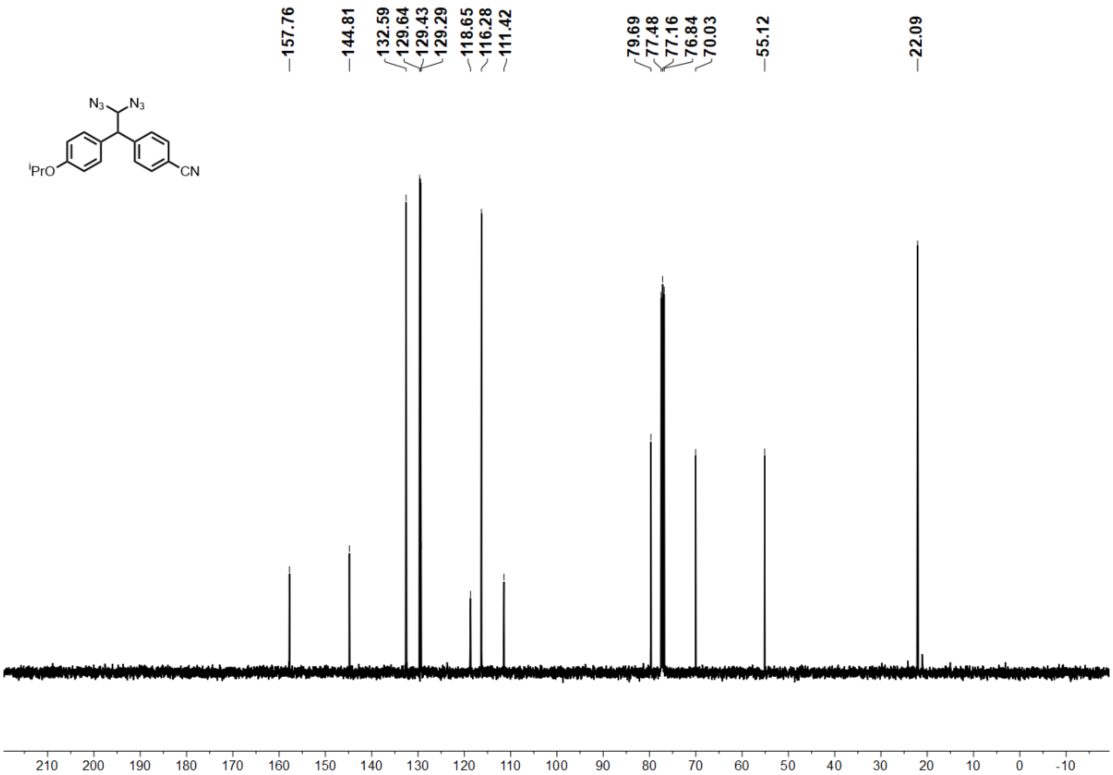


**Supplementary Figure 93.** $^{13}$C NMR (101 MHz, $CDCl_3$) spectrum of compound **2d**

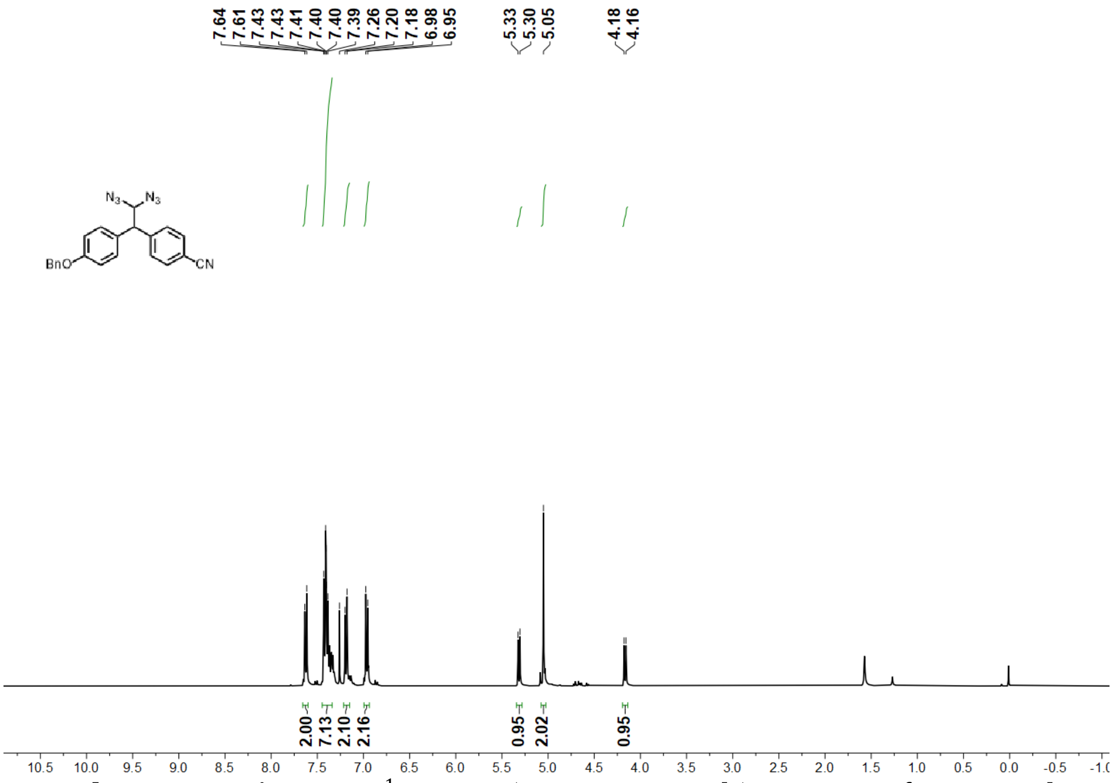


**Supplementary Figure 94.** $^{1}$H NMR (400 MHz, $CDCl_3$) spectrum of compound **2e**

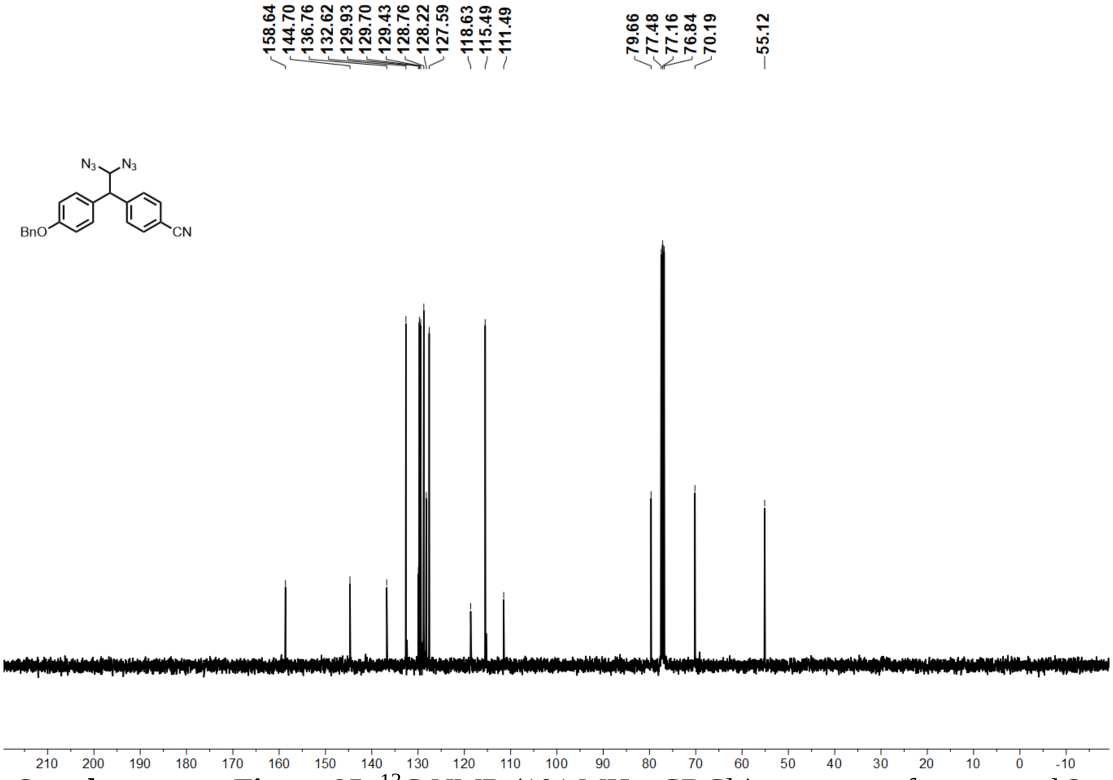


**Supplementary Figure 95.** $^{13}$C NMR (101 MHz, $CDCl_3$) spectrum of compound **2e**

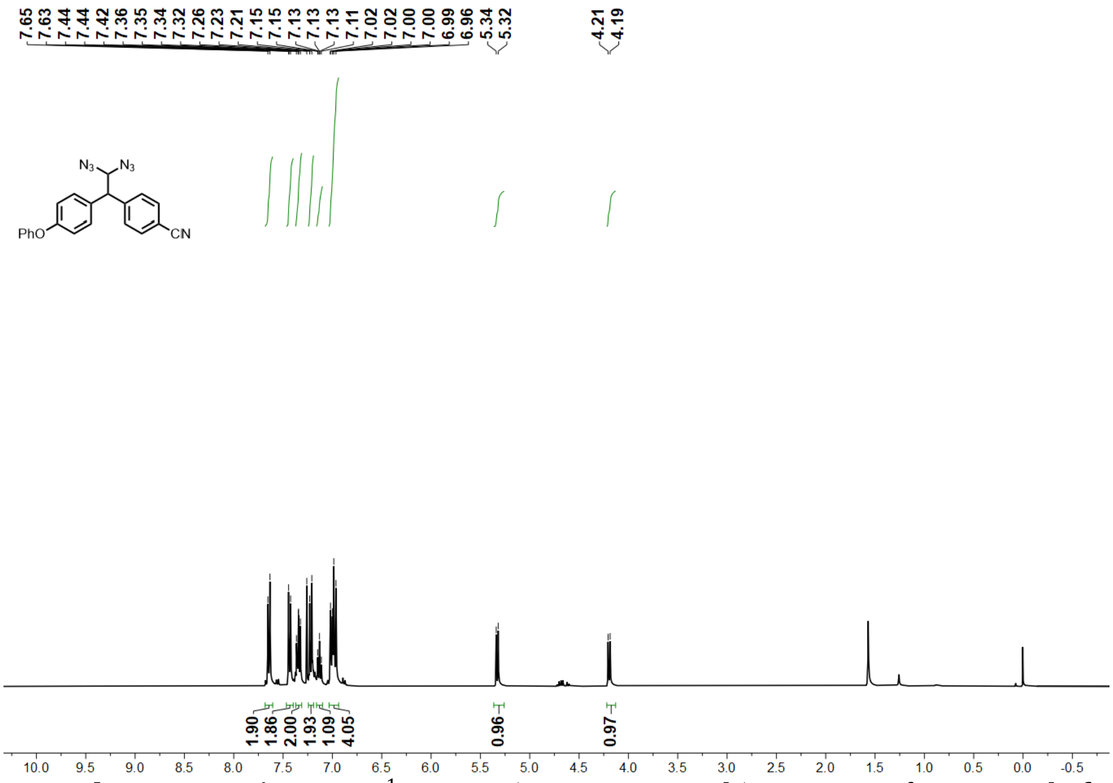

**Supplementary Figure 96.** $^{1}H$ NMR (400 MHz, $CDCl_3$) spectrum of compound **2f**

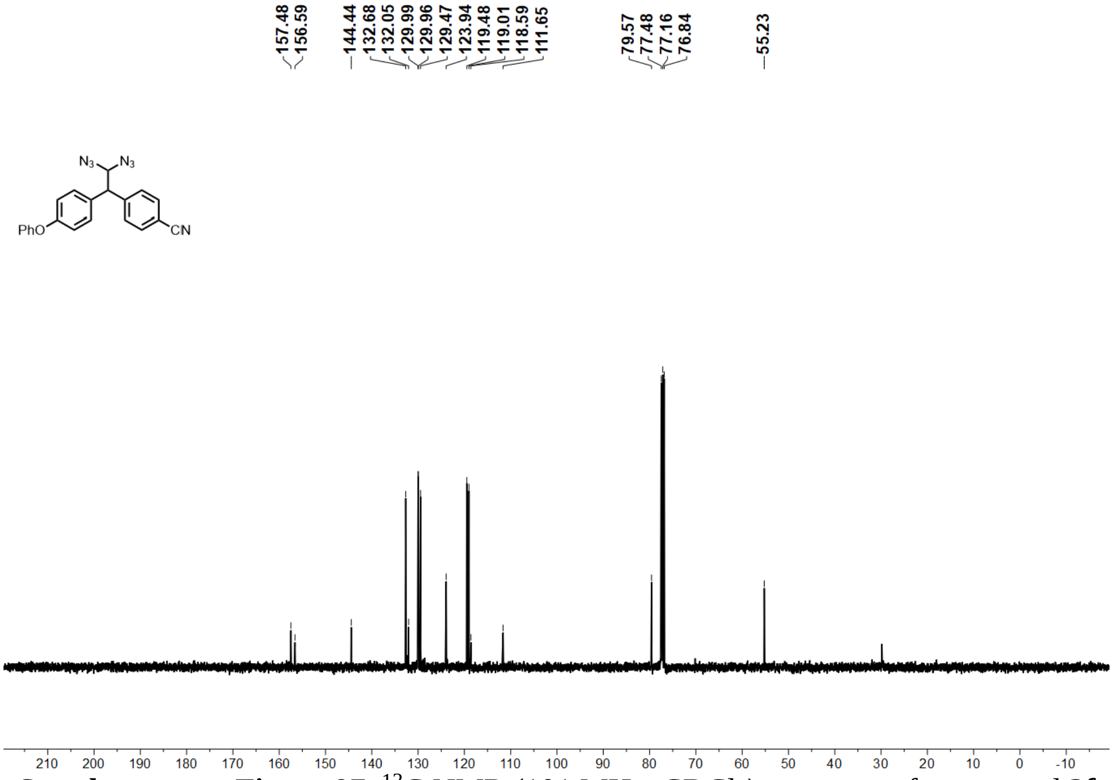

**Supplementary Figure 97.** $^{13}C$ NMR (101 MHz, $CDCl_3$) spectrum of compound **2f**

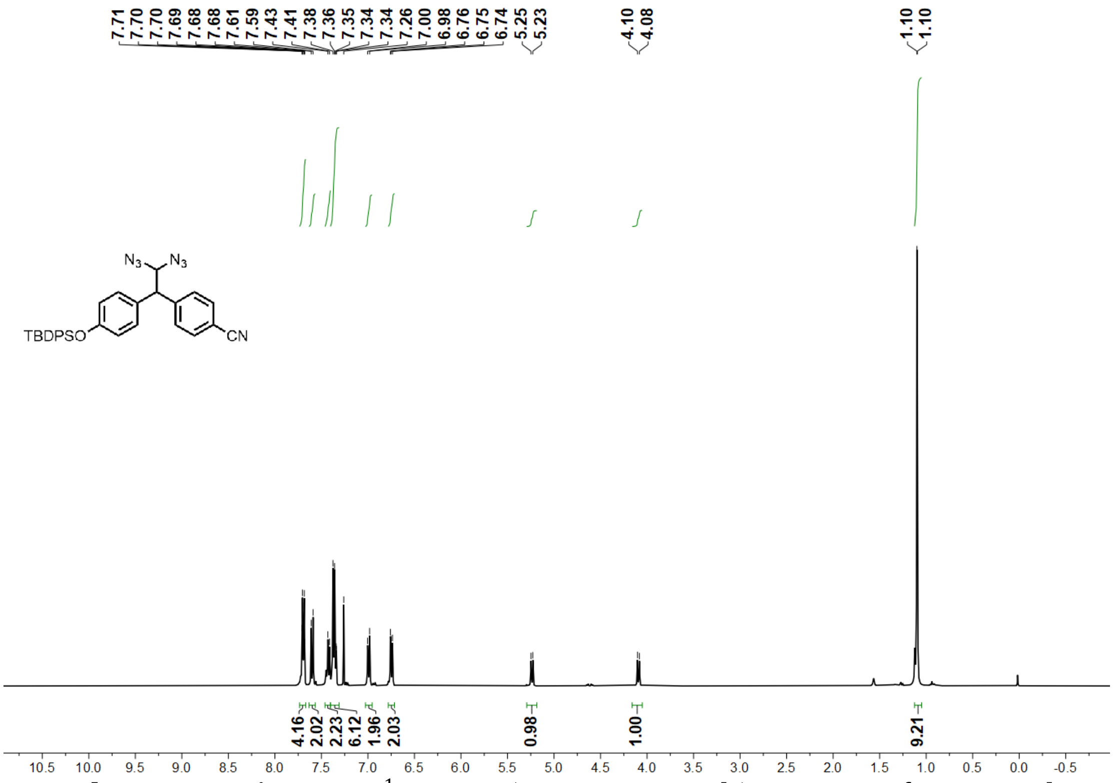


**Supplementary Figure 98.** $^{1}H$ NMR (400 MHz, $CDCl_3$) spectrum of compound **2g**

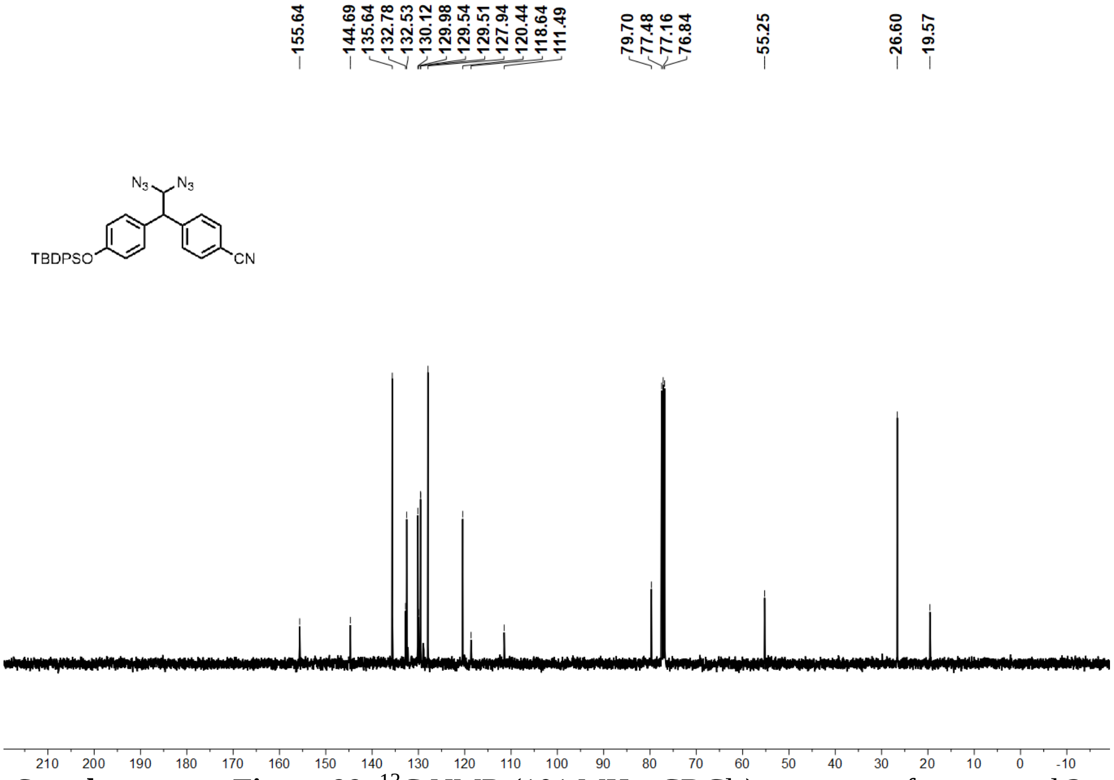


**Supplementary Figure 99.** $^{13}C$ NMR (101 MHz, $CDCl_3$) spectrum of compound **2g**

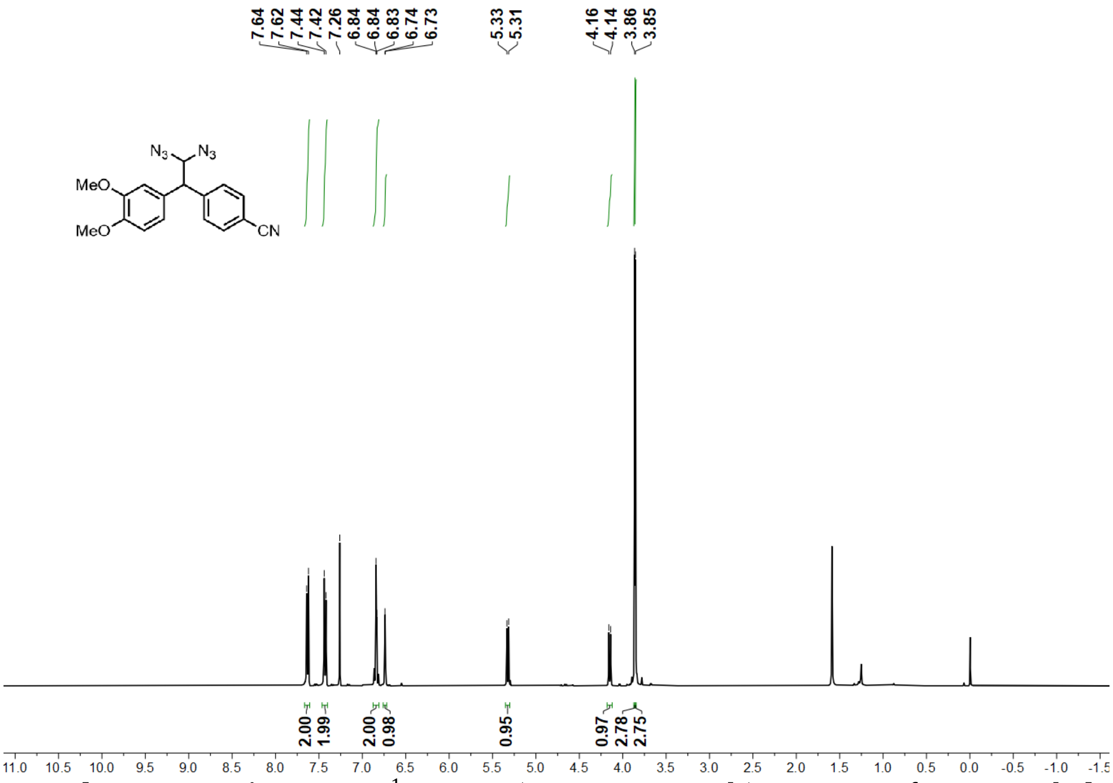

**Supplementary Figure 100.** $^{1}$H NMR (400 MHz, $CDCl_3$) spectrum of compound **2h**

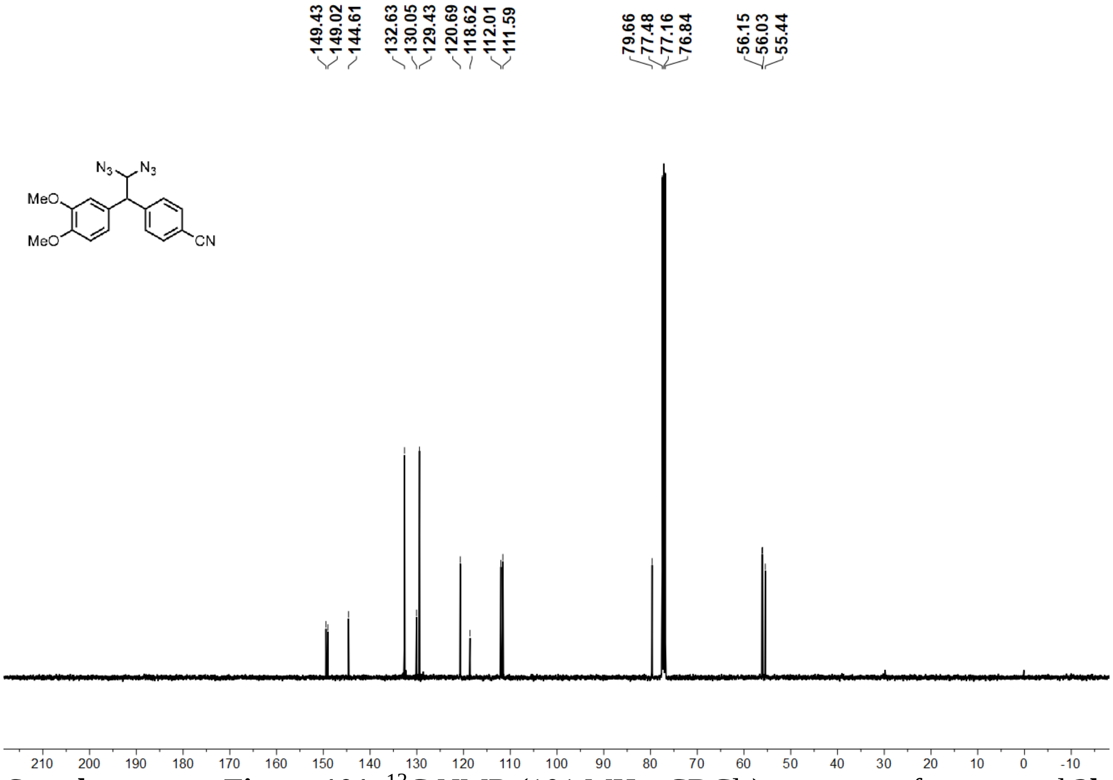

**Supplementary Figure 101.** $^{13}$C NMR (101 MHz, $CDCl_3$) spectrum of compound **2h**

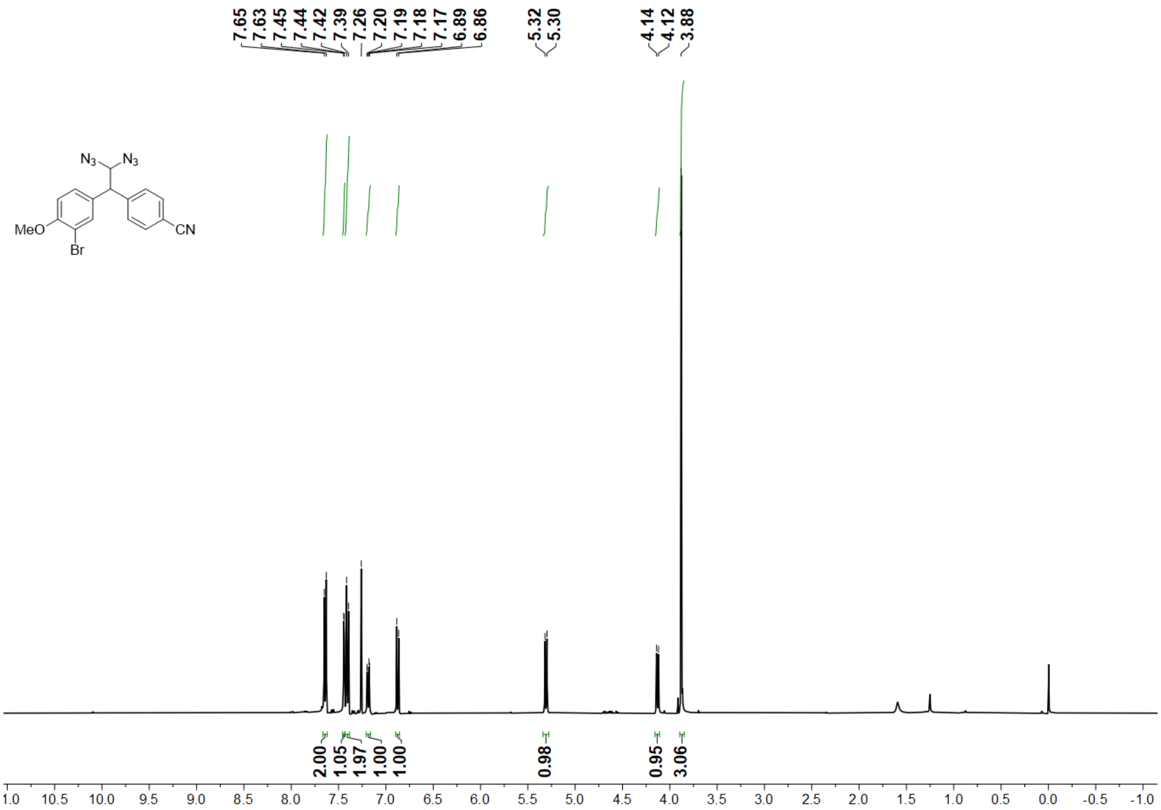


**Supplementary Figure 102.** $^{1}$H NMR (400 MHz, $CDCl_3$) spectrum of compound **2i**

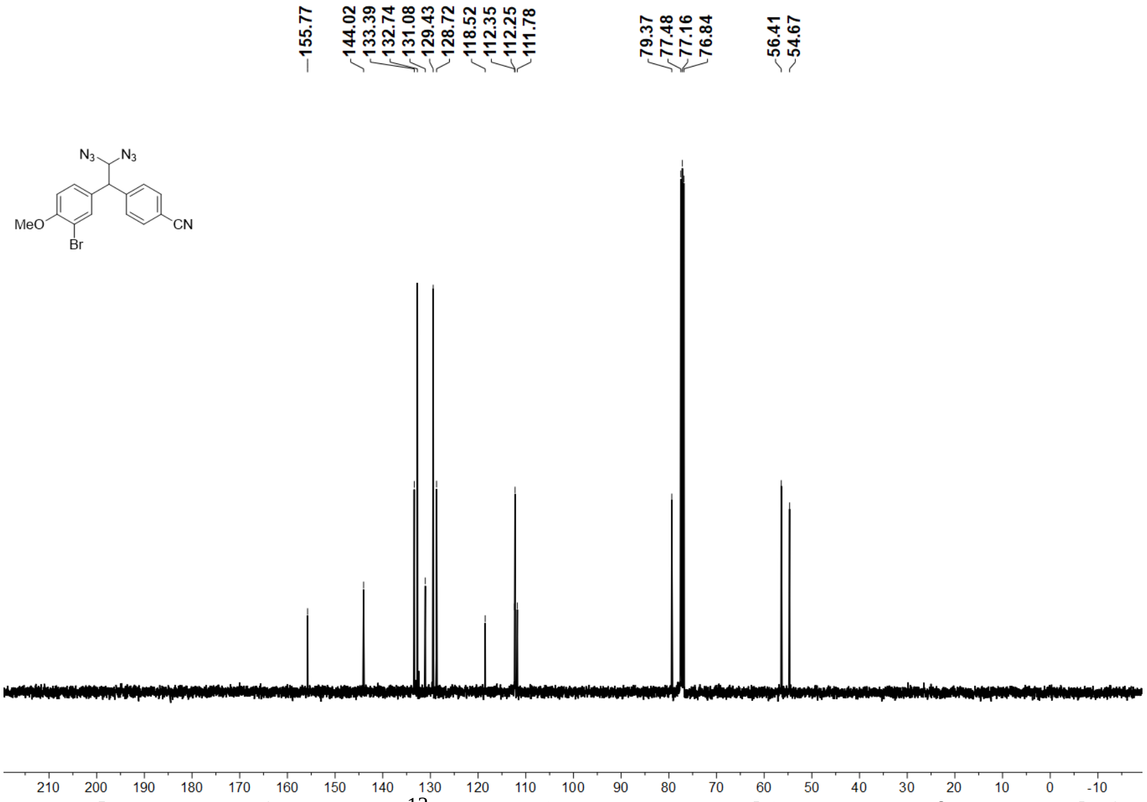


**Supplementary Figure 103.** $^{13}$C NMR (101 MHz, $CDCl_3$) spectrum of compound **2i**

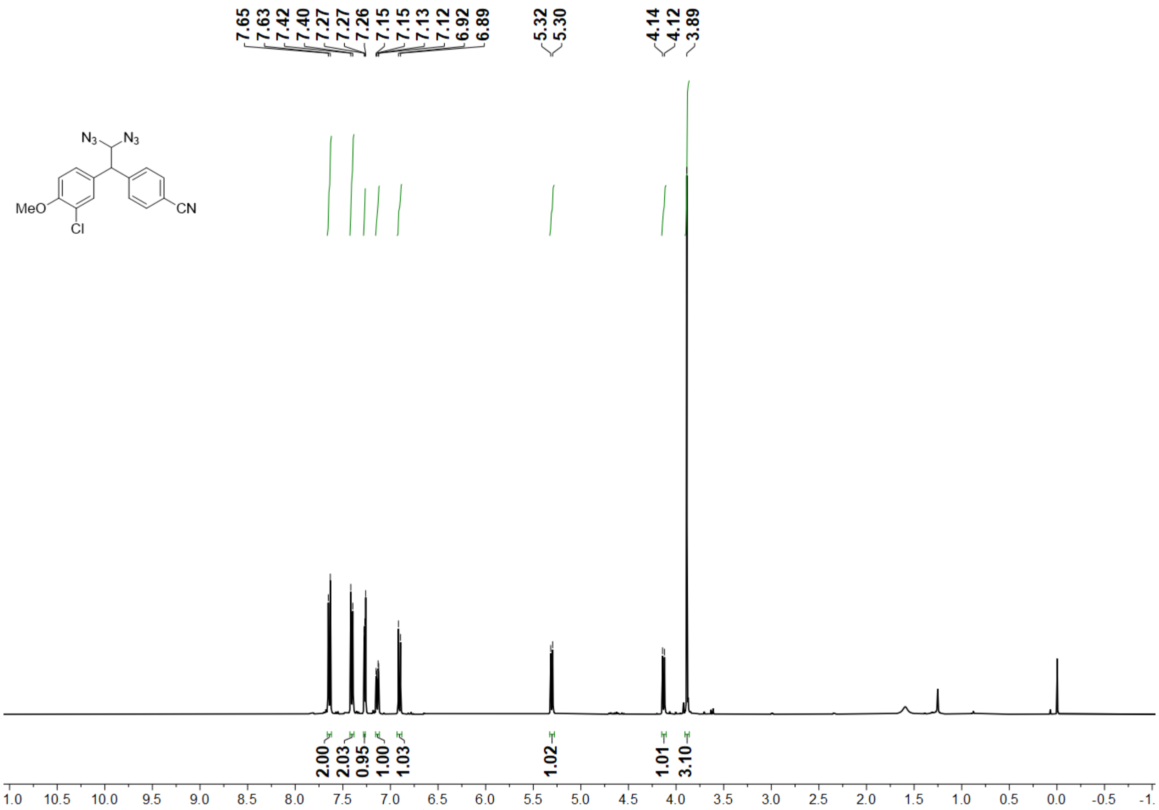


**Supplementary Figure 104.** $^{1}H$ NMR (400 MHz, $CDCl_3$) spectrum of compound **2j**

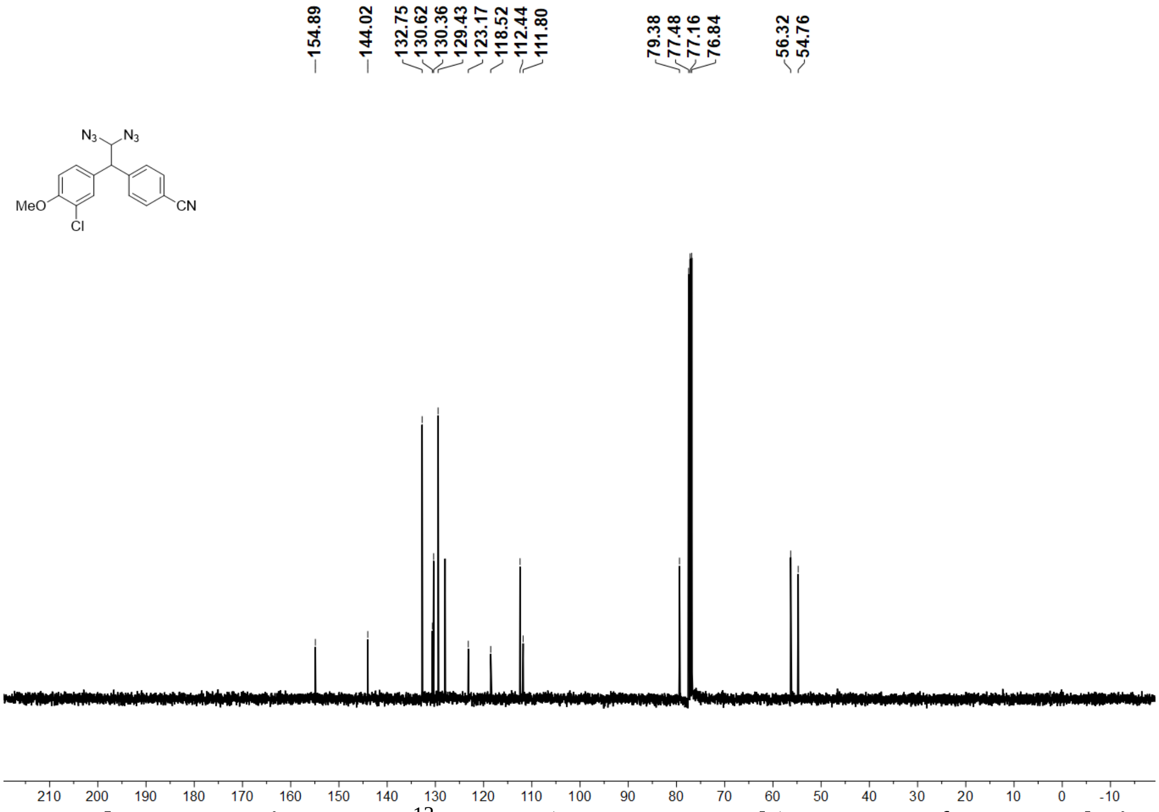


**Supplementary Figure 105.** $^{13}C$ NMR (101 MHz, $CDCl_3$) spectrum of compound **2j**

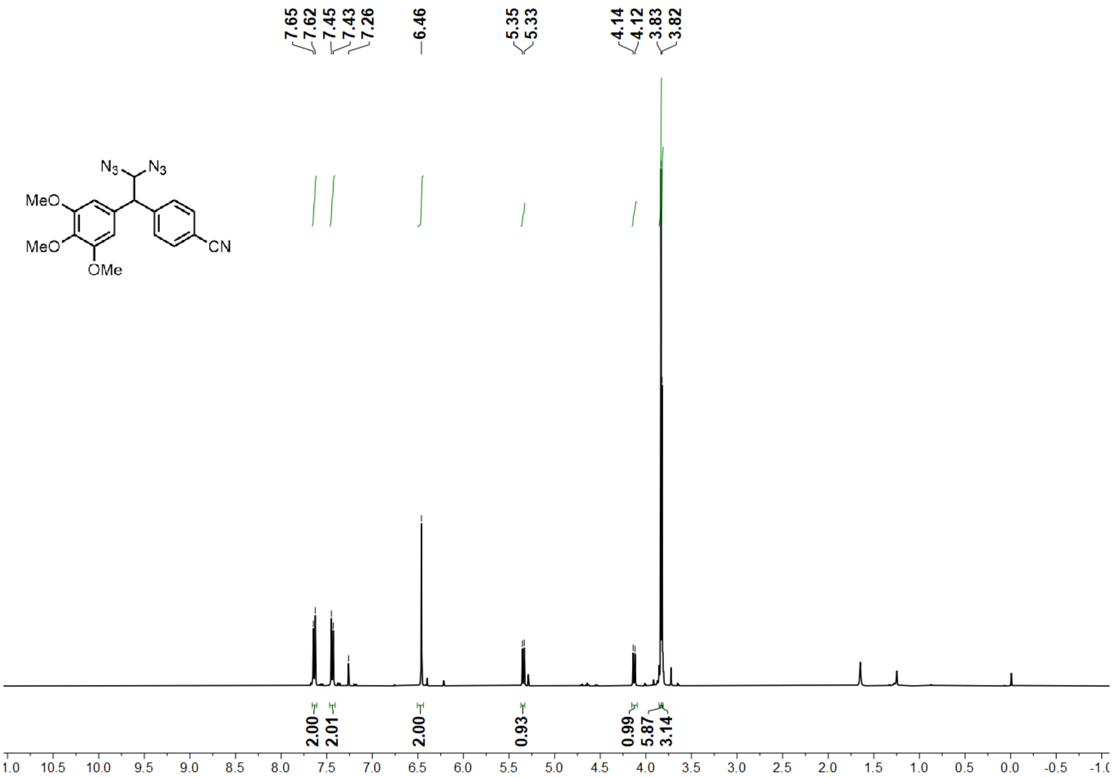


**Supplementary Figure 106.** $^{1}$H NMR (400 MHz, $CDCl_3$) spectrum of compound **2k**

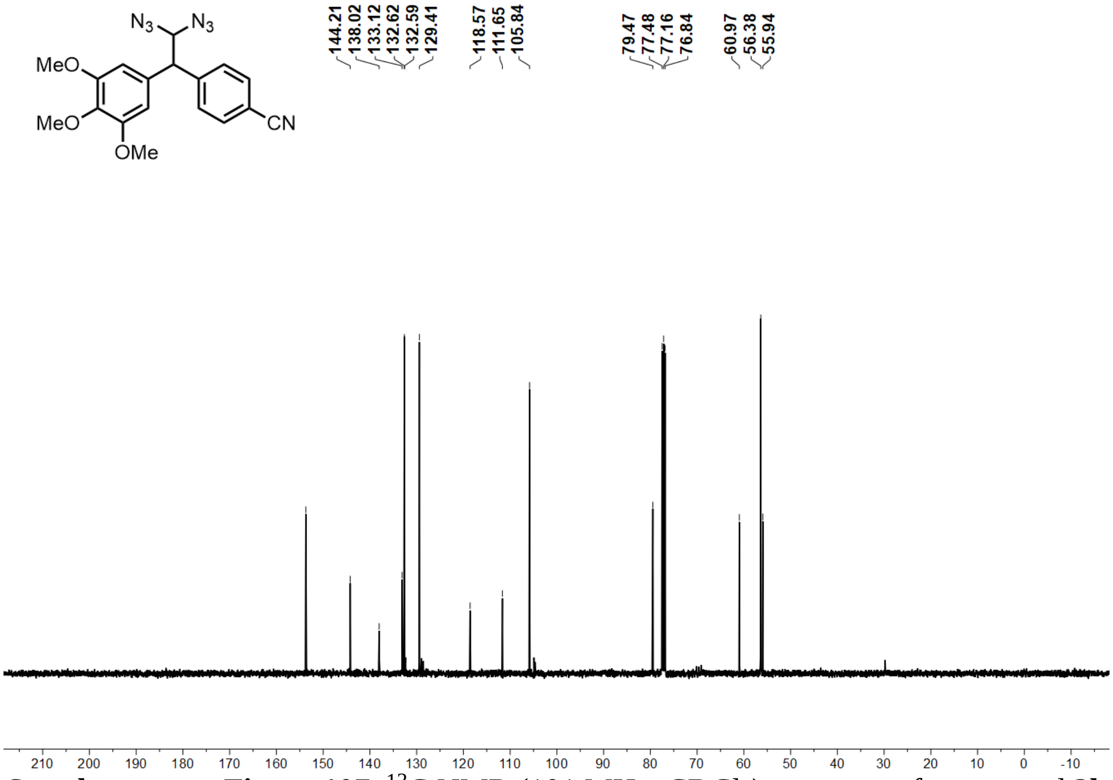


**Supplementary Figure 107.** $^{13}$C NMR (101 MHz, $CDCl_3$) spectrum of compound **2k**

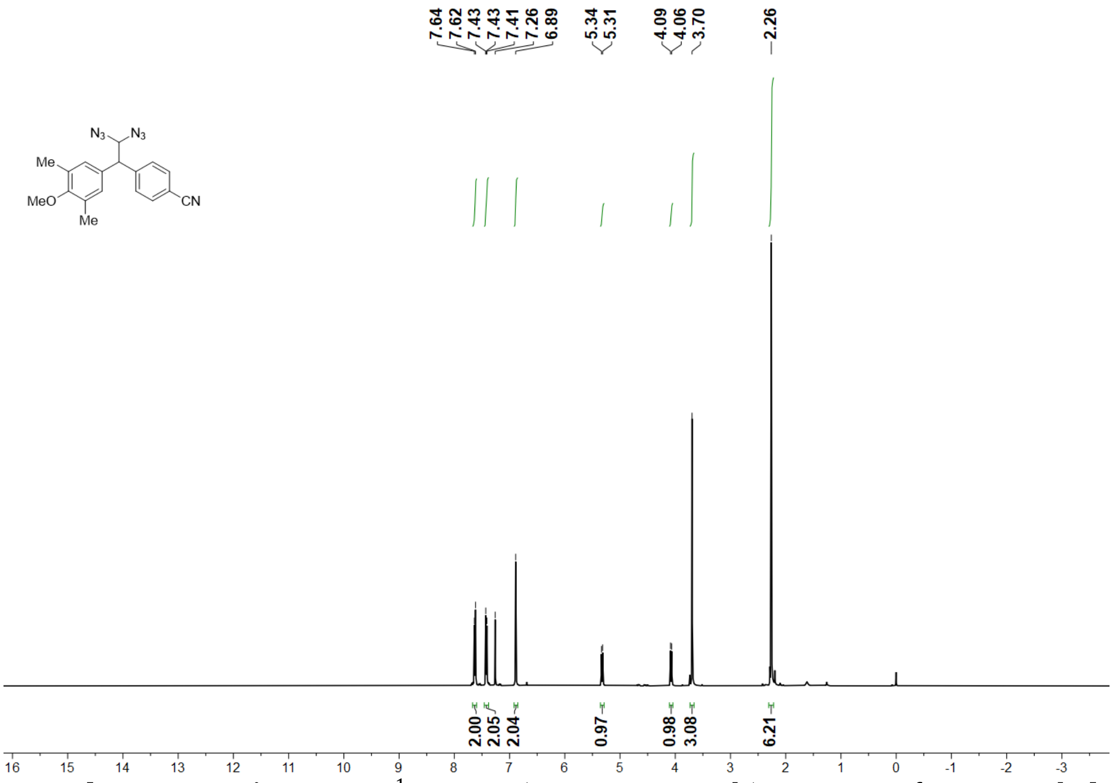


**Supplementary Figure 108.** $^{1}$H NMR (400 MHz, $CDCl_3$) spectrum of compound **2l**

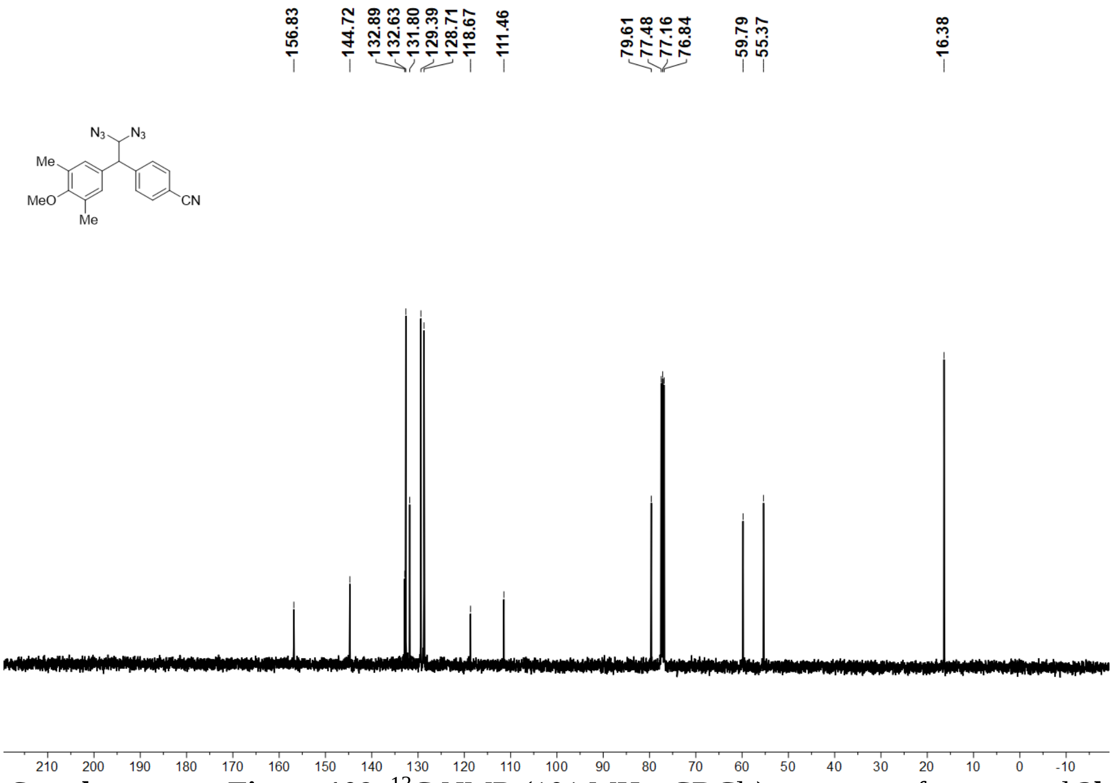


**Supplementary Figure 109.** $^{13}$C NMR (101 MHz, $CDCl_3$) spectrum of compound **2l**

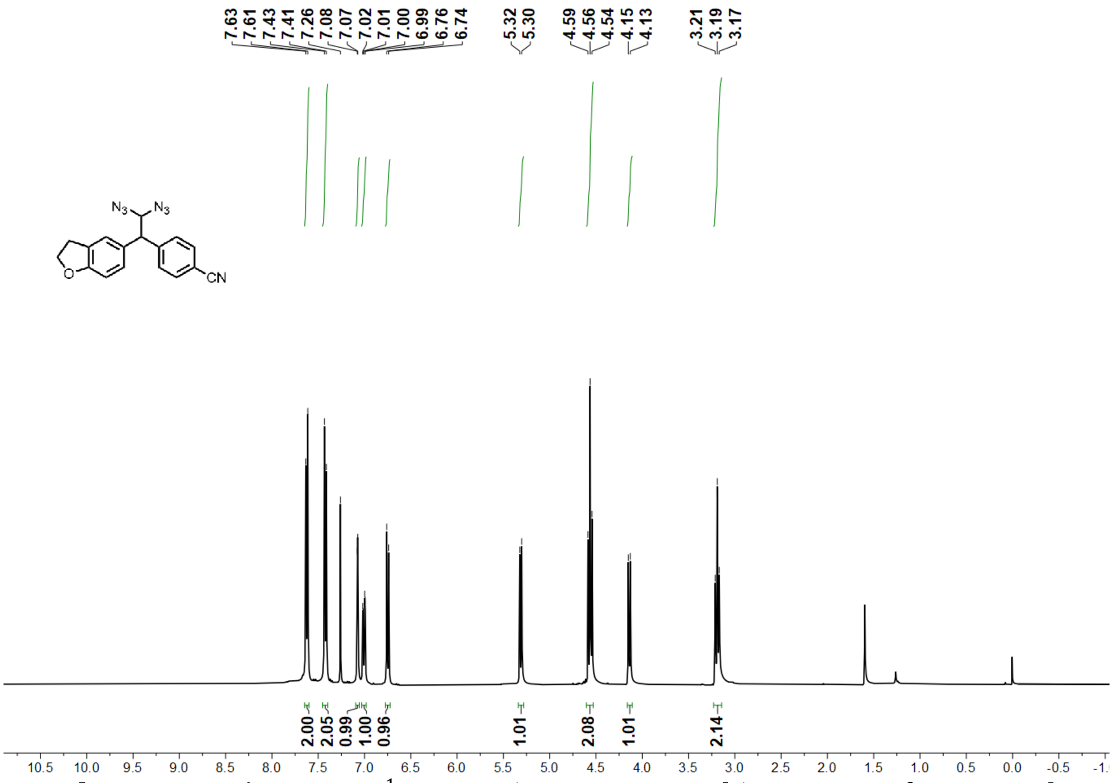


**Supplementary Figure 110.** $^{1}$H NMR (400 MHz, $CDCl_3$) spectrum of compound **2m**

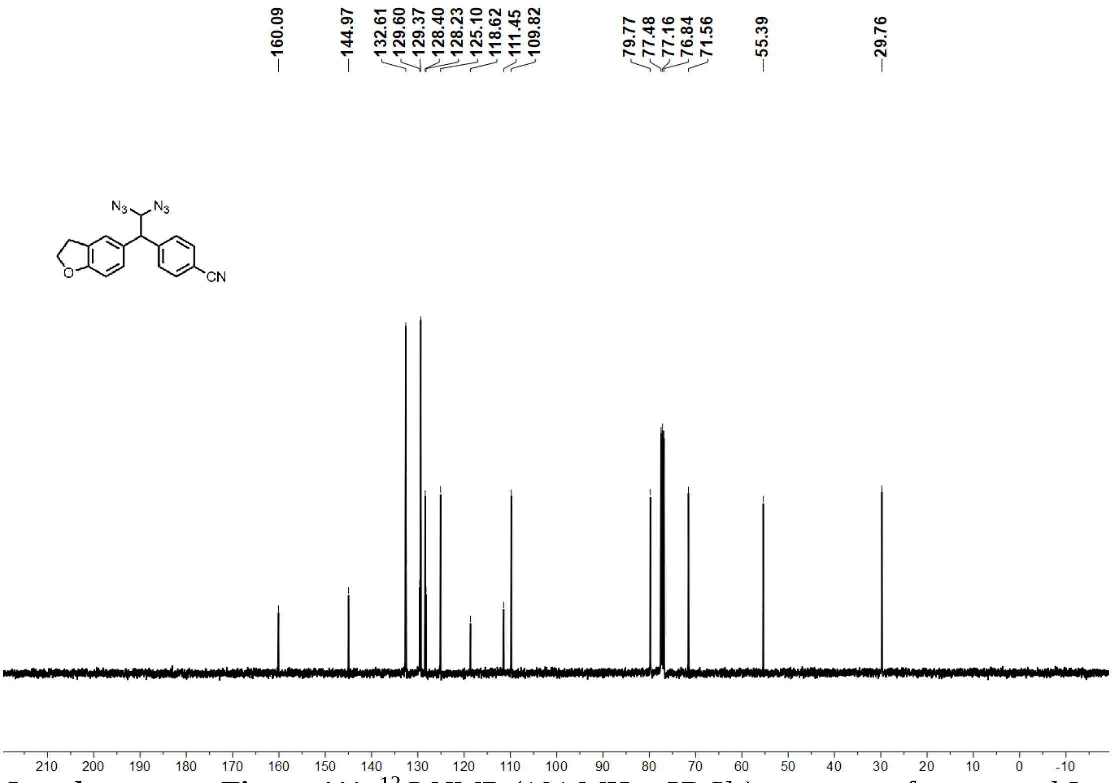


**Supplementary Figure 111.** $^{13}$C NMR (101 MHz, $CDCl_3$) spectrum of compound **2m**

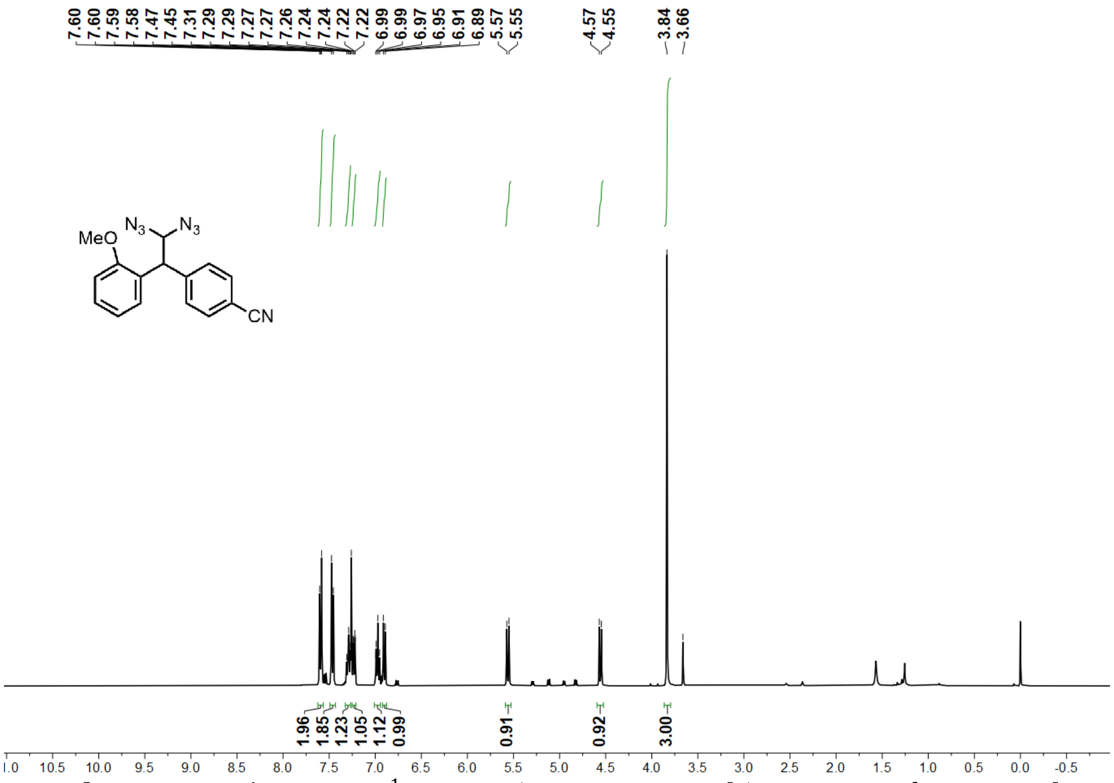


**Supplementary Figure 112.** $^{1}$H NMR (400 MHz, $CDCl_3$) spectrum of compound **2n**

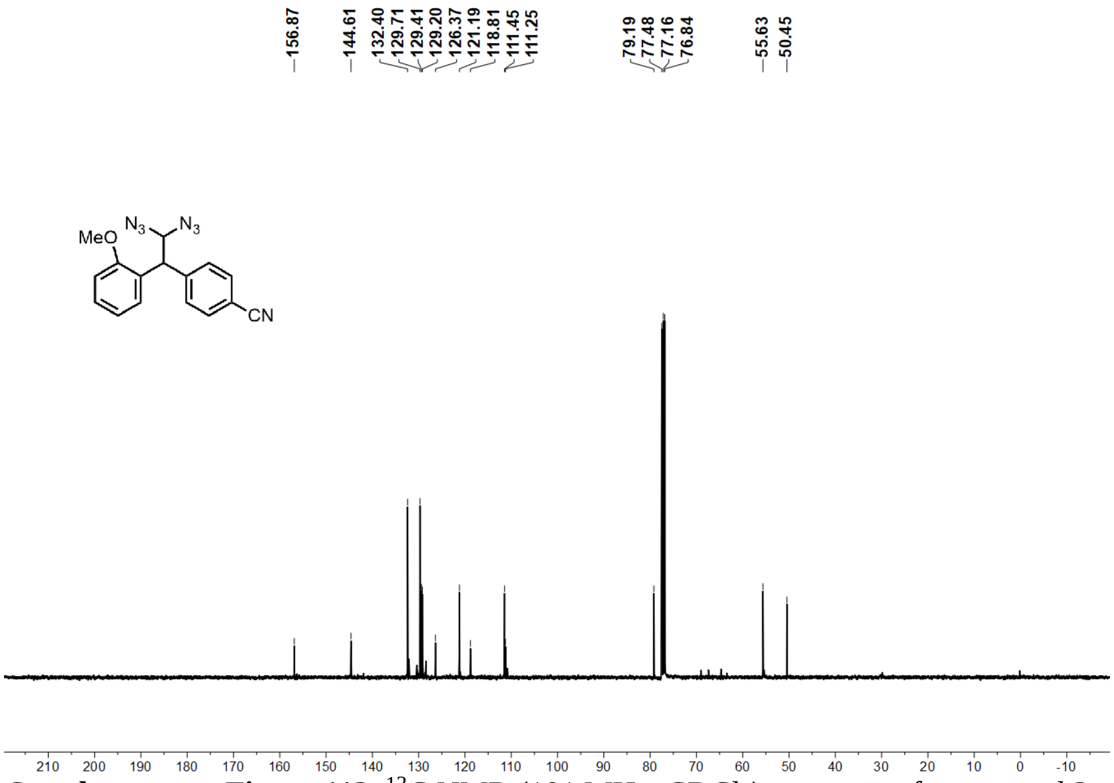


**Supplementary Figure 113.** $^{13}$C NMR (101 MHz, $CDCl_3$) spectrum of compound **2n**

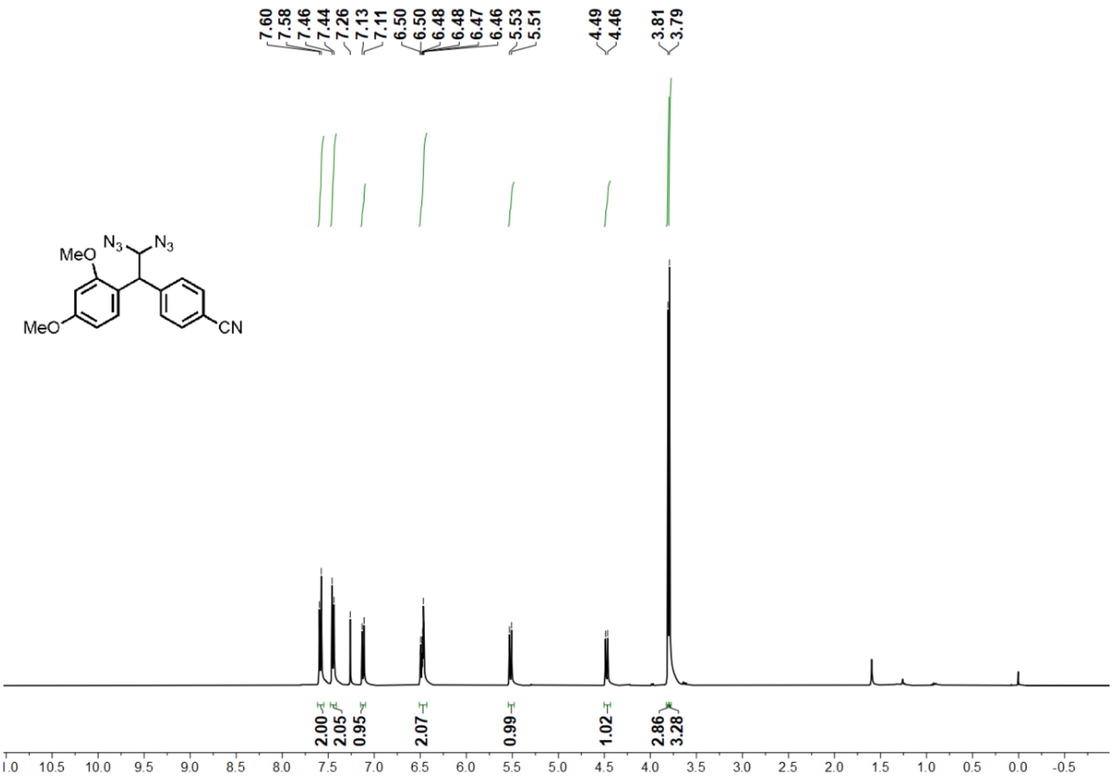


**Supplementary Figure 114.** $^{1}$H NMR (400 MHz, $CDCl_3$) spectrum of compound **2o**

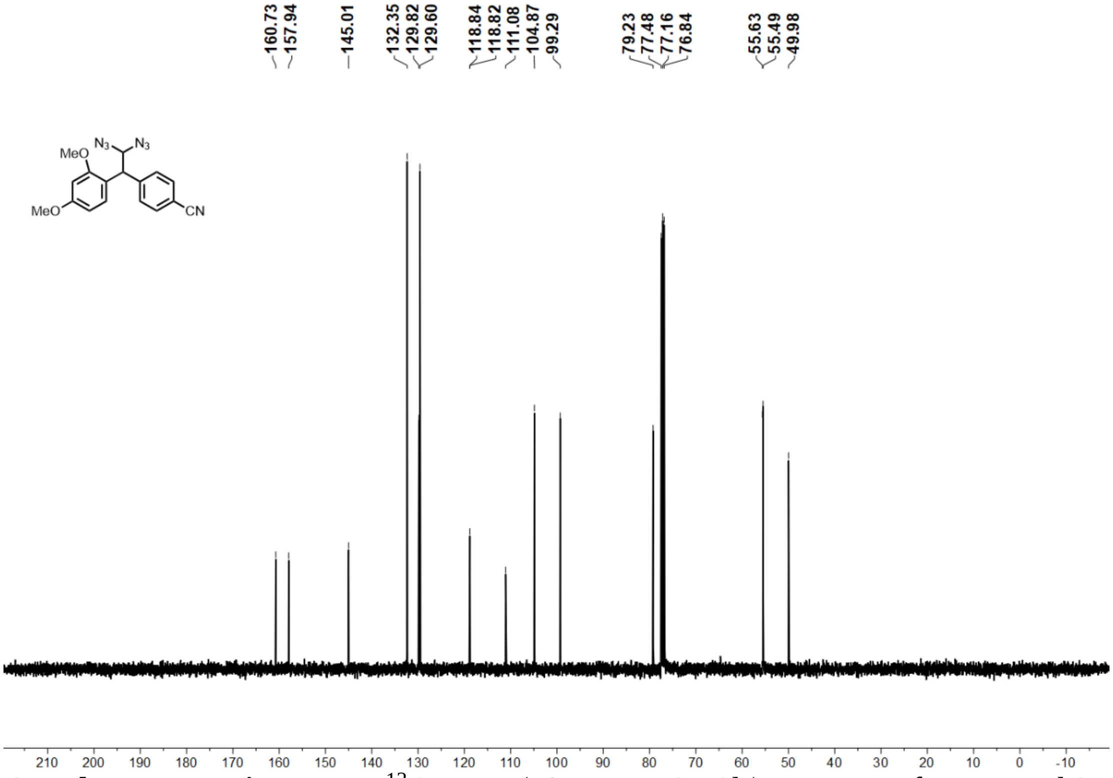


**Supplementary Figure 115.** $^{13}$C NMR (101 MHz, $CDCl_3$) spectrum of compound **2o**

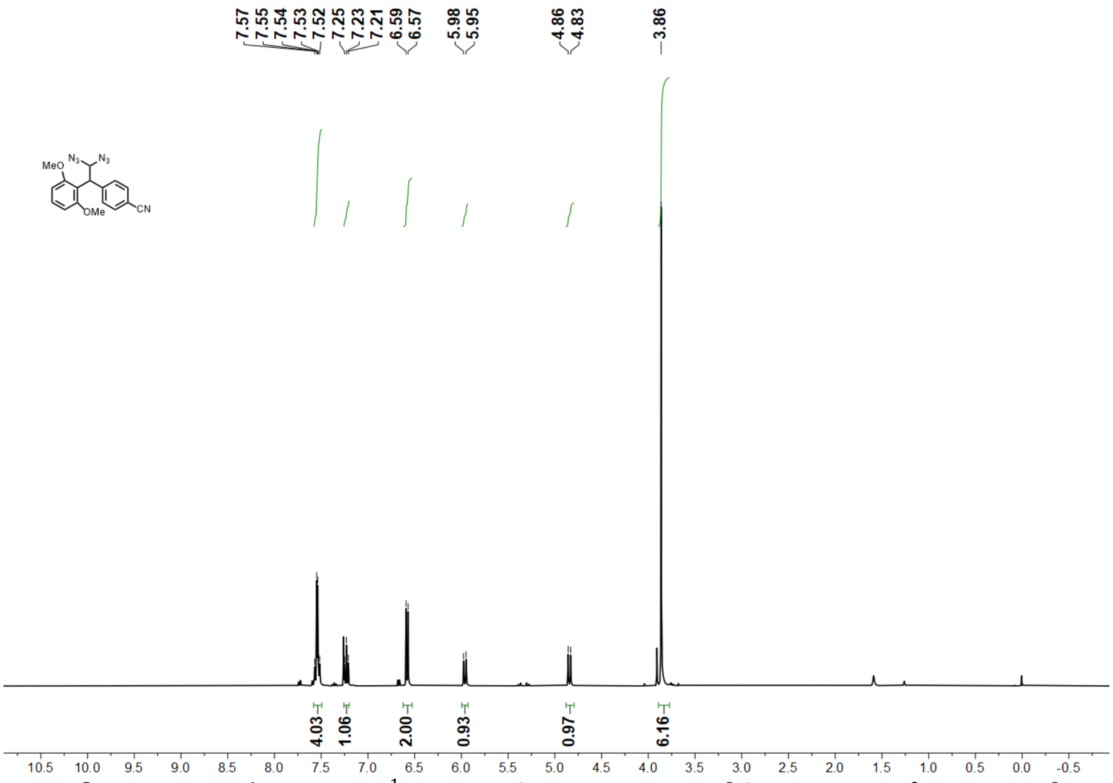


**Supplementary Figure 116.** $^{1}$H NMR (400 MHz, $CDCl_3$) spectrum of compound **2p**

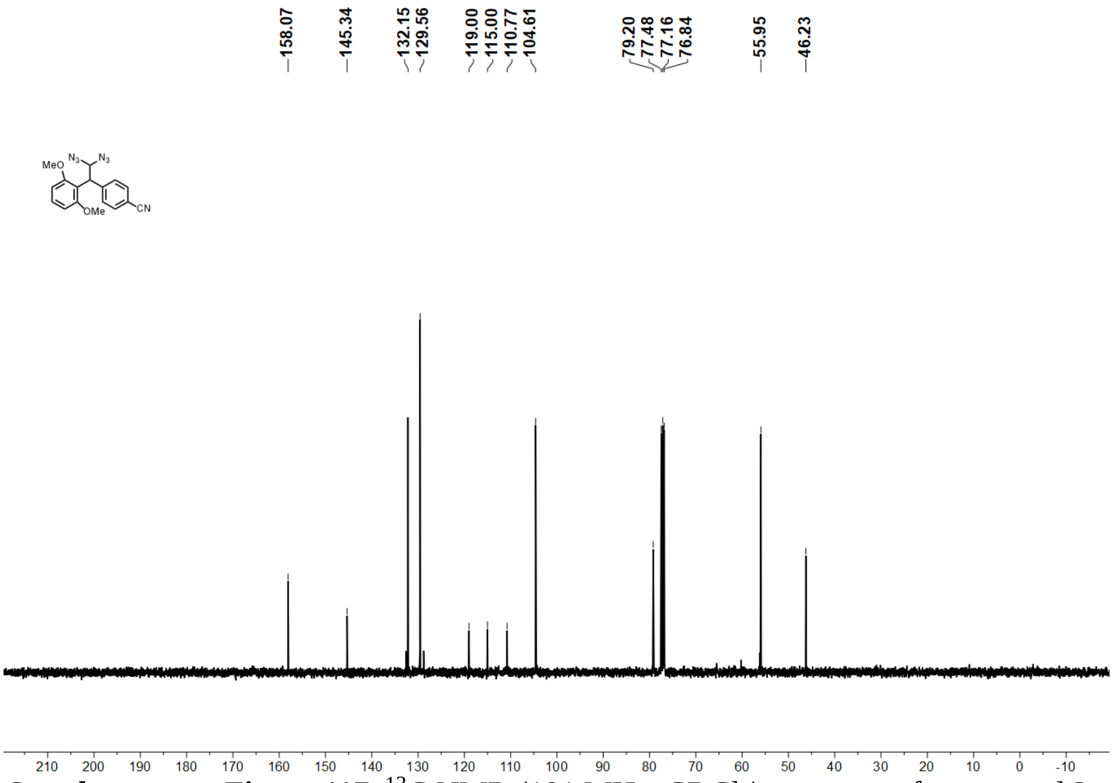


**Supplementary Figure 117.** $^{13}$C NMR (101 MHz, $CDCl_3$) spectrum of compound **2p**

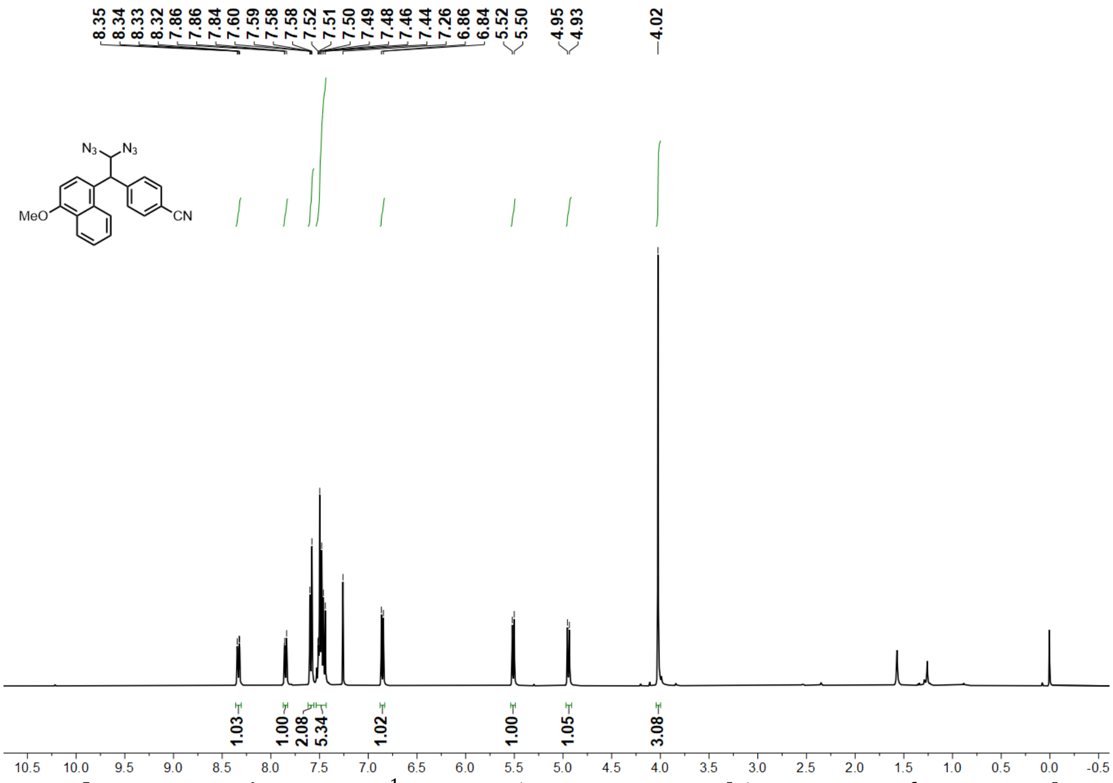

**Supplementary Figure 118.** $^{1}$H NMR (400 MHz, $CDCl_3$) spectrum of compound **2q**

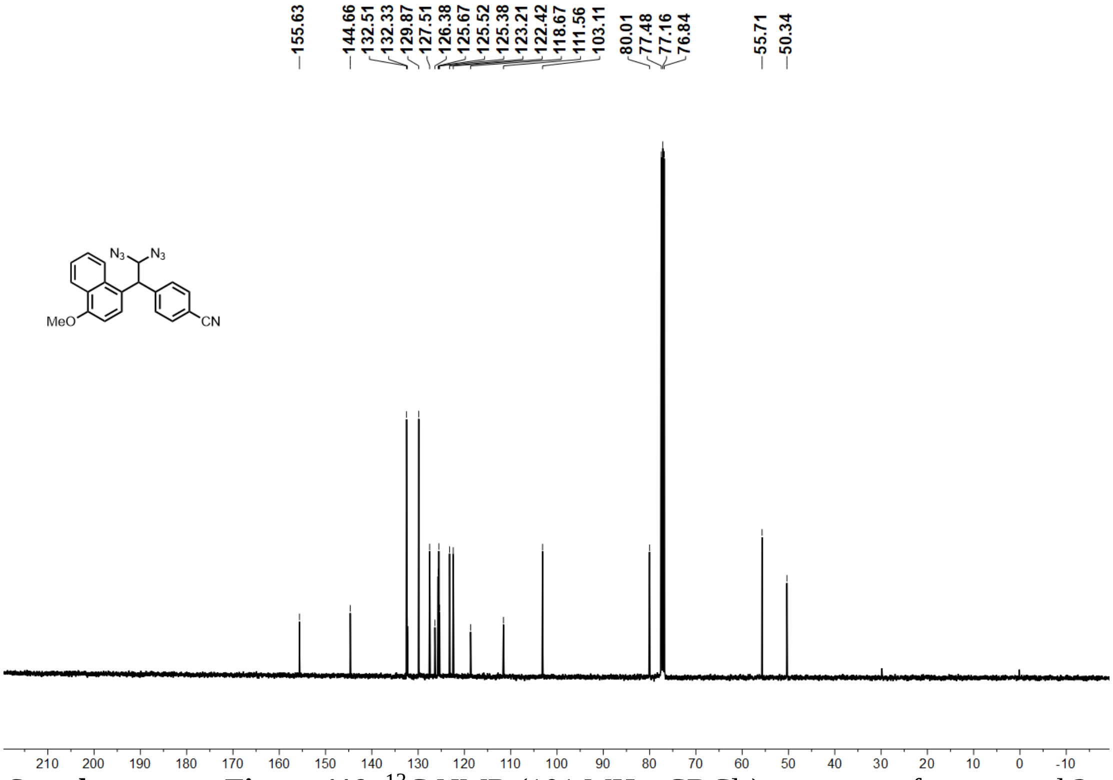

**Supplementary Figure 119.** $^{13}$C NMR (101 MHz, $CDCl_3$) spectrum of compound **2q**

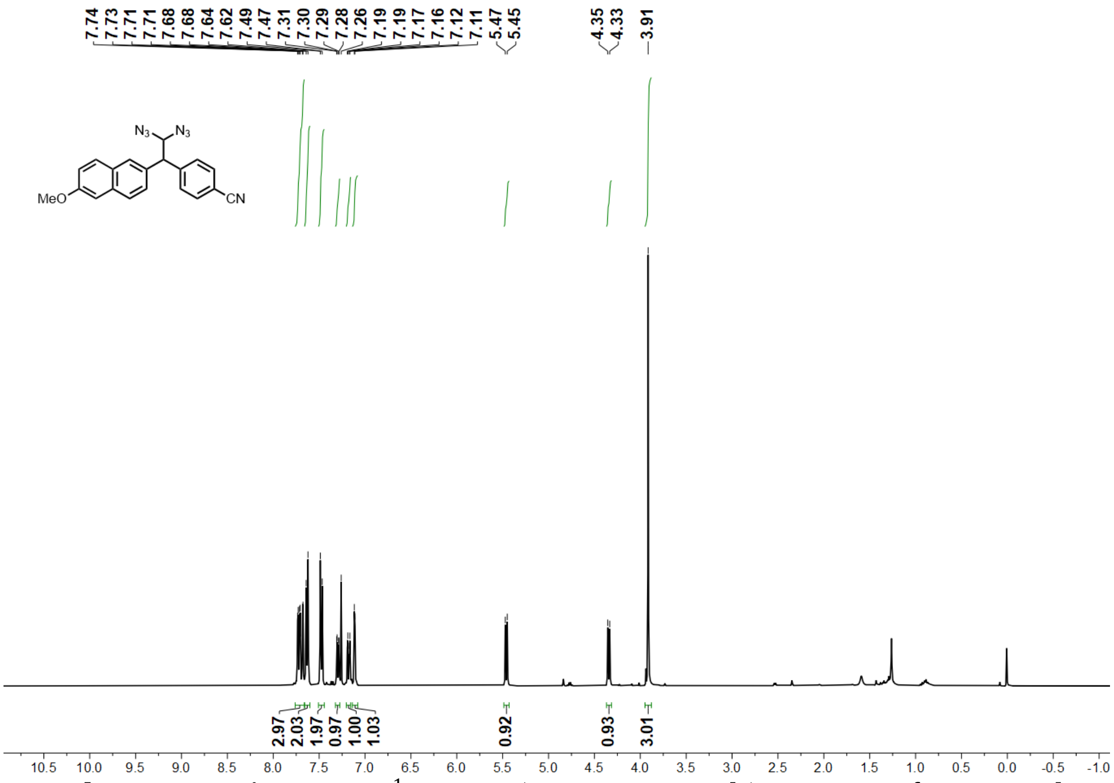

**Supplementary Figure 120.** $^{1}$H NMR (400 MHz, $CDCl_3$) spectrum of compound **2r**

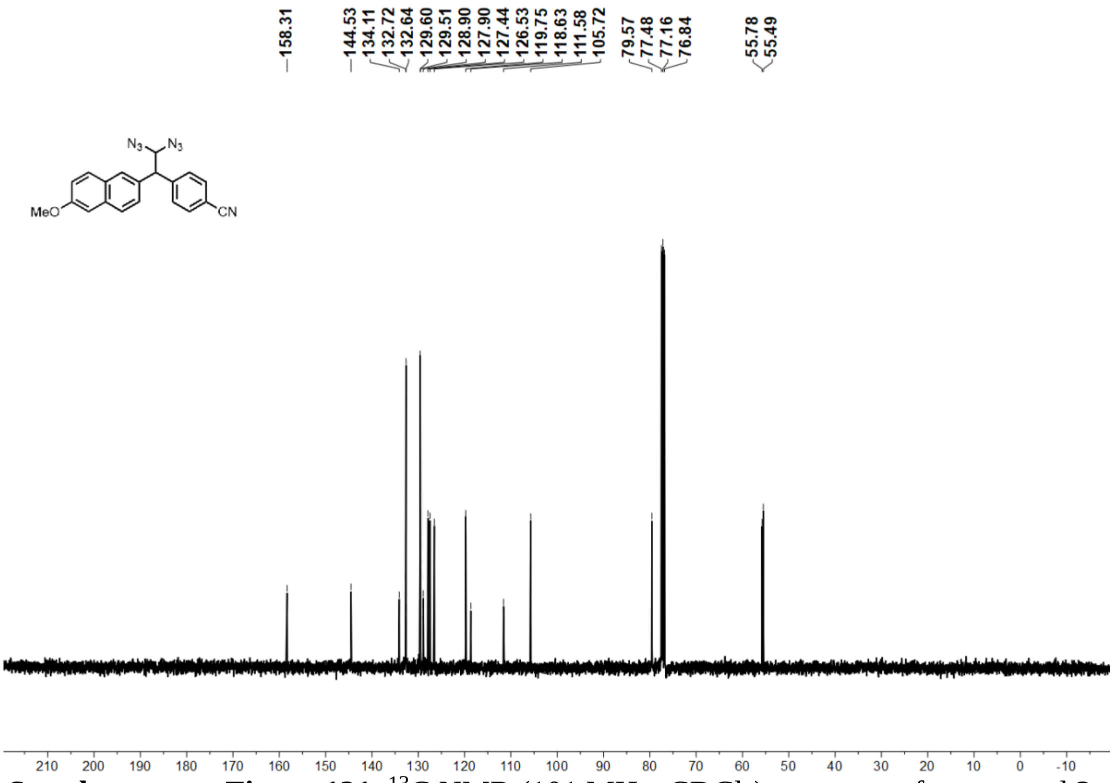

**Supplementary Figure 121.** $^{13}$C NMR (101 MHz, $CDCl_3$) spectrum of compound **2r**

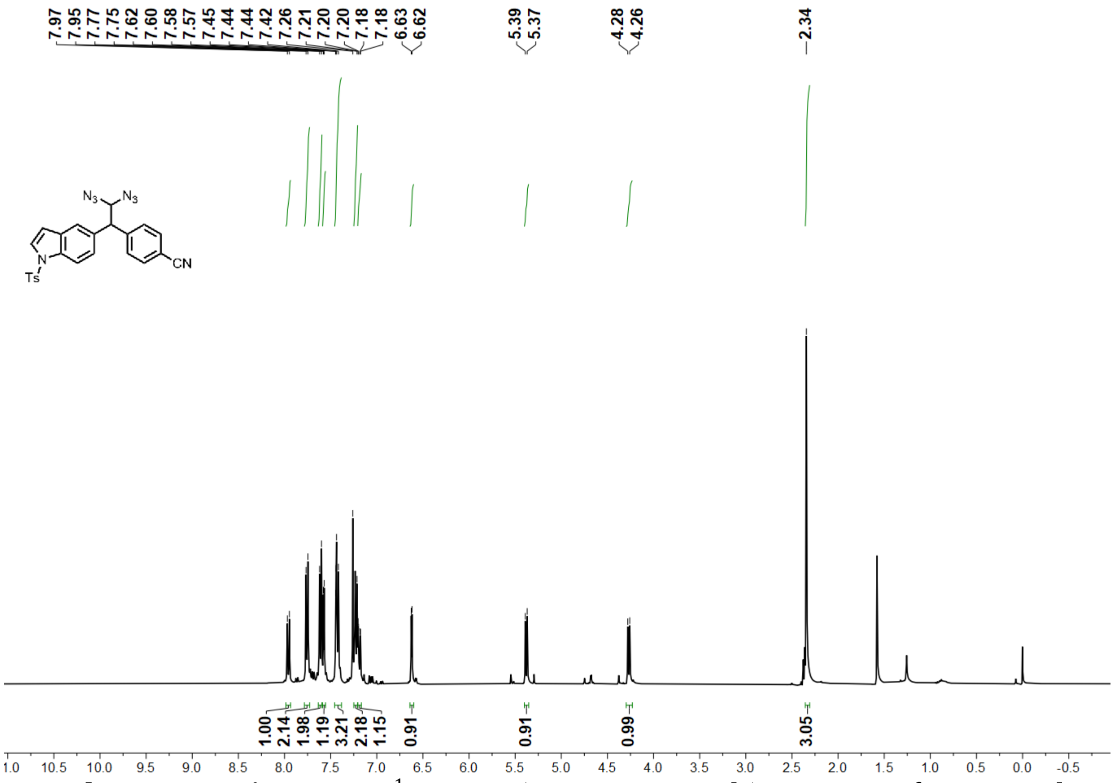

**Supplementary Figure 122.** $^{1}H$ NMR (400 MHz, $CDCl_3$) spectrum of compound **2s**

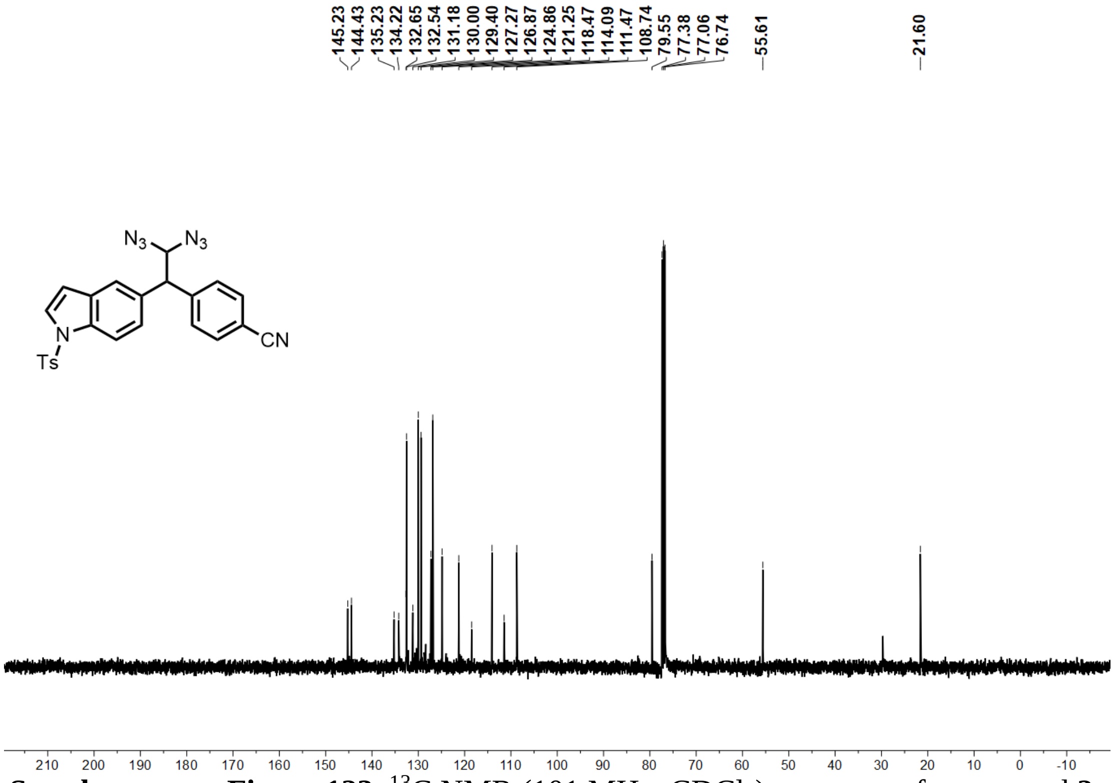

**Supplementary Figure 123.** $^{13}C$ NMR (101 MHz, $CDCl_3$) spectrum of compound **2s**

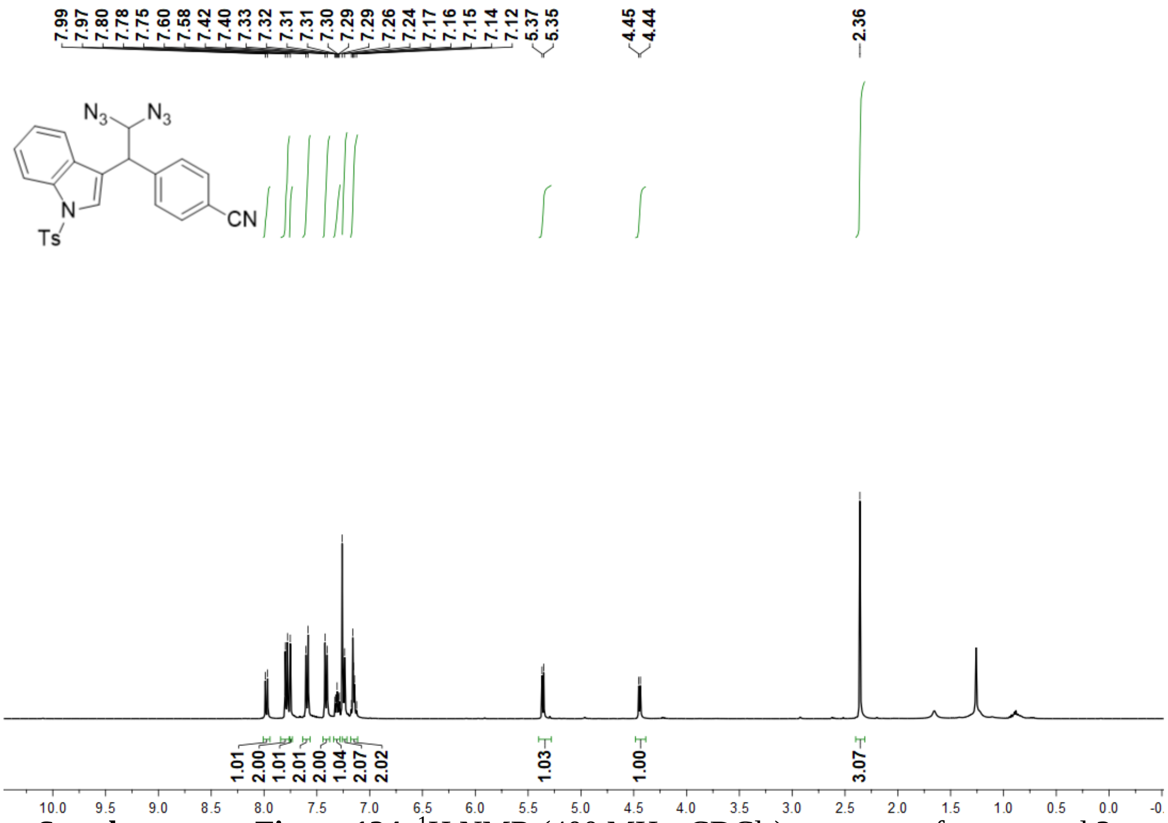

**Supplementary Figure 124.** $^{1}$H NMR (400 MHz, $CDCl_3$) spectrum of compound **2t**

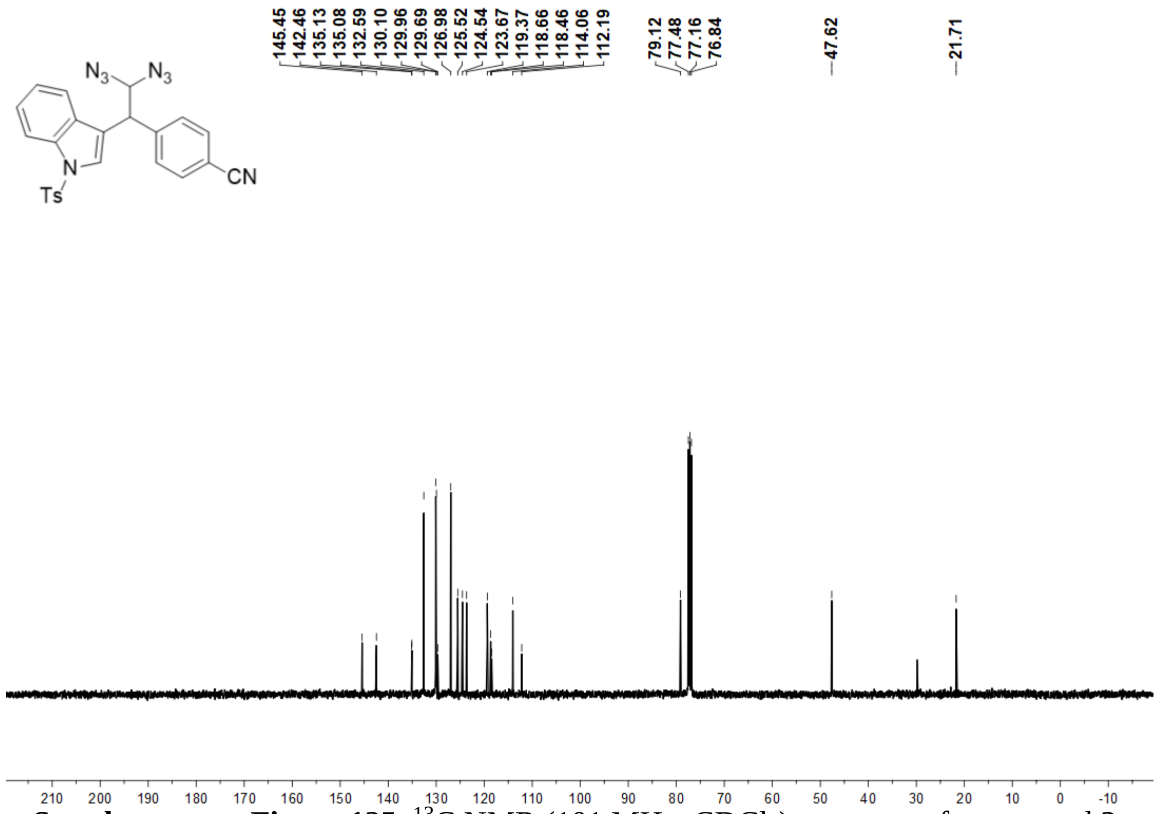

**Supplementary Figure 125.** $^{13}$C NMR (101 MHz, $CDCl_3$) spectrum of compound **2t**

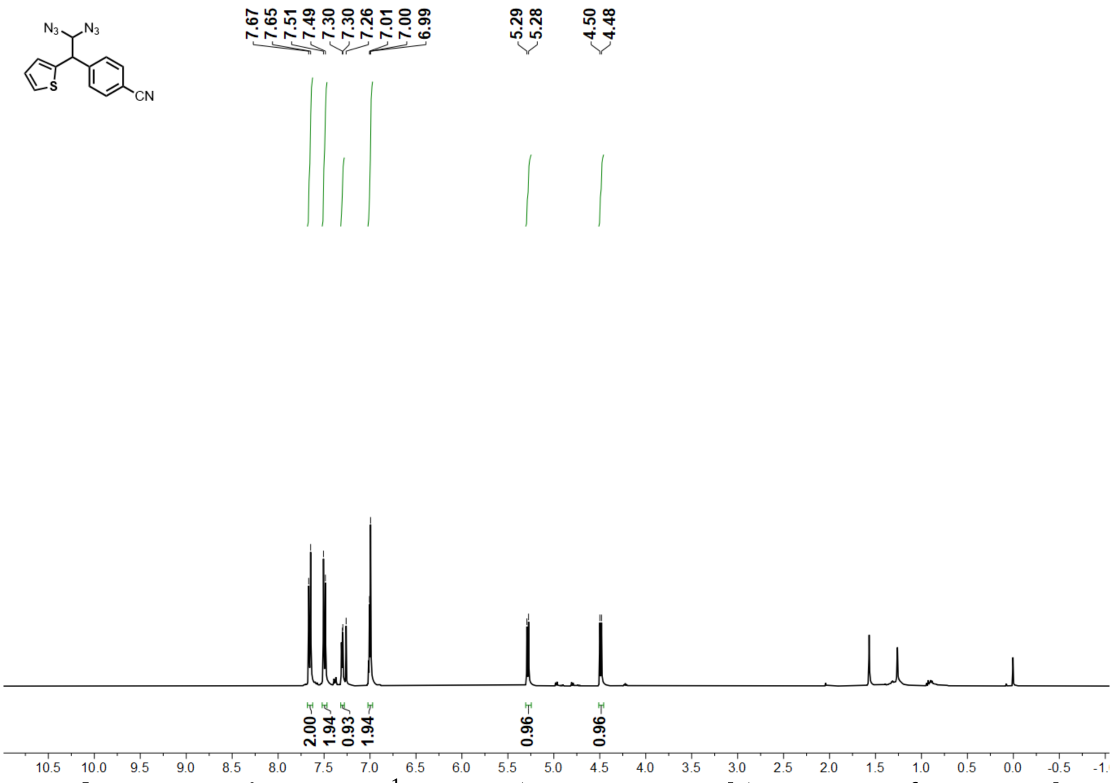

**Supplementary Figure 126.** $^{1}H$ NMR (400 MHz, $CDCl_3$) spectrum of compound **2u**

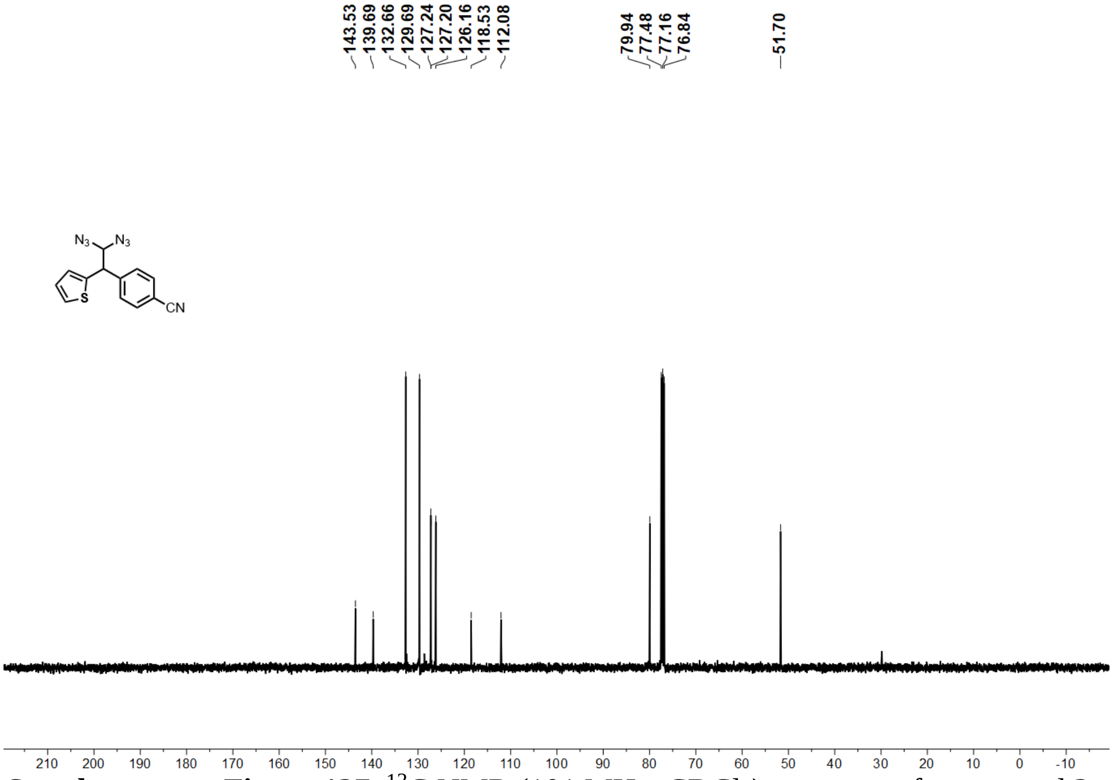

**Supplementary Figure 127.** $^{13}C$ NMR (101 MHz, $CDCl_3$) spectrum of compound **2u**

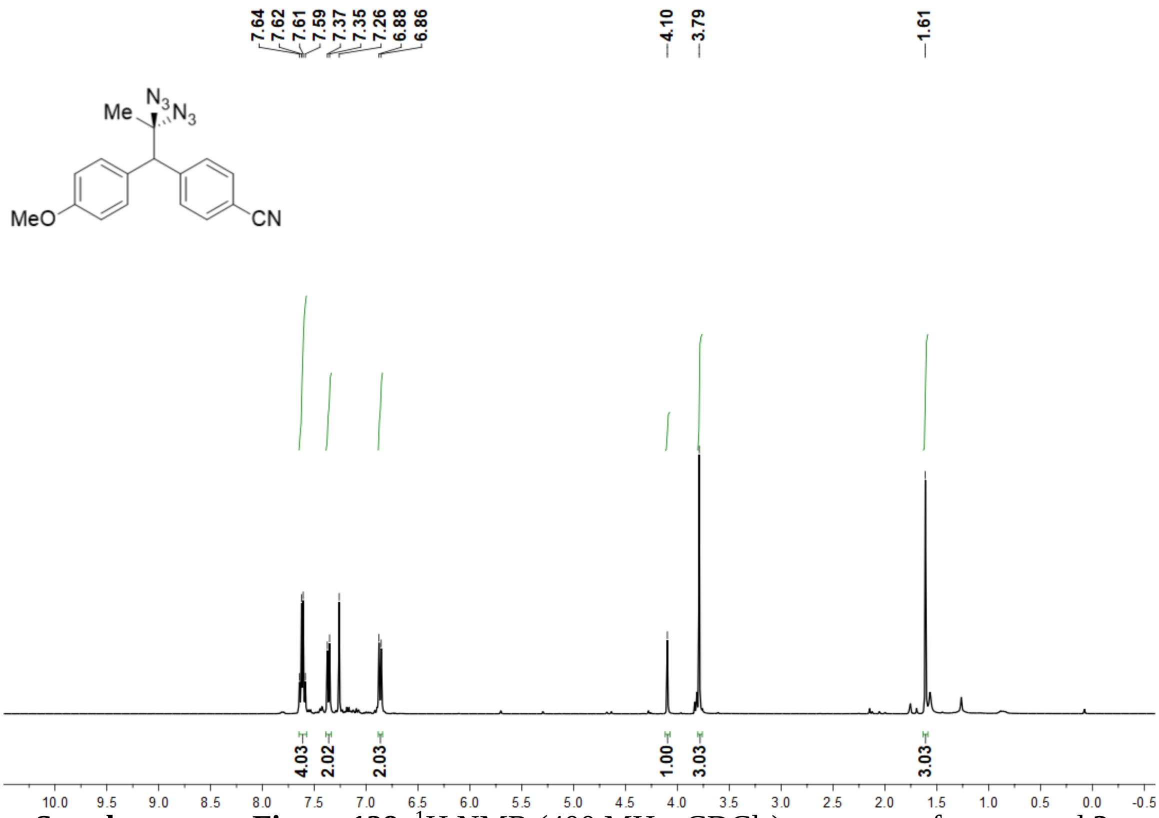


**Supplementary Figure 128.** $^{1}$H NMR (400 MHz, $CDCl_3$) spectrum of compound **2v**

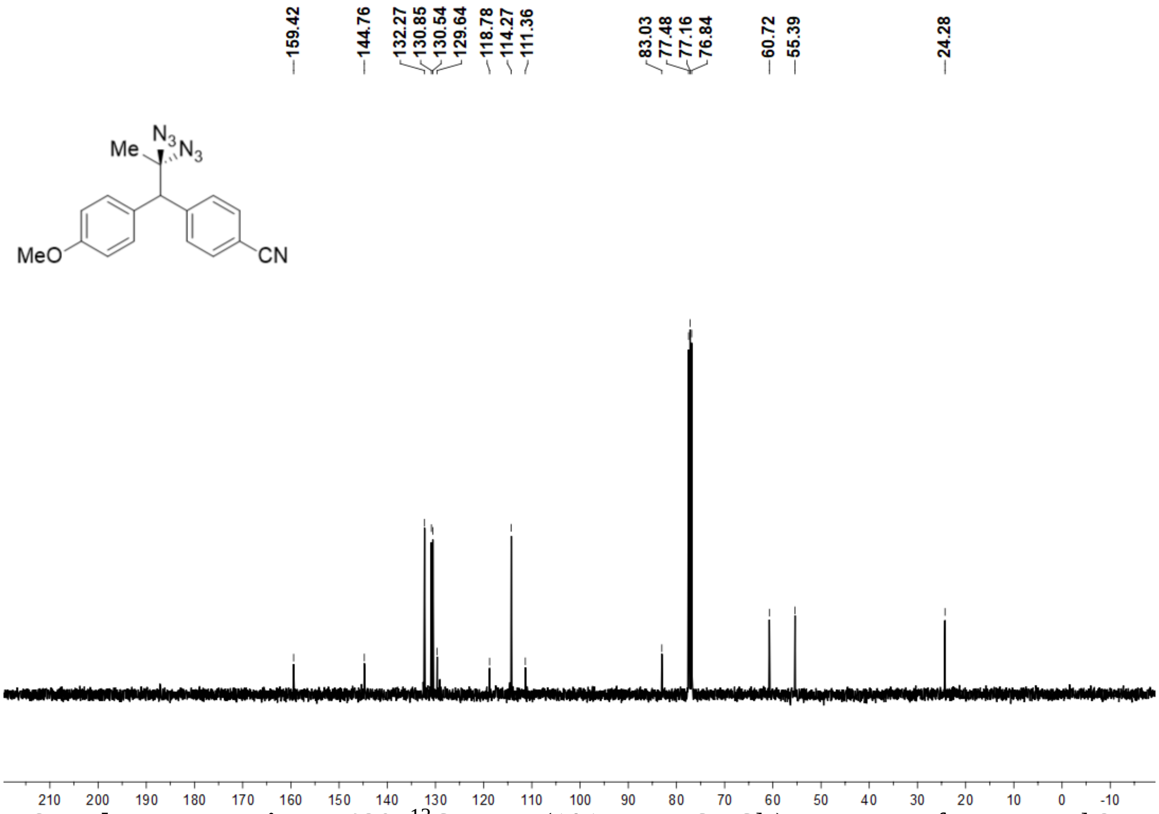


**Supplementary Figure 129.** $^{13}$C NMR (101 MHz, $CDCl_3$) spectrum of compound **2v**

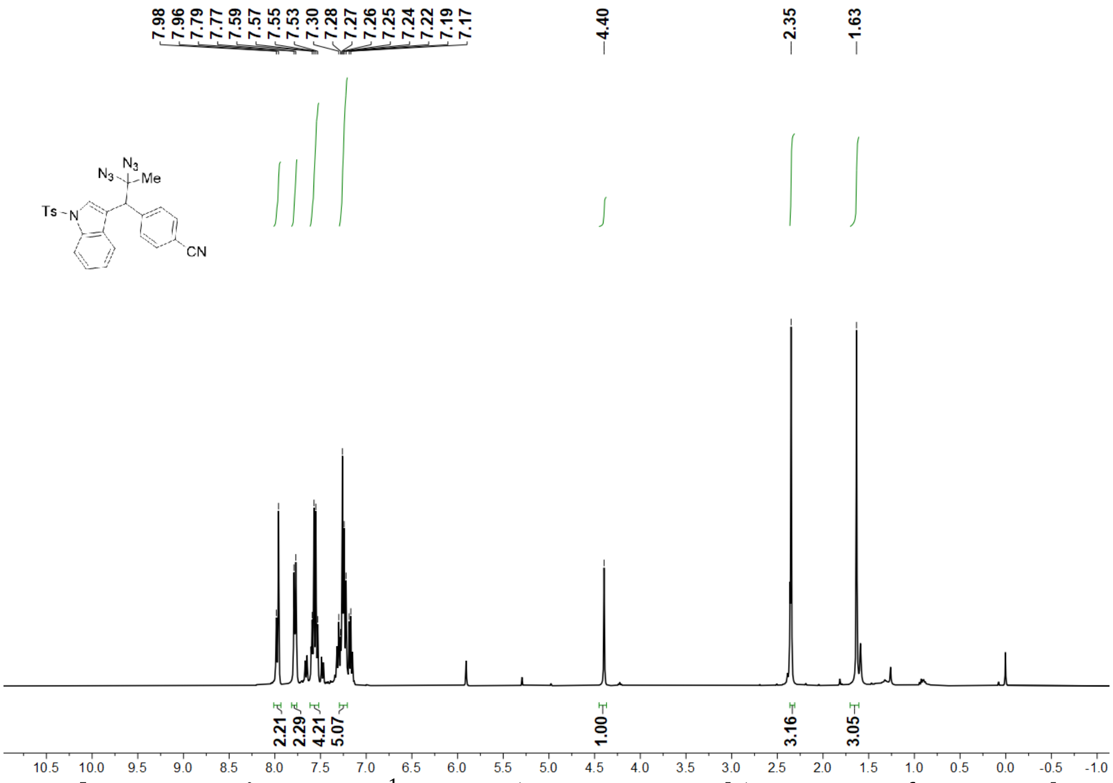


**Supplementary Figure 130.** $^{1}H$ NMR (400 MHz, $CDCl_3$) spectrum of compound **2w**

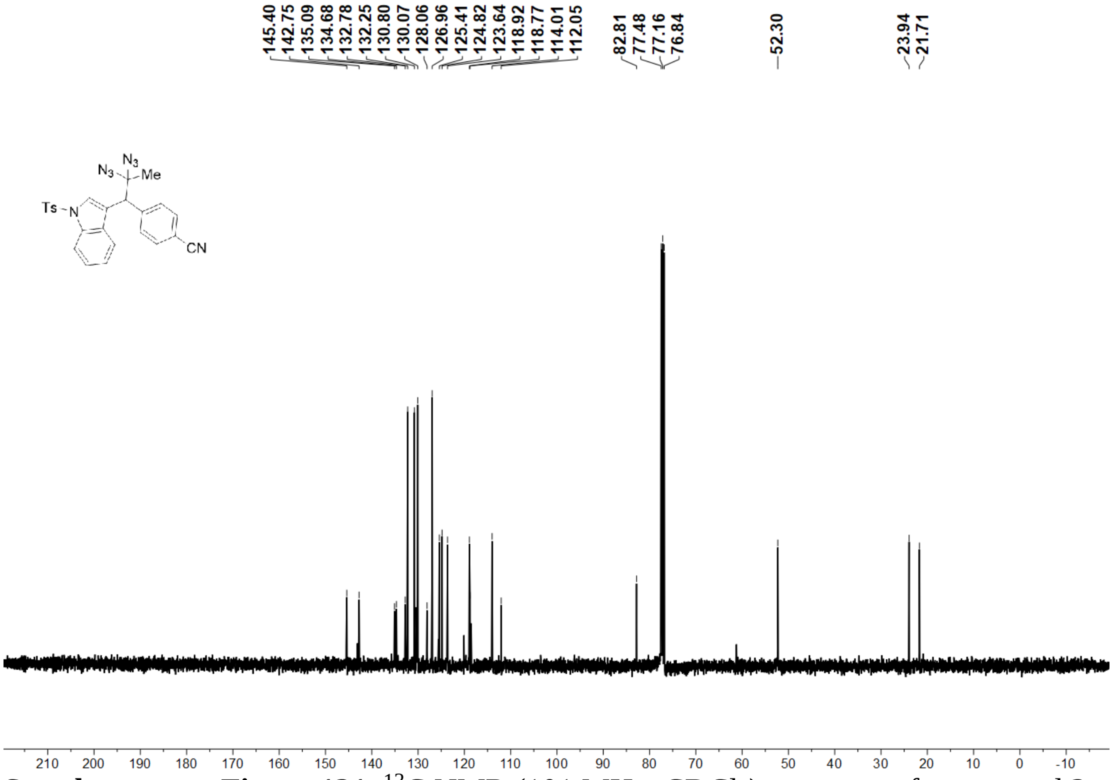


**Supplementary Figure 131.** $^{13}C$ NMR (101 MHz, $CDCl_3$) spectrum of compound **2w**

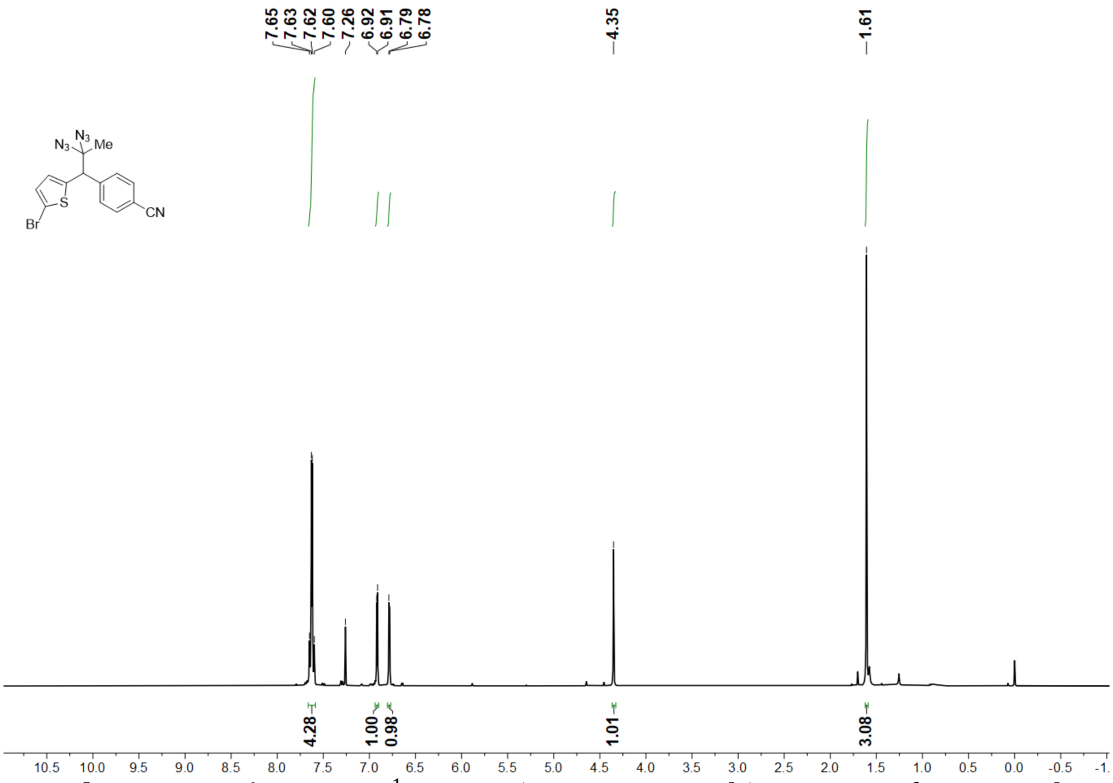


**Supplementary Figure 132.** $^{1}H$ NMR (400 MHz, $CDCl_3$) spectrum of compound **2x**

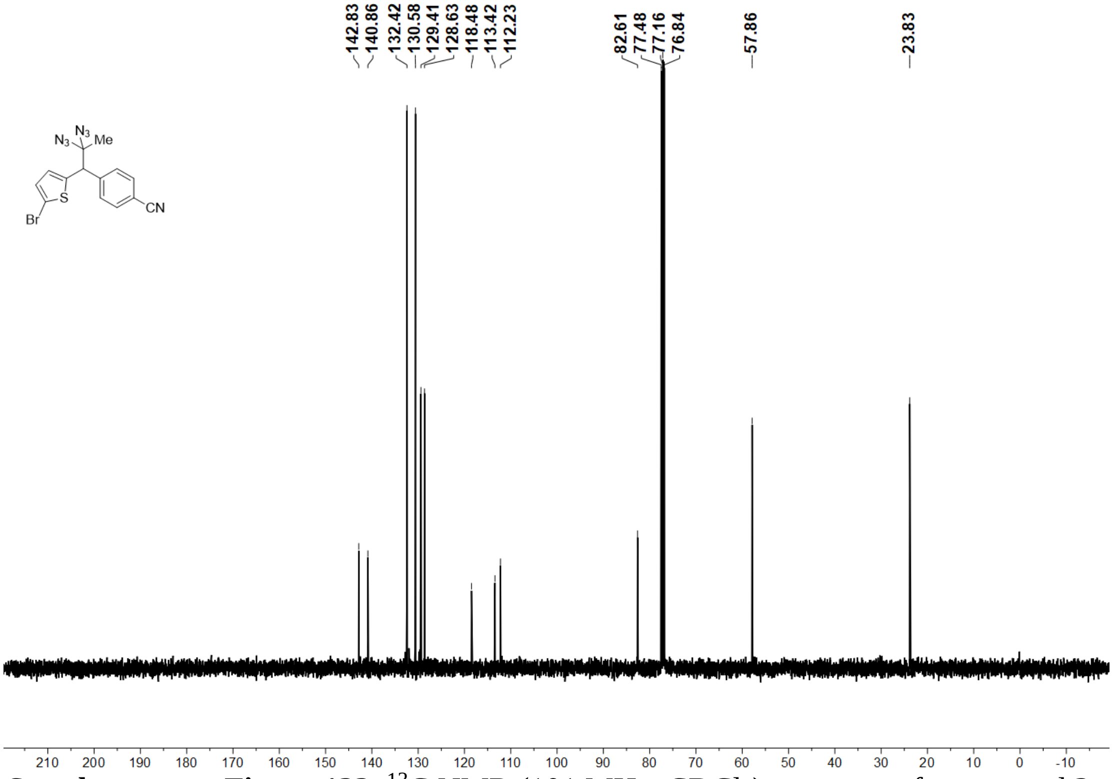


**Supplementary Figure 133.** $^{13}C$ NMR (101 MHz, $CDCl_3$) spectrum of compound **2x**

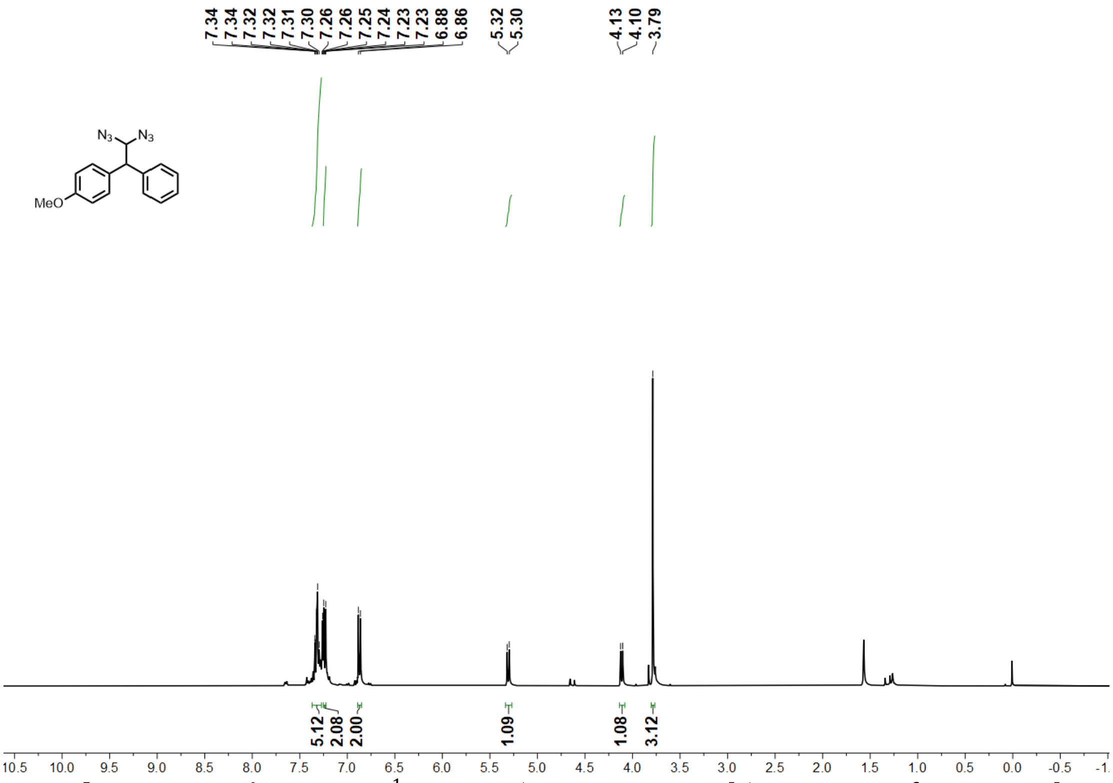


**Supplementary Figure 134.** $^{1}$H NMR (400 MHz, $CDCl_3$) spectrum of compound **2y**

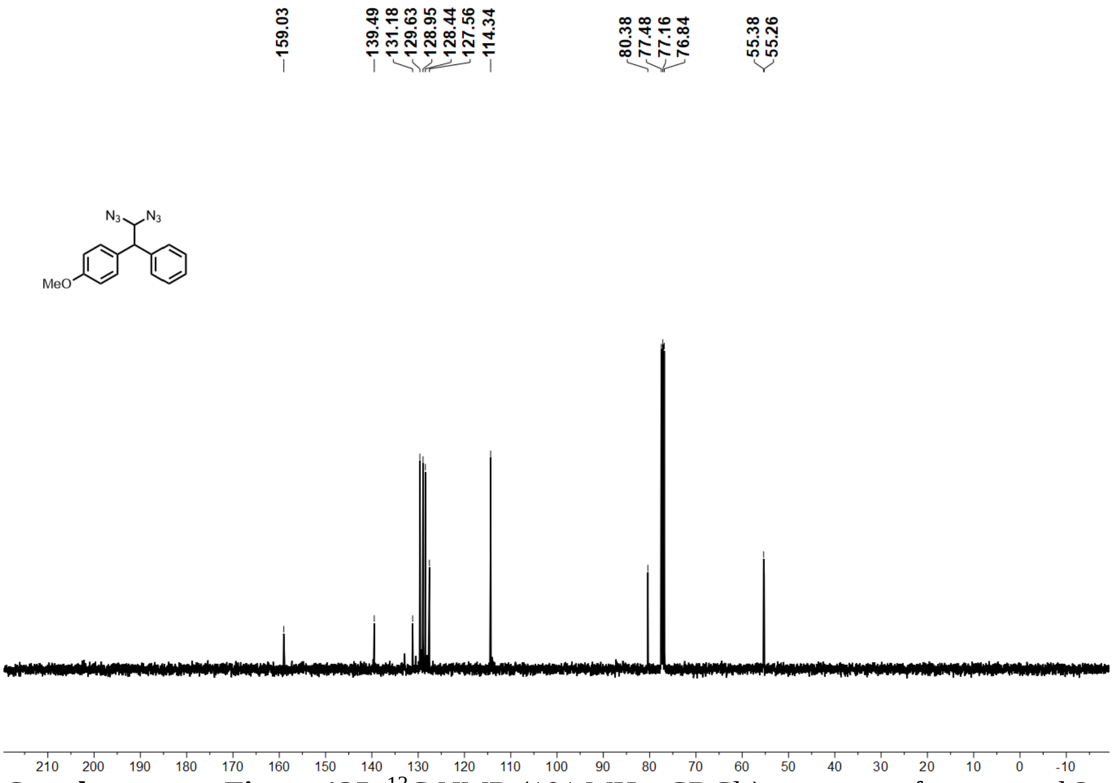


**Supplementary Figure 135.** $^{13}$C NMR (101 MHz, $CDCl_3$) spectrum of compound **2y**

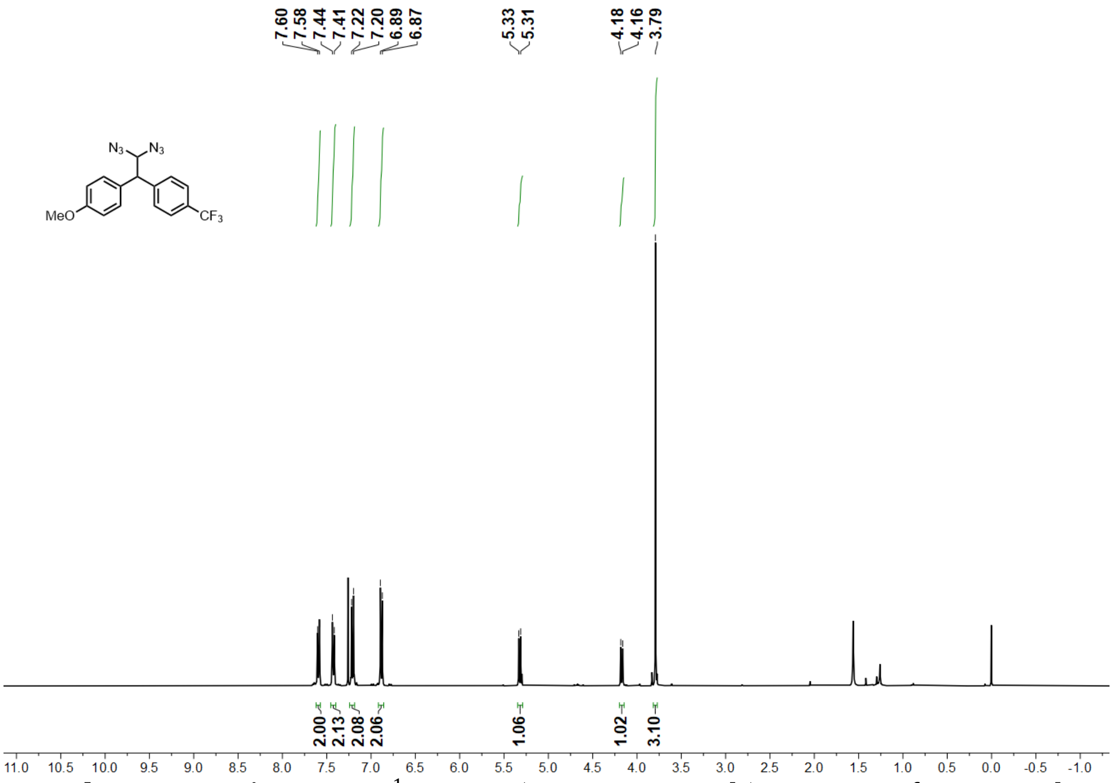


**Supplementary Figure 136.** $^{1}H$ NMR (400 MHz, $CDCl_3$) spectrum of compound **2z**

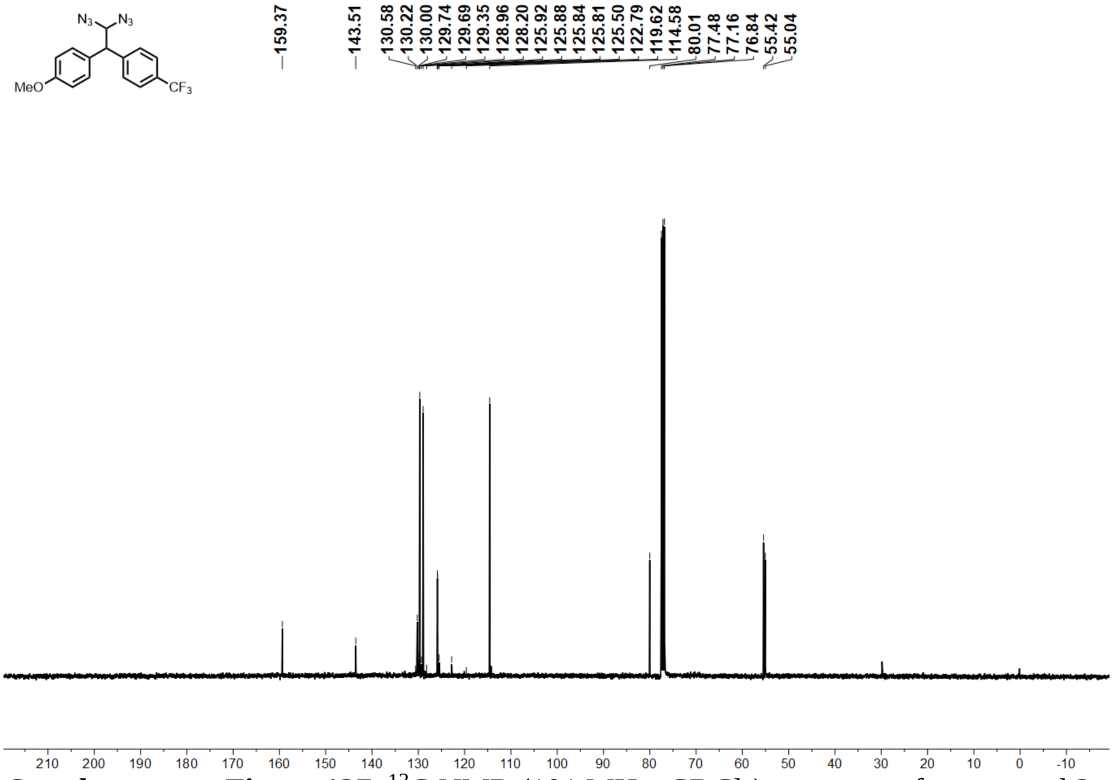


**Supplementary Figure 137.** $^{13}C$ NMR (101 MHz, $CDCl_3$) spectrum of compound **2z**

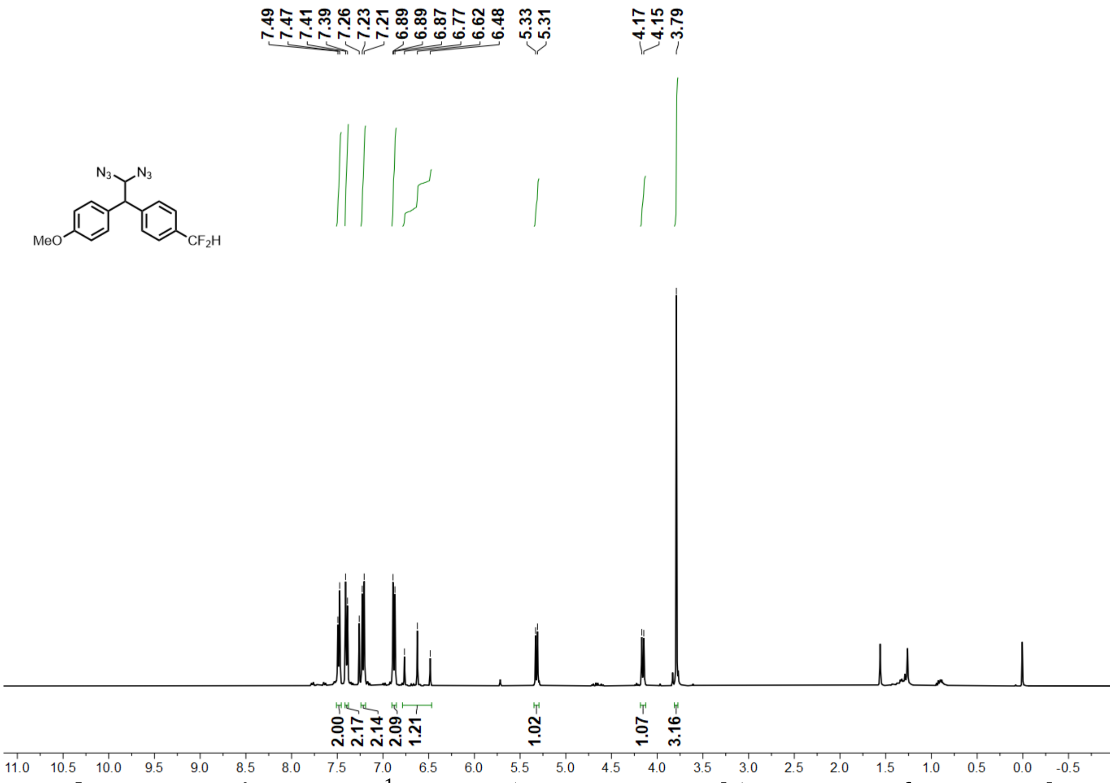


**Supplementary Figure 138.** $^{1}$H NMR (400 MHz, $CDCl_3$) spectrum of compound **2aa**

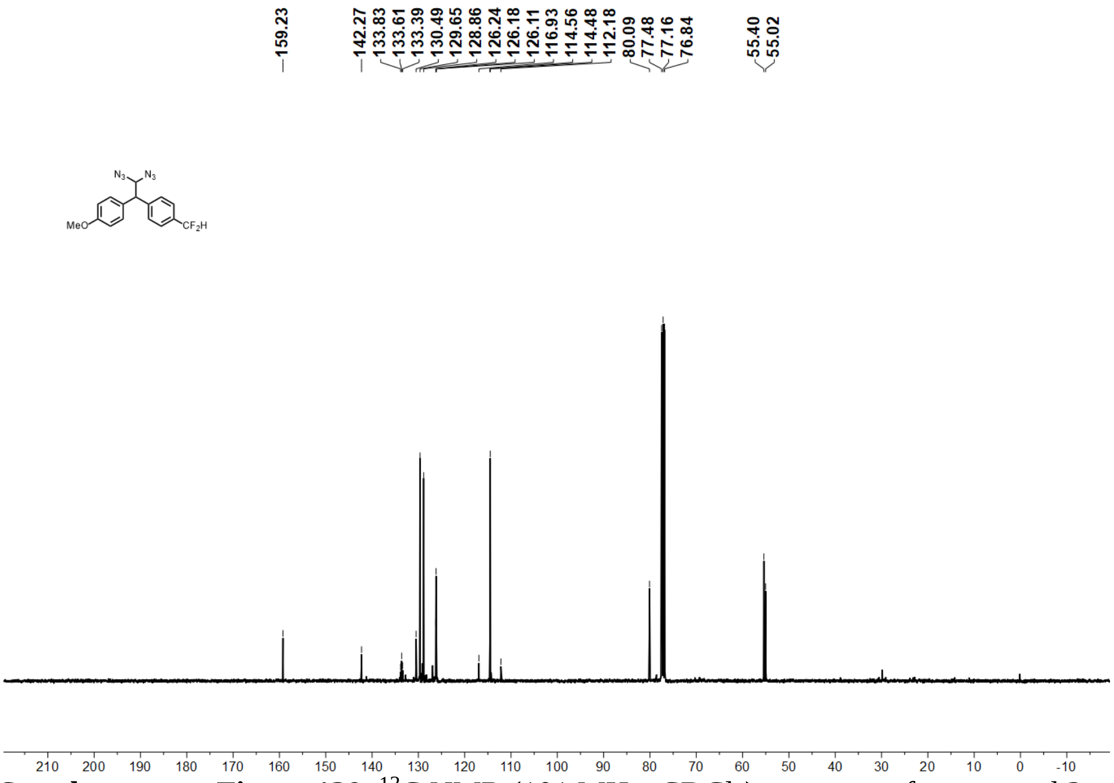


**Supplementary Figure 139.** $^{13}$C NMR (101 MHz, $CDCl_3$) spectrum of compound **2aa**

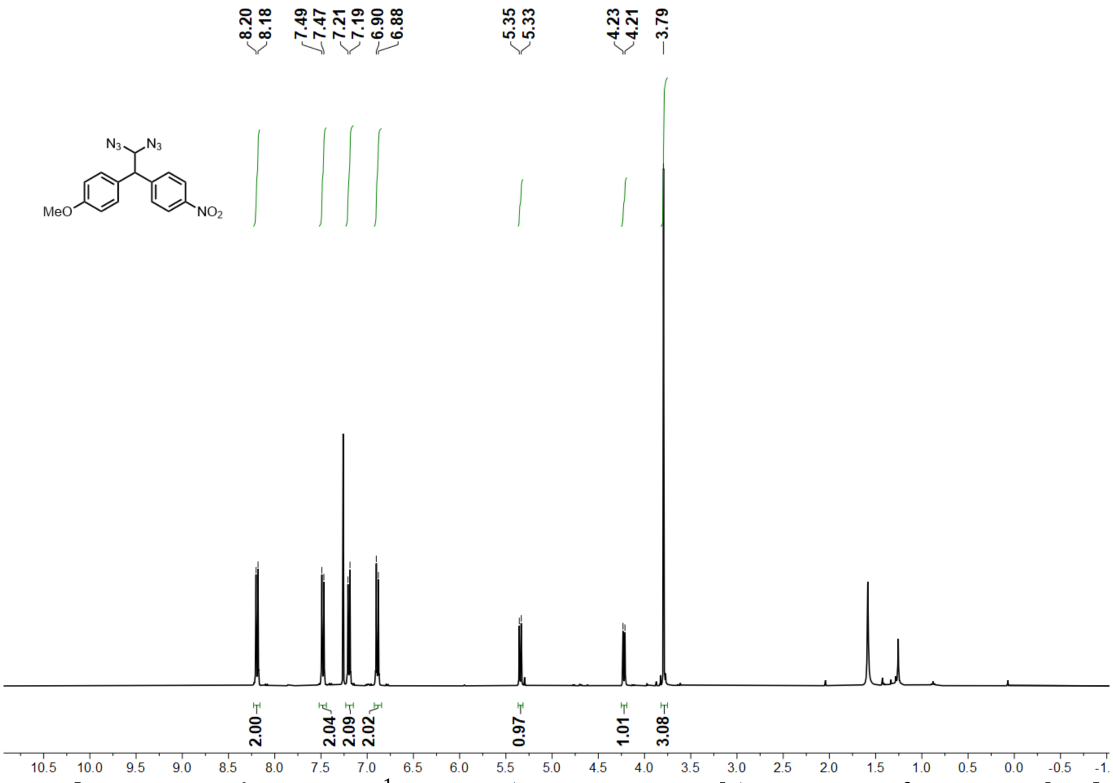


**Supplementary Figure 140.** $^{1}H$ NMR (400 MHz, $CDCl_3$) spectrum of compound **2ab**

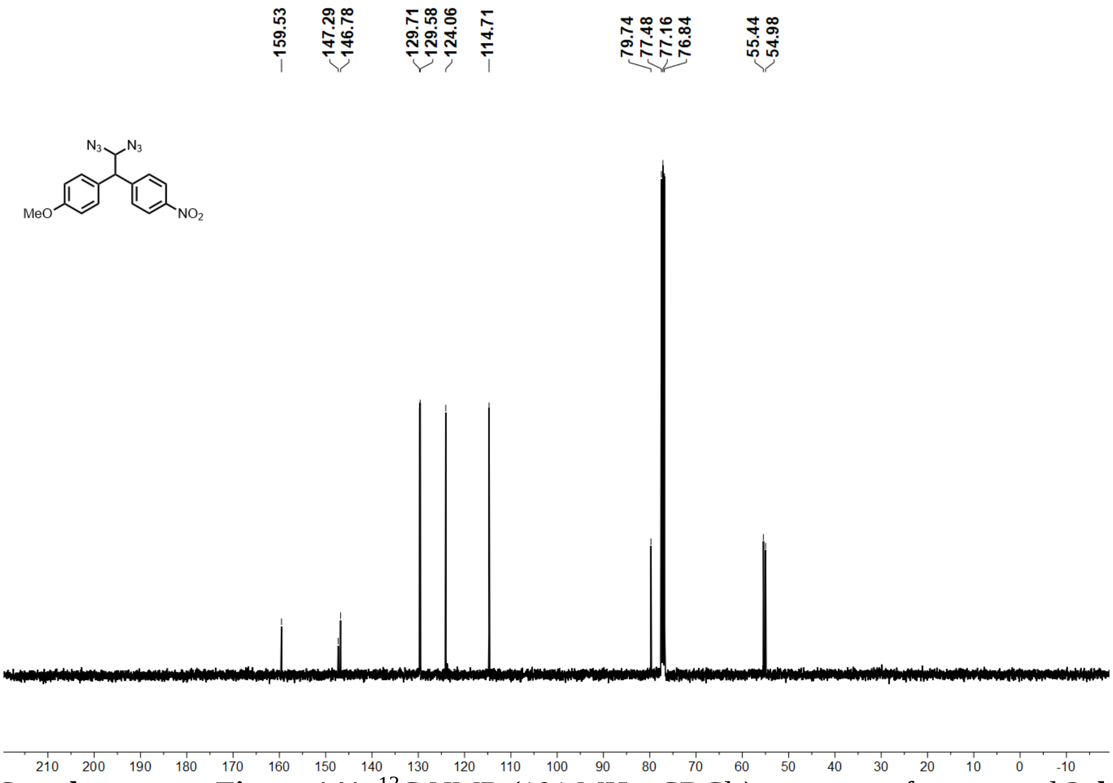


**Supplementary Figure 141.** $^{13}C$ NMR (101 MHz, $CDCl_3$) spectrum of compound **2ab**

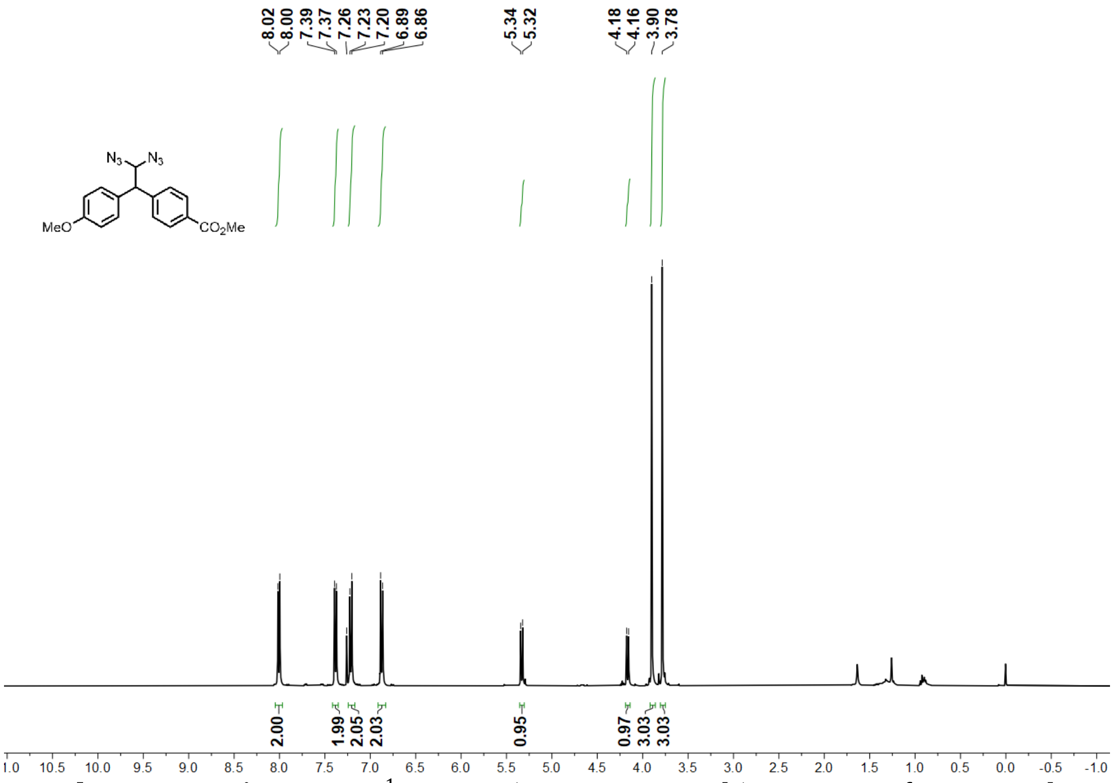


**Supplementary Figure 142.** $^{1}H$ NMR (400 MHz, $CDCl_3$) spectrum of compound **2ac**

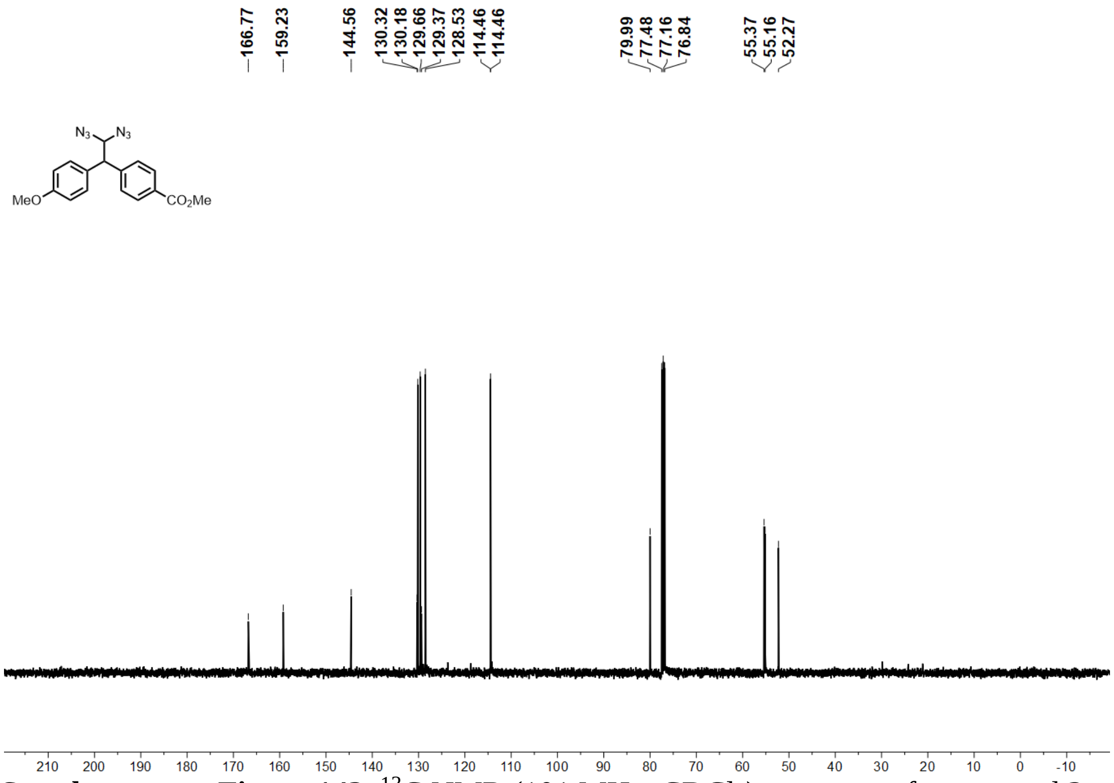


**Supplementary Figure 143.** $^{13}C$ NMR (101 MHz, $CDCl_3$) spectrum of compound **2ac**

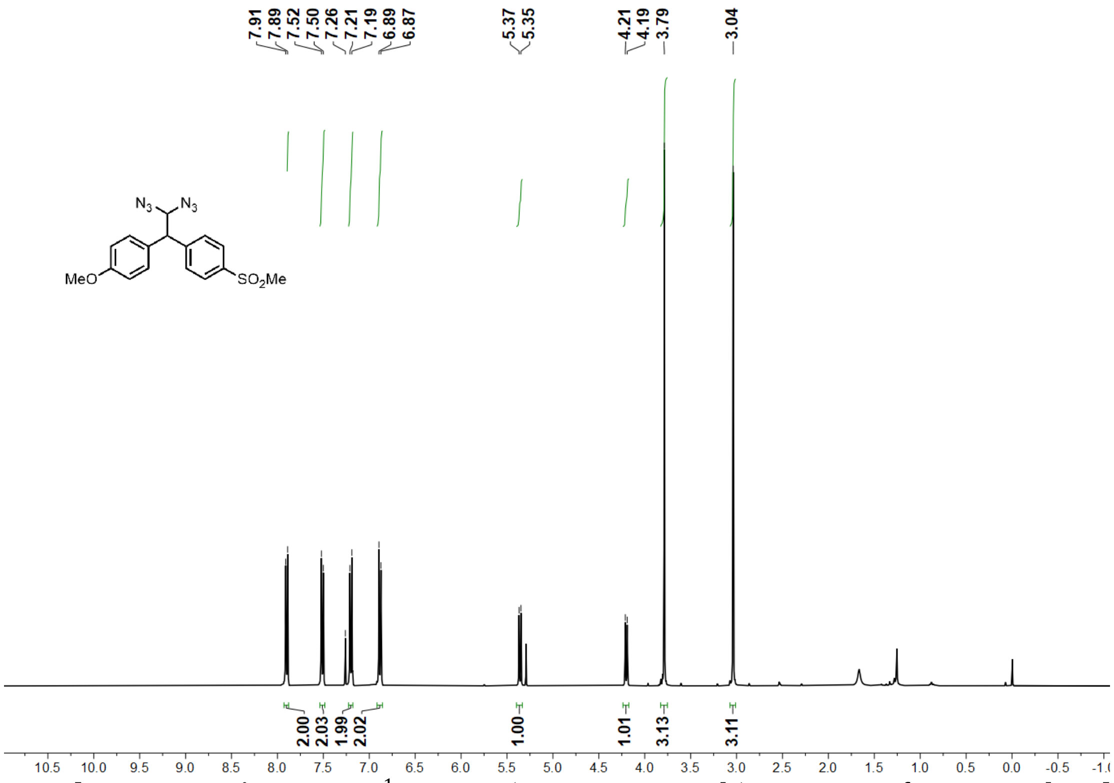


**Supplementary Figure 144.** $^{1}$H NMR (400 MHz, $CDCl_3$) spectrum of compound **2ad**

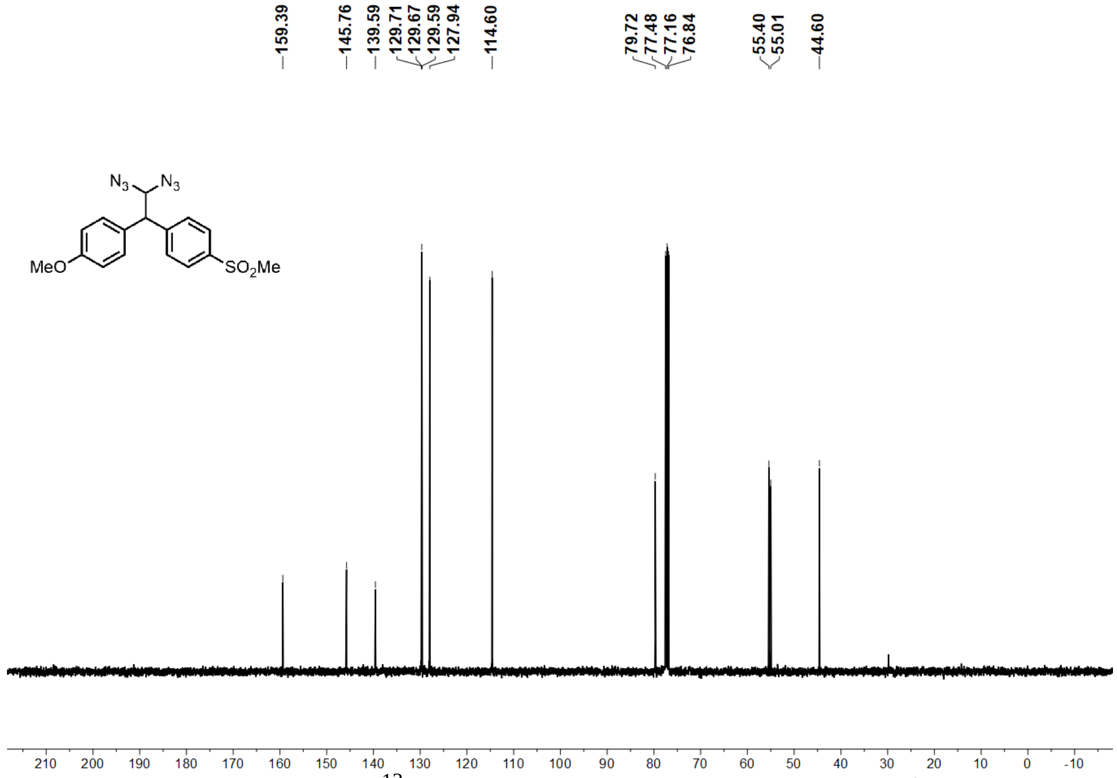


**Supplementary Figure 145.** $^{13}$C NMR (101 MHz, $CDCl_3$) spectrum of compound **2ad**

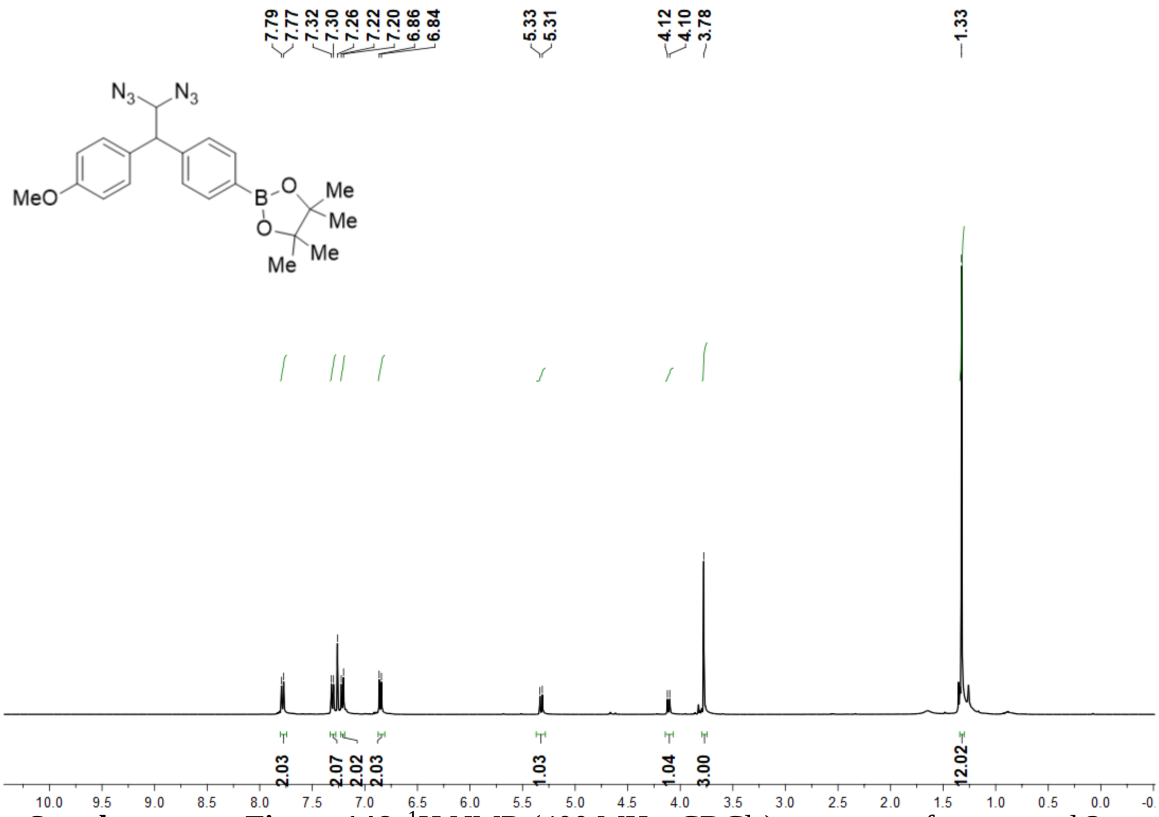


**Supplementary Figure 146.** $^{1}$H NMR (400 MHz, $CDCl_3$) spectrum of compound **2ae**

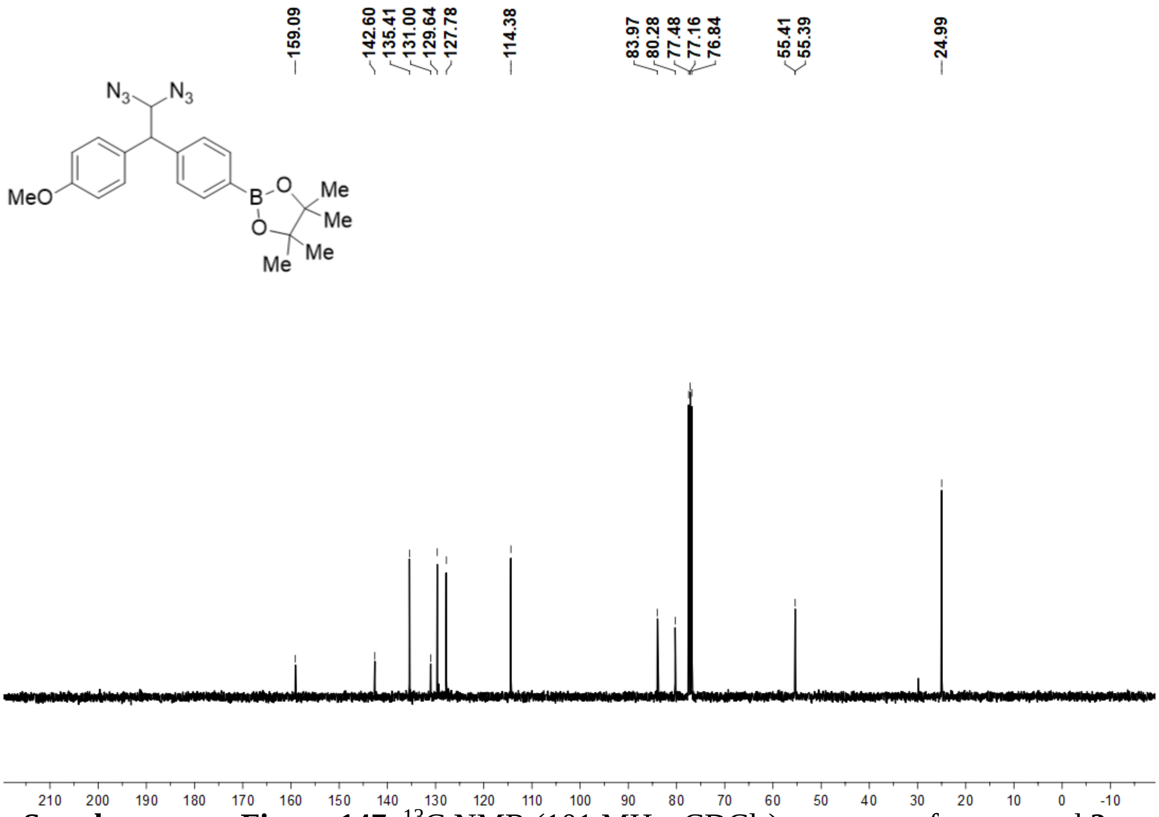


**Supplementary Figure 147.** $^{13}$C NMR (101 MHz, $CDCl_3$) spectrum of compound **2ae**

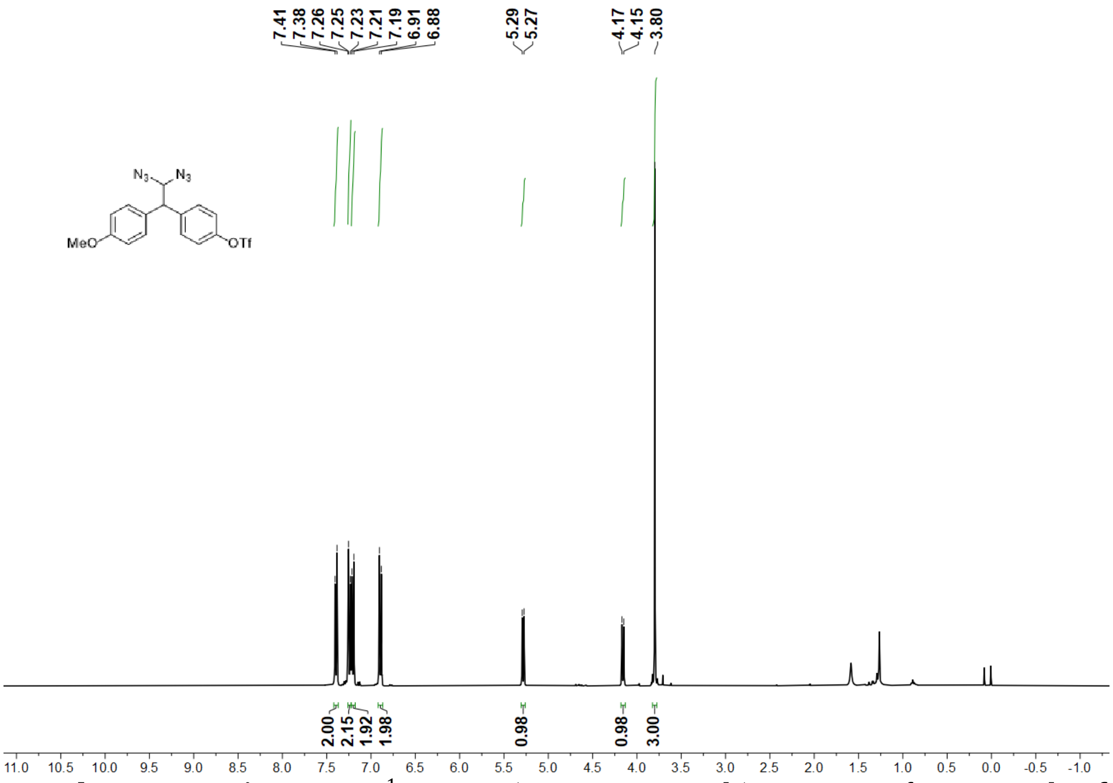

**Supplementary Figure 148.** $^{1}$H NMR (400 MHz, $CDCl_3$) spectrum of compound **2af**

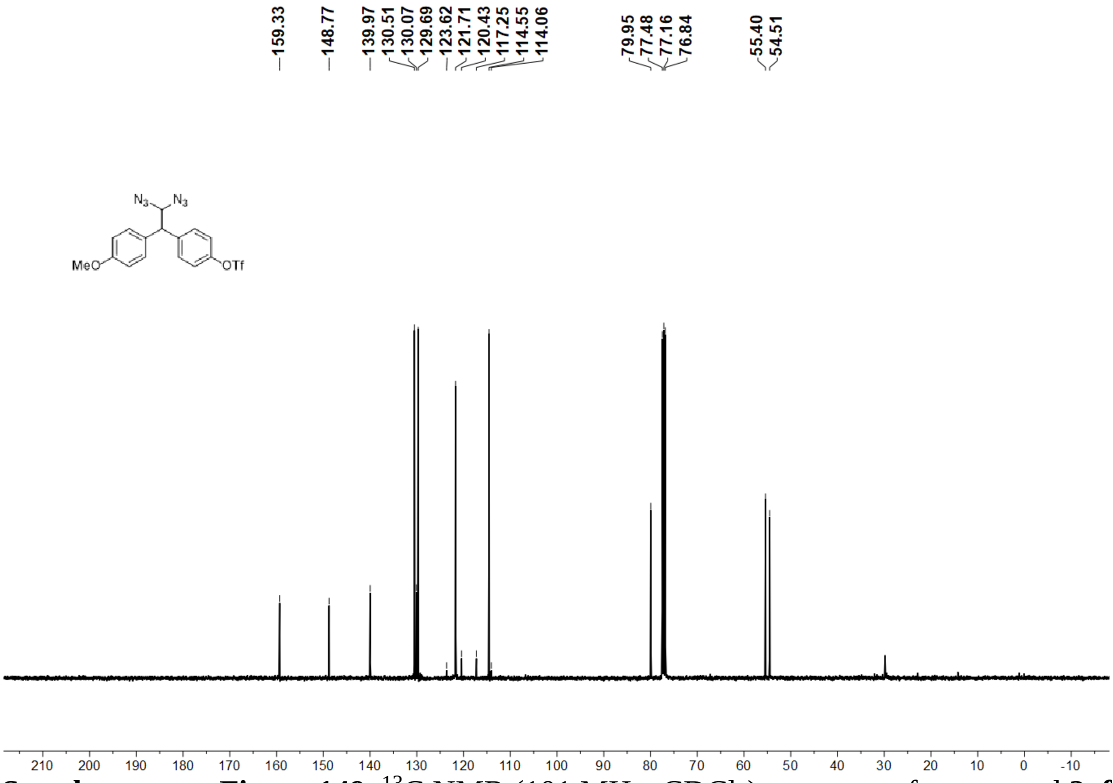

**Supplementary Figure 149.** $^{13}$C NMR (101 MHz, $CDCl_3$) spectrum of compound **2af**

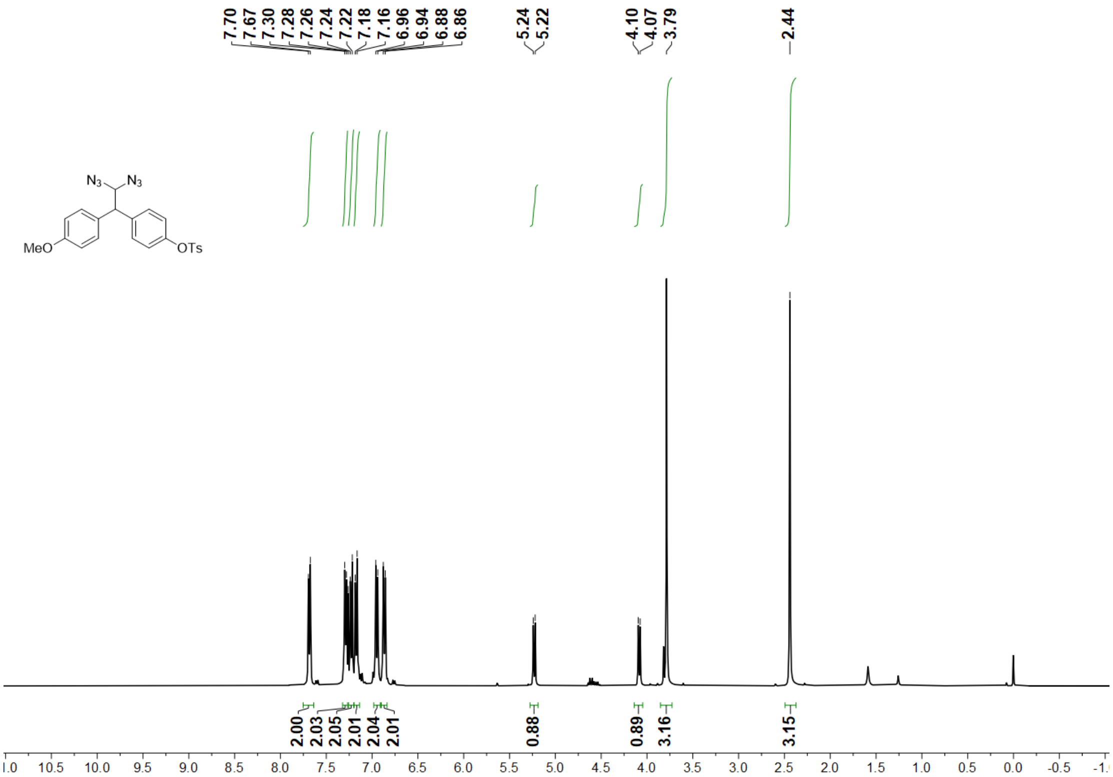


**Supplementary Figure 150.** $^{1}$H NMR (400 MHz, $CDCl_3$) spectrum of compound **2ag**

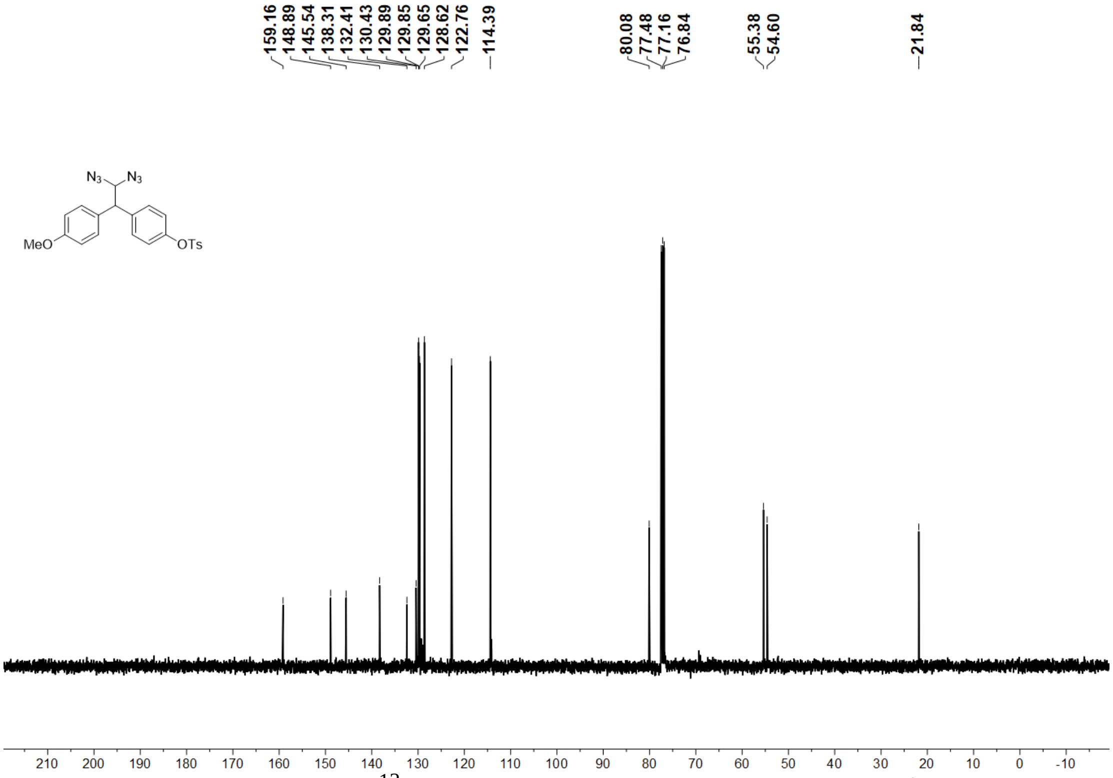


**Supplementary Figure 151.** $^{13}$C NMR (101 MHz, $CDCl_3$) spectrum of compound **2ag**

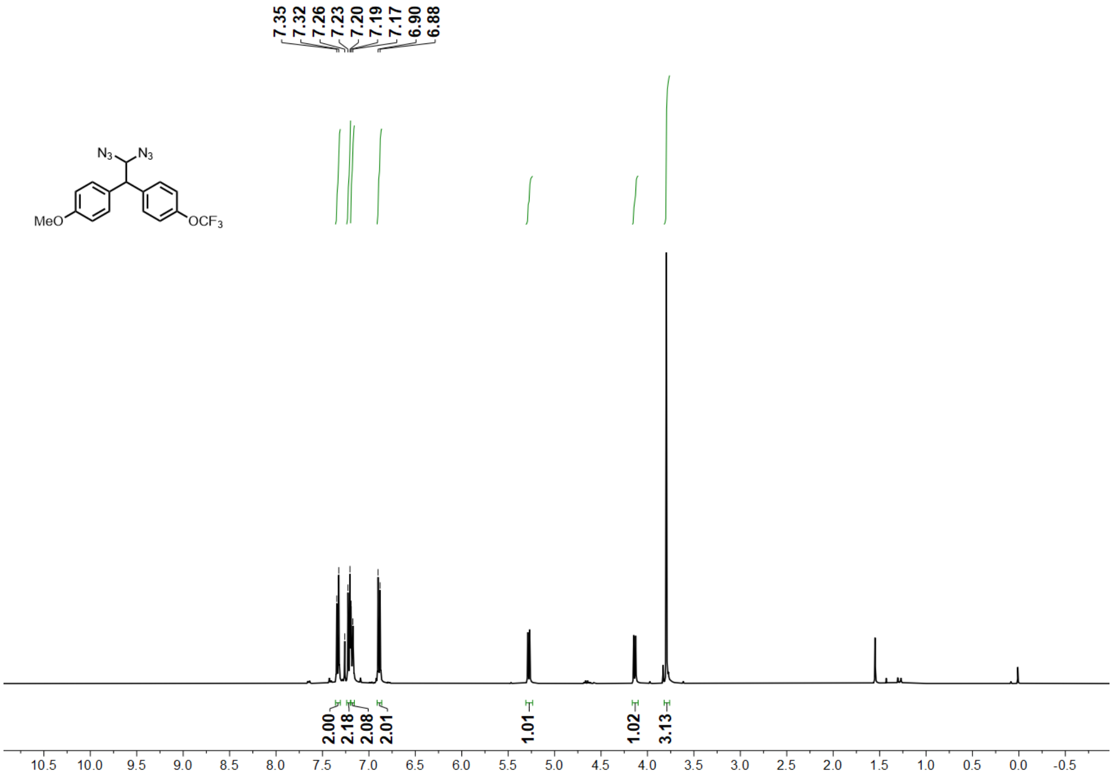


**Supplementary Figure 152.** $^{1}H$ NMR (400 MHz, $CDCl_3$) spectrum of compound **2ah**

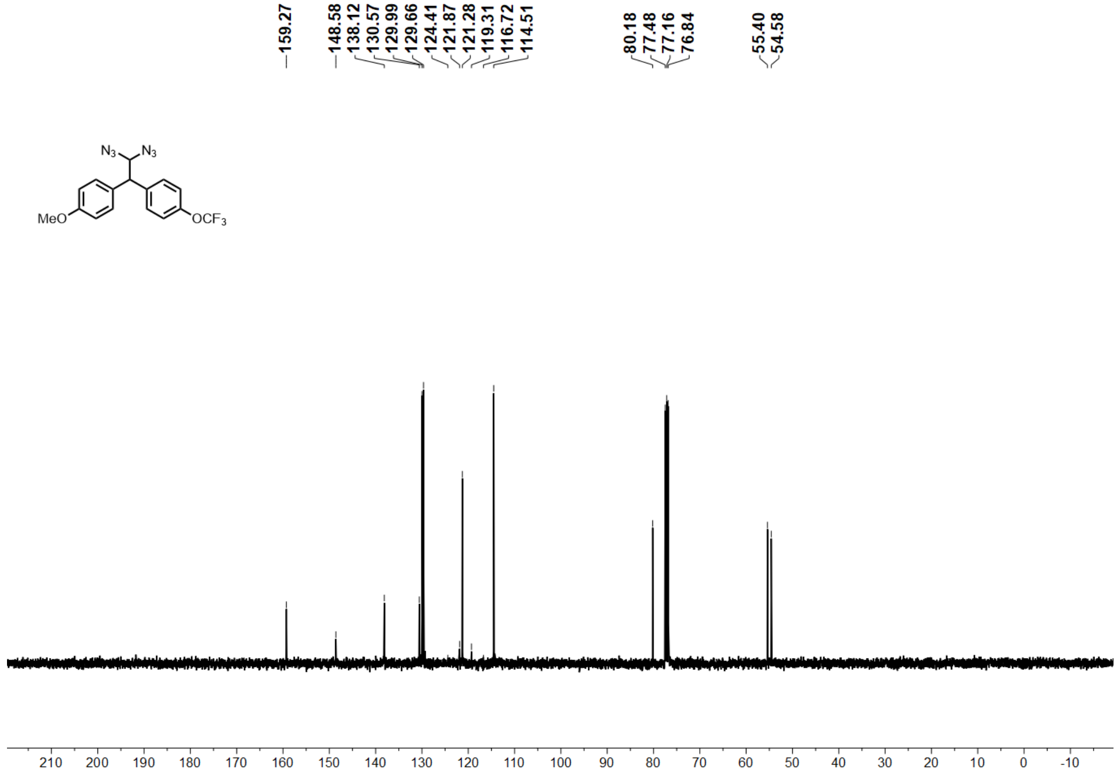


**Supplementary Figure 153.** $^{13}C$ NMR (101 MHz, $CDCl_3$) spectrum of compound **2ah**

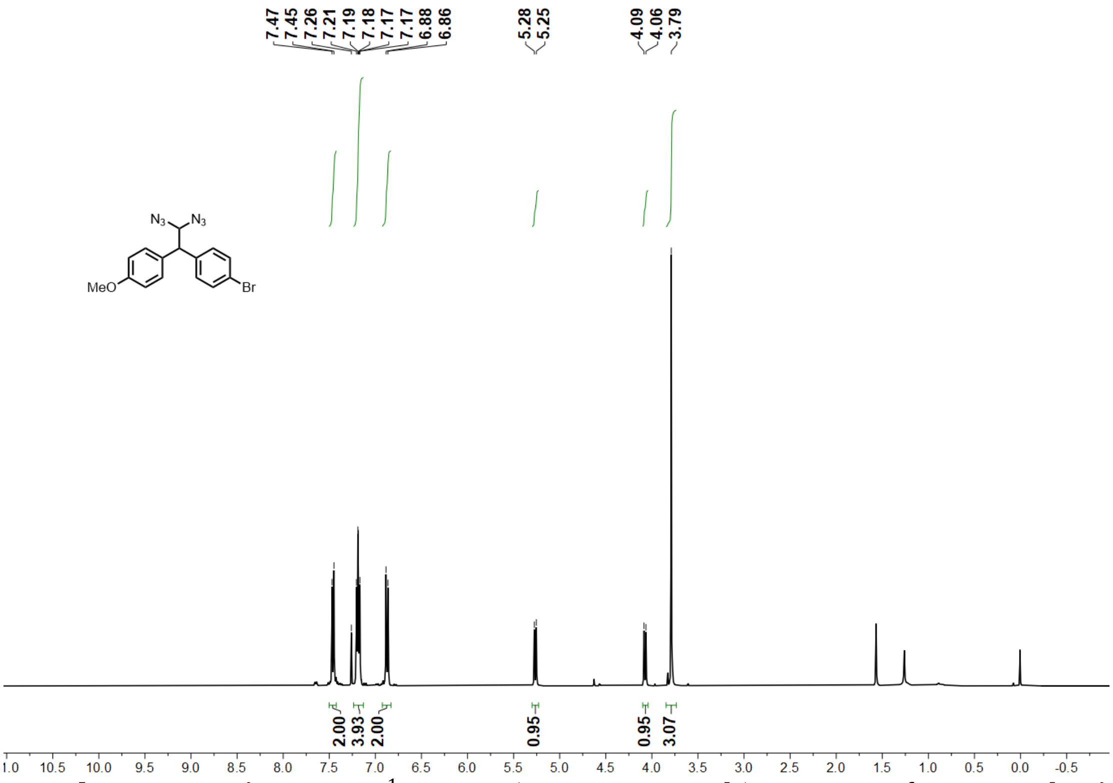


**Supplementary Figure 154.** $^{1}$H NMR (400 MHz, $CDCl_3$) spectrum of compound **2ai**

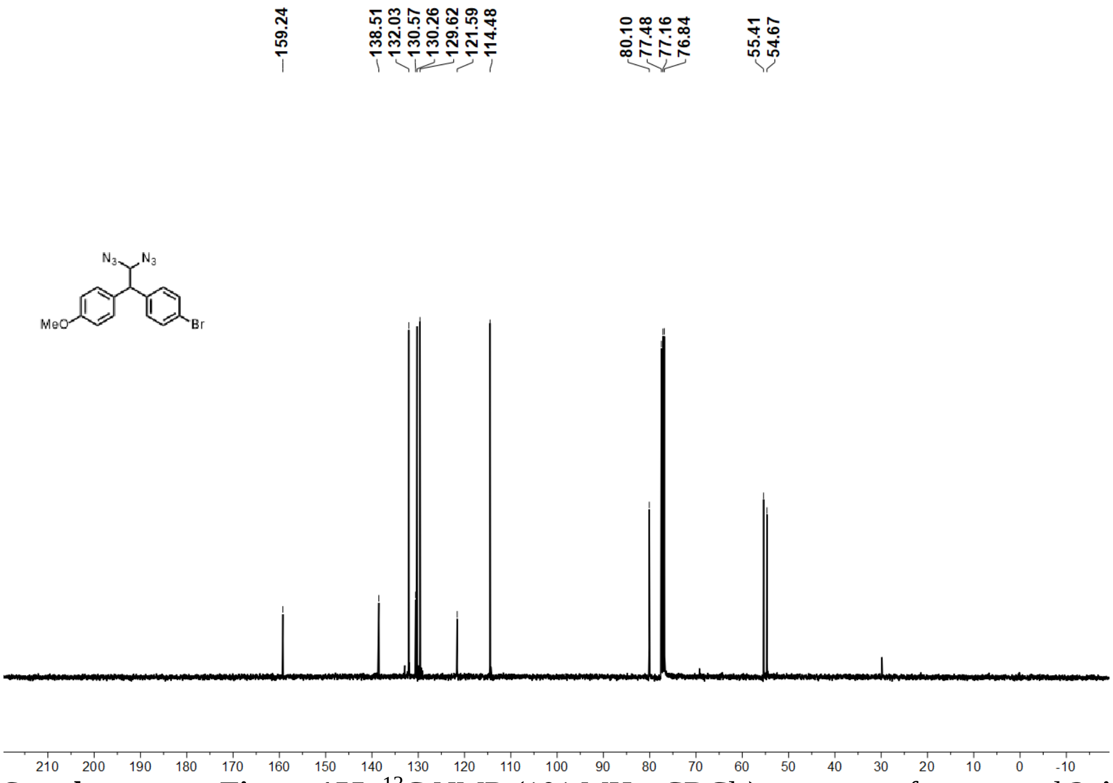


**Supplementary Figure 155.** $^{13}$C NMR (101 MHz, $CDCl_3$) spectrum of compound **2ai**

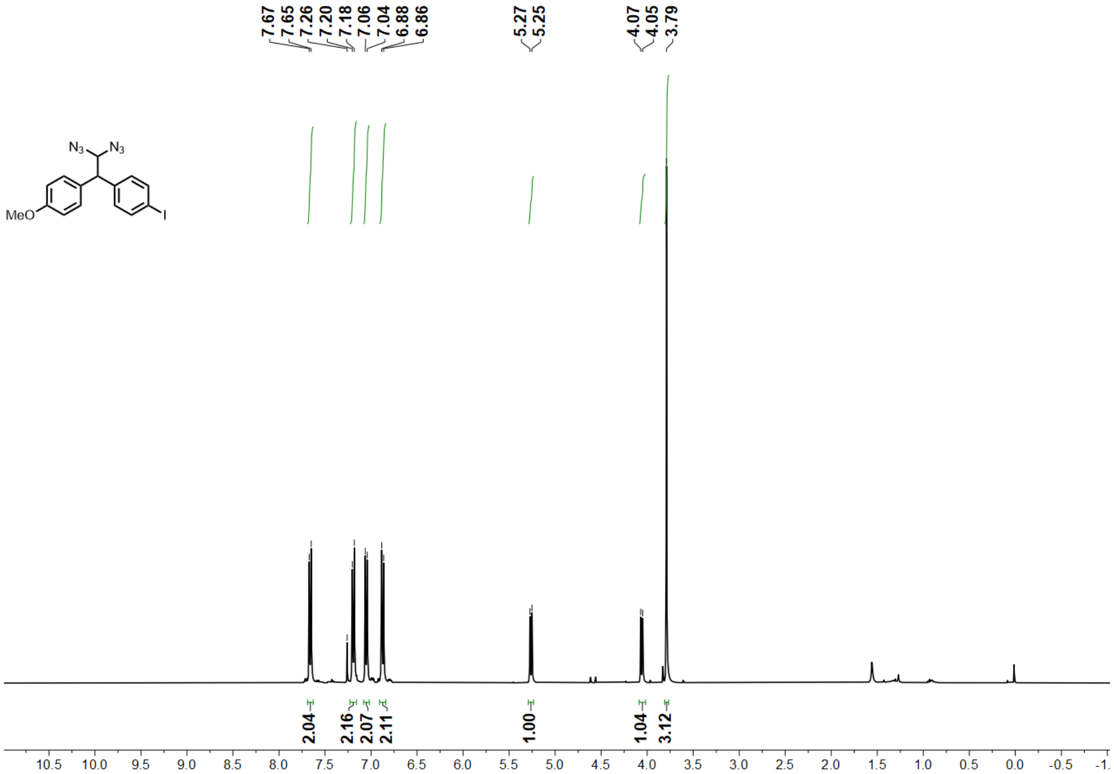


**Supplementary Figure 156.** $^{1}H$ NMR (400 MHz, $CDCl_3$) spectrum of compound **2aj**

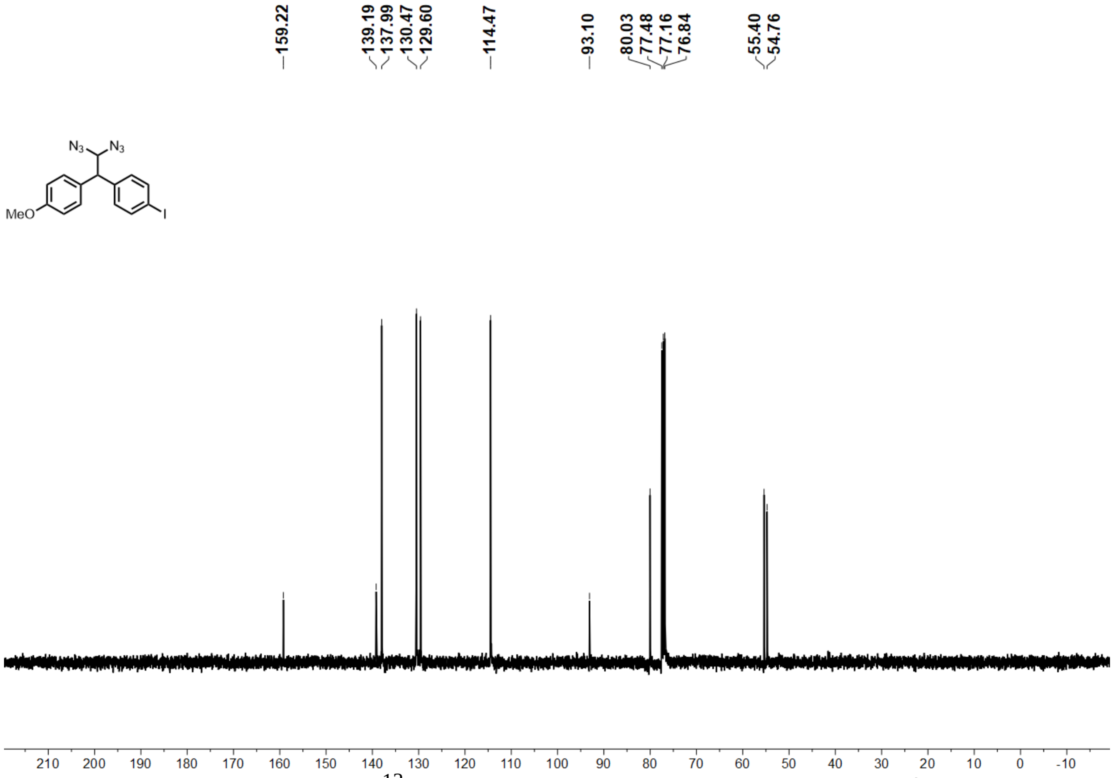


**Supplementary Figure 157.** $^{13}C$ NMR (101 MHz, $CDCl_3$) spectrum of compound **2aj**

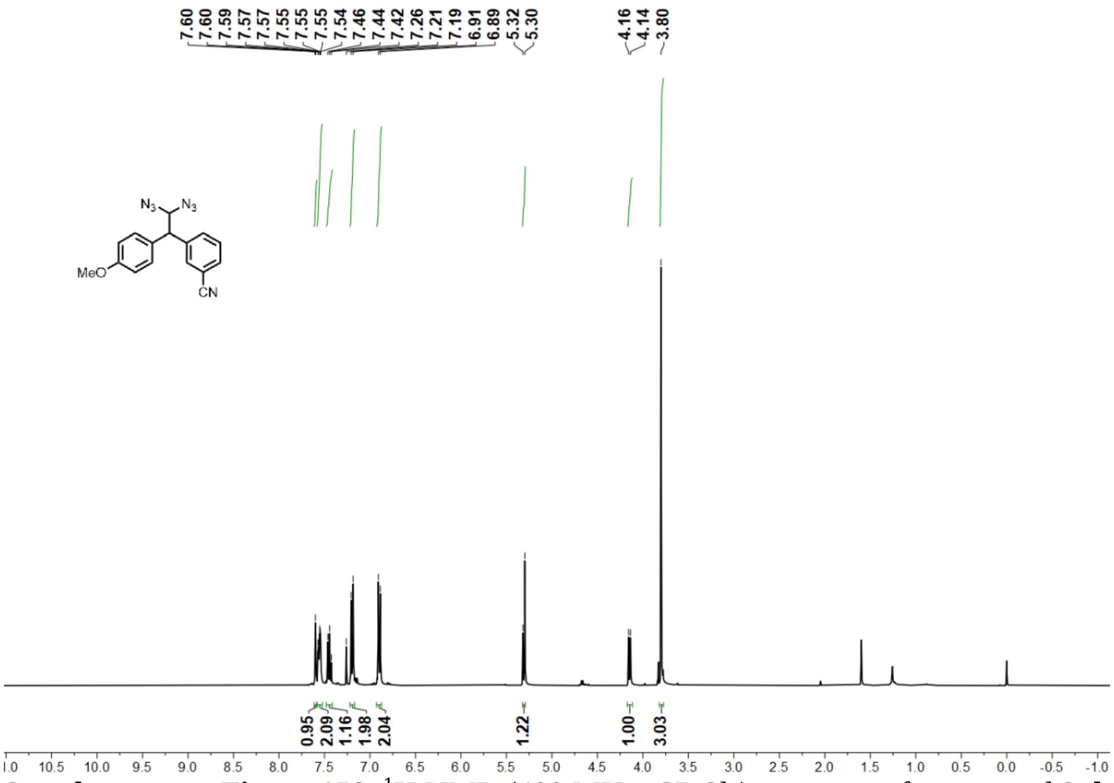

**Supplementary Figure 158.** $^{1}H$ NMR (400 MHz, $CDCl_3$) spectrum of compound **2ak**

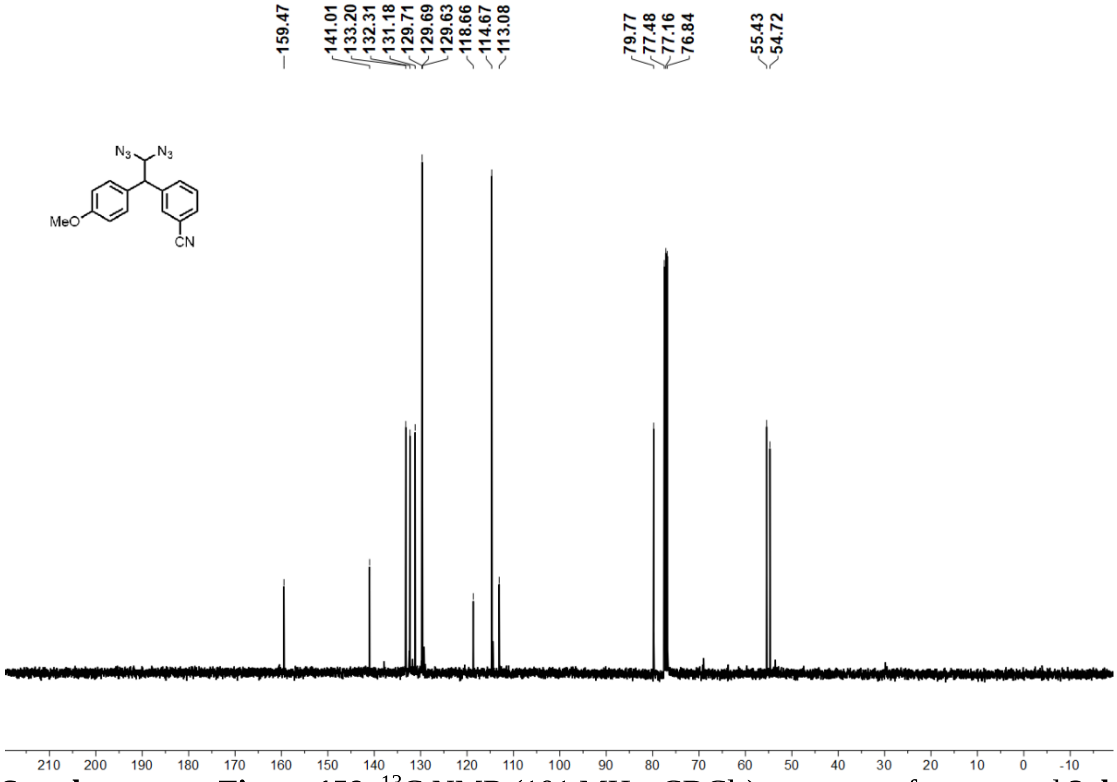

**Supplementary Figure 159.** $^{13}C$ NMR (101 MHz, $CDCl_3$) spectrum of compound **2ak**

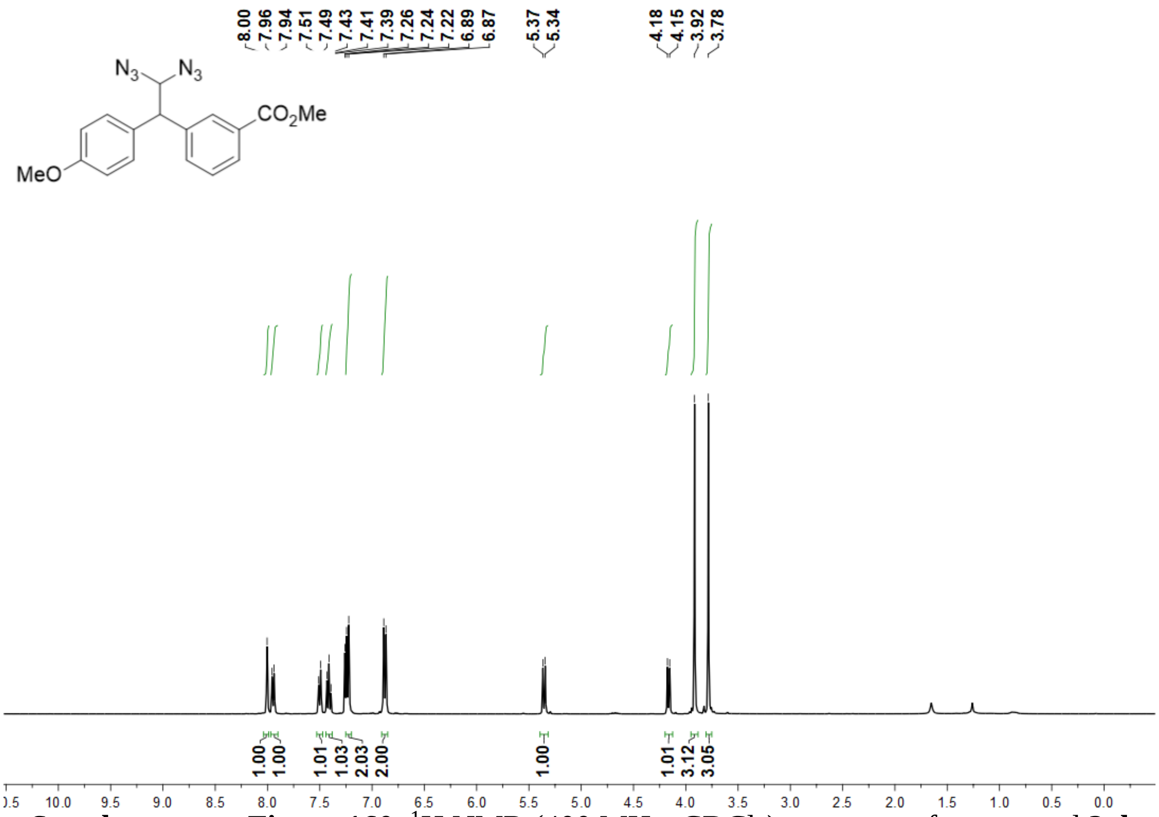

**Supplementary Figure 160.** $^{1}$H NMR (400 MHz, $CDCl_3$) spectrum of compound **2al**

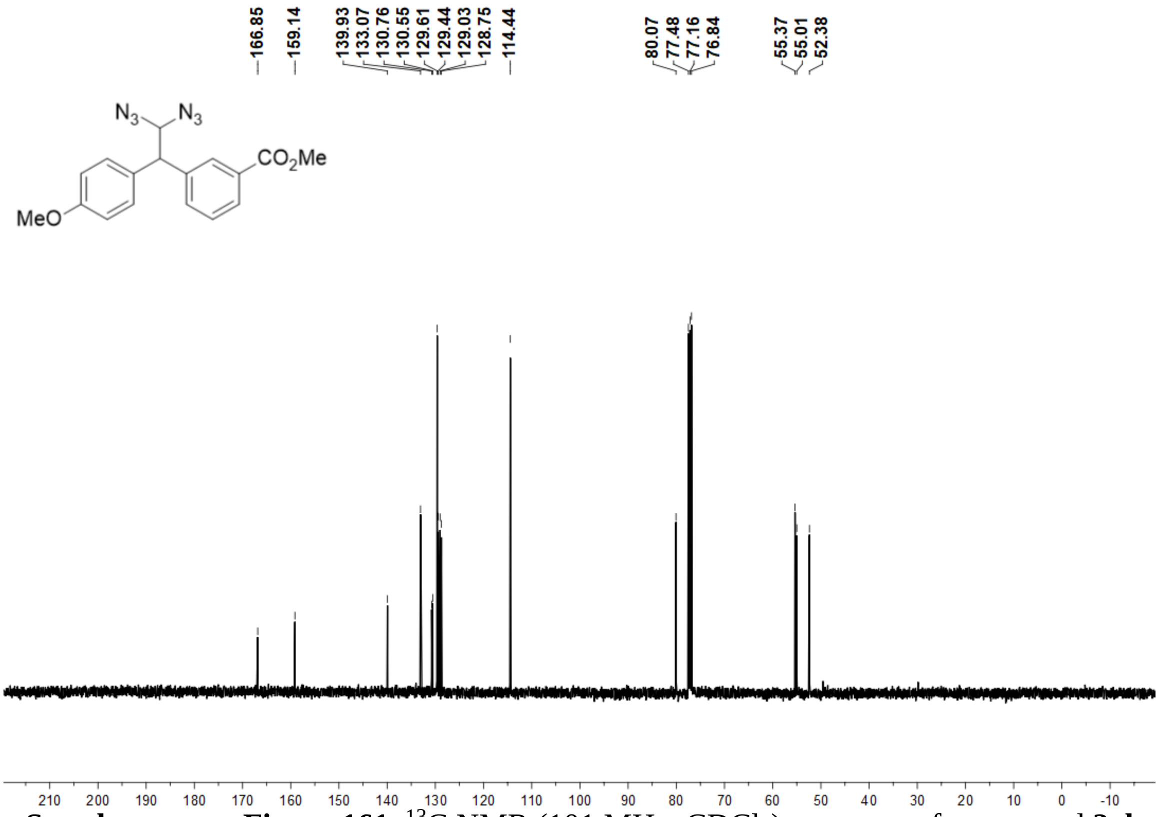

**Supplementary Figure 161.** $^{13}$C NMR (101 MHz, $CDCl_3$) spectrum of compound **2al**

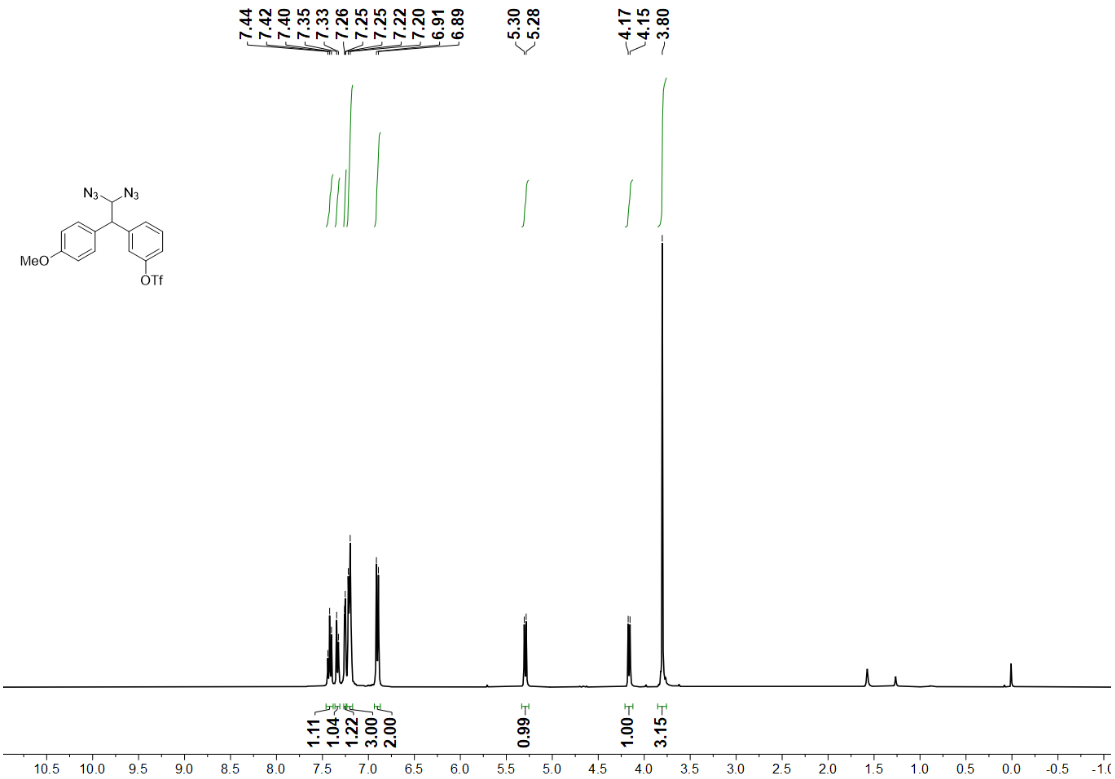

**Supplementary Figure 162.** $^{1}H$ NMR (400 MHz, $CDCl_3$) spectrum of compound **2am**

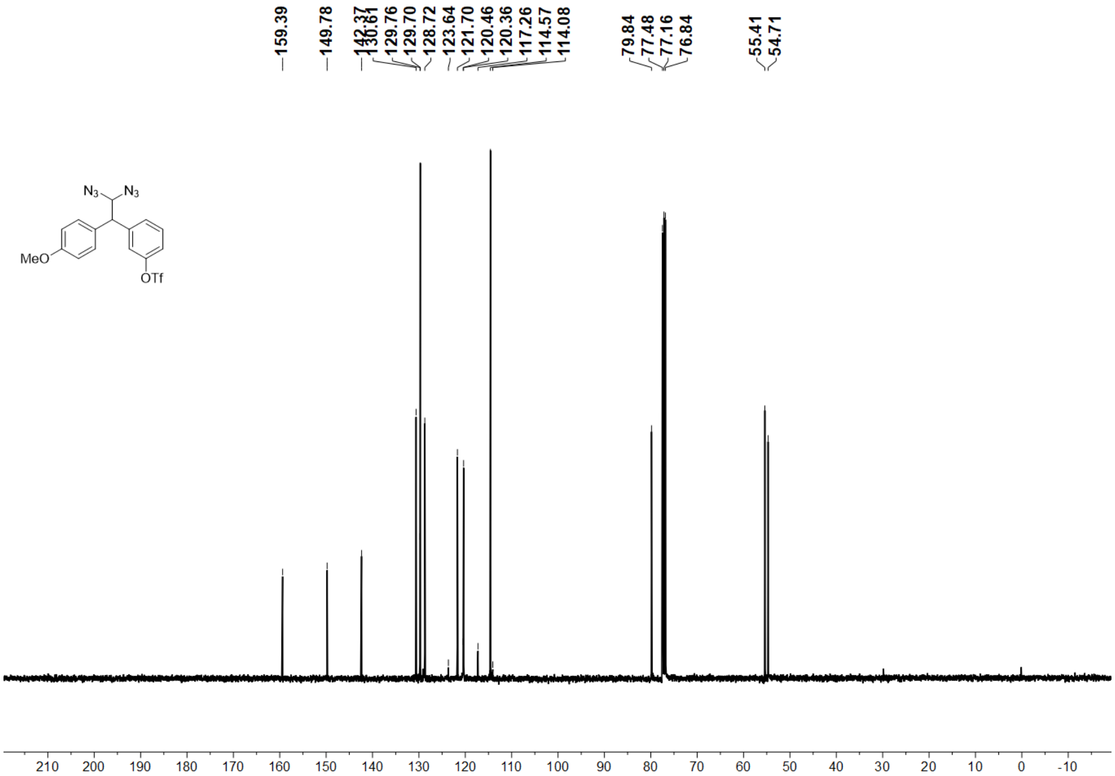

**Supplementary Figure 163.** $^{13}C$ NMR (101 MHz, $CDCl_3$) spectrum of compound **2am**

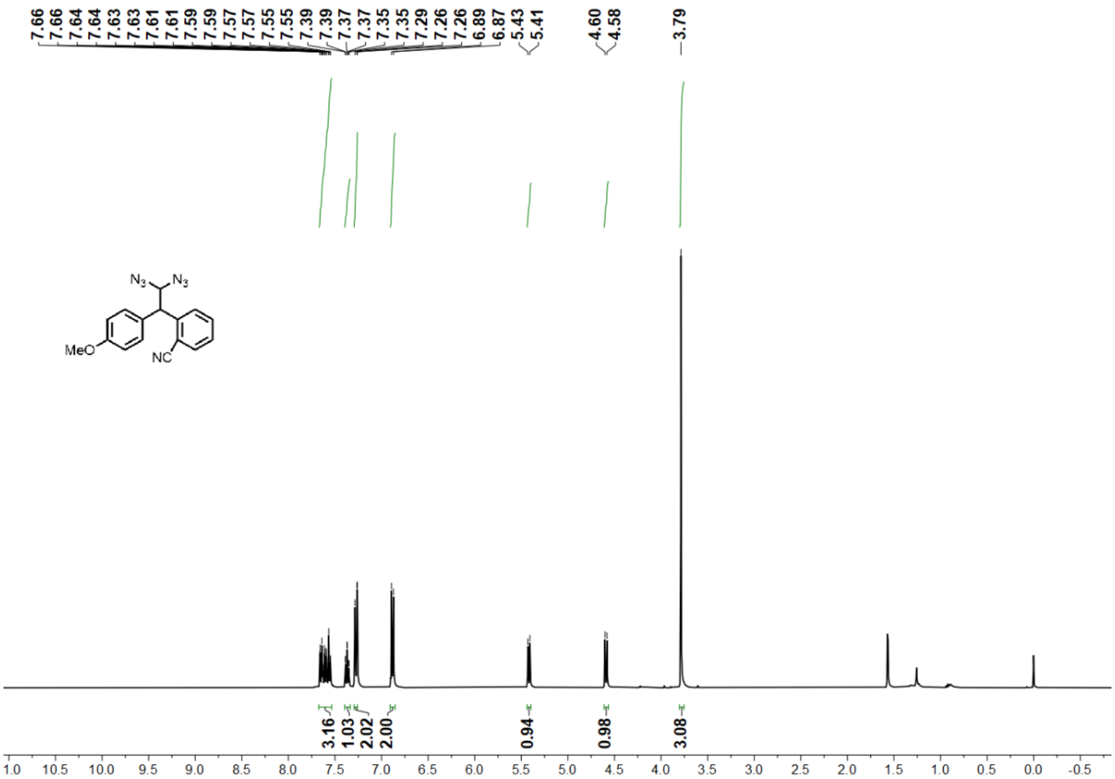


**Supplementary Figure 164.** $^{1}$H NMR (400 MHz, $CDCl_3$) spectrum of compound **2an**

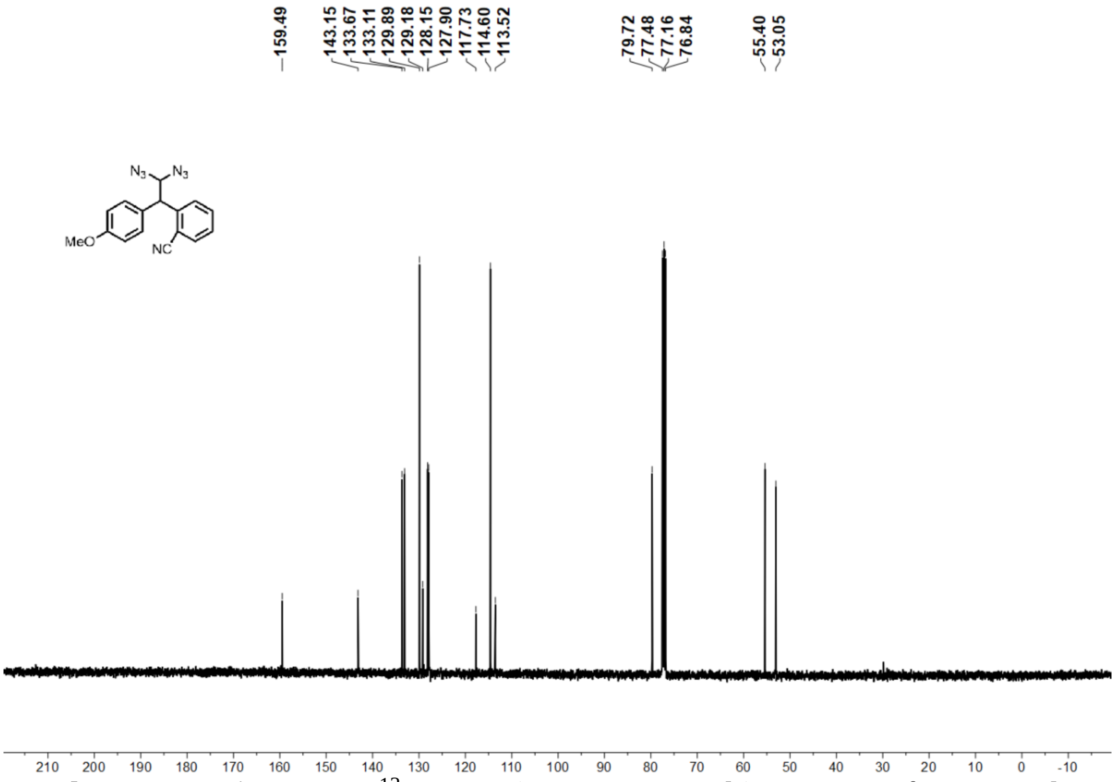


**Supplementary Figure 165.** $^{13}$C NMR (101 MHz, $CDCl_3$) spectrum of compound **2an**

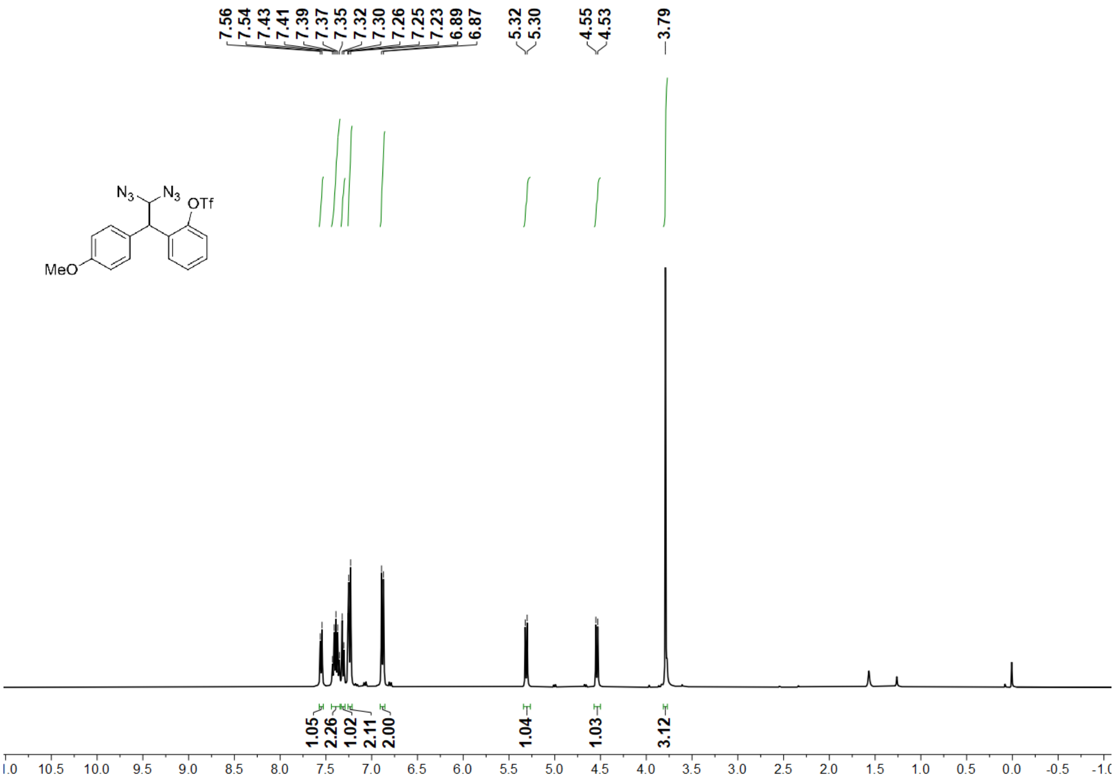


**Supplementary Figure 166.** $^1$H NMR (400 MHz, $CDCl_3$) spectrum of compound **2ao**

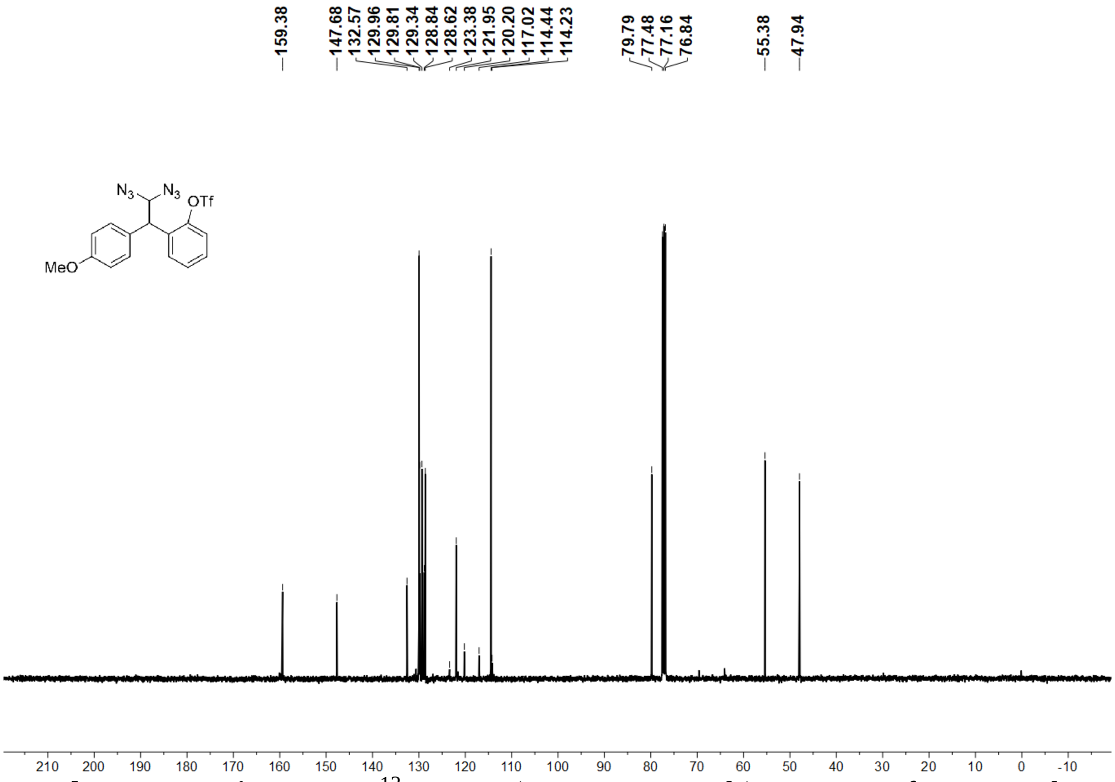


**Supplementary Figure 167.** $^{13}$C NMR (101 MHz, $CDCl_3$) spectrum of compound **2ao**

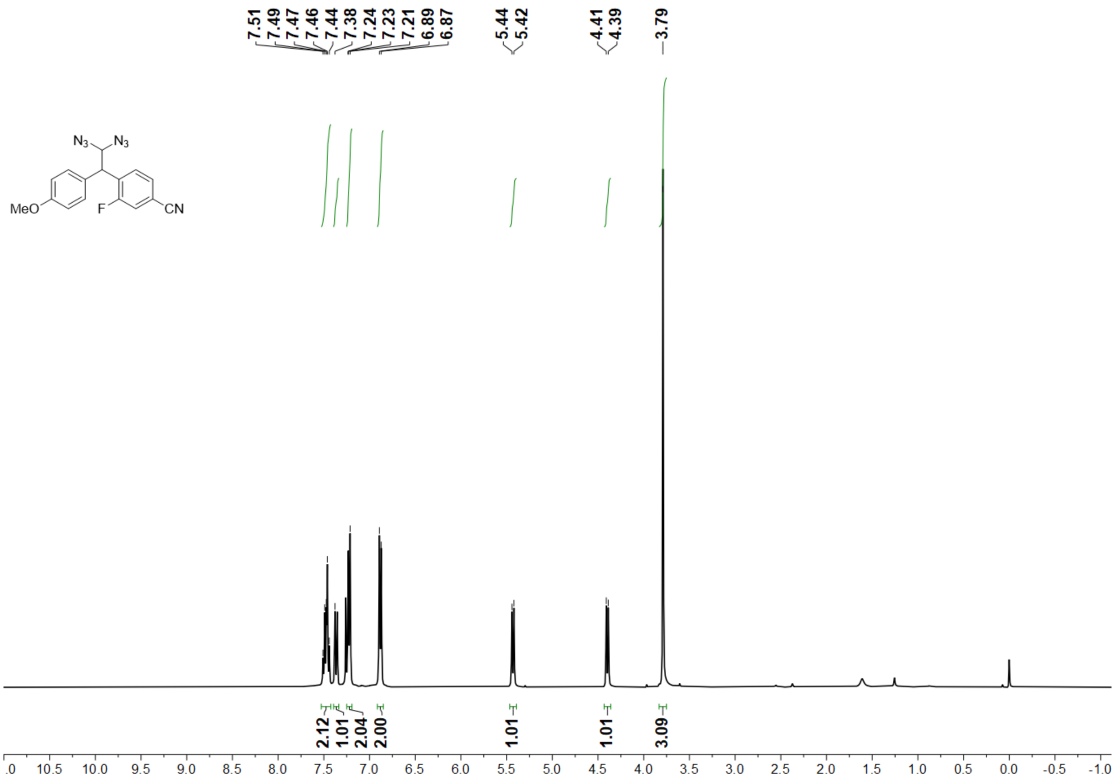


**Supplementary Figure 168.** $^{1}$H NMR (400 MHz, $CDCl_3$) spectrum of compound **2ap**

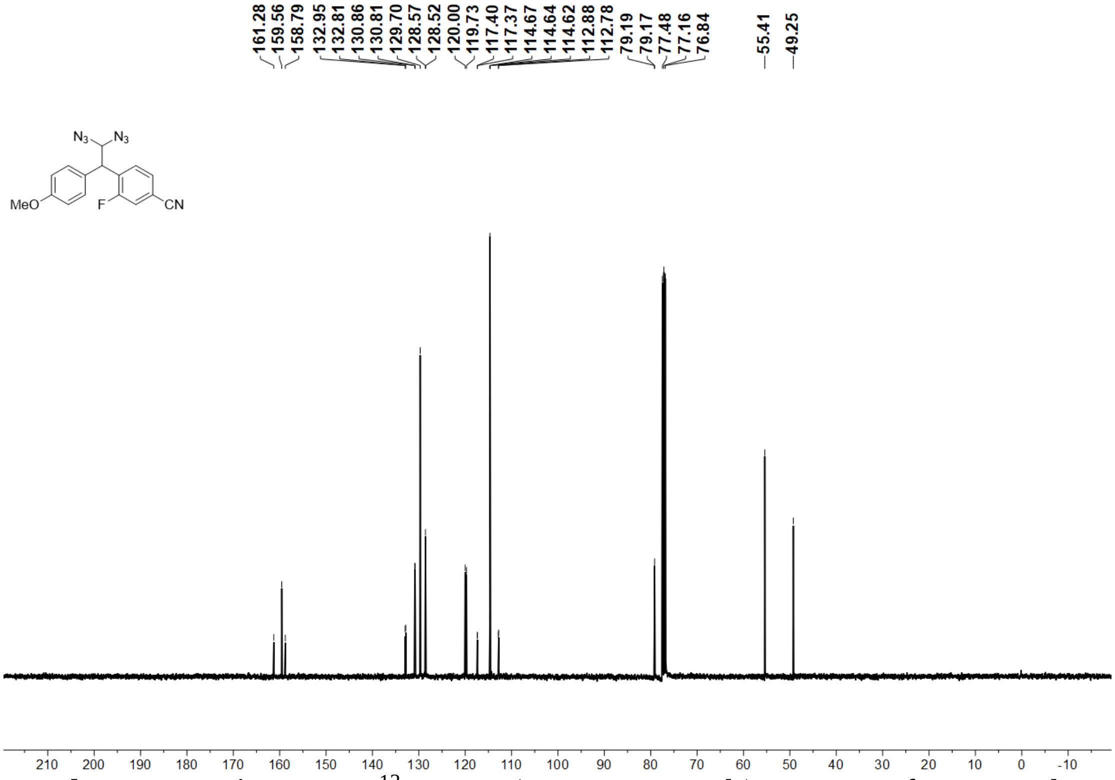


**Supplementary Figure 169.** $^{13}$C NMR (101 MHz, $CDCl_3$) spectrum of compound **2ap**

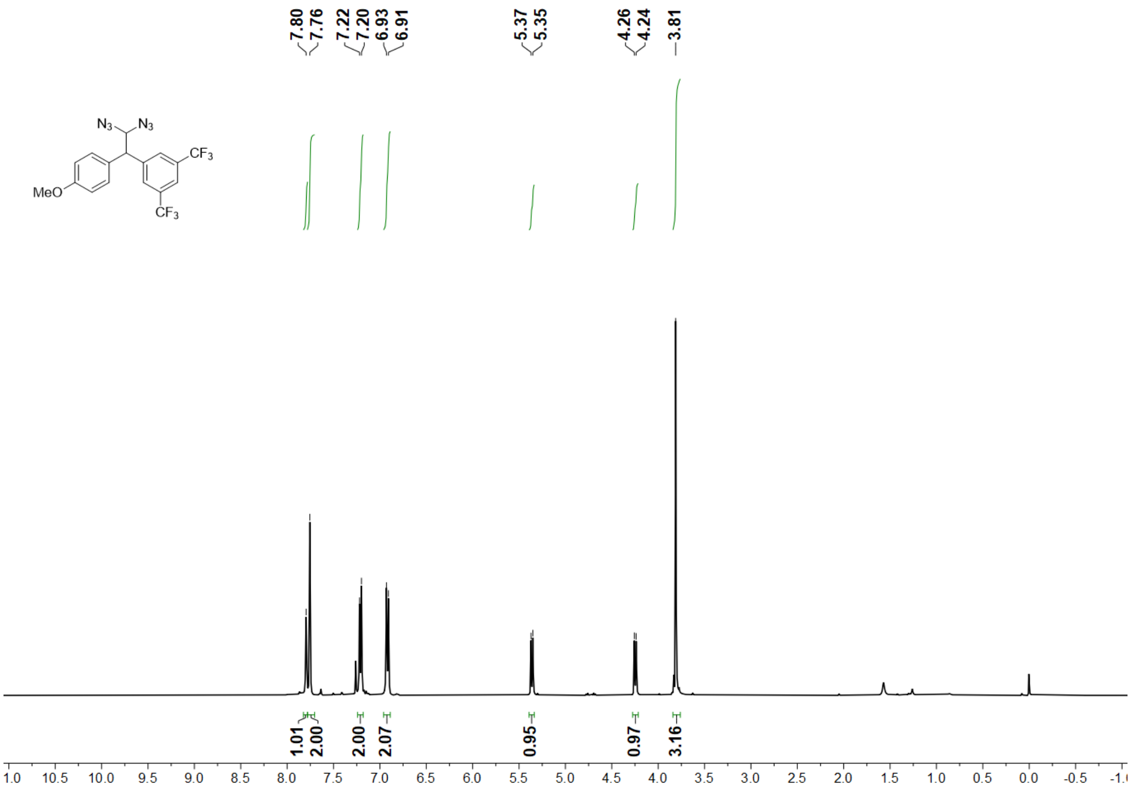

**Supplementary Figure 170.** $^{1}$H NMR (400 MHz, $CDCl_3$) spectrum of compound **2aq**

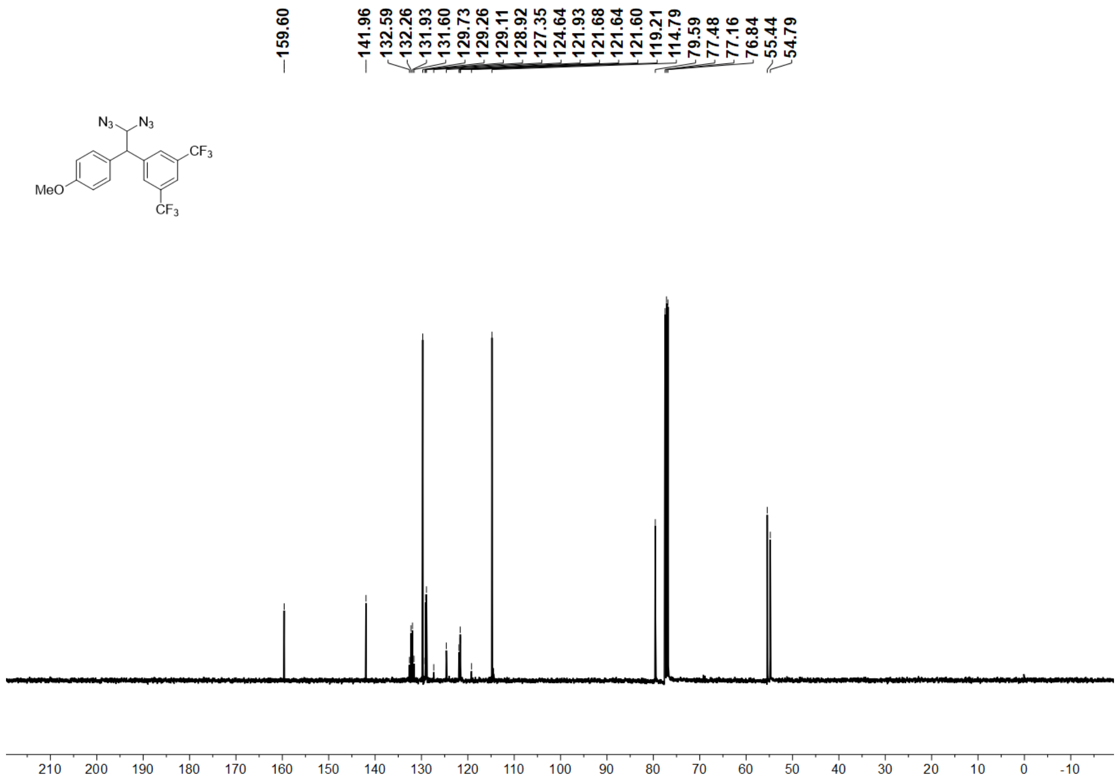

**Supplementary Figure 171.** $^{13}$C NMR (101 MHz, $CDCl_3$) spectrum of compound **2aq**

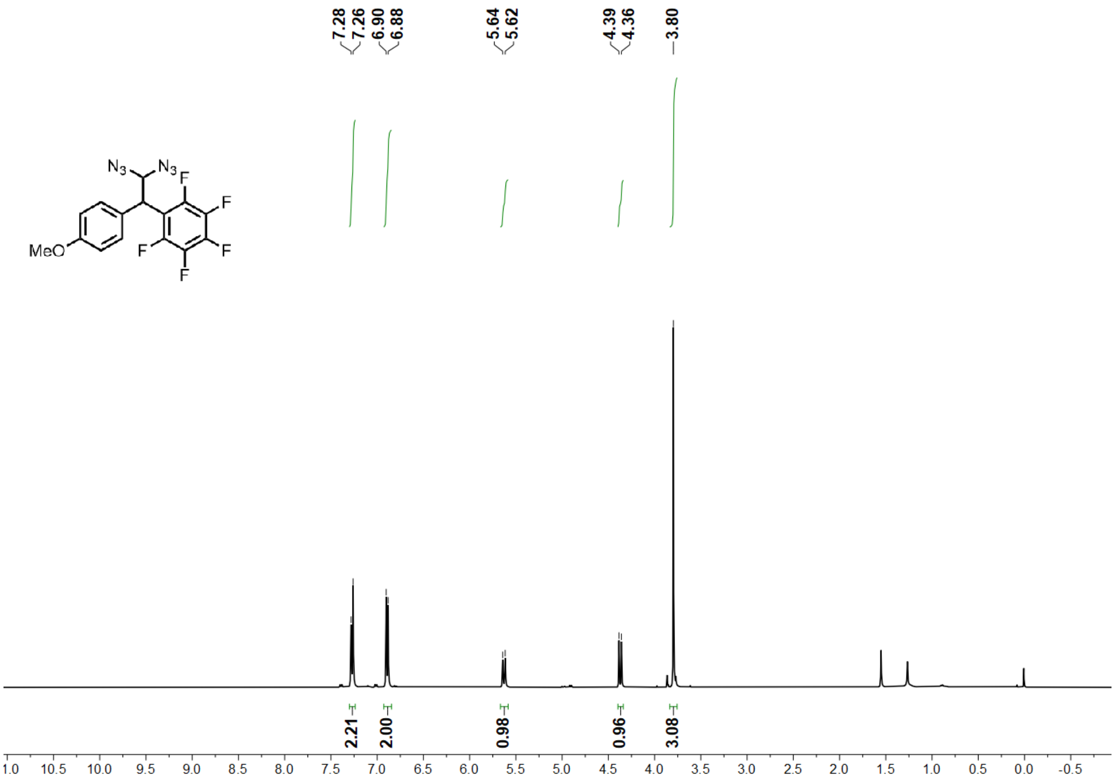

**Supplementary Figure 172.** $^{1}$H NMR (400 MHz, $CDCl_3$) spectrum of compound **2ar**

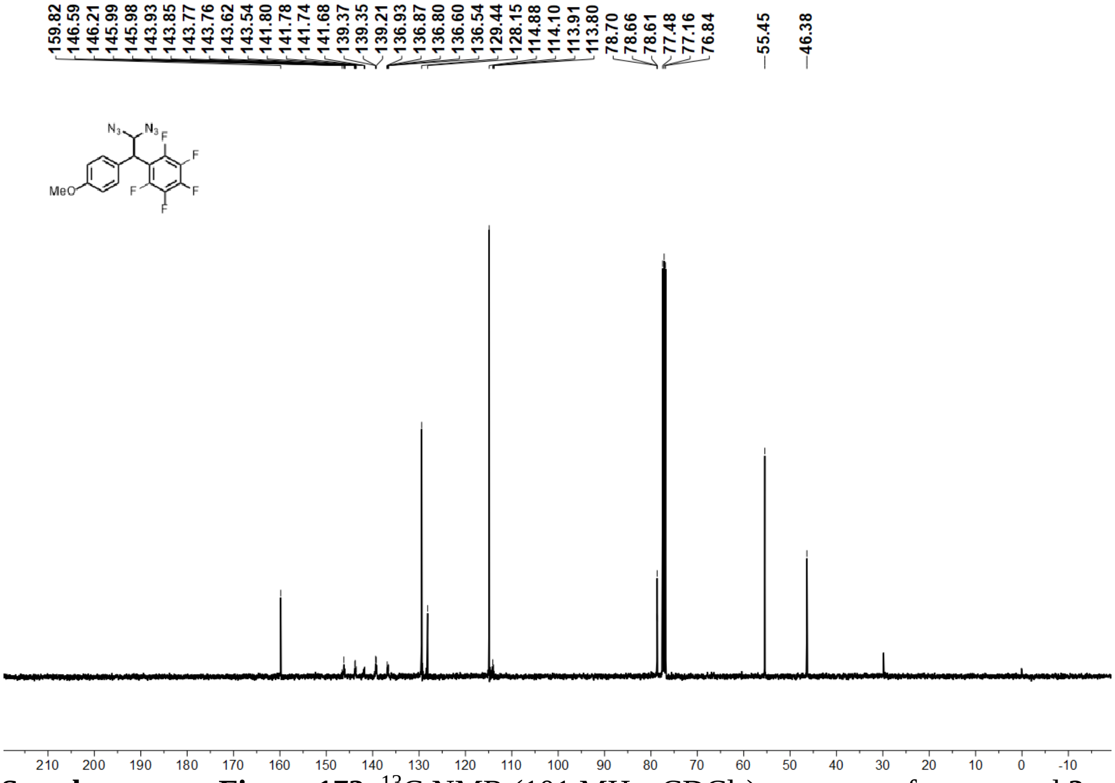

**Supplementary Figure 173.** $^{13}$C NMR (101 MHz, $CDCl_3$) spectrum of compound **2ar**

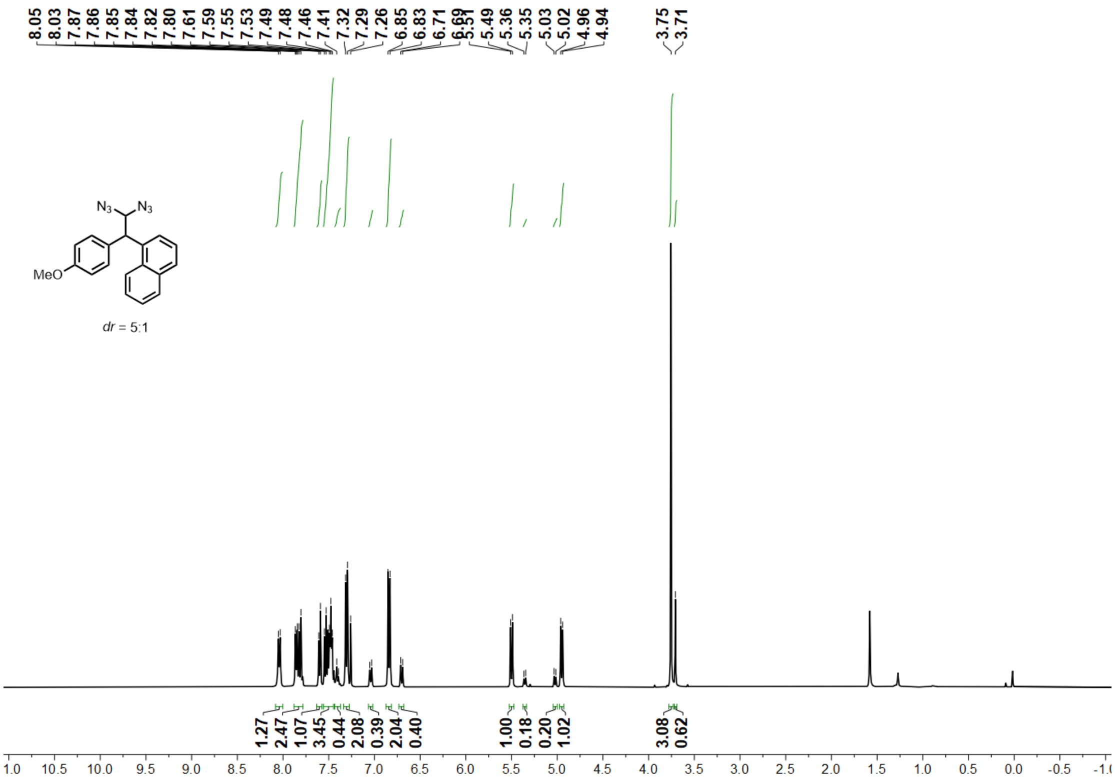

**Supplementary Figure 174.** $^{1}H$ NMR (400 MHz, $CDCl_3$) spectrum of compound **2as**

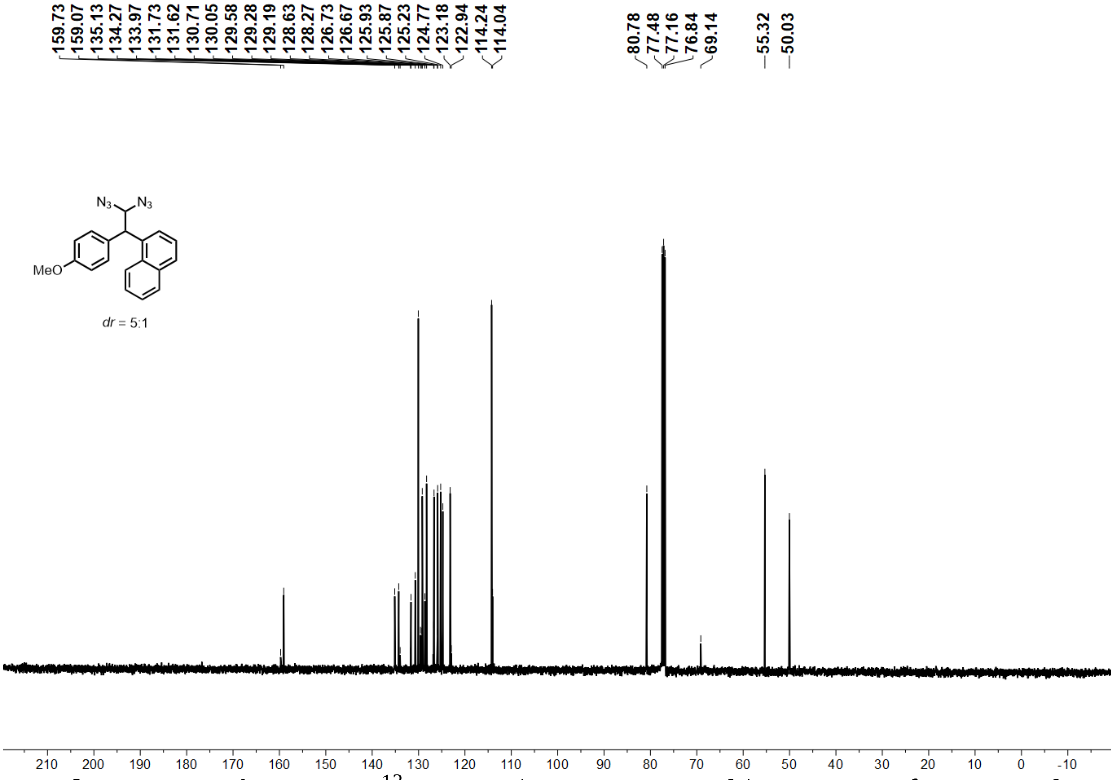

**Supplementary Figure 175.** $^{13}C$ NMR (101 MHz, $CDCl_3$) spectrum of compound **2as**

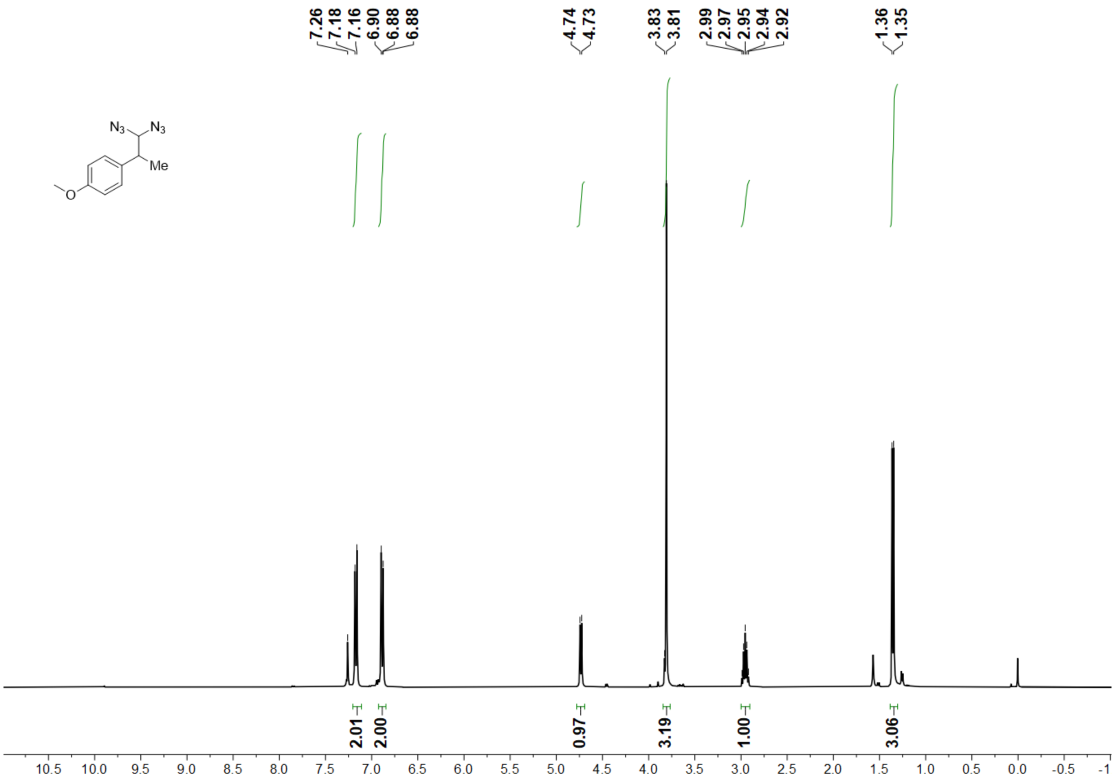


**Supplementary Figure 176.** $^{1}H$ NMR (400 MHz, $CDCl_3$) spectrum of compound **2at**

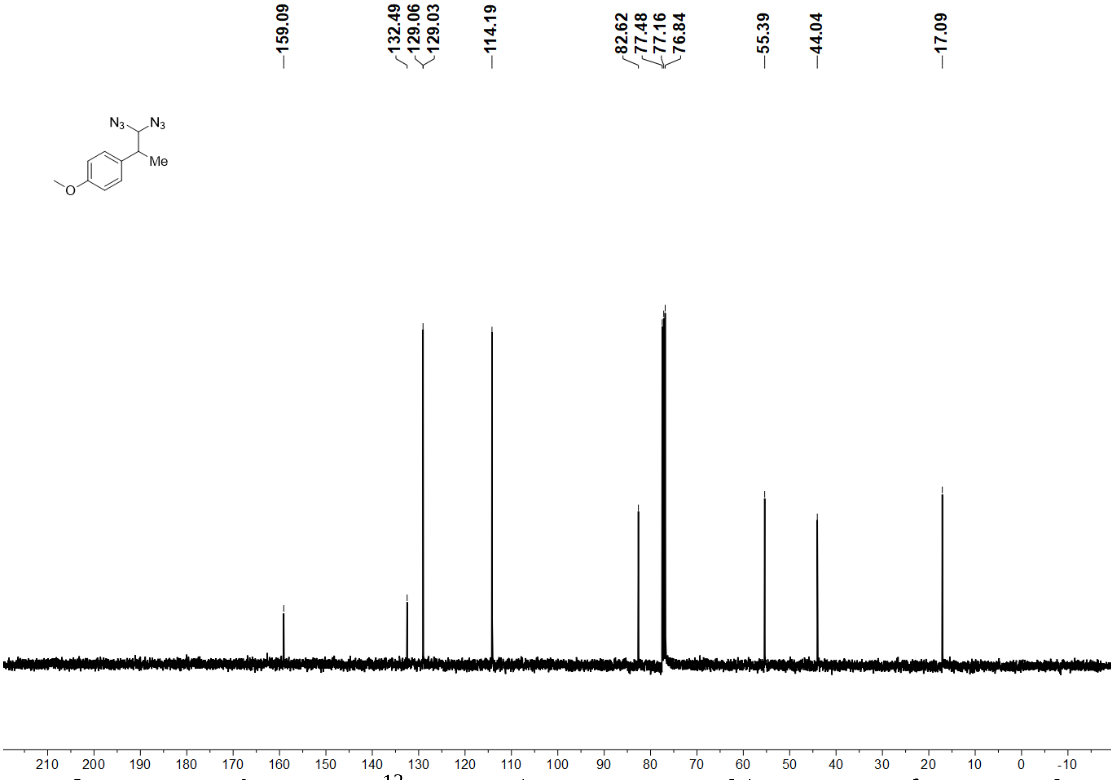


**Supplementary Figure 177.** $^{13}C$ NMR (101 MHz, $CDCl_3$) spectrum of compound **2at**

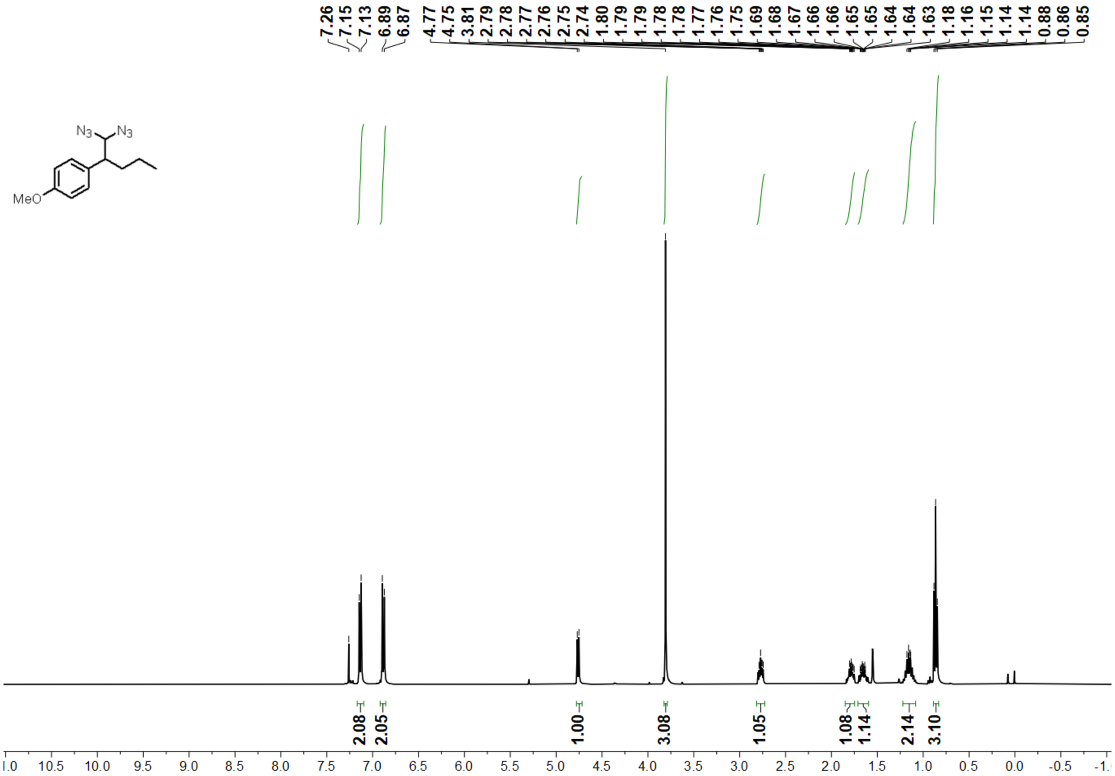

**Supplementary Figure 178.** $^{1}$H NMR (400 MHz, $CDCl_3$) spectrum of compound **2au**

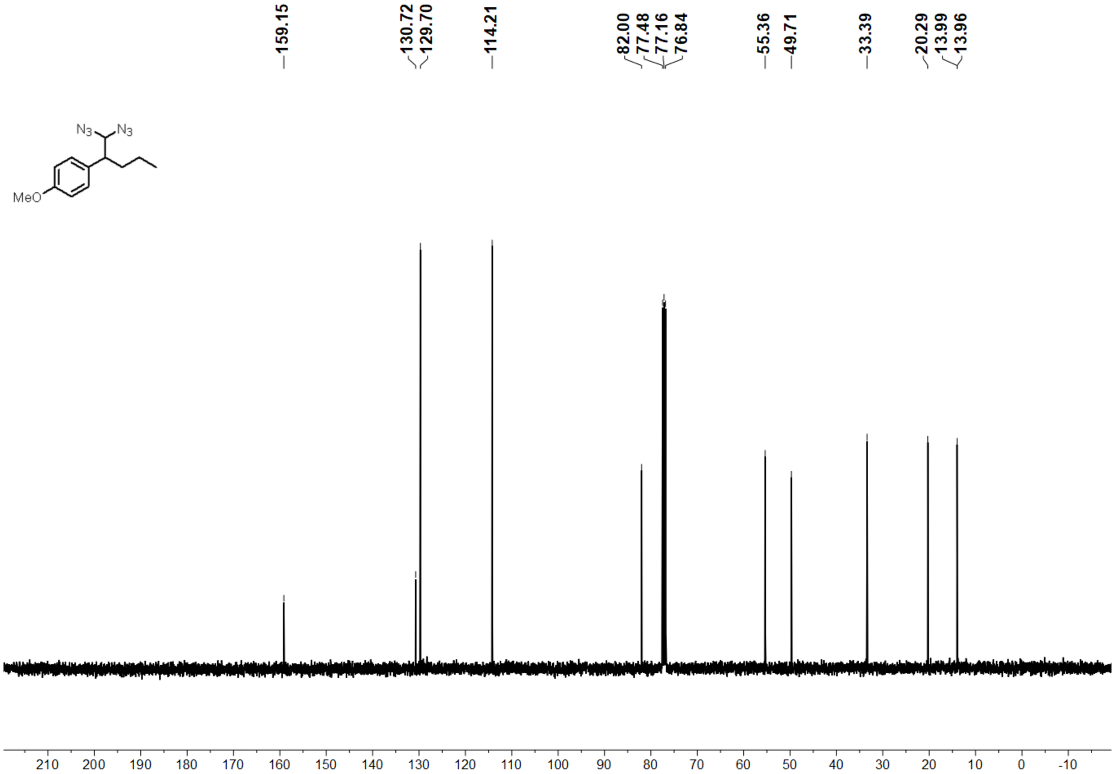

**Supplementary Figure 179.** $^{13}$C NMR (101 MHz, $CDCl_3$) spectrum of compound **2au**

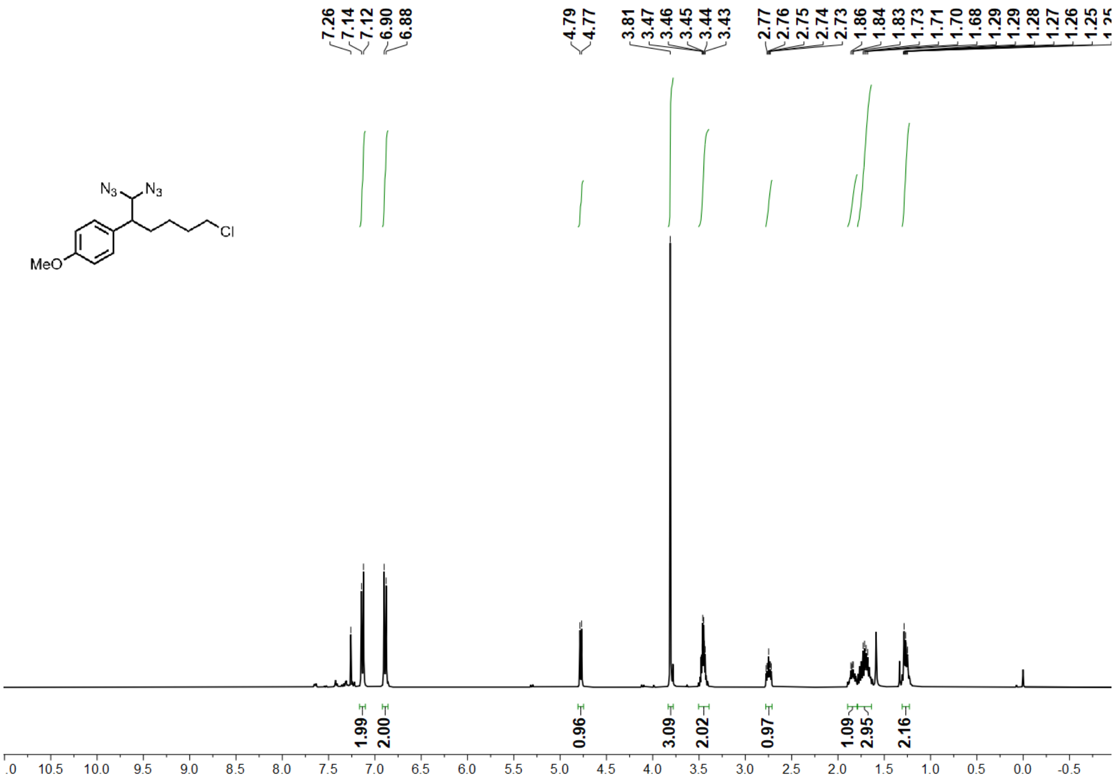

**Supplementary Figure 180.** $^{1}H$ NMR (400 MHz, $CDCl_3$) spectrum of compound **2av**

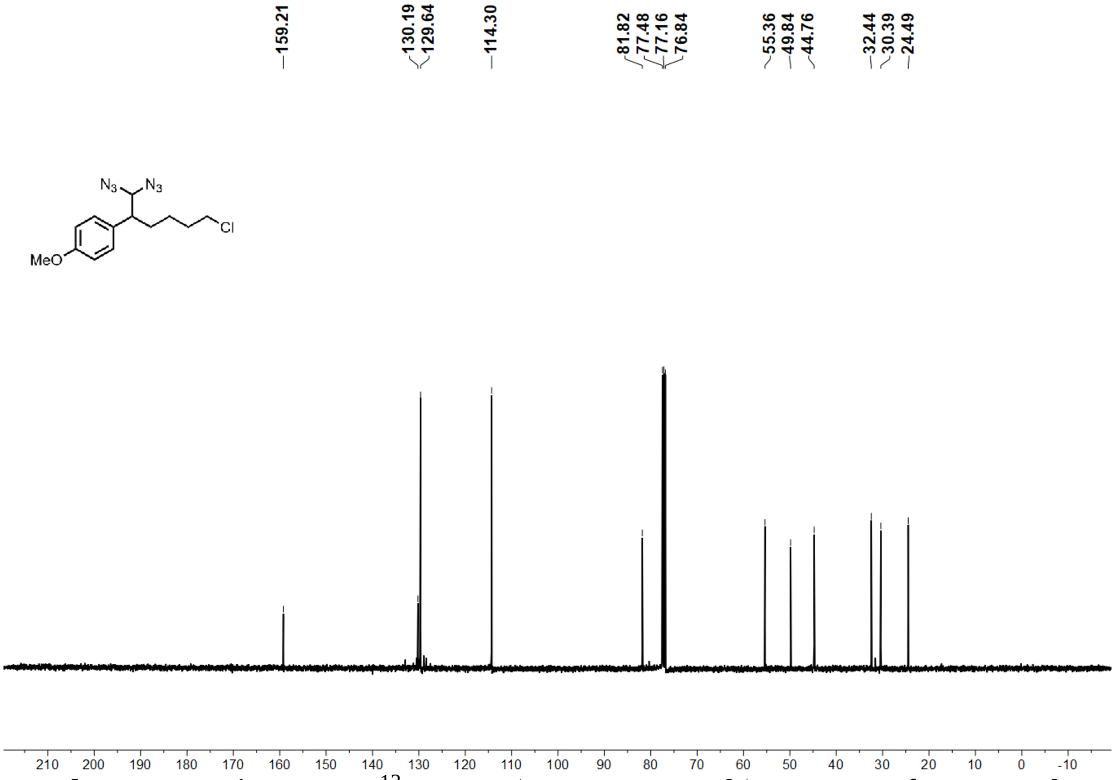

**Supplementary Figure 181.** $^{13}C$ NMR (101 MHz, $CDCl_3$) spectrum of compound **2av**

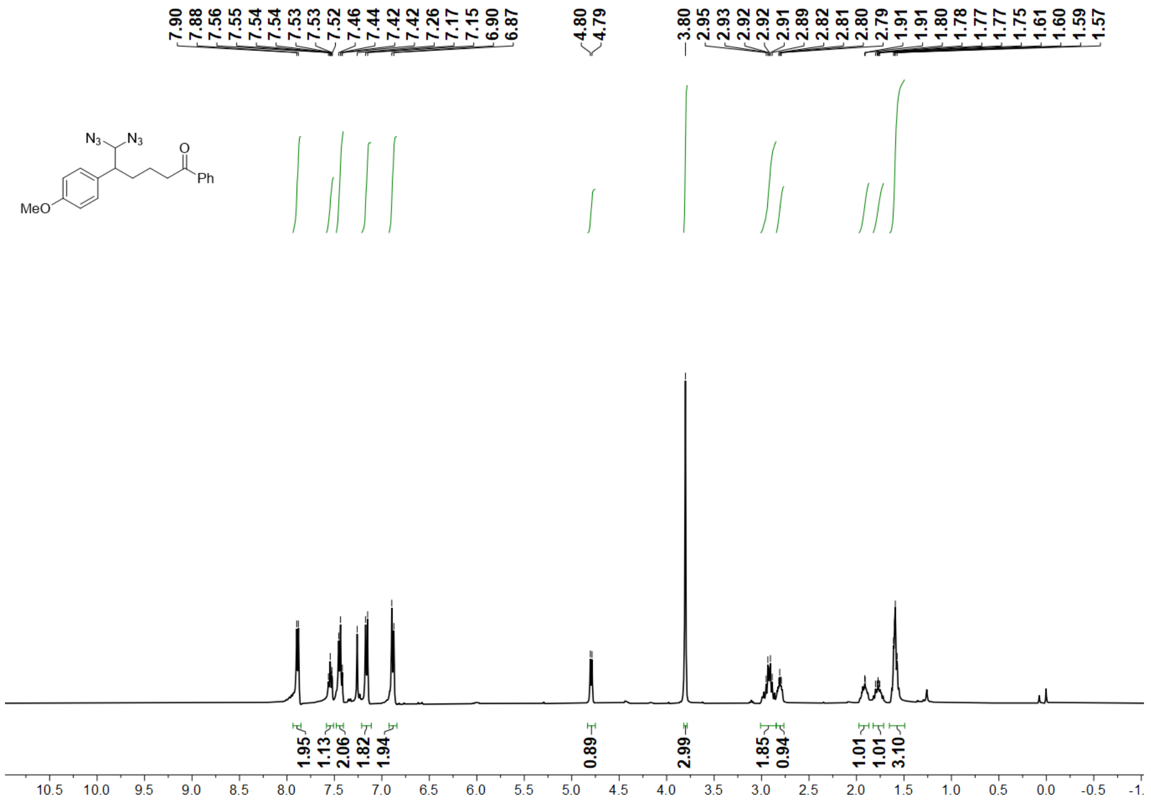

**Supplementary Figure 182.** $^{1}$H NMR (400 MHz, $CDCl_3$) spectrum of compound **2aw**

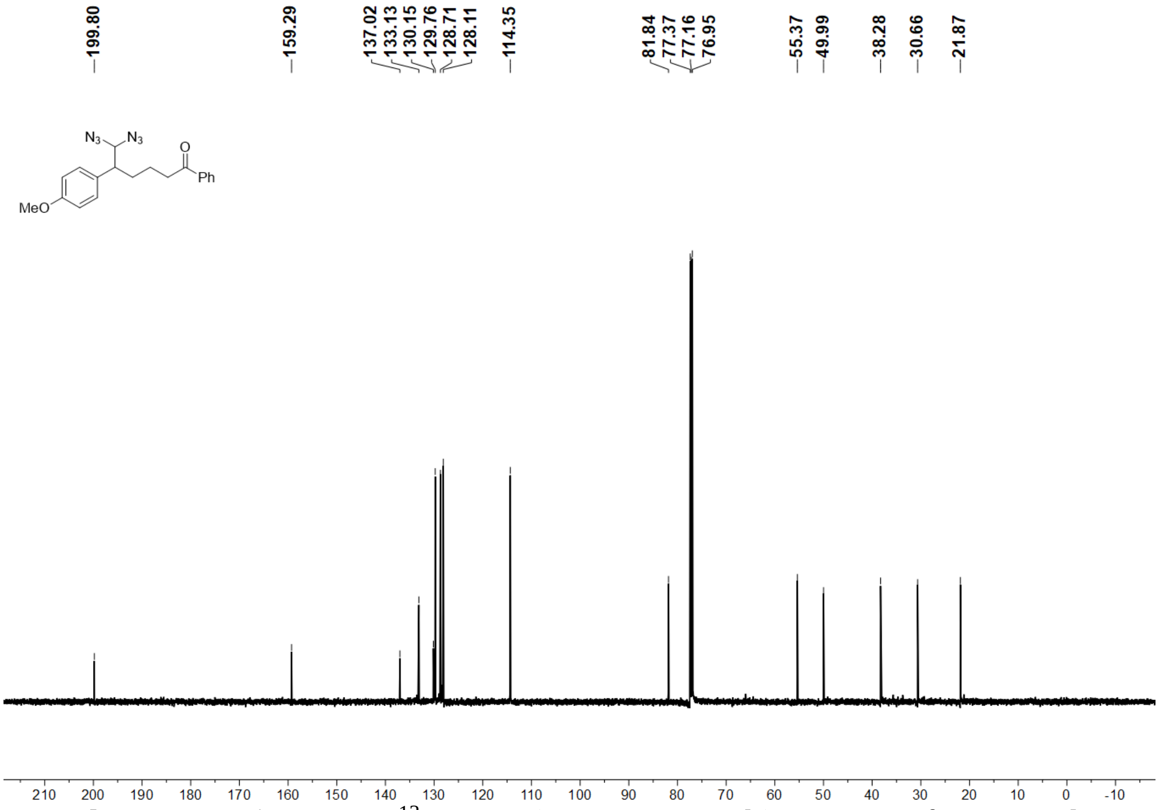

**Supplementary Figure 183.** $^{13}$C NMR (151 MHz, $CDCl_3$) spectrum of compound **2aw**

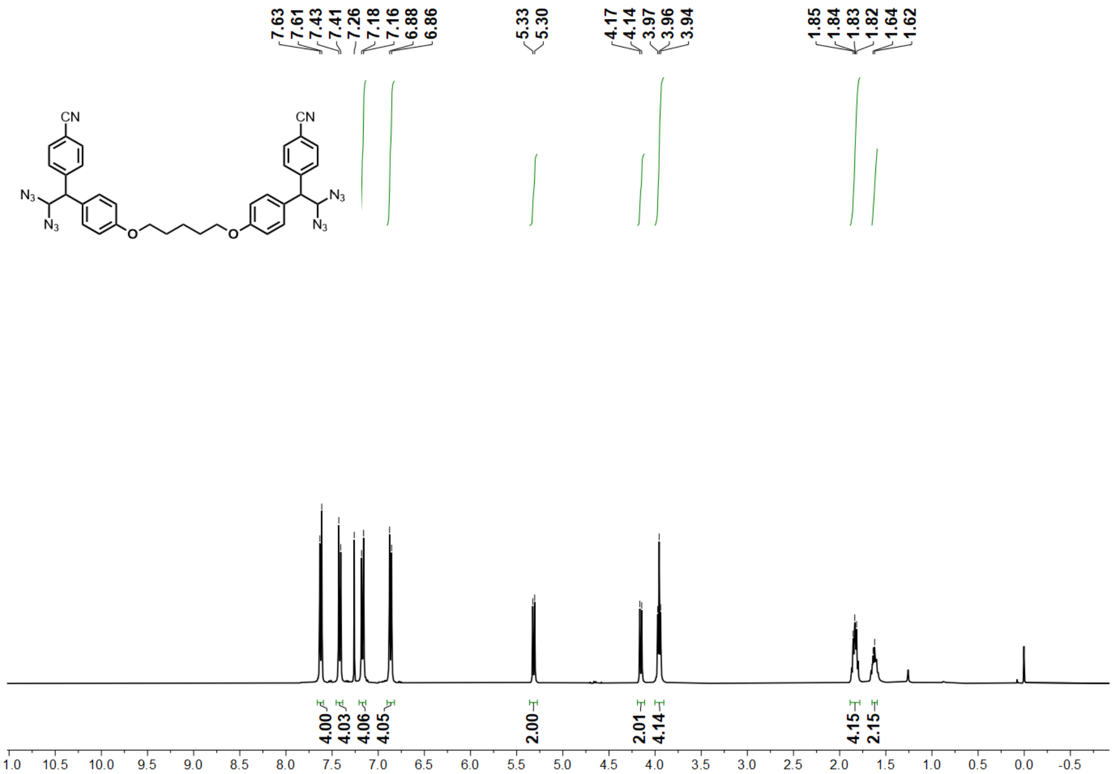


**Supplementary Figure 184.** $^{1}H$ NMR (400 MHz, $CDCl_3$) spectrum of compound **2ax**

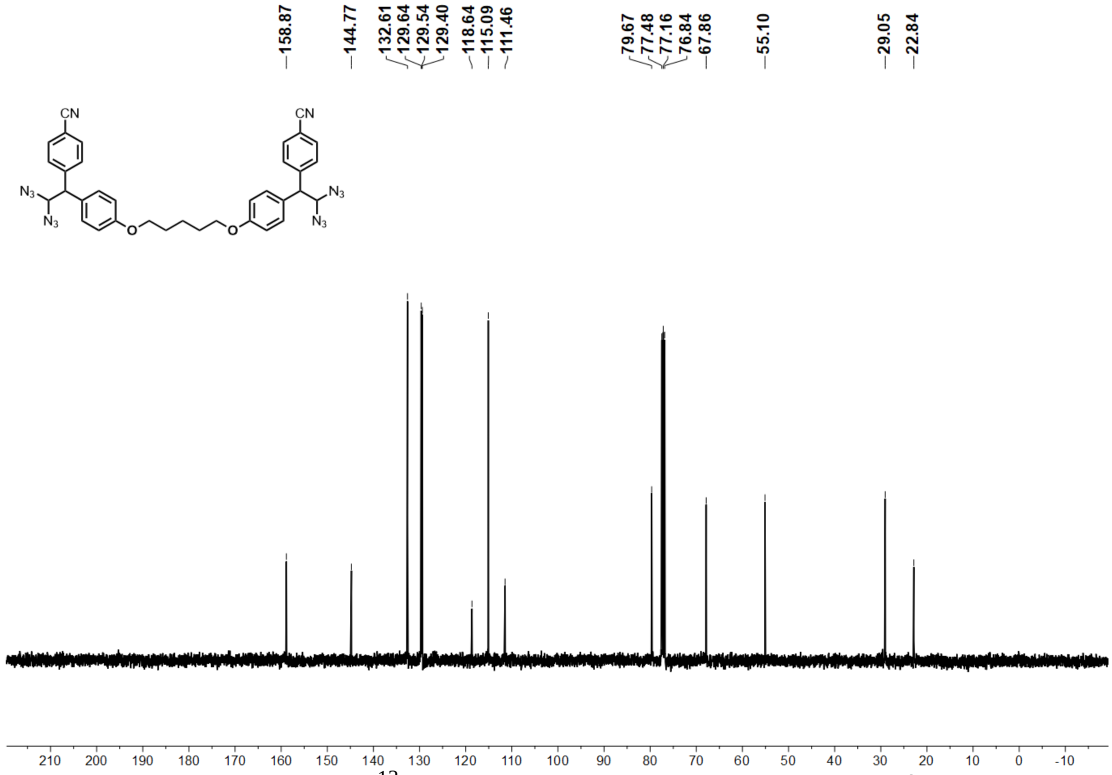


**Supplementary Figure 185.** $^{13}C$ NMR (101 MHz, $CDCl_3$) spectrum of compound **2ax**

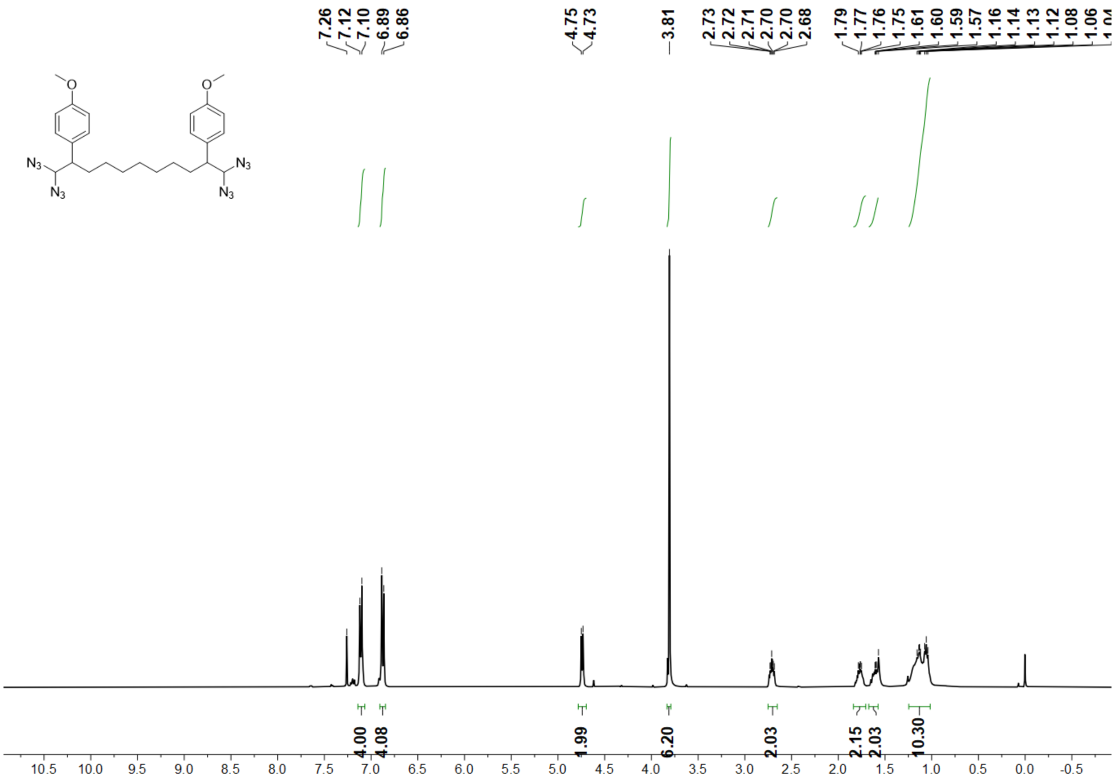


**Supplementary Figure 186.** $^{1}H$ NMR (400 MHz, $CDCl_3$) spectrum of compound **2ay**

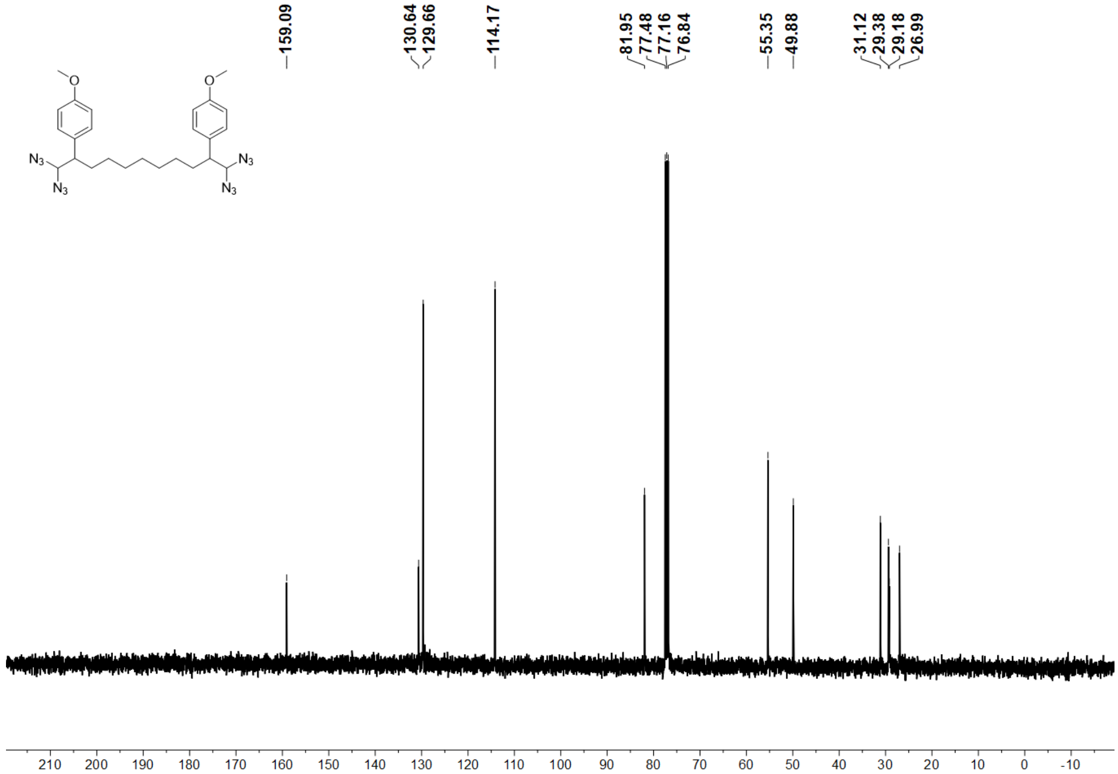


**Supplementary Figure 187.** $^{13}C$ NMR (101 MHz, $CDCl_3$) spectrum of compound **2ay**

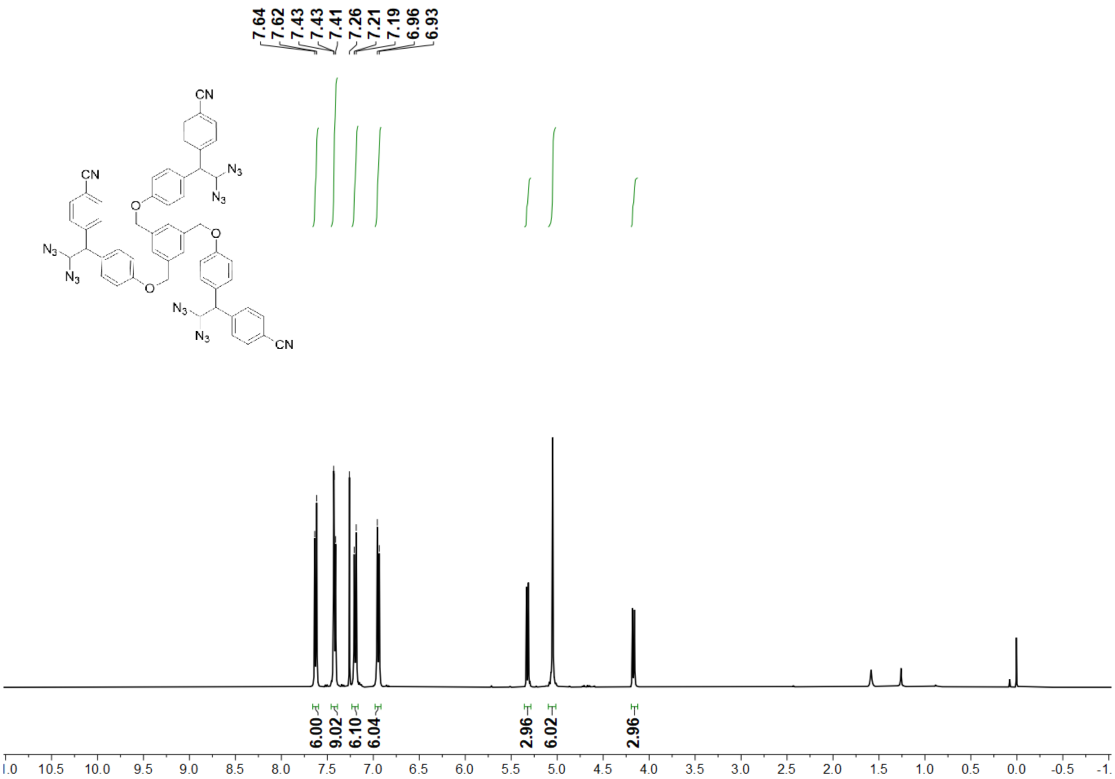

**Supplementary Figure 188.** $^{1}H$ NMR (400 MHz, $CDCl_3$) spectrum of compound **2az**

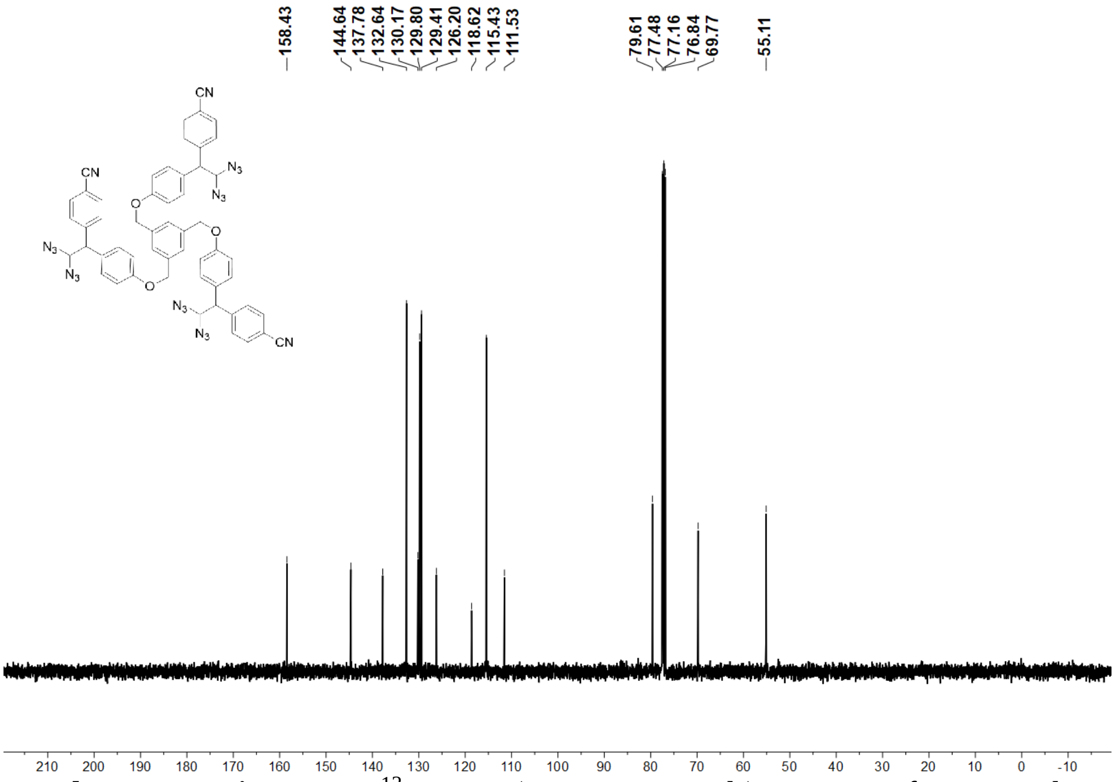

**Supplementary Figure 189.** $^{13}C$ NMR (101 MHz, $CDCl_3$) spectrum of compound **2az**

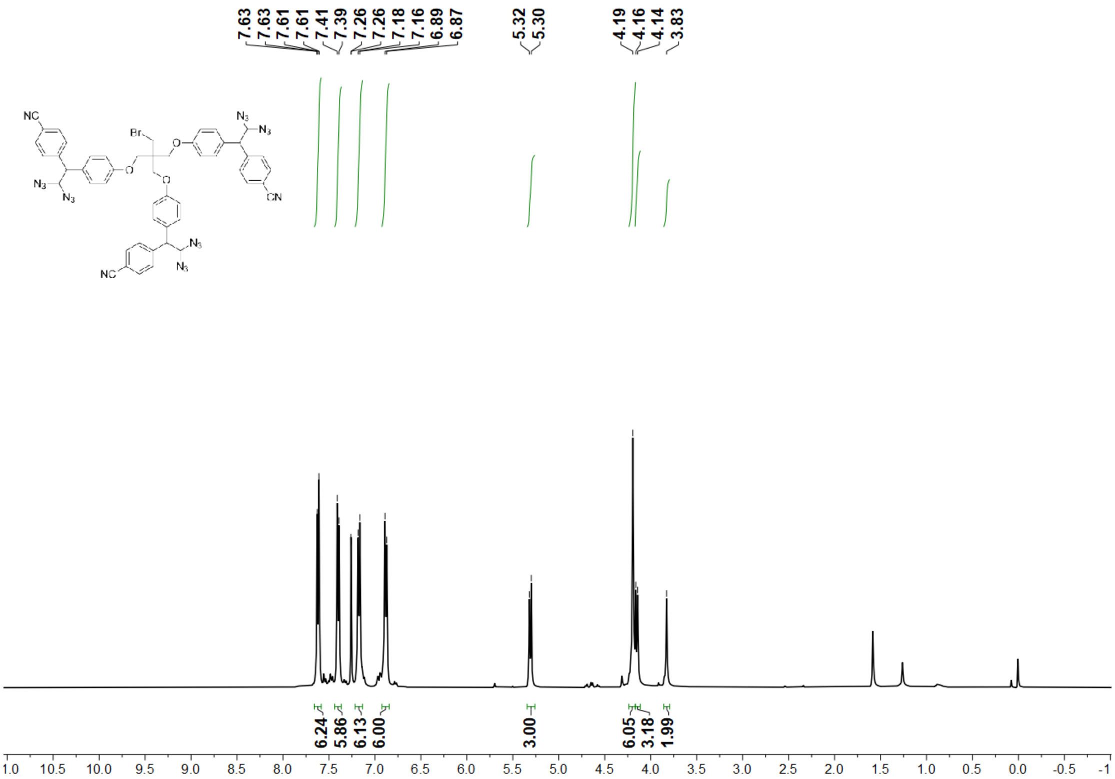

**Supplementary Figure 190.** $^{1}H$ NMR (400 MHz, $CDCl_3$) spectrum of compound **2ba**

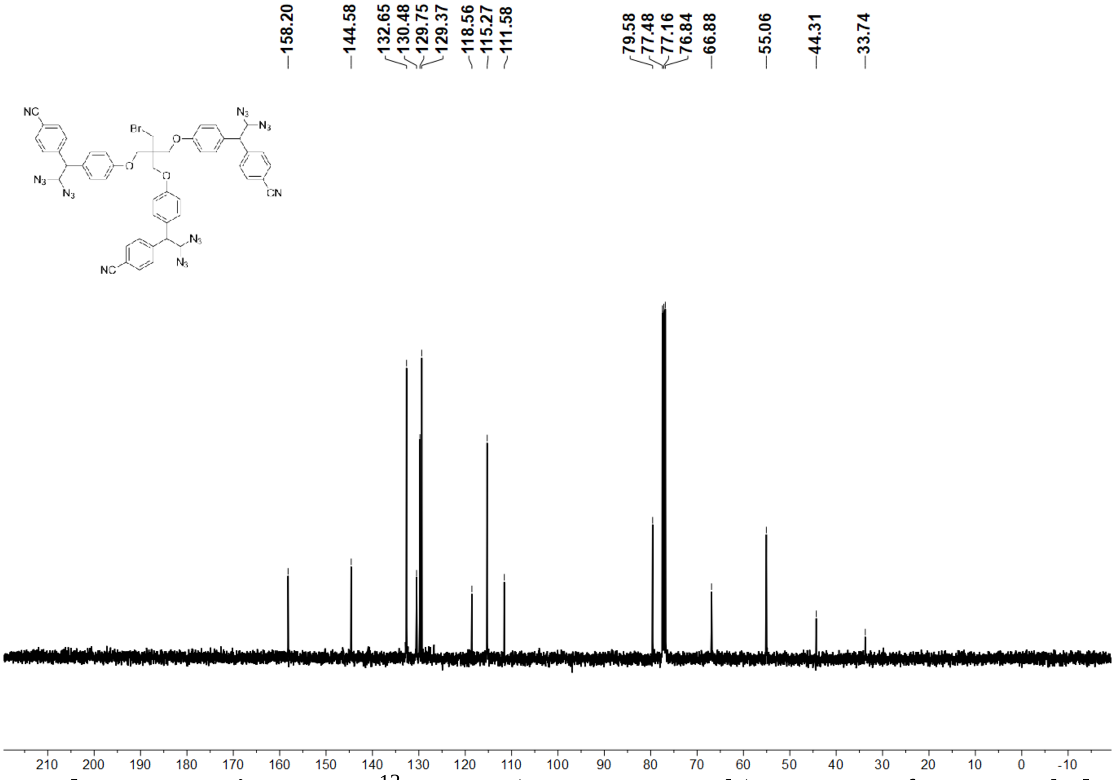

**Supplementary Figure 191.** $^{13}C$ NMR (101 MHz, $CDCl_3$) spectrum of compound **2ba**

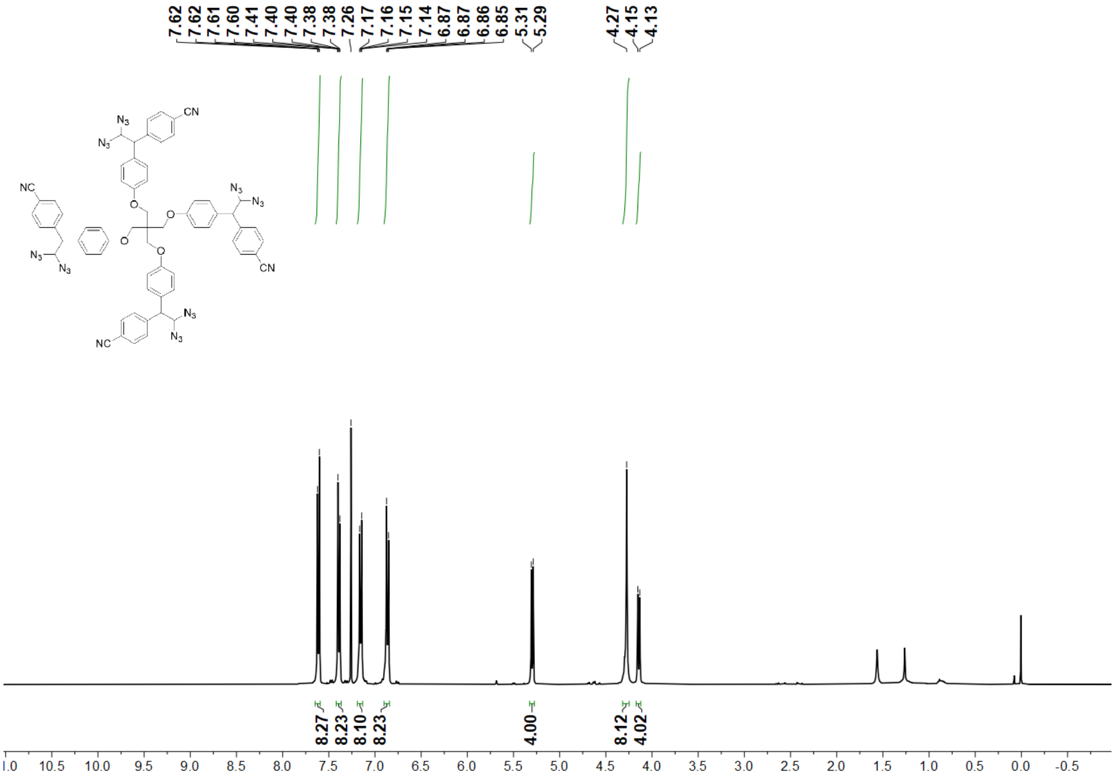

**Supplementary Figure 192.** $^1$H NMR (400 MHz, $CDCl_3$) spectrum of compound **2bb**

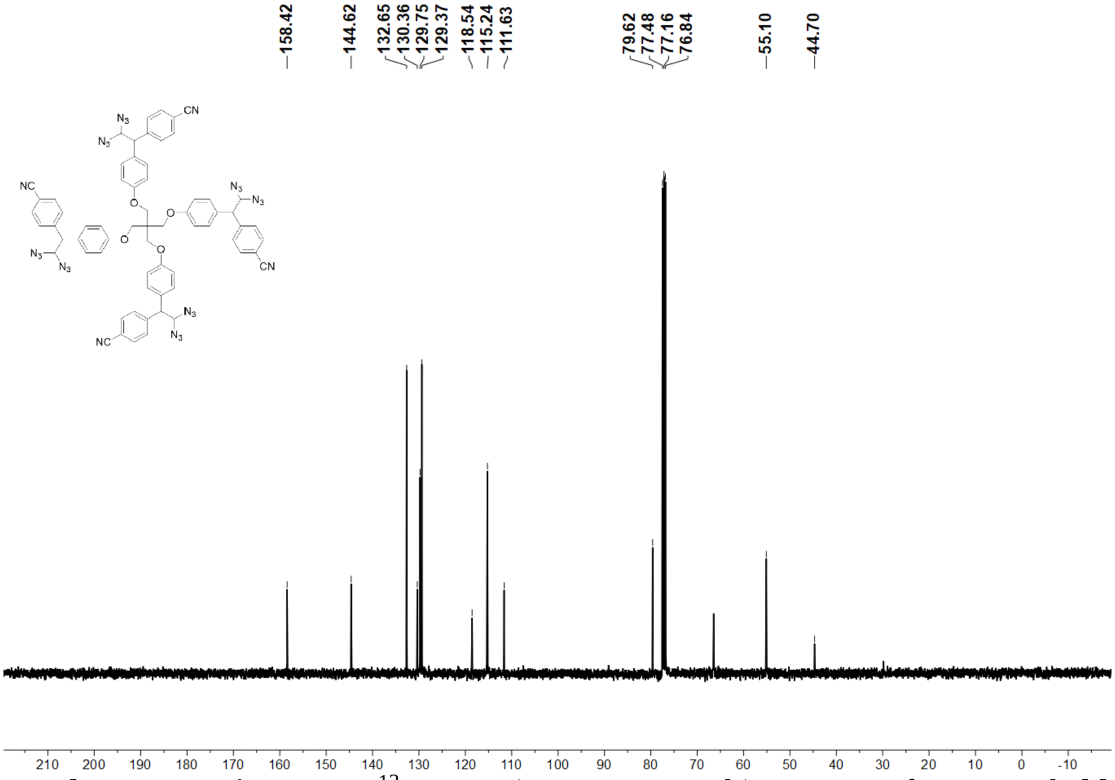

**Supplementary Figure 193.** $^{13}$C NMR (101 MHz, $CDCl_3$) spectrum of compound **2bb**

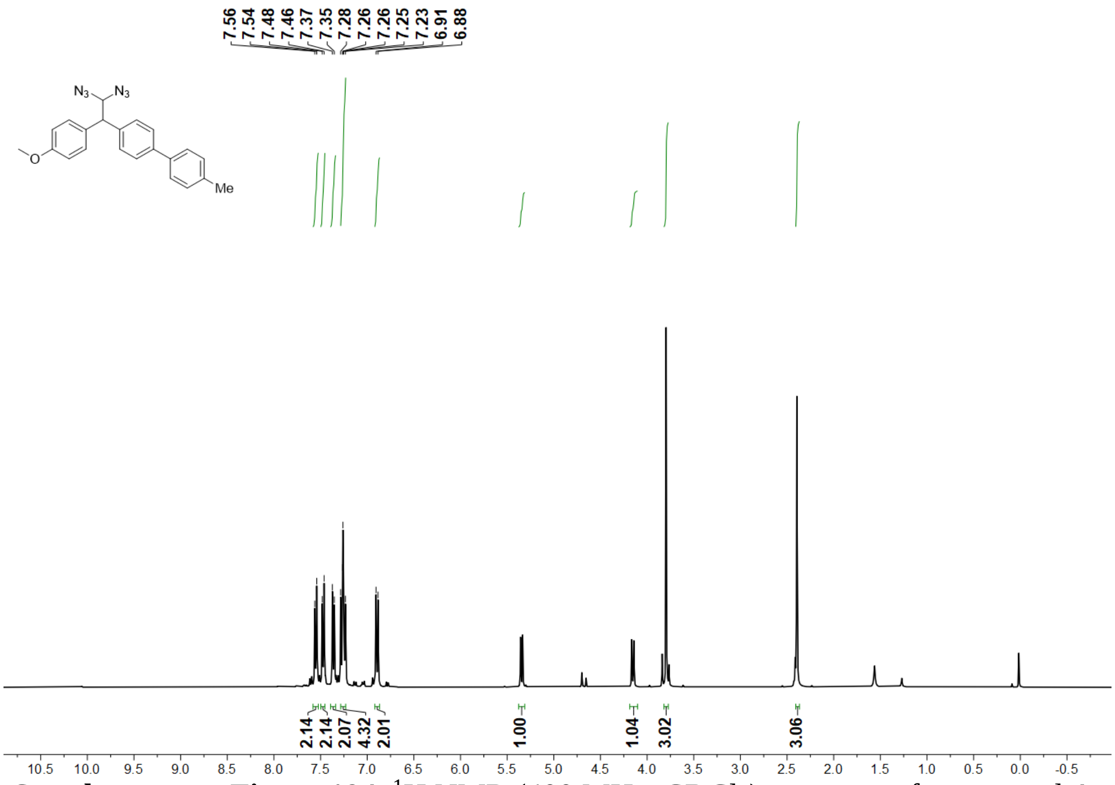

**Supplementary Figure 194.** $^{1}$H NMR (400 MHz, $CDCl_3$) spectrum of compound **4a**

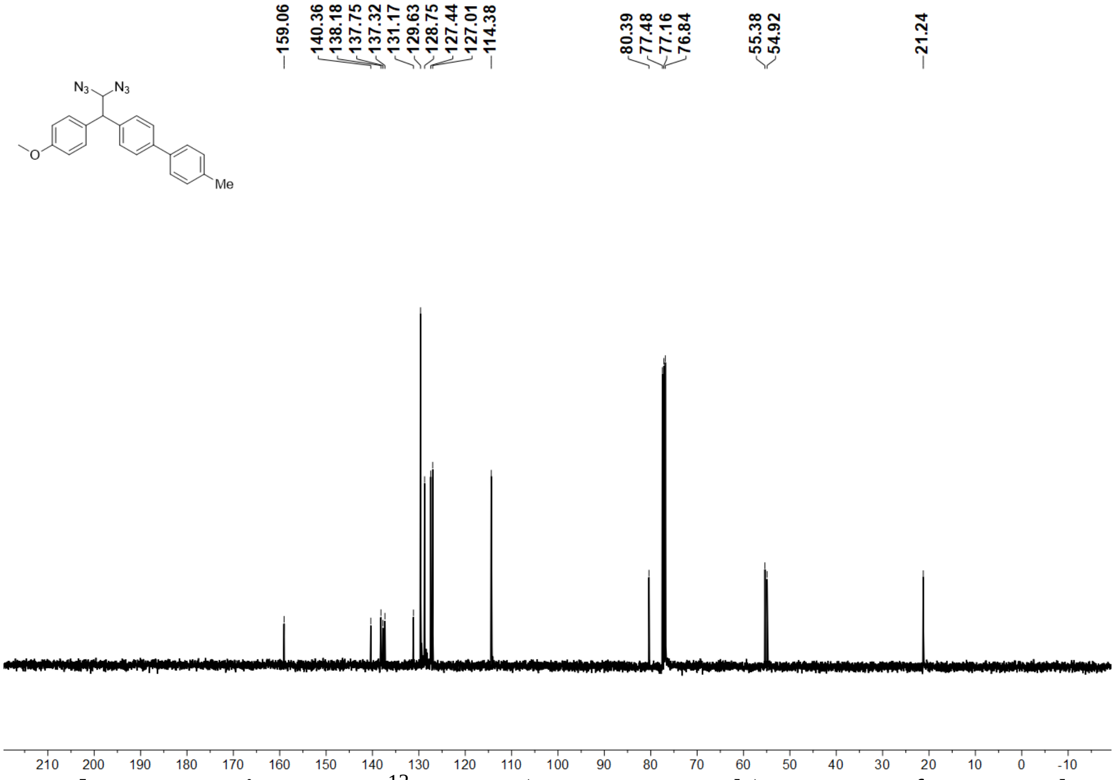

**Supplementary Figure 195.** $^{13}$C NMR (101 MHz, $CDCl_3$) spectrum of compound **4a**

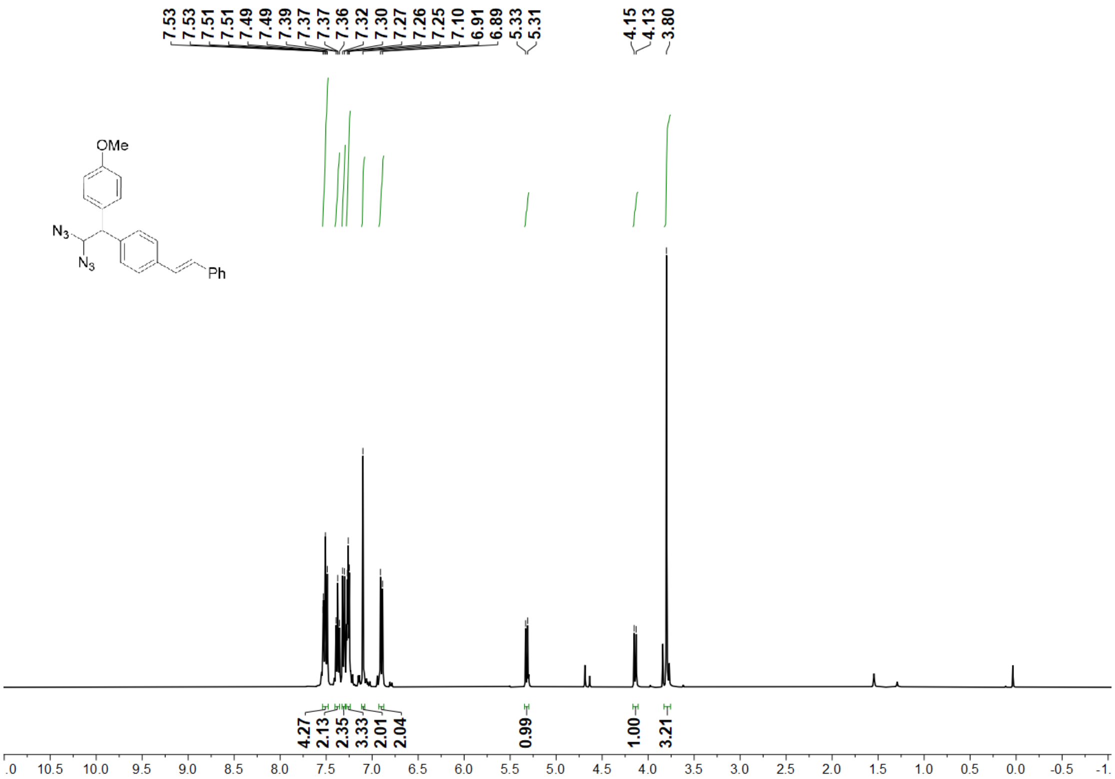

Supplementary Figure 196. $^{1}H$ NMR (400 MHz, $CDCl_3$) spectrum of compound **4b**

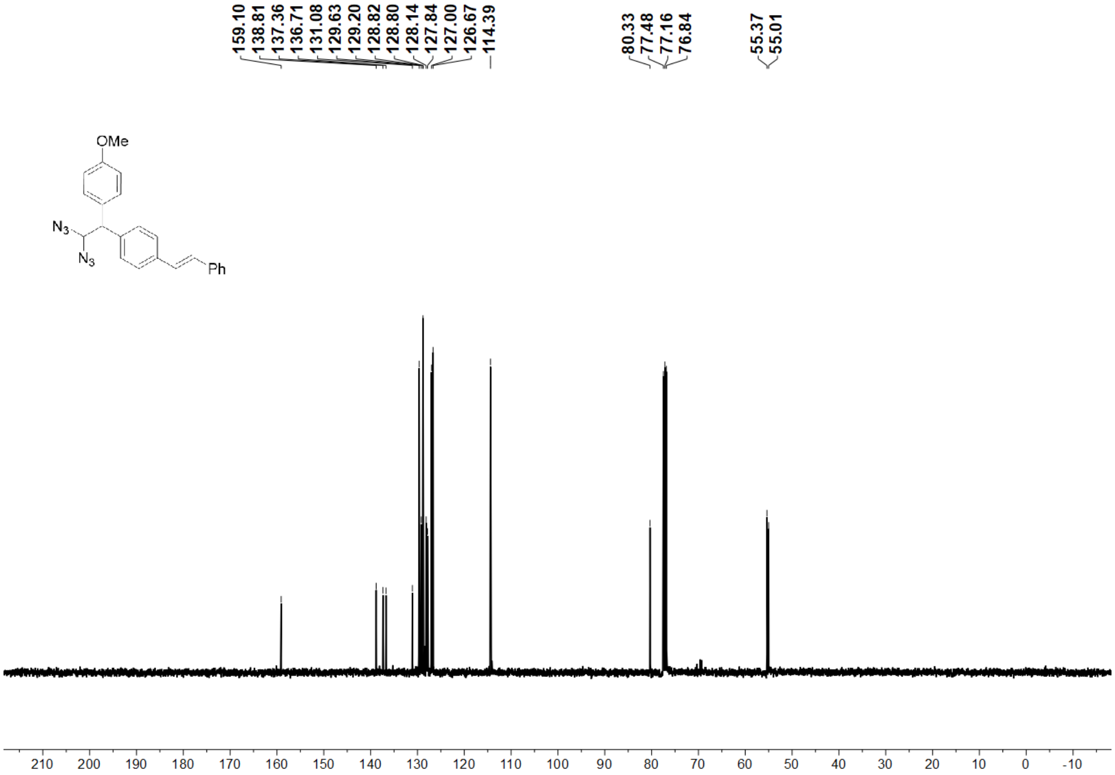

**Supplementary Figure 197.** $^{13}C$ NMR (101 MHz, $CDCl_3$) spectrum of compound **4b**

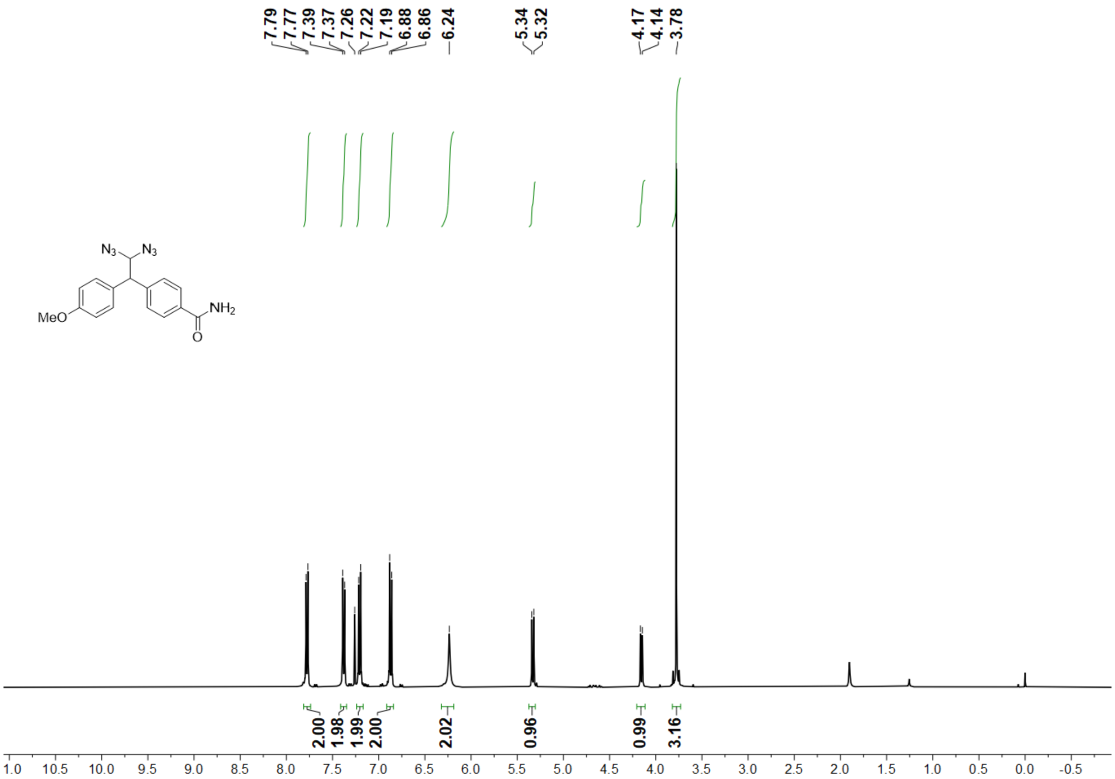


**Supplementary Figure 198.** $^{1}H$ NMR (400 MHz, $CDCl_3$) spectrum of compound **4c**

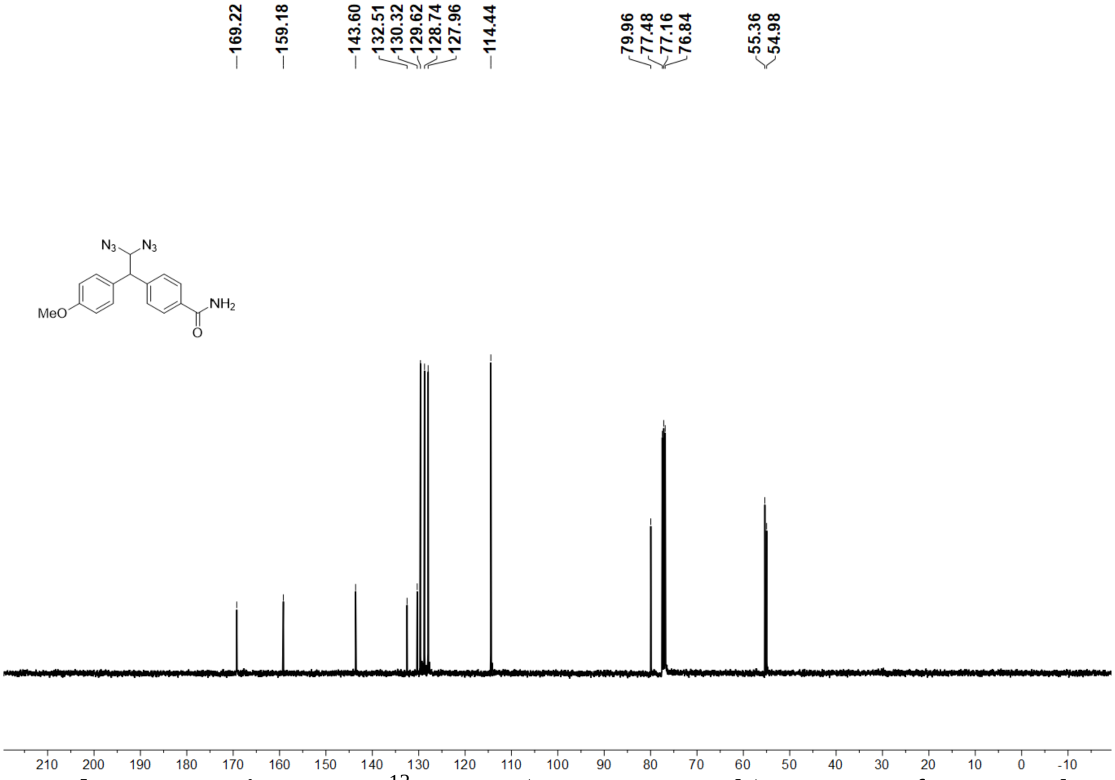


**Supplementary Figure 199.** $^{13}C$ NMR (101 MHz, $CDCl_3$) spectrum of compound **4c**

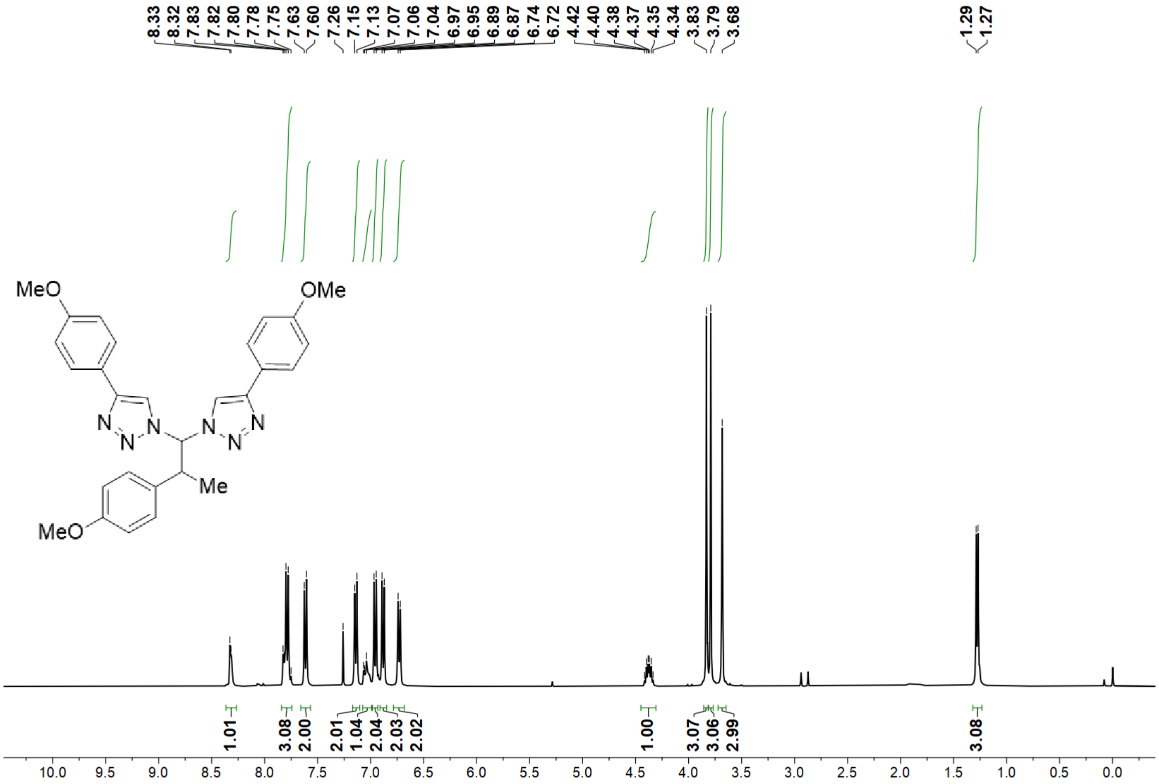

**Supplementary Figure 200.** $^{1}$H NMR (400 MHz, $CDCl_3$) spectrum of compound **4d**

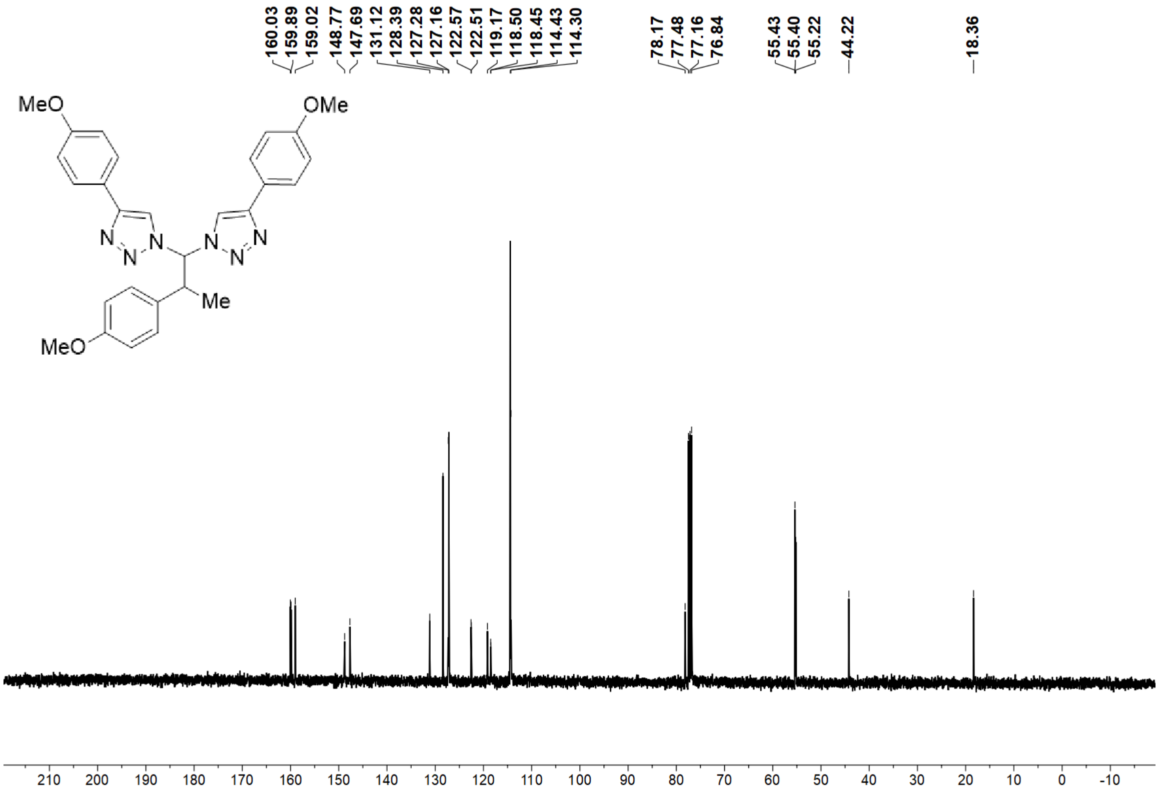

**Supplementary Figure 201.** $^{13}$C NMR (101 MHz, $CDCl_3$) spectrum of compound **4d**

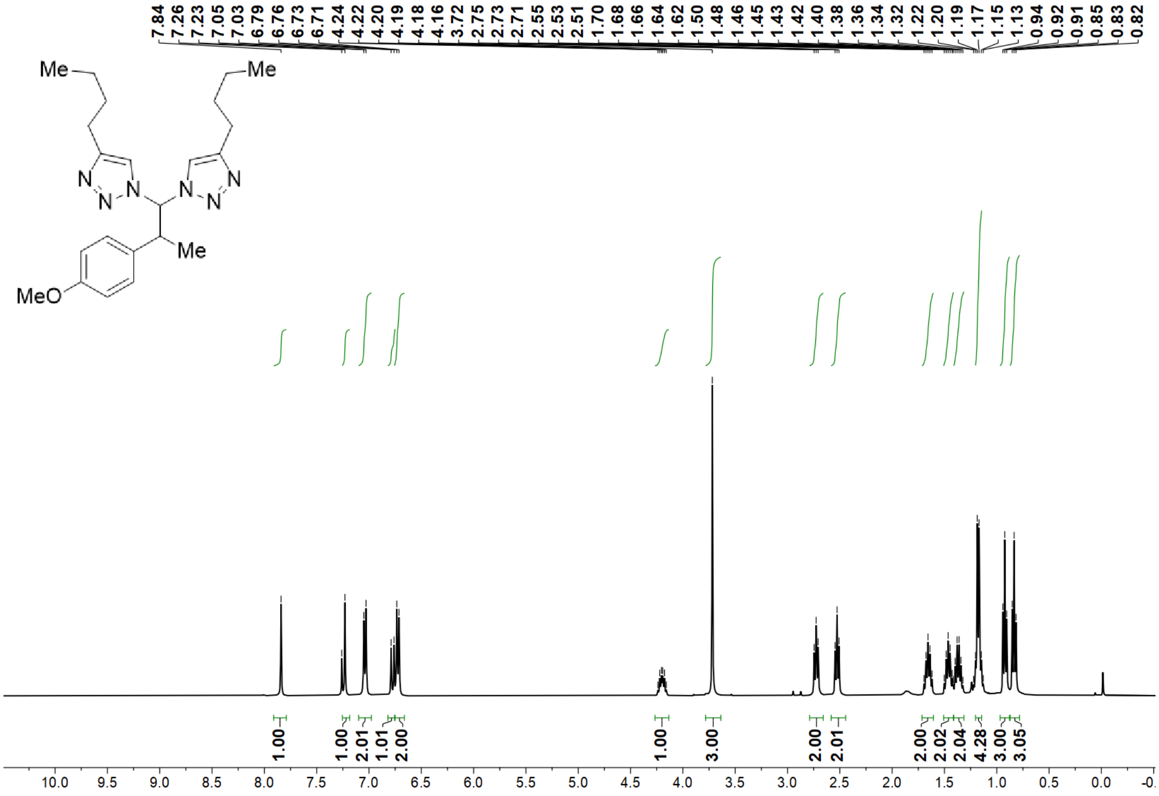


**Supplementary Figure 202.** $^{1}$H NMR (400 MHz, $CDCl_3$) spectrum of compound **4e**

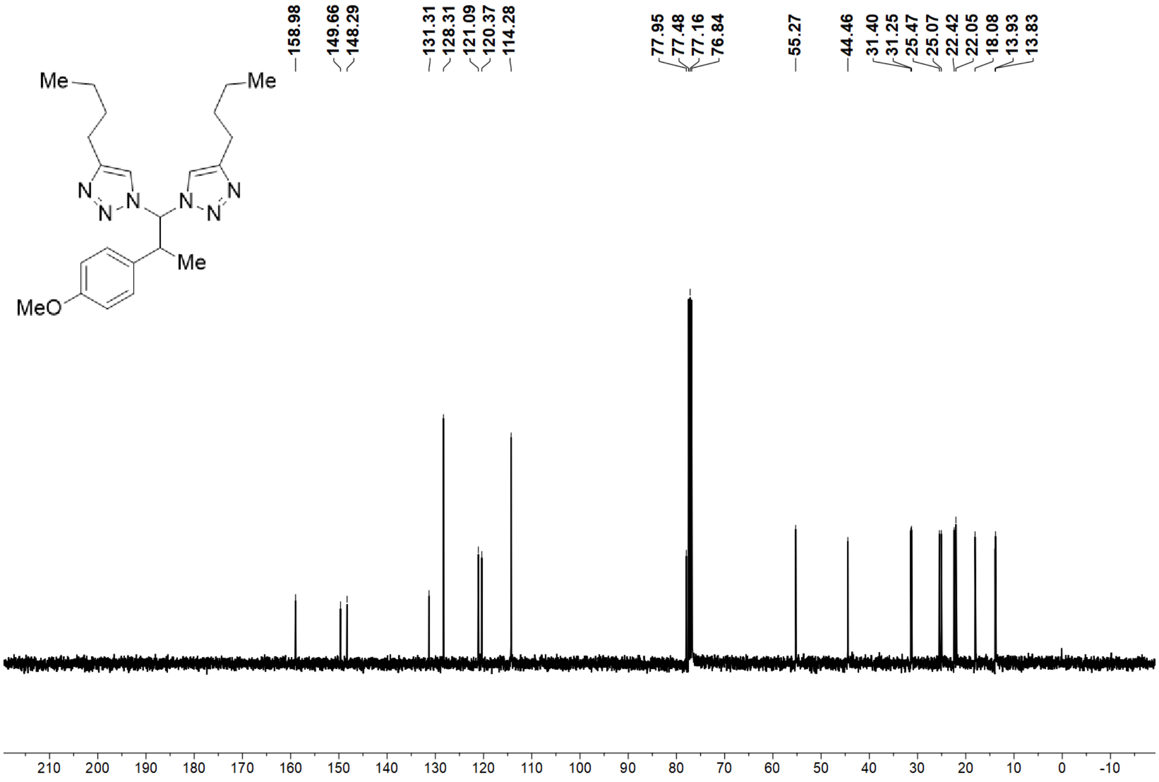


**Supplementary Figure 203.** $^{13}$C NMR (101 MHz, $CDCl_3$) spectrum of compound **4e**

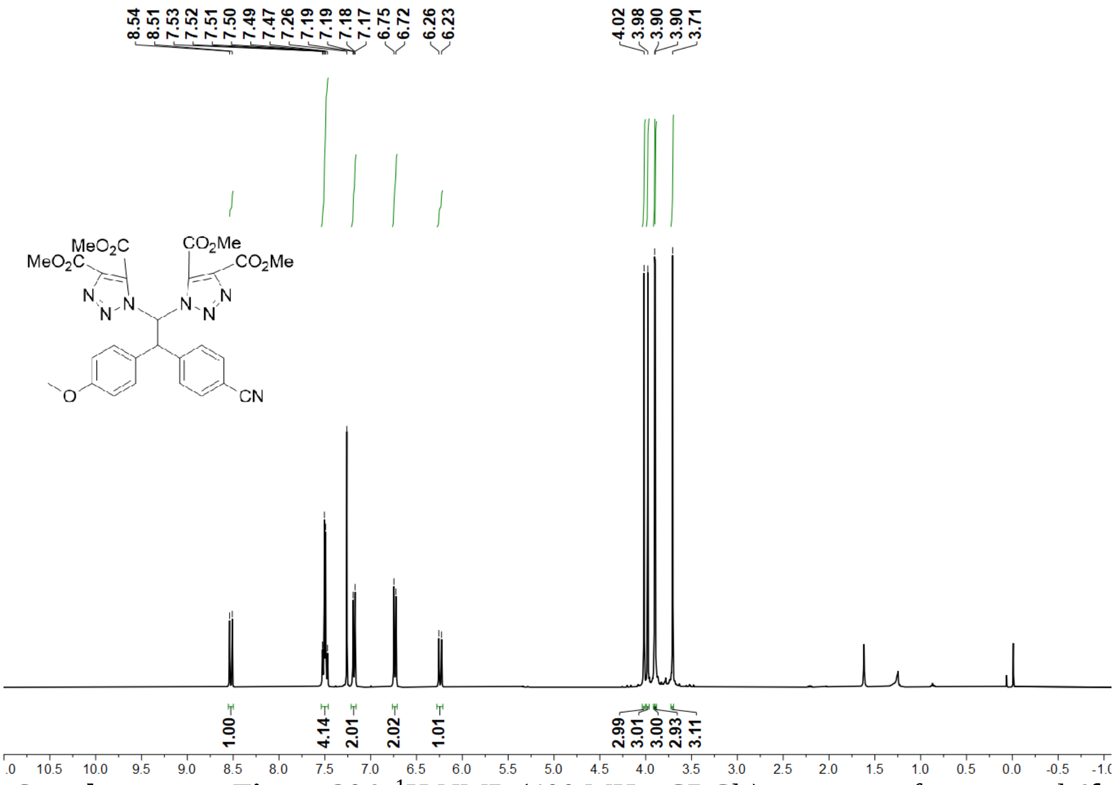

**Supplementary Figure 204.** $^{1}$H NMR (400 MHz, $CDCl_3$) spectrum of compound **4f**

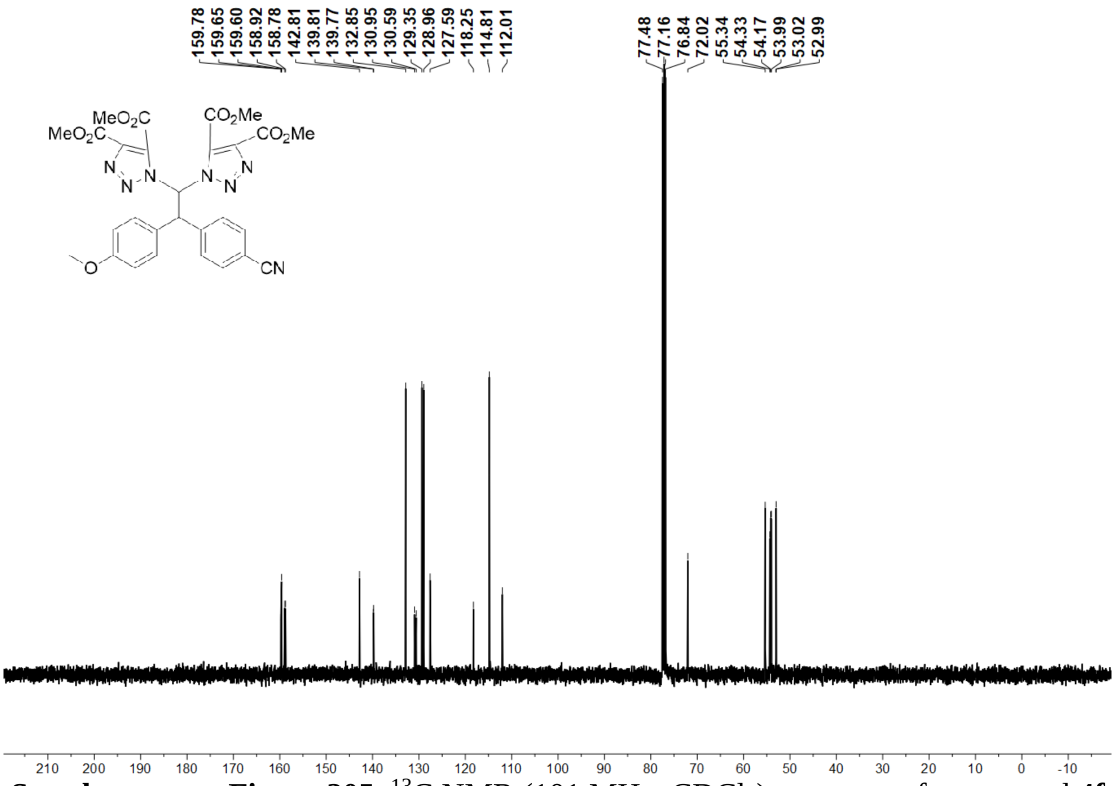

**Supplementary Figure 205.** $^{13}$C NMR (101 MHz, $CDCl_3$) spectrum of compound **4f**

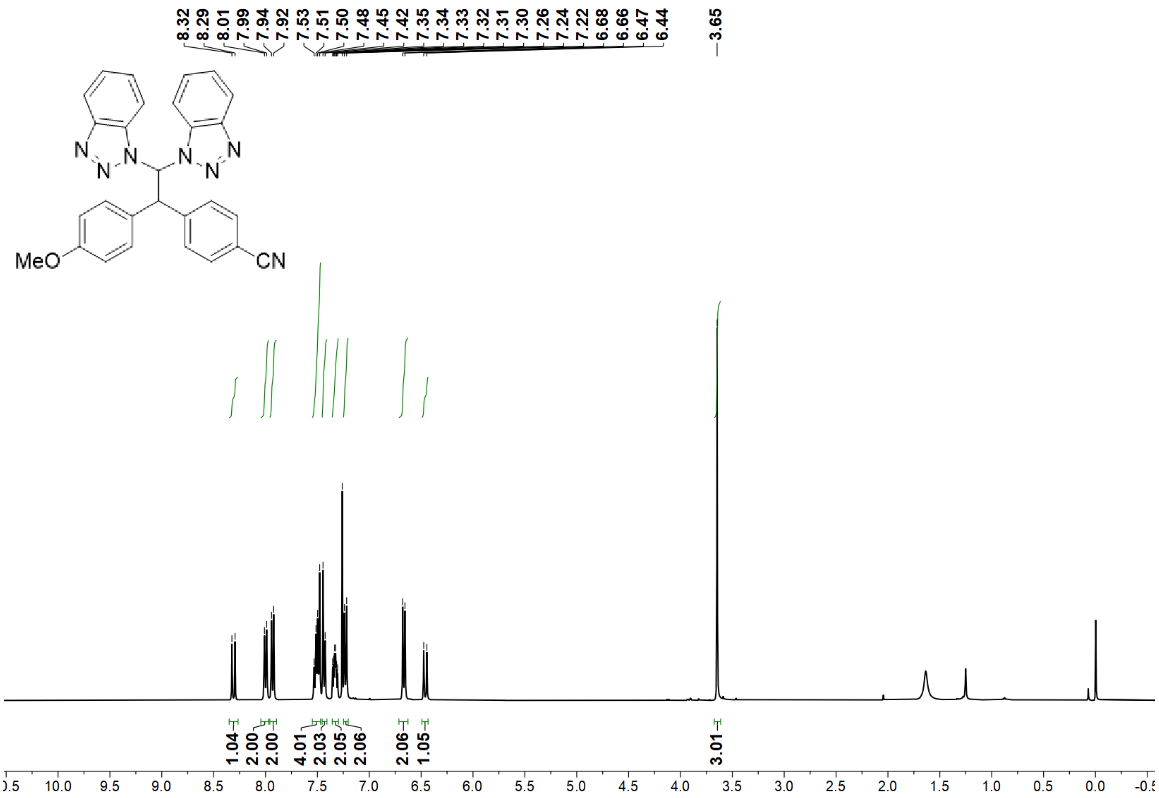

**Supplementary Figure 206.** $^{1}$H NMR (400 MHz, $CDCl_3$) spectrum of compound **4g**

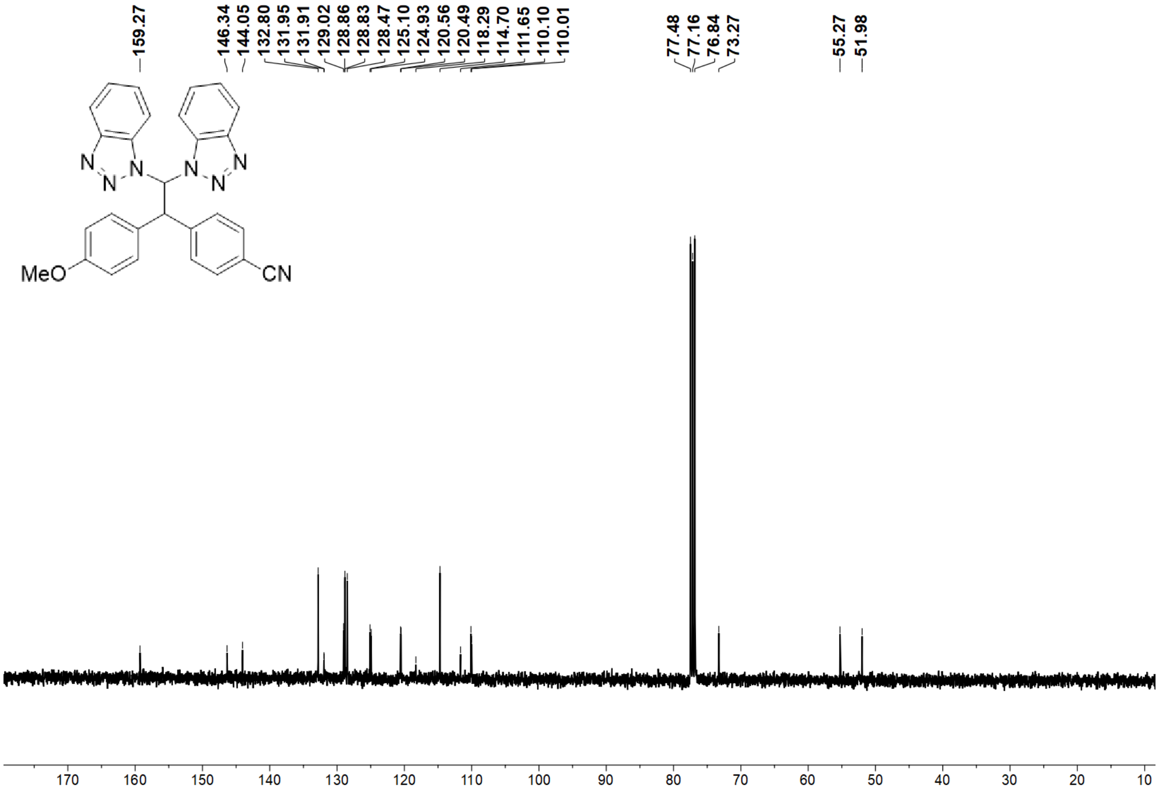

**Supplementary Figure 207.** $^{13}$C NMR (101 MHz, $CDCl_3$) spectrum of compound **4g**

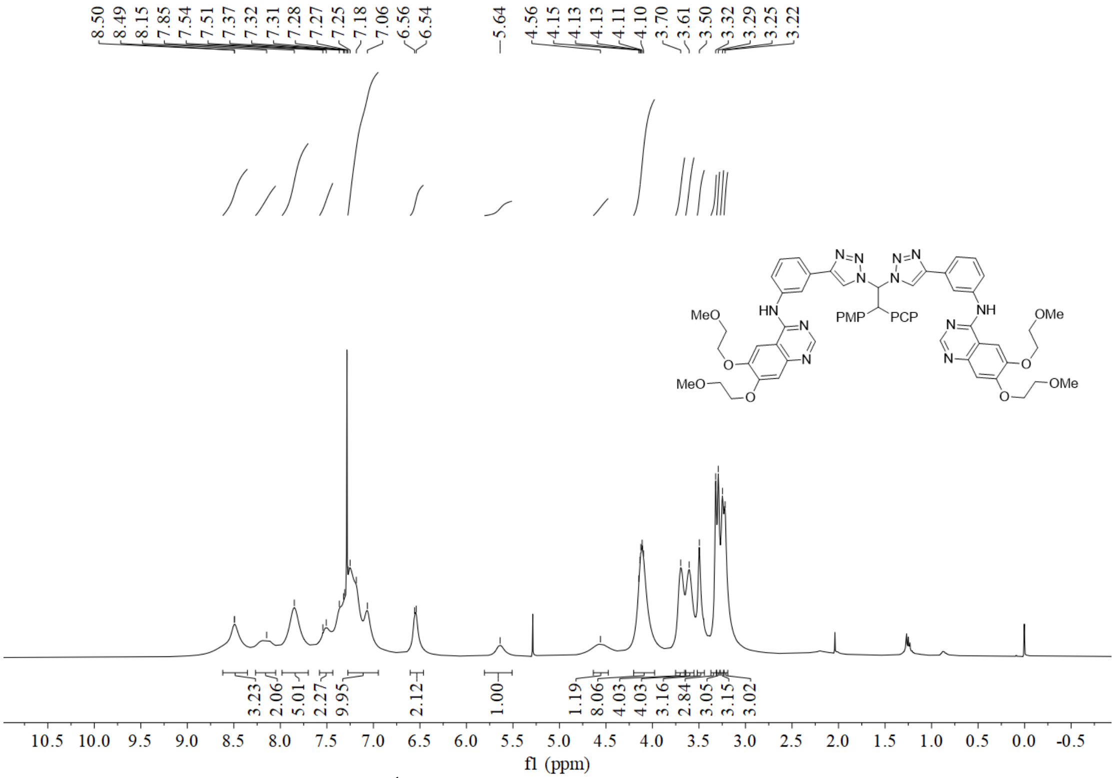


**Supplementary Figure 208.** $^{1}H$ NMR (400 MHz, $CDCl_3$) spectrum of compound **4h**

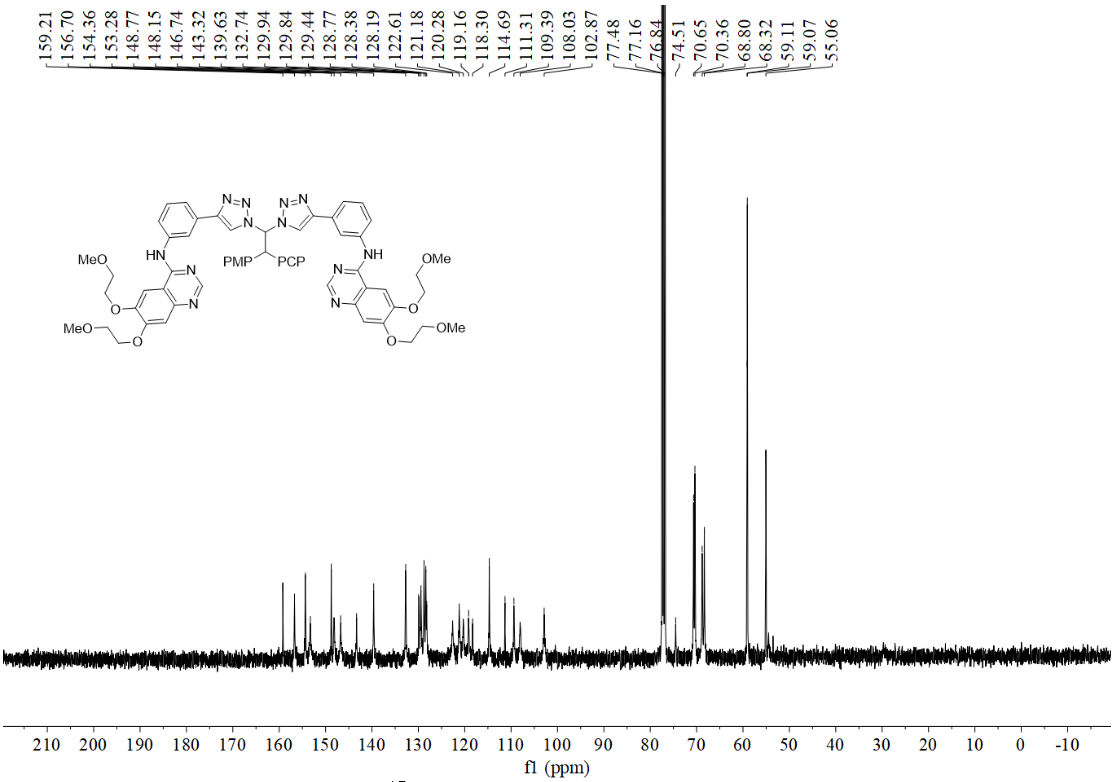


**Supplementary Figure 209.** $^{13}C$ NMR (101 MHz, $CDCl_3$) spectrum of compound **4h**

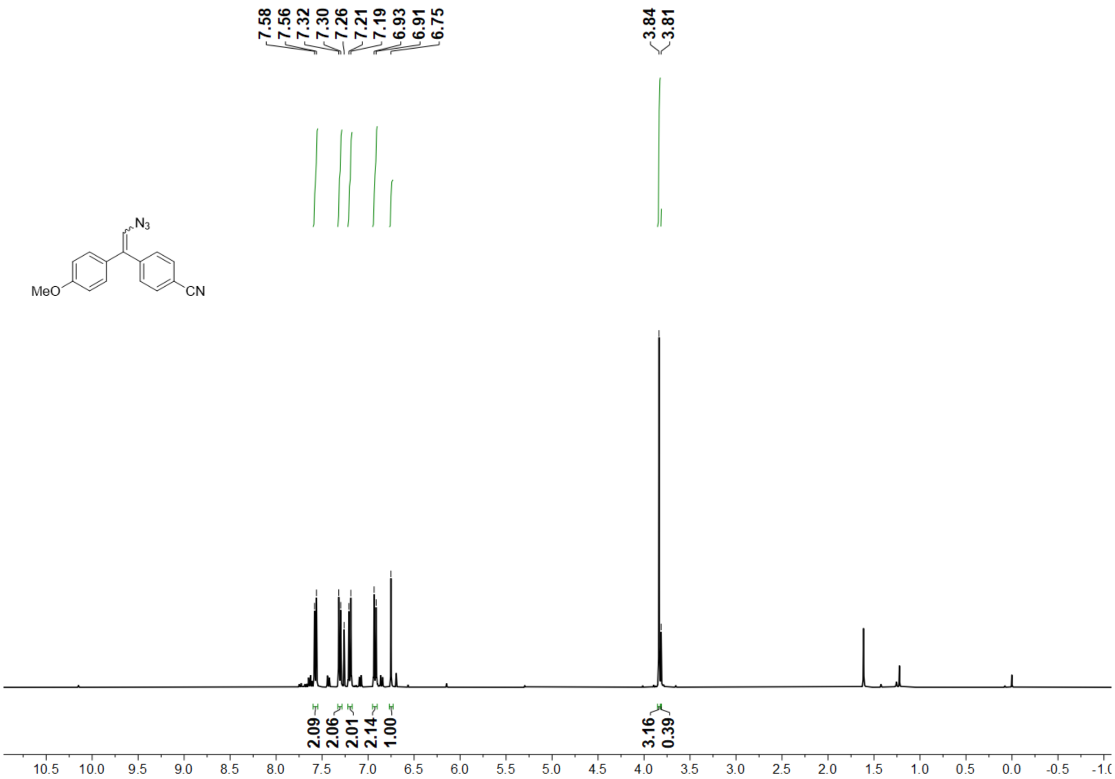


**Supplementary Figure 210.** $^{1}H$ NMR (400 MHz, $CDCl_3$) spectrum of compound **4i**

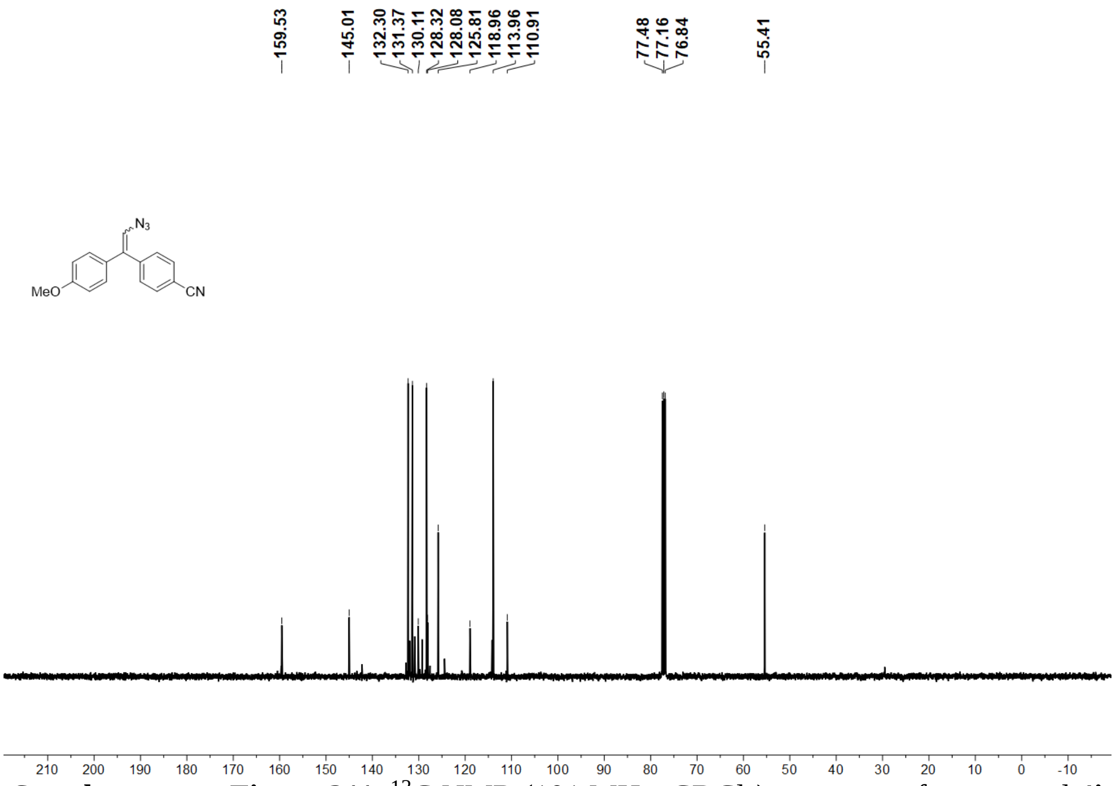


**Supplementary Figure 211.** $^{13}C$ NMR (101 MHz, $CDCl_3$) spectrum of compound **4i**

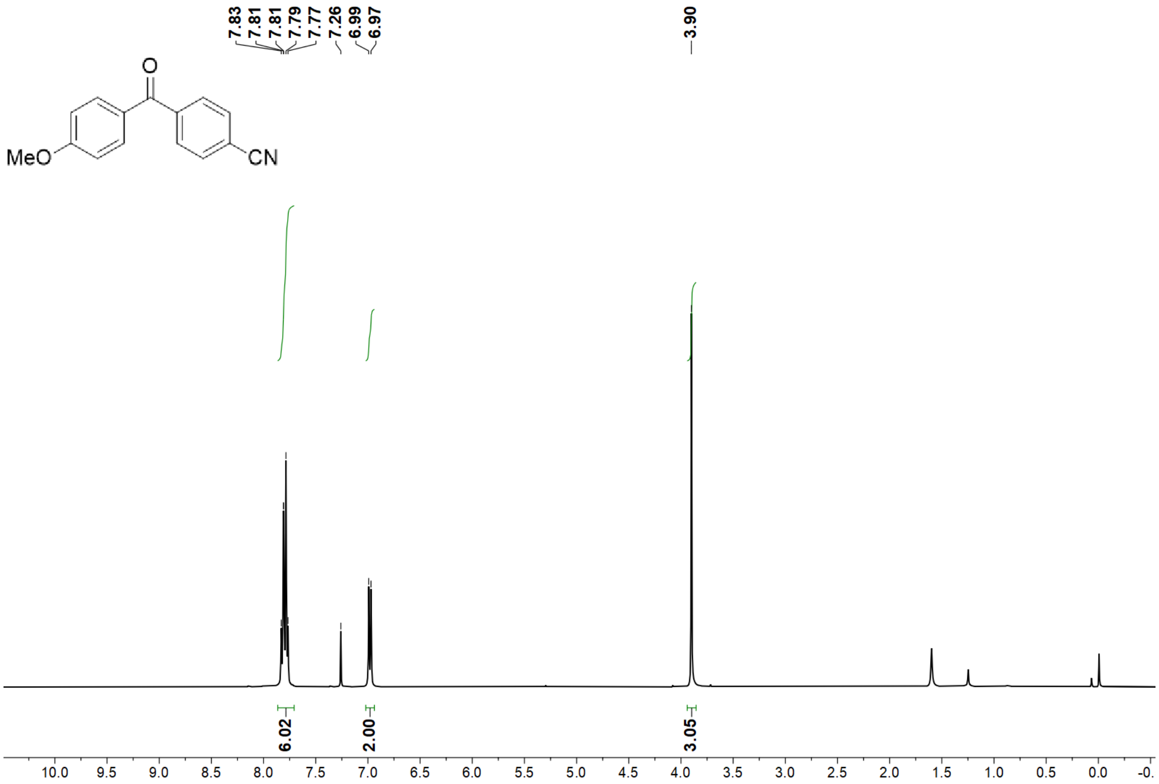


**Supplementary Figure 212.** $^{1}H$ NMR (400 MHz, $CDCl_3$) spectrum of compound **4j**

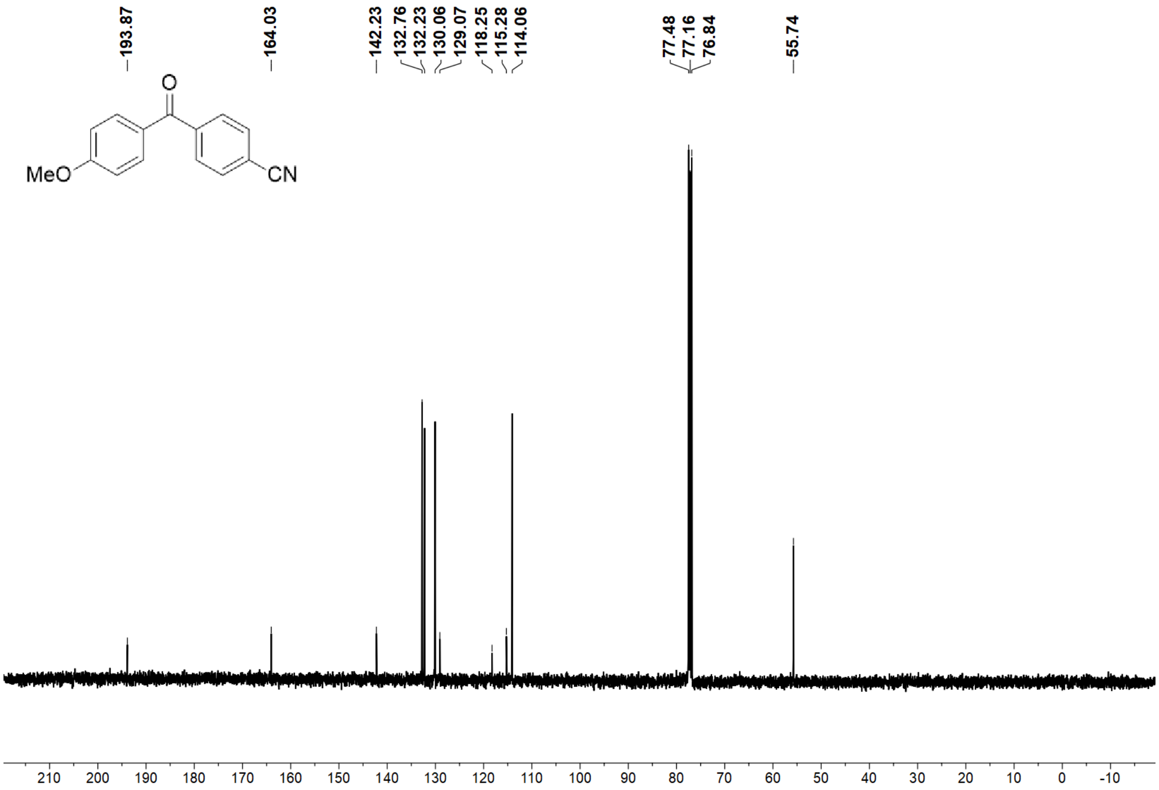


**Supplementary Figure 213.** $^{13}C$ NMR (101 MHz, $CDCl_3$) spectrum of compound **4j**

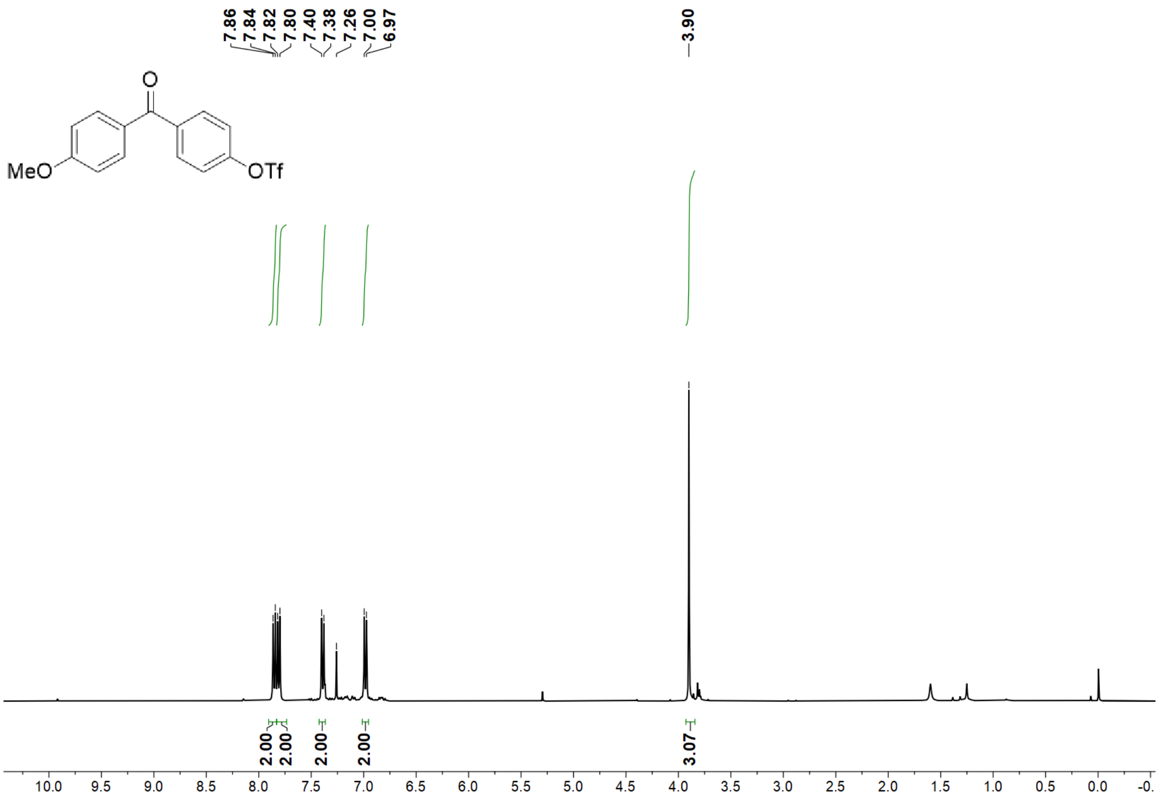


**Supplementary Figure 214.** $^{1}$H NMR (400 MHz, $CDCl_3$) spectrum of compound **4k**

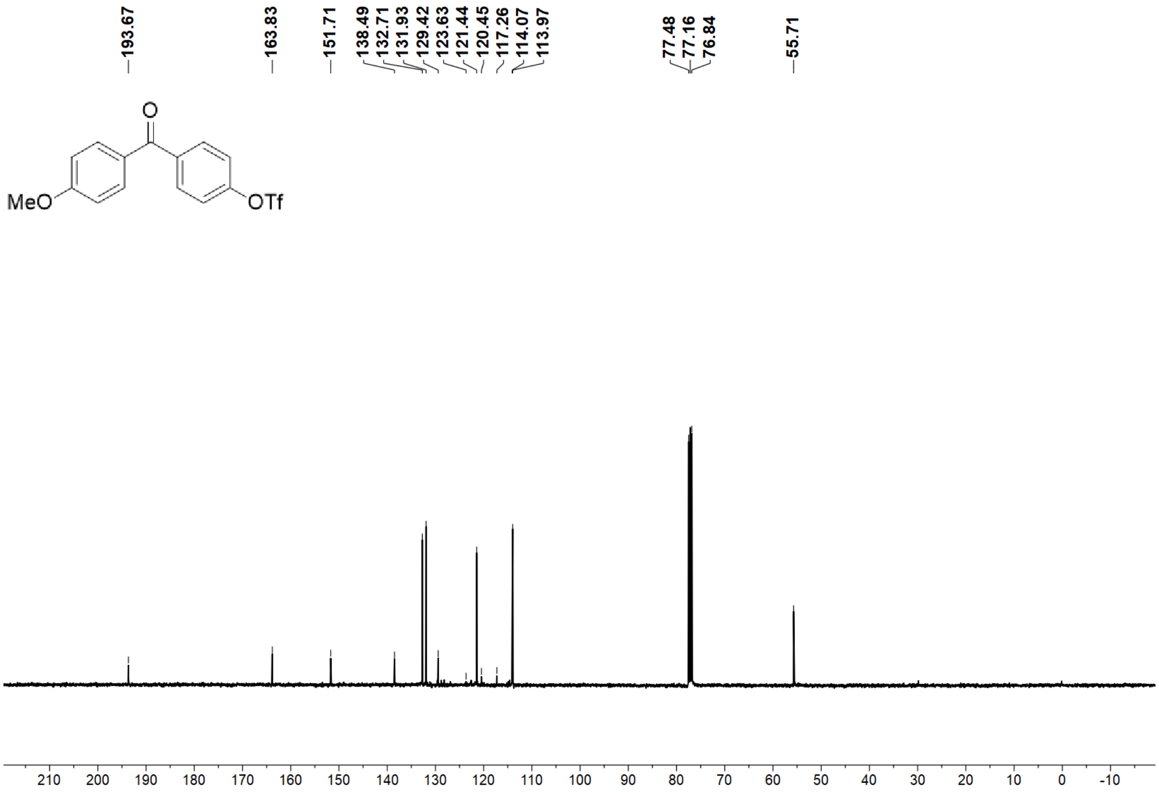


**Supplementary Figure 215.** $^{13}$C NMR (101 MHz, $CDCl_3$) spectrum of compound **4k**

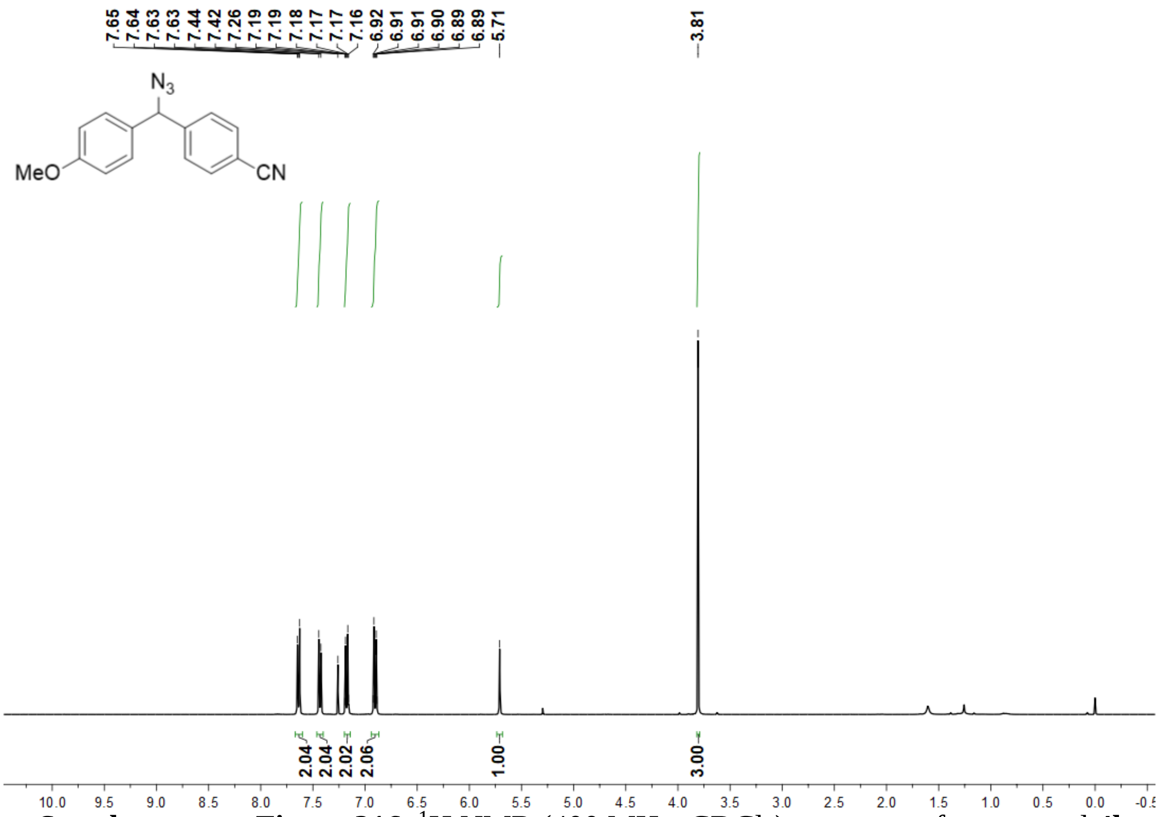


**Supplementary Figure 216.** $^{1}$H NMR (400 MHz, $CDCl_3$) spectrum of compound **4l**

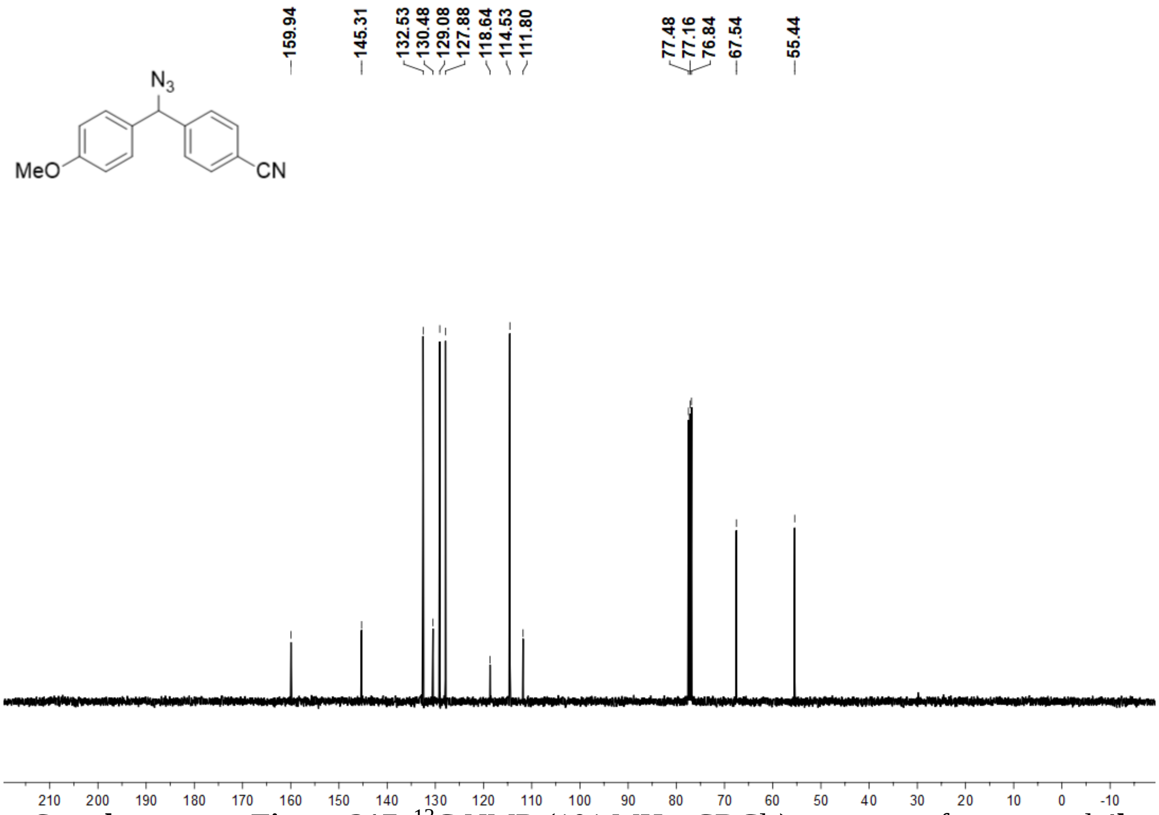


**Supplementary Figure 217.** $^{13}$C NMR (101 MHz, $CDCl_3$) spectrum of compound **4l**

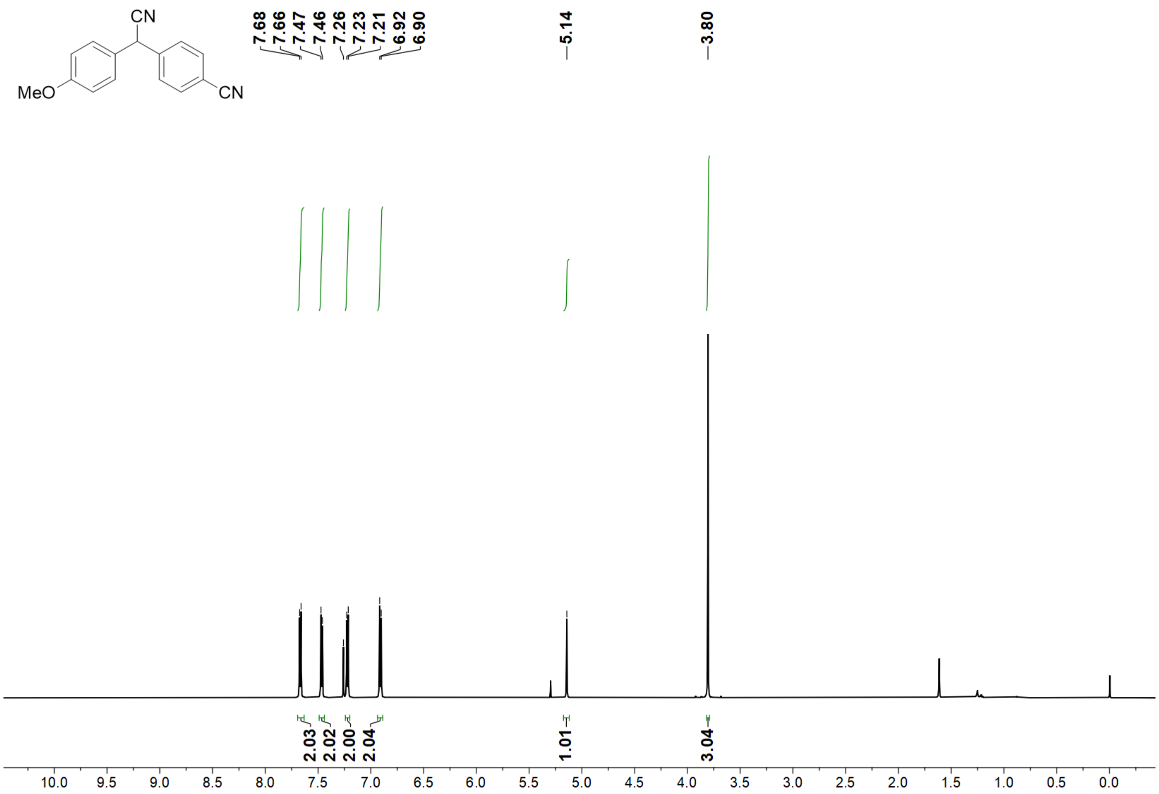


**Supplementary Figure 218.** $^{1}H$ NMR (600 MHz, $CDCl_3$) spectrum of compound **4m**

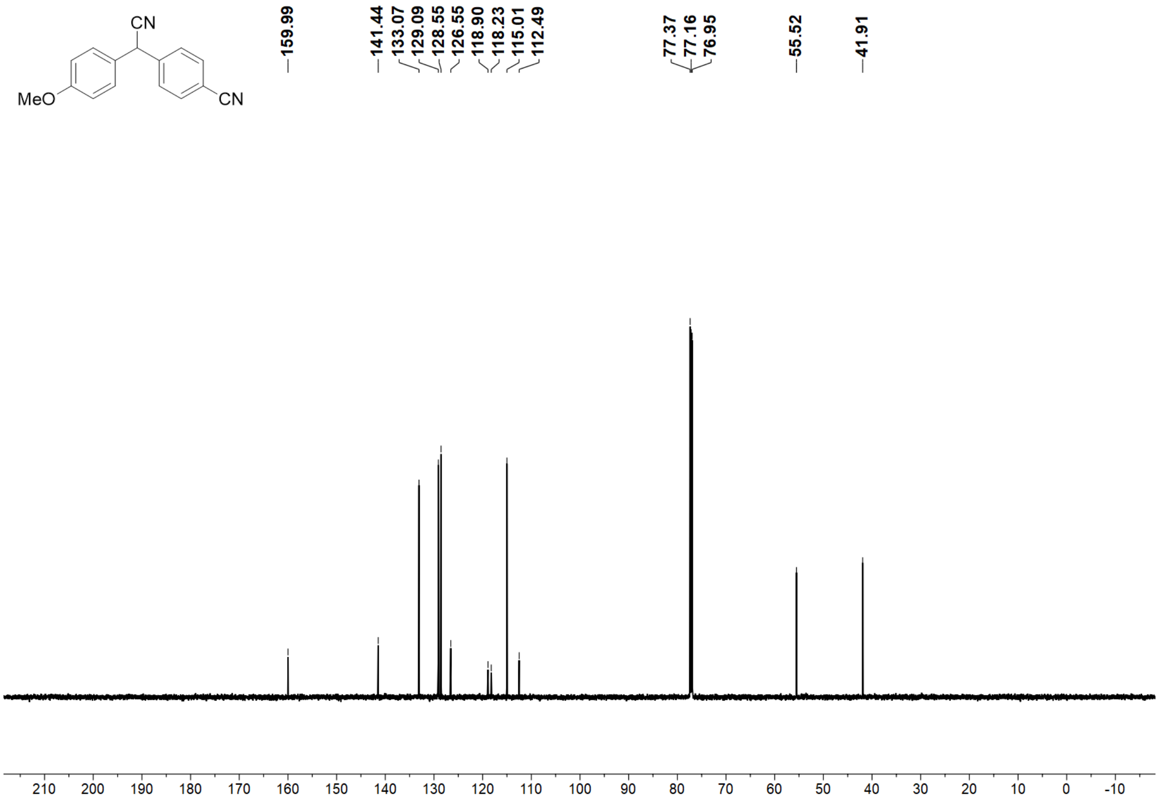


**Supplementary Figure 219.** $^{13}C$ NMR (151 MHz, $CDCl_3$) spectrum of compound **4m**

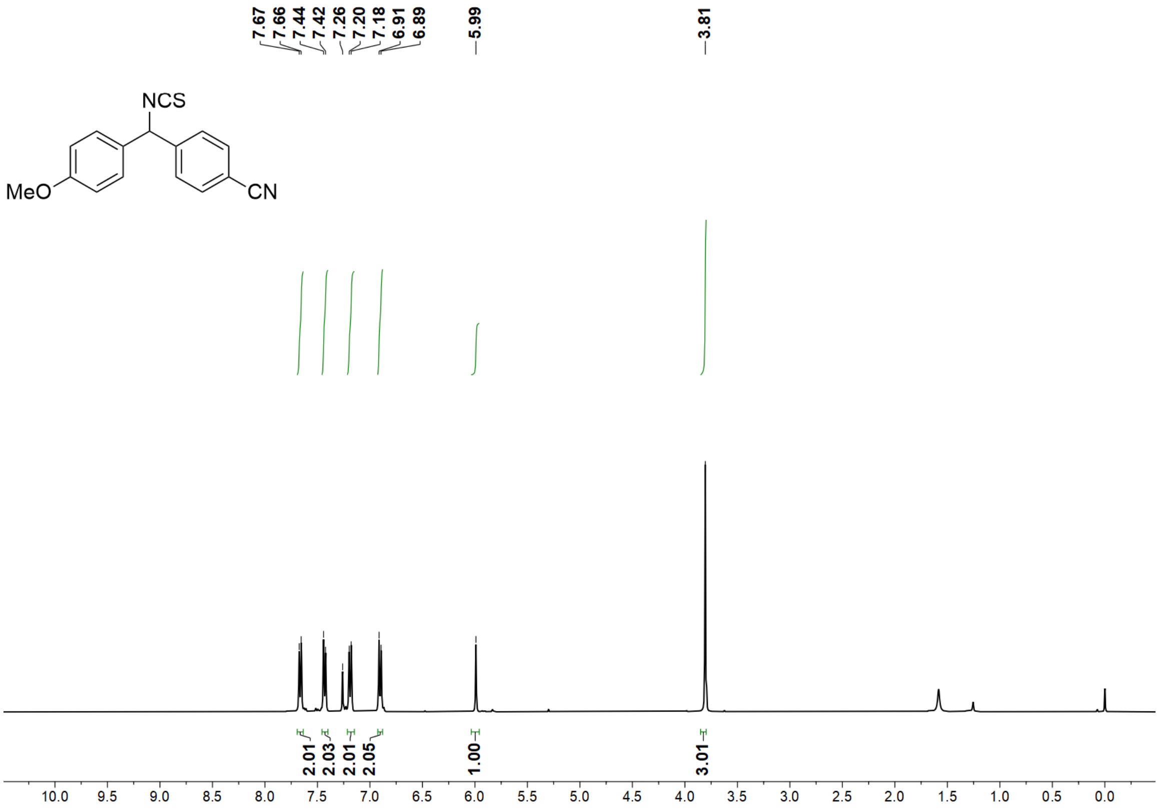


**Supplementary Figure 220.** $^{1}$H NMR (400 MHz, $CDCl_3$) spectrum of compound **4n**

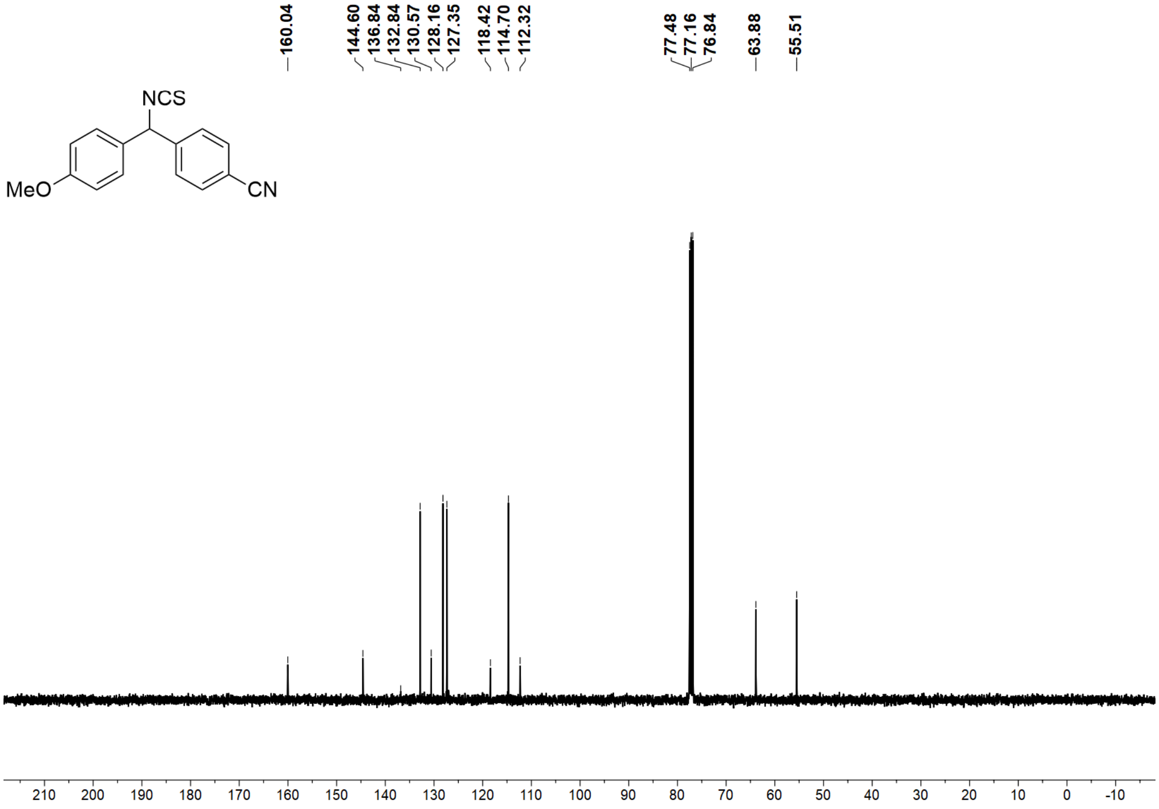


**Supplementary Figure 221.** $^{13}$C NMR (101 MHz, $CDCl_3$) spectrum of compound **4n**

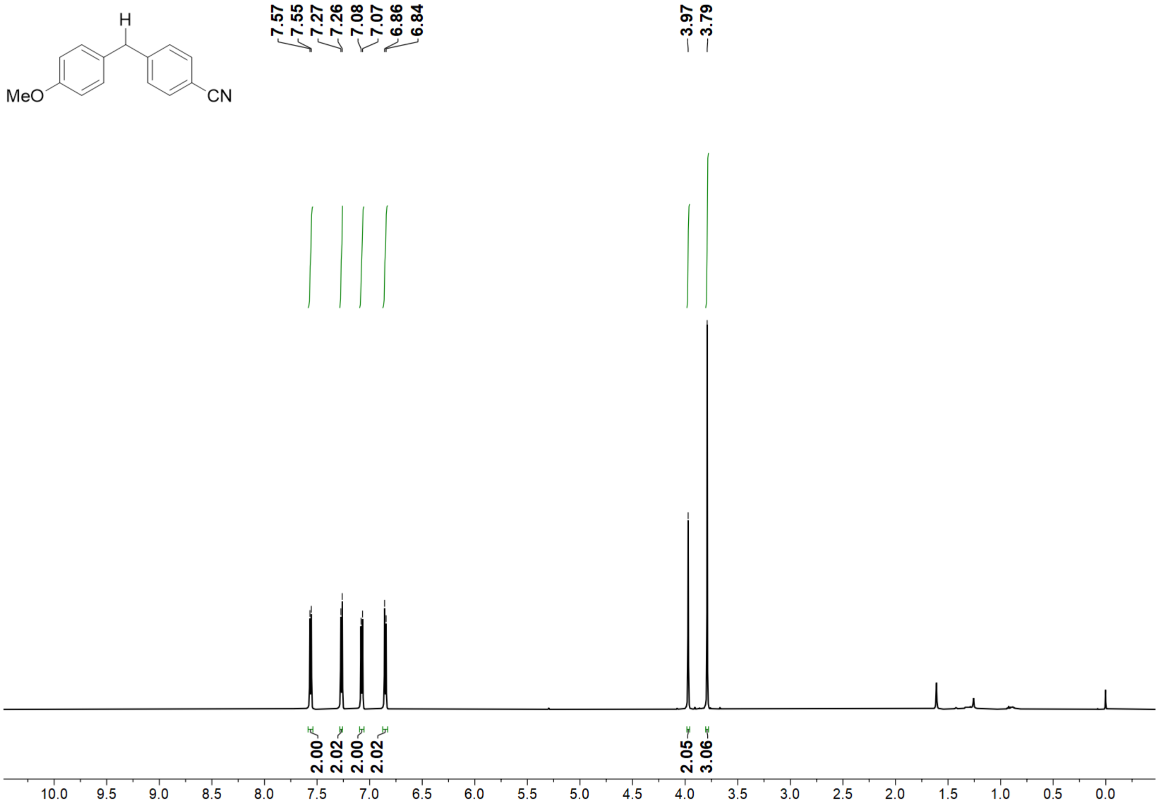


**Supplementary Figure 222.** $^{1}H$ NMR (600 MHz, $CDCl_3$) spectrum of compound **4o**

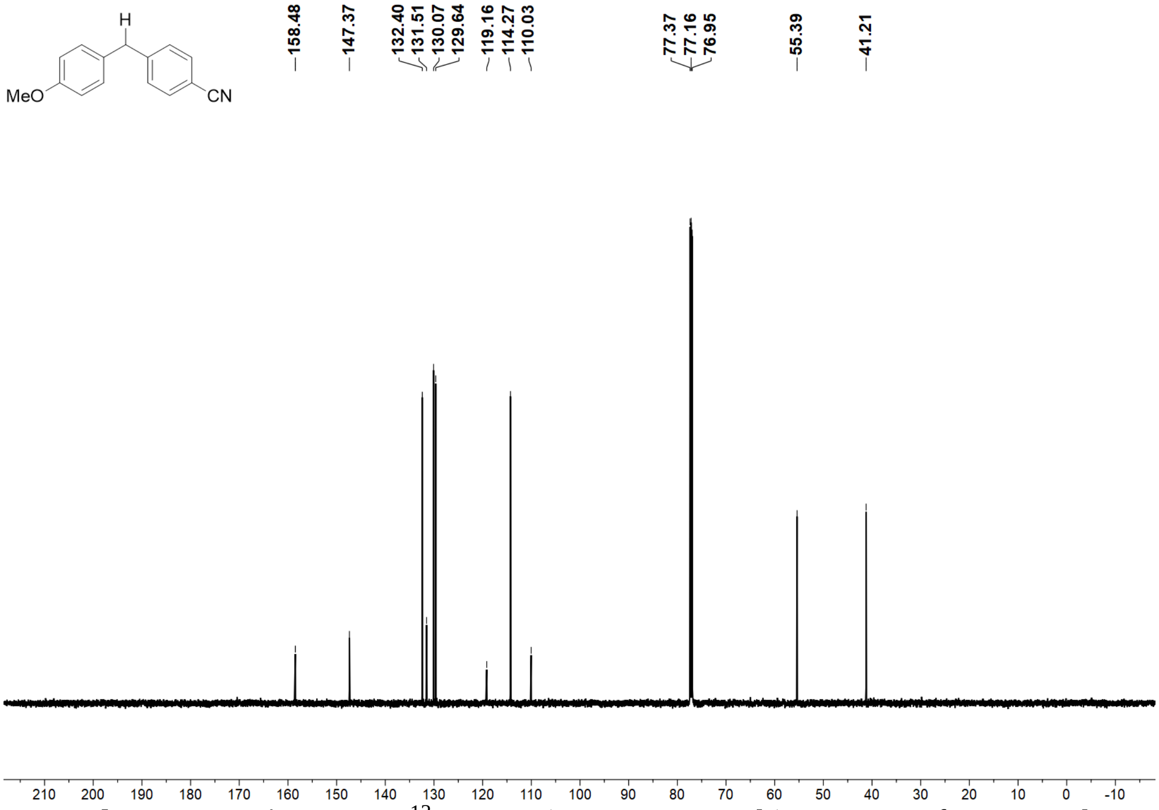


**Supplementary Figure 223.** $^{13}C$ NMR (151 MHz, $CDCl_3$) spectrum of compound **4o**

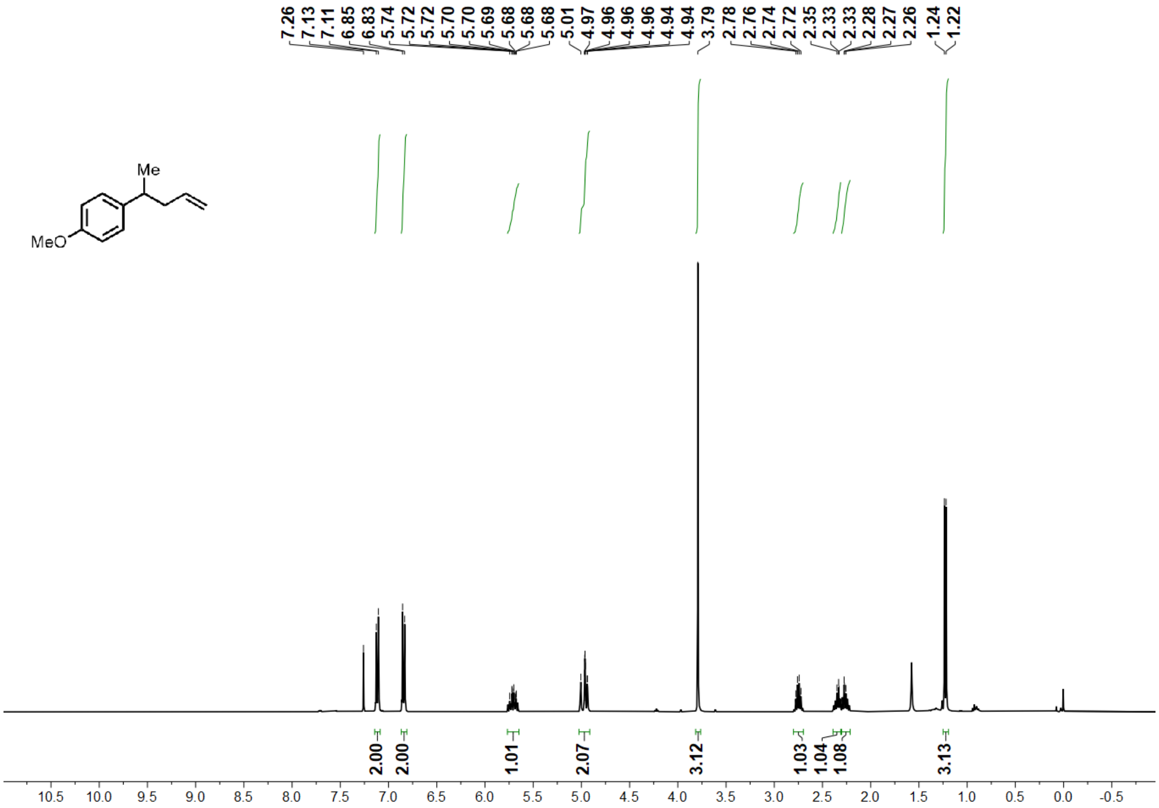

**Supplementary Figure 224.** $^{1}$H NMR (400 MHz, $CDCl_3$) spectrum of compound **4p**

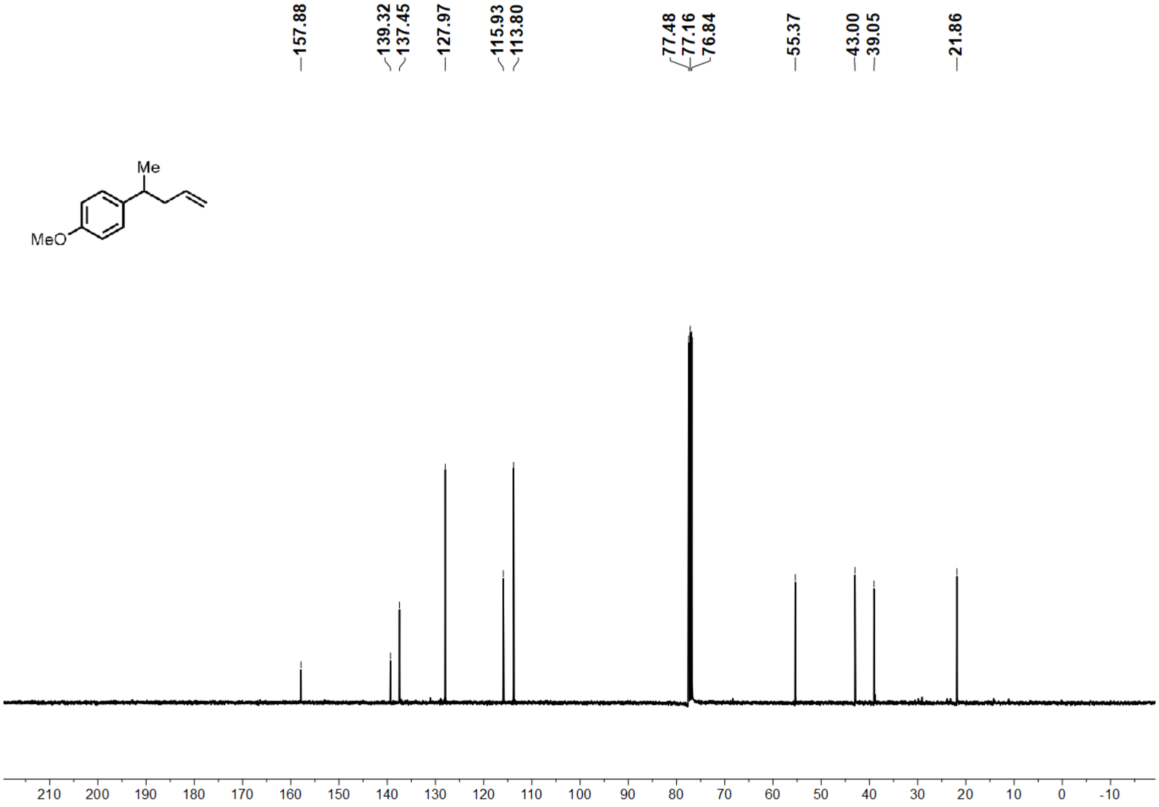

**Supplementary Figure 225.** $^{13}$C NMR (101 MHz, $CDCl_3$) spectrum of compound **4p**

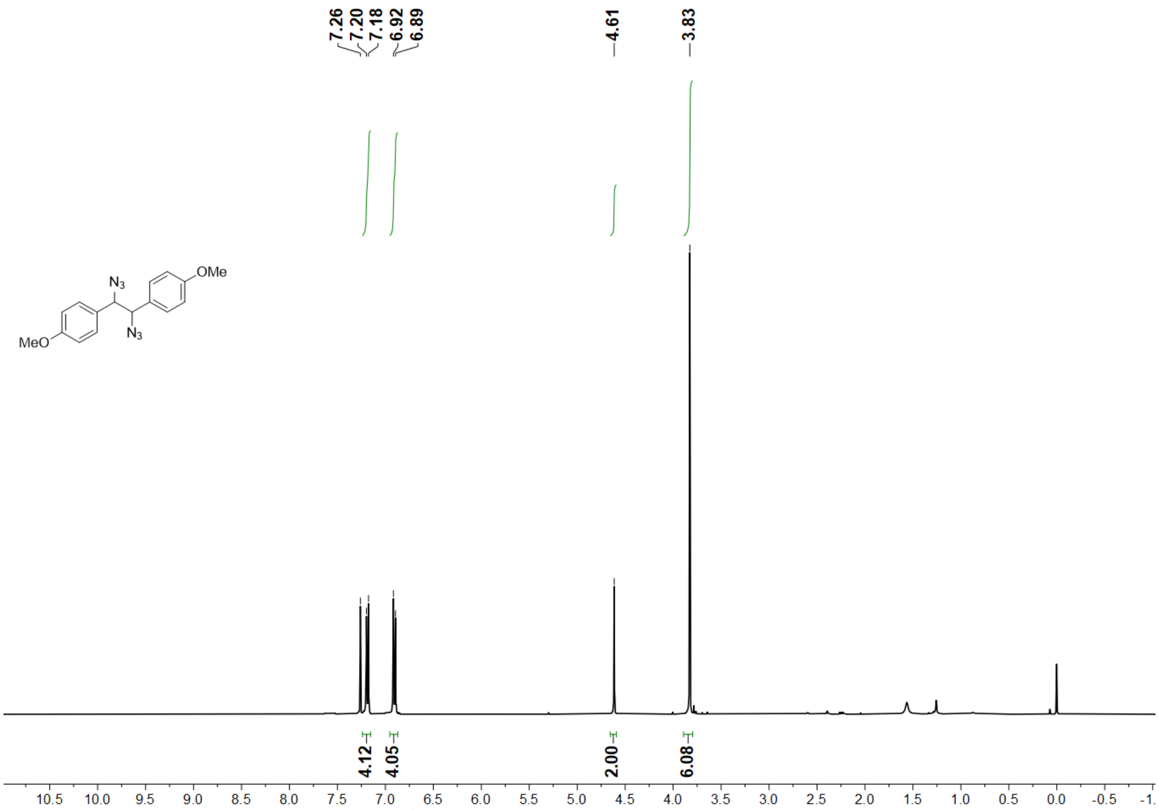


**Supplementary Figure 226.** $^{1}H$ NMR (400 MHz, $CDCl_3$) spectrum of compound **3be**

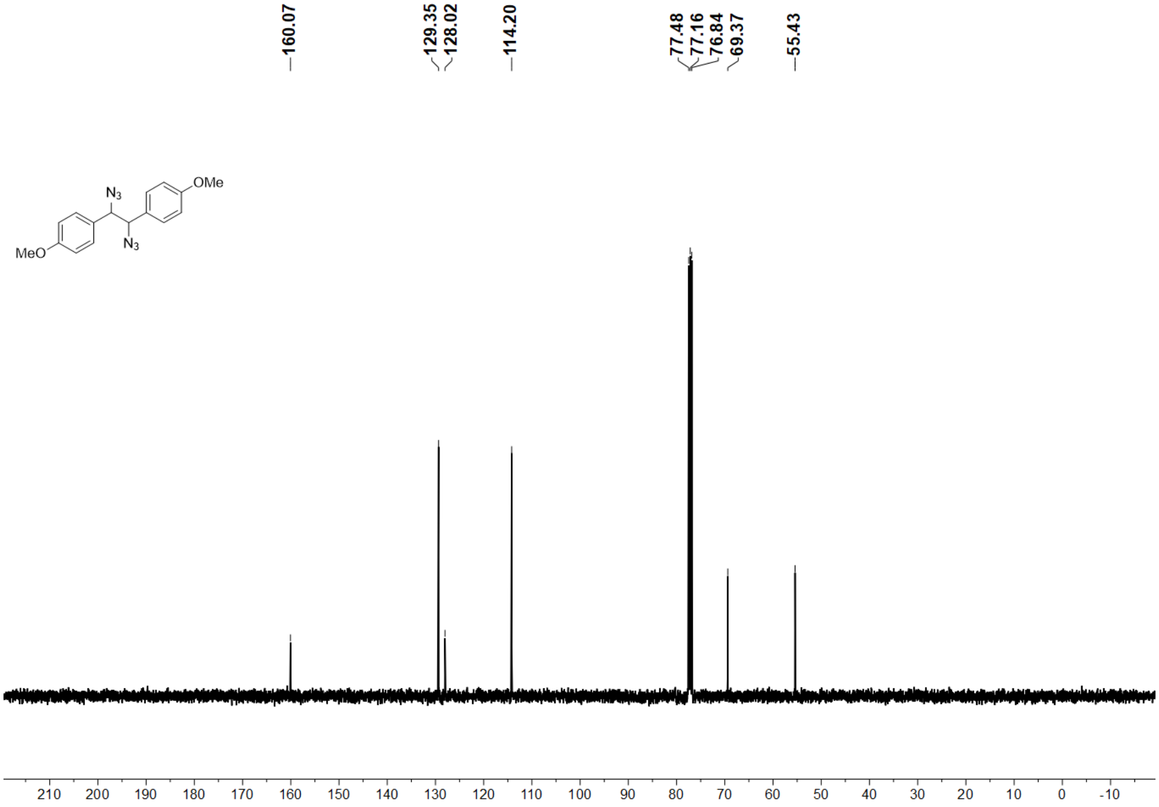


**Supplementary Figure 227.** $^{13}C$ NMR (101 MHz, $CDCl_3$) spectrum of compound **3be**

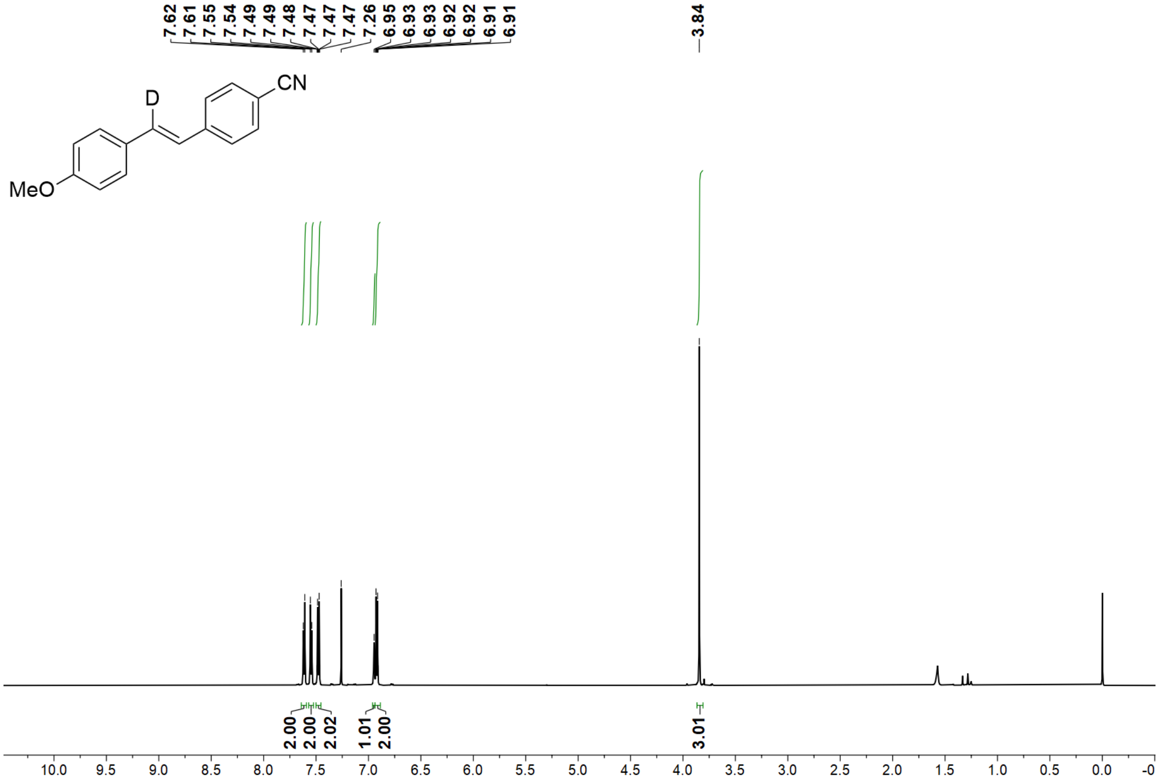


**Supplementary Figure 228.** $^{1}$H NMR (600 MHz, $CDCl_3$) spectrum of compound **1a-D**

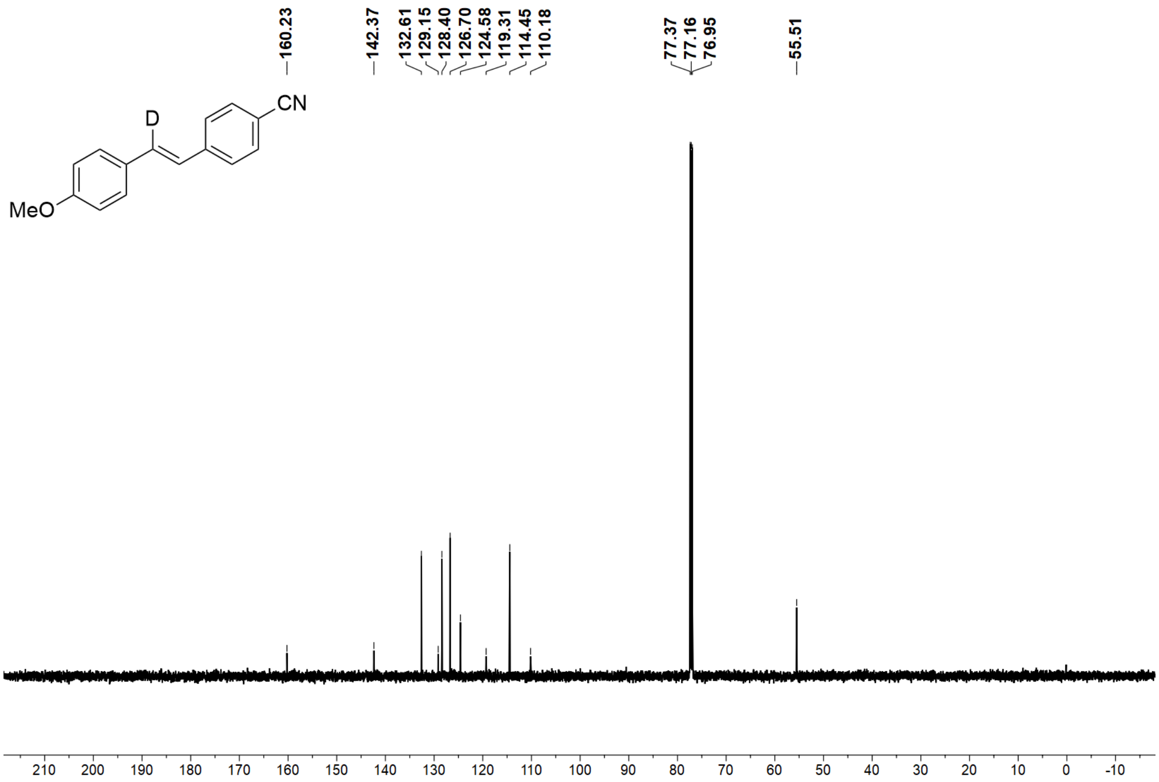


**Supplementary Figure 229.** $^{13}$C NMR (151 MHz, $CDCl_3$) spectrum of compound **1a-D**

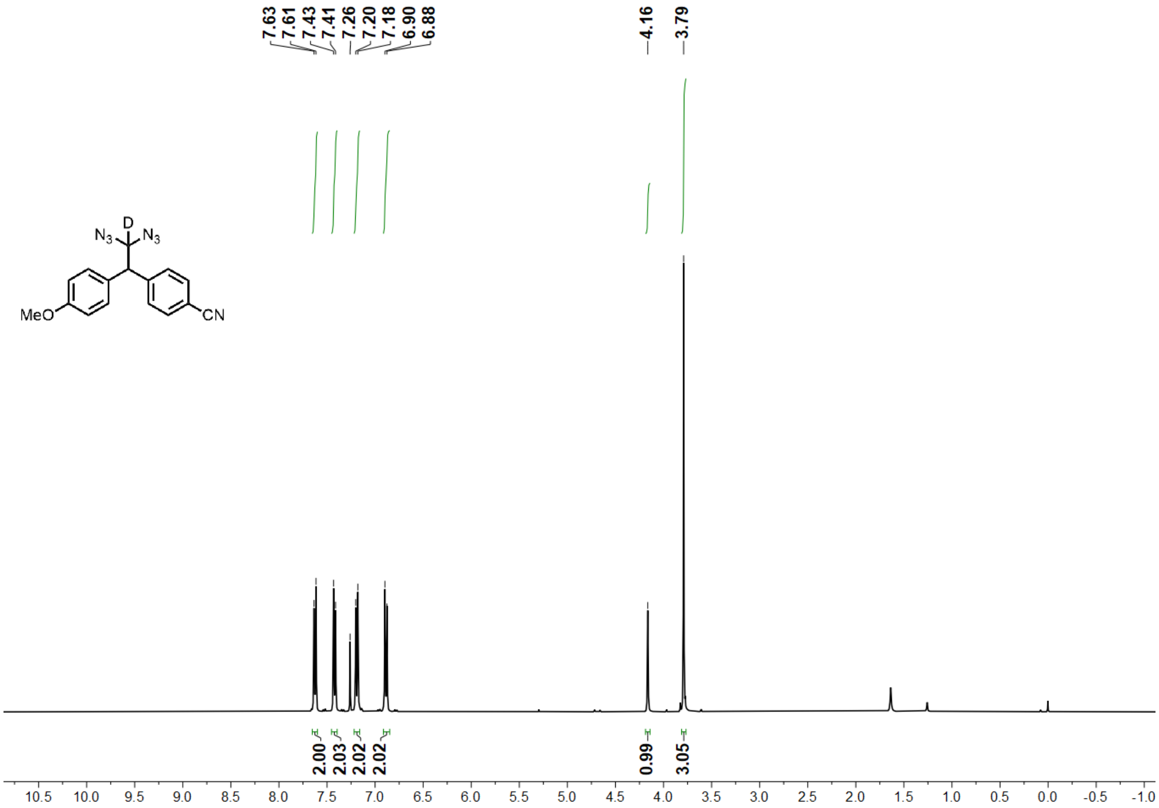


**Supplementary Figure 230.** $^{1}$H NMR (400 MHz, $CDCl_3$) spectrum of compound **2a-D**

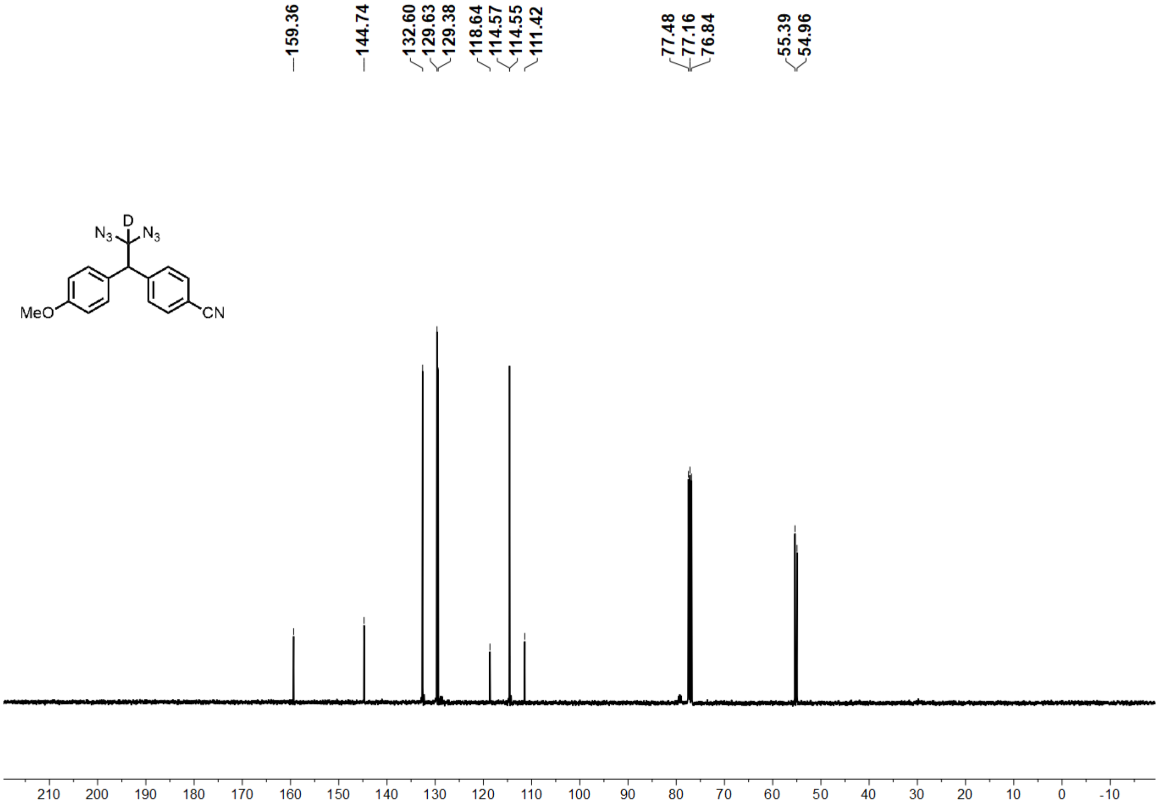


**Supplementary Figure 231.** $^{13}$C NMR (101 MHz, $CDCl_3$) spectrum of compound **2a-D**

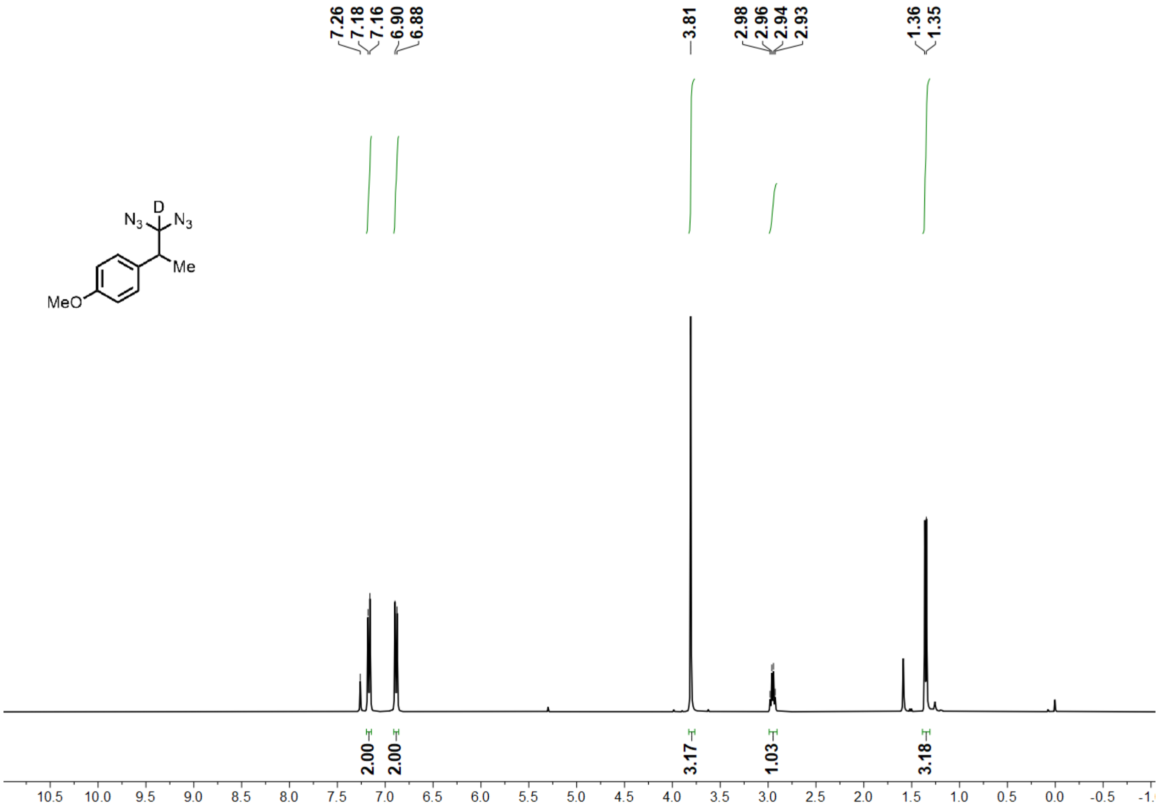


**Supplementary Figure 232.** $^{1}$H NMR (400 MHz, $CDCl_3$) spectrum of compound **2at-D**

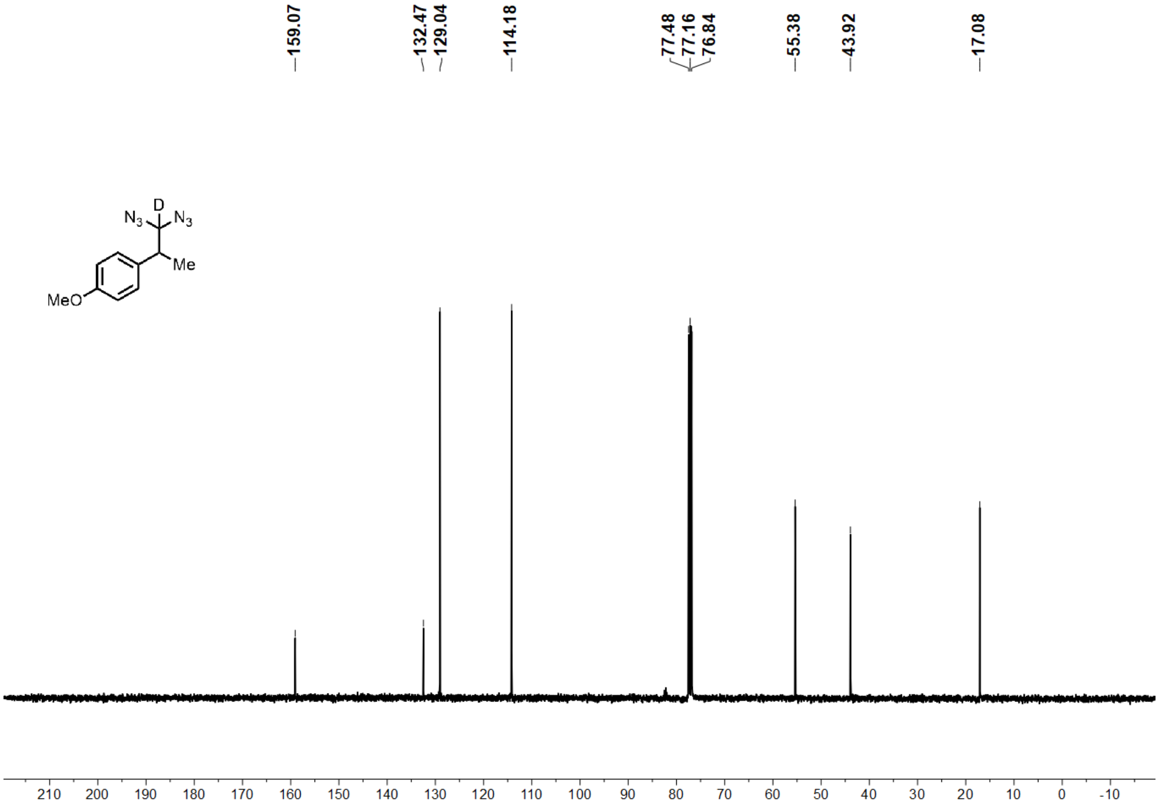


**Supplementary Figure 233.** $^{13}$C NMR (101 MHz, $CDCl_3$) spectrum of compound **2at-D**

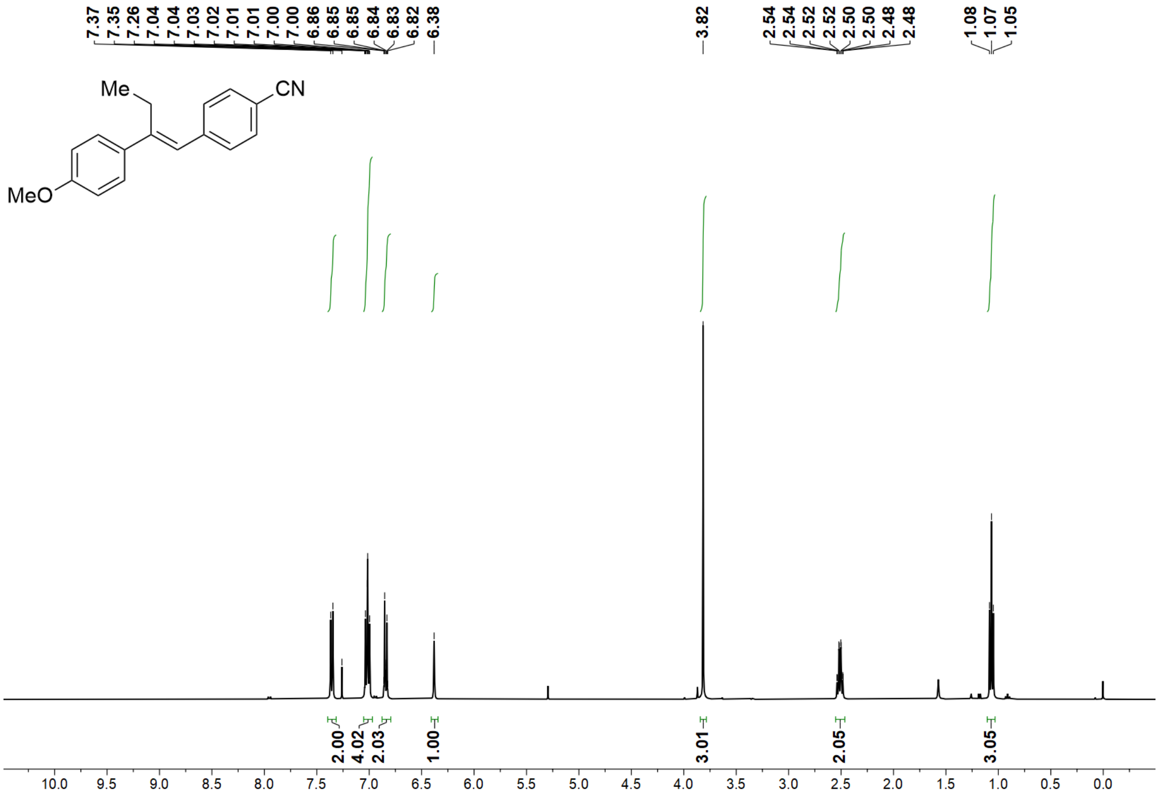


**Supplementary Figure 234.** $^{1}$H NMR (400 MHz, $CDCl_3$) spectrum of compound **1bj**

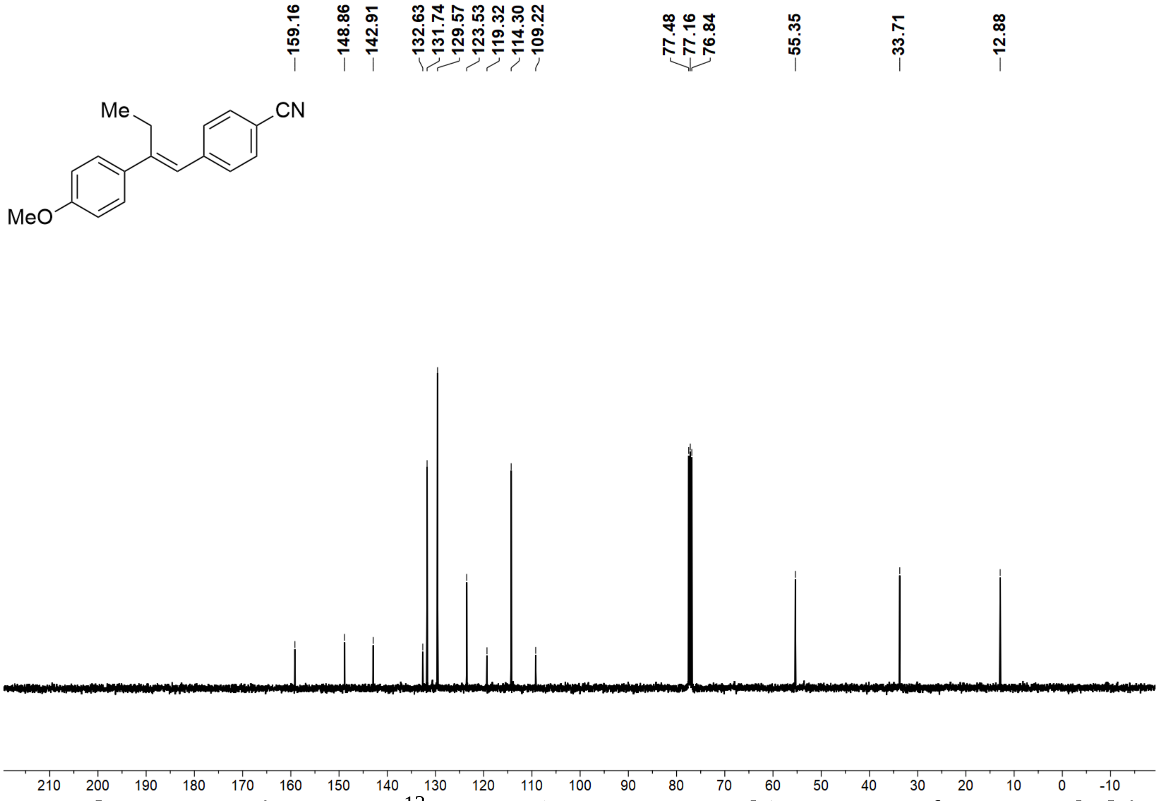


**Supplementary Figure 235.** $^{13}$C NMR (101 MHz, $CDCl_3$) spectrum of compound **1bj**

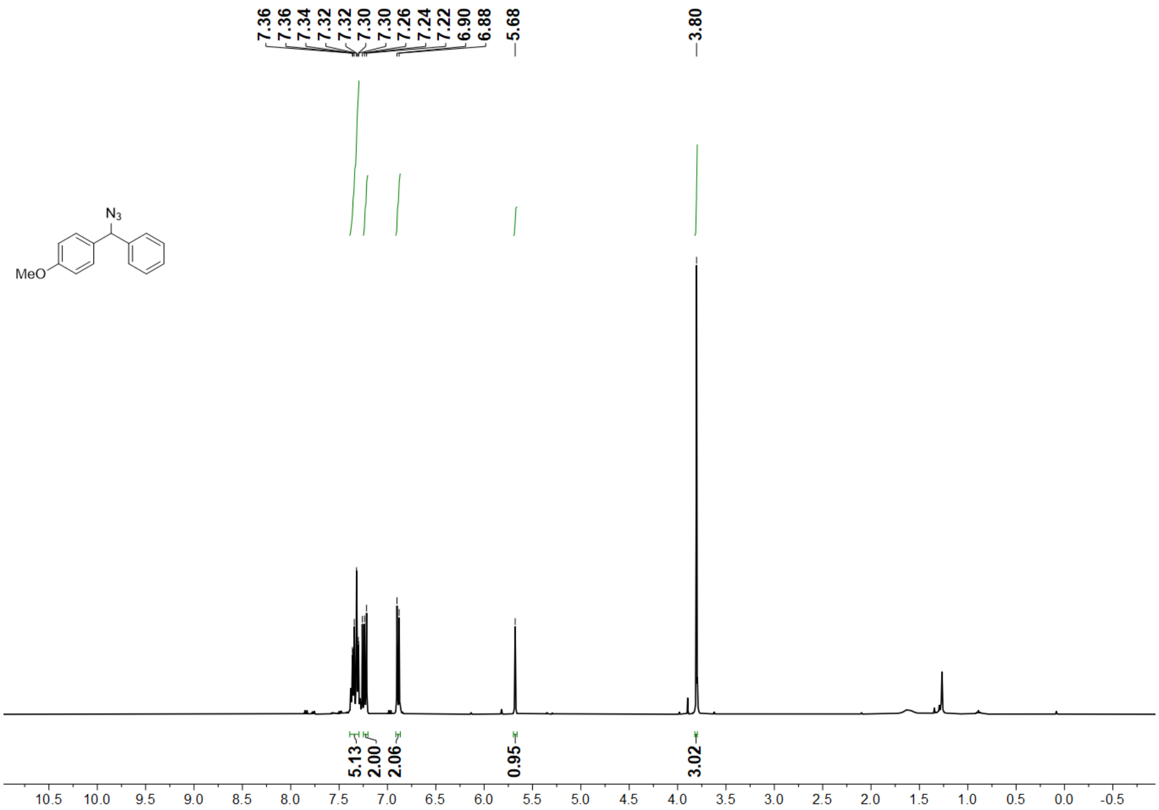


**Supplementary Figure 236.** $^{1}$H NMR (400 MHz, $CDCl_3$) spectrum of compound **5**

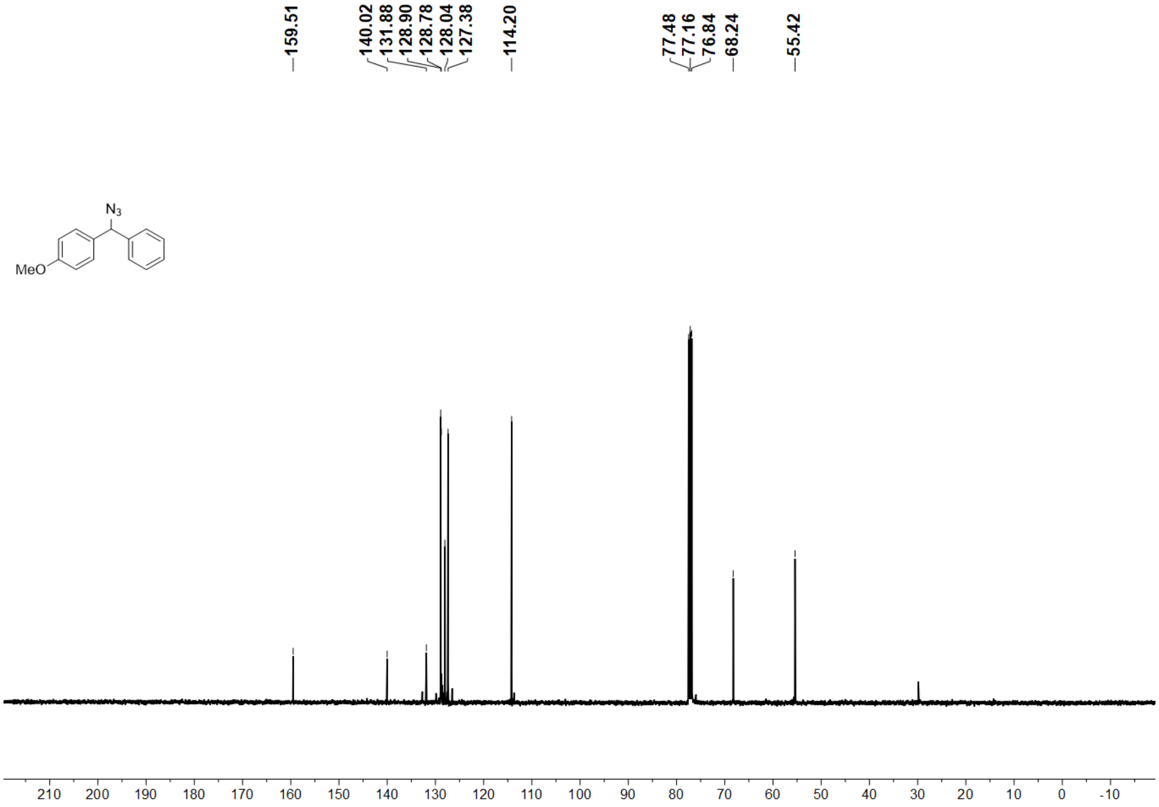


**Supplementary Figure 237.** $^{13}$C NMR (101 MHz, $CDCl_3$) spectrum of compound **5**

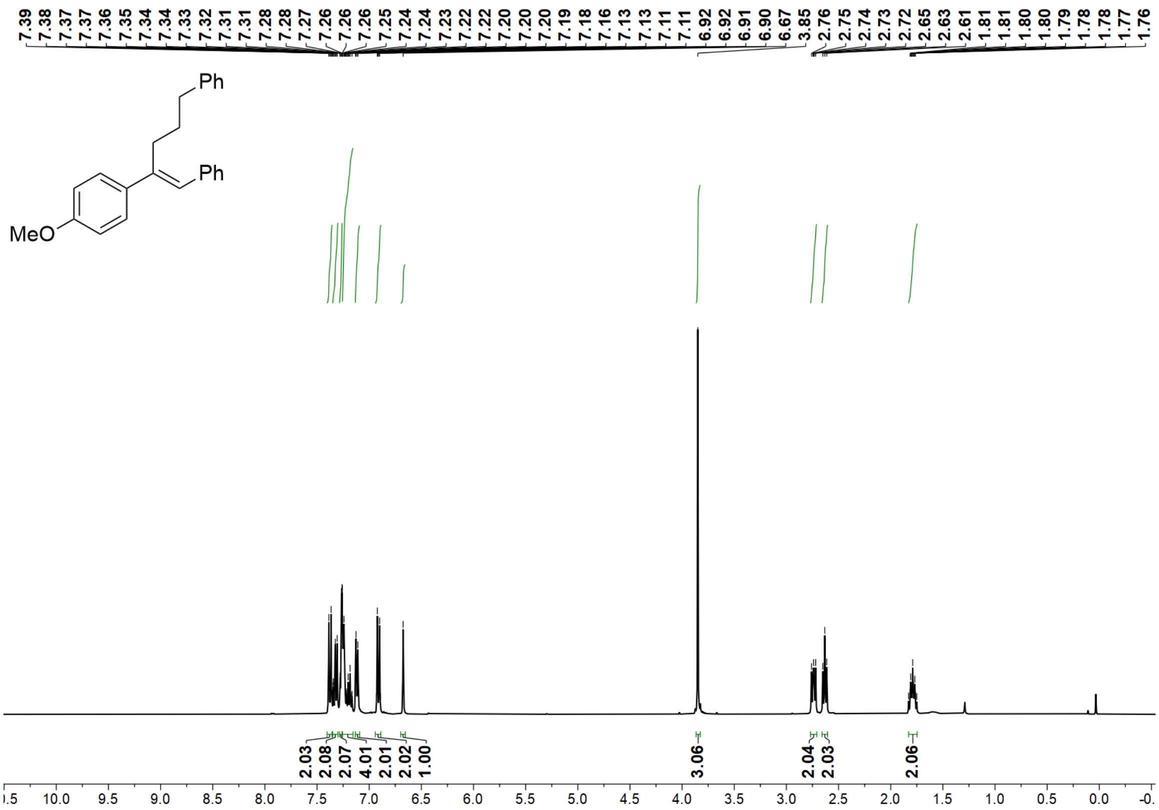


**Supplementary Figure 238.** $^{1}$H NMR (400 MHz, $CDCl_3$) spectrum of compound **1bl**

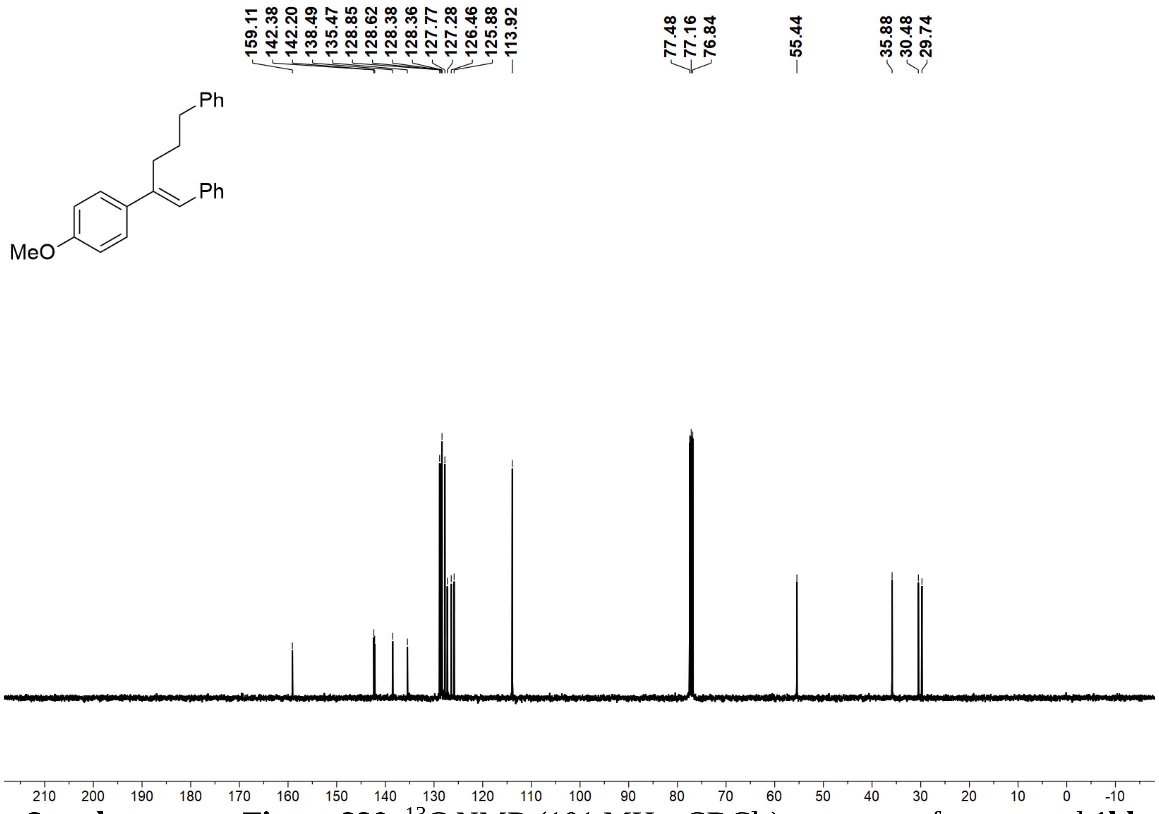


**Supplementary Figure 239.** $^{13}$C NMR (101 MHz, $CDCl_3$) spectrum of compound **1bl**

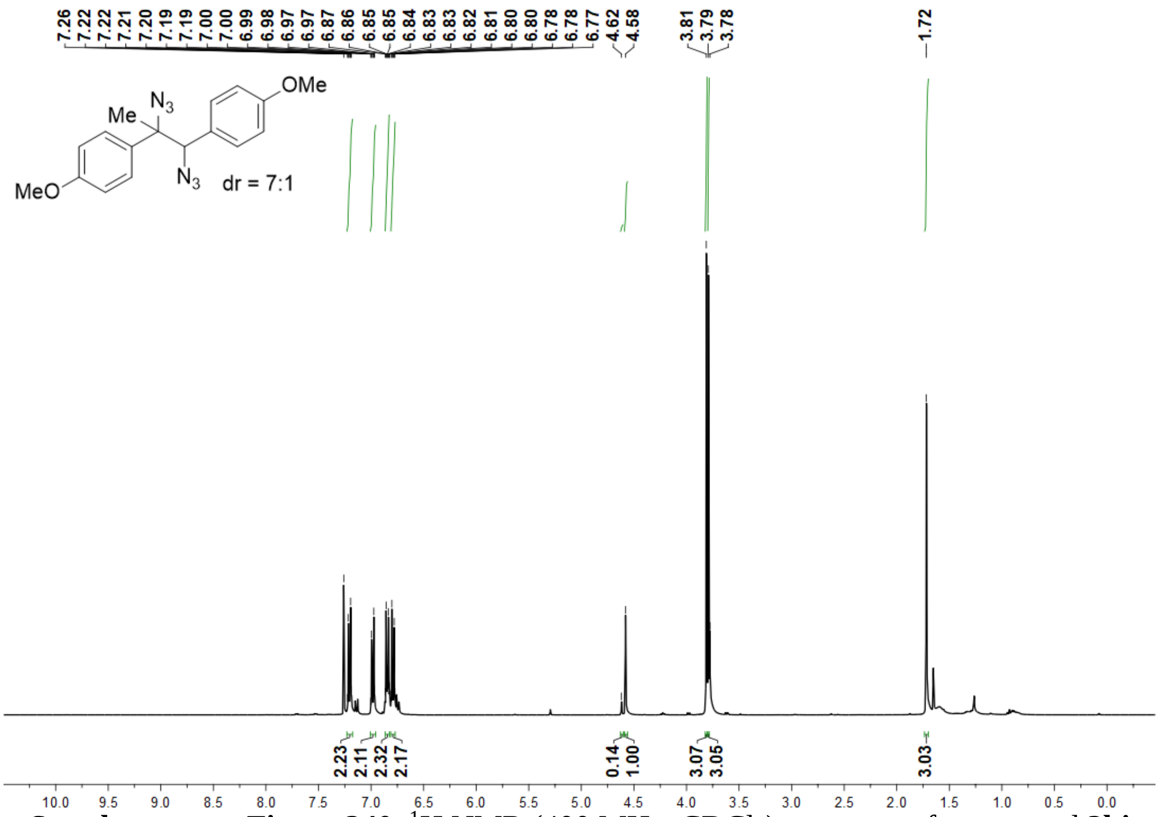

**Supplementary Figure 240.** $^{1}H$ NMR (400 MHz, $CDCl_3$) spectrum of compound **3bi**

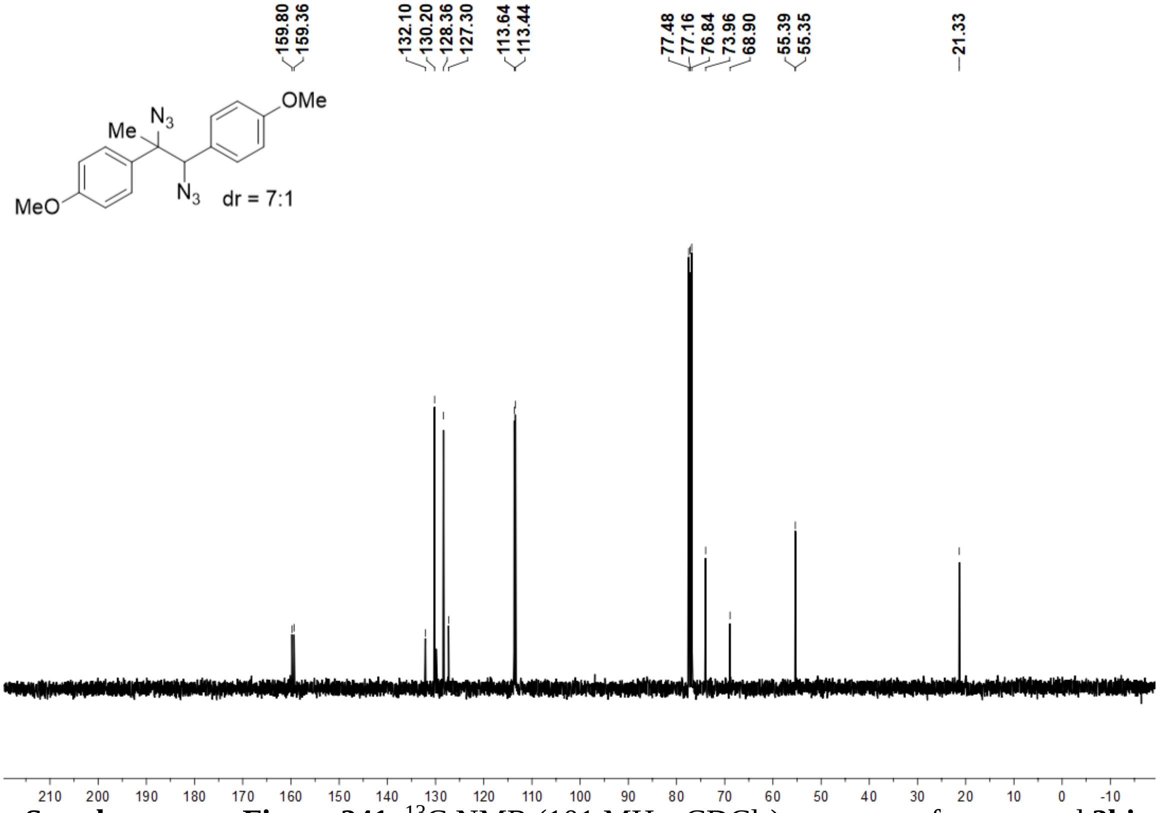

**Supplementary Figure 241.** $^{13}C$ NMR (101 MHz, $CDCl_3$) spectrum of compound **3bi**

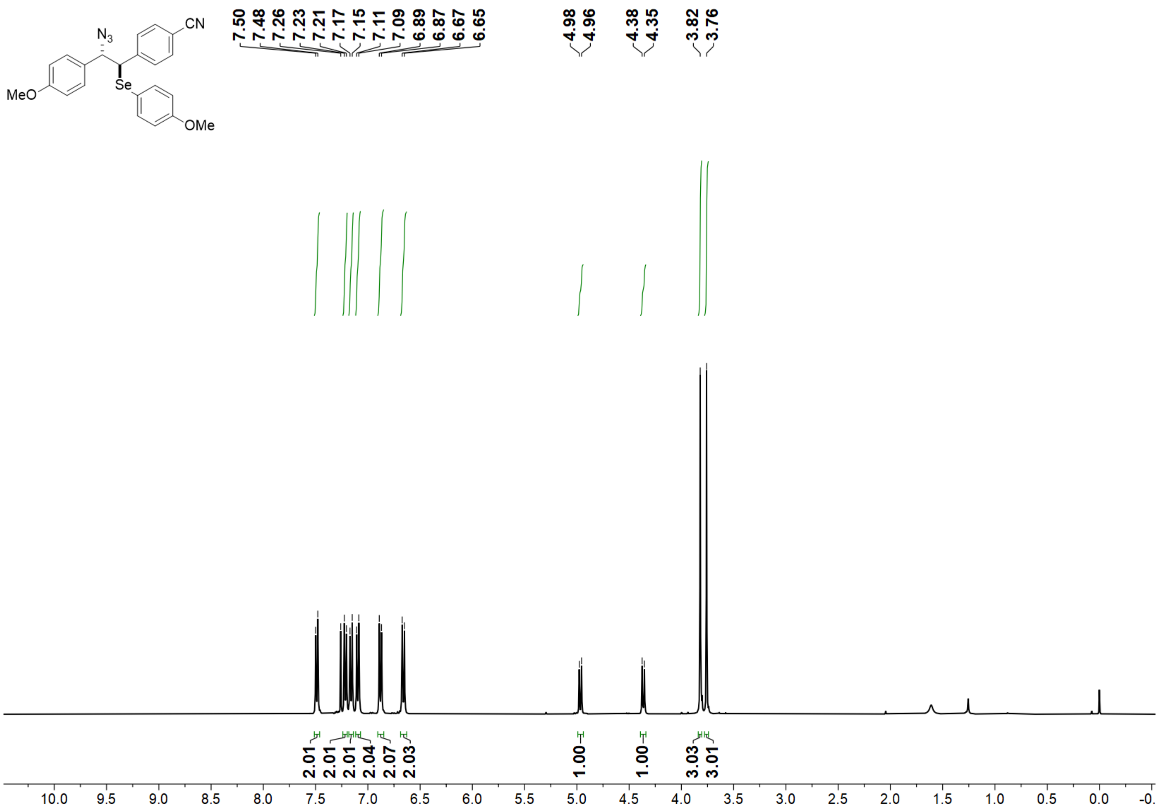


**Supplementary Figure 242.** $^{1}$H NMR (400 MHz, $CDCl_3$) spectrum of compound **7**

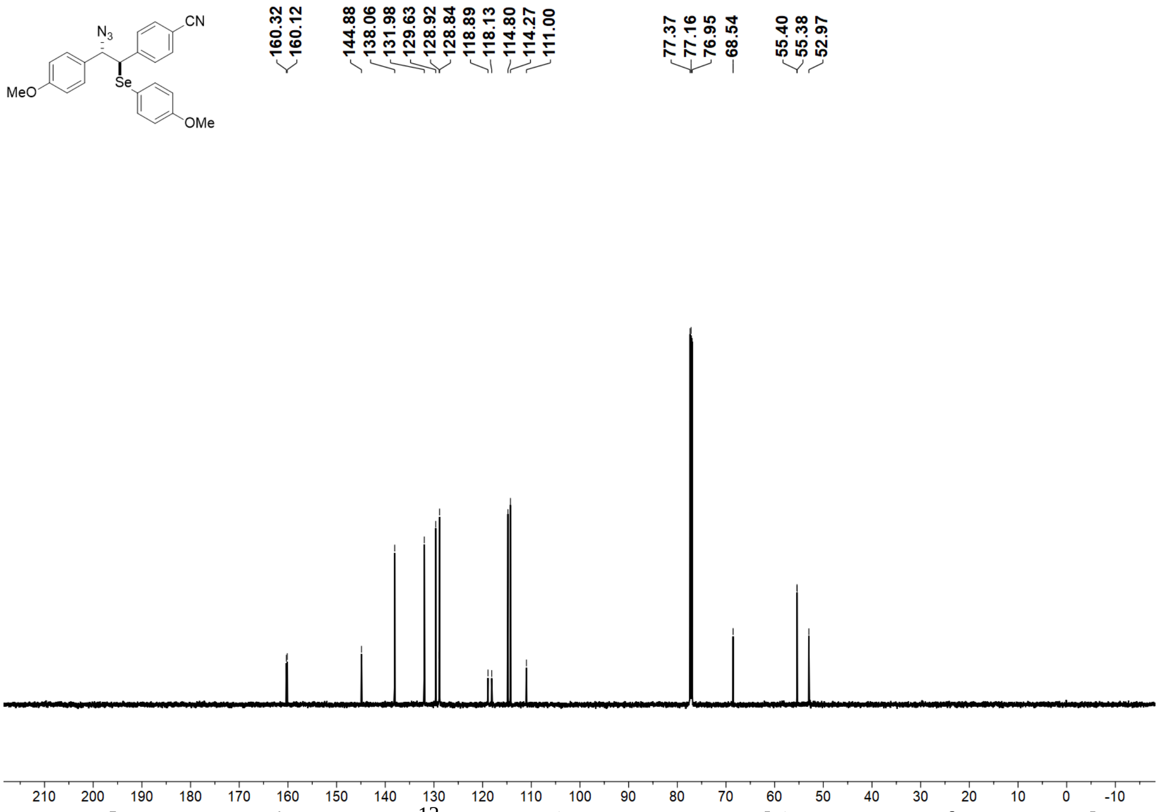


**Supplementary Figure 243.** $^{13}$C NMR (151 MHz, $CDCl_3$) spectrum of compound **7**

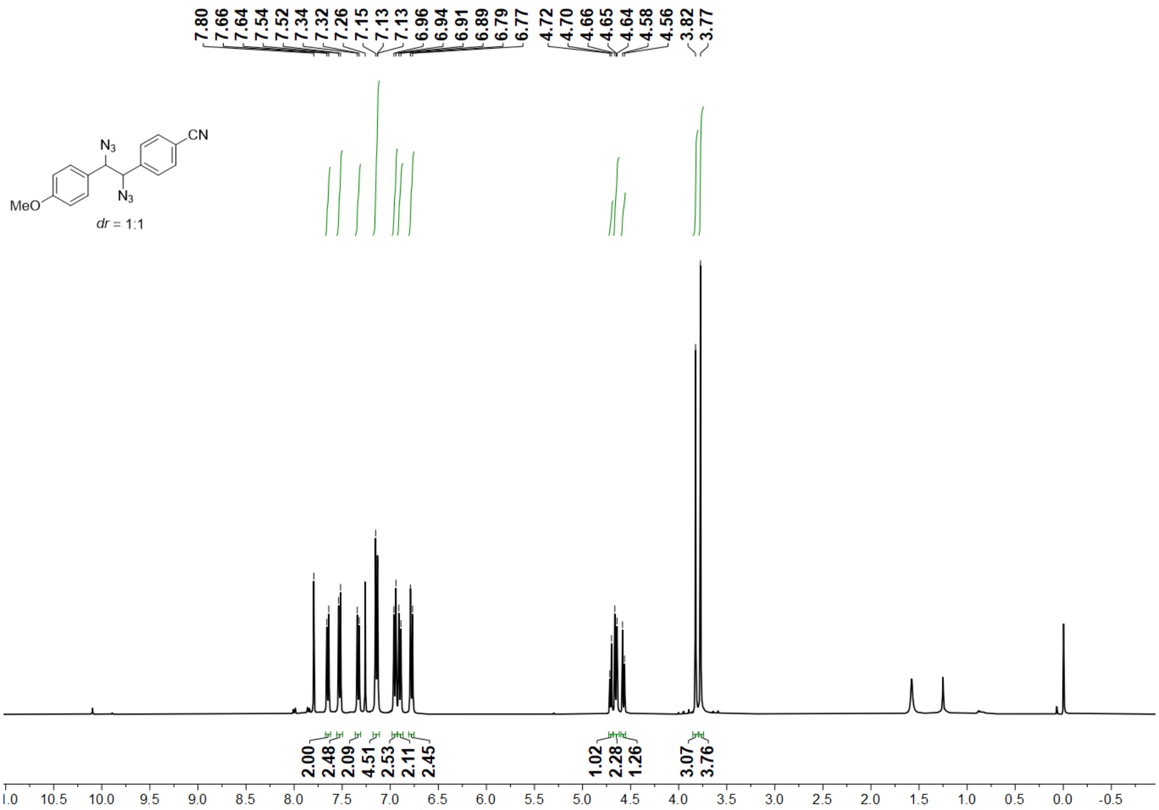

**Supplementary Figure 244.** $^{1}$H NMR (400 MHz, $CDCl_3$) spectrum of compound **3a**

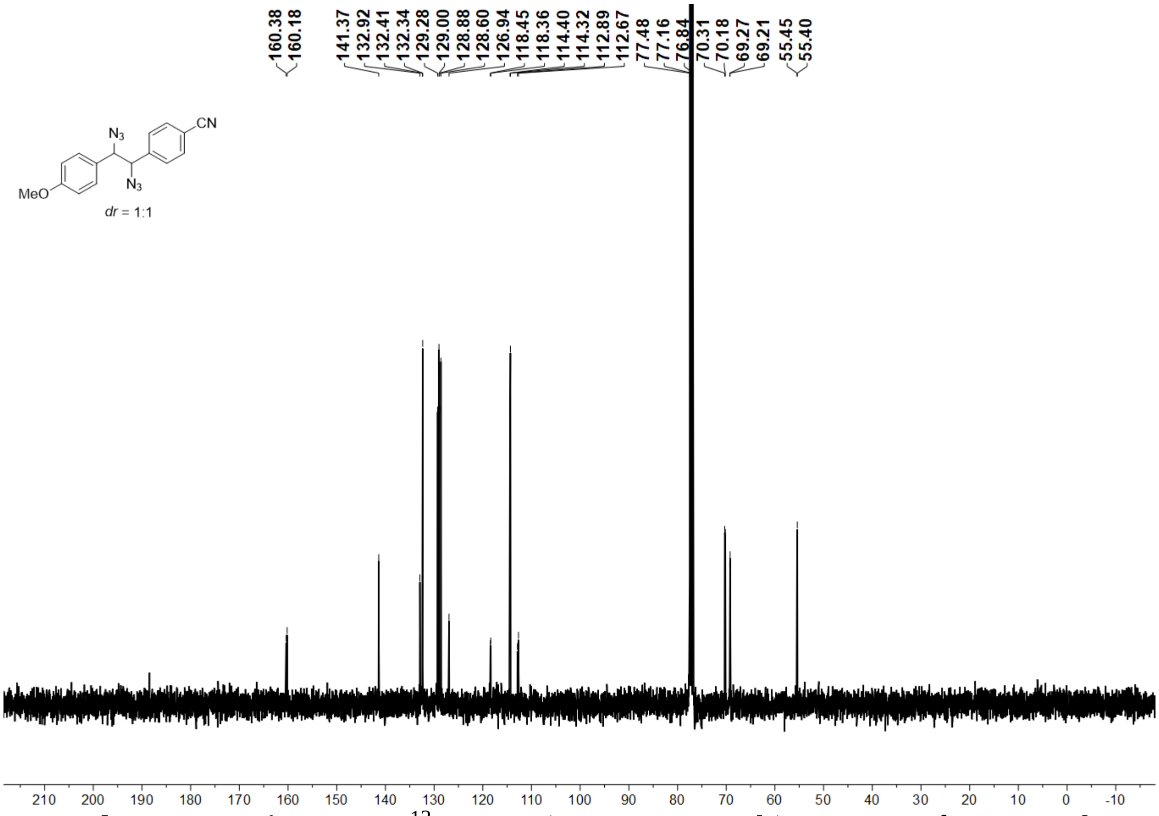

**Supplementary Figure 245.** $^{13}$C NMR (101 MHz, $CDCl_3$) spectrum of compound **3a**

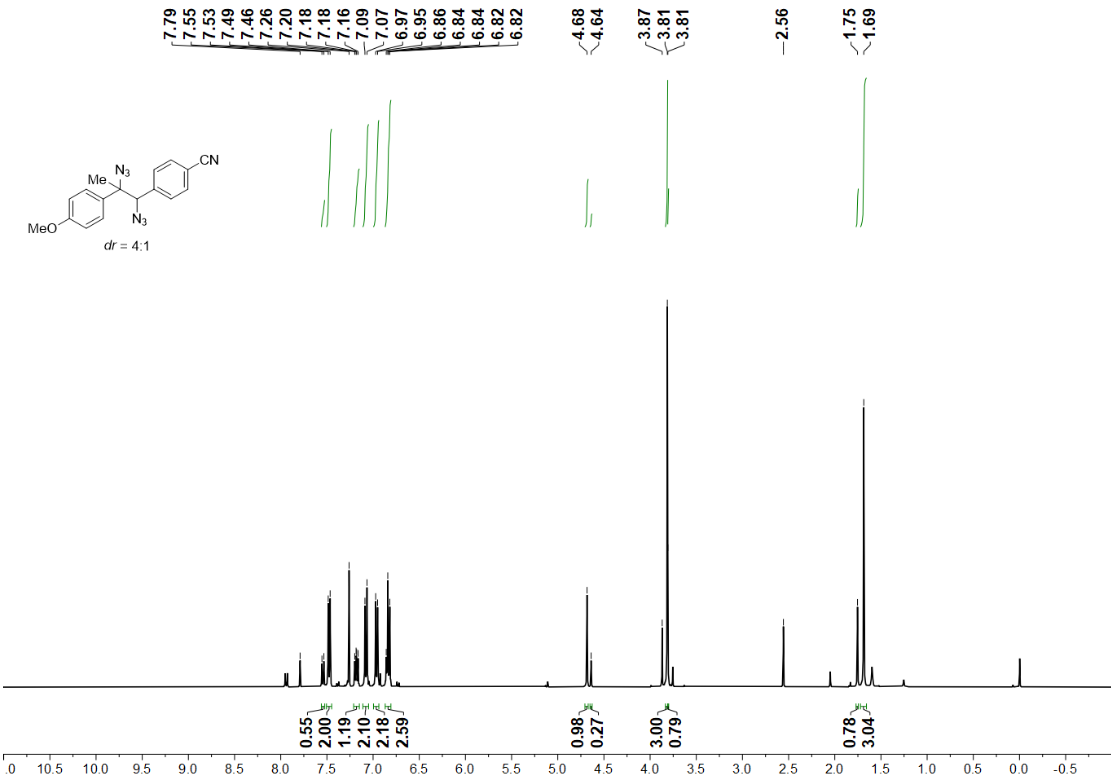

**Supplementary Figure 246.** $^{1}H$ NMR (400 MHz, $CDCl_3$) spectrum of compound **3v**

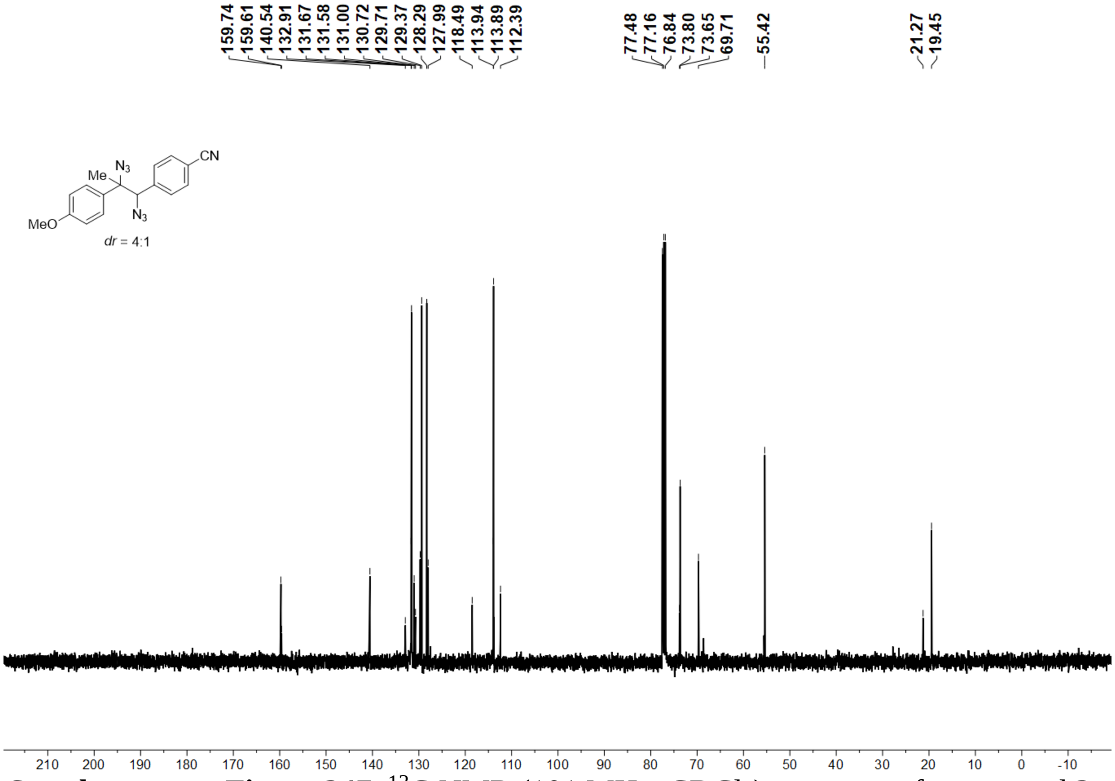

**Supplementary Figure 247.** $^{13}C$ NMR (101 MHz, $CDCl_3$) spectrum of compound **3v**

## 9. Supplementary References


1. Agarrabeitia, A. R. et al. Remarkable observations on triplet-sensitized reactions. The di-π-methane rearrangement of acyclic 1,4-dienes in the triplet excited state. *Org. Lett.* **11**, 4148-4151 **(**2009).
2. Dong, D.-J., Li, H.-H. & Tian, S.-K. A highly tunable stereoselective olefination of semistabilized triphenylphosphonium ylides with *N*-sulfonyl imines. *J. Am. Chem. Soc.* **132**, 5018-5020 (2010).
3. Luo, J. et al. Controlled selectivity through reversible inhibition of the catalyst: stereodivergent semihydrogenation of alkynes. *J. Am. Chem. Soc.* **144**, 13266-13275 (2022).
4. Hong, F., Low, Y., Chong, K., Thomas, N. F. & Kam, T. Biomimetic oxidative dimerization of anodically generated stilbene radical cations: effect of aromatic substitution on product distribution and reaction pathways. *J. Org. Chem.* **79**, 4528-4543 (2014).
5. Shafiee, A., Kassaee, M. Z. & Bekhradnia, A. R. Synthesis of novel 3,4-diaryl-1*H*-pyrroles. *J. Heterocyclic Chem.* **44**, 471-474 (2007).
6. Hammann, J. M., Lutter, F. H., Haas, D. & Knochel, P. A robust and broadly applicable cobalt-catalyzed cross-coupling of functionalized bench-stable organozinc pivalates with unsaturated halides. *Angew. Chem. Int. Ed.* **56**, 1082-1086 (2017).
7. Aukland, M. H. et al. An interrupted pummerer/nickel-catalysed cross-coupling sequence. *Angew. Chem. Int. Ed***.** **57**, 9785-9789 (2018).
8. Flaherty, D. P., Dong, Y. & Vennerstrom, J. L. A one-pot synthesis of unsymmetrical bis-styrylbenzenes. *Tetrahedron Lett.* **50**, 6228-6230 (2009).
9. Yang, X., Jin, X. & Wang, C. Manganese-catalyzed *ortho*-C-H alkenylation of aromatic N-H imidates with alkynes: versatile access to *mono*-alkenylated aromatic nitriles. *Adv. Synth. Catal.* **358**, 2436-2442 (2016).
10. Duan, Y.-C. et al. Discovery of resveratrol derivatives as novel $LSD_1$ inhibitors: design, synthesis and their biological evaluation. *Eur. J. Med. Chem.* **126**, 246-258 (2017).
11. Kormos, C. M. & Leadbeater, N. E. Preparation of nonsymmetrically substituted stilbenes in a one-pot two-step heck strategy using ethene as a reagent. *J. Org. Chem.*

**73**, 3854-3858 (2008).

12. Colbon, P. et al. Feeding the heck reaction with alcohol: one-pot synthesis of stilbenes from aryl alcohols and bromides. *Adv. Synth. Catal.* **354**, 1395-1400 (2012).
13. Ekebergh, A., Begon, R. & Kann, N. Ruthenium-catalyzed *E*-selective alkyne semihydrogenation with alcohols as hydrogen donors. *J. Org. Chem.* **85**, 2966-2975 (2020).
14. Lee, G. S., Kim, D. & Hong, S. H. Pd-catalyzed formal mizoroki-heck coupling of unactivated alkyl chlorides. *Nat. Commun.* **12**, 991-1002 (2021).
15. Scheidt, F., Neufeld, J., Schäfer, M., Thiehoff, C. & Gilmour, R. Catalytic *geminal* difluorination of styrenes for the construction of fluorine-rich bioisosteres. *Org. Lett.* **20**, 8073-8076 (2018).
16. Rerkrachaneekorn, T., Tankam, T., Sukwattanasinitt, M. & Wacharasindhu, S. NaI-mediated oxidative amidation of benzyl alcohols/aromatic aldehydes to benzamides via electrochemical reaction. *Tetrahedron Lett.* **70**, 153017-153022 (2021).
17. Eskandari, R., Hess, J. P. & Tochtrop, G. P. Synthesis of *α,β*-unsaturated epoxy ketones utilizing a bifunctional sulfonium/phosphonium ylide. *Chem. Commun.* **57**, 7136-7139 (2021).
18. Shin, J. H., Seong, E. Y., Mun, H. J., Jang, Y. J. & Kang, E. J. Electronically mismatched cycloaddition reactions via first-row transition metal, iron(III)–polypyridyl complex. *Org. Lett.* **20**, 5872-5876 (2018).
19. Hulvat, J. F., Sofos, M., Tajima, K. & Stupp, S. I. Self-assembly and luminescence of oligo (*p*-phenylene vinylene) amphiphiles. *J. Am. Chem. Soc.* **127**, 366-372 (2005).
20. Sivadas, A. P. et al. Supergelation via purely aromatic *π–π* driven self-assembly of pseudodiscotic oxadiazole mesogens. *J. Am. Chem. Soc.* **136**, 5416-5423 (2014).
21. Cao, C.-T., Yuan, H., Zhu, Q. & Cao, C. Determining the excited-state substituent constants $\sigma^{ex}_{CC(o)}$ of ortho-substituents from 2,4′-disubstituted stilbenes. *J. Phys. Org. Chem.* **32**, 3962-3968 (2019).
22. Chandrika, N. T., Shrestha, S. K., Ngo, H. X. & Garneau-Tsodikov, S. Synthesis and investigation of novel benzimidazole derivatives as antifungal agents. *Bioorgan. Med. Chem.* **24**, 3680-3686 (2016).

23. Liao, L., Xu, X., Ji, J. & Zhao, X. Asymmetric intermolecular iodinative difunctionalization of allylic sulfonamides enabled by organosulfide catalysis: modular entry to iodinated chiral molecules. *J. Am. Chem. Soc.* **144**, 16490-16501 (2022).
24. Singh, R. R. & Liu, R.-S. Gold-catalyzed 1,2-iminonitronation of electron-deficient alkynes with nitrosoarenes to afford *α*-imidoyl nitrones. *Chem. Commun.* **50**, 15864-15866 (2014).
25. Long, Y. et al. Discovery of novel 4-azaaryl-*N*-phenylpyrimidin-2-amine derivatives as potent and selective FLT3 Inhibitors for acute myeloid leukaemia with *FLT3* mutations. *Eur. J. Med. Chem.* **213**, 113215-113222 (2021).
26. Liu, Y. et al. Designed compounds for recognition of 10 base pairs of DNA with two AT binding sites. *J. Am. Chem. Soc.* **134**, 5290-5299 (2012).
27. Kungurtsev, V., Laakkonen, J., Molina, A. G. & Virta, P. Solution-phase synthesis of short oligo-2'-deoxyribonucleotides by using clustered nucleosides as a soluble support. *Eur. J. Org. Chem.* **2013**, 6687-6693 (2013).
28. Liu, Y. et al. Photosensitized [2+2]-cycloadditions of alkenylboronates and alkenes. *Angew. Chem. Int. Ed.* **61**, e2022007 (2022).
29. Cao, C., Cao, C. & Qu, J. Quantifying and fine adjusting the solid-state fluorescence wavelength of 1-thienyl-2-arylethylene. *J. Lumin.* **233**, 117895-117901 (2021).
30. Nishizawa, T., Lim, H. K., Tajima, K. & Hashimoto, K. Highly uniaxial orientation in oligo(*p*-phenylenevinylene) films induced during wet-coating process. *J. Am. Chem. Soc.* **131**, 2464-2465 (2009).
31. Dow AgroSciences LLC, United States. 3-(2-Arylcycloprop-1-ylcarbonylamino)benzamide Derivatives Having Pesticidal Utility, and Intermediates, Composition, and Processes, Related Thereto. A provisional patent (application no. WO2016-US26409) 2016-10-20.
32. Liao, L. et al. Catalytic access to functionalized allylic *gem*-difluorides via fluorinative meyer–schuster-like rearrangement. *Angew. Chem. Int. Ed.* **59**, 11010-11019 (2020).
33. Aukland, M. H. et al. An interrupted pummerer/nickel-catalysed cross-coupling sequence. *Angew. Chem. Int. Ed.* **57**, 9785-9789 (2018).

34. Hossian, A., Bhunia, S. K. & Jana, R. Substrate-dependent mechanistic divergence in decarboxylative heck reaction at room temperature. *J. Org. Chem.* **81**, 2521-2533 (2016).
35. Meng, G. & Szostak, M. Palladium-catalyzed suzuki-miyaura coupling of amides by carbon-nitrogen cleavage: general strategy for amide N-C bond activation. *Org. Biomol. Chem.* **14**, 5690-5707 (2016).
36. He, Z., Song, F., Sun, H. & Huang, Y. Transition-metal-free suzuki-type cross-coupling reaction of benzyl halides and boronic acids via 1,2-metalate shift. *J. Am. Chem. Soc.* **140**, 2693-2699 (2018).
37. Saito, T., Nishimoto, Y., Yasuda, M. & Baba, A. Direct coupling reaction between alcohols and silyl compounds: enhancement of lewis acidity of $Me_3SiBr$ using $InCl_3$. *J. Org. Chem.* **71**, 8516-8522 (2006).
38. Zhang, X., Guo, R. & Zhao, X. Organoselenium-catalyzed synthesis of indoles through intramolecular C–H amination. *Org. Chem. Front.* **2**, 1334-1337 (2015).
39. Hiba, K., Krishna, G. A., Prathapan, S. & Sreekumar, K. Palladium loaded dendronized polymer as efcient polymeric sustainable catalyst for heck coupling reaction. *Catal. Lett.* **152**, 1819-1834 (2022).
40. Rele, S., Talukdar, S., Banerji, A. & Chattopadhyay, S. Generation of reactive low-valent titanium species using metal-arenes as efficient organic reductants for $TiCl_3$: applications to organic synthesis. *J. Org. Chem.* **66**, 2990-2994 (2001).
41. Huang, T., Chen, T. & Han, L.-B. Oxidative dephosphorylation of benzylic phosphonates with dioxygen generating symmetrical *trans*-stilbenes. *J. Org. Chem.* **83**, 2959-2965 (2018).
42. Das, M. et al. Stereoselective peterson olefinations from bench-stable reagents and *N*-phenyl imines. *Chem. Eur. J.* **21**, 8737-8740 (2015).
43. Yan, X., Liu, H., Wei, S. & Huang, H. Catalytic claisen rearrangement by intercepting ketenimines with propargylic alcohols: a strategy to generate and transform ketenimines from radicals. *Org. Lett.* **22**, 6794-6798 (2020).
44. Li, W.-T., Hu, M.-Y., Xiong, J.-W., Zhang, X.-Y. & Zhu, S.-F. Iron-catalysed hydroalumination of internal alkynes. *Chem. Sci.* **13**, 7873-7879 (2022).
45. Zhu, G.; Kong, W.; Feng, H. & Qian, Z. Synthesis of (*Z*)-1-thio- and

(*Z*)-2-thio-1-alkenyl boronates via copper-catalyzed regiodivergent hydroboration of thioacetylenes: an experimental and theoretical study. *J. Org. Chem.* **79**, 1786-1795 (2014).

46. Wen, W., Shi, Z.-F., Cao, X.-P. & Xu, N.-S. Triphenylethylene-based fluorophores: facile preparation and fullcolor emission in both solution and solid states. *Dyes Pigments* **132**, 282-290 (2016).
47. Ojha, D. P. & Prabhu, K. R. Palladium catalyzed coupling of tosylhydrazones with aryl and heteroaryl halides in the absence of external ligands: synthesis of substituted olefins. *J. Org. Chem.* **77**, 11027-11033 (2012).
48. Sha, Q., Ling, Y., Wang, W. & Wei, Y. Capture of *in situ* generated diazo compounds or copper carbenoids by triphenylphosphine: selective synthesis of *trans*-alkenes and unsymmetric azines *via* reaction of aldehydes with ketone-derived *N*-tosylhydrazones. *Adv. Synth. Catal.* **355**, 2145-2150 (2013).
49. Liu, C.-F. et al. Olefin functionalization/isomerization enables stereoselective alkene synthesis. *Nat. Catal.* **4**, 674-683 (2021).
50. Kapat, A., K€onig, A., Montermini, F. & Renaud, P. A radical procedure for the anti-markovnikov hydroazidation of alkenes. *J. Am. Chem. Soc.* **133**, 13890-13893 (2011).
51. Regier, J., Maillet, R. & Bolshan, Y. A direct brønsted acid-catalyzed azidation of benzhydrols and carbohydrates. *Eur. J. Org. Chem.* **13**, 2390-2396 (2019).
52. Xue, F., Zhao, J. & Hor T. S. A. Ambient arylmagnesiation of alkynes catalysed by ligandless nickel(II). *Chem. Commun.* **49**, 10121-10123 (2013).
53. Ray, R. et al. Ligand controlled switchable selectivity in ruthenium catalyzed aerobic oxidation of primary amines. *Chem. Commun.* **53**, 4006-4009 (2017).
54. Havare, N. & Plattner, D. A. Oxidative cleavage of $\alpha$-aryl aldehydes using iodosylbenzene. *Org. Lett.* **14**, 5078-5081 (2012).
55. Gurawa, A., Kumar, M. & Kashyap, S. Selective azidooxygenation of alkenes enabled by photo-induced radical transfer using aryl-$\lambda^3$-azidoiodane species. *ACS Omega* **6**, 26623-26639 (2021).
56. Zhou, H. et al. Copper-catalyzed ligand-free diazidation of olefins with $TMSN_3$ in $CH_3CN$ or in $H_2O$. *Org. Lett.* **19**, 6120-6123 (2017).

57. Dolomanov, O. V., Bourhis, L. J., Gildea, R. J., Howard, J. A. K. & Puschmann, H. OLEX2: a complete structure solution, refinement and analysis program. *J. Appl. Cryst.* **42**, 339- 341 (2009).

58. Sheldrick, G. M. SHELXT-integrated space-group and crystal-structure determination. *Acta Cryst.* **A71**, 3-8 (2015).

59. Sheldrick, G. M. Crystal structure refinement with SHELXL. *Acta Cryst.* **C71**, 3-8 **(**2015).
